# Potential microRNA-mediated oncogenic intercellular communication revealed by pan-cancer analysis

**DOI:** 10.1038/srep07097

**Published:** 2014-11-18

**Authors:** Yue Li, Zhaolei Zhang

**Affiliations:** 1 Department of Computer Science, University of Toronto, Toronto, Ontario M5S 3G4, Canada; 2 The Donnelly Centre, University of Toronto, Toronto, Ontario M5S 3E1, Canada; 3 Department of Molecular Genetics, University of Toronto, Toronto, Ontario M5S 1A8, Canada; 4 Banting and Best Department of Medical Research, University of Toronto, Toronto, Ontario M5S 3E1, Canada

## Abstract

Carcinogenesis consists of oncogenesis and metastasis, and intriguingly microRNAs (miRNAs) are involved in both processes. Although aberrant miRNA activities are prevalent in diverse tumor types, the exact mechanisms for how they regulate cancerous processes are not always clear. To this end, we performed a large-scale pan-cancer analysis via a novel probabilistic approach to infer recurrent miRNA-target interactions implicated in 12 cancer types using data from The Cancer Genome Atlas. We discovered ~20,000 recurrent miRNA regulations, which are enriched for cancer-related miRNAs/genes. Notably, miRNA 200 family (miR-200/141/429) is among the most prominent miRNA regulators, which is known to be involved in metastasis. Importantly, the recurrent miRNA regulatory network is not only enriched for cancer pathways but also for extracellular matrix (ECM) organization and ECM-receptor interactions. The results suggest an intriguing cancer mechanism involving miRNA-mediated cell-to-cell communication, which possibly involves delivery of tumorigenic miRNA messengers to adjacent cells via exosomes. Finally, survival analysis revealed 414 recurrent-prognostic associations, where both gene and miRNA involved in each interaction conferred significant prognostic power in one or more cancer types. Together, our comprehensive pan-cancer analysis provided not only biological insights into metastasis but also brought to bear the clinical relevance of the proposed recurrent miRNA-gene associations.

Cancer is a complex disease implicated by various molecular abnormalities at the structural and expression level of both coding and non-coding genes^1^. While the alterations of protein-coding oncogenes and/or tumour-suppressor genes have long been considered as the causal variants for oncogenesis, exact molecular mechanisms on how the tumorigenic signals propagate from tumor cells to adjacent cells are often unclear. A crucial clue may lie in a class of endogenous 20–22 nucleotide non-coding RNA species called *microRNA* (miRNA)^1^,^2^. Since the discovery of the first miRNA *let-7* in *Caenorhabditis elegans*^3^ in 1993, a vast amount of studies have been dedicated to functionally characterizing miRNAs with a special emphasis on their roles in cancer^4^,^5^,^6^,^7^,^8^. On the other hand, it has proved difficult to identify miRNA-target interactions in mammals based on sequence features alone primarily due to the imperfect base-pairings between miRNAs and the target genes^2^. Some of the earlier popular prediction methods such as TargetScan^9^ and miRanda^10^ are based on sequence complementarity between the 2–7 nucleotide positions termed as the “seed region” of the miRNAs and the 3′UTR of the target genes. However, many non-canonical interactions also exist. For instance, a recent technology called CLASH (cross-linking, ligation, and sequencing of hybrids)^11^ and a similar protocol^12^ that directly interrogate the genome-wide physical interactions between miRNA and RNA targets revealed many interactions occurring outside of the seed regions in human and worm. Furthermore, these new data revealed that over 40% (and only about 23%) of the physical interactions in fact occurred at the coding regions (and 3′UTRs) of the target genes^11^,^12^.

Nonetheless, physical bindings are not necessarily functional *in vivo*. To explore miRNA regulatory landscape implicated in cancer, we need to take into account both the miRNA and target gene expression profiles. In this regard, The Cancer Genome Atlas (TCGA) Research Network has generated an unprecedented large amount of high quality paired miRNA and gene expression profiles along with other genomic and epigenetic measurements for thousands of serous and epithelial specimens across diverse tumor types^13^. Importantly, the consortium raised the notion of *pan-cancer* to identify molecular aberrations that transcend multiple cancer lineages^14^. MiRNAs often repress target gene expression by inducing RNA degradation^15^. Identifying pervasive negative expression correlation resulted from specific miRNA-target interactions across all cancer types may provide functional insights to the oncogenic mechanism. However, there is a current lack of a systematic approach to infer the recurrent miRNA-mRNA associations while taking into account the systematic biases due to different experimental conditions, sample heterogeneity, sample size, and many other non-uniform aspects that make the pan-cancer analysis particularly challenging.

Recently, Jacobsen *et al.* (2013) developed a rank-based method called recurrence (REC) to evaluate the recurrent interactions across 11 cancer types using TCGA data^6^. The miRNA-target interaction strength in individual cancer type is estimated by the corresponding coefficients in a multivariate linear regression model, which also takes into account the biases in estimating target expression changes due to the corresponding copy number and DNA methylation changes. Essentially, a specific miRNA-target pair that is ranked high in terms of its regression coefficients among all cancer types will confer a high REC score. Since the rank-based approach is scale-free, REC avoids direct comparisons between cancer types, which can be misleading due to the aforementioned systematic biases inherent in each cancer dataset. Notably, however, the REC score is derived from a *χ*^2^ approximation, and the magnitude of the score is arbitrary. Perhaps a more natural way to evaluate the statistical confidence of the recurrent interactions is by inferring the corresponding posterior probabilities. In this regard, a related but not directly applicable method called MCMG (Multiple Cancer for MicroRNA-Gene interactions) employs an empirical Bayes approach to infer the posterior probability of the interaction within a specific cancer by borrowing information from other cancer types in order to estimate the prior distribution for the interactions^7^.

In this study, we developed a probabilistic approach called PanMiRa (Pan-cancer MiRNA-target Associations) to infer recurrent miRNA-target interactions across 12 cancer types from TCGA. The major innovation in our method in comparison to previously published methods is its Bayesian inference of the posterior distribution of the recurrent interactions after taking into consideration the confounding effects from copy number and DNA methylation. Sequence-based feature scores, known interactions, CLASH-detected chimera, and miRNA perturbation data all corroborate the ~20,000 positive recurrent interactions identified by PanMiRa. Moreover, PanMiRa predictions are enriched for oncomirs and oncogenes, implying their functional relevance in cancer biology. Intriguingly, we find that the targets involved in the 1480 high-confidence pan-cancer interactions are not only enriched for cancer pathways such as TGF-*β* signalling pathways but also pathways related to extracellular matrix (ECM) organization, focal adhesion, and ECM-receptor interactions, all of which are essential pathways in oncogenesis. A natural way to interpret these results is perhaps that miRNA may mediate cell-to-cell communication through modulating the expression of the ECM-constituents to facilitate tumor invasion during metastasis. Moreover, survival analysis revealed 414 prognostic-recurrent interactions, some of which are also associated with ECM organization and intercellular interaction. Together, the large pan-cancer atlas and the novel pan-cancer inference method provide a valuable resource for the scientific community. Finally, we further postulate that the hypothesized miRNA-mediated intercellular communication may involve extracellular RNA (also known as exosomal RNA or abbreviated as exRNA) transfer mechanism^16^, where the extracellular vesicles manoeuvre through ECM to deliver tumorigenic miRNA messengers to the cells in vicinity.

## Results

### Pan-cancer data compendium construction across 12 cancer types

We constructed a large pan-cancer data compendium from TCGA, which is by far the most comprehensive data resource, providing paired mRNA and miRNA (m/miRNA) expression data as well as copy number (CN) and DNA methylation (DM) data measured for the same sample across diverse cancer types^13^. Here we chose 12 cancer types from the TCGA Data Portal based on the availability of the above-mentioned molecular measurements for at least 100 samples per cancer type. For each cancer type, we downloaded the processed long and short RNA-seq data. We then filtered the target RNAs or miRNAs by non-zero expression level in at least 5% of the samples within the same cancer type. Additionally, we required that each target RNA must also have CN measurements for the same sample, which are the processed SNP array data from Broad Institute-Firehose^17^. Moreover, we obtained the processed DM microarray data measured for each target RNA, and retained the probes at the promoter regions by their negative correlations with the target RNA expression level. Finally, we merged the data from the 12 cancer types to form a pan-cancer data compendium containing 4,258 samples, each having the expression, CN, and DM values for 17,788 target RNAs and expression levels for 677 miRNAs. Table 1 summarizes the pan-cancer data atlas. More details are described in Methods.

### Inferring recurrent miRNA-target interactions implicated by the pan-cancer data

**Figure 1:**
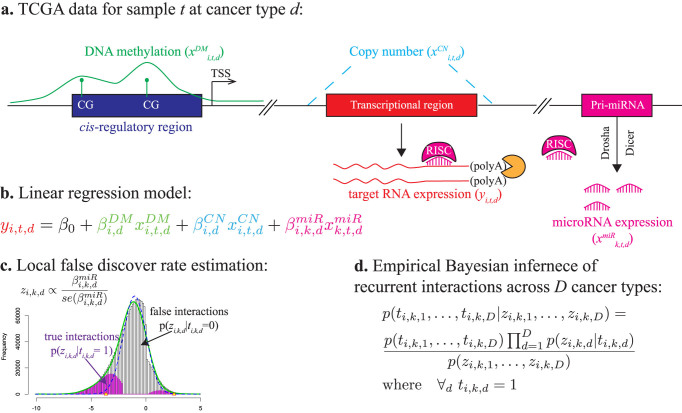
PanMiRa model schema. (a) Suppose target RNA expression (*y_i_*_,*t*,*d*_) in sample *t* of cancer type *d* is a function of DNA methylation (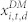), copy number (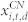) and miRNA regulation (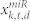). (b) The expression change across samples for the target RNA is modelled as the response variable in a multivariate linear regression framework using the input variables as indicted above. (c) The resulting linear coefficient 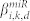 indicate the corresponding interaction between miRNA *k* and target gene *i* of cancer type *d* and are transformed into *z*-scores, which are then subsequently subjected to local false discovery rate (*locfdr*) estimation^18^. (d) The joint posteriors for the recurrent interactions given the *z*-scores are inferred by empirical Bayes using the probabilistic quantities obtained from the *locfdr* procedure above.

To reliably infer the distribution of recurrent miRNA-target interactions from the pan-cancer data compendium, we developed a novel Bayesian framework called PanMiRa (Pan-cancer MiRNA-target Associations). Fig. 1 illustrates the concept of the proposed model (detailed in Methods). Suppose the mRNA target expression level is a function of DM, CN, and miRNA regulation (Fig. 1a). To estimate the individual miRNA-target relationships, we employed a multivariate linear regression model, which was fitted to the observed target expression, taking into account the biases due to the baseline expression level, DM, and CN effects (Fig. 1b). The resulting linear coefficients are indicative of the corresponding miRNA-target relationships in the specific cancer type: the more negative they are the more likely the interactions are real. To translate the numerical coefficients into the language of probability, we first transformed the coefficients into *z*-scores and then estimated the corresponding local false discovery rate (*locfdr*) using the procedure developed by Efron (2004) (Fig. 1c). The technique was invented for large-scale simultaneous hypothesis testings by estimating the null distribution directly from the test statistics^18^. The resulting local FDR can be considered as an empirical Bayes version of the FDR derived from the popular Benjamini-Hochberg (BH) method^19^ and is in principle equivalent to the posterior probability of false interactions given the *z*-scores.

Additionally, the empirical distribution estimated from the *locfdr* approach is essentially the likelihood of *z*-scores given the underlying interaction status and enables estimating the joint posterior of the true interactions across the 12 cancer types via an empirical Bayes algorithm^7^ (Fig. 1d). For each interaction, we first assume a uniform prior for all possible combinations of its binary value over the 12 cancer types. In particular, each interaction can take on 2^12^ = 4096 possible combinations, and the initial prior is 1/4096 for each combination. Using Bayes rule and assuming the interactions at different cancer types are conditionally independent given their true interaction status, we can compute the posterior for each combination and re-estimate the prior by marginalizing the joint posteriors. We then alternate between the prior and posterior inference steps until little change occurs between the likelihoods at the current and previous iteration. The posteriors of interest associated with the recurrent interaction correspond to the particular binary combination, where the interaction status is positive across all of the 12 cancer types. From the pan-cancer compendium constructed above, PanMiRa identified 20,819 recurrent interactions with positive joint posterior among the ~1.8 million candidate interactions with negative *z*-scores between the corresponding miRNAs and target genes in at least 75% of the cancer types.

### Functional validation of the predicted recurrent interactions

**Figure 2:**
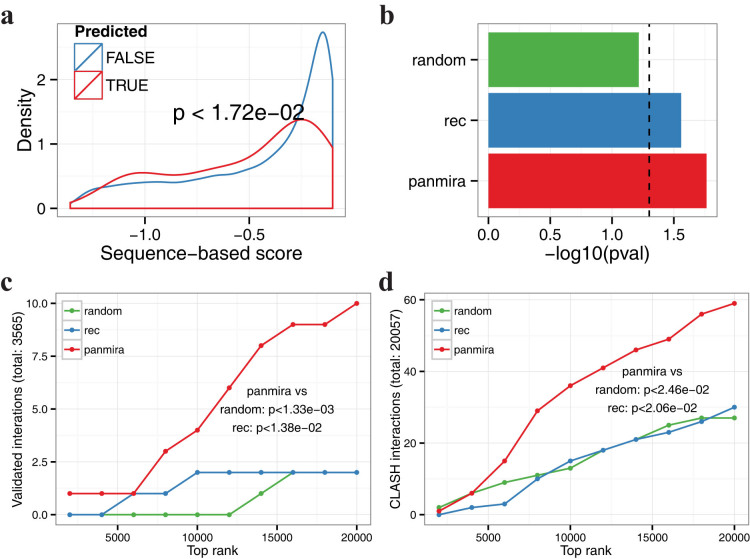
Validation of positive recurrent interactions. (a) The distribution of the sequence-based scores from miRanda-mirSVR are compared between the positive recurrent interactions predicted by PanMiRa and the remaining pairs. The more negative the scores the stronger implication of a true interaction based on the sequence-based features. P-value indicated above was computed by one-sided Wilcoxon rank-sum test. (b) The same test was performed for the interactions detected by randomly shuffled posteriors and REC. The resulting significance levels in terms of -log10(p-value) are compared among the three methods. The dash line indicates the significance cutoff of p-value = 0.05. (c) & (d) Validated interactions from miRTarBase and CLASH-detected chimera are counted as a function of the top rankings from 2000–20,000 with 2000-interval. P-values indicated above were computed by comparing PanMiRa with random/REC using Wilcoxon rank-sum test.

As a proof-of-principle, we used four types of complementary functional data to examine the validity of the 20,819 predicted recurrent interactions, each having a positive joint posterior probability (Supplementary Table S1): (1) sequence-based scores from miRanda-mirSVR^10^ and TargetScan^9^; (2) validated interactions from miRTarBase (release 4.5)^20^; (3) interactions detected by CLASH (cross-linking, ligation, and sequencing of hybrids)^11^ in HEK293 cell; (4) miRNA perturbation data extracted from public domains. As a comparison, we implemented the recently developed rank-based method called REC (RECurrence)^6^, which (to our knowledge) is the only published method to predict recurrent miRNA-target interactions, and we defined the positive interactions as having REC score < −1 (Methods). We also explored several more lenient/stringent REC cutoffs and observed similar or worse performances. First, we found that the positive interactions predicted by PanMiRa exhibit significantly higher sequence-based scores than the remaining non-recurrent interactions (*p* < 1.72 × 10^−2^; one-sided Wilcoxon rank-sum test), which together contributed to the general pool of ~1.8 million interactions with negative correlations in at least 75% of the cancer types (Fig. 2a). Supplementary Fig. S1 illustrates the full results for the analysis using the sequence-based predictions as a “gold-standard”. Additionally, the general interaction pool with randomized recurrent scores performed much poorer than the predicted recurrent interactions, further confirming that the improvement is due to the recurrence nature of the interactions (Fig. 2b). Notably, REC-predictions can also distinguish strong from weak sequence-based interactions whereas the overall statistical significance is lower when compared with PanMiRa-interactions (Fig. 2b; Supplementary Fig. S1). Thus, the unbiased pan-cancer predictions indeed accurately reflect the sequence-context features such as the canonical 2–7 position seed match, AU content, sequence conservation, etc^9^,^10^.

We then examined whether the PanMiRa-predicted interactions were more enriched for validated interactions from miRTarBase^20^ than random or REC-predicted pairs. Due to the paucity of the known interactions, however, conventional power analysis such as Receiver Operating Characteristic (ROC) curves is unable to discriminate method performances. Instead, we ranked the interactions based on the corresponding posteriors or REC scores and counted within the top 2000–20,000 interactions with 2000-interval the total number of validated interactions. As shown in Fig. 2c, PanMiRa predictions are significantly more enriched for the known interactions than random and REC-predicted pairs (*p* < 1.33 × 10^−3^, *p* < 1.38 × 10^−2^, respectively; Wilcoxon signed rank test). Similarly, we observed a significantly higher enrichment for interactions detected by CLASH when comparing PanMiRa with REC and randomly shuffled posteriors (*p* < 2.5 × 10^−2^, Wilcoxon signed rank test; Fig. 2d).

**Figure 3:**
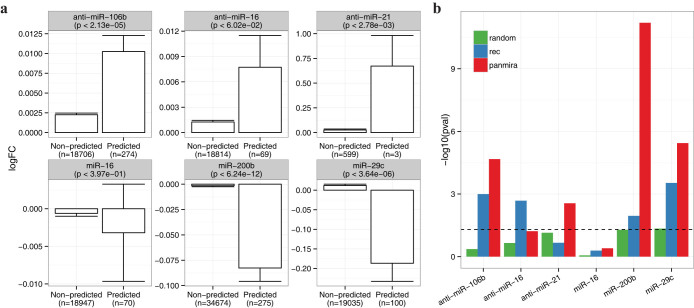
Functional validation using public miRNA perturbation data. We obtained from public domains six miRNA perturbation microarray datasets measuring the expression fold-changes followed by miRNA inhibition (anti-miR-106b/16/21) or miRNA overexpression (miR-16/200b/29c). (a) Average log fold-change due to a miRNA perturbation, where the p-values in the parentheses indicate the statistical significance from one-sided Wilcoxon rank-sum test and the number of non-predicted and predicted targets are indicated at the x-axis. Error bars indicate one standard deviations from the mean. (b) The same tests were performed for the interactions detected by randomly shuffled posteriors and REC. The statistical significance levels -log10(p-value) were compared among the three methods. The dash line indicate the standard cutoff of p-value = 0.05.

To further ascertain the functional implication of the pan-cancer miRNA-target interactions, we compared the expression fold-changes of the predicted targets with the remaining genes using publicly available microarray data for six miRNA perturbation experiments (Methods). Notably, three of the six experiments were done by knocking down (or overexpressing) the respective miRNAs using anti-mirs (or miRNA mimics) expecting an up-regulation (or down-regulation) of the true targets. Indeed, we observed an overall coherent expression changes for both the PanMiRa and REC-predicted targets (Fig. 3a), where four of the six tests passed the statistical significance test (*p* < 0.05, one-sided Wilcoxon rank-sum test). Notably, however, the targets predicted by PanMiRa exhibit more significant expression changes in four out of the six tests (Fig. 3b). Together, we show that the predicted pan-cancer interactions from the proposed model are corroborated by the sequence-based context and conservation scores, experimental validations, physical interactions from CLASH, the miRNA perturbation experiments, and therefore biologically plausible.

### Enrichment for cancer-related miRNAs and target genes in the recurrent interactions

**Figure 4:**
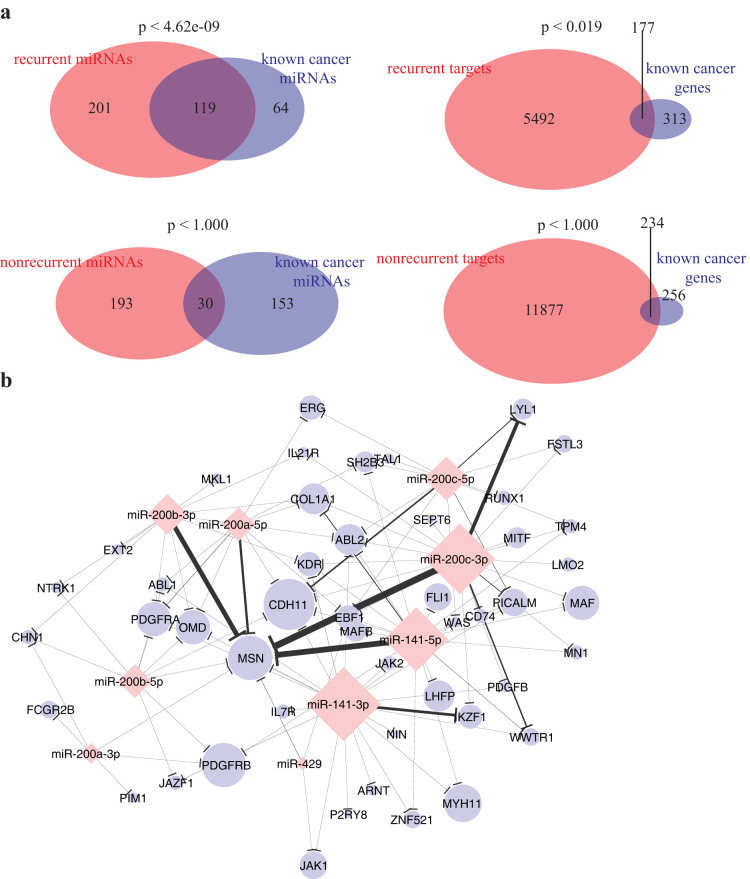
Recurrent miRNA-target network are enriched for oncomir and oncogenes. (a) Venn diagrams displaying the overlaps between the miRNA (left panel) or targets (right panel) involved (top) or not involved (bottom) in the recurrent network and the known cancer miRNAs^21^,^22^ or cancer genes^23^, respectively. P-values indicated above were computed by hypergeometric test. (b) Select recurrent oncomir-oncogene subnetwork involving miR-200 family (miR-200a/b/c/141/429) (red diamonds) and their target genes (blue ellipses). The node size and the edge width are proportional to the in/out-degree and recurrence posterior in the overall oncogene-oncomir network, respectively.

To explore the biological implications of the recurrent interactions in oncogenesis, we first examined whether the miRNAs and genes involved in the pan-cancer pairs are enriched for the known cancer miRNAs and genes, which are previously shown by independent studies to exhibit aberrant expression in tumors and denoted from now on as oncomir and oncogenes, respectively. To this end, we compiled cancer-related miRNAs from two separate sources^21^,^22^ and collected canonical cancer genes from COSMIC database^23^. Remarkably, the recurrent interactions are significantly enriched for both the oncomirs and oncogenes/tumor suppressors (*p* < 6.38 × 10^−8^, *p* < 0.019, respectively; hypergeometric test) (Fig. 4a). We confirmed that the enrichment for cancer related miRNAs and genes are also due to the recurrence nature by repeating the same enrichment analysis using genes or miRNAs involved in the non-recurrent interactions (i.e., excluding genes and miRNAs in the ~20,000 positive recurrent interactions) (Fig. 4a lower panel). Using only the recurrent interactions each involving both the oncomirs and oncogenes, we generated a cancer network and visualized it under Cytoscape^24^, where the size of the nodes were drawn proportional to their in/out-degree in the overall network, and the edge widths are proportional to the recurrence posterior probabilities (Supplementary Fig. S2). Importantly, we observed that a large number of pan-cancer interactions involve miR-200 miRNA family (MIPF0000019; miRBase release 21)^25^, which contains miR-200a/b/c, miR-141, and miR-429. Fig. 4b depicts the corresponding subnetwork. Growing evidences suggest that miR-200 miRNAs are involved in cell proliferation^26^ and cancer metastasis^27^,^28^. In particular, Nam *et al.* (2008) observed an up-regulation of the miR-200 family members in ovarian cancer^27^. Korpal *et al.* (2008) demonstrated that overexpressing each miR-200 family member in NMuMG murine mammary epithelial cells consistently inhibited epithelial-mesenchymal transition (EMT) and promoted mesenchymal-epithelial transition (MET) by de-repressing E-cadherin via targeting of ZEB1 and ZEB2, which are repressors of E-cadherin (but not oncogenes themselves)^28^. In line with these recent studies, our results suggest a prominent cancer-related role of the miR-200 family in not only regulating E-cadherin repressor ZEB1 (by miR-141-3p with posterior equal to 0.42; Supplementary Table S1) but also a large number of known oncogenes. Notably, most of the oncomir-oncogenes interactions remain to be validated, which calls for focused experimental studies.

### Target genes in the recurrent interactions are enriched for cancer-related pathways

**Figure 5:**
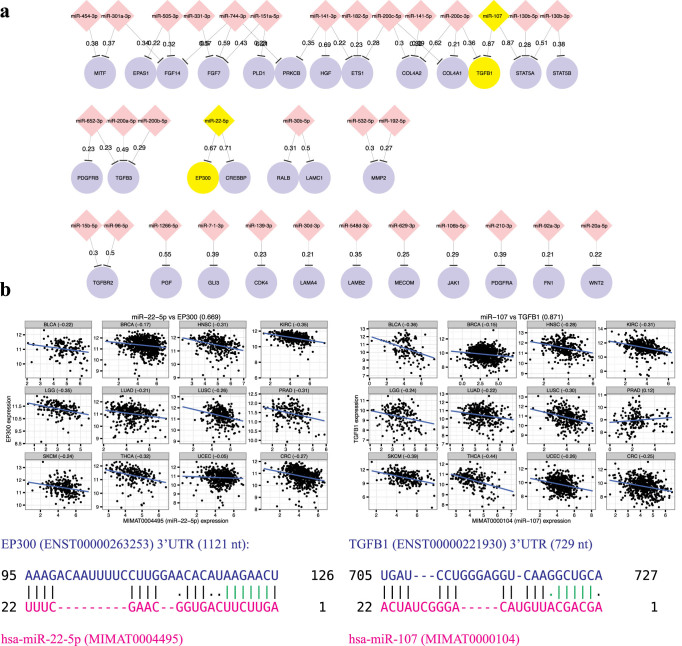
Confidence recurrent subnetwork involved in the KEGG pathways in cancer. (a) Pan-cancer miRNA-mRNA interactions with posteriors at least 0.2 were used to construct a confidence recurrent network and visualized via Cytoscape^24^. The subnetwork shown above involved target genes in the KEGG pathways in cancer (KEGG ID: hsa05200)^30^. The miRNAs and genes are displayed in red diamonds and blue ellipses, respectively, and the edge labels are the posterior probabilities. The yellow highlighted nodes are chosen as two detailed examples described below. (b) We generated the scatterplots to visualize the expression correlation between miR-22-5p and EP300 (left panel) and between miR-107 and TGFB1 (right panel). The corresponding base-paring between the mature miRNAs and the 3′UTR sequences displayed in the bottom were predicted by Smith-Waterman local alignment from EMBOSS suite^32^.

To gain mechanistic insights into the recurrent interaction effects in cancer, we performed a functional enrichment analysis by DAVID^29^ using the target genes involved in the 1480 high confidence recurrent interactions with posteriors at least 0.2. Indeed, we found that the target genes are significantly enriched for meaningful cancer-related processes or pathways such as regulation of endothelial cell proliferation (GO:0001936; *p* < 3.09 × 10^−04^), p53 binding (GO:0002039; *p* < 0.0022), TGF-*β* signalling pathways (hsa04350; *p* < 0.01), regulation of EMT (GO:0010717; *p* < 0.0011), and pathways in cancers (hsa05200; *p* < 0.0058) (Supplementary Table S2). Fig. 5a depicts the miRNAs (red diamond) and the target genes (purple circle) involved in the KEGG (Kyoto Encyclopedia of Genes and Genomes)^30^ pathways in cancer (hsa05200), where the edges are annotated with the corresponding posterior probabilities. Moreover, we generated 49 scattered plots each corresponding to a recurrent interaction involved in this pathway (Supplementary Fig. S3). As expected, we observed that the miRNA-target pairs indeed exhibit negative correlations pervasively across the 12 cancer types. Fig. 5b illustrates two of the 49 examples, namely miR-22-5p-EP300 and miR-107-TGFB1, whose recurrence posterior probabilities are 0.669 and 0.871, respectively.

For the former example, EP300 is a well-known oncogene^23^ and has been recently considered based on exome sequencing and RNA interference as one of the 28 postulated cancer-driver gene hubs, which exhibit distinctive expression pattern in the luminal and basal subtypes of the 16 breast cancer cell lines and 402 breast tumor samples^31^. However, to our best knowledge, the cancer recurrent interaction relationship between EP300 and miR-22-5p has not been previously elucidated. Perhaps one reason is that miR-22-5p is on the star strand (*) of the stem-loop precursor miRNA hairpin, which gives rise to the more conserved counterpart miR-22-3p (TargetScanHuman database 6.2)^9^. Nonetheless, Smith-Waterman local alignment by EMBOSS^32^ (default settings) between EP300 3′UTR sequence (Ensembl 75) and the mature miR-22-5p sequence (miRBase 21) revealed a canonical 6-mer seed match at the 2-7 position of the miRNA^2^ (green vertical lines in Fig. 5b left) plus the base-pairing at the first position.

For the latter example, TGFB1 (transforming growth factor beta 1) was predicted by our analysis to be regulated by the oncomir miR-107 (posterior: 0.871), which is supported by the negative correlation observed in 11 out of the 12 cancer types (Fig. 5b right). Notably, TGFB1 is also an oncogene^23^ and a putative cancer gene hub^31^. It is one of the TGF-*β* family ligands that bind to TFG-*β* type II receptor from outside the cell to initiate the well-known TGF-*β* signaling pathway, which is involved in tumor suppression through apoptosis but paradoxically also modulates cell proliferation, cell invasion, and immune regulation that tumor cells can take advantage of^33^. On the other hand, overexpression of miR-107 has been associated with metastastic potential of colorectal cancer (CRC) cell lines, poor prognosis in CRC patients^34^, and may function as tumor suppressor gene in Head and neck squamous cell carcinoma (HNSC)^35^. Notably, miR-107 and TGFB1 are negatively correlated in both CRC and HNSC in our pan-cancer TCGA data compendium along with other cancer types (Fig. 5b right). Here again, to the best of our knowledge, their cancer recurrent interaction is elusive in the current literature. Perhaps the main reason is due to the disrupted seed match region at the 7th position of miR-107, where an A is facing against a G in the 3′UTR of the TFGB1 transcript (Fig. 5b right). Consequently, most well-known databases such as TargetScan^9^, which are primarily based on the seed matches, do not report this interaction pair. Notably, however, the downstream 3 base-pairings at the non-seed region plus the anchor A at the 3′UTR facing the first miRNA nucleotide may compensate for the mismatch in the seed region and may in fact give rise to an effective interaction. Thus, our pan-cancer analyses not only identified canonical interactions between non-conserved miRNA and target genes but also non-canonical ones, which are nonetheless highly implicated by their confidence recurrence posteriors as well as the strong negative correlations across the 12 cancer types.

### Possibility of miRNA-mediated intercellular communication in metastasis

The above analyses identified a plausible role of recurrent interactions in regulating TGF-*β* signalling, eluding the possibility for miRNA-mediated intercellular communication. More intriguingly, however, the top hits from the functional enrichment analysis are mostly related to extracellular matrix (ECM) organization, cell focal adhesion, and ECM-receptor interactions (Table 2). Notably, the enrichment results was based on a standard hypergeometric test performed by DAVID online database^29^ using a general gene pool as background and the signature genes involved in the 1480 putative recurrent interactions as gene list. To ascertain these enrichments, we performed a randomization test by sampling 1480 random interactions from the ~1.8 million interactions pool and counted the number of genes involved in the above-mentioned KEGG pathways or GO-BP terms. We repeated the samplings 1 million times to construct an empirical distribution to assess the significance of the observed gene counts in each gene set. As expected, we observed a significant enrichment attributed to the 1480 recurrent interaction for each of the four gene sets of interest relative to the background distributions (*p* < 0.0007 or 0.017; Supplementary Fig. S4). Thus, the empirical enrichment analysis confirmed the validity of the above parametric enrichment results obtained from DAVID.

**Figure 6:**
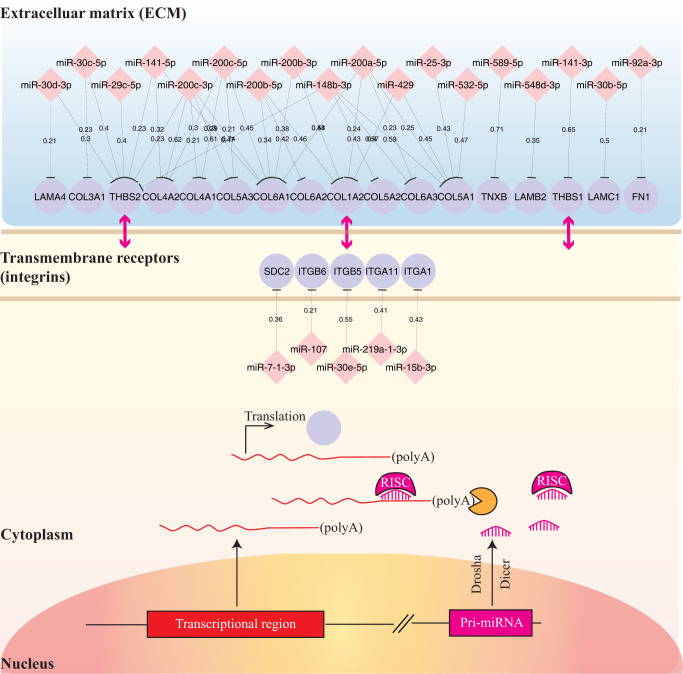
Pan-cancer miRNA-target network involving ECM-receptor interactions. Based on the KEGG ECM-receptor interactions pathway (hsa04512; Supplementary Fig. S5), we depicted a plausible mechanism, where miRNAs regulate components of both extracellular matrix (top panel) and transmembrane receptors (bottom panel) to mediate cell-to-cell communications. The miRNAs and genes drawn via Cytoscape^24^ are displayed in red diamonds and blue ellipses, respectively, and the edge labels are the posterior probabilities.

These results point to the intriguing possibility that miRNAs may not only regulate the ligand-receptor interaction but may also modulate the extracellular environment to facilitate cell-to-cell communication during metastasis. For instance, Fig. 6 depicts the recurrent pairs related to ECM-receptor interaction pathway from KEGG (hsa04512; also see original KEGG screenshot with targets highlighted in Supplementary Fig. S5). The targets are divided into two categories, namely the transmembrane receptors and the ECM components. Each target group are regulated by a set of specific miRNAs as implicated by the recurrence posterior probabilities (edge labels).

The ECM components include (among others) collagens, fibronectin, laminin, thrombospondin, which are the targets involved in the confidence recurrent interactions (Fig. 6 top panel). These extracellular macromolecules are produced intracellularly by resident cells and secreted into the ECM via exocytosis^36^. Specific interactions between cells and the ECM are mediated by the transmembrane receptors including integrins and syndecan^29^, whose components are targeted by five separate miRNAs as shown in the bottom panel of Fig. 6. The diverse ECM structure and composition enable a complex dynamics of tumor invasion and metastasis in cancer biology^36^. For instance, an early study suggested that metastasis is often accompanied with local dissolution of the ECM components including degradation of collagens to perhaps facilitate the invading tumour cells to traverse through the extracellular matrix^37^. Moreover, many cells bind to components of the ECM by focal adhesions, which is indeed another enriched GO biological process we identified from the pan-cancer interactions (Table 2).

### Survival analysis

To examine the clinical relevance of the identified recurrent interactions, we performed an extensive survival analyses by considering each gene or miRNA involved in the 1480 confidence recurrent interactions as a potential prognostic signature^38^. For each of the 12 cancer types, we obtained the corresponding clinical information from TCGA Data Portal. In particular, we used as a prognostic indicator the number of days elapsed before death or whether the patients were still alive since the initial diagnosis. We excluded PRAD (Prostate adenocarcinoma) due to the paucity of the survival data because most patients were still alive at the time of the measurements. For each interaction, we divided the samples into two groups depending on whether the corresponding gene or miRNA expression level is lower or higher than the sample means. We then employed Kaplan-Meier (KM) method using R package *survival*^39^ to compare the survival rates of the two groups, where the significance was determined by log-rank test. For each putative recurrent interaction, we required both the corresponding gene and miRNA to confer prognostic power at the significance level of *p* < 0.1.

As a result, 414 out of the 1480 interactions passed the threshold in at least one cancer type. Among these prognostic-recurrent interactions, 64 interactions are prominent in more than one cancer type (Supplementary Table S3). As a proof-of-principle, we repeated the same survival analysis on the genes and miRNAs involved in the ~1 million remaining interactions (i.e., excluding genes or miRNAs belonging to the 1480 recurrent interactions). Notably, all of these interactions exhibit negative correlation in at least 75% of the cancer types (Methods). Remarkably, we observed only 20% (or 225141) of the non-recurrent pairs that satisfied the selection criteria in contrast to 27% in the case of recurrent interactions. Thus, the recurrent interactions were significantly associated with patient survival outcomes (Fisher's exact test *p* < 9.52 × 10^−12^).

**Figure 7:**
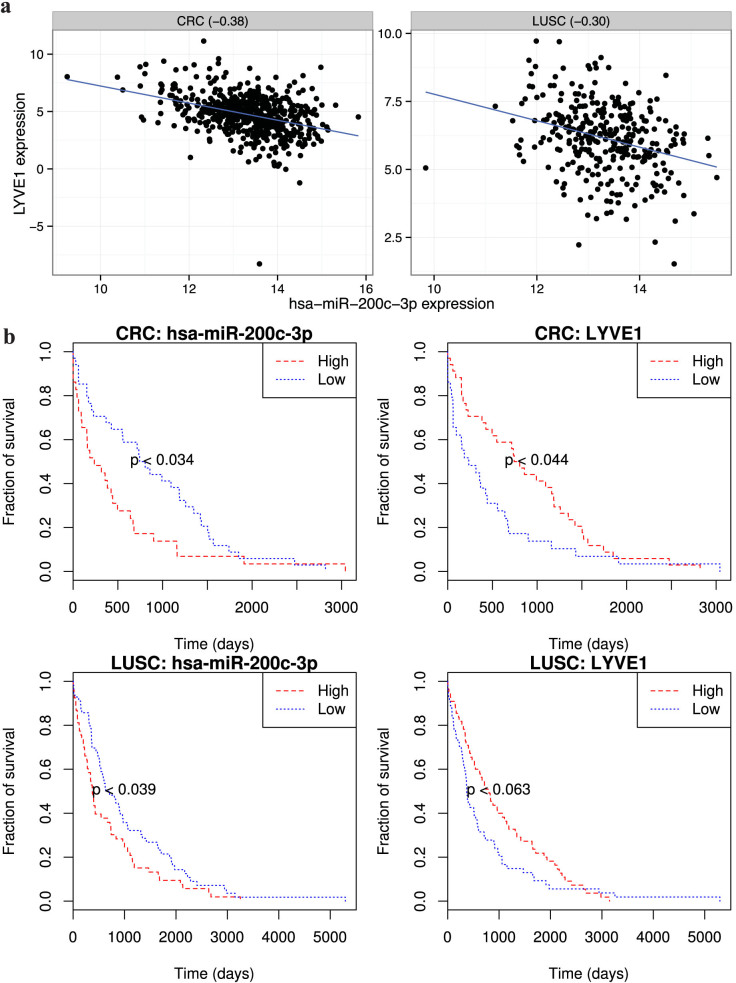
An example of the prognostic and recurrent miRNA-target interaction. (a). Scatterplots of miR-200c-3p and LYVE1 expression across samples in CRC and LUSC illustrate a recurrent negative expression correlation pattern (PanMiRa Bayesian joint posterior > 0.51 across all cancers) and cancer-specific Pearson correlation equal to −0.38 and −0.30, respectively. (b). Kaplan-Meier survival plots. Samples with the corresponding miR-200c-3p or LYVE1 expression higher (red dash) and lower (blue dot) than the sample means were divided into two groups, which were subject to KM survival estimates^39^. The fit was plotted as KM survival plot for CRC (upper panels) and LUSC (lower panels). P-values indicated above were computed by log-rank test.

We then generated the survival curves for the 414 prognostic-recurrent interactions in the corresponding cancer types (Supplementary Fig. S7). For instance, both the expression of oncomir hsa-miR-200c-3p^21^ and LYVE1 (lymphatic vessel endothelial hyaluronan receptor 1) involved in a recurrent interaction (PanMiRa recurrence posterior > 0.51) conferred significant log-rank statistics in CRC (colon rectum carcinoma) and LUSC (lung squamous-cell carcinoma) (Fig. 7). Notably, miR-200c-3p and LYVE1 assume prominent negative expression correlation in CRC (Pearson correlation: −0.38) and LUSC (−0.30) (Fig. 7a). More remarkably, the patient survival curves exhibit a coherent trend, namely patients with higher miR-200c-3p expression (lower LYVE1 expression) suffered poor prognostic outcomes, and *vice versa* (Fig. 7b). Interestingly, the expression of LYVE1 alone can significantly explained the survival rates in 4 other cancer types, namely BLCA, LGG, THCA, and UCEC (Supplementary Table S3). Importantly, LYVE1 (although not an oncogene per se; hence not shown in Fig. 4) is involved in cell-matrix adhesion and cellular component movement^40^. In particular, the gene *LYVE1* encodes type I integral membrane glycoprotein, which acts as a major receptor for the extracellular matrix (ECM) glycosaminoglycan hyaluronan (HA) and binds to both soluble and immobilized HA^40^. HA is an abundant component of skin and mesenchymal tissues where it facilitates cell migration^40^. Recent studies have shown that LYVE1 may function in lymphatic HA transport and have a role in tumor metastasis^41^,^42^. In particular, the interaction between LYVE1 and HA on the cell surface may play a role in the diversity of adhesion to cancer cells^41^. Moreover, LYVE1 dysregulation and lymphatic invasion are associated with poor prognostic outcomes and lymph node metastasis in neuroblastoma^42^.

Additionally, we also found in various cancer types several other prognostic pairs potentially associated with intercellular communications such as the recurrent interactions between miR-532-5p and COL5A1 (prognostically promising in KIRC and LGG) and between miR-1307-3p and ST6GALNAC6 (Supplementary Table S3; Supplementary Fig. S7). Notably, ST6GALNAC6 belongs to a family of sialyltransferases that modify proteins and ceramides on the cell surface to alter cell-cell or cell-extracellular matrix interactions^43^. Remarkably, both miR-1307-3p and ST6GALNAC6 conferred significant prognostic power in three different cancer types namely KIRC (Kidney renal clear-cell carcinoma), LUAD (Lung adenocarcinoma), and LUSC (Lung squamous-cell carcinoma). Together, our survival analysis revealed that some of the recurrent interactions associated with ECM organization are also prognostically promising, thus providing further support to the proposed miRNA-mediated intercellular communication mechanism in metastasis.

## Discussion

MicroRNAs (miRNAs) have been proposed to contribute to oncogenesis because they can function as tumour suppressors (e.g., miR-200 family), oncogenes (e.g., miR-155), or both (e.g., miR-29 family)^1^,^21^. Aberrant expression level of miRNAs are implicated in various cancers, and miRNA expression profiles have been shown to provide better discriminative power in classifying human cancers than mRNA profiles^4^. Furthermore, in one of our own recent studies, we demonstrated the diagnostic power of using a novel class of probabilistic miRNA-mRNA interaction signatures derived from the paired miRNA and mRNA expression from individual samples and sequence-based information to discriminate tumor from normal samples in thyroid and breast cancer patients^8^. However, one intriguing question remained for us was whether there is a set of recurrent miRNA-target interactions that are present in most if not all of the cancers. To examine this hypothesis, we constructed from TCGA Data Portal^13^ a large-scale pan-cancer data compendium of 12 cancer types each consisting of expression profiles of long RNAs and miRNAs measured by RNA-seq as well as DNA methylation (DM) and copy number (CN) measured by microarrays over hundreds of samples. In total, our pan-cancer data compendium contains molecular measurements of 17,788 genes and 677 miRNAs in each of the 4258 samples, which to our knowledge represents one of the largest pan-cancer studies in the recent literature.

Notably, to identify recurrent interactions, a simple approach would have been using negative correlation in all cancers as a direct filter. However, population heterogeneity, non-uniform tumor purity, various laboratory settings, and different sample sizes between different cancer types will confer different statistical confidence even for the same correlation scores or linear coefficients calculated in different cancer types. For instance, a negative correlation of −0.1 may be only modestly significant in one cancer type but highly significant in another cancer type due to different sample sizes, which may to some extent even mislead one to think that the identified interactions were cancer-specific when it in fact ubiquitously occurs among many other cancers. Thus, it is difficult to choose a universal cutoff based directly on the correlation scores without arbitrary decision. Jacobsen *et al.* (2013) has recently proposed a rank-based method called REC to evaluate the recurrence of miRNA-target association, which avoids direct comparison between cancer types and thus mitigates the above-mentioned issues^6^. In this study, we introduced a probabilistic approach called PanMiRa to directly infer the posterior distribution of the pan-cancer miRNA-target associations, thus obviating the need of resorting to *χ*^2^ approximation as was the case in the rank-based REC scoring regime. Some of the ideas from PanMiRa were based on a recent method called MCMG (Multiple Cancers for MicroRNA-Gene interactions)^7^. However, the objectives are fundamentally different: MCMG was developed for predicting cancer-specific interactions by borrowing information from other cancers whereas PanMiRa aims at inferring recurrent interactions across all cancer types. Moreover, MCMG operates on a Fisher-transformed Pearson correlation matrix, which does not take into account the CN and DM effects as in REC and PanMiRa.

To establish the confidence of the method, we performed a comprehensive evaluation on the ~20 thousand positive recurrent interactions identified by PanMiRa as having positive recurrence posterior probabilities among the ~1.8 million candidate interactions with negative *z*-scores in at least 75% of the cancer types. Encouragingly, we found that these interactions exhibit significantly higher sequence-based scores than the remaining interactions and more enriched for validated and CLASH-detected interactions than the interaction pairs derived from randomly shuffled posteriors and from REC. Moreover, targets involved in the recurrent interactions exhibit significantly stronger expression fold-changes than the remaining genes in four out of the six publicly available miRNA perturbation datasets. Importantly, miRNAs and targets involved in the recurrent interactions are significantly enriched for known oncomirs and oncogenes, respectively. In particular, we found that miR-200 family is especially prevalent in the oncomir-oncogene recurrent network, perhaps highlighting their under-appreciated importance in cancer pathogenesis.

Nonetheless, compared to the total number of validated and CLASH interactions the observed number among the top ranked recurrent interactions are relatively small (Fig. 2). One major reason could be the indirect interactions between miRNAs and targets, which are currently difficult to distinguish from the genuine interactions because of the confounding effects such as the cascading transcriptional events. Most previous methods used sequence-based information to filter false positive interactions^44^,^45^,^46^. However, our knowledge about the general principles of miRNA target recognition is still limited. In this regard, CLASH is an unbiased experimental approach to tackle such problem^11^. For future reference, we dedicated an additional column in Supplementary Table S1 to indicate whether each recurrent interaction is also implicated in CLASH. However, the retention rates of CLASH (i.e., sensitivity of the protocol to detect all Argonaute-pulled down miRNA:RNA interactions) is not very high, and many *bona fide* interactions might be unobserved from the data^12^. Additionally, CLASH was performed on a generic cell-line namely HEK293^11^, which may exhibit a different miRNA target repertoire from the actual miRNA-target landscape in the clinical samples. *However, we emphasize that the identified recurrent interactions from our simple Bayesian model may include both direct and indirect miRNA and gene associations*. As a future work, we will develop a sophisticated statistical model to first learn the intrinsic miRNA recognition elements from the CLASH data and then incorporate that information as Bayesian prior to infer the posterior distributions of the functional and direct miRNA-target interactions based on expression data.

Intriguingly and perhaps the highlight of the present study is the discovery of the significant association of the 1480 high confidence recurrent interactions with not only the cancer-related pathways such as TGF-*β* signalling pathways but also (even more significantly so) the pathways related to *intercellular communication* such as extracellular matrix (ECM) structural organization, cell focal adhesion, and ECM-receptor interactions. The results point to the possibility of miRNA-mediated re-organization of extracellular environment to facilitate cell-to-cell communication, which may be advantageous to tumor cell invasion to adjacent cells during metastasis. Moreover, another possibility in line with the miRNA-related cancer mechanism is that the miRNA-altered extracellular environment may in turn facilitate transportations of the *extracellular/exosomal RNA* (exRNA) via extracellular vesicles or exosomes^16^, which contain (among other RNA materials) miRNAs themselves, from tumor cell to adjacent cell^16^,^47^. Indeed, tumor-derived exosomes have been identified in the plasma of various cancer patients^16^. Only recently, however, miRNAs were found to be present in these vesicles^48^,^49^. Importantly, the exosomal miRNAs can be functionally delivered to target cells and post-transcriptionally regulate the cognate target genes in the host^47^. For instance, Montecalvo *et al.* (2012) demonstrated that endogenously released exosomes constitute an effective means of communication between mouse dendritic cells (DCs), and that such vesicles are capable of delivering functional exosome-shuttle miRNAs into the cytosol of the target DCs, which in turn caused a significant and dose-dependent decrease in normalized expression of luciferase reporters in the recipient cells^47^. If the theory follows, then the cells in vicinity may be rapidly or insidiously assimilated with the tumorigenic information in the form of “miRNA messengers” both quantitively and qualitatively and eventually turn into cancer cells.

**Figure 8:**
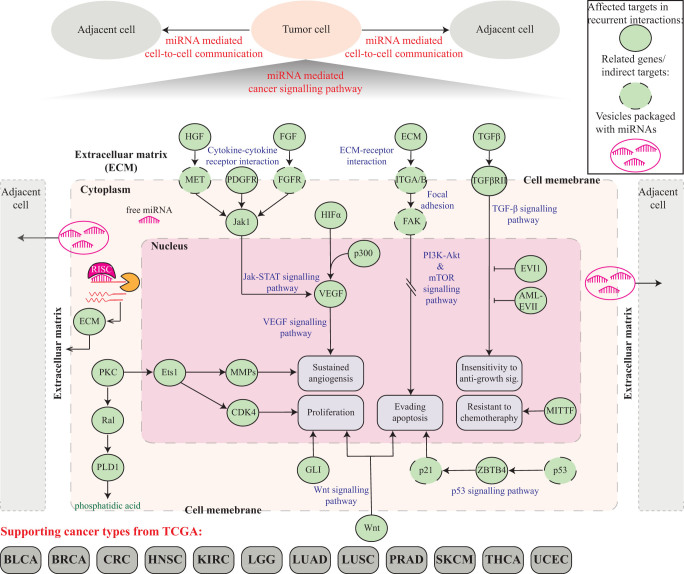
The proposed model of the miRNA-mediated intercellular communication mechanism. The top panel depicted the cancer cell invasion to other cells in vinicty, possibly mediated by miRNA regulation of extracellular environment such as extracellular matrix (ECM) organization as well as receptor interactions such as focal adhesion. The bottom panel zooms into the tumor or tumor-transforming cell, which was drawn based on the KEGG pathways in cancers (hsa05200)^30^ and the confidence recurrent miRNA regulatory network. Several important cancer signalling pathways possibly affected by miRNA dysregulation include Jak-STAT, VEGF, PI3K-Akt, mTOR, Wnt, p53, and TGF-*β* signalling pathways, which may drive phenotypic changes such as sustained angiogenesis, cell proliferation, evading apoptosis, insensitivity to anti-growth signals, and resistant to chemotherapy. On the other hand, intercellular interactions including cytokine-cytokine receptor interactions, ECM-receptor interactions, and focal adhesion are also likely to be mediated by miRNAs to facilitate tumor invasion. Also drawn is the transportation of the *extracellular RNA* (exRNA) via extracellular vesicles or exosomes^16^. It is possible that miRNAs not only mediate cell-to-cell communication by altering the ECM composition and receptor interactions but also serve as an effective communication means as tumorigenic messengers in the form of exRNA to infiltrate into adjacent cells^47^. The exRNA transportation via exosomes may be in turn facilitated by the miRNA-related re-organization of the extracellular environment.

Finally, we postulate a plausible biological mechanism in attempt to elucidate a novel aspect of the cancer biology. The top panel depicted in Fig. 8 illustrates the cancer cell invasion to adjacent cells, which is possibly mediated by miRNA regulation of the expression of the ECM constituents and ultimately the ECM organization as well as receptor interactions such as focal adhesion. The bottom panel illustrates a sketch of what might occur inside the tumor or tumor-transforming cell, which is drawn based on the KEGG pathways in cancers (hsa05200)^30^ and the cognate targets (green solid circle) identified from the confidence recurrent interactions (see highlighted boxes in Supplementary Fig. S6). Several important cancer signalling pathways may be affected by miRNA dysregulation including Jak-STAT, VEGF, PI3K-Akt, mTOR, Wnt, p53, and TGF-*β* signalling pathways, which may drive phenotypic changes such as sustained angiogenesis, cell proliferation, evading apoptosis, insensitivity to anti-growth signals, and resistant to chemotherapy. On the other hand, intercellular interactions including cytokine-cytokine receptor interactions, ECM-receptor interactions, and focal adhesion are also likely to be mediated by miRNAs to facilitate tumor invasion. As discussed above, it is also possible that miRNAs not only mediate cell-to-cell communication by post-transcriptionally altering the expression of genes involved in ECM organization and receptor interactions but also contribute to a more profound impact on cell reprogramming by “infiltrating” into adjacent cells via the exRNA transport mechanism, which is in turn facilitated by the miRNA-related re-organization of the extracellular environment. Together, we speculate that miRNAs may have three regulatory roles in cancer biology: (1) regulating cancer signalling pathways within a cell; (2) regulating extracellular environment to facilitate intercellular communication; (3) participating exRNA transfer program to infiltrate into adjacent cells. It is easy to see that the three roles are related and form a reinforcing feedback loop that appears natural from an informatics perspective.

## Methods

### TCGA data processing

We chose 12 cancer types from the TCGA Data Portal based on the availability of the molecular measurements and the sample size (Table 1). For each cancer type, we downloaded the processed (Level 3) long and short RNA-seq data corresponding to the expression levels of target RNAs and miRNAs, respectively. For the RNA-seq data, we used RNASeqV2 (i.e., files with extension rsem.genes.normalized_results) mainly because more samples were recorded in this version and the gene expression quantification by normalized read counts is more accurate than simply summing the transcript-level RPKM (reads per kilobase per million mapped reads) due to the accurate estimation of isoform expression by the RSEM (RNA-Seq by Expectation Maximization) algorithm^50^. For the miRNA-seq data, we chose the mature isoform expression data (i.e., files with extension isoform.quantification.txt). We filtered out cross-mapped regions and then summed over the reads per million miRNAs mapped (RPM) values for each mature miRNA sequence. Both target RNAs and miRNAs with expression in less than 5% samples within the same cancer type were filtered out. The remaining expression values were log2-transformed after replacing all the zero values with the lowest non-zero values. DNA copy number data were obtained as the processed Level 4 data in the form of Gistic2 scores^17^ from Firehose (http://gdac.broadinstitute.org/runs/analyses__2012_12_21/). DNA methylation Level 3 data as the beta-values derived from the Illumina HumanMethylation (HM) 27 or 450 platform were obtained from TCGA Data Portal. Only probes mapped to the promoter regions of the target genes were used. For multiple probes mapped to the same promoter region, we chose the probe with strongest negative expression correlation with the mRNA expression based on the Level 4 data from Firehose (http://gdac.broadinstitute.org/runs/analyses__2012_12_21/). In the case where the same sample were measured with both HM27 and HM450 data, HM450 took precedence due to the higher coverage. The processed TCGA data panel is available at www.cs.utoronto.ca/~yueli/PanMiRa.html.

### PanMiRa model details

Let *y_i_*_,*t*,*d*_ and 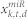 denote the expression of target gene *i* and miRNA *k* in sample *t* of cancer type *d*, respectively; and 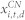, 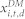 denote the corresponding copy number (CN) and DNA methylation (DM) for gene *i*, respectively. To estimate the repression effects of miRNA *k* on gene *i* (without loss of generality), we employed a multivariate linear regression model with ordinary least-squares estimation: 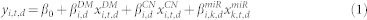
where *β*_0_ is the bias. 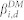 and 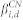 are the offsets accounting for the expression changes due to CN and DM, respectively. In R, this was performed by the built-in function lm(y ~ cnv + dm + miR). The coefficient of interest 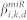 indicates the targeting relationship between miRNA *k* and gene *i* in cancer type *d* and was first transformed into *t*-statistic: 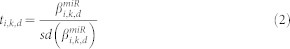
where 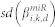 is the standard error of the coefficient as calculated by the standard ANOVA from the summary.lm function in R. The *t*-statistics were first transformed into probabilities based on *t*-distribution with *T* − 4 degrees of freedom, where *T* is the number of samples, and then to standard normal statistics or *z*-scores together by the R command qnorm(pt(t, T − 4)). The resulting *z*-scores are theoretically distributed as *N*(0, 1) under the null hypothesis. To reduce false positives, we first retained only the interactions with negative *z*-scores in at least 75% of the cancer types, resulting in 1,848,529 candidate recurrent interactions and subjected them to the local false discover rate (*locfdr*) estimation^7^,^18^: 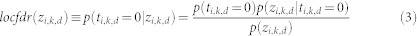
where *p*(*z_i_*_,*k*,*d*_) = *p*(*t_i_*_,*k*,*d*_ = 0)*p*(*z_i_*_,*k*,*d*_|*t_i_*_,*k*,*d*_ = 0) + *p*(*t_i_*_, *k*,*d*_ = 1)*p*(*z_i_*_,*k*,*d*_|*t_i_*_,*k*,*d*_ = 1). Similarly, 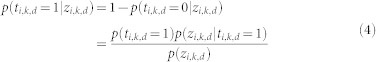
Rearranging (3) and (4), we can obtain the following likelihoods: 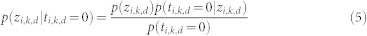

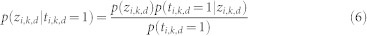
Suppose the latent variables *t_i_*_,*k*,*d*_'s are independent and *z_i_*_,*k*,*d*_'s are conditionally independent given *t_i_*_,*k*,*d*_. Then, the joint likelihood can be factorized into the products of individual likelihoods: 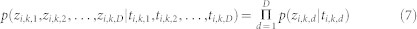
We now have everything except for the prior *p*(*t_i_*_,*k*,1_, *t_i_*_,*k*,2_, …, *t_i_*_,*k*,*D*_) to infer the joint posterior: 
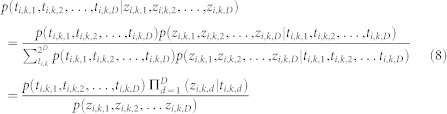
In order to perform the above inference, we employ an empirical Bayes approach. First, we initialize the prior to be uniformly distributed over the binary configuration: 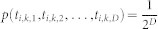, where *D* is the total number of cancer types. Then, we perform the inference using (8). Given the joint posteriors, we then re-estimate the prior by: 
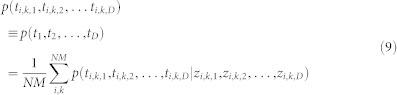
where *N* and *M* are the total number of target genes and miRNAs, respectively. We then iteratively alternate between (8) and (9) until the overall likelihood: 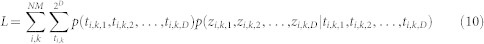
increases by less than *tol* (default: 10^−5^). Finally, the recurrence posterior is defined as the posterior *p*(*t_i_*_,*k*,1_, *t_i_*_,*k*,2_, …, *t_i_*_,*k*,*D*_|*z_i_*_,*k*,1_, *z_i_*_,*k*,2_, …, *z_i_*_, *k*,*D*_), where ∀*t_i_*_,*k*,*d*_ = 1. To account for numerical underflow, some of the above operations such as (7) were performed at the logarithmic scale, where the log of products becomes the sum of logs, and transformed back to probabilities in the end. Please see the R source code (www.cs.utoronto.ca/~yueli/PanMiRa.html) for implementation details.

### Implementation of recurrence score (REC)

As a comparison to PanMiRa, we implemented a recently published method called recurrence (REC)^6^. We first calculated the *t*-statistic *t_i_*_,*k*,*d*_ the same way as in (2) to estimate the interactions between gene *i* and miRNA *k* in cancer type *d*. Using *t_i_*_,*k*,*d*_'s, we ranked the targets *i* for each miRNA *k* in cancer type *d* to obtain the rank score *r_i_*_,*k*,*d*_'s, which were then normalized by *rr_i_*_,*k*,*d*_ = *r_i_*_,*k*,*d*_/*N* − 1/2*N*, where *N* is the total number of genes. As a result, *rr_i_*_,*k*,*d*_ is uniformly distributed under null hypothesis on the interval [0, 1]. The *rr_i_*_,*k*,*d*_'s were then transformed into 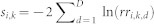, where *D* is the number of cancer types. *s_i_*_,*k*_ is assumed to approximately follow *χ*^2^ distribution with 2 × *D* degrees of freedom. The REC score *REC_i_*_,*k*_ is essentially the log 10 transformation of the one-sided *χ*^2^ probability: 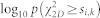 computed by the R function pchisq(*s_i_*_,*k*_, 2*D, lower.tail = TRUE, log.p = TRUE)/log(10). Thus, the more negative the *REC_i_*_,*k*_ the more likely the interaction between miRNA *k* and gene *i* is real.

### Sequence-based predictions

Sequence-based feature scores namely MiRanda-mirSVR (August 2010 release) were downloaded from http://www.microrna.org10. “Good mirSVR score” for conserved and non-conserved miRNA were used. TargetScan predictions were downloaded from TargetScan-Human 6.2 database (targetscan.org). We assigned each gene the mirSVR score or TargetScan PCT corresponding to its transcript with the longest 3′UTR based on the RefSeq annotation (Release 66).

### Physical miRNA-target interactions from CLASH

Processed CLASH data were obtained from Supplementary Table 1 and 2 of the original study^11^ corresponding to 18,514 miRNA:mRNA and 4,484 miRNA:ncRNA interactions observed in human HEK293 cell line.

### miRNA perturbation data

Microarray miRNA perturbation data were obtained from Gene Expression Omnibus (GEO) database with accessions GSE6838 for (anti-)miR-16/106b, GSE38581 for miR-29c, GSE37119 for miR-200b, and from Frankel *et al.* (2008) for anti-miR-21^51^. The expression fold-changes were log2 transformed if not yet done so by the original studies.

### Data and software available

The processed TCGA pan-cancer data compendium as in R Data, the PanMiRa R source code, and the results generated for this paper are all available at www.cs.utoronto.ca/~yueli/PanMiRa.html.

## Author Contributions

Y.L. designed the study, developed and implemented the algorithms, analyzed the data, and wrote the manuscript. Z.Z. supervised the study. All authors commented and approved the final manuscript.

## Supplementary Material

Supplementary Information Supplementary Information

Supplementary Information Supplementary Table S1

Supplementary Information Supplementary Table S2

Supplementary Information Supplementary Table S3

## Figures and Tables

**Table 1:** Summary of the analyzed TCGA data sets of 12 major cancer types

Disease	Description	Samples	target RNAs	miRNAs
BLCA	Bladder urothelial carcinoma	134	18,622	875
BRCA	Breast invasive carcinoma	847	18,564	806
CRC*	Colon rectum carcinoma	395 (155)	18,395 (18,390)	794 (804)
HNSC	Head and neck squamous-cell carcinoma	303	18,689	864
KIRC	Kidney renal clear-cell carcinoma	474	18,648	737
LGG	Brain Lower Grade Glioma	179	18,525	993
LUAD	Lung adenocarcinoma	350	18,539	847
LUSC	Lung squamous-cell carcinoma	315	18,670	857
PRAD	Prostate adenocarcinoma	170	18,514	765
SKCM	Skin Cutaneous Melanoma	234	18,552	892
THCA	Thyroid carcinoma	224	18,299	830
UCEC	Uterine corpus endometrioid carcinoma	478	19,003	877
Pan-cancer panel of the above 12 cancer types		4,258	17,788	677

For each target RNA or miRNA, we obtained the corresponding expression levels, copy number, and DNA methylation at the promoter region within the same sample. Genes or miRNA expressed in less than 10% of the samples in the same cancer type were filtered out. The pan-cancer panel combines the 12 cancer types based on commonly measured genes and miRNA.

*The CRC data is the union of colon adenocarcinoma (COAD) and rectum adenocarcinoma (READ) data from TCGA.

**Table 2:** Functional enrichment of genes involved in the recurrent interactions

Database	Term	Hits	Total Hits	p-value	q-value
UniProt	Extracellular matrix (ECM)	62	241	6.7E-26	3.7E-23
GO-BP	GO:0030198 extracellular matrix organization	33	104	7.9E-16	2.2E-12
GO-BP	GO:0007155 cell adhesion	91	700	8.3E-14	1.2E-10
GO-BP	GO:0022610 biological adhesion	91	701	8.9E-14	8.5E-11
GO-BP	GO:0043062 extracellular structure organization	35	163	2.2E-11	1.3E-08
KEGG	hsa04510:Focal adhesion	38	201	7.6E-11	1.2E-08
GO-MF	GO:0019838 growth factor binding	25	105	4.2E-09	1.7E-06
KEGG	hsa04512:ECM-receptor interaction	22	84	4.3E-09	3.3E-07
GO-BP	GO:0051271 negative regulation of cell motion	15	63	8.3E-06	1.7E-03
GO-CC	GO:0044459 plasma membrane part	171	2203	1.1E-05	2.9E-04
GO-BP	GO:0030155 regulation of cell adhesion	22	137	2.6E-05	3.8E-03
GO-BP	GO:0048771 tissue remodeling	13	56	5.3E-05	6.3E-03
GO-BP	GO:0030334 regulation of cell migration	24	169	7.6E-05	8.7E-03
GO-BP	GO:0001936 regulation of endothelial cell prolif.	9	32	3.1E-04	3.0E-02
GO-BP	GO:0002039 p53 binding	6	17	2.1E-03	1.1E-01
GO-MF	GO:0046332 SMAD binding	9	46	4.3E-03	1.8E-01
KEGG	hsa05200:Pathways in cancer	31	328	5.7E-03	1.0E-01
KEGG	hsa04350:TGF-beta signaling pathway	12	87	9.7E-03	1.3E-01

Targets from recurrent miRNA-target interactions were subjected to functional enrichment tests using DAVID^29^. The top functional terms were listed above along with its database sources, (total) number of target genes, p-value, and q-value (Benjamini-Hochberg adjusted).
