# Supplementary material for: Potential microRNA-mediated oncogenic intercellular communication revealed by pan-cancer analysis
Source: Sci Rep. 2014 Nov 18;4:7097. doi: 10.1038/srep07097 (PMC4235308; doi:10.1038/srep07097)
Supplement: Supplementary Information [file srep07097-s1.pdf]

Potential microRNA-mediated oncogenic intercellular communication revealed by pan-cancer analysis

Yue Li[1,2,*] and Zhaolei Zhang[1,2,3,4,*]

[1]Department of Computer Science, University of Toronto, Toronto, Ontario M5S 3G4, Canada
[2]The Donnelly Centre, University of Toronto, Toronto, Ontario M5S 3E1, Canada
[3]Department of Molecular Genetics, University of Toronto, Toronto, Ontario M5S 1A8, Canada
[4]Banting and Best Department of Medical Research, University of Toronto, Toronto, Ontario M5S 3E1, Canada
[*]Correspondence to yueli@cs.toronto.edu or zhaolei.zhang@utoronto.ca

Supplementary Figures

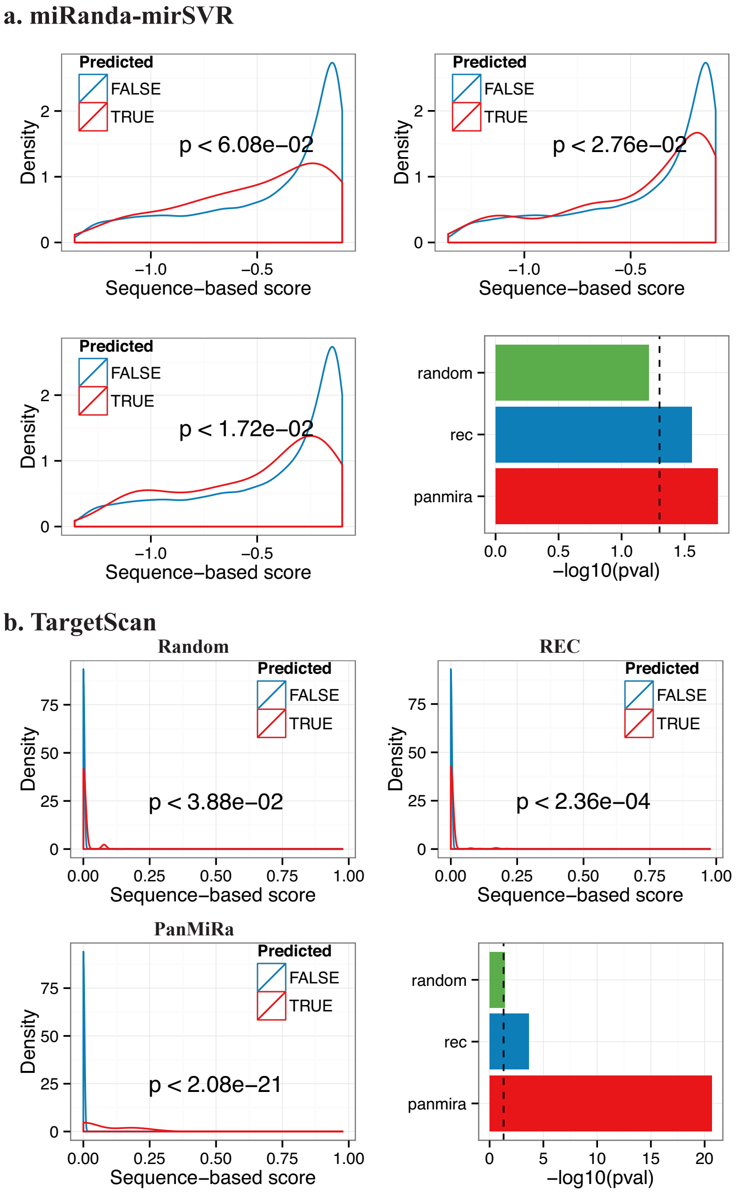

Figure S1: **Sequence-based score comparison.** The distributions of the sequence-based scores from (**a**) miRanda-mirSVR and (**b**) TargetScan probability of conserved targeting (PCT) were compared between the positive recurrent interactions predicted by PanMiRa and the remaining pairs. The same test were performed for the interactions detected by randomly shuffled posteriors and REC with -1 as the cutoff. For miRanda-mirSVR (TargetScan PCT), the more negative (positive) the scores the stronger implication of a true interaction based on the sequence-based features. P-values indicated above were computed by one-sided Wilcoxon rank-sum test. The resulting significance levels in terms of -log10(p-value) are compared among the three methods. The dash line indicates the standard cutoff of p-value = 0.05.

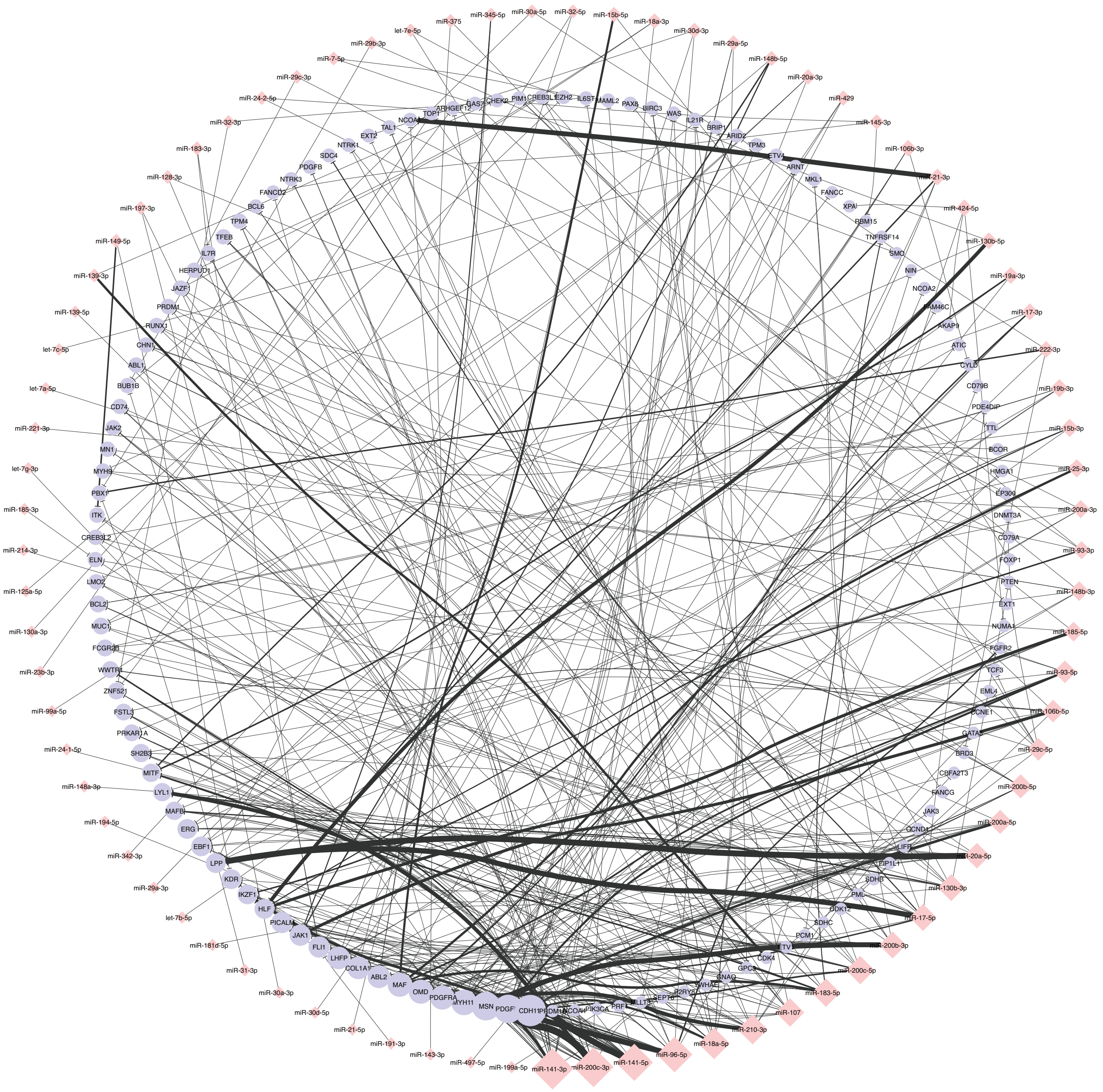

Figure S2: **Full oncomir-oncogene cancer network.** We filtered the the positive recurrent interactions by the miRNAs and target genes that are in known oncomirs [1, 2] and oncogenes [3], respectively. Then, we generated the oncomir-oncogene pan-cancer network and analyzed it under Cytoscape [4]. Node size and the edge width are proportional to the in/out-degree and recurrence posterior in the overall oncogene-oncomir network, respectively.

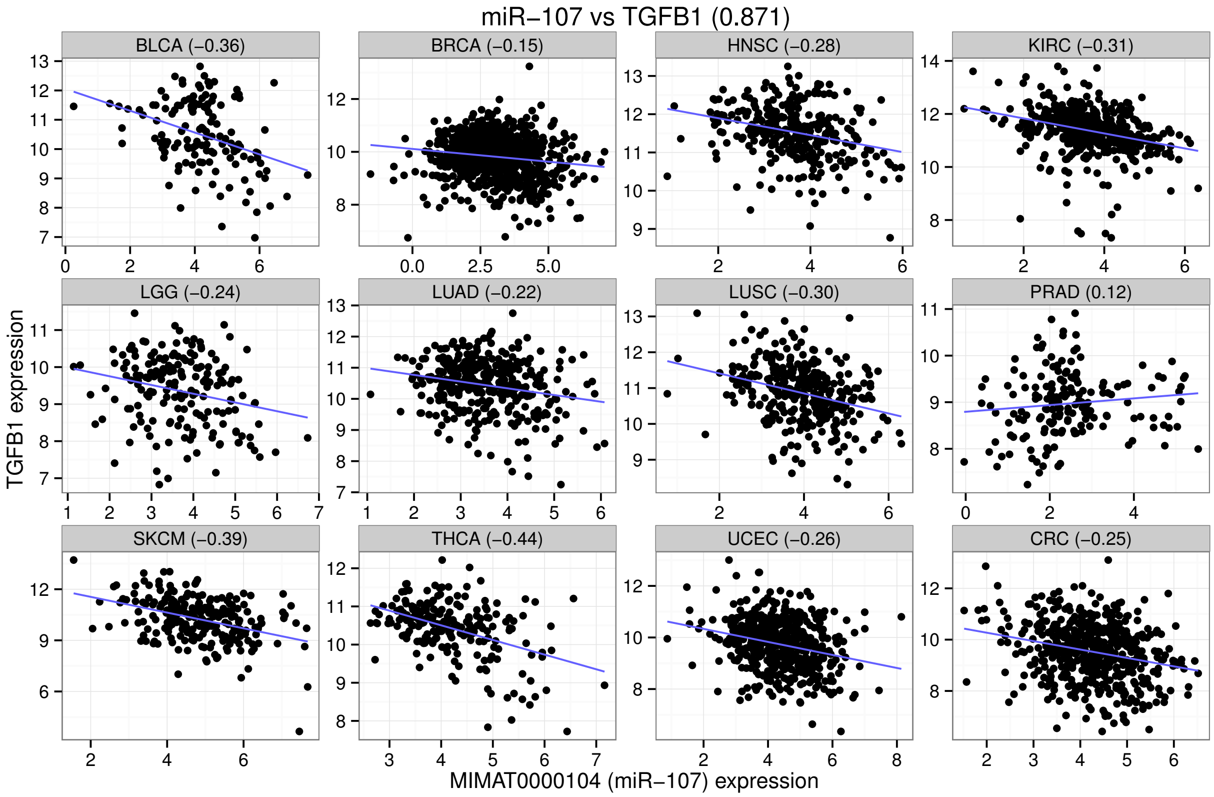
miR−107 vs TGFB1 (0.871)
BLCA (−0.36)
BRCA (−0.15)
HNSC (−0.28)
KIRC (−0.31)
LGG (−0.24)
LUAD (−0.22)
LUSC (−0.30)
PRAD (0.12)
SKCM (−0.39)
THCA (−0.44)
UCEC (−0.26)
CRC (−0.25)
TGFB1 expression
MIMAT0000104 (miR−107) expression

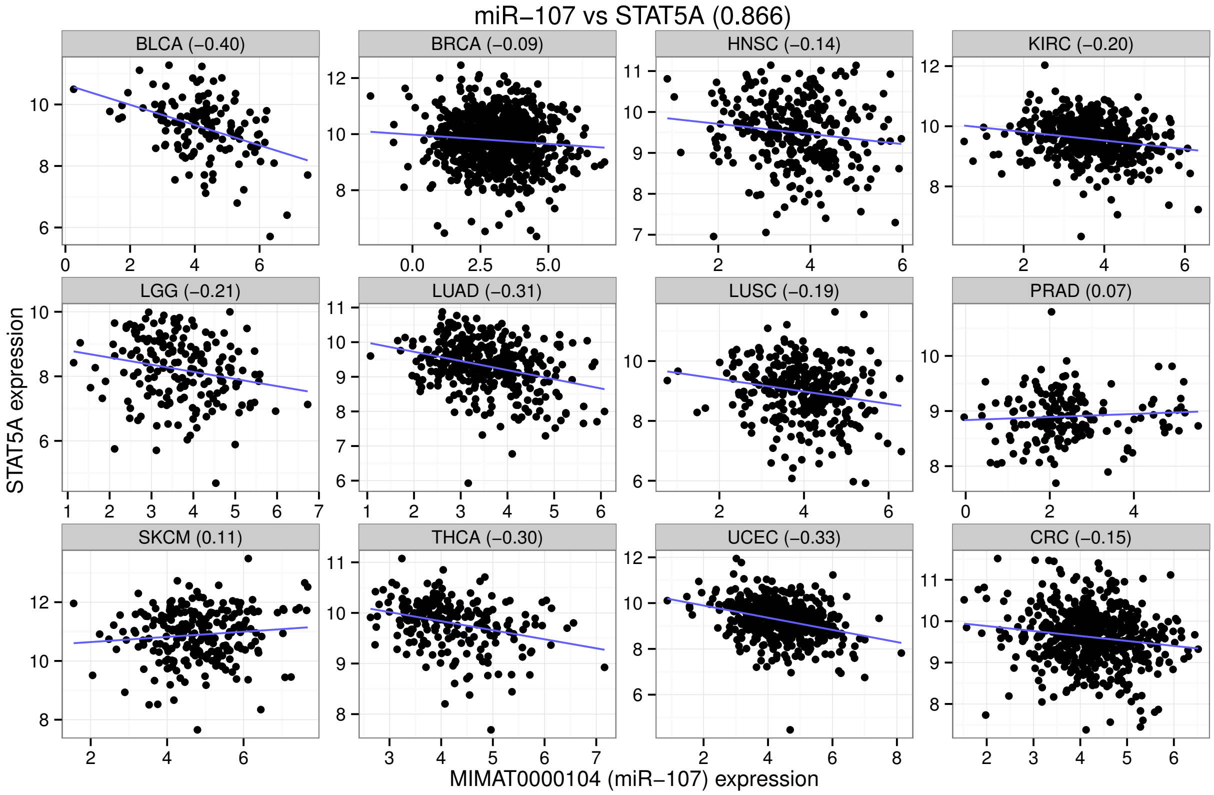
miR−107 vs STAT5A (0.866)
BLCA (−0.40)
BRCA (−0.09)
HNSC (−0.14)
KIRC (−0.20)
LGG (−0.21)
LUAD (−0.31)
LUSC (−0.19)
PRAD (0.07)
SKCM (0.11)
THCA (−0.30)
UCEC (−0.33)
CRC (−0.15)
STAT5A expression
MIMAT0000104 (miR−107) expression

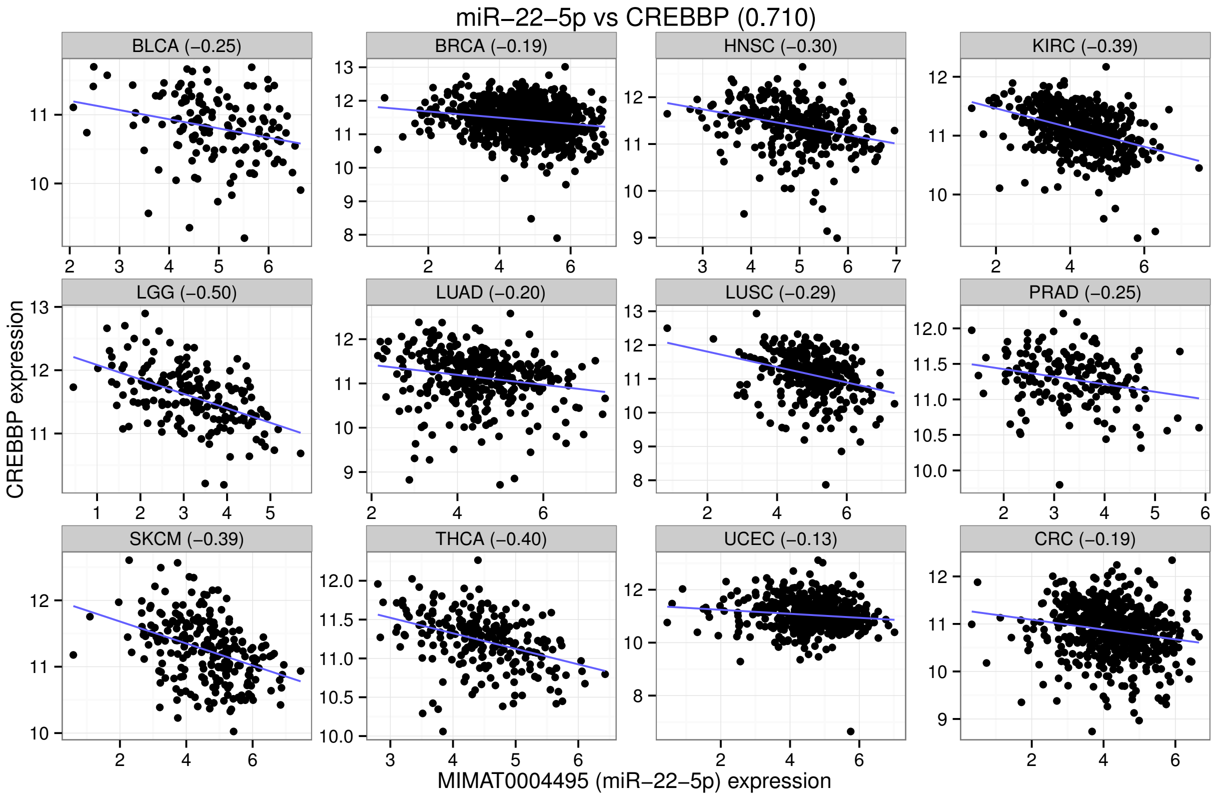
miR−22−5p vs CREBBP (0.710)
BLCA (−0.25)
BRCA (−0.19)
HNSC (−0.30)
KIRC (−0.39)
LGG (−0.50)
LUAD (−0.20)
LUSC (−0.29)
PRAD (−0.25)
SKCM (−0.39)
THCA (−0.40)
UCEC (−0.13)
CRC (−0.19)
CREBBP expression
MIMAT0004495 (miR−22−5p) expression

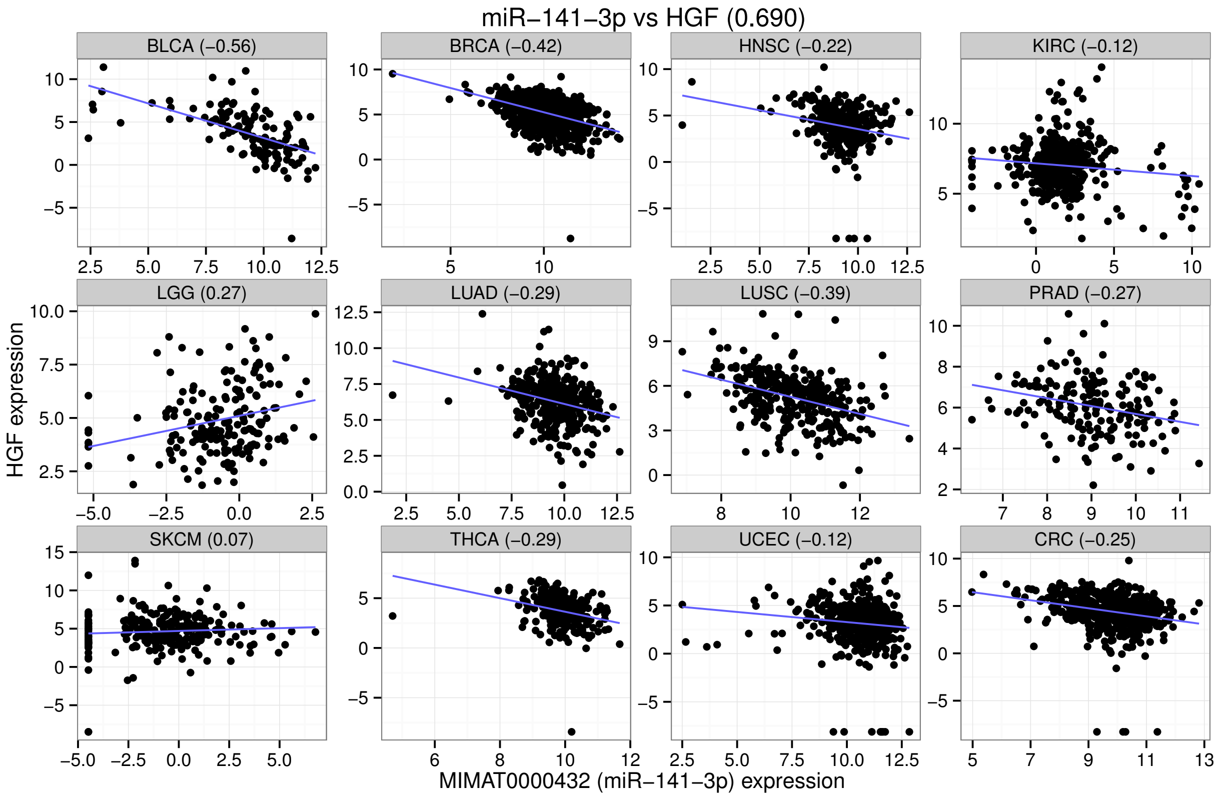
miR−141−3p vs HGF (0.690)
BLCA (−0.56)
BRCA (−0.42)
HNSC (−0.22)
KIRC (−0.12)
LGG (0.27)
LUAD (−0.29)
LUSC (−0.39)
PRAD (−0.27)
SKCM (0.07)
THCA (−0.29)
UCEC (−0.12)
CRC (−0.25)
HGF expression
MIMAT0000432 (miR−141−3p) expression

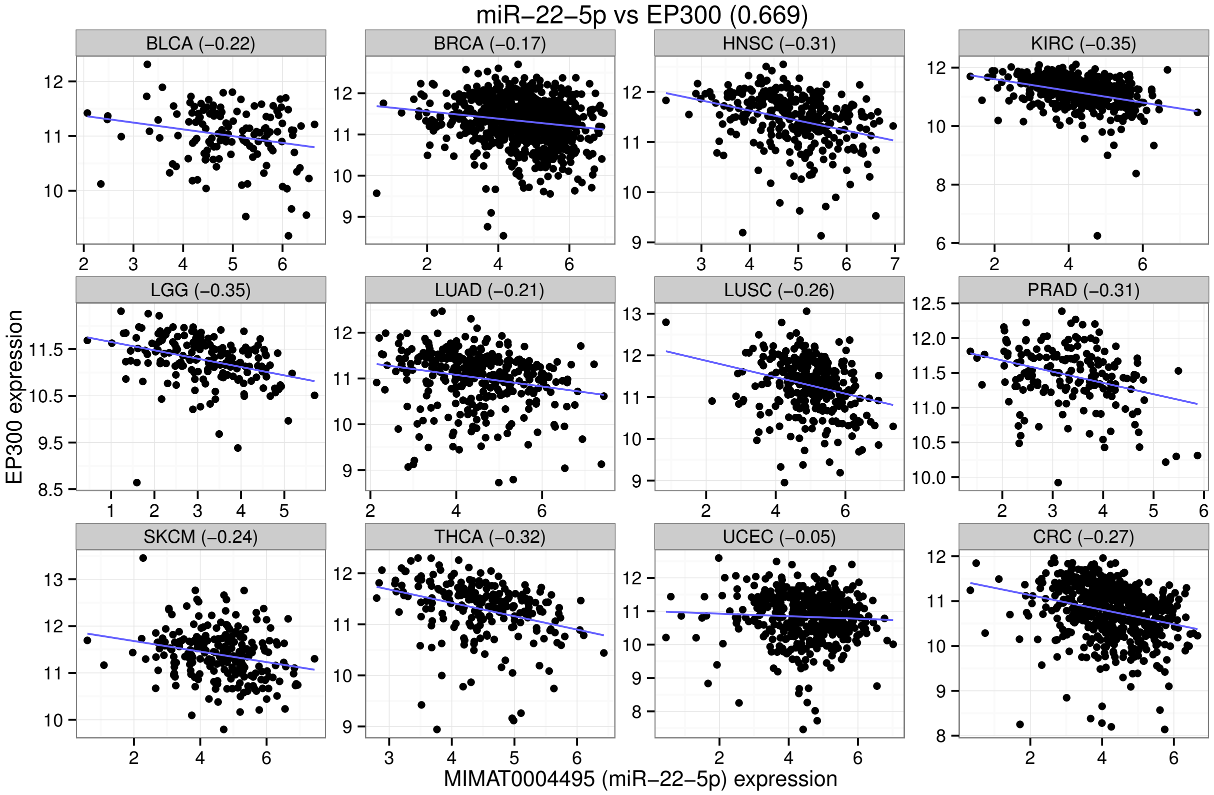
miR−22−5p vs EP300 (0.669)
BLCA (−0.22)
BRCA (−0.17)
HNSC (−0.31)
KIRC (−0.35)
LGG (−0.35)
LUAD (−0.21)
LUSC (−0.26)
PRAD (−0.31)
SKCM (−0.24)
THCA (−0.32)
UCEC (−0.05)
CRC (−0.27)
EP300 expression
MIMAT0004495 (miR−22−5p) expression

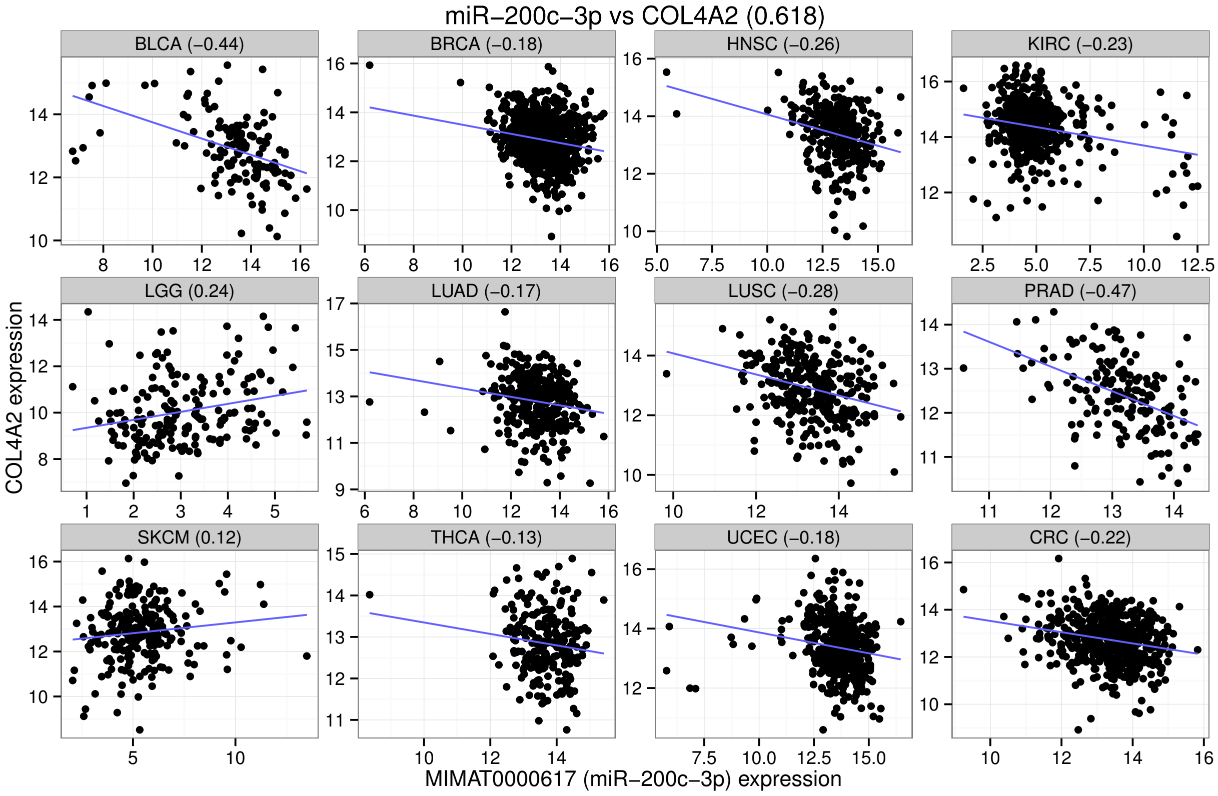
miR−200c−3p vs COL4A2 (0.618)
BLCA (−0.44)
BRCA (−0.18)
HNSC (−0.26)
KIRC (−0.23)
LGG (0.24)
LUAD (−0.17)
LUSC (−0.28)
PRAD (−0.47)
SKCM (0.12)
THCA (−0.13)
UCEC (−0.18)
CRC (−0.22)
COL4A2 expression
MIMAT0000617 (miR−200c−3p) expression

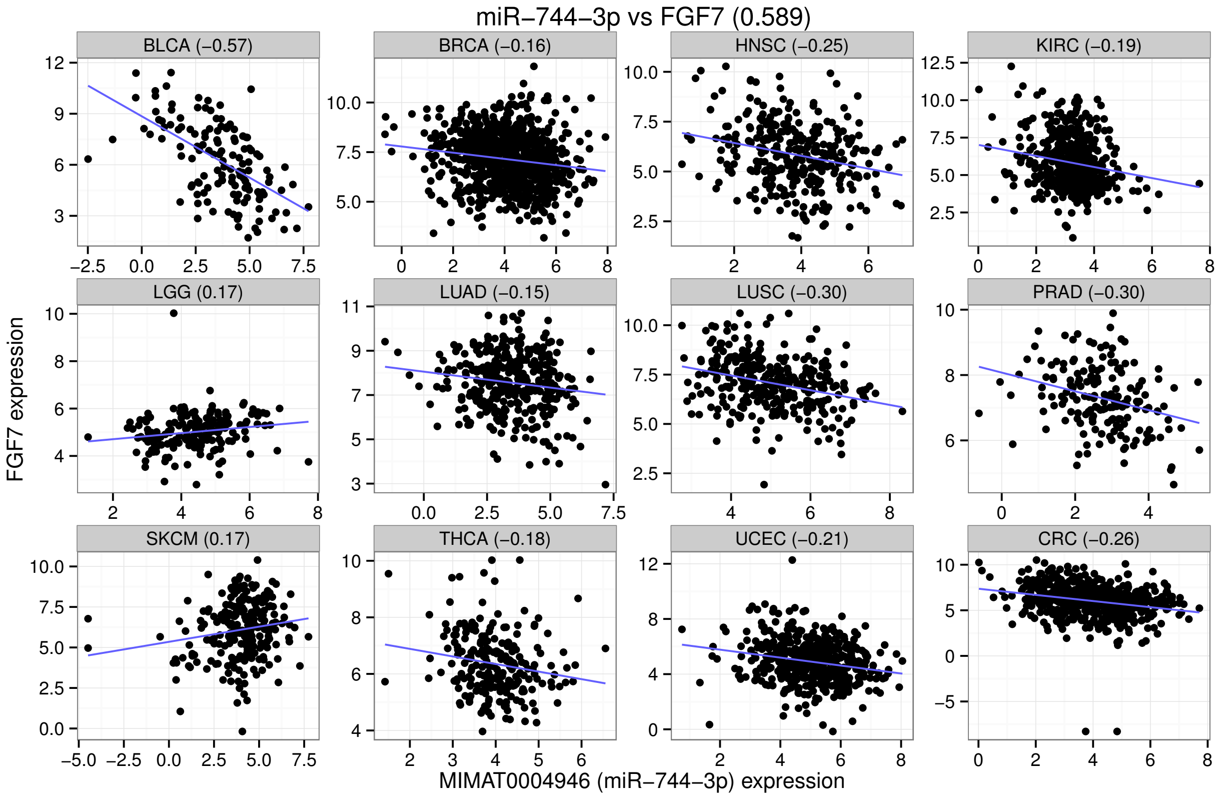
miR−744−3p vs FGF7 (0.589)
BLCA (−0.57)
BRCA (−0.16)
HNSC (−0.25)
KIRC (−0.19)
LGG (0.17)
LUAD (−0.15)
LUSC (−0.30)
PRAD (−0.30)
SKCM (0.17)
THCA (−0.18)
UCEC (−0.21)
CRC (−0.26)
FGF7 expression
MIMAT0004946 (miR−744−3p) expression

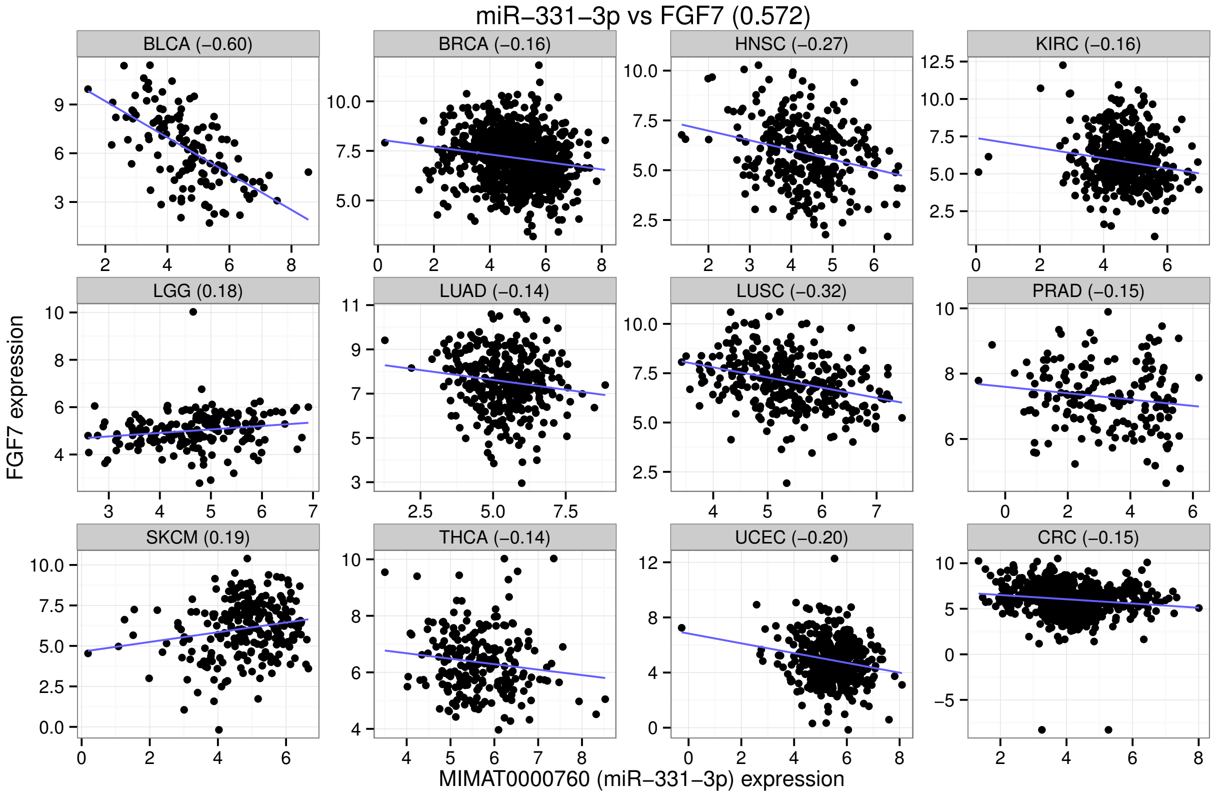
miR−331−3p vs FGF7 (0.572)
BLCA (−0.60)
BRCA (−0.16)
HNSC (−0.27)
KIRC (−0.16)
LGG (0.18)
LUAD (−0.14)
LUSC (−0.32)
PRAD (−0.15)
SKCM (0.19)
THCA (−0.14)
UCEC (−0.20)
CRC (−0.15)
FGF7 expression
MIMAT0000760 (miR−331−3p) expression

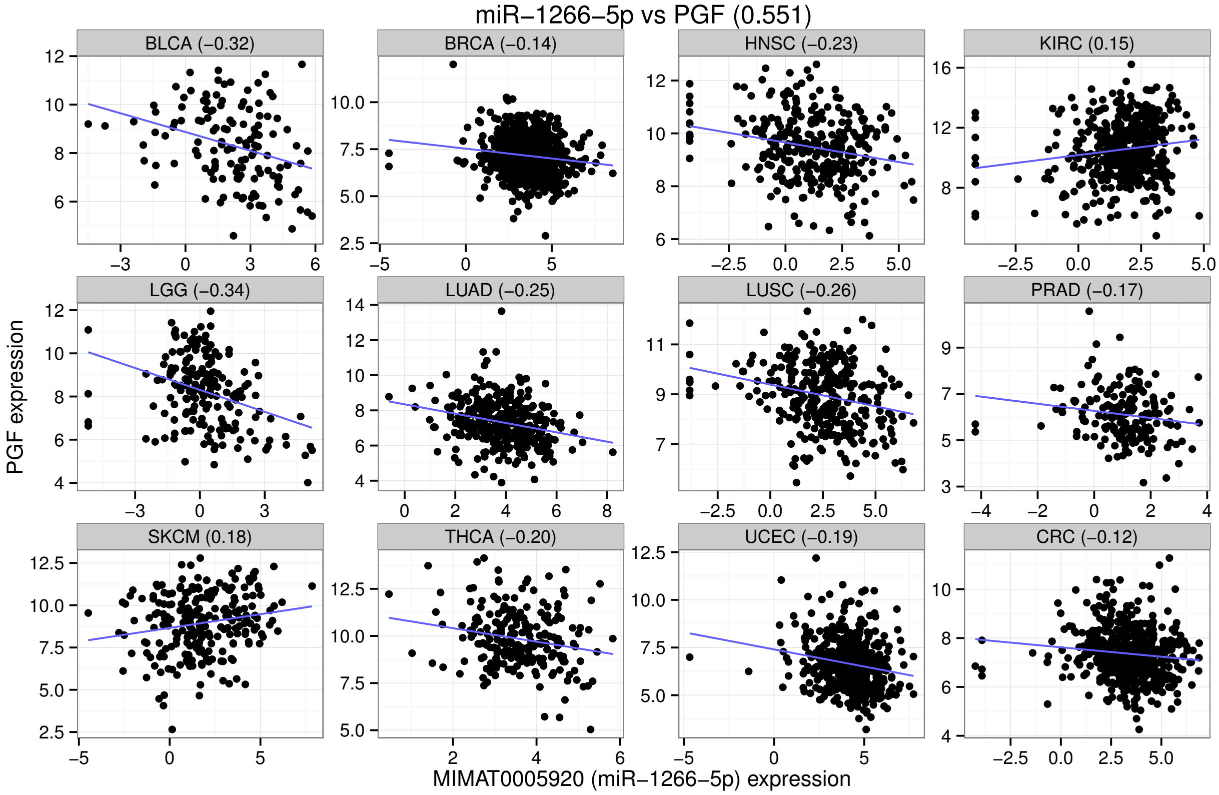
miR−1266−5p vs PGF (0.551)
BLCA (−0.32)
BRCA (−0.14)
HNSC (−0.23)
KIRC (0.15)
LGG (−0.34)
LUAD (−0.25)
LUSC (−0.26)
PRAD (−0.17)
SKCM (0.18)
THCA (−0.20)
UCEC (−0.19)
CRC (−0.12)
PGF expression
MIMAT0005920 (miR−1266−5p) expression

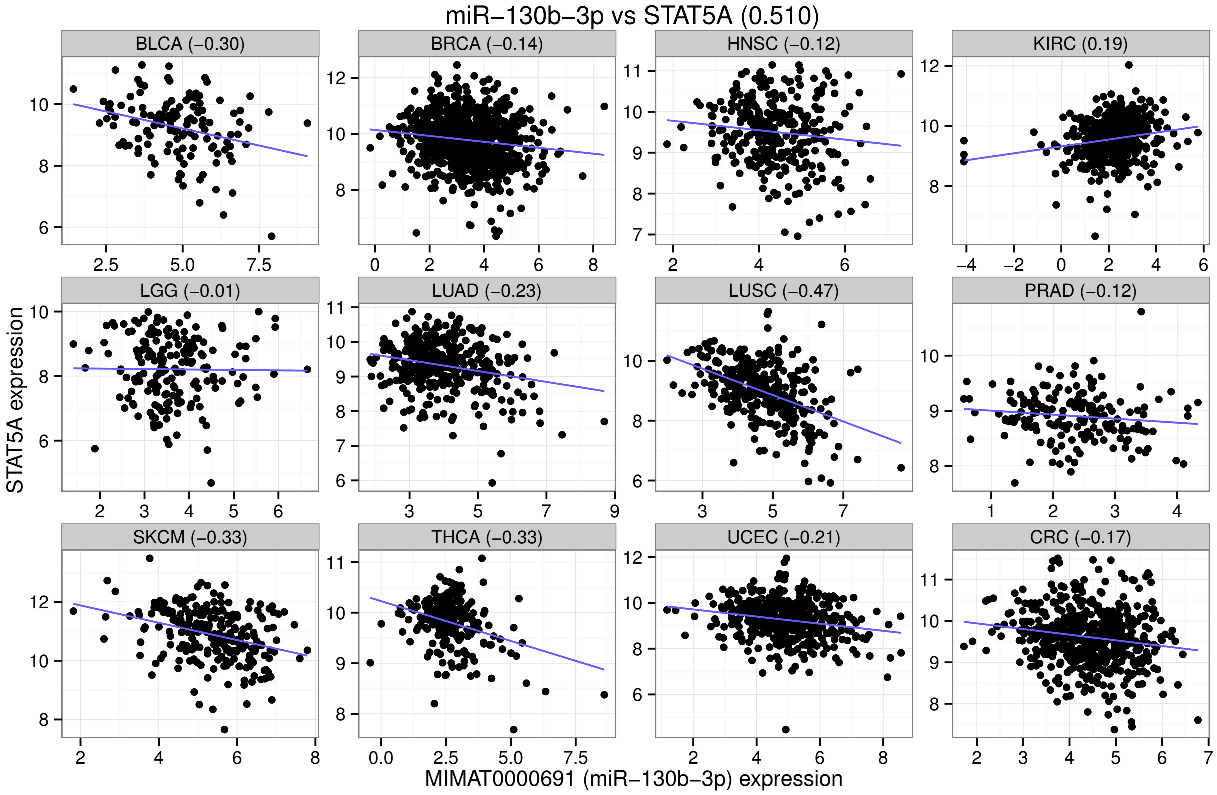
miR−130b−3p vs STAT5A (0.510)
BLCA (−0.30)
BRCA (−0.14)
HNSC (−0.12)
KIRC (0.19)
LGG (−0.01)
LUAD (−0.23)
LUSC (−0.47)
PRAD (−0.12)
SKCM (−0.33)
THCA (−0.33)
UCEC (−0.21)
CRC (−0.17)
STAT5A expression
MIMAT0000691 (miR−130b−3p) expression

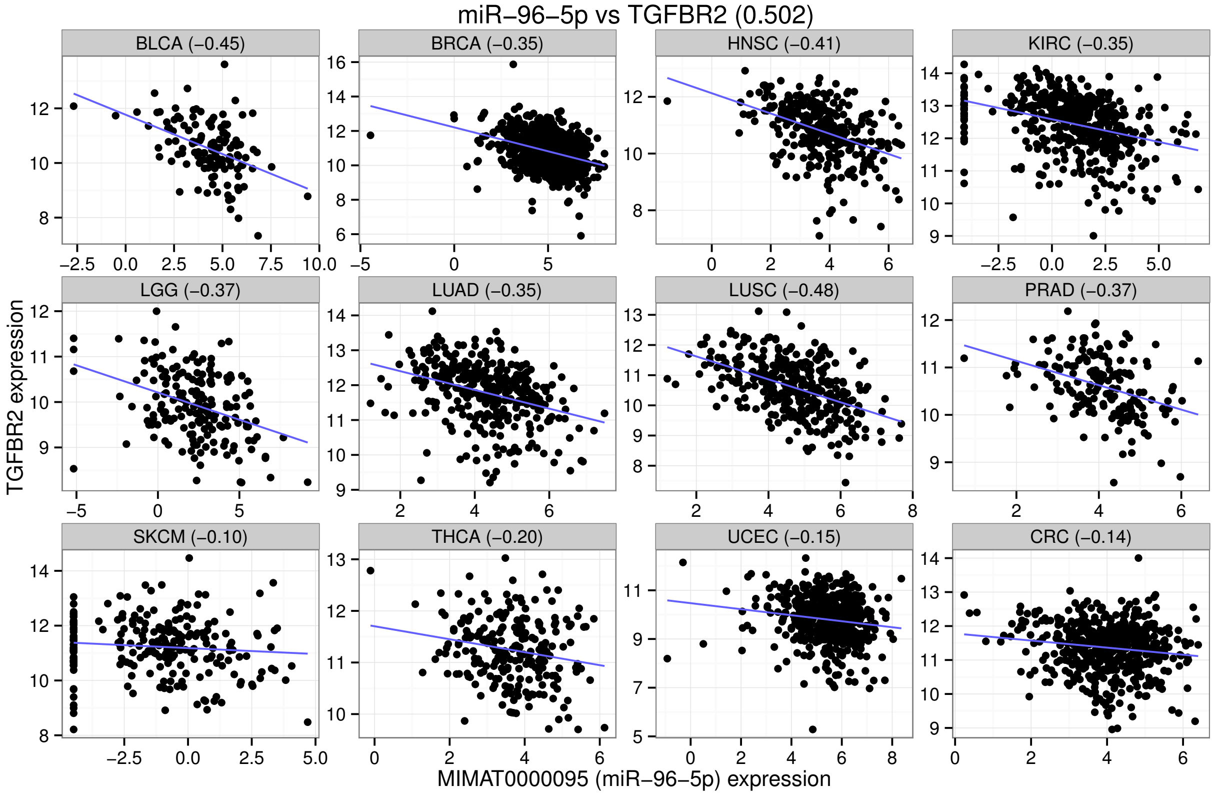
miR−96−5p vs TGFBR2 (0.502)
BLCA (−0.45)
BRCA (−0.35)
HNSC (−0.41)
KIRC (−0.35)
LGG (−0.37)
LUAD (−0.35)
LUSC (−0.48)
PRAD (−0.37)
SKCM (−0.10)
THCA (−0.20)
UCEC (−0.15)
CRC (−0.14)
TGFBR2 expression
MIMAT0000095 (miR−96−5p) expression

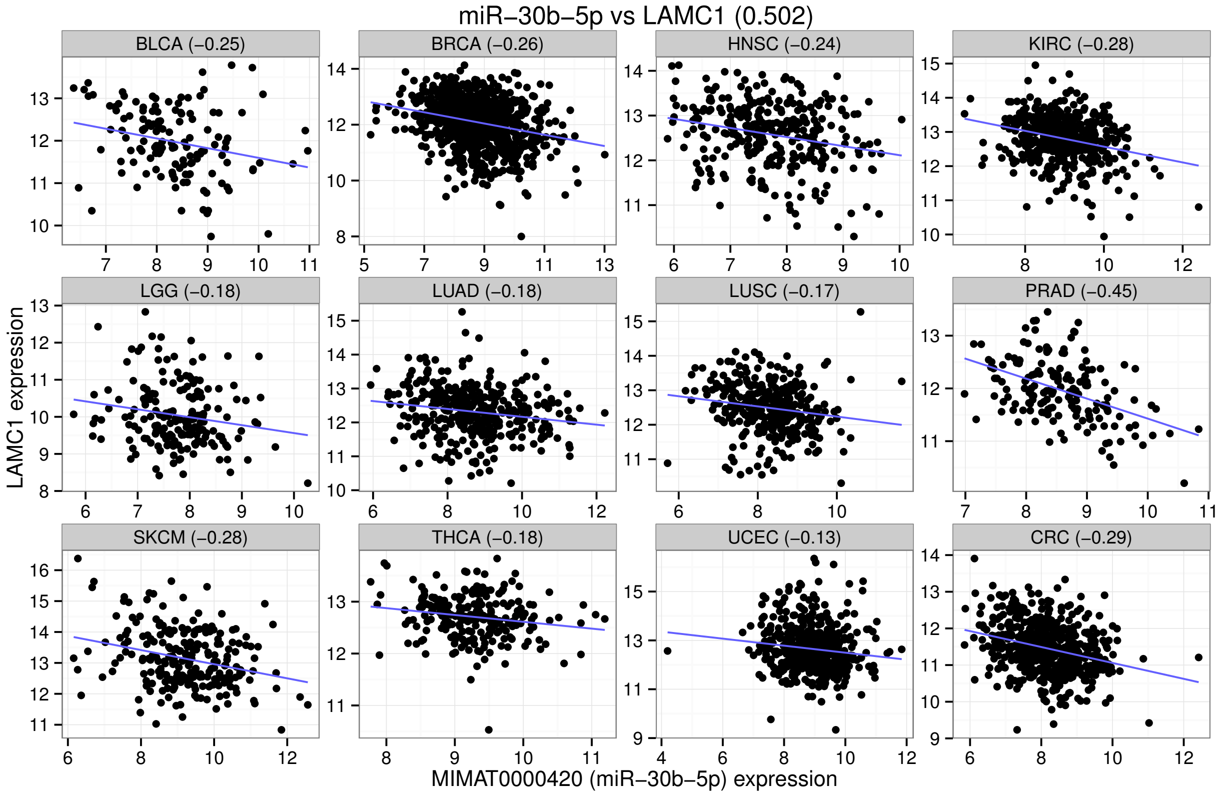
miR−30b−5p vs LAMC1 (0.502)
BLCA (−0.25)
BRCA (−0.26)
HNSC (−0.24)
KIRC (−0.28)
LGG (−0.18)
LUAD (−0.18)
LUSC (−0.17)
PRAD (−0.45)
SKCM (−0.28)
THCA (−0.18)
UCEC (−0.13)
CRC (−0.29)
LAMC1 expression
MIMAT0000420 (miR−30b−5p) expression

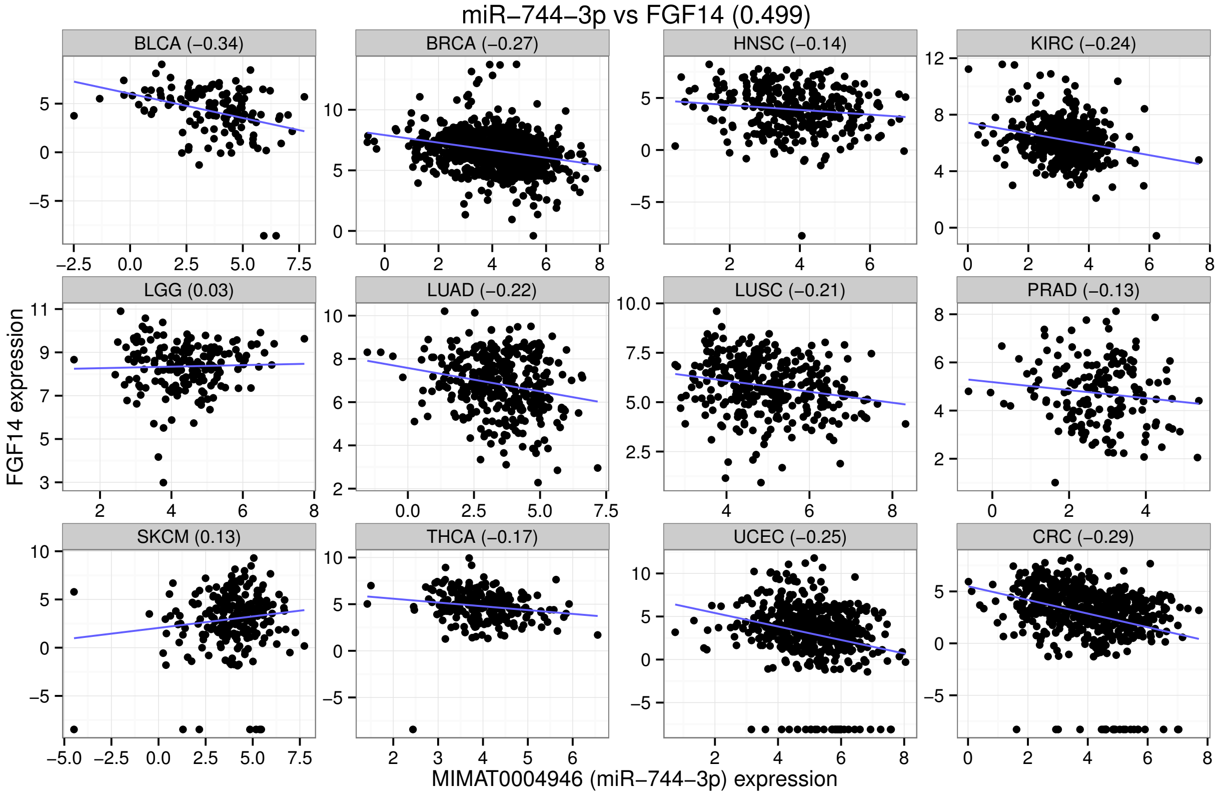

miR−744−3p vs FGF14 (0.499)
BLCA (−0.34)
BRCA (−0.27)
HNSC (−0.14)
KIRC (−0.24)
LGG (0.03)
LUAD (−0.22)
LUSC (−0.21)
PRAD (−0.13)
SKCM (0.13)
THCA (−0.17)
UCEC (−0.25)
CRC (−0.29)
FGF14 expression
MIMAT0004946 (miR−744−3p) expression

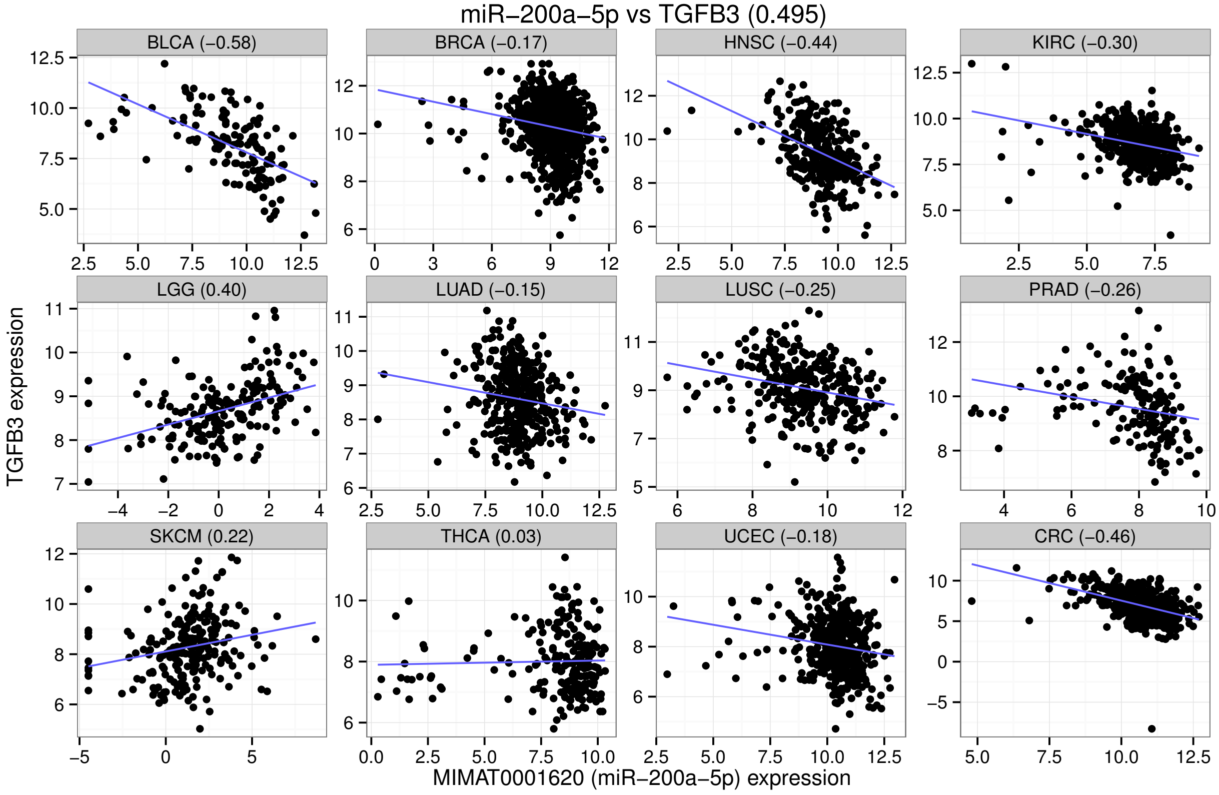

miR−200a−5p vs TGFB3 (0.495)
BLCA (−0.58)
BRCA (−0.17)
HNSC (−0.44)
KIRC (−0.30)
LGG (0.40)
LUAD (−0.15)
LUSC (−0.25)
PRAD (−0.26)
SKCM (0.22)
THCA (0.03)
UCEC (−0.18)
CRC (−0.46)
TGFB3 expression
MIMAT0001620 (miR−200a−5p) expression

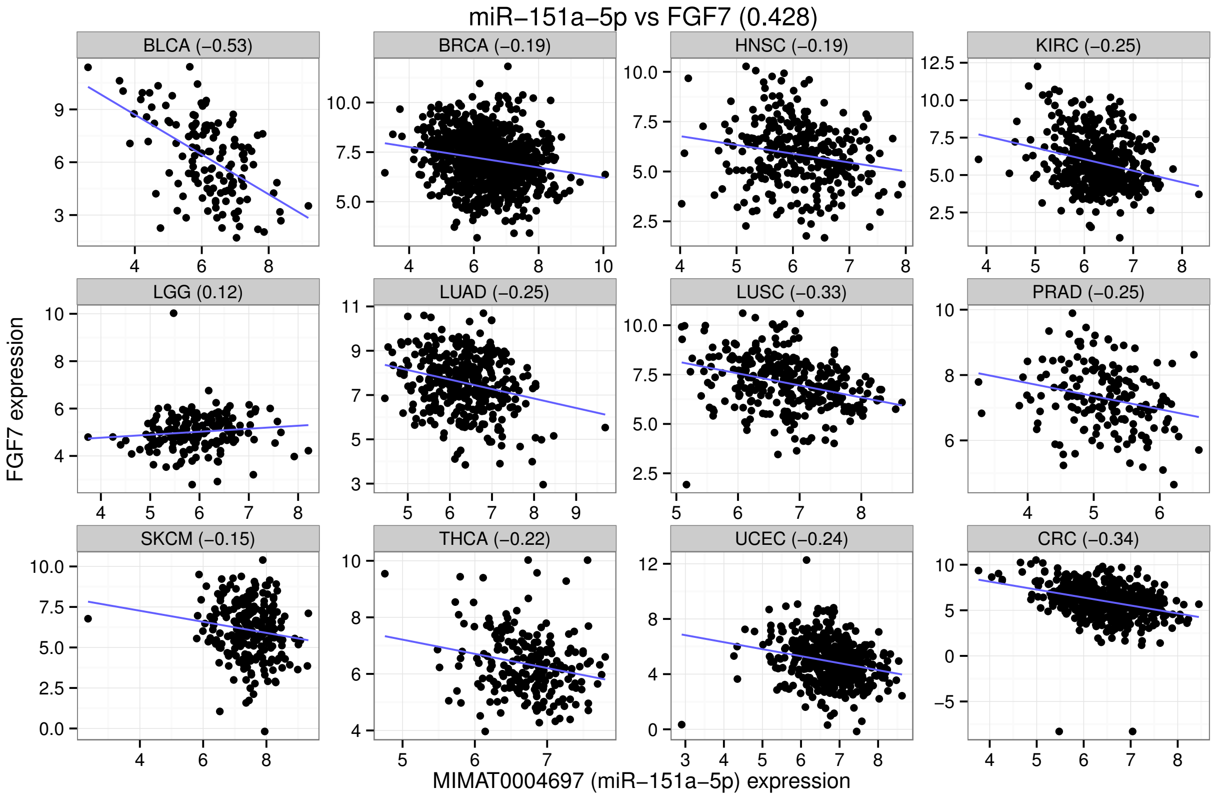
miR−151a−5p vs FGF7 (0.428)
BLCA (−0.53)
BRCA (−0.19)
HNSC (−0.19)
KIRC (−0.25)
LGG (0.12)
LUAD (−0.25)
LUSC (−0.33)
PRAD (−0.25)
SKCM (−0.15)
THCA (−0.22)
UCEC (−0.24)
CRC (−0.34)
FGF7 expression
MIMAT0004697 (miR−151a−5p) expression

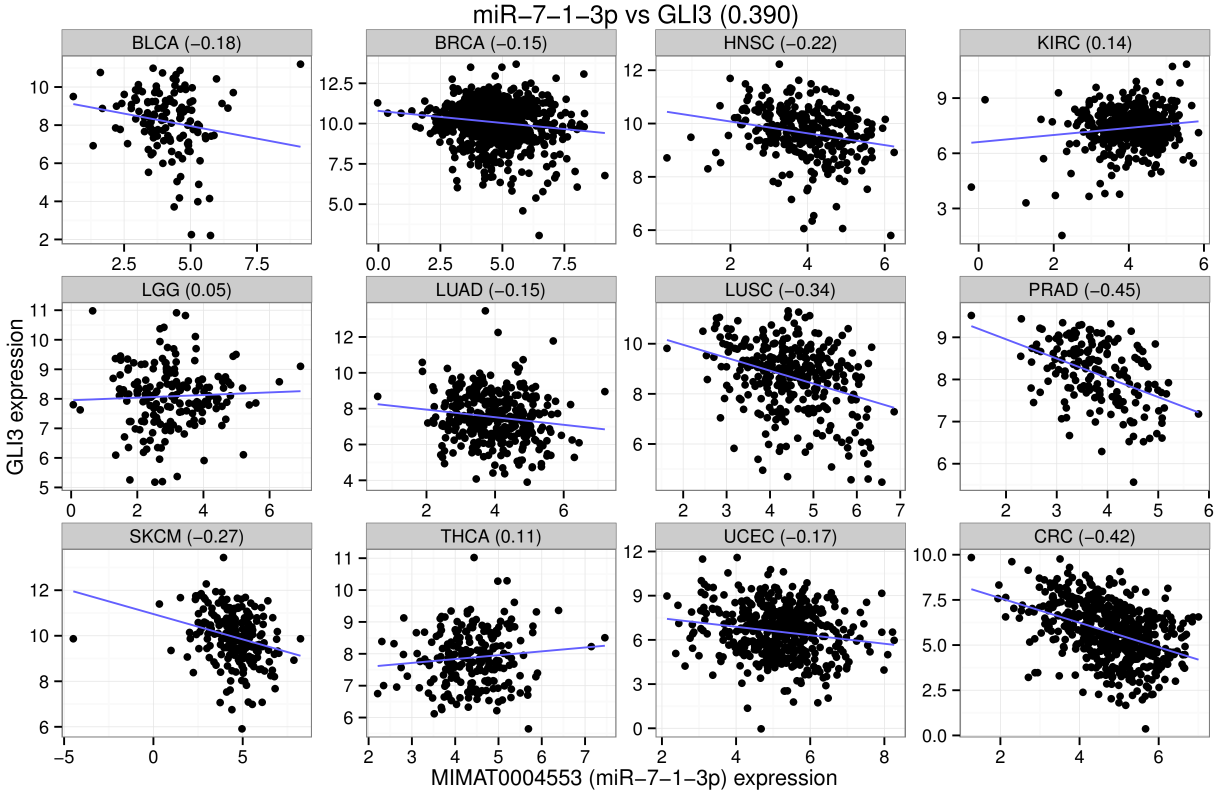
miR−7−1−3p vs GLI3 (0.390)
BLCA (−0.18)
BRCA (−0.15)
HNSC (−0.22)
KIRC (0.14)
LGG (0.05)
LUAD (−0.15)
LUSC (−0.34)
PRAD (−0.45)
SKCM (−0.27)
THCA (0.11)
UCEC (−0.17)
CRC (−0.42)
GLI3 expression
MIMAT0004553 (miR−7−1−3p) expression

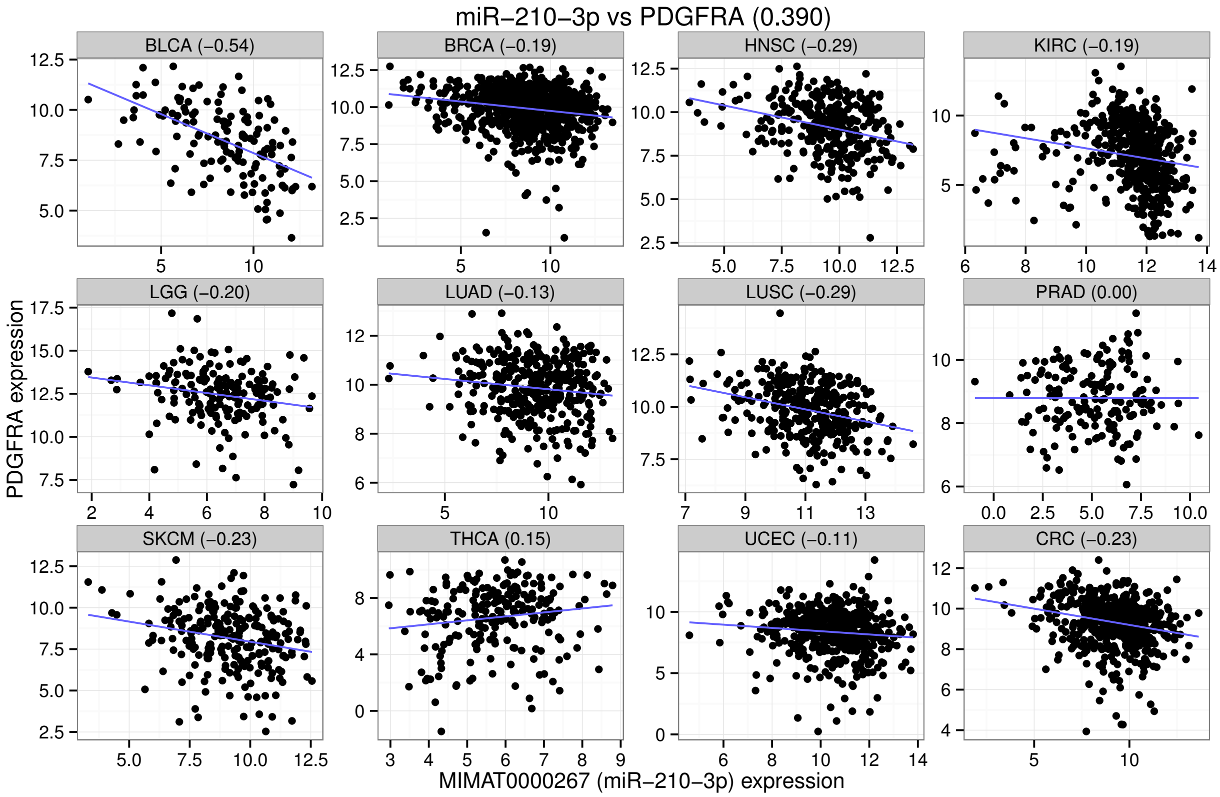
miR−210−3p vs PDGFRA (0.390)
BLCA (−0.54)
BRCA (−0.19)
HNSC (−0.29)
KIRC (−0.19)
LGG (−0.20)
LUAD (−0.13)
LUSC (−0.29)
PRAD (0.00)
SKCM (−0.23)
THCA (0.15)
UCEC (−0.11)
CRC (−0.23)
PDGFRA expression
MIMAT0000267 (miR−210−3p) expression

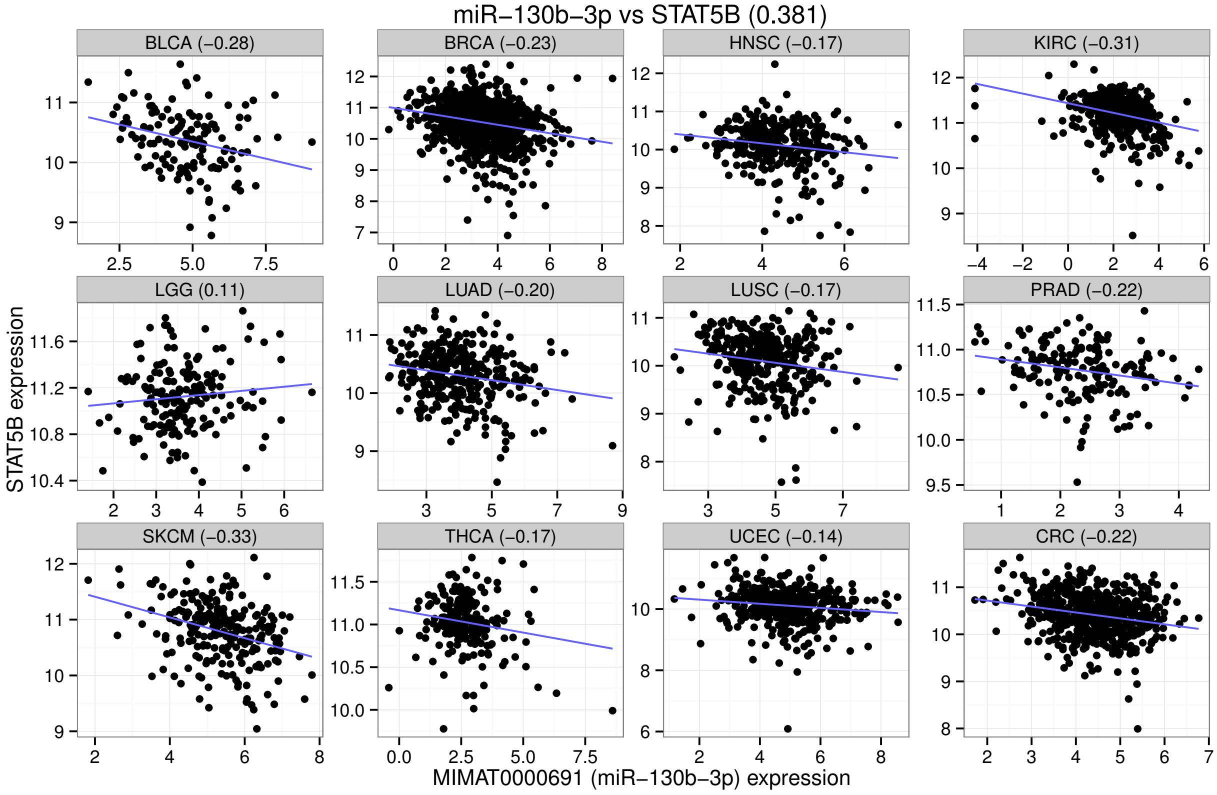
miR−130b−3p vs STAT5B (0.381)
BLCA (−0.28)
BRCA (−0.23)
HNSC (−0.17)
KIRC (−0.31)
LGG (0.11)
LUAD (−0.20)
LUSC (−0.17)
PRAD (−0.22)
SKCM (−0.33)
THCA (−0.17)
UCEC (−0.14)
CRC (−0.22)
STAT5B expression
MIMAT0000691 (miR−130b−3p) expression

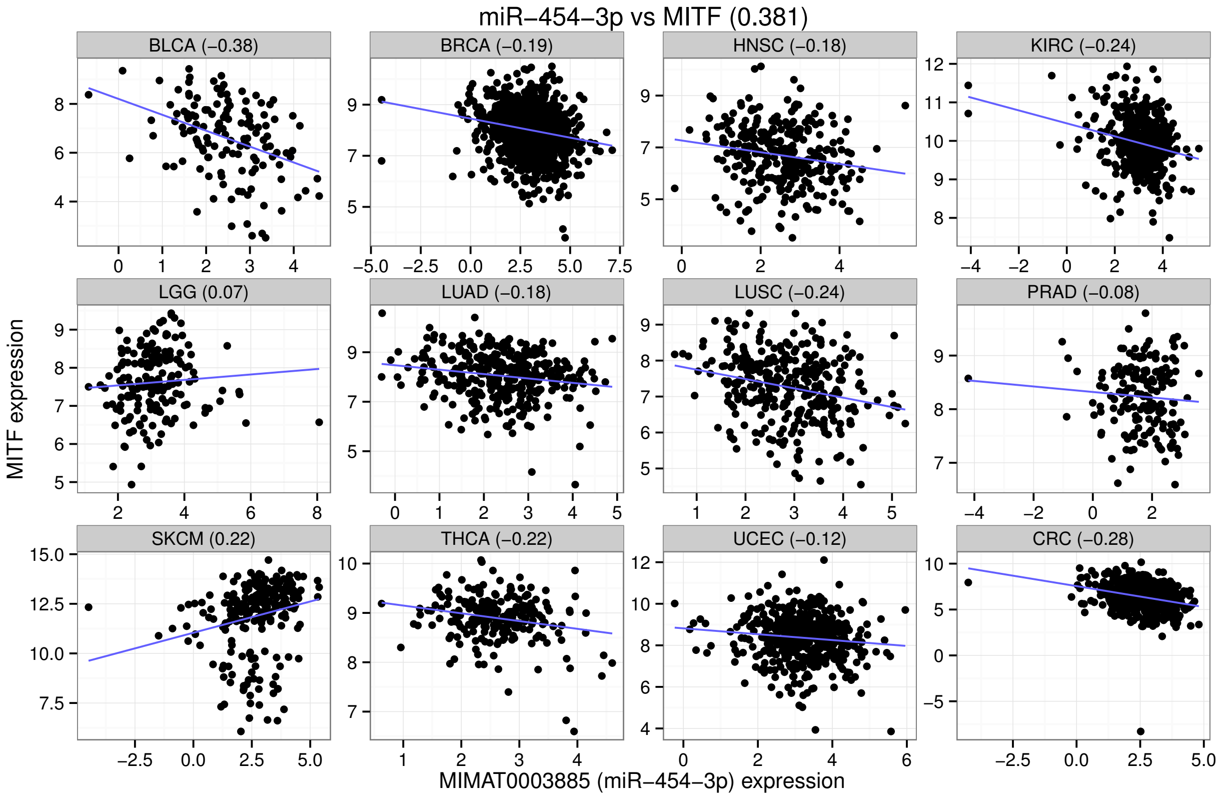

miR−454−3p vs MITF (0.381)
BLCA (−0.38)
BRCA (−0.19)
HNSC (−0.18)
KIRC (−0.24)
LGG (0.07)
LUAD (−0.18)
LUSC (−0.24)
PRAD (−0.08)
SKCM (0.22)
THCA (−0.22)
UCEC (−0.12)
CRC (−0.28)
MITF expression
MIMAT0003885 (miR−454−3p) expression

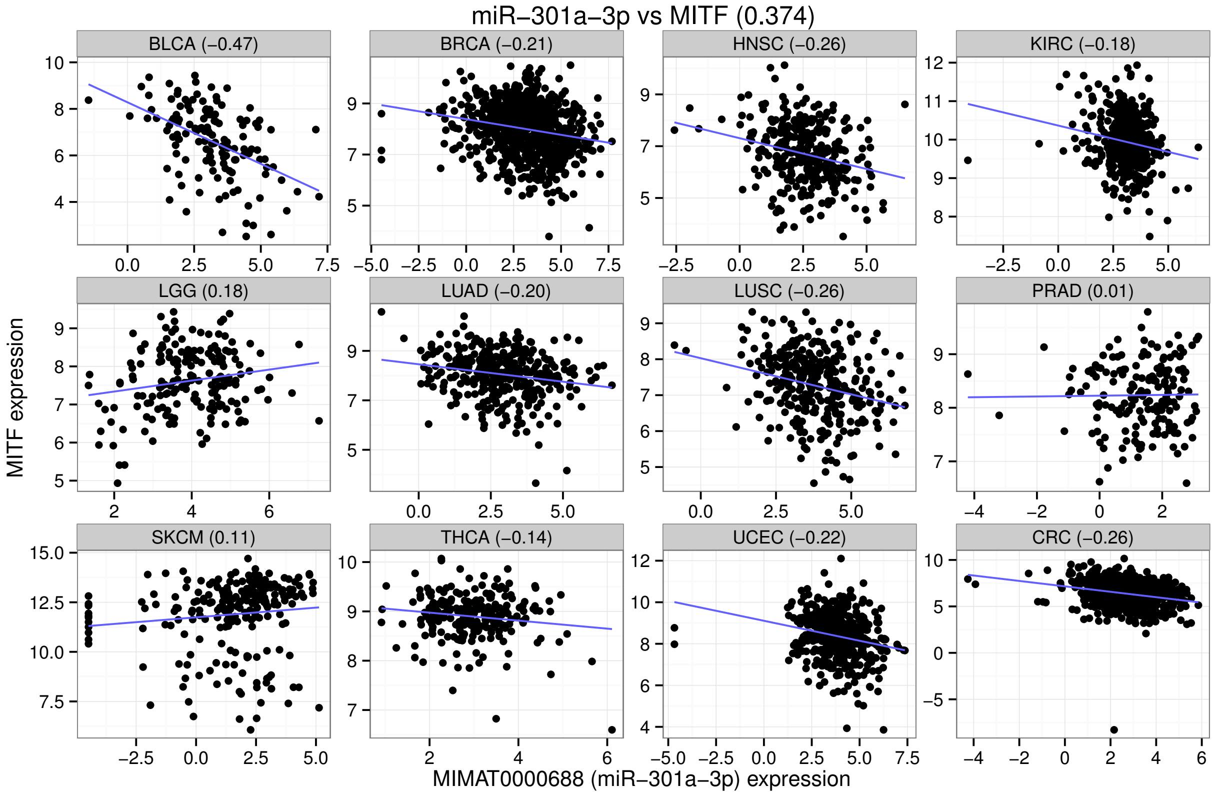

miR−301a−3p vs MITF (0.374)
BLCA (−0.47)
BRCA (−0.21)
HNSC (−0.26)
KIRC (−0.18)
LGG (0.18)
LUAD (−0.20)
LUSC (−0.26)
PRAD (0.01)
SKCM (0.11)
THCA (−0.14)
UCEC (−0.22)
CRC (−0.26)
MITF expression
MIMAT0000688 (miR−301a−3p) expression

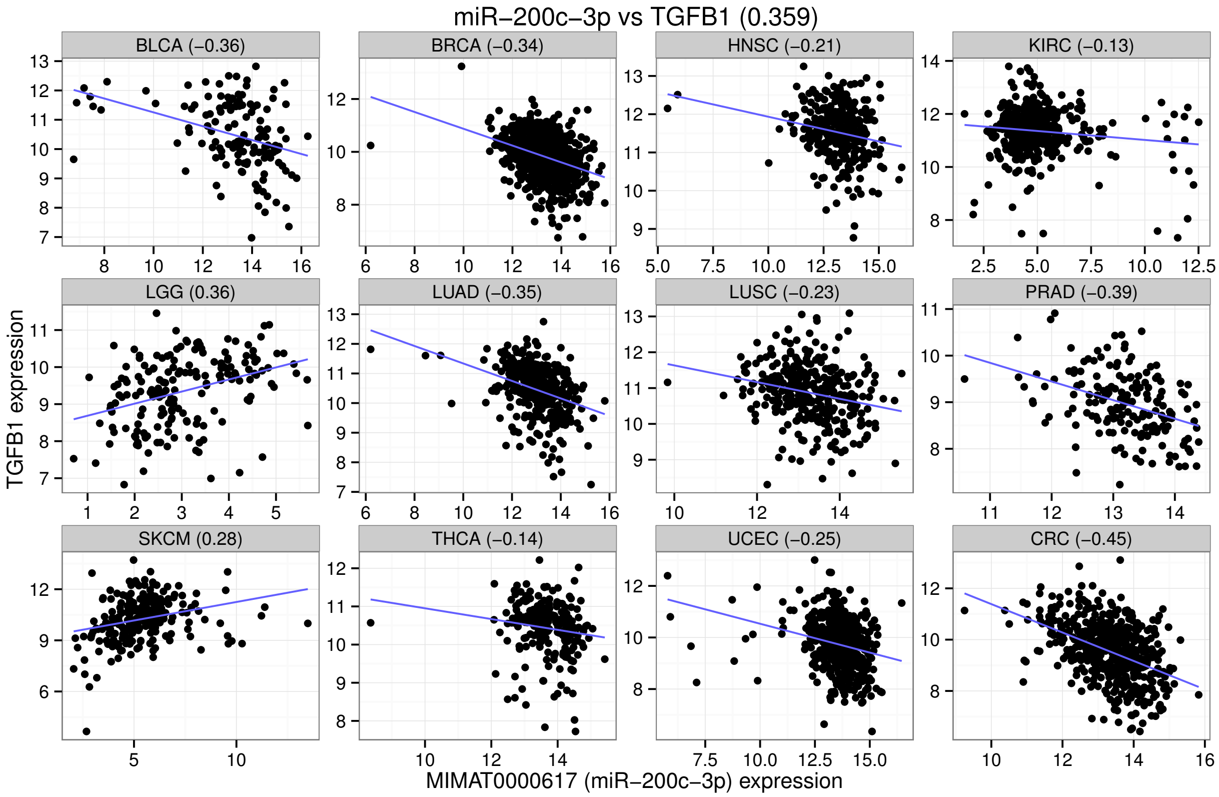
miR−200c−3p vs TGFB1 (0.359)
BLCA (−0.36)
BRCA (−0.34)
HNSC (−0.21)
KIRC (−0.13)
LGG (0.36)
LUAD (−0.35)
LUSC (−0.23)
PRAD (−0.39)
SKCM (0.28)
THCA (−0.14)
UCEC (−0.25)
CRC (−0.45)
TGFB1 expression
MIMAT0000617 (miR−200c−3p) expression

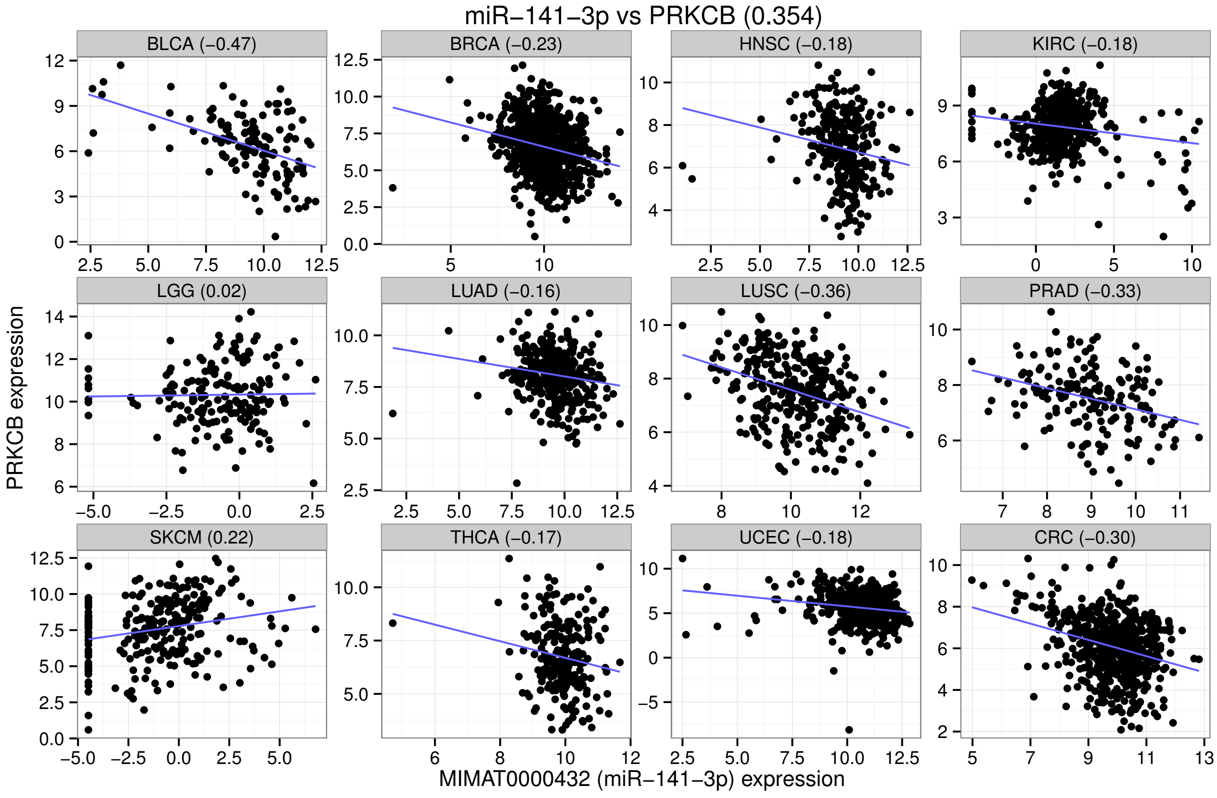
miR−141−3p vs PRKCB (0.354)
BLCA (−0.47)
BRCA (−0.23)
HNSC (−0.18)
KIRC (−0.18)
LGG (0.02)
LUAD (−0.16)
LUSC (−0.36)
PRAD (−0.33)
SKCM (0.22)
THCA (−0.17)
UCEC (−0.18)
CRC (−0.30)
PRKCB expression
MIMAT0000432 (miR−141−3p) expression

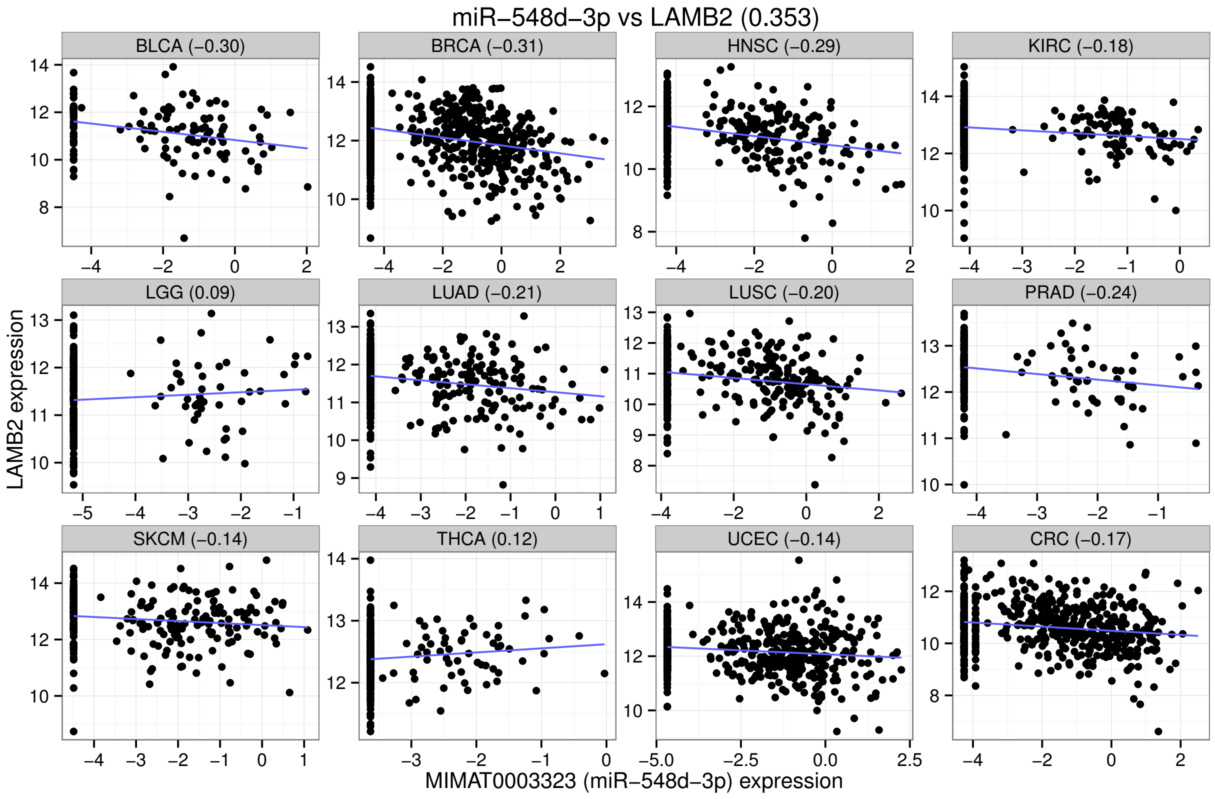
miR−548d−3p vs LAMB2 (0.353)
BLCA (−0.30)
BRCA (−0.31)
HNSC (−0.29)
KIRC (−0.18)
LGG (0.09)
LUAD (−0.21)
LUSC (−0.20)
PRAD (−0.24)
SKCM (−0.14)
THCA (0.12)
UCEC (−0.14)
CRC (−0.17)
LAMB2 expression
MIMAT0003323 (miR−548d−3p) expression

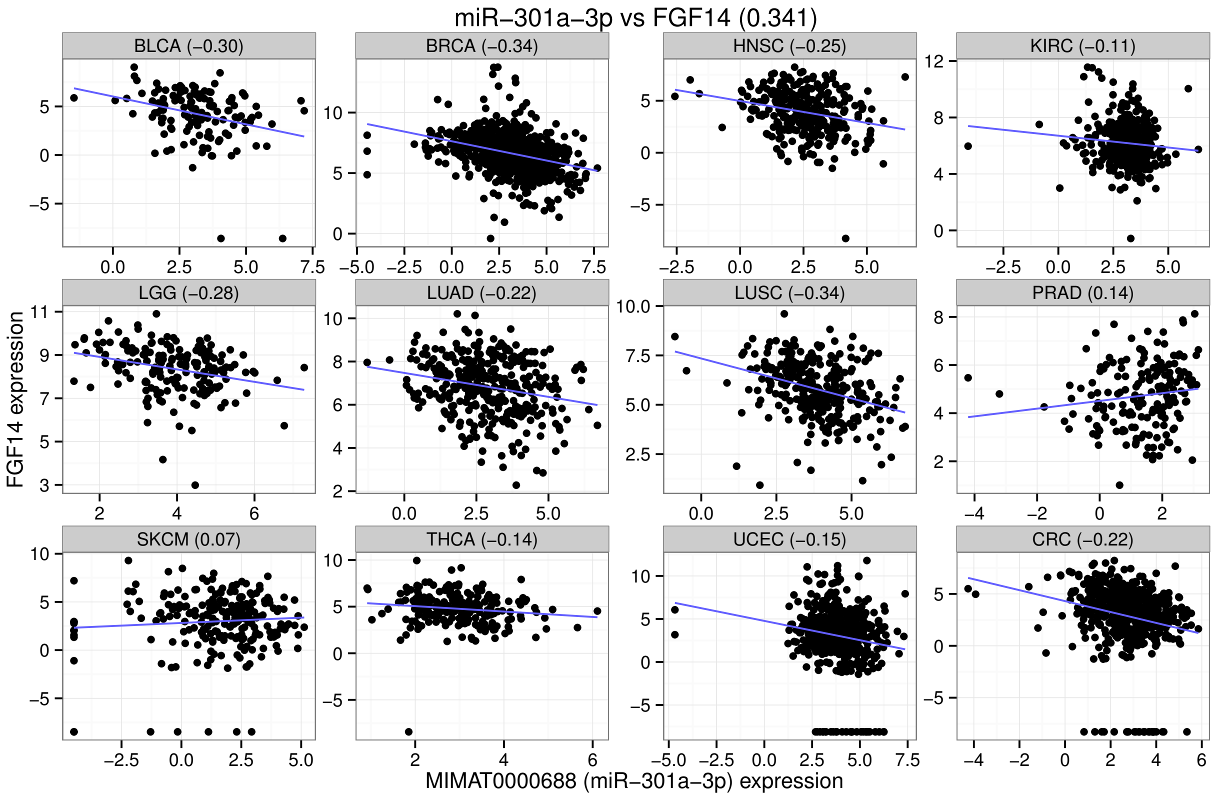
miR−301a−3p vs FGF14 (0.341)
BLCA (−0.30)
BRCA (−0.34)
HNSC (−0.25)
KIRC (−0.11)
LGG (−0.28)
LUAD (−0.22)
LUSC (−0.34)
PRAD (0.14)
SKCM (0.07)
THCA (−0.14)
UCEC (−0.15)
CRC (−0.22)
FGF14 expression
MIMAT0000688 (miR−301a−3p) expression

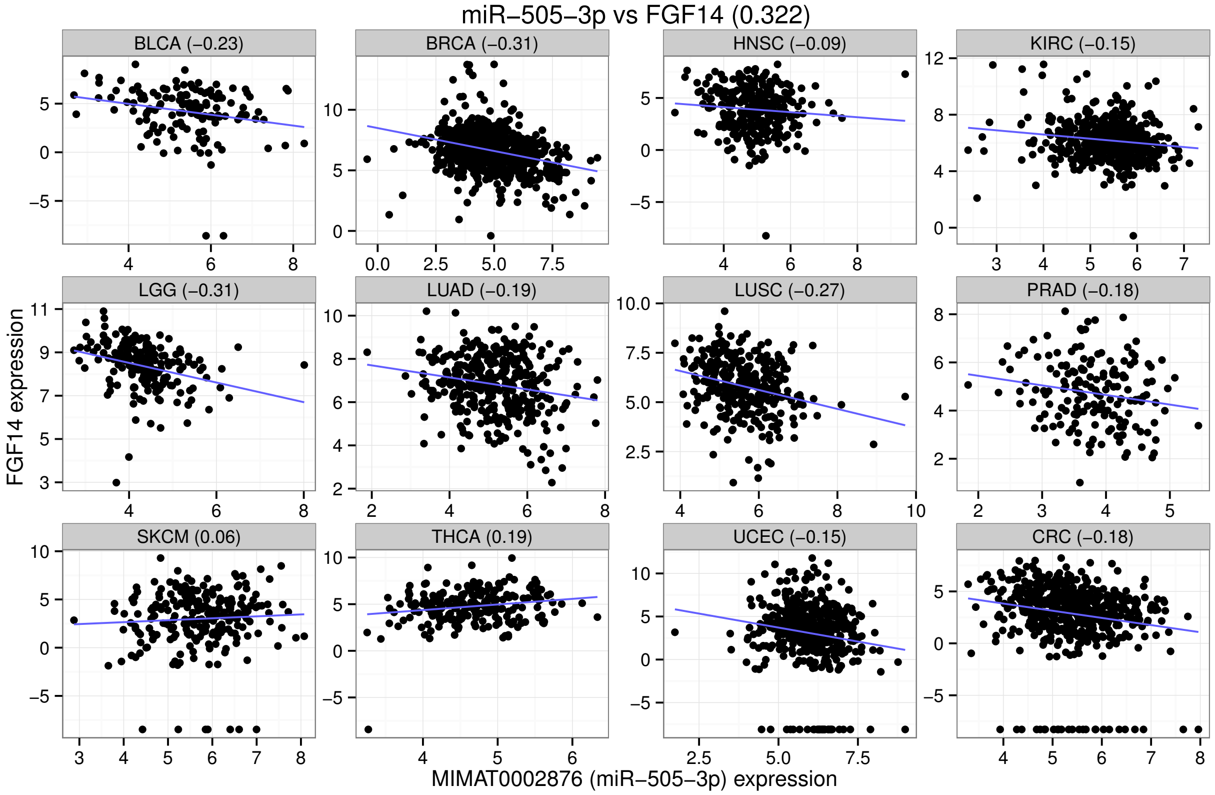
miR−505−3p vs FGF14 (0.322)
BLCA (−0.23)
BRCA (−0.31)
HNSC (−0.09)
KIRC (−0.15)
LGG (−0.31)
LUAD (−0.19)
LUSC (−0.27)
PRAD (−0.18)
SKCM (0.06)
THCA (0.19)
UCEC (−0.15)
CRC (−0.18)
FGF14 expression
MIMAT0002876 (miR−505−3p) expression

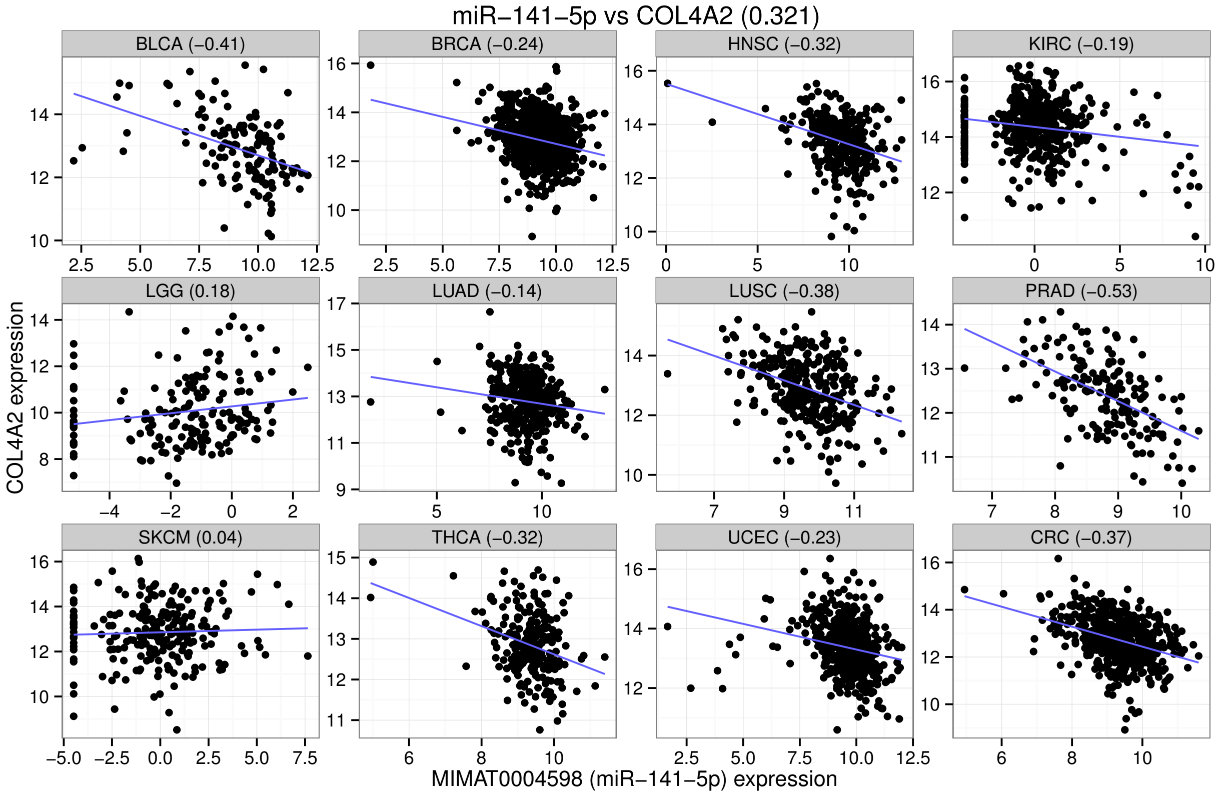
miR−141−5p vs COL4A2 (0.321)
BLCA (−0.41)
BRCA (−0.24)
HNSC (−0.32)
KIRC (−0.19)
LGG (0.18)
LUAD (−0.14)
LUSC (−0.38)
PRAD (−0.53)
SKCM (0.04)
THCA (−0.32)
UCEC (−0.23)
CRC (−0.37)
COL4A2 expression
MIMAT0004598 (miR−141−5p) expression

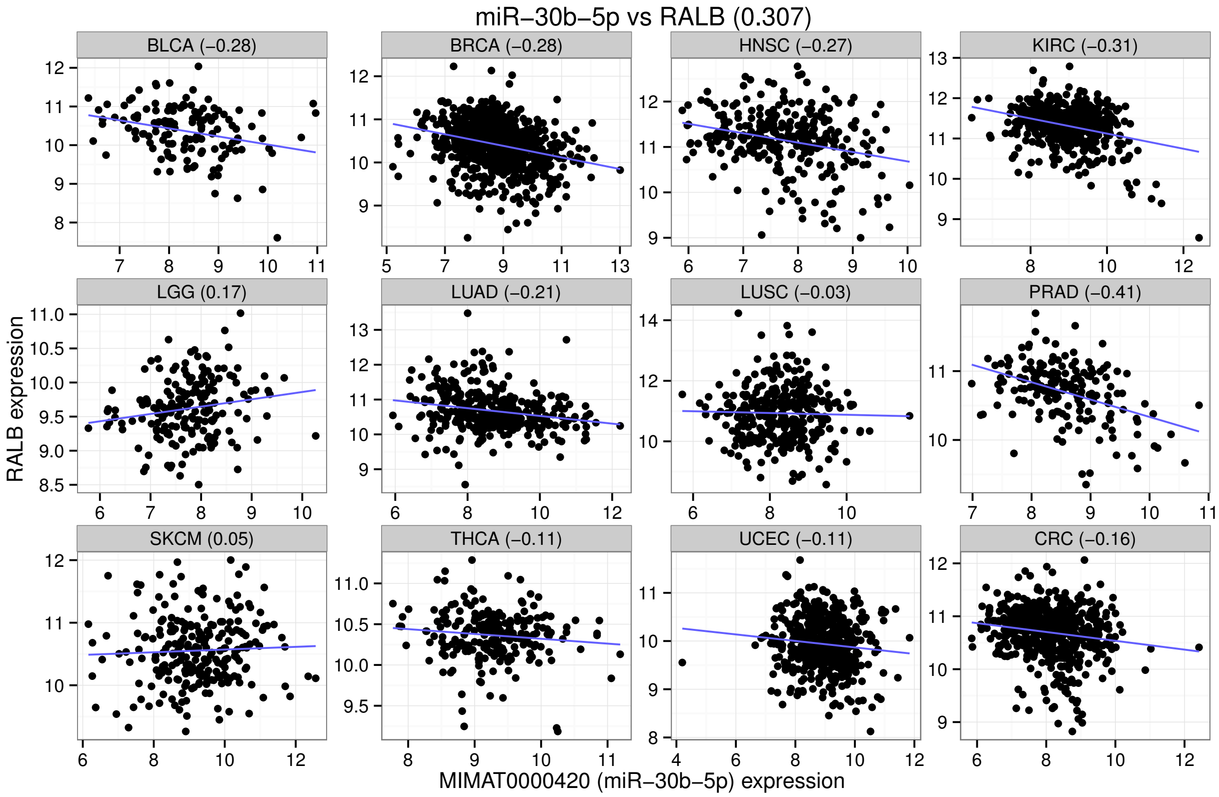
miR−30b−5p vs RALB (0.307)
BLCA (−0.28)
BRCA (−0.28)
HNSC (−0.27)
KIRC (−0.31)
LGG (0.17)
LUAD (−0.21)
LUSC (−0.03)
PRAD (−0.41)
SKCM (0.05)
THCA (−0.11)
UCEC (−0.11)
CRC (−0.16)
RALB expression
MIMAT0000420 (miR−30b−5p) expression

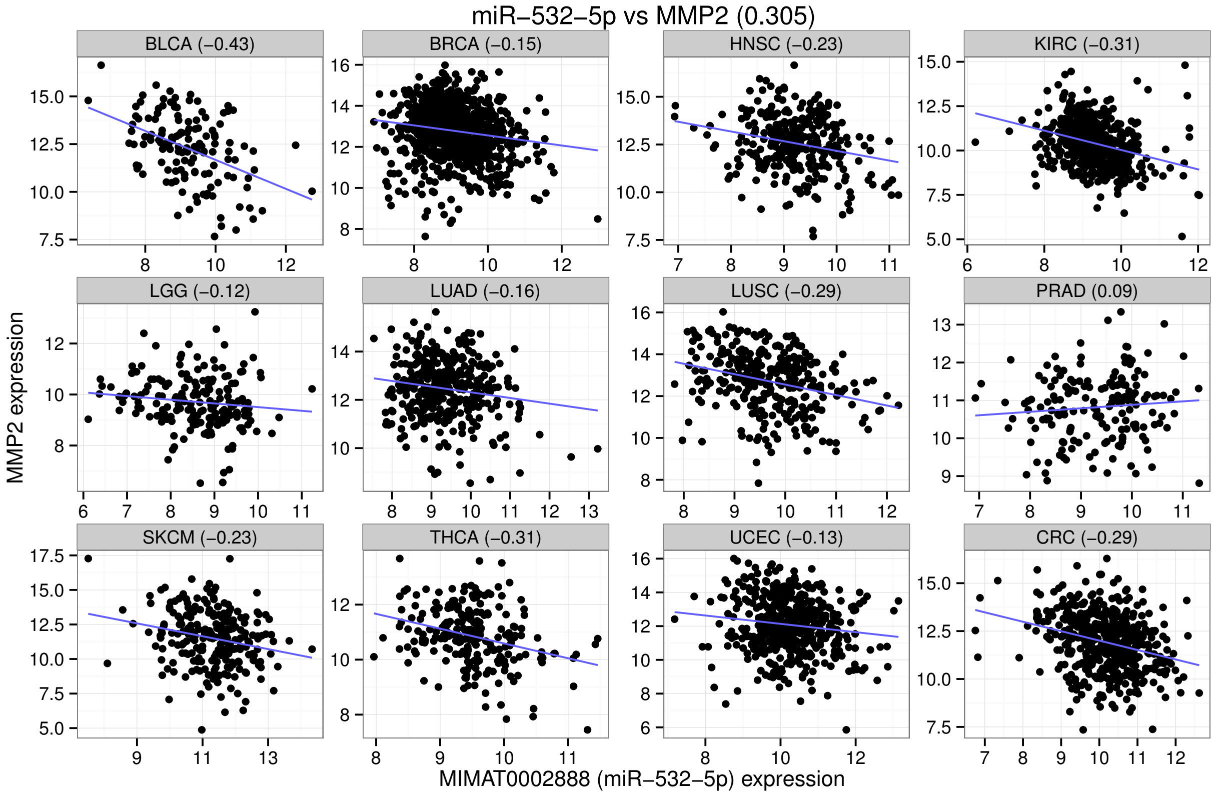
miR−532−5p vs MMP2 (0.305)
BLCA (−0.43)
BRCA (−0.15)
HNSC (−0.23)
KIRC (−0.31)
LGG (−0.12)
LUAD (−0.16)
LUSC (−0.29)
PRAD (0.09)
SKCM (−0.23)
THCA (−0.31)
UCEC (−0.13)
CRC (−0.29)
MMP2 expression
MIMAT0002888 (miR−532−5p) expression

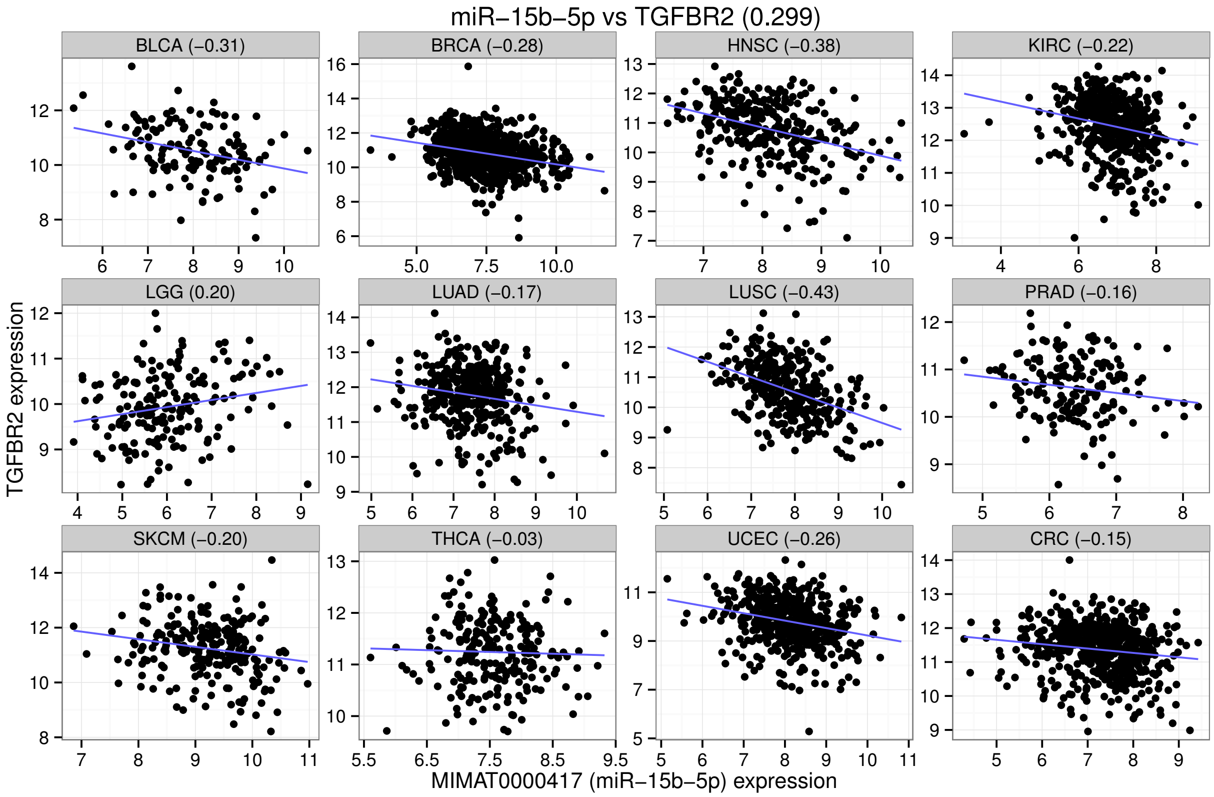
miR−15b−5p vs TGFBR2 (0.299)
BLCA (−0.31)
BRCA (−0.28)
HNSC (−0.38)
KIRC (−0.22)
LGG (0.20)
LUAD (−0.17)
LUSC (−0.43)
PRAD (−0.16)
SKCM (−0.20)
THCA (−0.03)
UCEC (−0.26)
CRC (−0.15)
TGFBR2 expression
MIMAT0000417 (miR−15b−5p) expression

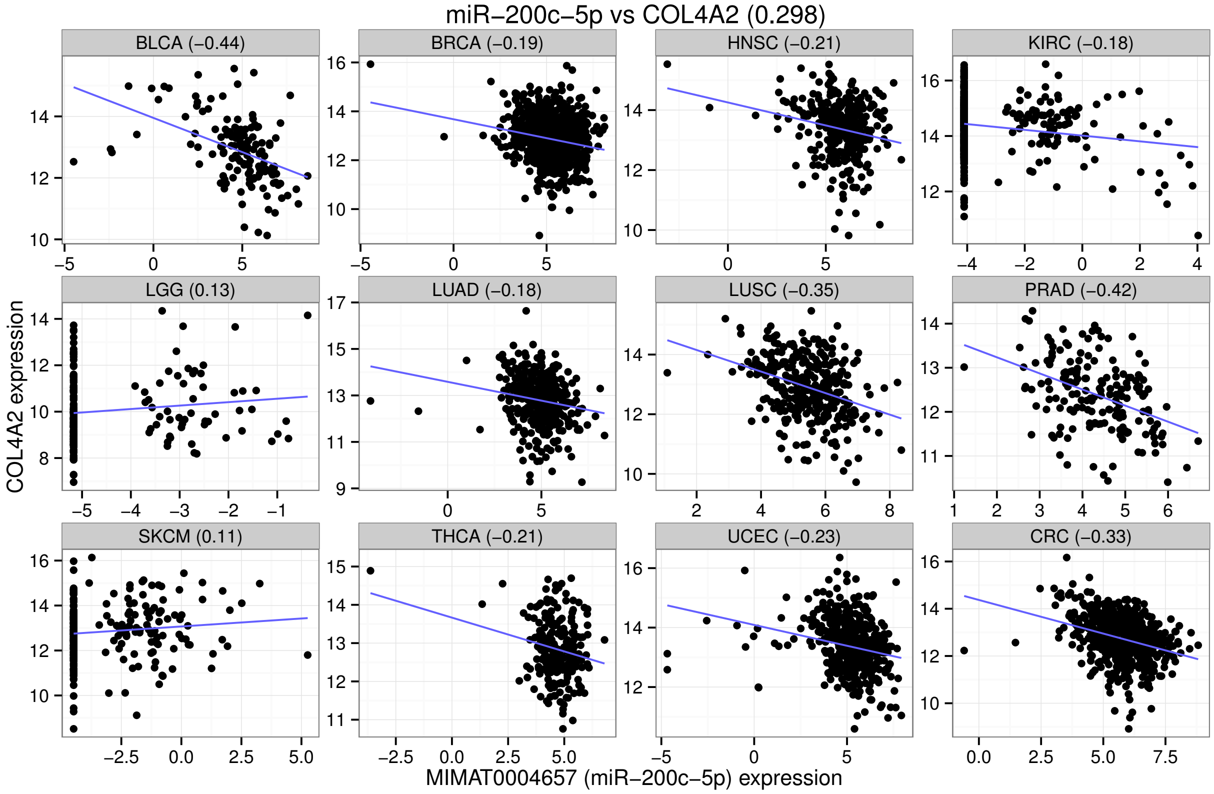
miR−200c−5p vs COL4A2 (0.298)
BLCA (−0.44)
BRCA (−0.19)
HNSC (−0.21)
KIRC (−0.18)
LGG (0.13)
LUAD (−0.18)
LUSC (−0.35)
PRAD (−0.42)
SKCM (0.11)
THCA (−0.21)
UCEC (−0.23)
CRC (−0.33)
COL4A2 expression
MIMAT0004657 (miR−200c−5p) expression

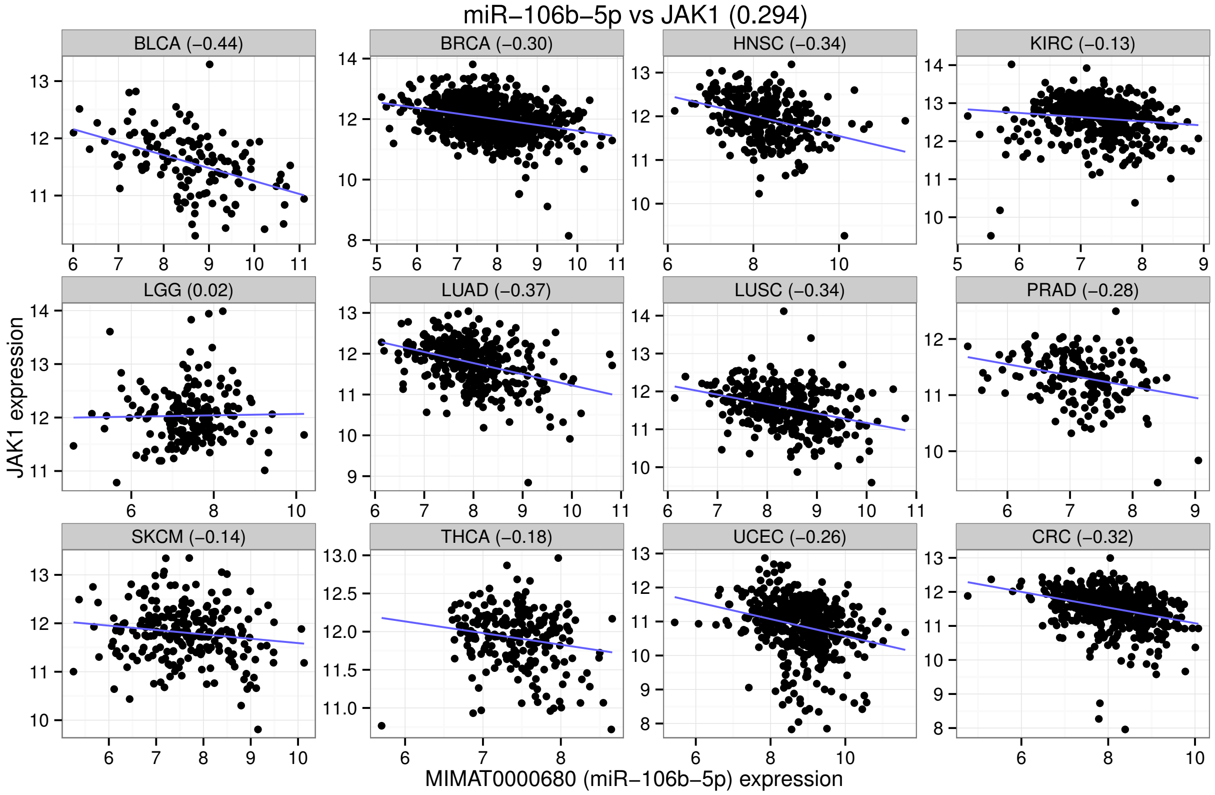
miR−106b−5p vs JAK1 (0.294)
BLCA (−0.44)
BRCA (−0.30)
HNSC (−0.34)
KIRC (−0.13)
LGG (0.02)
LUAD (−0.37)
LUSC (−0.34)
PRAD (−0.28)
SKCM (−0.14)
THCA (−0.18)
UCEC (−0.26)
CRC (−0.32)
JAK1 expression
MIMAT0000680 (miR−106b−5p) expression

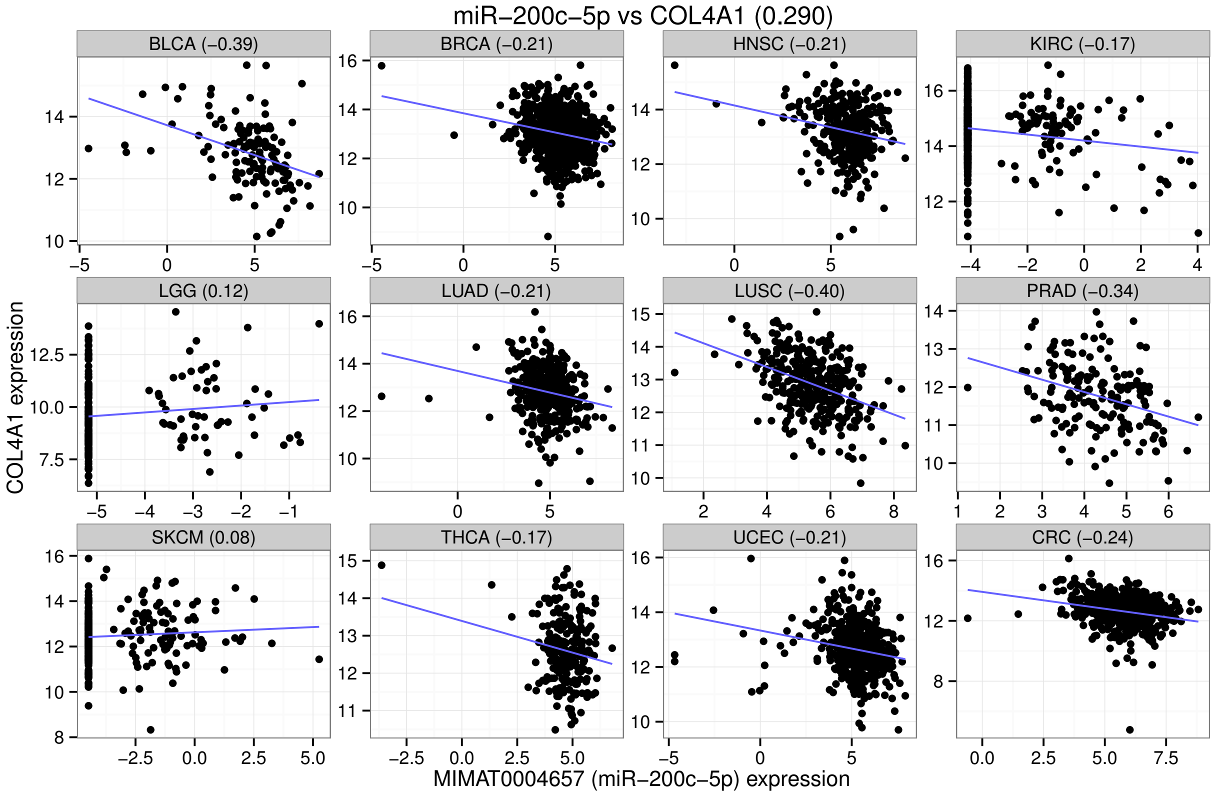
miR−200c−5p vs COL4A1 (0.290)
BLCA (−0.39)
BRCA (−0.21)
HNSC (−0.21)
KIRC (−0.17)
LGG (0.12)
LUAD (−0.21)
LUSC (−0.40)
PRAD (−0.34)
SKCM (0.08)
THCA (−0.17)
UCEC (−0.21)
CRC (−0.24)
COL4A1 expression
MIMAT0004657 (miR−200c−5p) expression

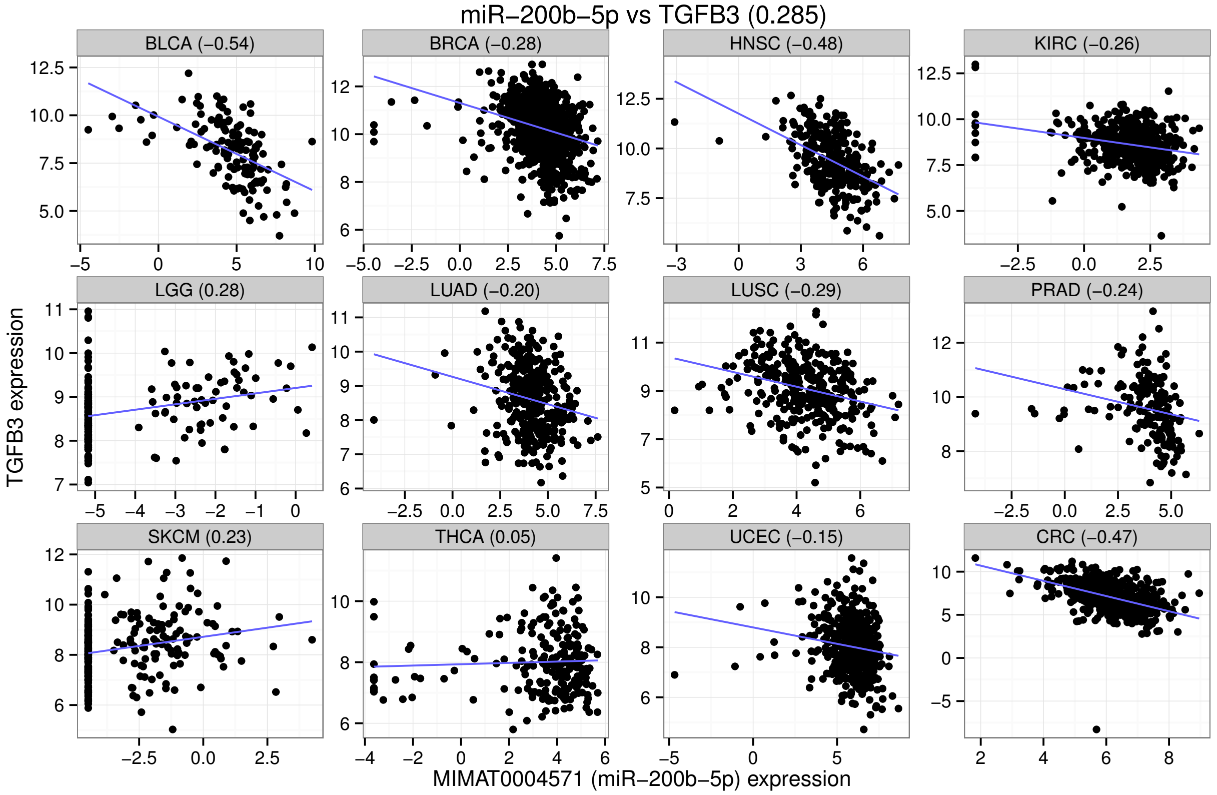
miR−200b−5p vs TGFB3 (0.285)
BLCA (−0.54)
BRCA (−0.28)
HNSC (−0.48)
KIRC (−0.26)
LGG (0.28)
LUAD (−0.20)
LUSC (−0.29)
PRAD (−0.24)
SKCM (0.23)
THCA (0.05)
UCEC (−0.15)
CRC (−0.47)
TGFB3 expression
MIMAT0004571 (miR−200b−5p) expression

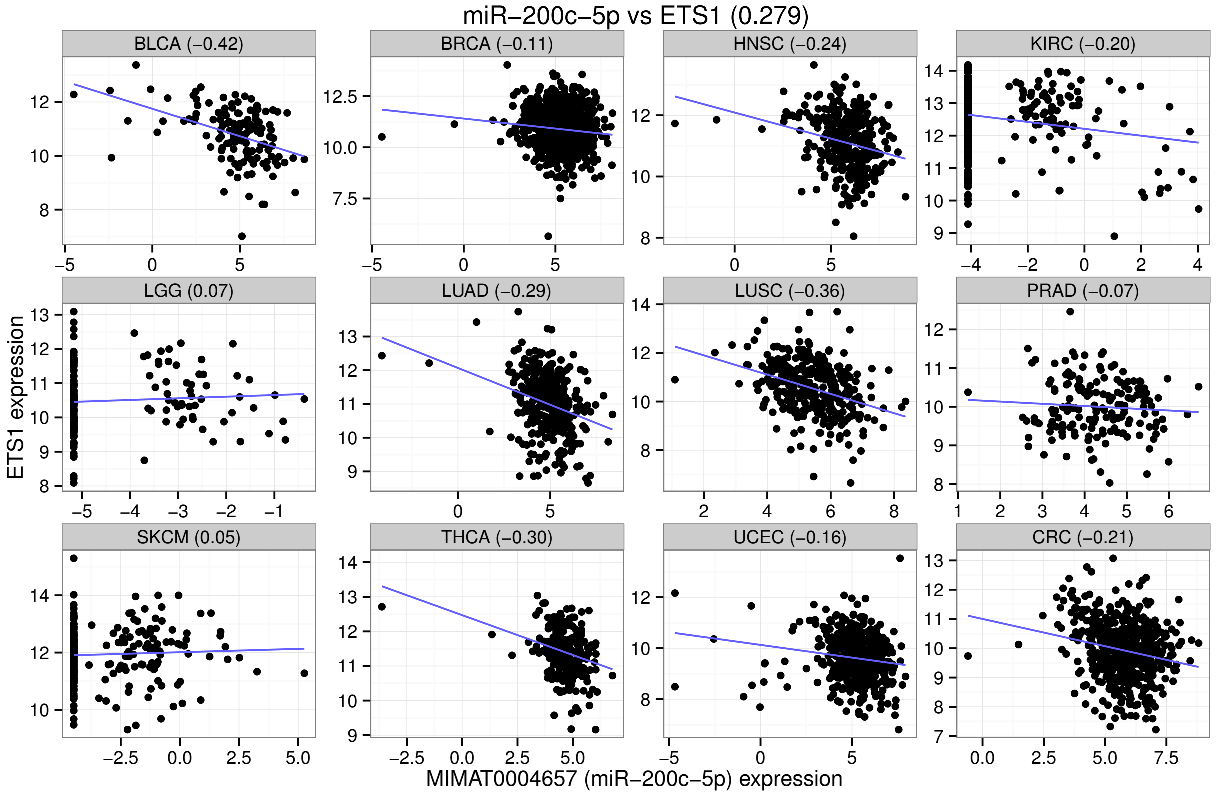
miR−200c−5p vs ETS1 (0.279)
BLCA (−0.42)
BRCA (−0.11)
HNSC (−0.24)
KIRC (−0.20)
LGG (0.07)
LUAD (−0.29)
LUSC (−0.36)
PRAD (−0.07)
SKCM (0.05)
THCA (−0.30)
UCEC (−0.16)
CRC (−0.21)
ETS1 expression
MIMAT0004657 (miR−200c−5p) expression

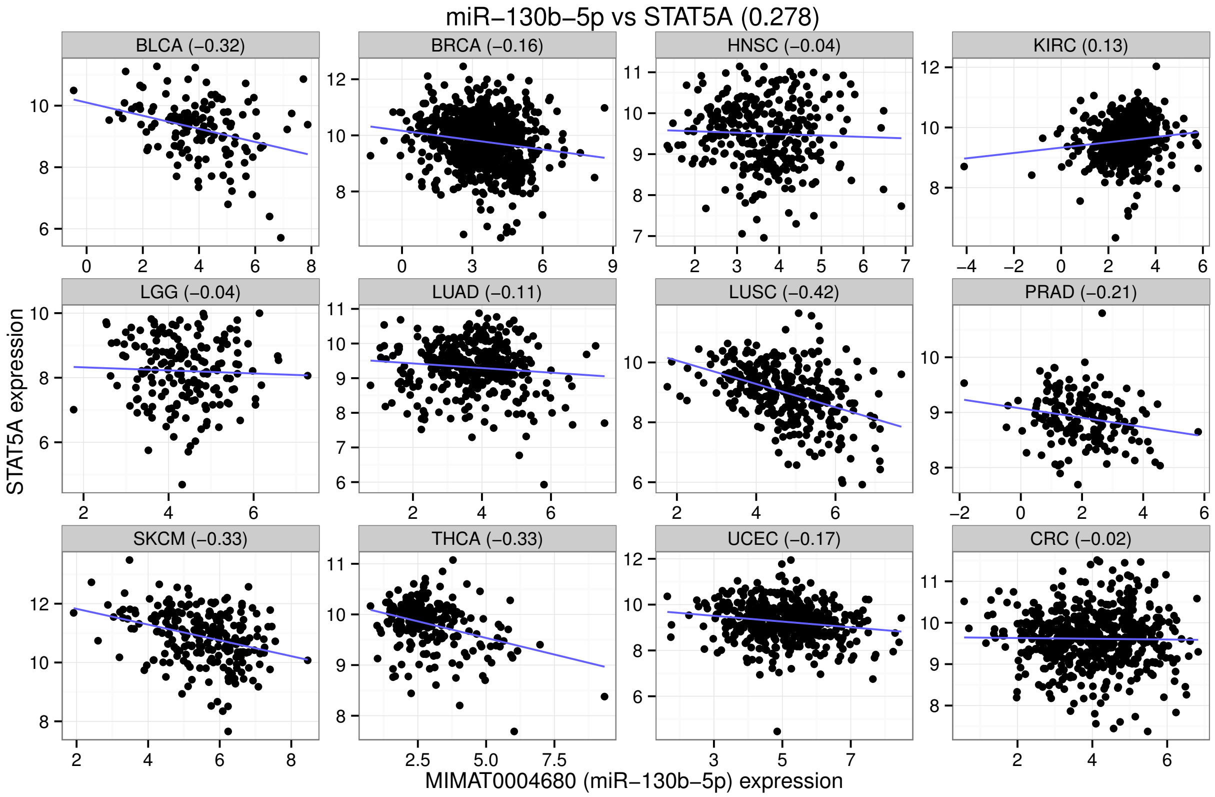
miR−130b−5p vs STAT5A (0.278)
BLCA (−0.32)
BRCA (−0.16)
HNSC (−0.04)
KIRC (0.13)
LGG (−0.04)
LUAD (−0.11)
LUSC (−0.42)
PRAD (−0.21)
SKCM (−0.33)
THCA (−0.33)
UCEC (−0.17)
CRC (−0.02)
STAT5A expression
MIMAT0004680 (miR−130b−5p) expression

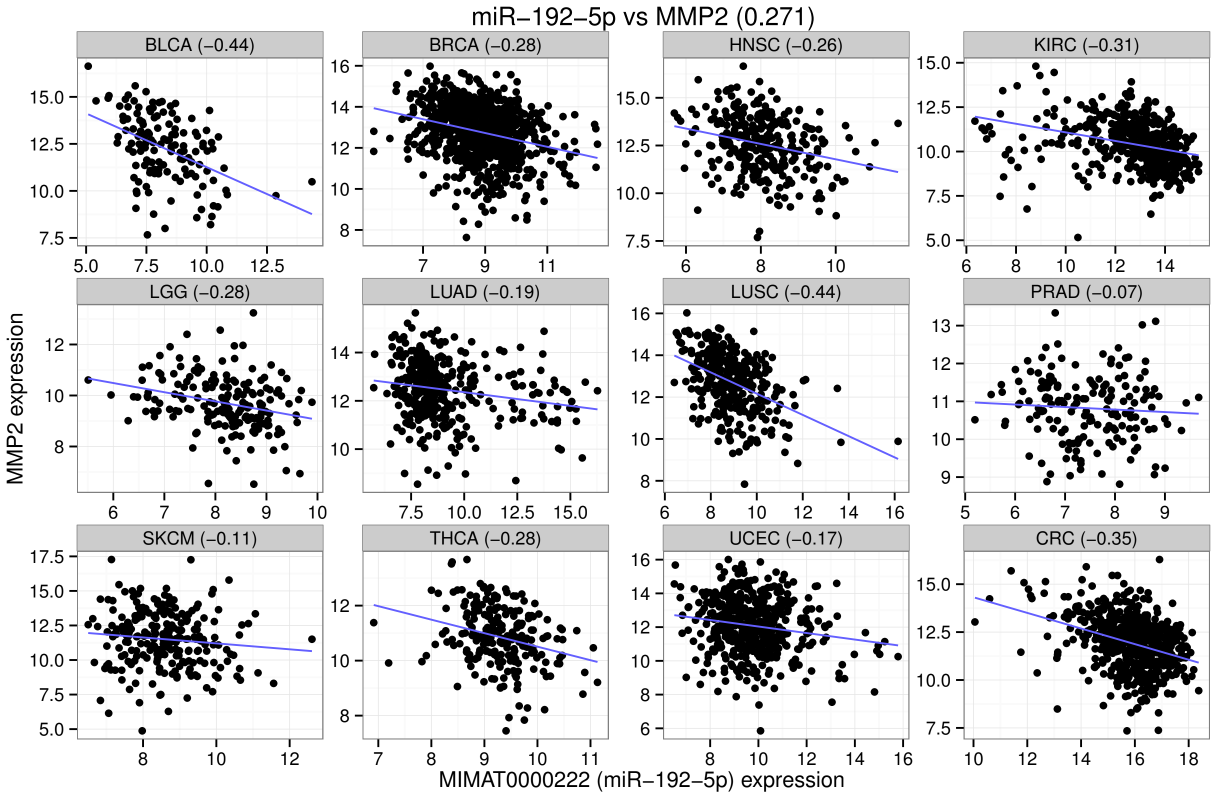
miR−192−5p vs MMP2 (0.271)
BLCA (−0.44)
BRCA (−0.28)
HNSC (−0.26)
KIRC (−0.31)
LGG (−0.28)
LUAD (−0.19)
LUSC (−0.44)
PRAD (−0.07)
SKCM (−0.11)
THCA (−0.28)
UCEC (−0.17)
CRC (−0.35)
MMP2 expression
MIMAT0000222 (miR−192−5p) expression

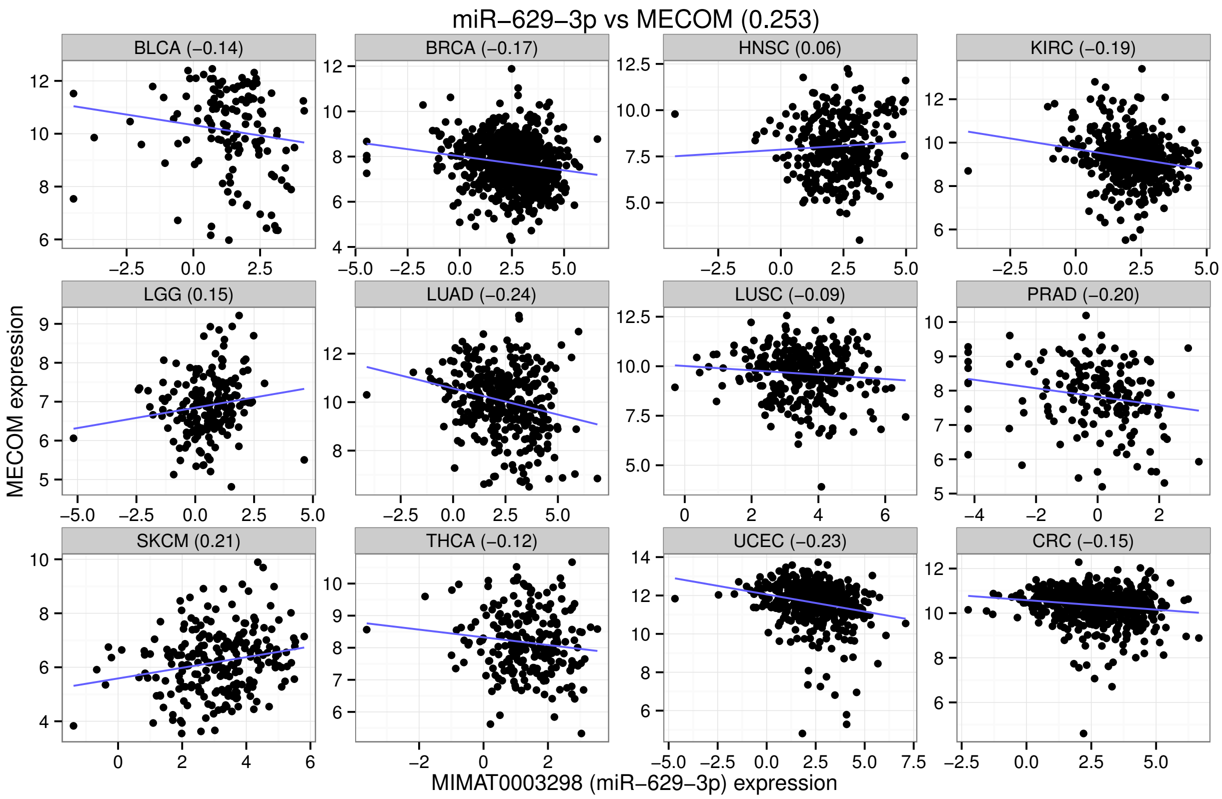
miR−629−3p vs MECOM (0.253)
BLCA (−0.14)
BRCA (−0.17)
HNSC (0.06)
KIRC (−0.19)
LGG (0.15)
LUAD (−0.24)
LUSC (−0.09)
PRAD (−0.20)
SKCM (0.21)
THCA (−0.12)
UCEC (−0.23)
CRC (−0.15)
MECOM expression
MIMAT0003298 (miR−629−3p) expression

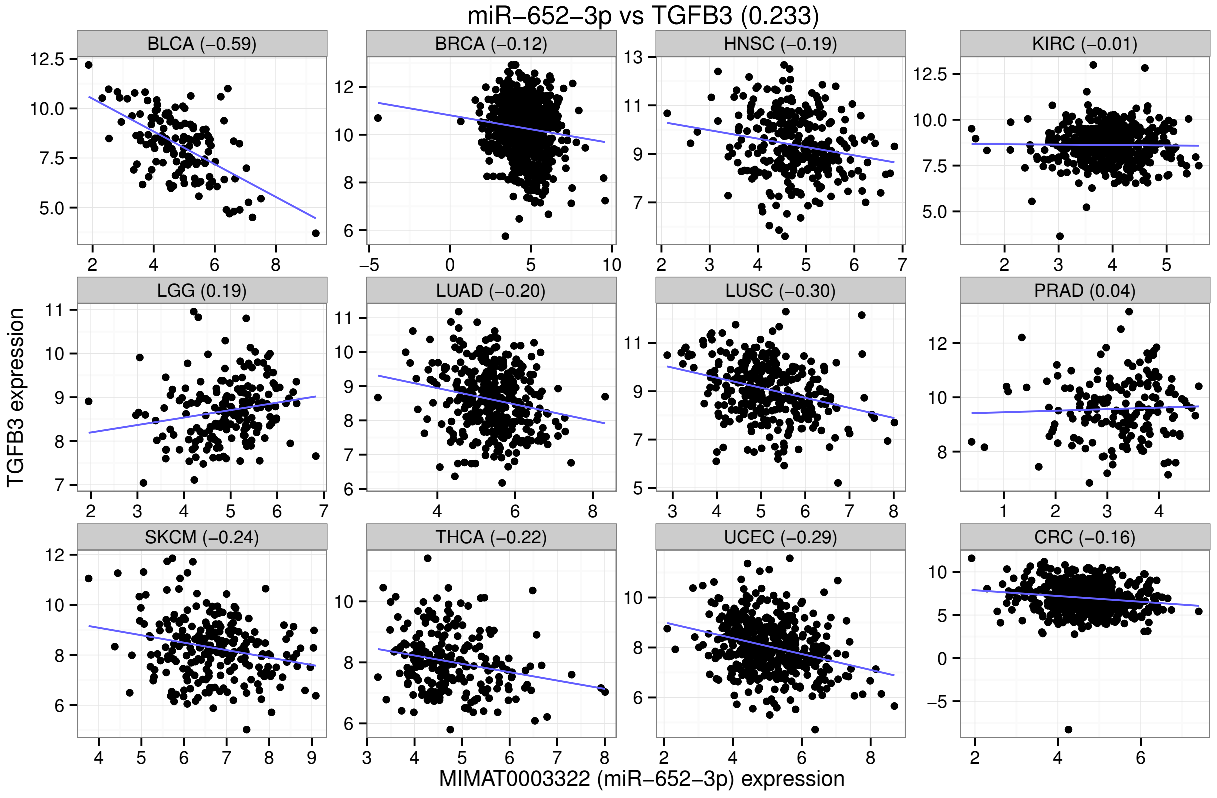
miR−652−3p vs TGFB3 (0.233)
BLCA (−0.59)
BRCA (−0.12)
HNSC (−0.19)
KIRC (−0.01)
LGG (0.19)
LUAD (−0.20)
LUSC (−0.30)
PRAD (0.04)
SKCM (−0.24)
THCA (−0.22)
UCEC (−0.29)
CRC (−0.16)
TGFB3 expression
MIMAT0003322 (miR−652−3p) expression

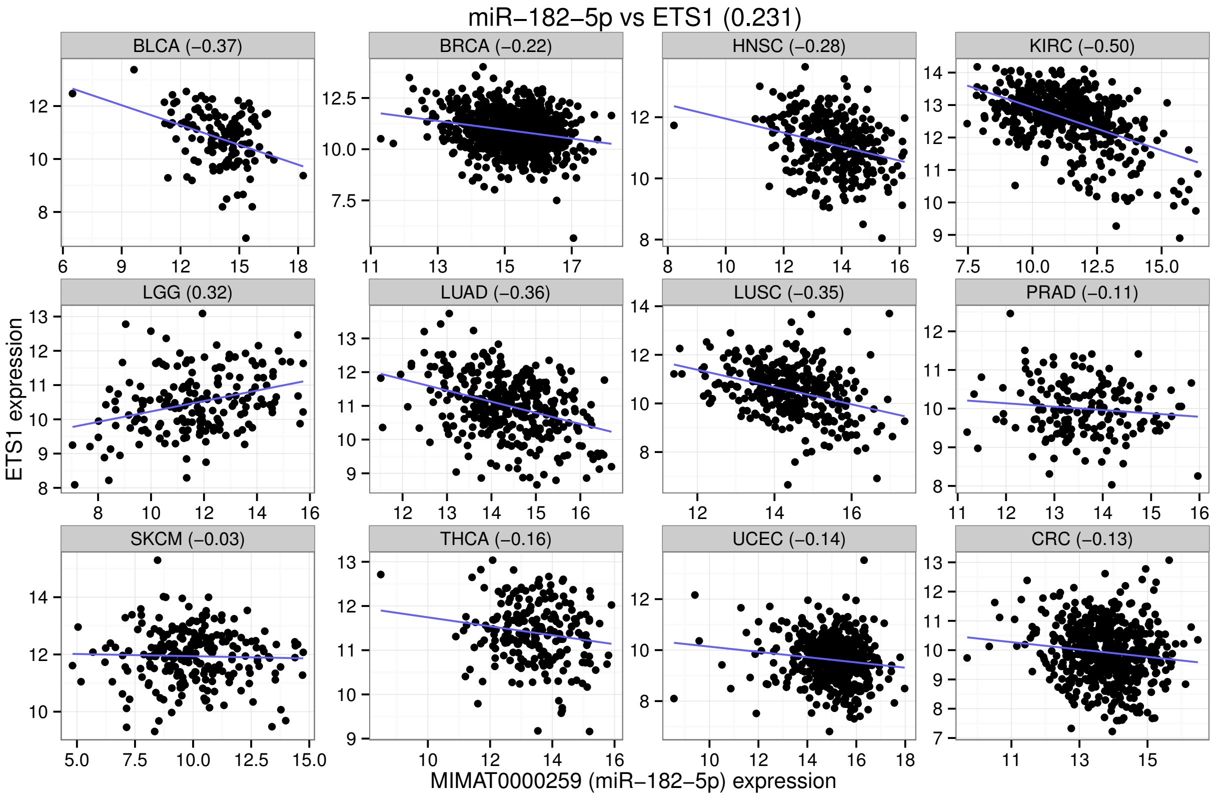

miR−182−5p vs ETS1 (0.231)
BLCA (−0.37)
BRCA (−0.22)
HNSC (−0.28)
KIRC (−0.50)
LGG (0.32)
LUAD (−0.36)
LUSC (−0.35)
PRAD (−0.11)
SKCM (−0.03)
THCA (−0.16)
UCEC (−0.14)
CRC (−0.13)
ETS1 expression
MIMAT0000259 (miR−182−5p) expression

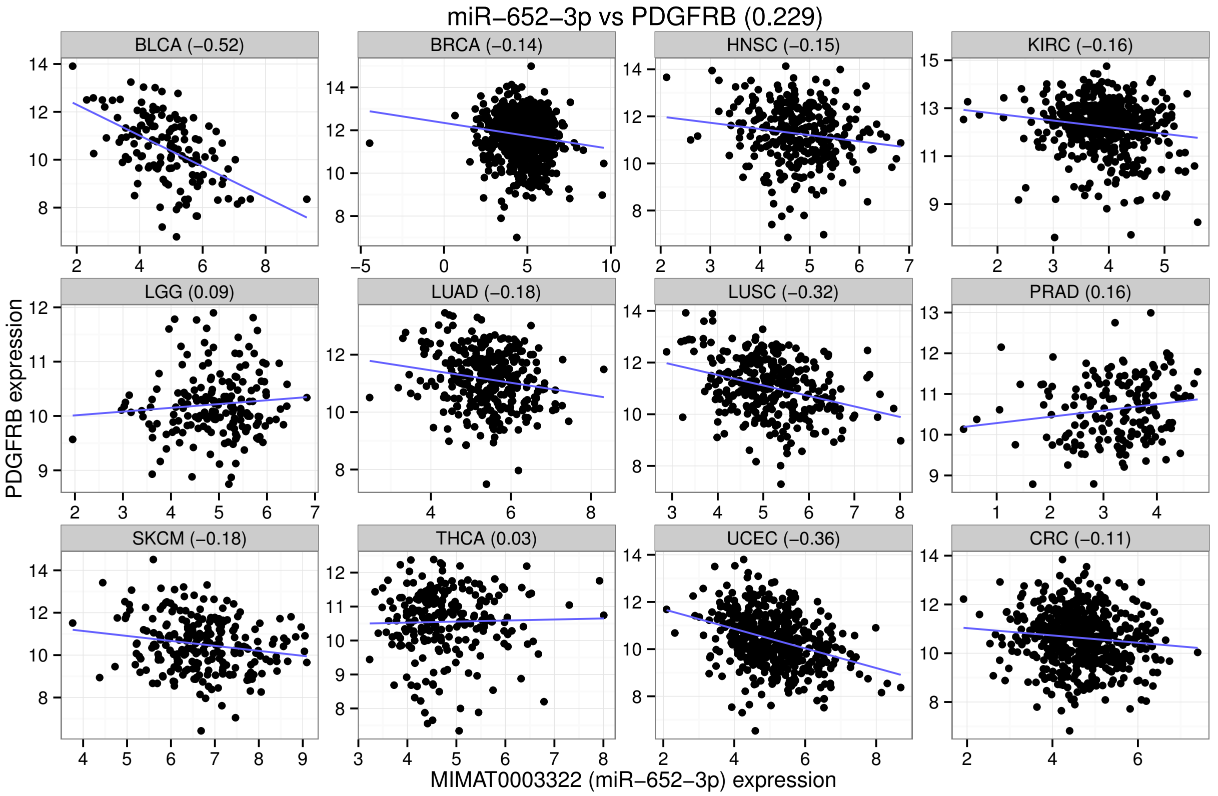

miR−652−3p vs PDGFRB (0.229)
BLCA (−0.52)
BRCA (−0.14)
HNSC (−0.15)
KIRC (−0.16)
LGG (0.09)
LUAD (−0.18)
LUSC (−0.32)
PRAD (0.16)
SKCM (−0.18)
THCA (0.03)
UCEC (−0.36)
CRC (−0.11)
PDGFRB expression
MIMAT0003322 (miR−652−3p) expression

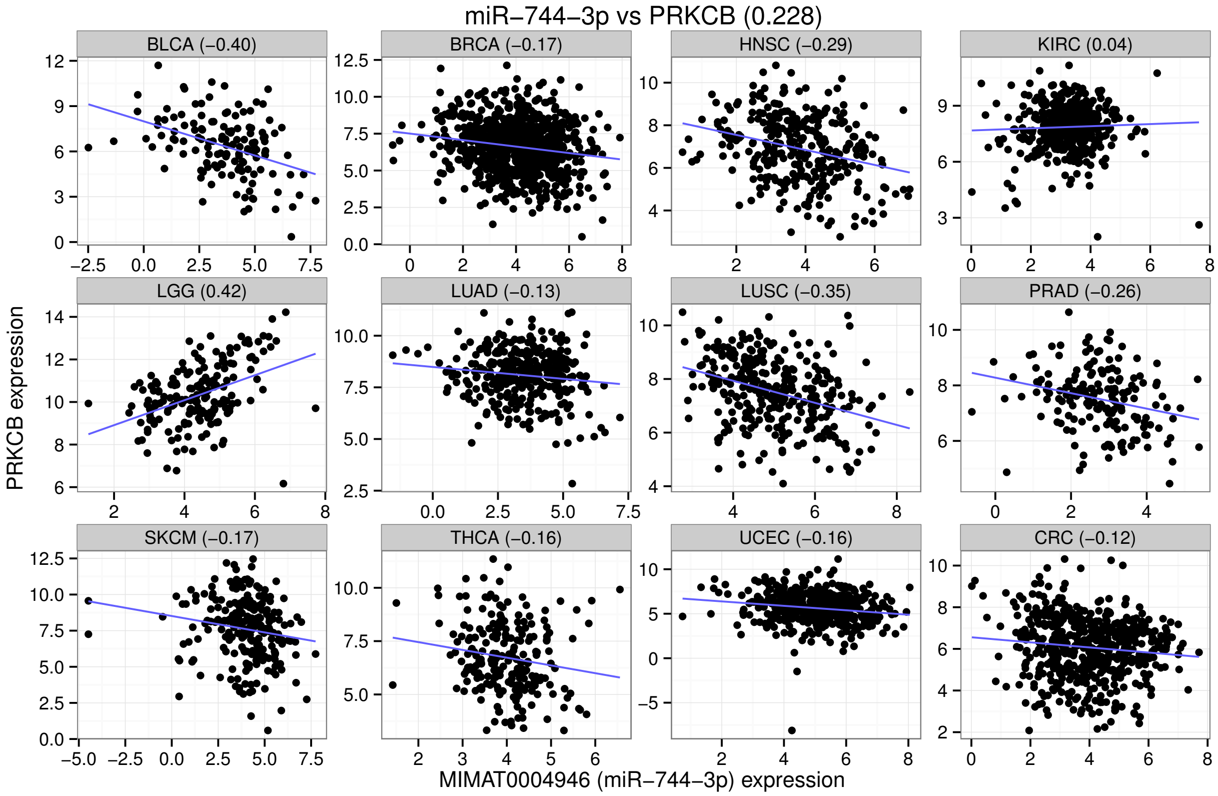
miR−744−3p vs PRKCB (0.228)
BLCA (−0.40)
BRCA (−0.17)
HNSC (−0.29)
KIRC (0.04)
LGG (0.42)
LUAD (−0.13)
LUSC (−0.35)
PRAD (−0.26)
SKCM (−0.17)
THCA (−0.16)
UCEC (−0.16)
CRC (−0.12)
PRKCB expression
MIMAT0004946 (miR−744−3p) expression

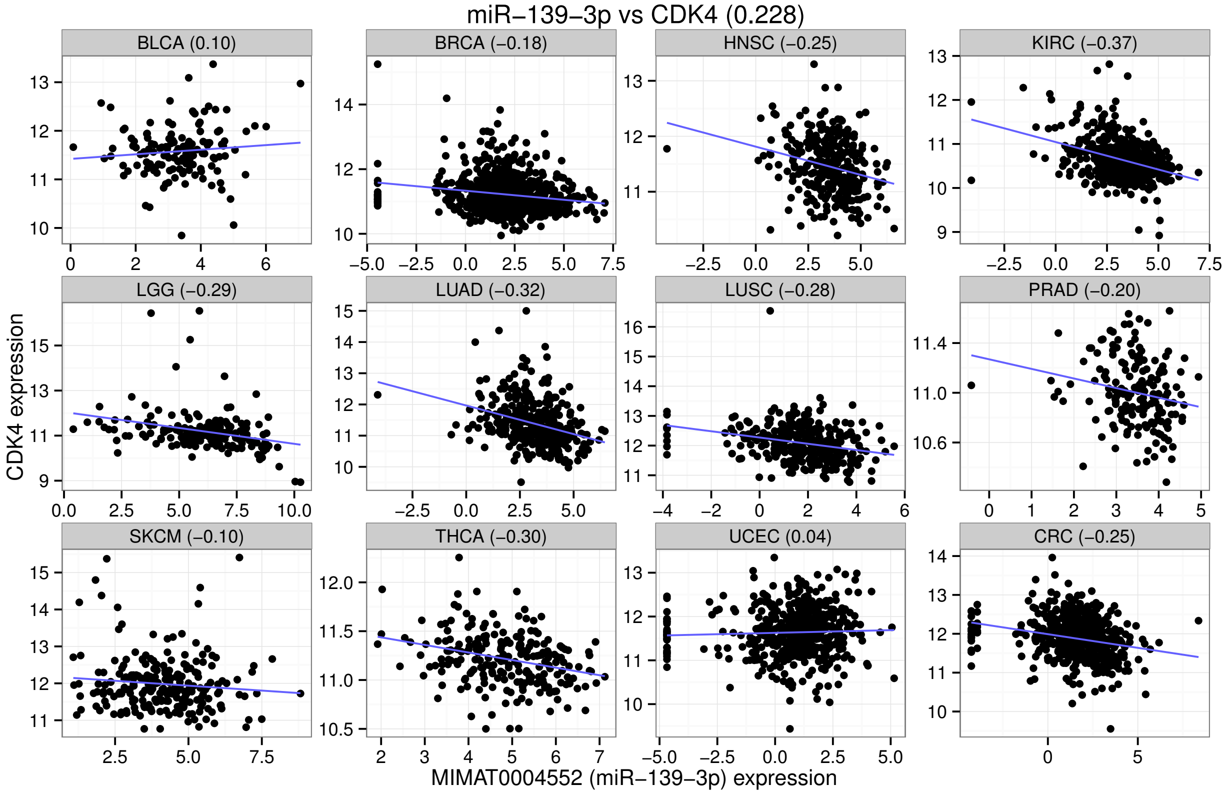
miR−139−3p vs CDK4 (0.228)
BLCA (0.10)
BRCA (−0.18)
HNSC (−0.25)
KIRC (−0.37)
LGG (−0.29)
LUAD (−0.32)
LUSC (−0.28)
PRAD (−0.20)
SKCM (−0.10)
THCA (−0.30)
UCEC (0.04)
CRC (−0.25)
CDK4 expression
MIMAT0004552 (miR−139−3p) expression

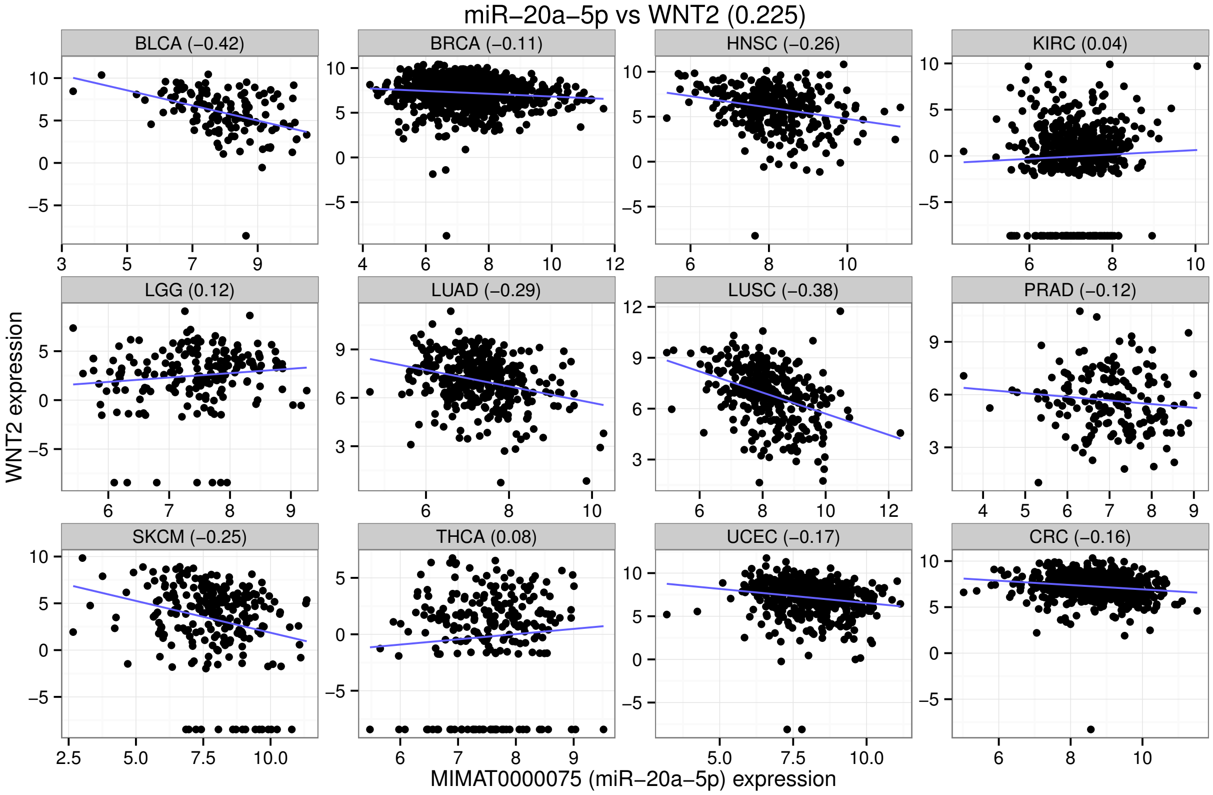
miR−20a−5p vs WNT2 (0.225)
BLCA (−0.42)
BRCA (−0.11)
HNSC (−0.26)
KIRC (0.04)
LGG (0.12)
LUAD (−0.29)
LUSC (−0.38)
PRAD (−0.12)
SKCM (−0.25)
THCA (0.08)
UCEC (−0.17)
CRC (−0.16)
WNT2 expression
MIMAT0000075 (miR−20a−5p) expression

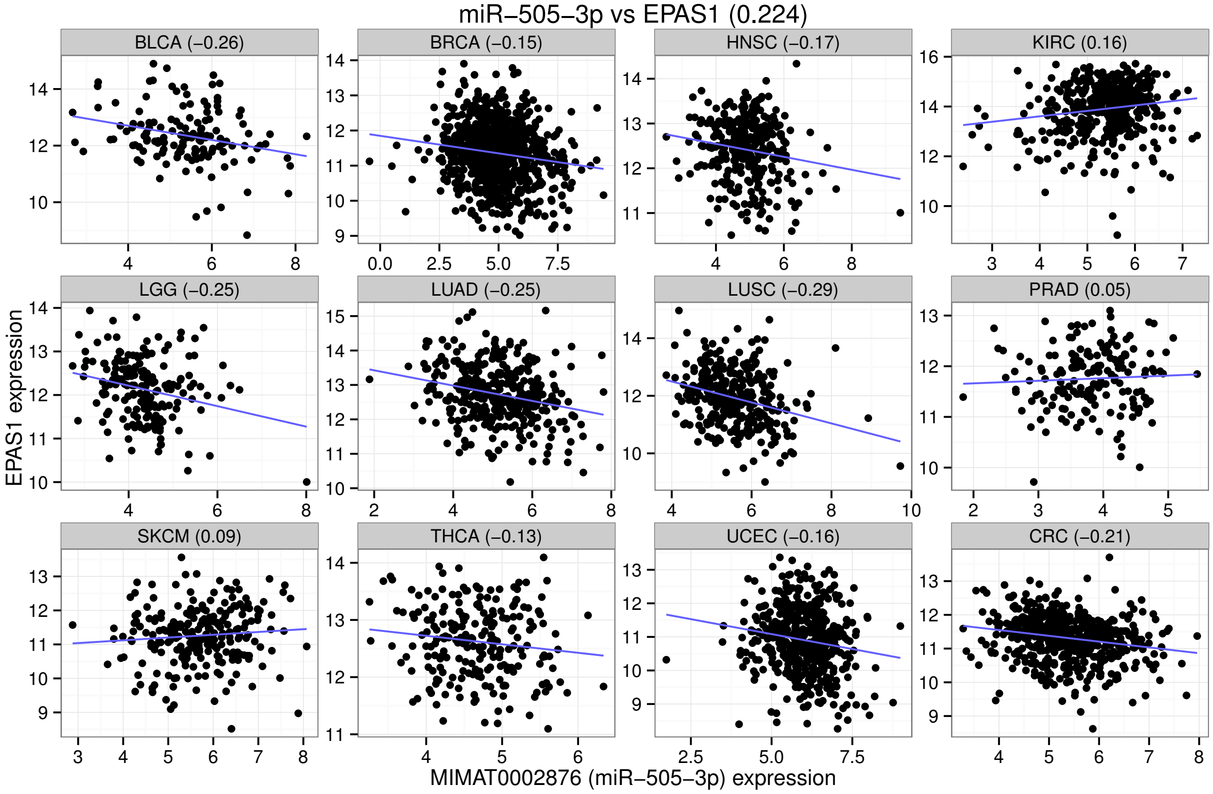
miR−505−3p vs EPAS1 (0.224)
BLCA (−0.26)
BRCA (−0.15)
HNSC (−0.17)
KIRC (0.16)
LGG (−0.25)
LUAD (−0.25)
LUSC (−0.29)
PRAD (0.05)
SKCM (0.09)
THCA (−0.13)
UCEC (−0.16)
CRC (−0.21)
EPAS1 expression
MIMAT0002876 (miR−505−3p) expression

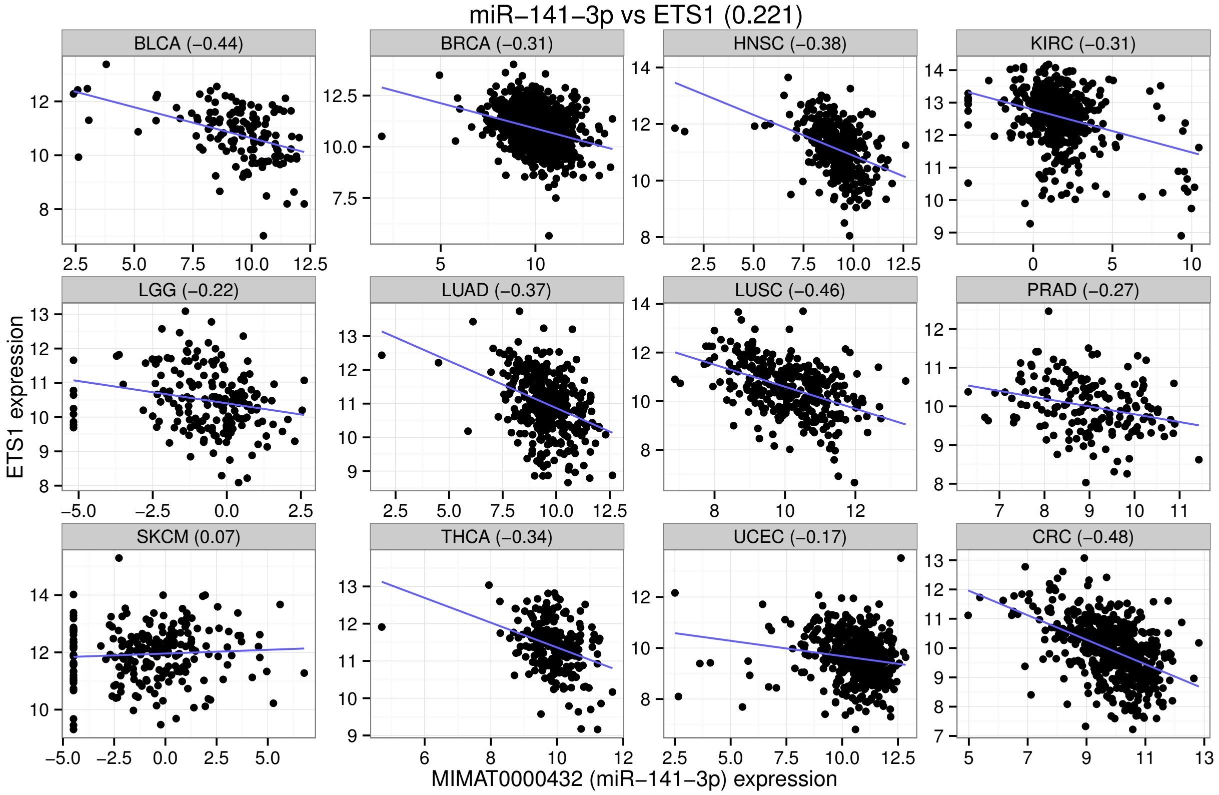
miR−141−3p vs ETS1 (0.221)
BLCA (−0.44)
BRCA (−0.31)
HNSC (−0.38)
KIRC (−0.31)
LGG (−0.22)
LUAD (−0.37)
LUSC (−0.46)
PRAD (−0.27)
SKCM (0.07)
THCA (−0.34)
UCEC (−0.17)
CRC (−0.48)
ETS1 expression
MIMAT0000432 (miR−141−3p) expression

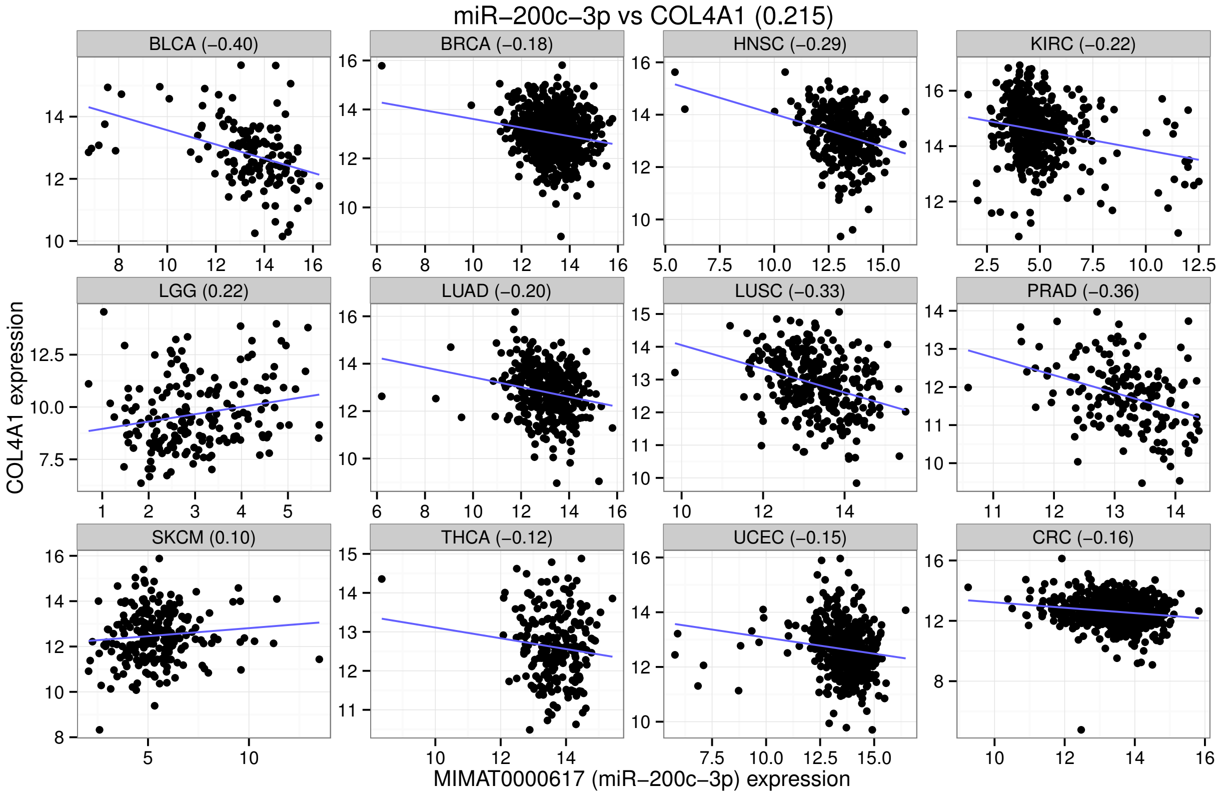
miR−200c−3p vs COL4A1 (0.215)
BLCA (−0.40)
BRCA (−0.18)
HNSC (−0.29)
KIRC (−0.22)
LGG (0.22)
LUAD (−0.20)
LUSC (−0.33)
PRAD (−0.36)
SKCM (0.10)
THCA (−0.12)
UCEC (−0.15)
CRC (−0.16)
COL4A1 expression
MIMAT0000617 (miR−200c−3p) expression

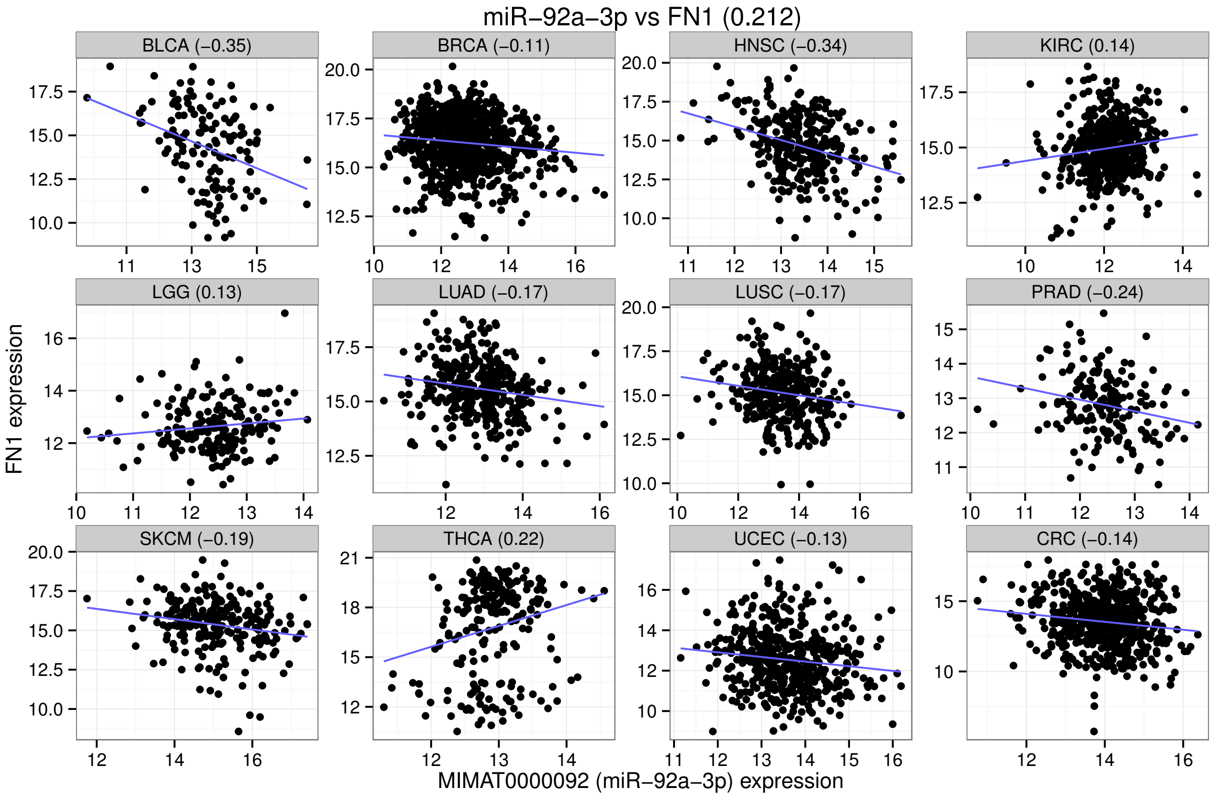
miR−92a−3p vs FN1 (0.212)
BLCA (−0.35)
BRCA (−0.11)
HNSC (−0.34)
KIRC (0.14)
LGG (0.13)
LUAD (−0.17)
LUSC (−0.17)
PRAD (−0.24)
SKCM (−0.19)
THCA (0.22)
UCEC (−0.13)
CRC (−0.14)
FN1 expression
MIMAT0000092 (miR−92a−3p) expression

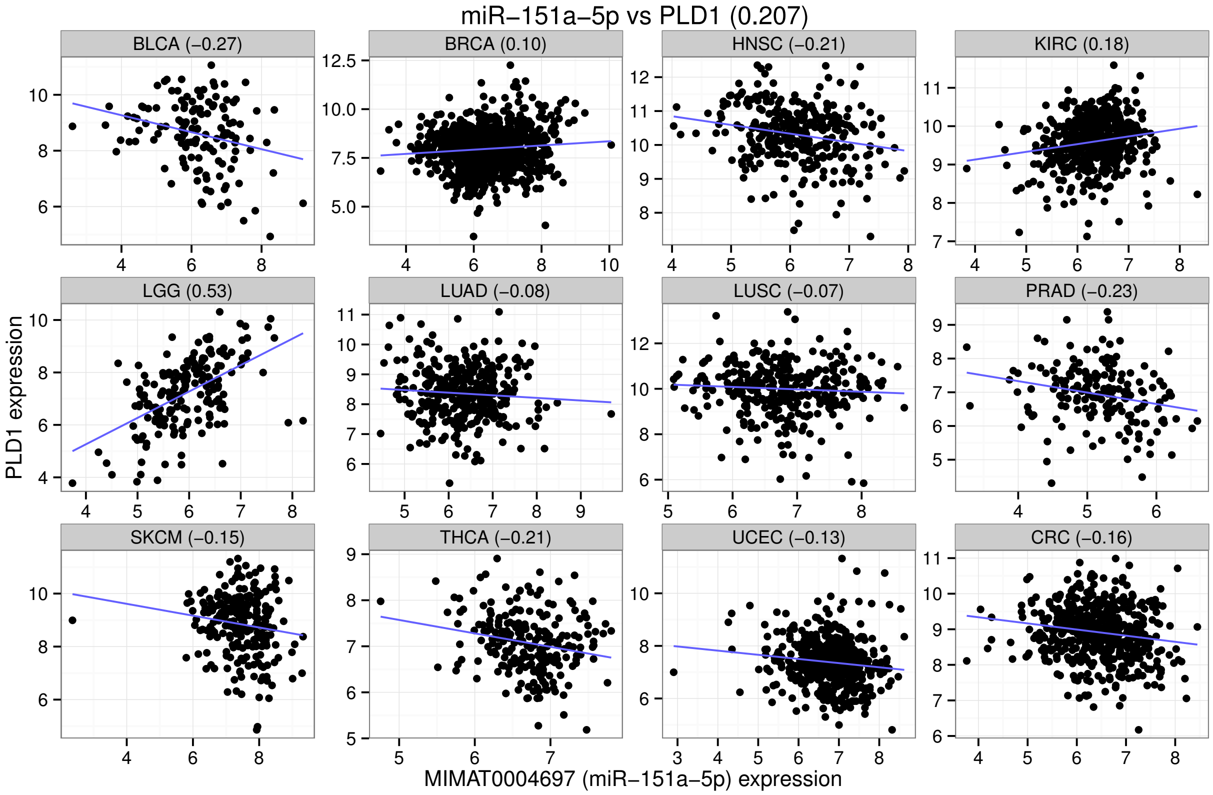
miR−151a−5p vs PLD1 (0.207)
BLCA (−0.27)
BRCA (0.10)
HNSC (−0.21)
KIRC (0.18)
LGG (0.53)
LUAD (−0.08)
LUSC (−0.07)
PRAD (−0.23)
SKCM (−0.15)
THCA (−0.21)
UCEC (−0.13)
CRC (−0.16)
PLD1 expression
MIMAT0004697 (miR−151a−5p) expression

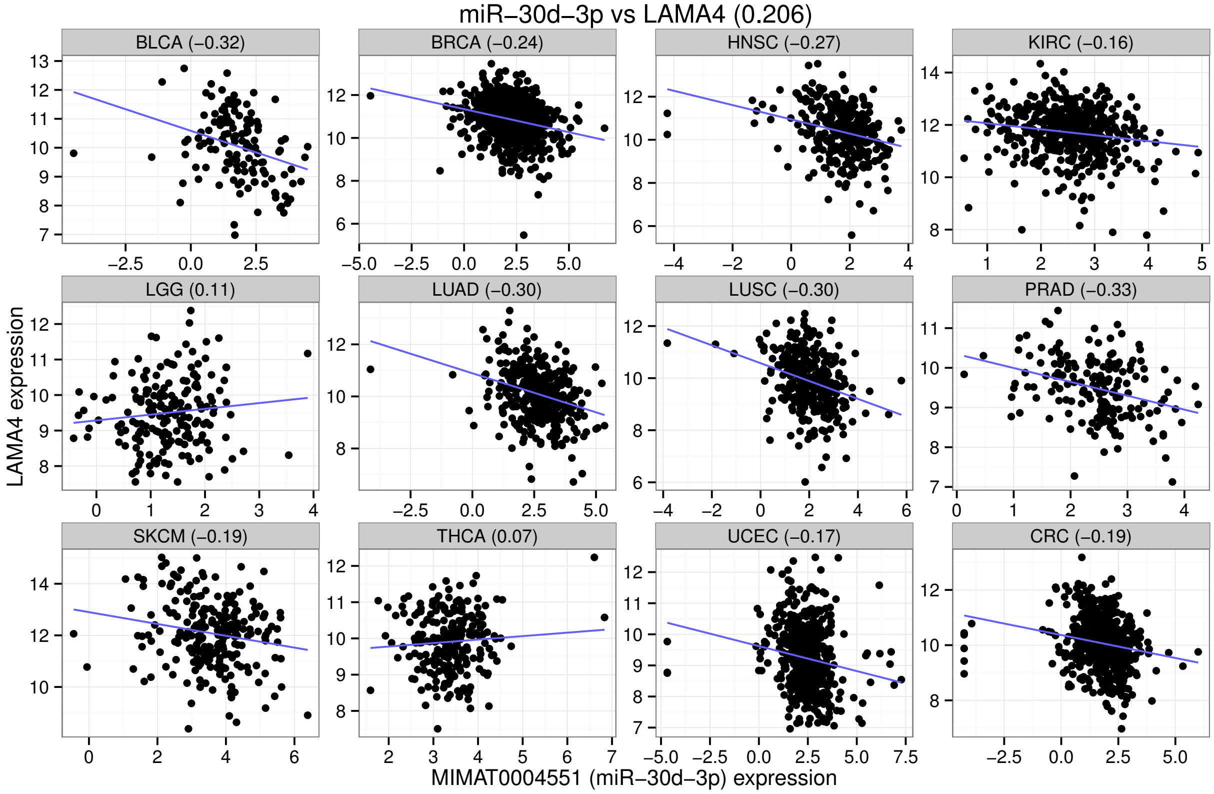

Figure S3: **Scatter plot of the confidence miRNA-target pairs involved in the KEGG pathways in cancer.** Each of the 12 panels represents a specific cancer type as indicated in the strip header. In each panel, each dot represents a sample with the coordinate determined by the corresponding miRNA (x-axis) and target expression (y-axis).

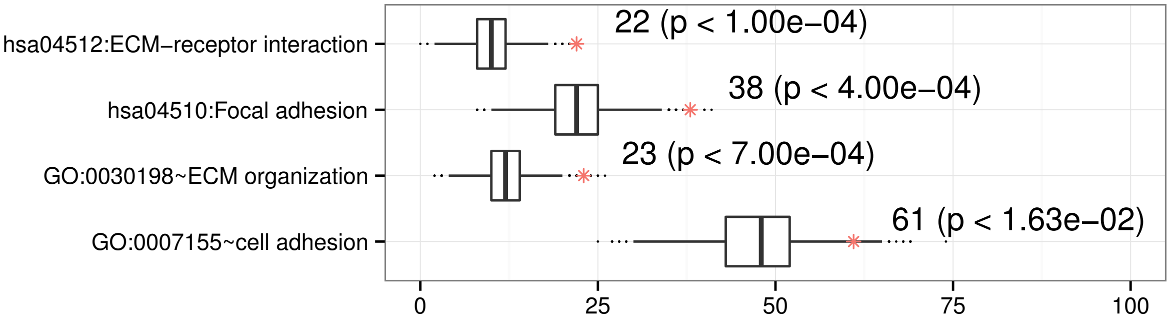

Figure S4: **Empirical distribution of random gene counts involved in the four select GO-BP or KEGG pathways.** We randomly sampled 1480 interactions from the ∼1.8 million interactions and counted the number genes involved in each of the four GO terms or KEGG pathways related to ECM receptor interaction/organization or cell/focal adhesion. The distribution for each gene set were displayed as boxplot, and the red asterisk indicate the location of the observed gene counts. P-values were calculated by the fraction of gene count in a million samplings that have gene count greater than the corresponding observed values: $p = 10^{-6} \sum_{i=1}^{10^6} I(N_i > N_o)$, where N_i and N_o are the number of gene hits in the randomly sampled and the observed gene hits in the recurrent interactions, respectively; $I(.)$ is a binary indicator that gives 1 for a true statement and 0 otherwise.

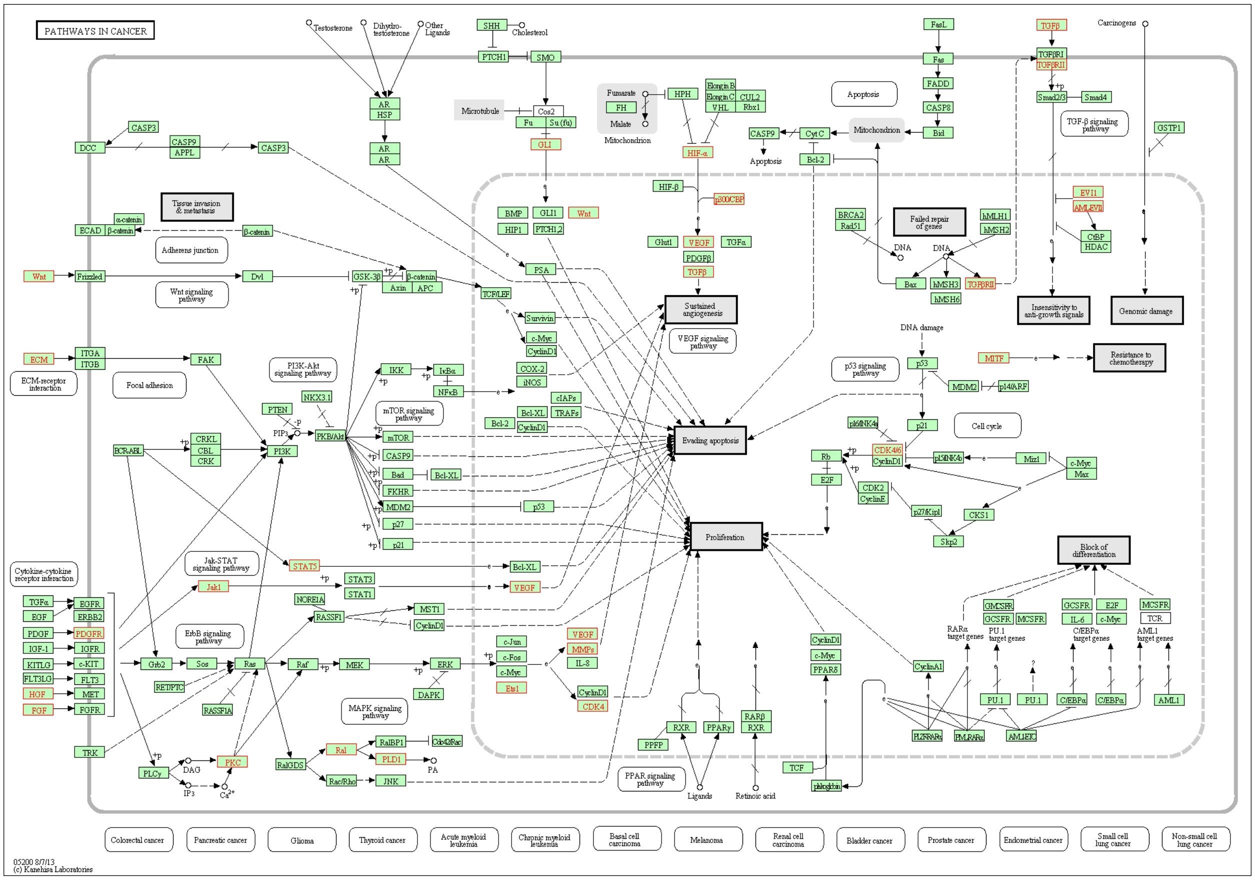

Figure S5: **The original screenshot of the KEGG ECM-receptor interactions**. The highlighted boxes indicating the targets involved in the recurrent miRNA regulatory network.

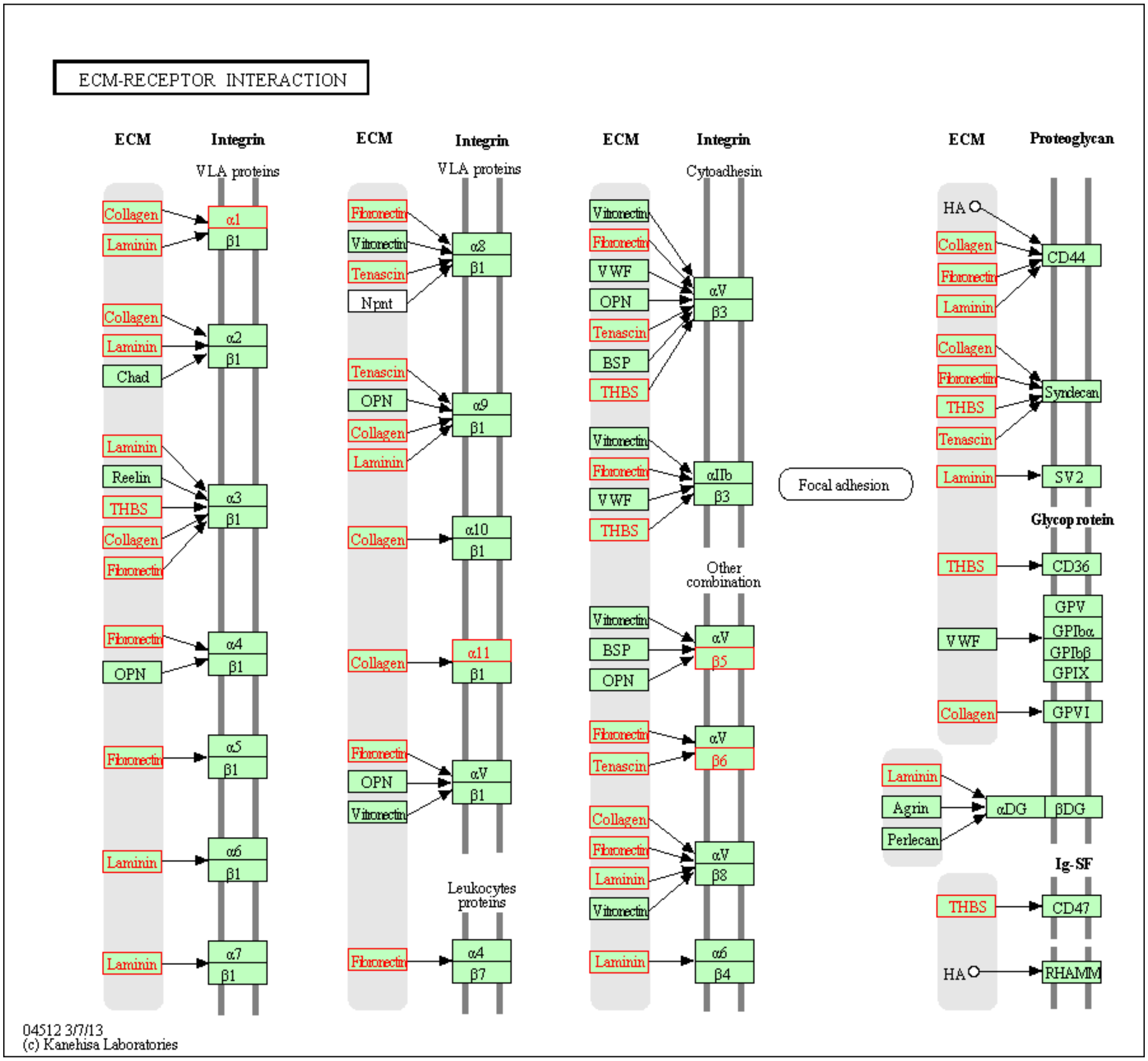

Figure S6: **The original screenshot of the KEGG pathways in cancer**. The highlighted boxes indicating the targets involved in the recurrent miRNA regulatory network.

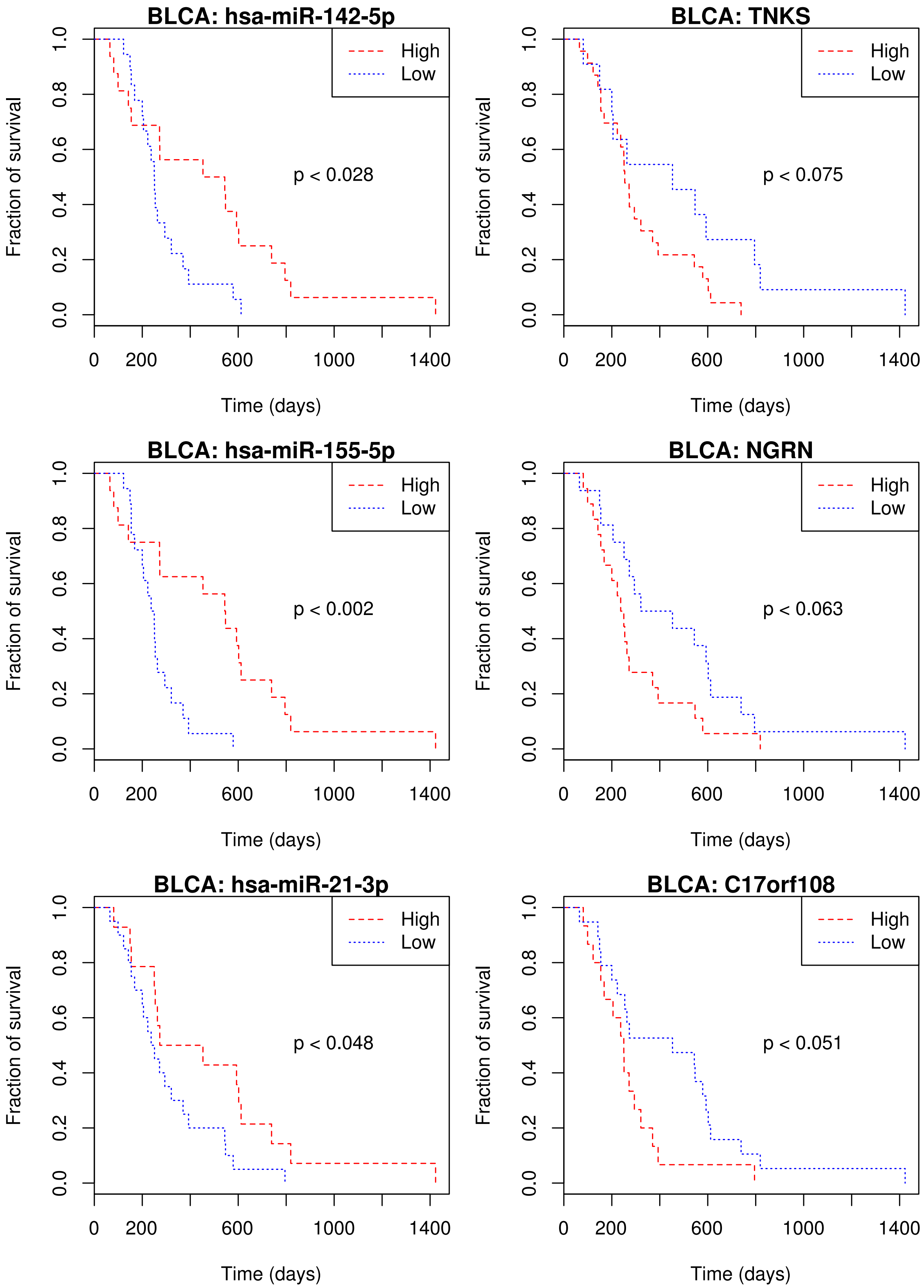
BLCA: hsa-miR-142-5p
High
Low
p < 0.028
Fraction of survival
Time (days)
BLCA: TNKS
High
Low
p < 0.075
Fraction of survival
Time (days)
BLCA: hsa-miR-155-5p
High
Low
p < 0.002
Fraction of survival
Time (days)
BLCA: NGRN
High
Low
p < 0.063
Fraction of survival
Time (days)
BLCA: hsa-miR-21-3p
High
Low
p < 0.048
Fraction of survival
Time (days)
BLCA: C17orf108
High
Low
p < 0.051
Fraction of survival
Time (days)

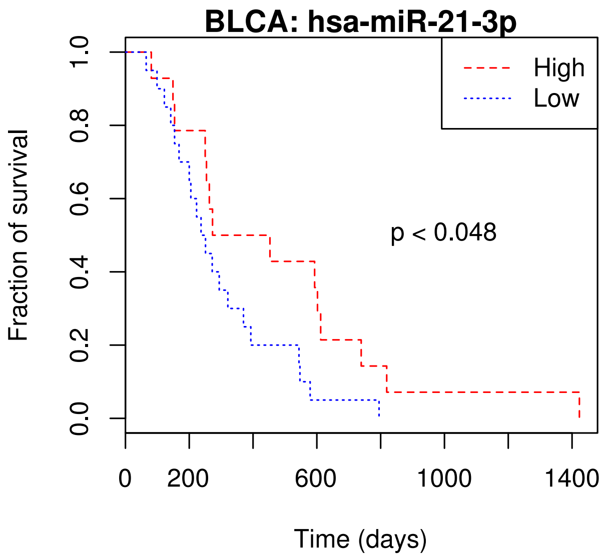
BLCA: hsa-miR-21-3p
High
Low
p < 0.048
Fraction of survival
Time (days)
0.0 0.2 0.4 0.6 0.8 1.0
0 200 600 1000 1400

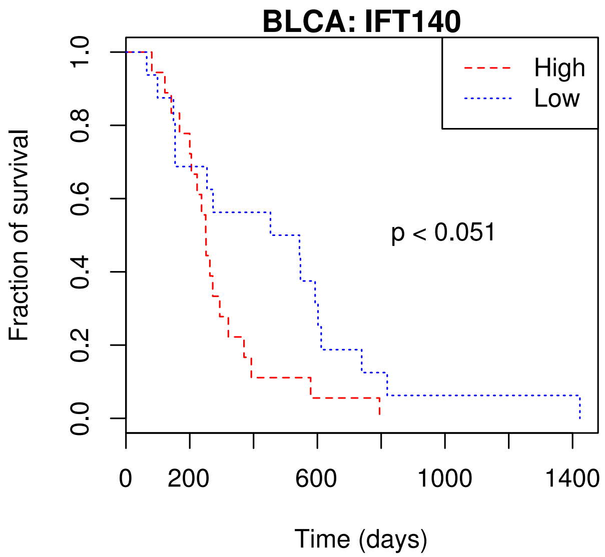
BLCA: IFT140
High
Low
p < 0.051
Fraction of survival
Time (days)
0.0 0.2 0.4 0.6 0.8 1.0
0 200 600 1000 1400

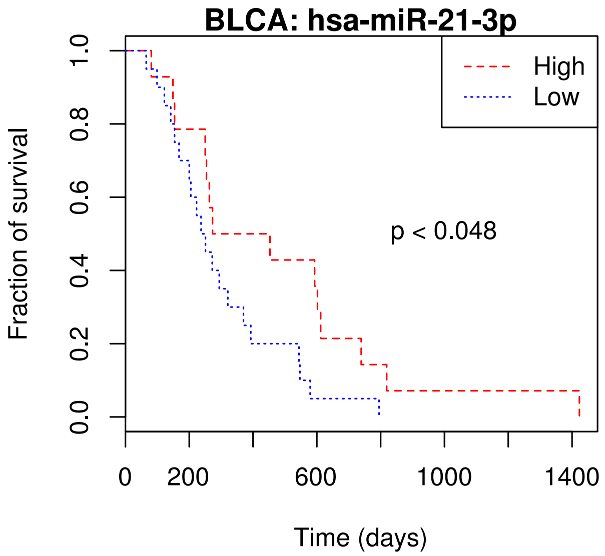
BLCA: hsa-miR-21-3p
High
Low
p < 0.048
Fraction of survival
Time (days)
0.0 0.2 0.4 0.6 0.8 1.0
0 200 600 1000 1400

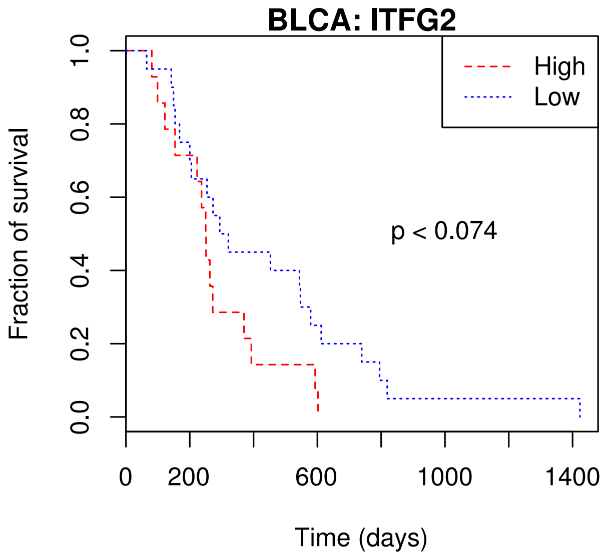
BLCA: ITFG2
High
Low
p < 0.074
Fraction of survival
Time (days)
0.0 0.2 0.4 0.6 0.8 1.0
0 200 600 1000 1400

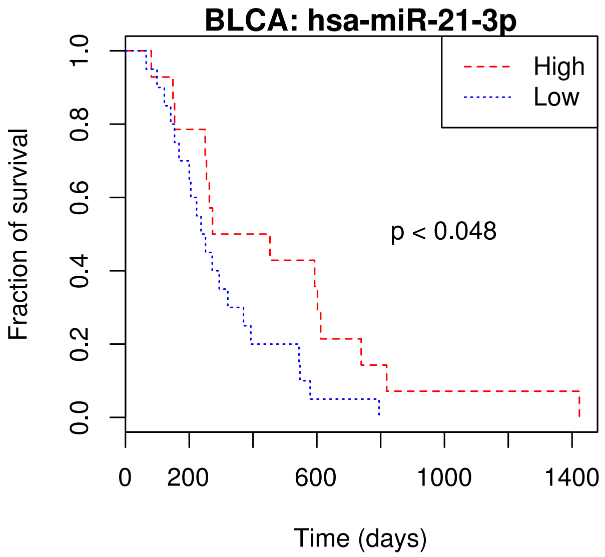
BLCA: hsa-miR-21-3p
High
Low
p < 0.048
Fraction of survival
Time (days)
0.0 0.2 0.4 0.6 0.8 1.0
0 200 600 1000 1400

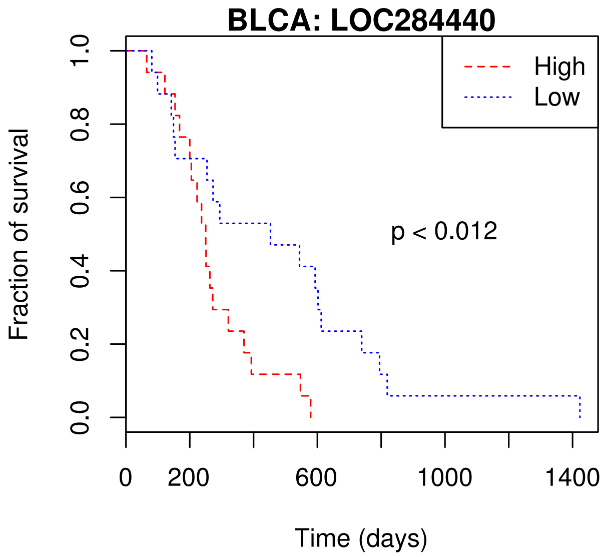
BLCA: LOC284440
High
Low
p < 0.012
Fraction of survival
Time (days)
0.0 0.2 0.4 0.6 0.8 1.0
0 200 600 1000 1400

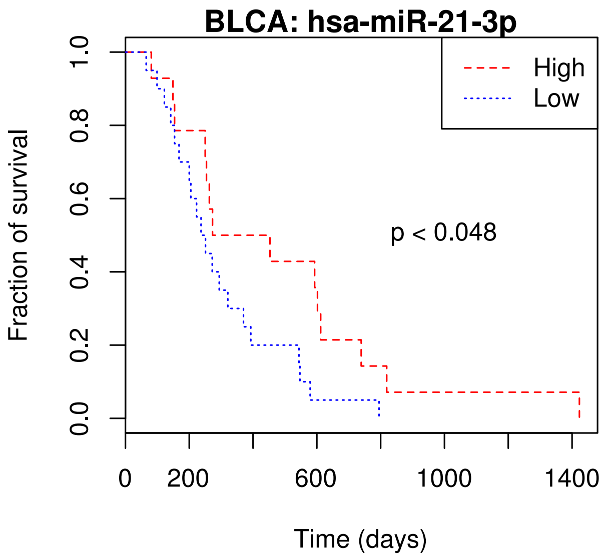
BLCA: hsa-miR-21-3p
High
Low
p < 0.048
Fraction of survival
0.0
0.2
0.4
0.6
0.8
1.0
0
200
600
1000
1400
Time (days)

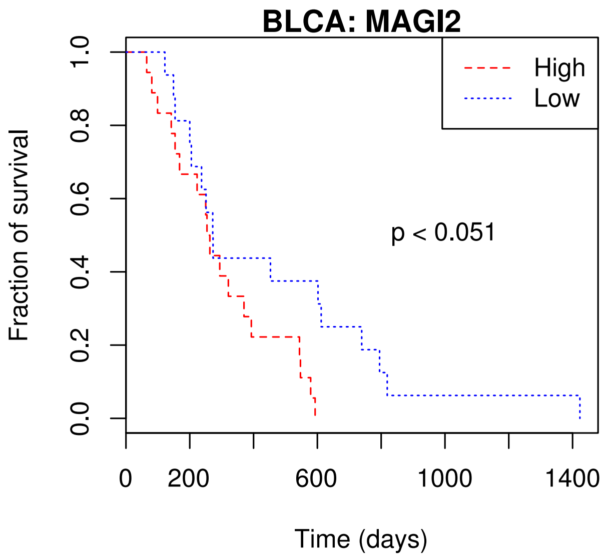
BLCA: MAGI2
High
Low
p < 0.051
Fraction of survival
0.0
0.2
0.4
0.6
0.8
1.0
0
200
600
1000
1400
Time (days)

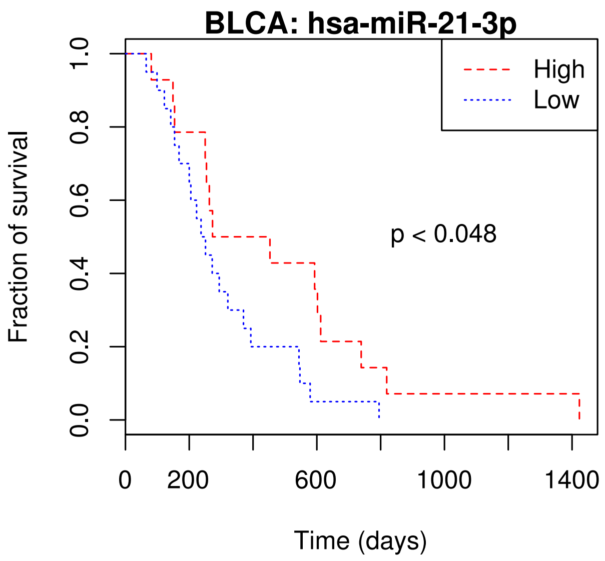
BLCA: hsa-miR-21-3p
High
Low
p < 0.048
Fraction of survival
0.0
0.2
0.4
0.6
0.8
1.0
0
200
600
1000
1400
Time (days)

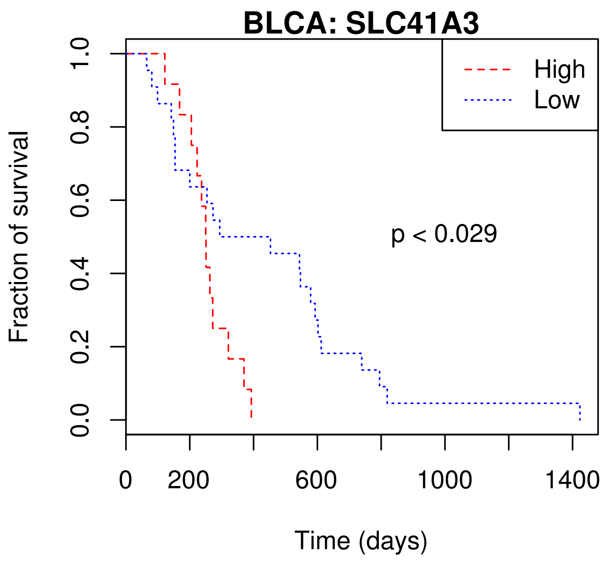
BLCA: SLC41A3
High
Low
p < 0.029
Fraction of survival
0.0
0.2
0.4
0.6
0.8
1.0
0
200
600
1000
1400
Time (days)

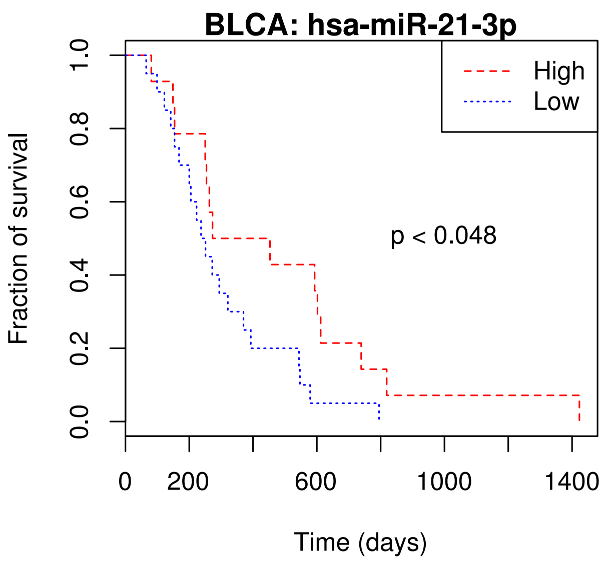
BLCA: hsa-miR-21-3p
High
Low
p < 0.048
Fraction of survival
0.0
0.2
0.4
0.6
0.8
1.0
0
200
600
1000
1400
Time (days)

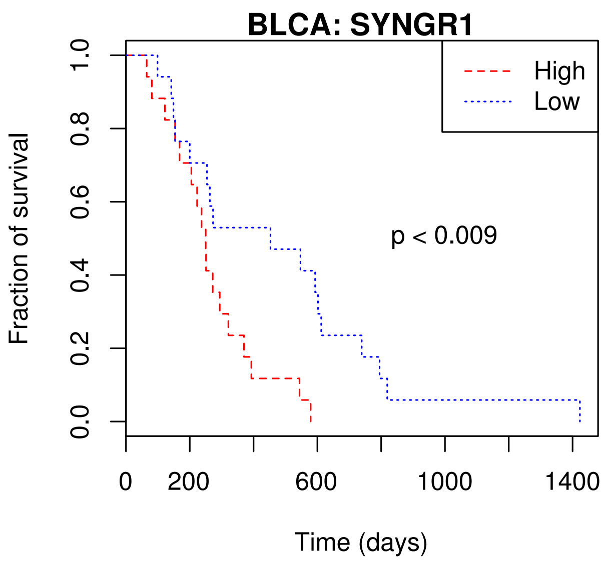
BLCA: SYNGR1
High
Low
p < 0.009
Fraction of survival
0.0
0.2
0.4
0.6
0.8
1.0
0
200
600
1000
1400
Time (days)

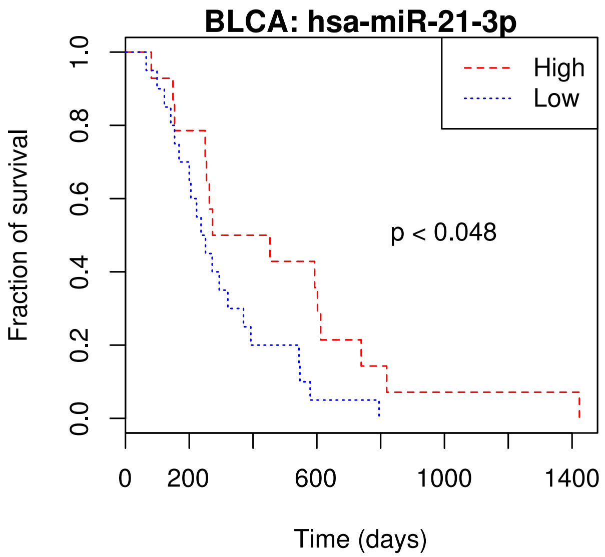
BLCA: hsa-miR-21-3p
High
Low
p < 0.048
Fraction of survival
Time (days)
0.0 0.2 0.4 0.6 0.8 1.0
0 200 600 1000 1400

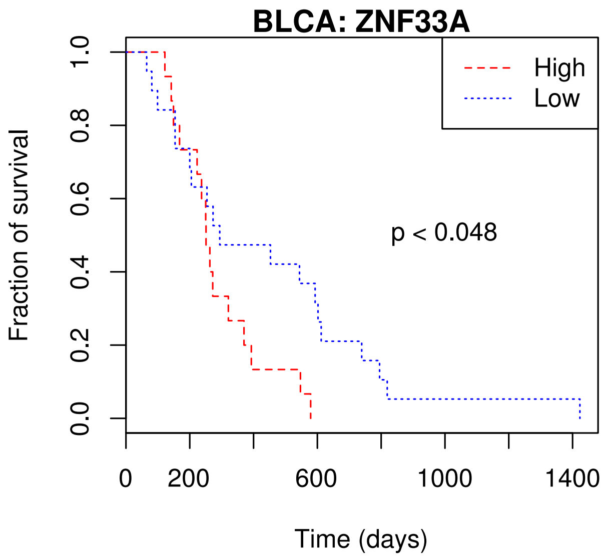
BLCA: ZNF33A
High
Low
p < 0.048
Fraction of survival
Time (days)
0.0 0.2 0.4 0.6 0.8 1.0
0 200 600 1000 1400

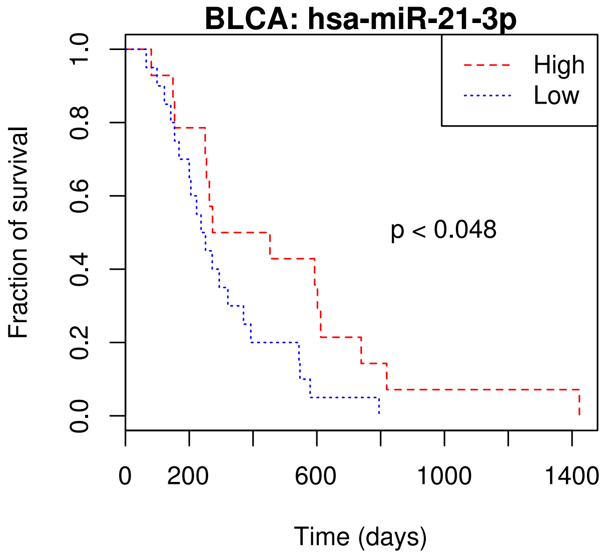
BLCA: hsa-miR-21-3p
High
Low
p < 0.048
Fraction of survival
Time (days)
0.0 0.2 0.4 0.6 0.8 1.0
0 200 600 1000 1400

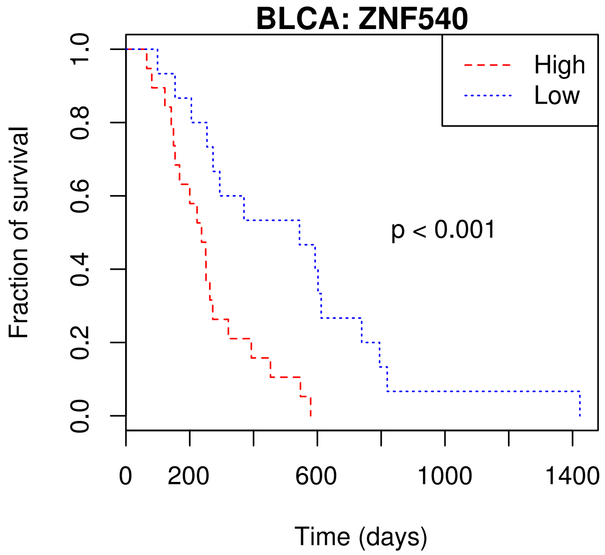
BLCA: ZNF540
High
Low
p < 0.001
Fraction of survival
Time (days)
0.0 0.2 0.4 0.6 0.8 1.0
0 200 600 1000 1400

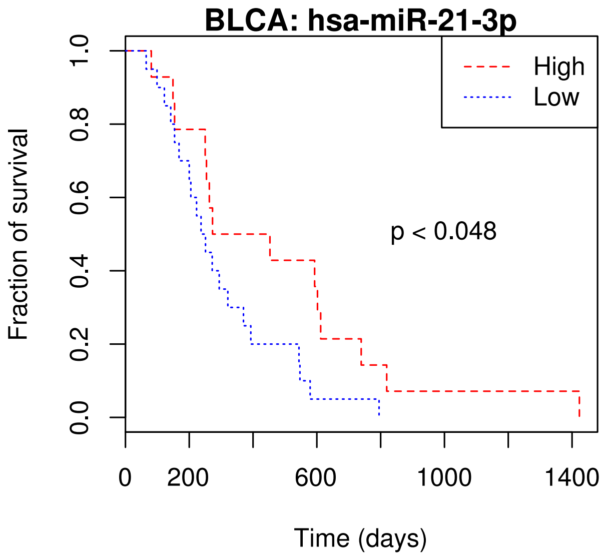
BLCA: hsa-miR-21-3p
High
Low
p < 0.048
Fraction of survival
Time (days)
0.0 0.2 0.4 0.6 0.8 1.0
0 200 600 1000 1400

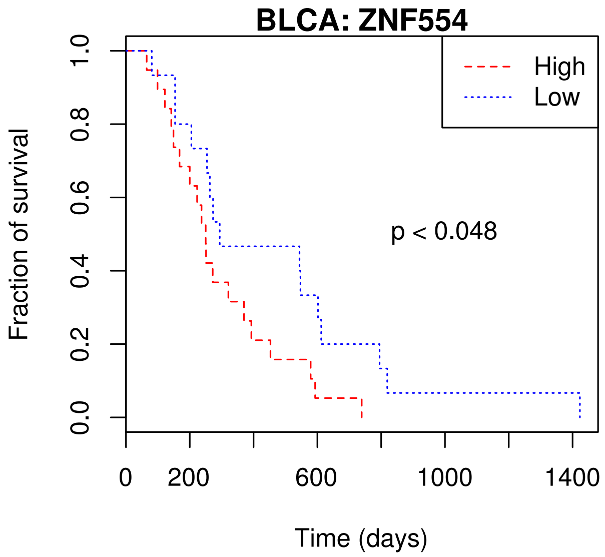
BLCA: ZNF554
High
Low
p < 0.048
Fraction of survival
Time (days)
0.0 0.2 0.4 0.6 0.8 1.0
0 200 600 1000 1400

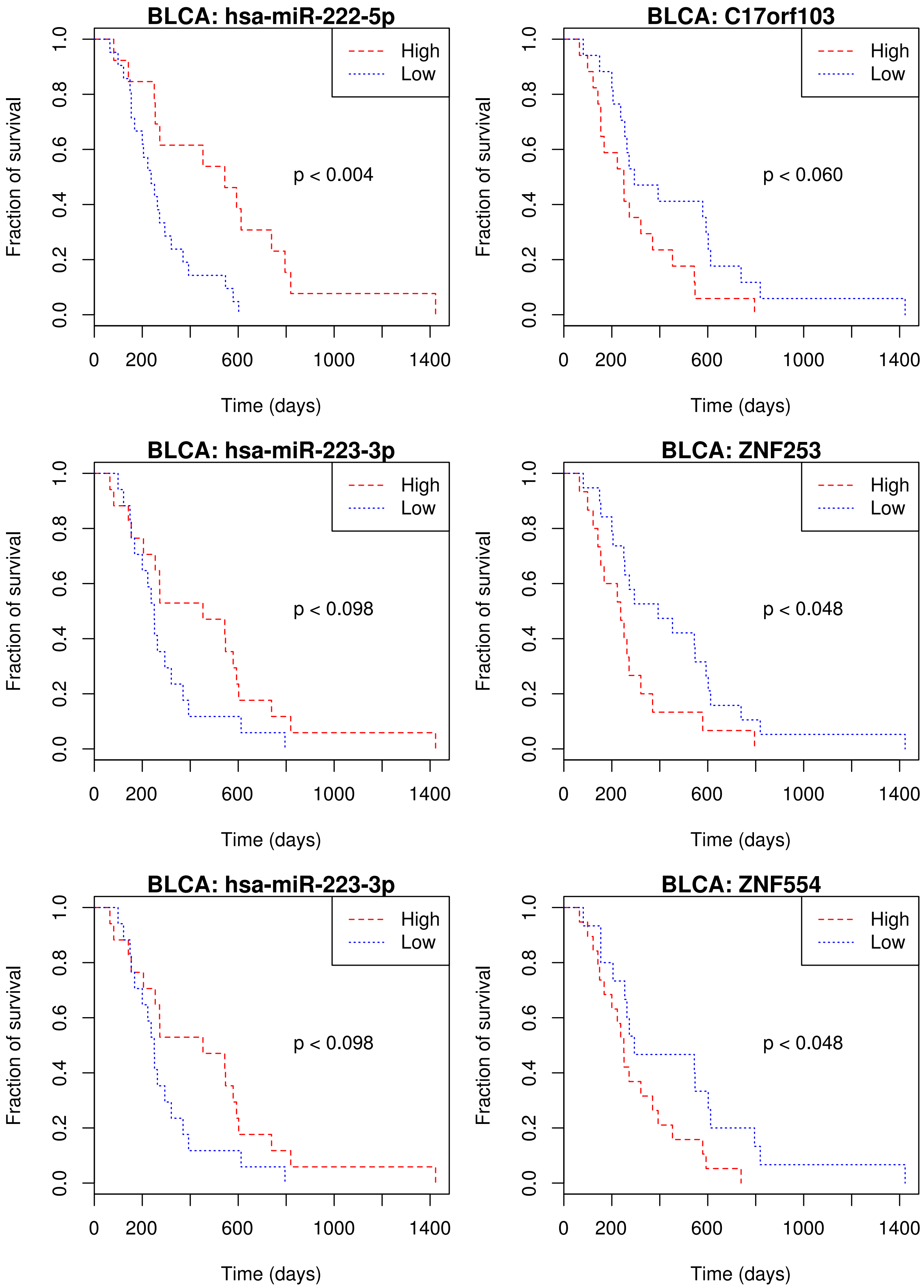
BLCA: hsa-miR-222-5p
High
Low
p < 0.004
Fraction of survival
Time (days)
BLCA: C17orf103
High
Low
p < 0.060
Fraction of survival
Time (days)
BLCA: hsa-miR-223-3p
High
Low
p < 0.098
Fraction of survival
Time (days)
BLCA: ZNF253
High
Low
p < 0.048
Fraction of survival
Time (days)
BLCA: hsa-miR-223-3p
High
Low
p < 0.098
Fraction of survival
Time (days)
BLCA: ZNF554
High
Low
p < 0.048
Fraction of survival
Time (days)

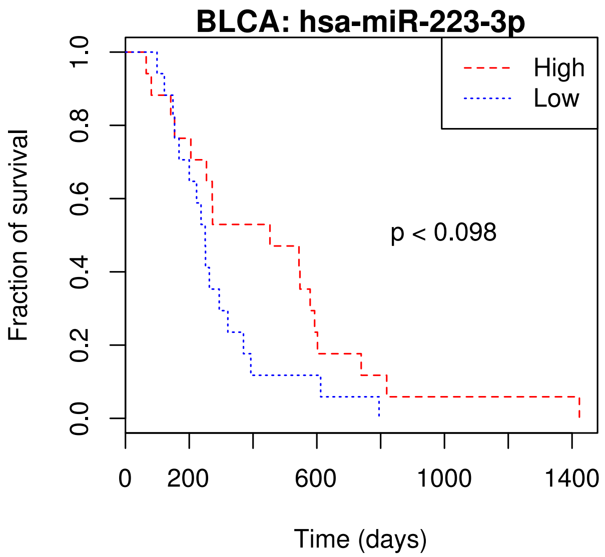
BLCA: hsa-miR-223-3p
High
Low
p < 0.098
Fraction of survival
Time (days)
0.0 0.2 0.4 0.6 0.8 1.0
0 200 600 1000 1400

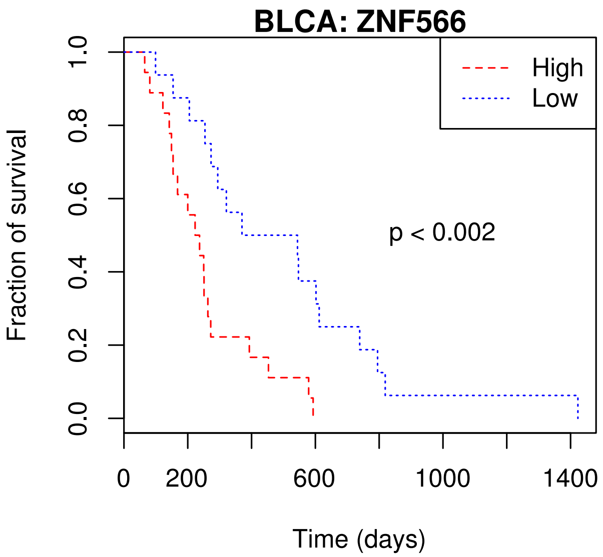
BLCA: ZNF566
High
Low
p < 0.002
Fraction of survival
Time (days)
0.0 0.2 0.4 0.6 0.8 1.0
0 200 600 1000 1400

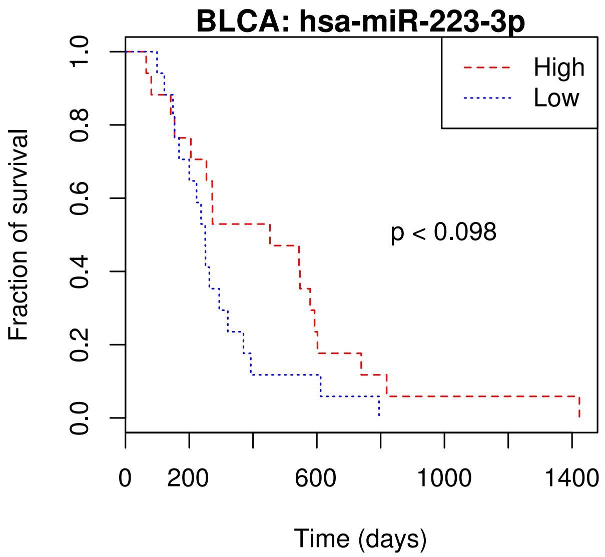
BLCA: hsa-miR-223-3p
High
Low
p < 0.098
Fraction of survival
Time (days)
0.0 0.2 0.4 0.6 0.8 1.0
0 200 600 1000 1400

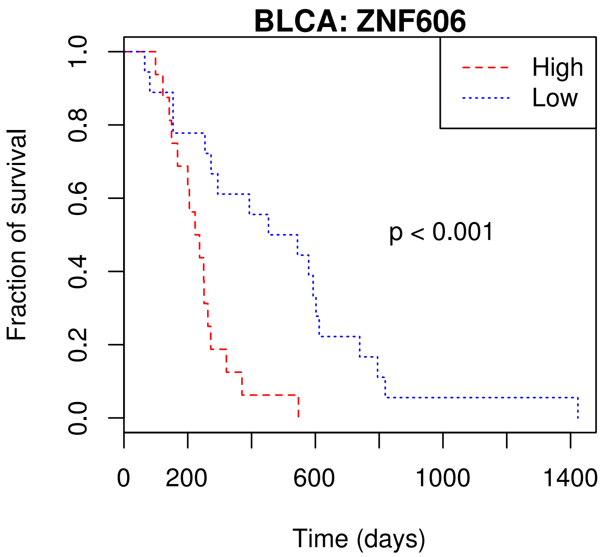
BLCA: ZNF606
High
Low
p < 0.001
Fraction of survival
Time (days)
0.0 0.2 0.4 0.6 0.8 1.0
0 200 600 1000 1400

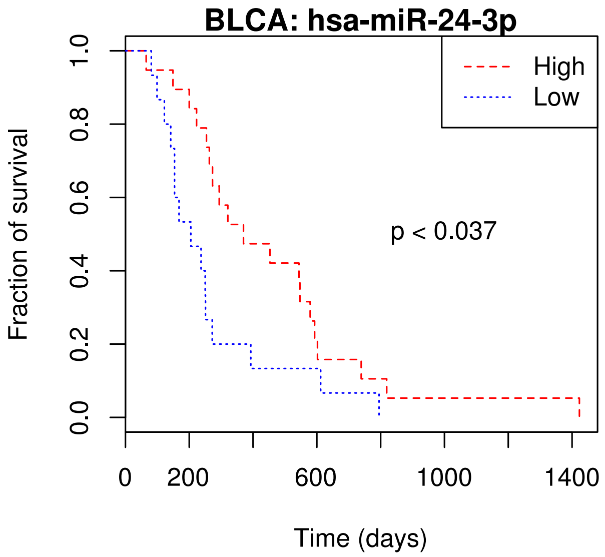
BLCA: hsa-miR-24-3p
High
Low
p < 0.037
Fraction of survival
Time (days)
0.0 0.2 0.4 0.6 0.8 1.0
0 200 600 1000 1400

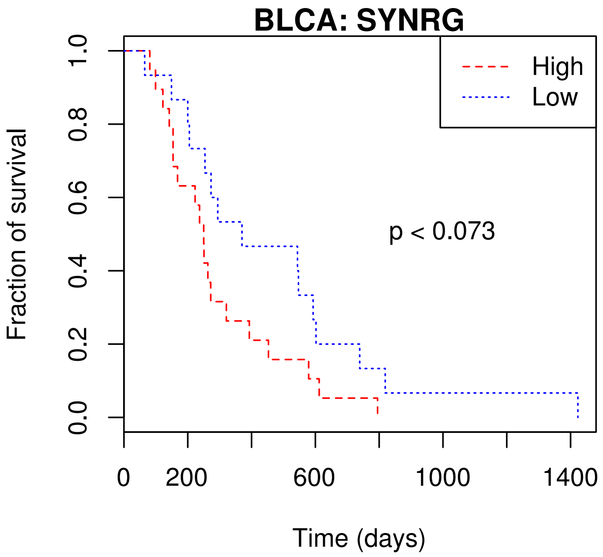
BLCA: SYNRG
High
Low
p < 0.073
Fraction of survival
Time (days)
0.0 0.2 0.4 0.6 0.8 1.0
0 200 600 1000 1400

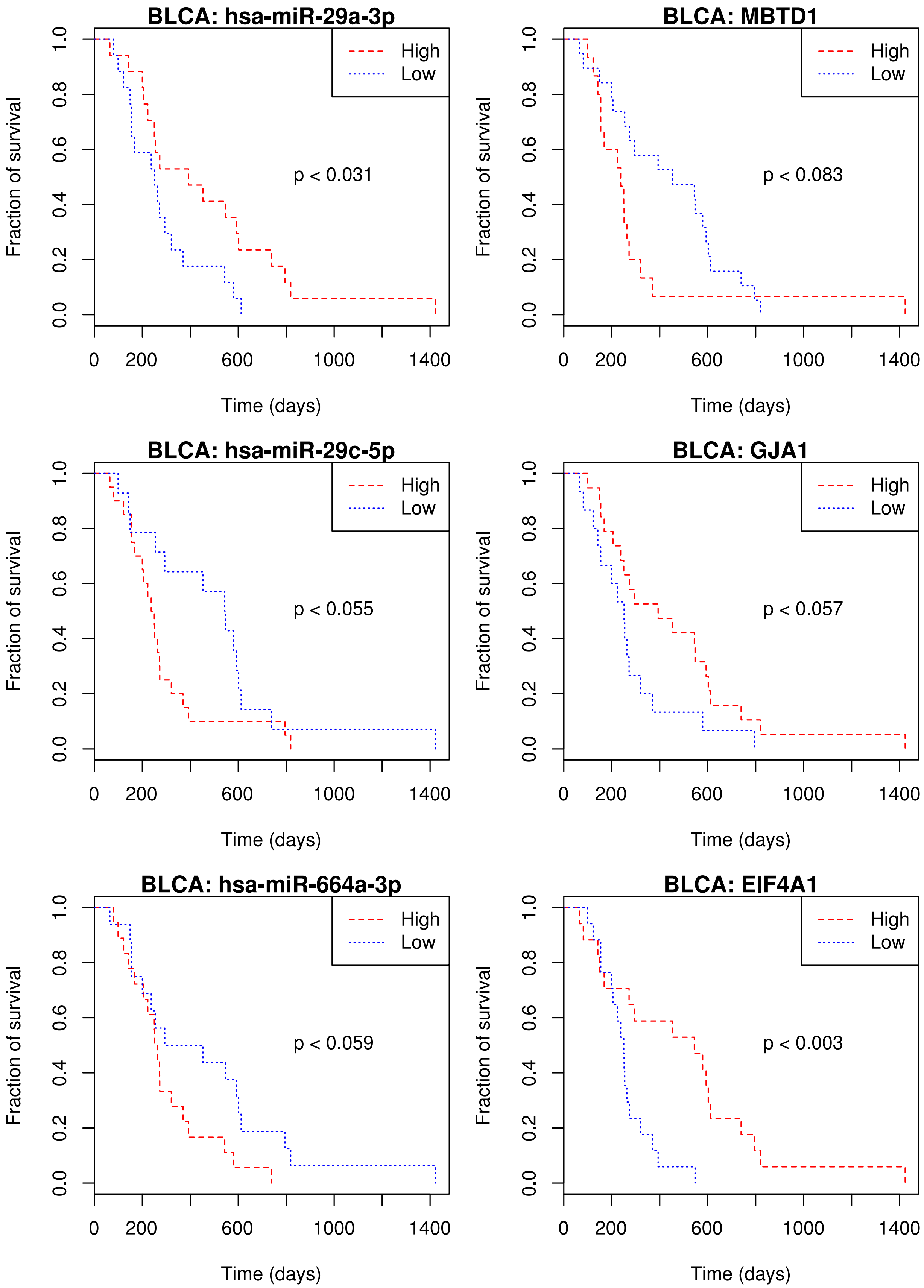
BLCA: hsa-miR-29a-3p
p < 0.031
BLCA: MBTD1
p < 0.083
BLCA: hsa-miR-29c-5p
p < 0.055
BLCA: GJA1
p < 0.057
BLCA: hsa-miR-664a-3p
p < 0.059
BLCA: EIF4A1
p < 0.003
High
Low
Fraction of survival
Time (days)
0.0
0.2
0.4
0.6
0.8
1.0
0
200
600
1000
1400

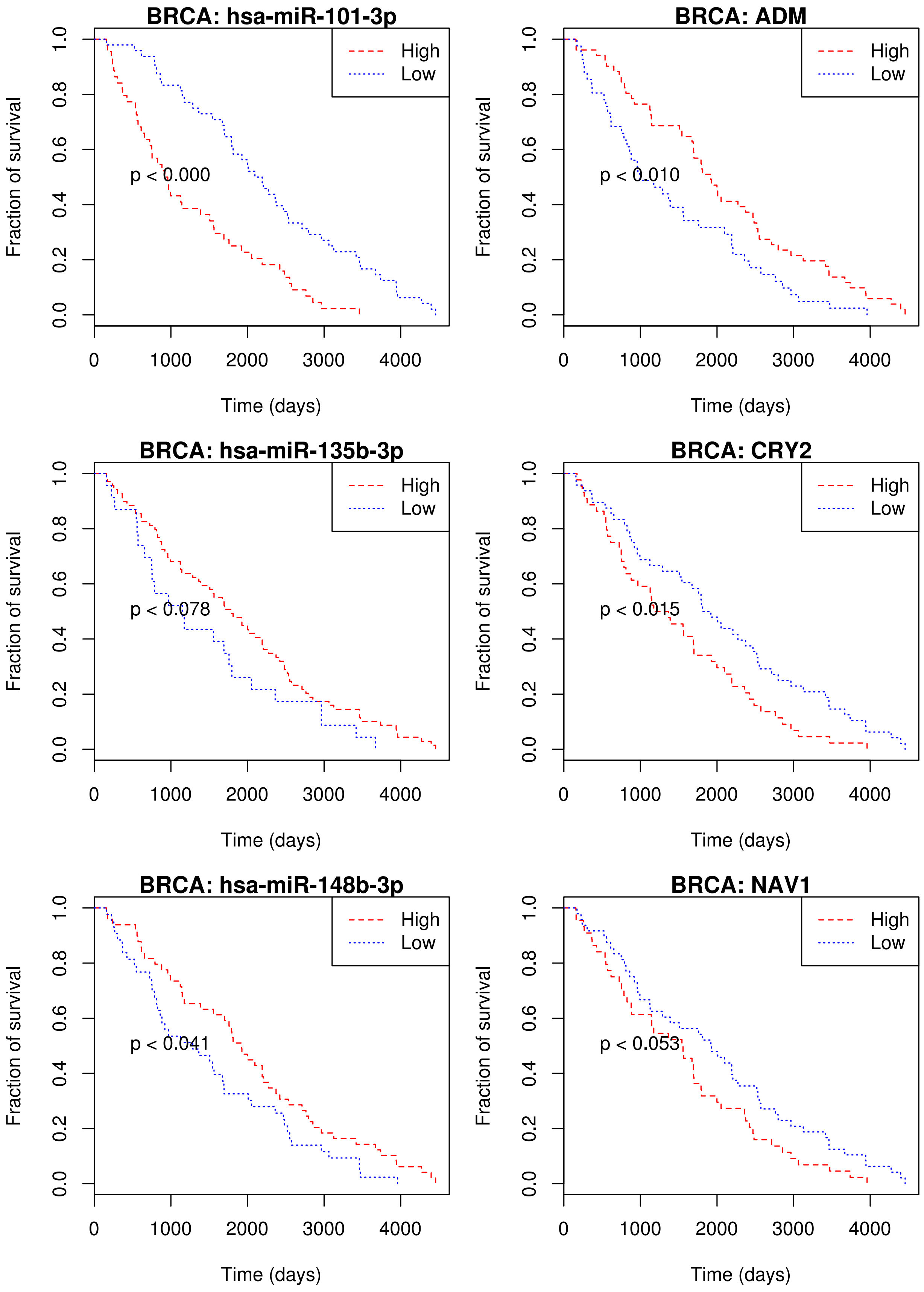
BRCA: hsa-miR-101-3p
p < 0.000
BRCA: ADM
p < 0.010
BRCA: hsa-miR-135b-3p
p < 0.078
BRCA: CRY2
p < 0.015
BRCA: hsa-miR-148b-3p
p < 0.041
BRCA: NAV1
p < 0.053
High
Low
Fraction of survival
Time (days)
0.0
0.2
0.4
0.6
0.8
1.0
0
1000
2000
3000
4000

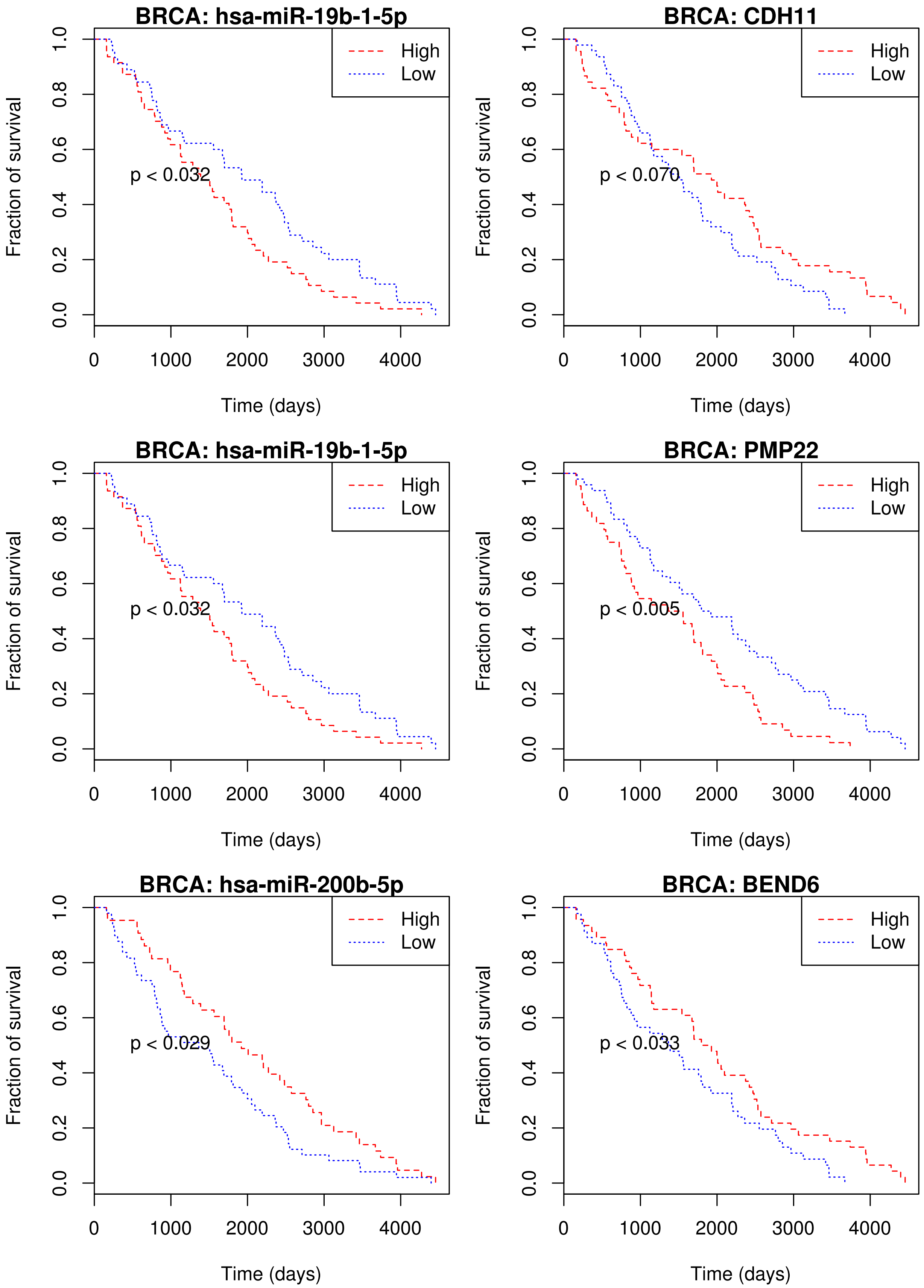
BRCA: hsa-miR-19b-1-5p
High
Low
p < 0.032
Fraction of survival
Time (days)
BRCA: CDH11
High
Low
p < 0.070
Fraction of survival
Time (days)
BRCA: hsa-miR-19b-1-5p
High
Low
p < 0.032
Fraction of survival
Time (days)
BRCA: PMP22
High
Low
p < 0.005
Fraction of survival
Time (days)
BRCA: hsa-miR-200b-5p
High
Low
p < 0.029
Fraction of survival
Time (days)
BRCA: BEND6
High
Low
p < 0.033
Fraction of survival
Time (days)

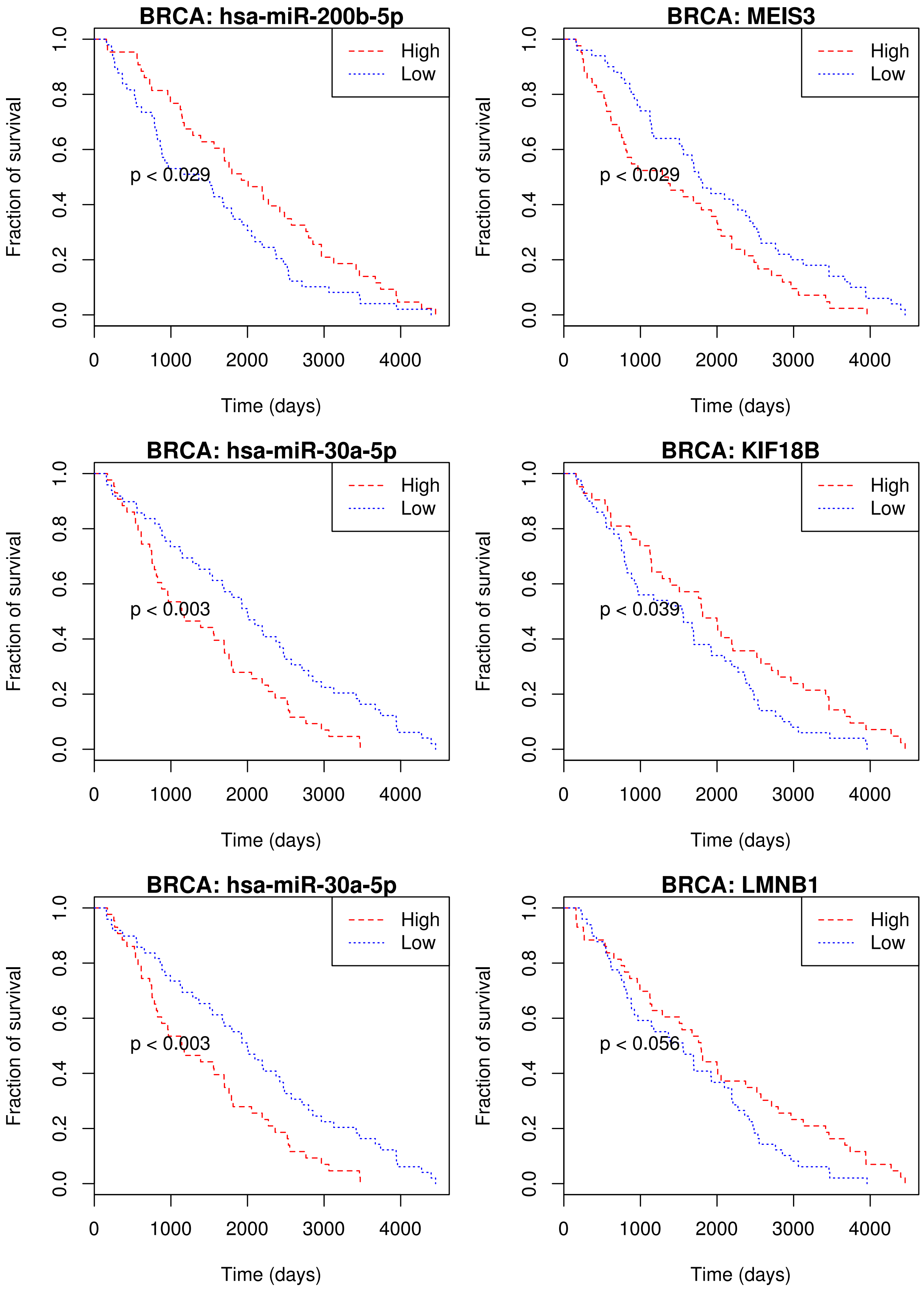
BRCA: hsa-miR-200b-5p
High
Low
p < 0.029
Fraction of survival
Time (days)
BRCA: MEIS3
High
Low
p < 0.029
Fraction of survival
Time (days)
BRCA: hsa-miR-30a-5p
High
Low
p < 0.003
Fraction of survival
Time (days)
BRCA: KIF18B
High
Low
p < 0.039
Fraction of survival
Time (days)
BRCA: hsa-miR-30a-5p
High
Low
p < 0.003
Fraction of survival
Time (days)
BRCA: LMNB1
High
Low
p < 0.056
Fraction of survival
Time (days)

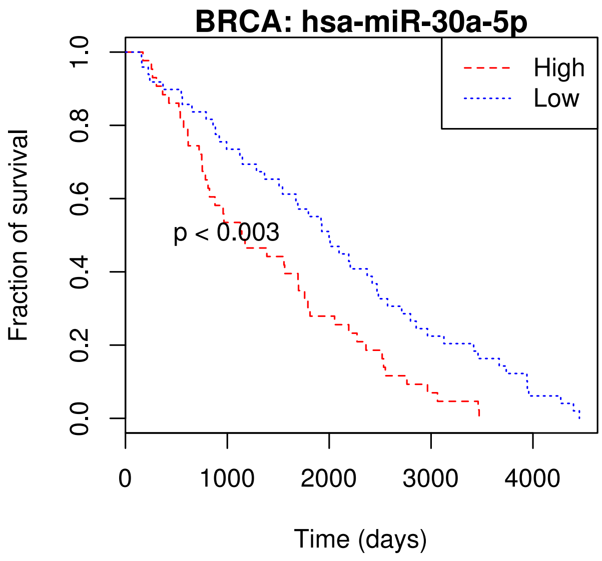
BRCA: hsa-miR-30a-5p
High
Low
p < 0.003
Fraction of survival
0.0
0.2
0.4
0.6
0.8
1.0
0
1000
2000
3000
4000
Time (days)

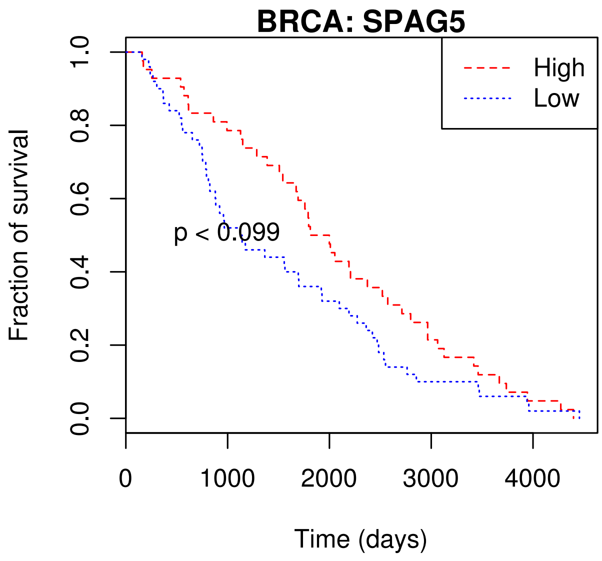
BRCA: SPAG5
High
Low
p < 0.099
Fraction of survival
0.0
0.2
0.4
0.6
0.8
1.0
0
1000
2000
3000
4000
Time (days)

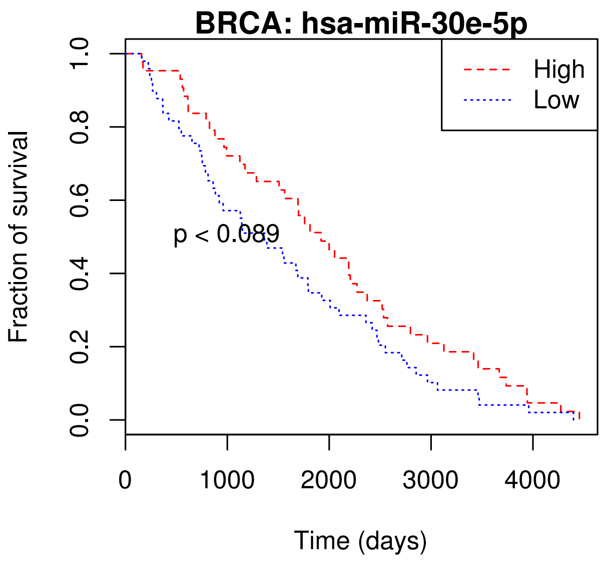
BRCA: hsa-miR-30e-5p
High
Low
p < 0.089
Fraction of survival
0.0
0.2
0.4
0.6
0.8
1.0
0
1000
2000
3000
4000
Time (days)

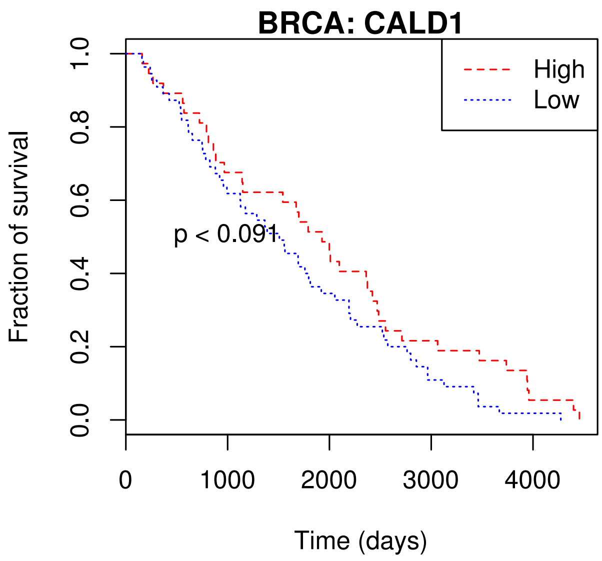
BRCA: CALD1
High
Low
p < 0.091
Fraction of survival
0.0
0.2
0.4
0.6
0.8
1.0
0
1000
2000
3000
4000
Time (days)

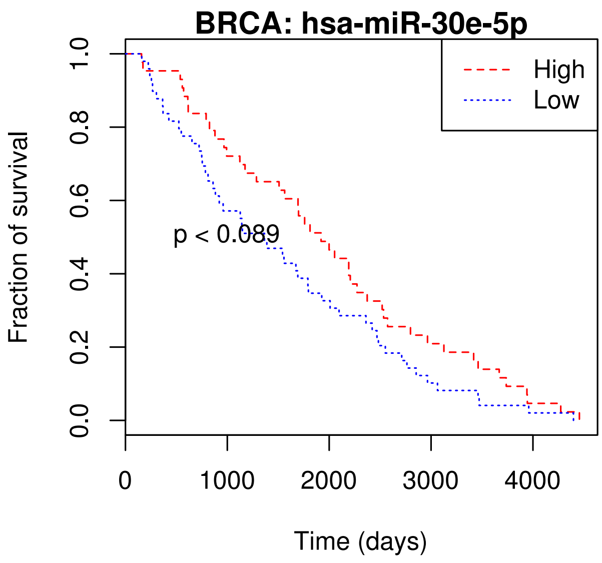
BRCA: hsa-miR-30e-5p
High
Low
p < 0.089
Fraction of survival
0.0
0.2
0.4
0.6
0.8
1.0
0
1000
2000
3000
4000
Time (days)

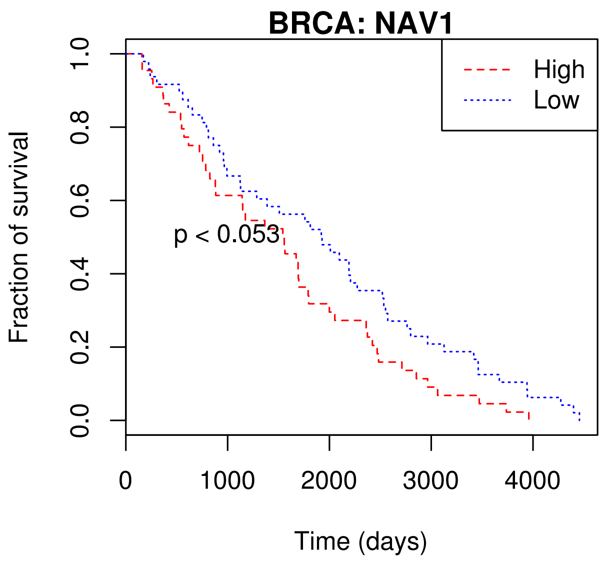
BRCA: NAV1
High
Low
p < 0.053
Fraction of survival
0.0
0.2
0.4
0.6
0.8
1.0
0
1000
2000
3000
4000
Time (days)

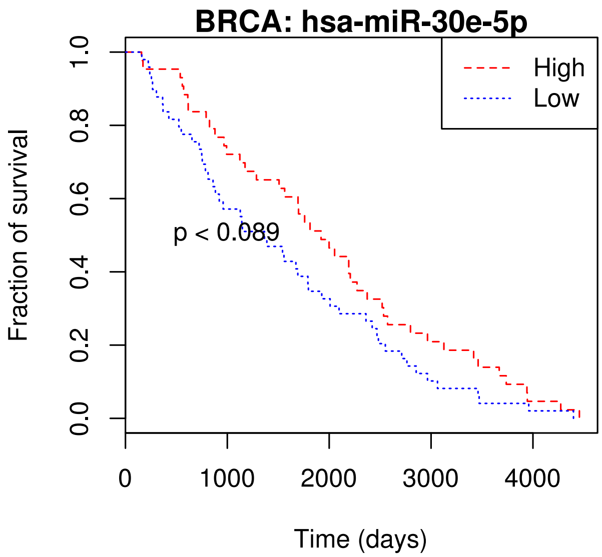
BRCA: hsa-miR-30e-5p
High
Low
p < 0.089
Fraction of survival
Time (days)
0.0
0.2
0.4
0.6
0.8
1.0
0
1000
2000
3000
4000

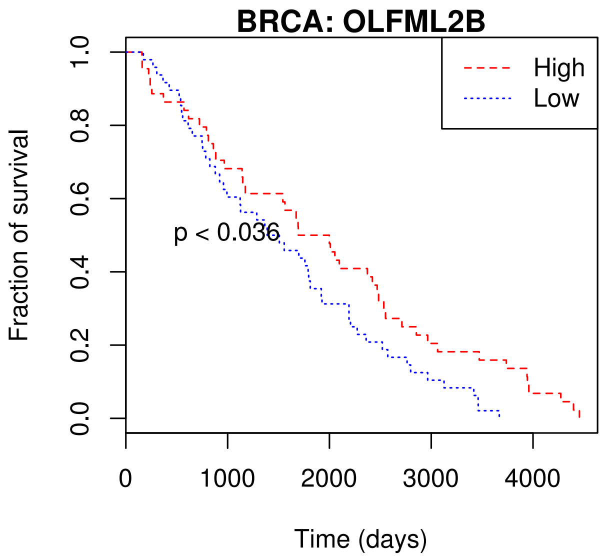
BRCA: OLFML2B
High
Low
p < 0.036
Fraction of survival
Time (days)
0.0
0.2
0.4
0.6
0.8
1.0
0
1000
2000
3000
4000

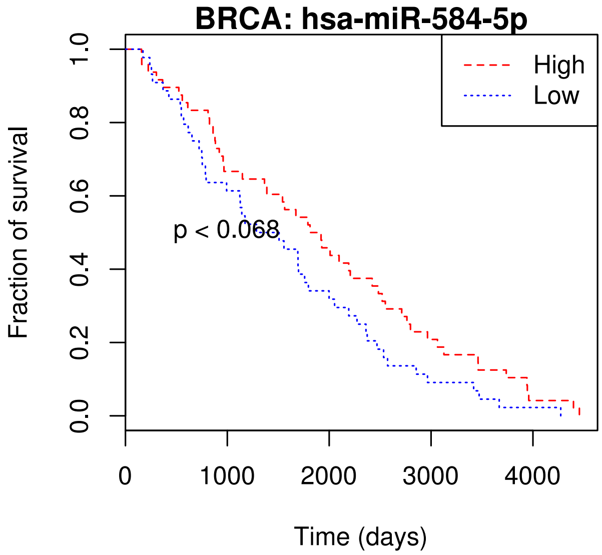
BRCA: hsa-miR-584-5p
High
Low
p < 0.068
Fraction of survival
Time (days)
0.0
0.2
0.4
0.6
0.8
1.0
0
1000
2000
3000
4000

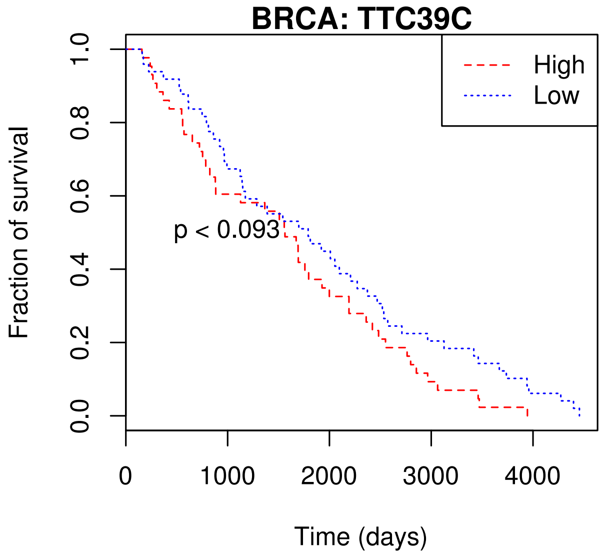
BRCA: TTC39C
High
Low
p < 0.093
Fraction of survival
Time (days)
0.0
0.2
0.4
0.6
0.8
1.0
0
1000
2000
3000
4000

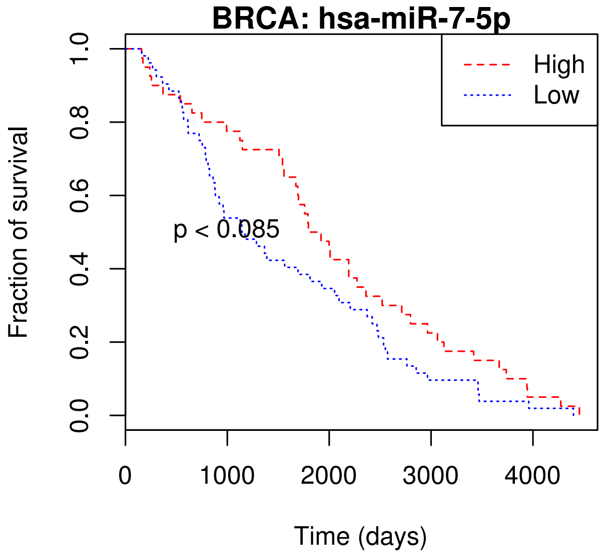
BRCA: hsa-miR-7-5p
High
Low
p < 0.085
Fraction of survival
Time (days)
0.0
0.2
0.4
0.6
0.8
1.0
0
1000
2000
3000
4000

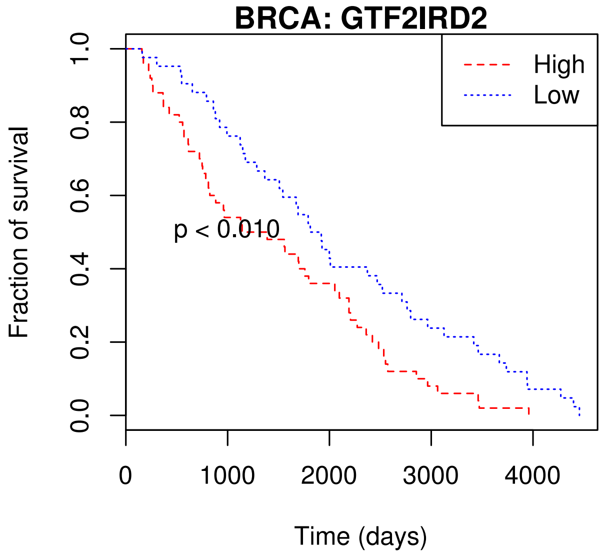
BRCA: GTF2IRD2
High
Low
p < 0.010
Fraction of survival
Time (days)
0.0
0.2
0.4
0.6
0.8
1.0
0
1000
2000
3000
4000

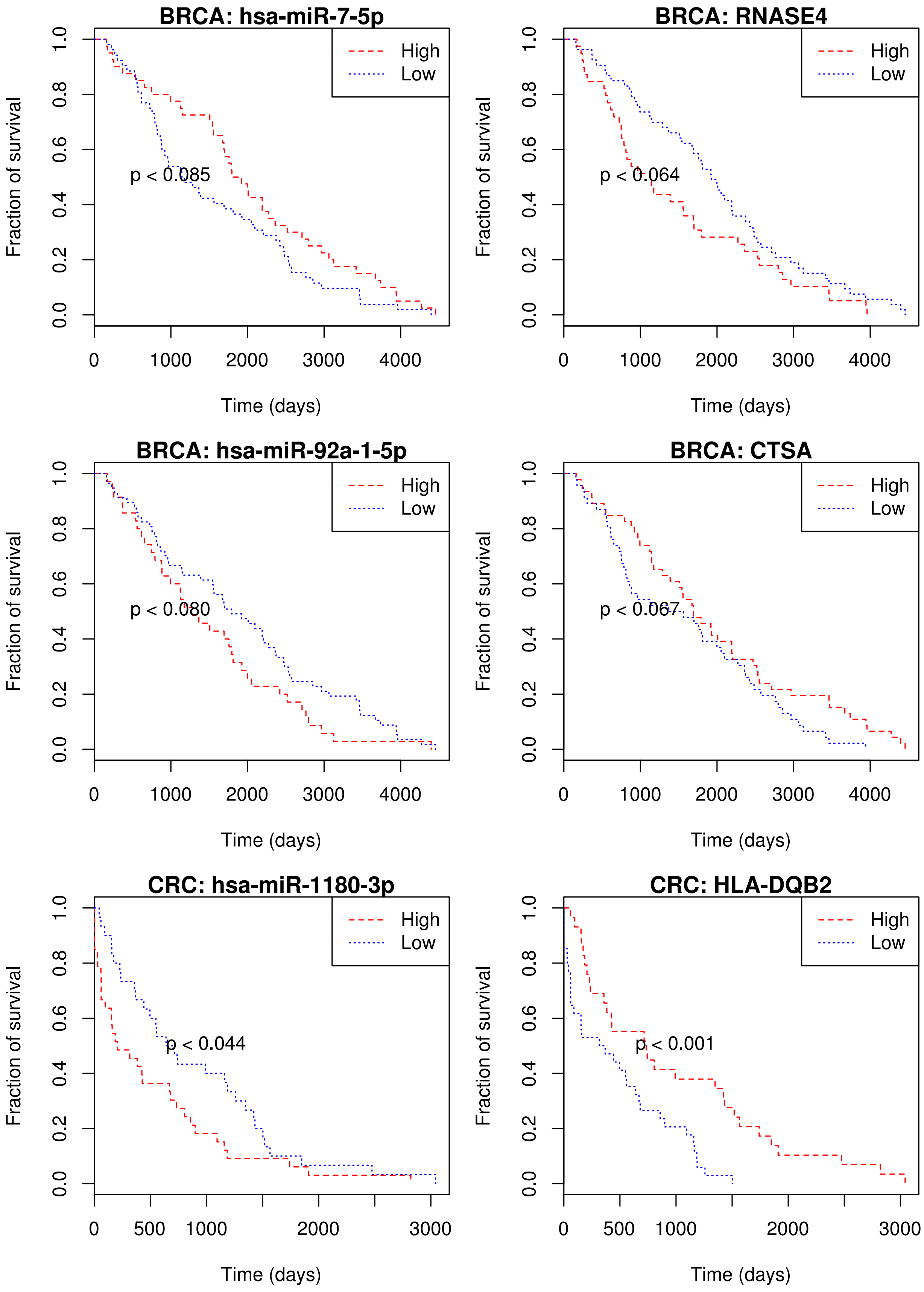
BRCA: hsa-miR-7-5p
p < 0.085
BRCA: RNASE4
p < 0.064
BRCA: hsa-miR-92a-1-5p
p < 0.080
BRCA: CTSA
p < 0.067
CRC: hsa-miR-1180-3p
p < 0.044
CRC: HLA-DQB2
p < 0.001
High
Low
Fraction of survival
Time (days)

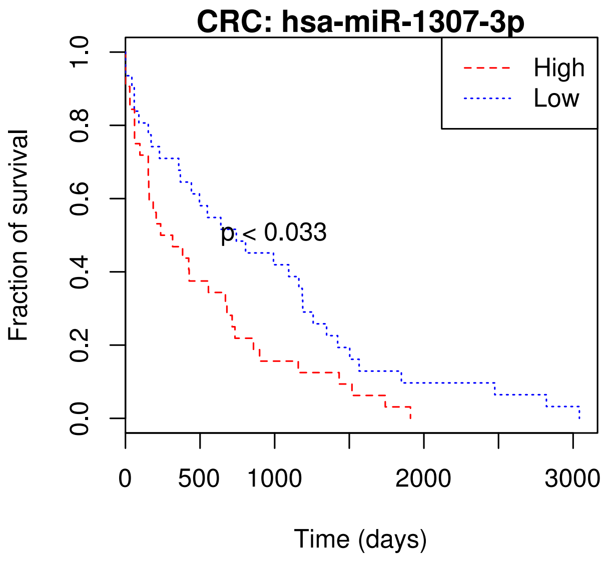
CRC: hsa-miR-1307-3p
High
Low
p < 0.033
Fraction of survival
0.0
0.2
0.4
0.6
0.8
1.0
0
500
1000
2000
3000
Time (days)

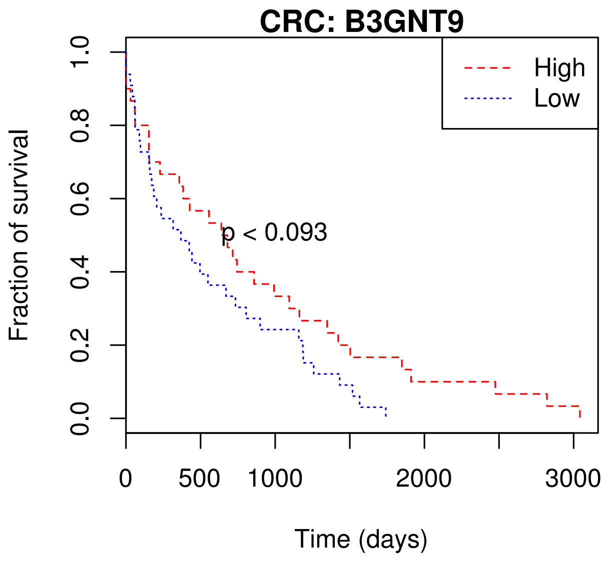
CRC: B3GNT9
High
Low
p < 0.093
Fraction of survival
0.0
0.2
0.4
0.6
0.8
1.0
0
500
1000
2000
3000
Time (days)

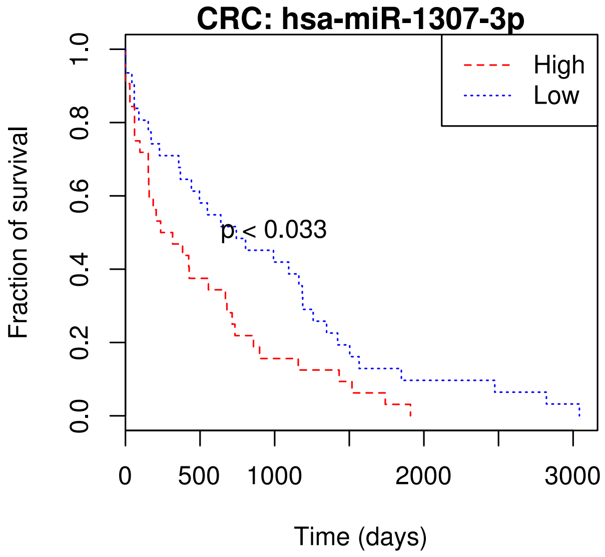
CRC: hsa-miR-1307-3p
High
Low
p < 0.033
Fraction of survival
0.0
0.2
0.4
0.6
0.8
1.0
0
500
1000
2000
3000
Time (days)

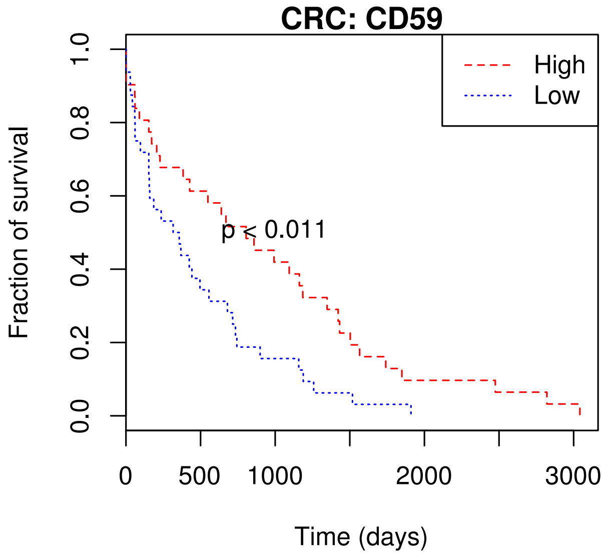
CRC: CD59
High
Low
p < 0.011
Fraction of survival
0.0
0.2
0.4
0.6
0.8
1.0
0
500
1000
2000
3000
Time (days)

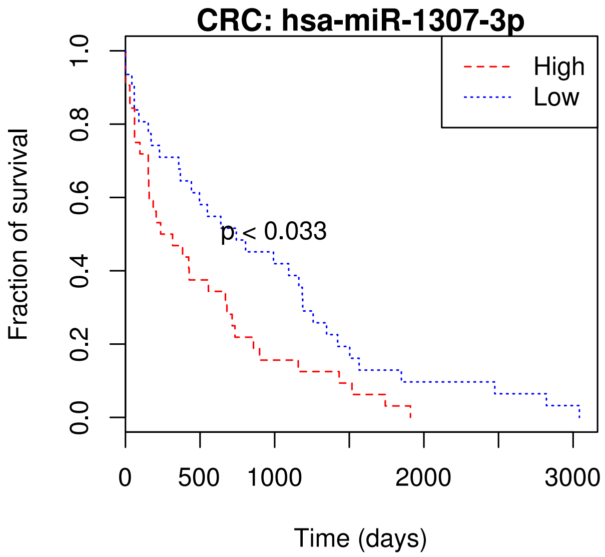
CRC: hsa-miR-1307-3p
High
Low
p < 0.033
Fraction of survival
0.0
0.2
0.4
0.6
0.8
1.0
0
500
1000
2000
3000
Time (days)

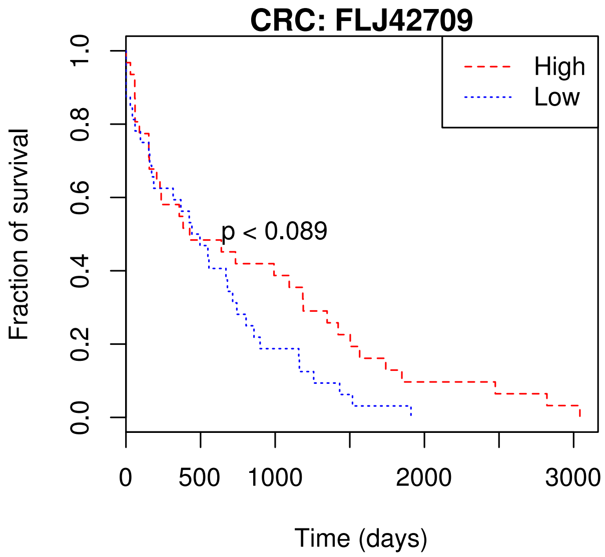
CRC: FLJ42709
High
Low
p < 0.089
Fraction of survival
0.0
0.2
0.4
0.6
0.8
1.0
0
500
1000
2000
3000
Time (days)

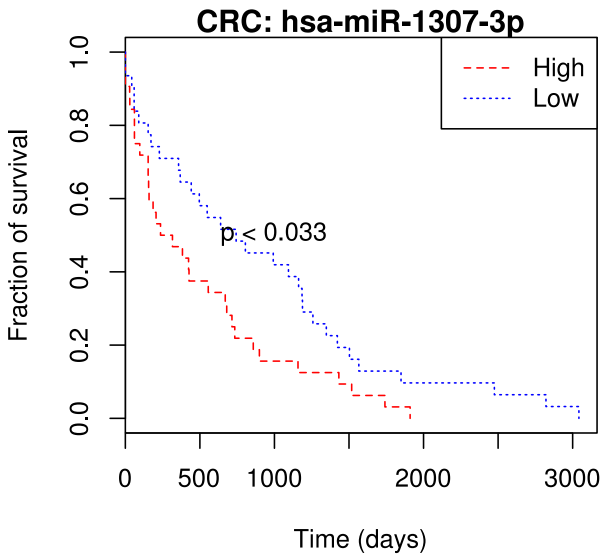
CRC: hsa-miR-1307-3p
High
Low
p < 0.033
Fraction of survival
Time (days)
0.0
0.2
0.4
0.6
0.8
1.0
0
500
1000
2000
3000

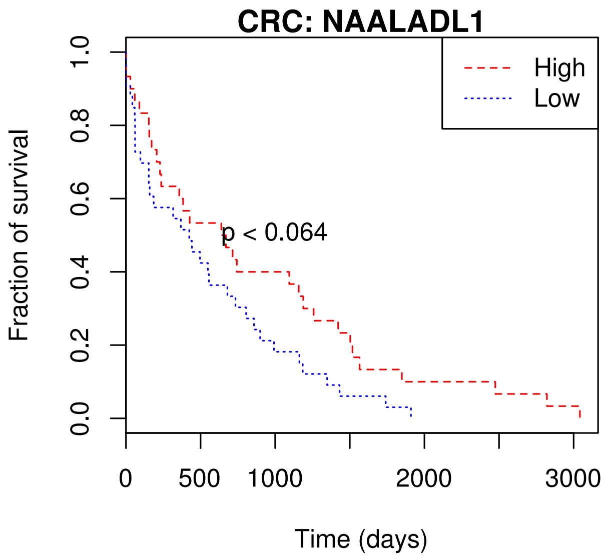
CRC: NAALADL1
High
Low
p < 0.064
Fraction of survival
Time (days)
0.0
0.2
0.4
0.6
0.8
1.0
0
500
1000
2000
3000

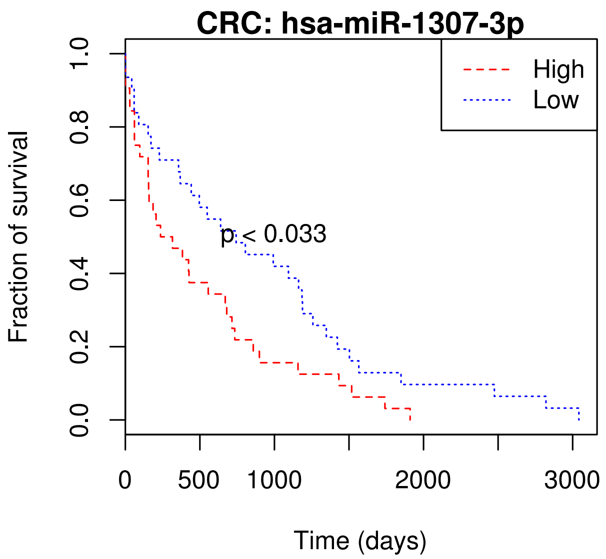
CRC: hsa-miR-1307-3p
High
Low
p < 0.033
Fraction of survival
Time (days)
0.0
0.2
0.4
0.6
0.8
1.0
0
500
1000
2000
3000

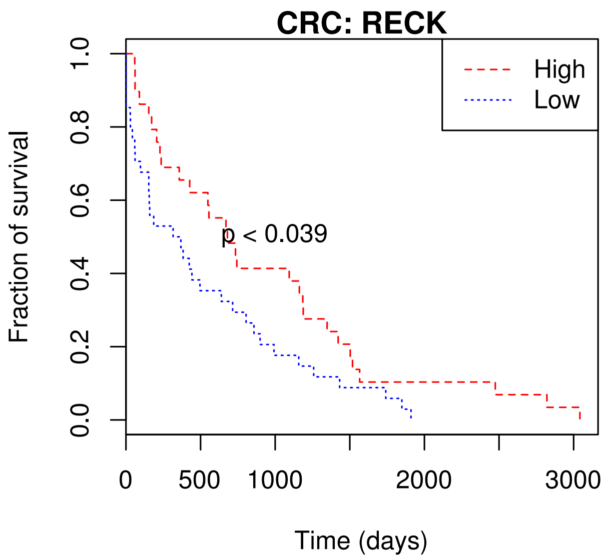
CRC: RECK
High
Low
p < 0.039
Fraction of survival
Time (days)
0.0
0.2
0.4
0.6
0.8
1.0
0
500
1000
2000
3000

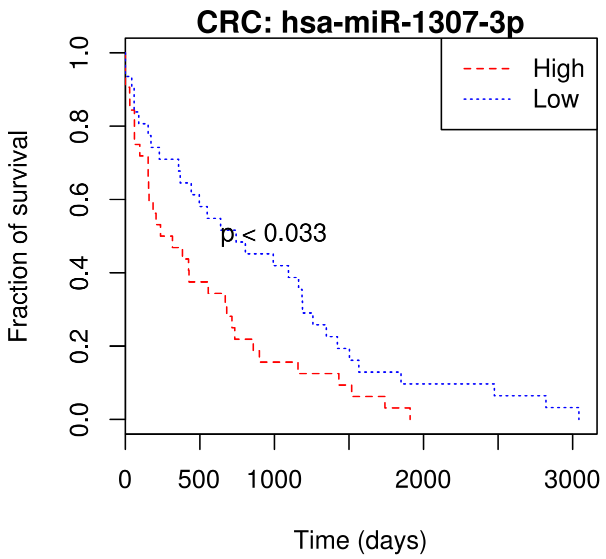
CRC: hsa-miR-1307-3p
High
Low
p < 0.033
Fraction of survival
Time (days)
0.0
0.2
0.4
0.6
0.8
1.0
0
500
1000
2000
3000

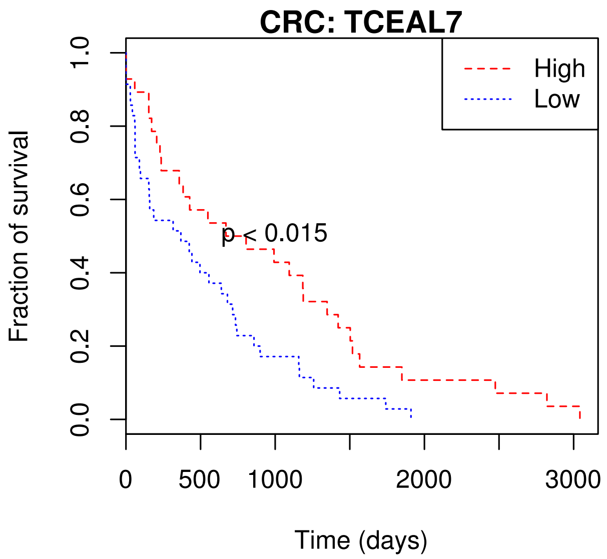
CRC: TCEAL7
High
Low
p < 0.015
Fraction of survival
Time (days)
0.0
0.2
0.4
0.6
0.8
1.0
0
500
1000
2000
3000

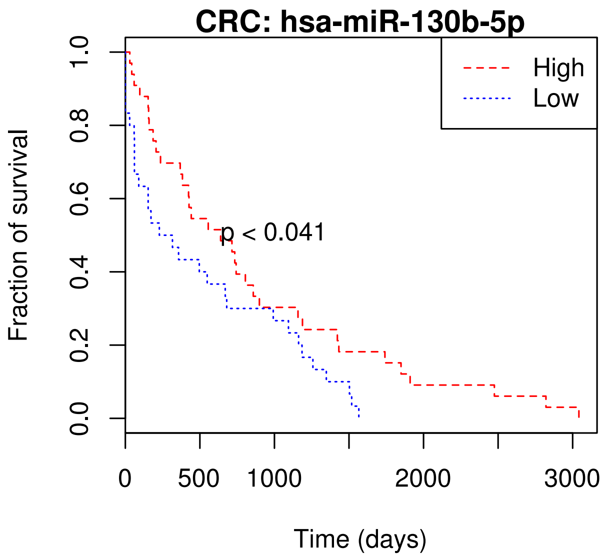
CRC: hsa-miR-130b-5p
High
Low
p < 0.041
Fraction of survival
0.0
0.2
0.4
0.6
0.8
1.0
0
500
1000
2000
3000
Time (days)

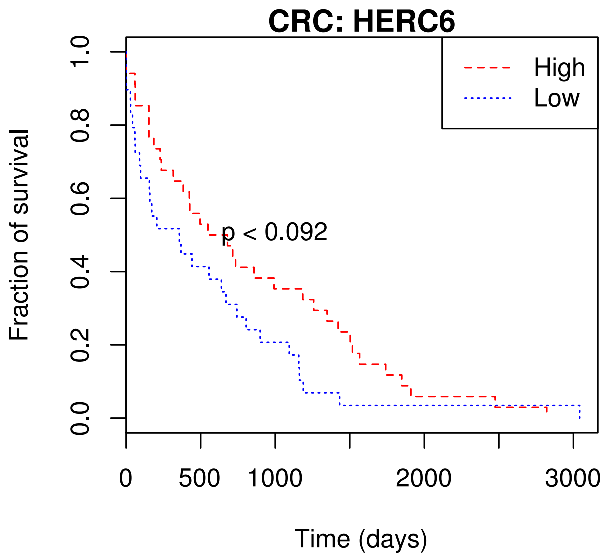
CRC: HERC6
High
Low
p < 0.092
Fraction of survival
0.0
0.2
0.4
0.6
0.8
1.0
0
500
1000
2000
3000
Time (days)

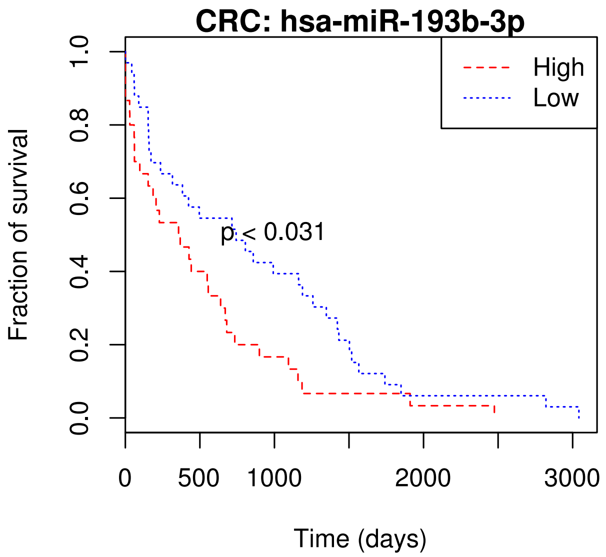
CRC: hsa-miR-193b-3p
High
Low
p < 0.031
Fraction of survival
0.0
0.2
0.4
0.6
0.8
1.0
0
500
1000
2000
3000
Time (days)

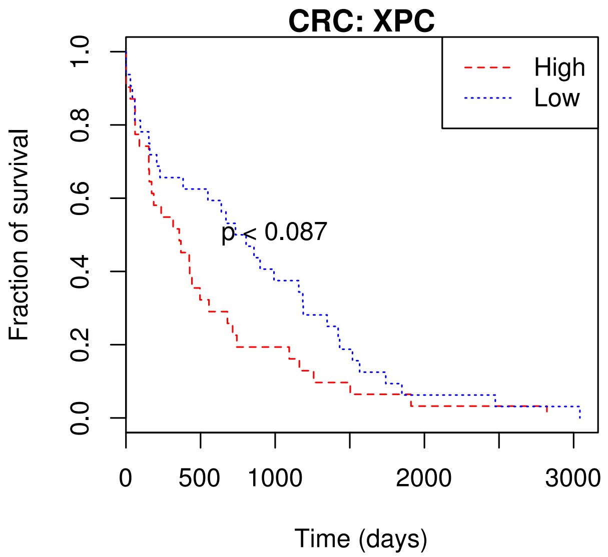
CRC: XPC
High
Low
p < 0.087
Fraction of survival
0.0
0.2
0.4
0.6
0.8
1.0
0
500
1000
2000
3000
Time (days)

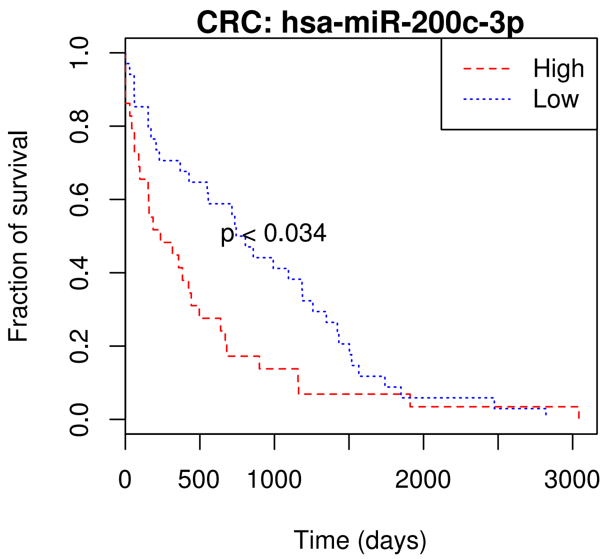
CRC: hsa-miR-200c-3p
High
Low
p < 0.034
Fraction of survival
0.0
0.2
0.4
0.6
0.8
1.0
0
500
1000
2000
3000
Time (days)

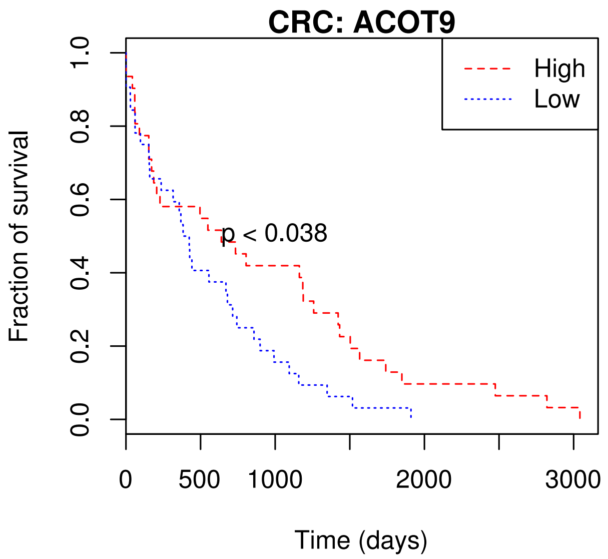
CRC: ACOT9
High
Low
p < 0.038
Fraction of survival
0.0
0.2
0.4
0.6
0.8
1.0
0
500
1000
2000
3000
Time (days)

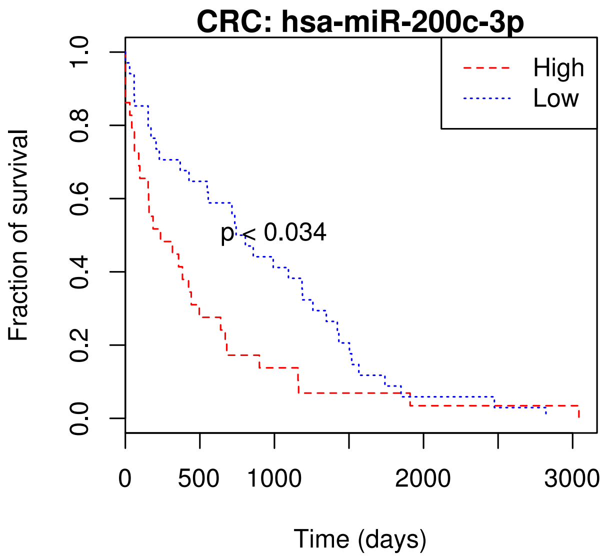
CRC: hsa-miR-200c-3p
High
Low
p < 0.034
Fraction of survival
Time (days)
0.0 0.2 0.4 0.6 0.8 1.0
0 500 1000 2000 3000

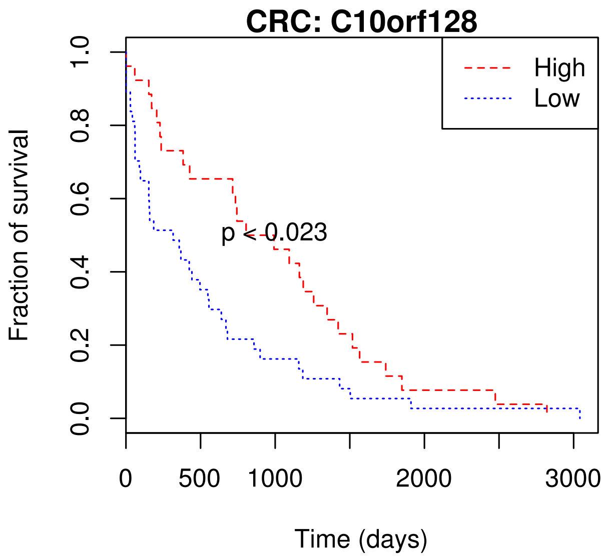
CRC: C10orf128
High
Low
p < 0.023
Fraction of survival
Time (days)
0.0 0.2 0.4 0.6 0.8 1.0
0 500 1000 2000 3000

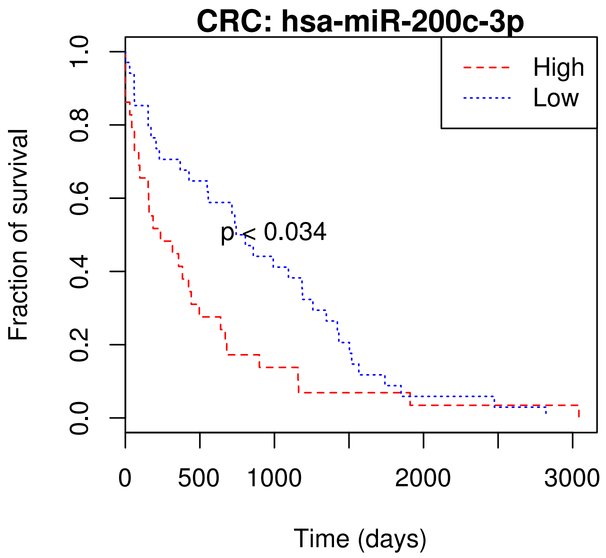
CRC: hsa-miR-200c-3p
High
Low
p < 0.034
Fraction of survival
Time (days)
0.0 0.2 0.4 0.6 0.8 1.0
0 500 1000 2000 3000

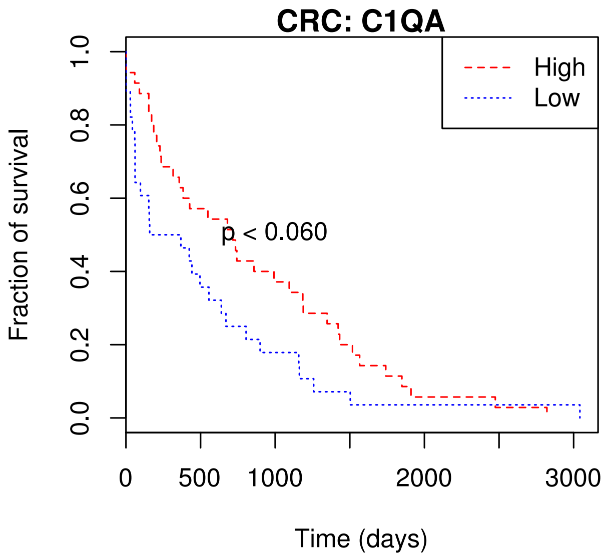
CRC: C1QA
High
Low
p < 0.060
Fraction of survival
Time (days)
0.0 0.2 0.4 0.6 0.8 1.0
0 500 1000 2000 3000

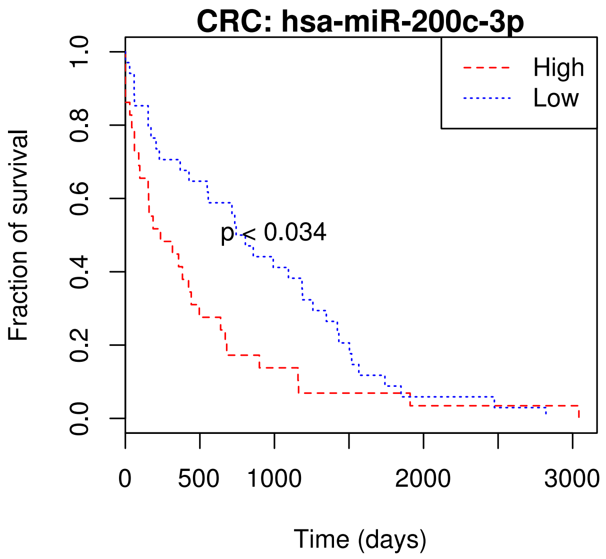
CRC: hsa-miR-200c-3p
High
Low
p < 0.034
Fraction of survival
Time (days)
0.0 0.2 0.4 0.6 0.8 1.0
0 500 1000 2000 3000

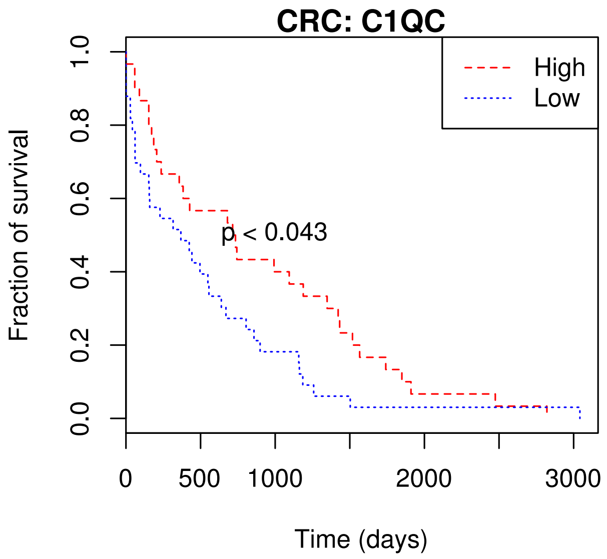
CRC: C1QC
High
Low
p < 0.043
Fraction of survival
Time (days)
0.0 0.2 0.4 0.6 0.8 1.0
0 500 1000 2000 3000

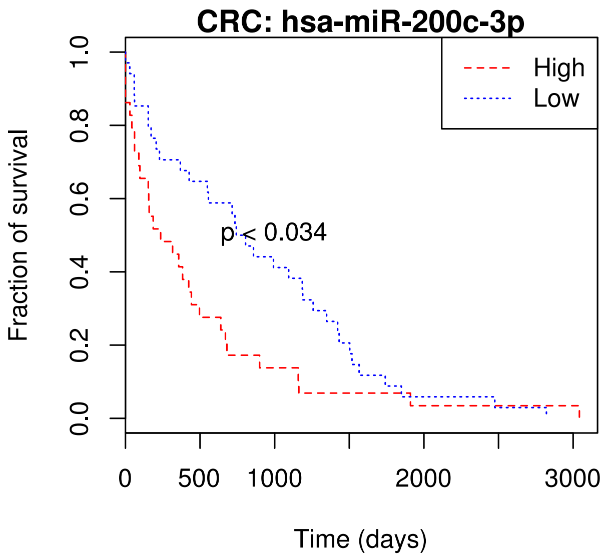
CRC: hsa-miR-200c-3p
High
Low
p < 0.034
Fraction of survival
0.0 0.2 0.4 0.6 0.8 1.0
0 500 1000 2000 3000
Time (days)

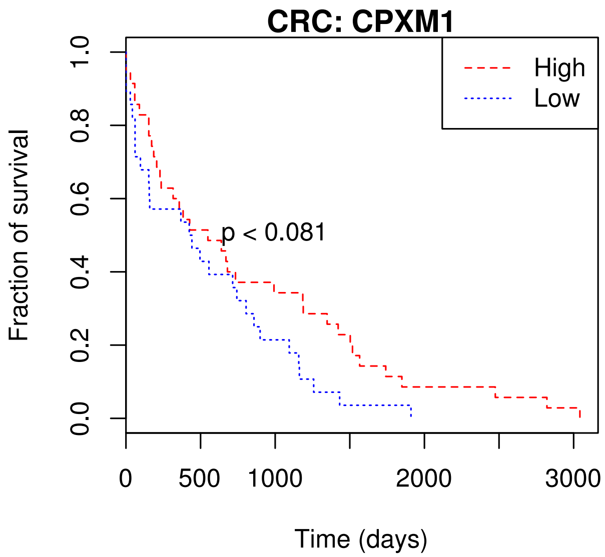
CRC: CPXM1
High
Low
p < 0.081
Fraction of survival
0.0 0.2 0.4 0.6 0.8 1.0
0 500 1000 2000 3000
Time (days)

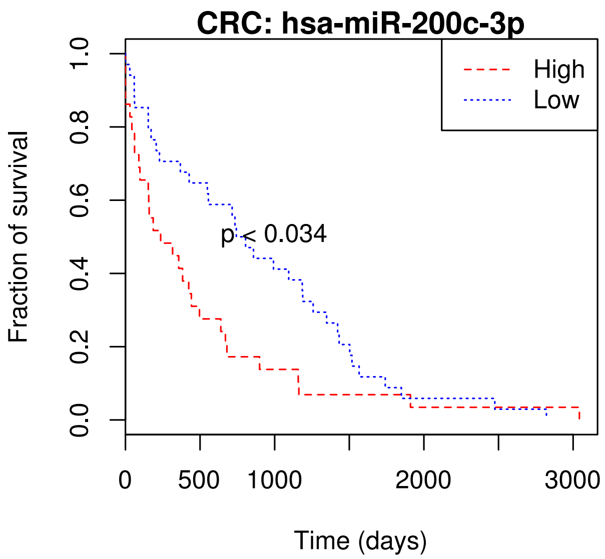
CRC: hsa-miR-200c-3p
High
Low
p < 0.034
Fraction of survival
0.0 0.2 0.4 0.6 0.8 1.0
0 500 1000 2000 3000
Time (days)

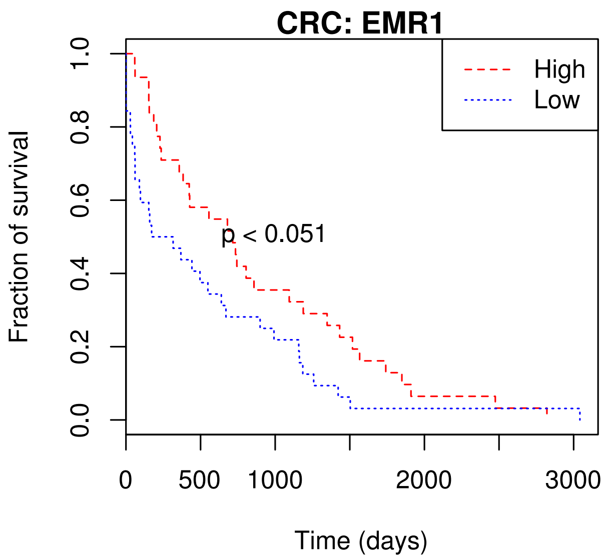
CRC: EMR1
High
Low
p < 0.051
Fraction of survival
0.0 0.2 0.4 0.6 0.8 1.0
0 500 1000 2000 3000
Time (days)

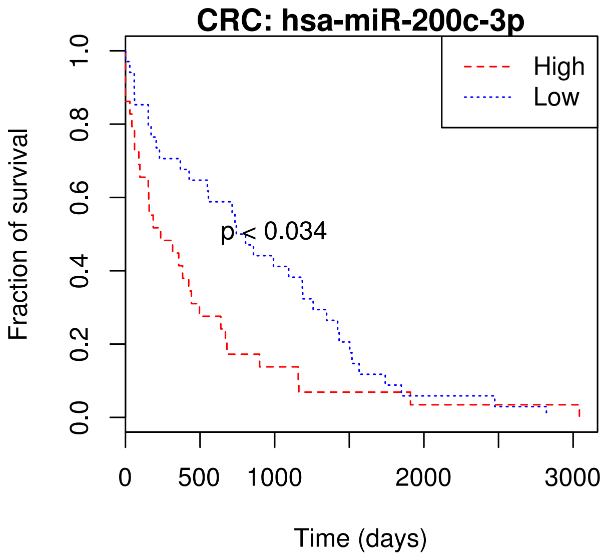
CRC: hsa-miR-200c-3p
High
Low
p < 0.034
Fraction of survival
0.0 0.2 0.4 0.6 0.8 1.0
0 500 1000 2000 3000
Time (days)

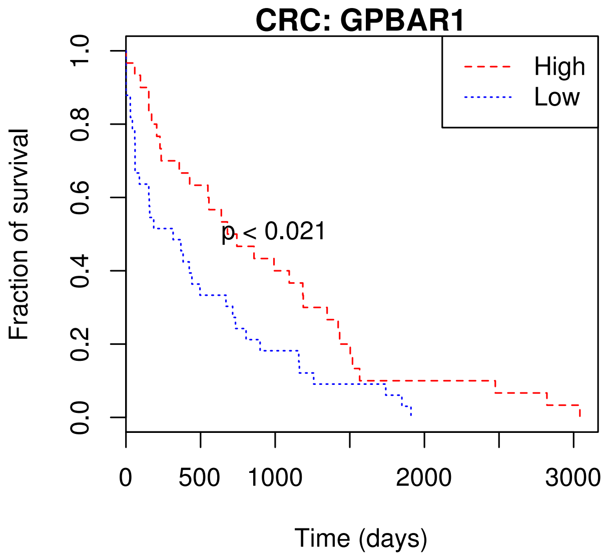
CRC: GPBAR1
High
Low
p < 0.021
Fraction of survival
0.0 0.2 0.4 0.6 0.8 1.0
0 500 1000 2000 3000
Time (days)

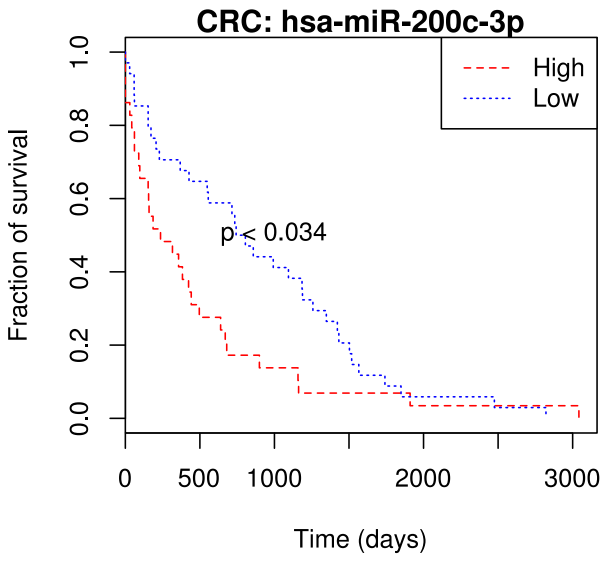
CRC: hsa-miR-200c-3p
High
Low
p < 0.034
Fraction of survival
Time (days)
0.0 0.2 0.4 0.6 0.8 1.0
0 500 1000 2000 3000

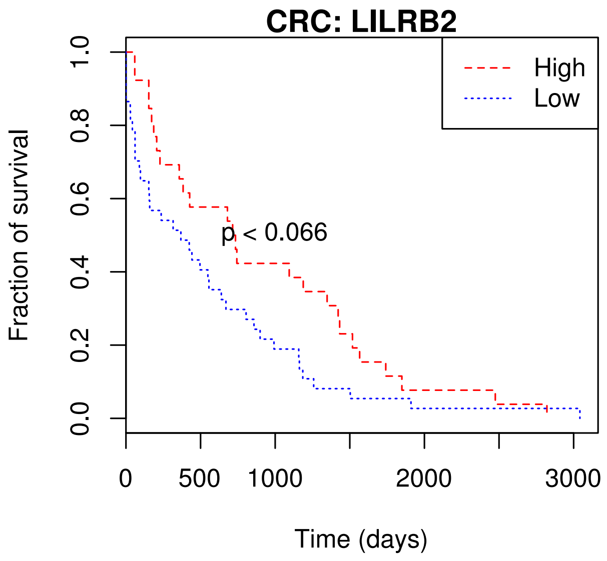
CRC: LILRB2
High
Low
p < 0.066
Fraction of survival
Time (days)
0.0 0.2 0.4 0.6 0.8 1.0
0 500 1000 2000 3000

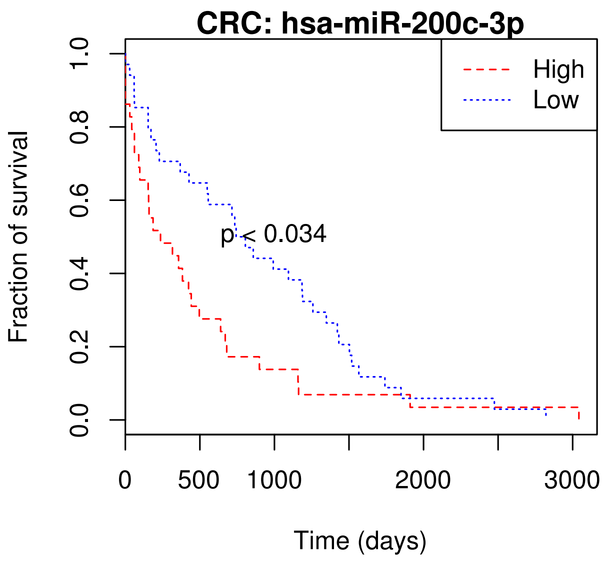
CRC: hsa-miR-200c-3p
High
Low
p < 0.034
Fraction of survival
Time (days)
0.0 0.2 0.4 0.6 0.8 1.0
0 500 1000 2000 3000

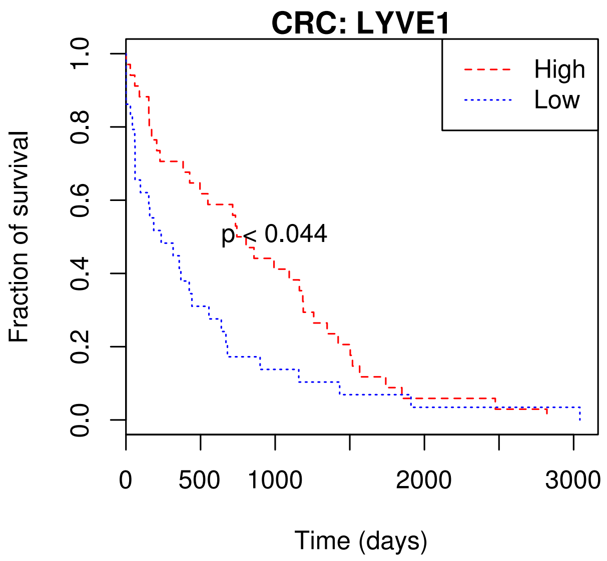
CRC: LYVE1
High
Low
p < 0.044
Fraction of survival
Time (days)
0.0 0.2 0.4 0.6 0.8 1.0
0 500 1000 2000 3000

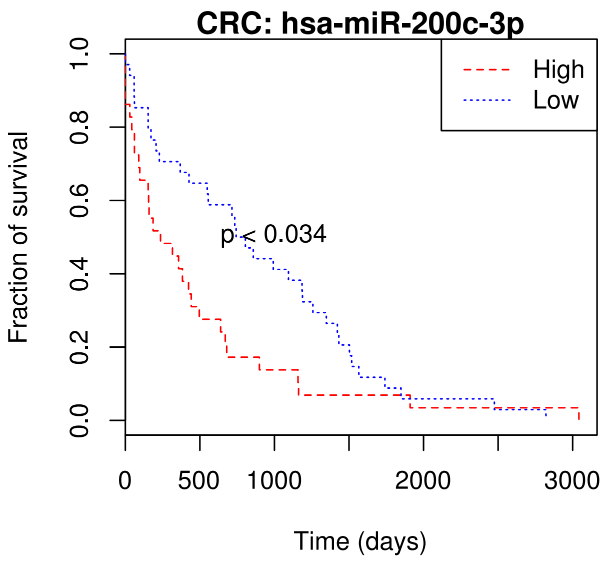
CRC: hsa-miR-200c-3p
High
Low
p < 0.034
Fraction of survival
Time (days)
0.0 0.2 0.4 0.6 0.8 1.0
0 500 1000 2000 3000

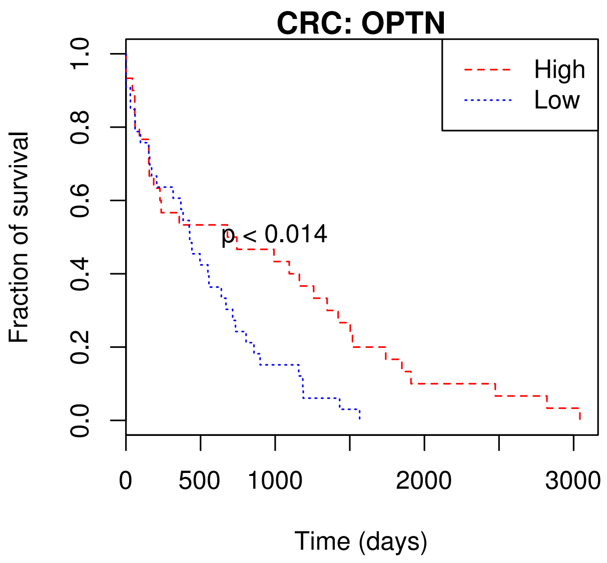
CRC: OPTN
High
Low
p < 0.014
Fraction of survival
Time (days)
0.0 0.2 0.4 0.6 0.8 1.0
0 500 1000 2000 3000

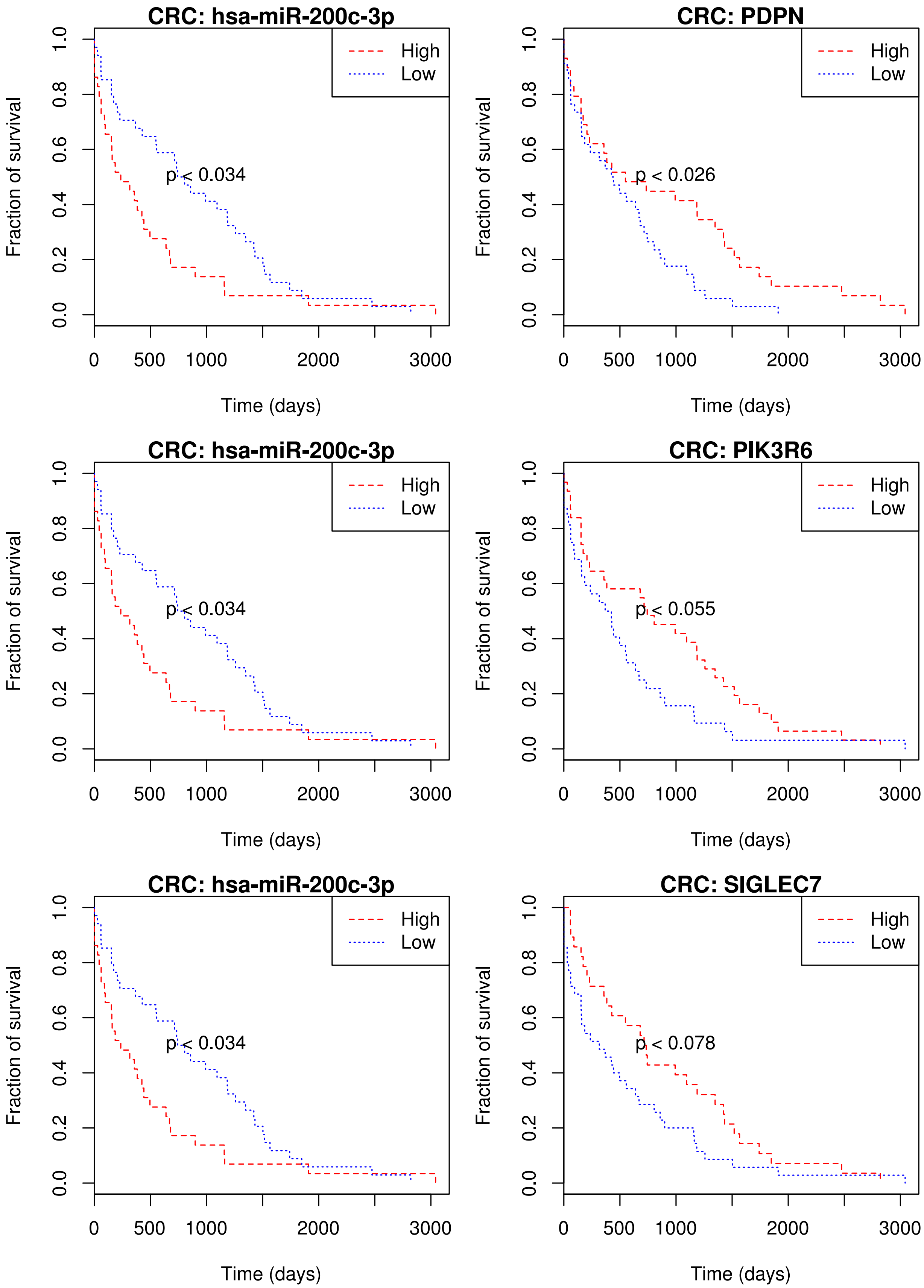
CRC: hsa-miR-200c-3p
High
Low
p < 0.034
Fraction of survival
Time (days)
CRC: PDPN
High
Low
p < 0.026
Fraction of survival
Time (days)
CRC: hsa-miR-200c-3p
High
Low
p < 0.034
Fraction of survival
Time (days)
CRC: PIK3R6
High
Low
p < 0.055
Fraction of survival
Time (days)
CRC: hsa-miR-200c-3p
High
Low
p < 0.034
Fraction of survival
Time (days)
CRC: SIGLEC7
High
Low
p < 0.078
Fraction of survival
Time (days)

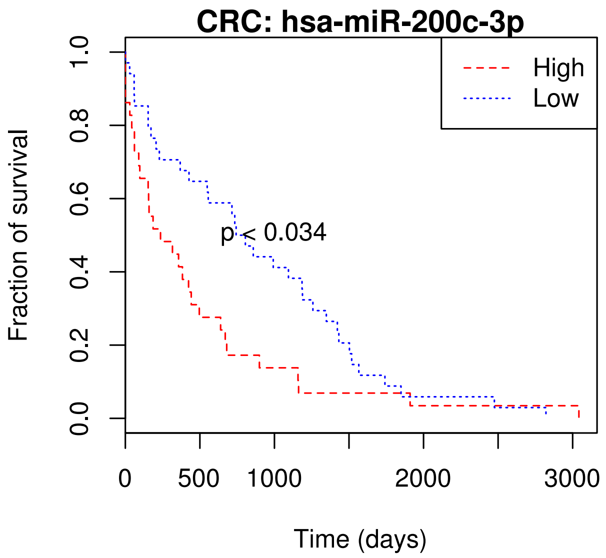
CRC: hsa-miR-200c-3p
High
Low
p < 0.034
Fraction of survival
0.0 0.2 0.4 0.6 0.8 1.0
0 500 1000 2000 3000
Time (days)

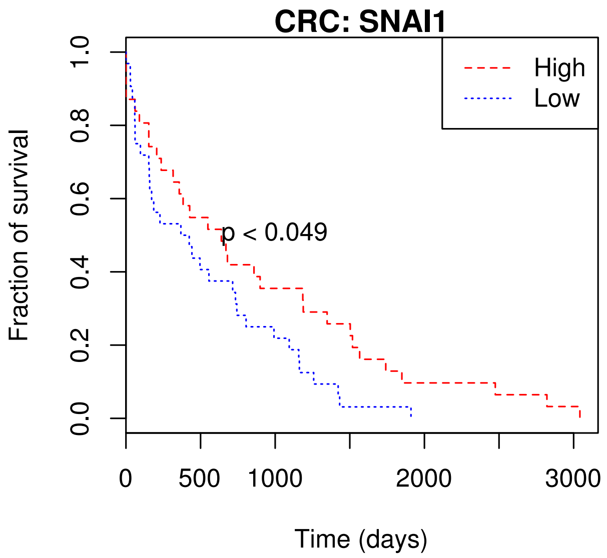
CRC: SNAI1
High
Low
p < 0.049
Fraction of survival
0.0 0.2 0.4 0.6 0.8 1.0
0 500 1000 2000 3000
Time (days)

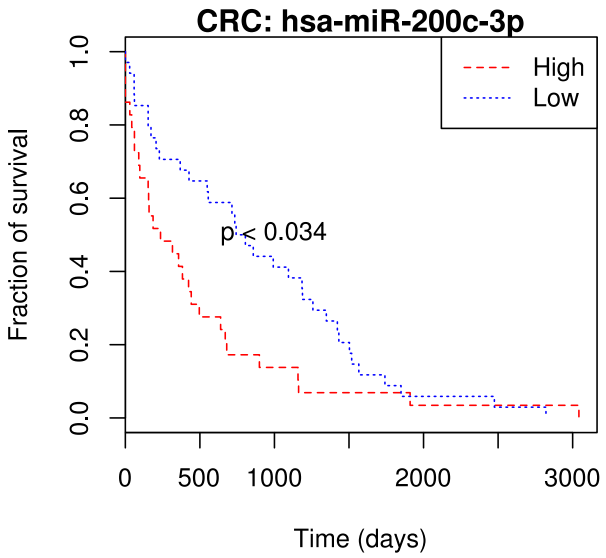
CRC: hsa-miR-200c-3p
High
Low
p < 0.034
Fraction of survival
0.0 0.2 0.4 0.6 0.8 1.0
0 500 1000 2000 3000
Time (days)

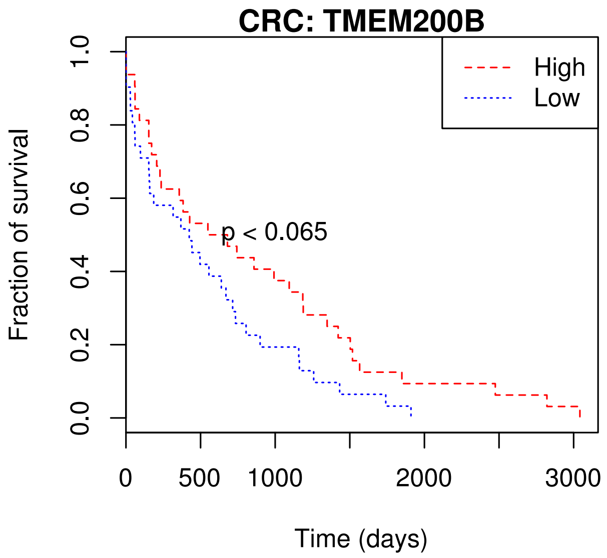
CRC: TMEM200B
High
Low
p < 0.065
Fraction of survival
0.0 0.2 0.4 0.6 0.8 1.0
0 500 1000 2000 3000
Time (days)

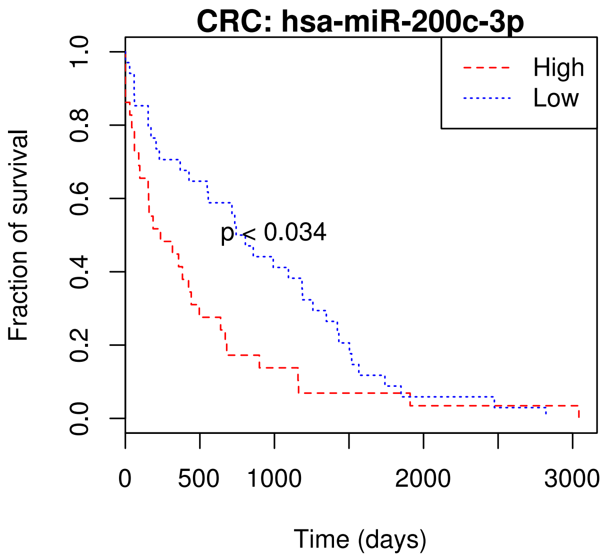
CRC: hsa-miR-200c-3p
High
Low
p < 0.034
Fraction of survival
0.0 0.2 0.4 0.6 0.8 1.0
0 500 1000 2000 3000
Time (days)

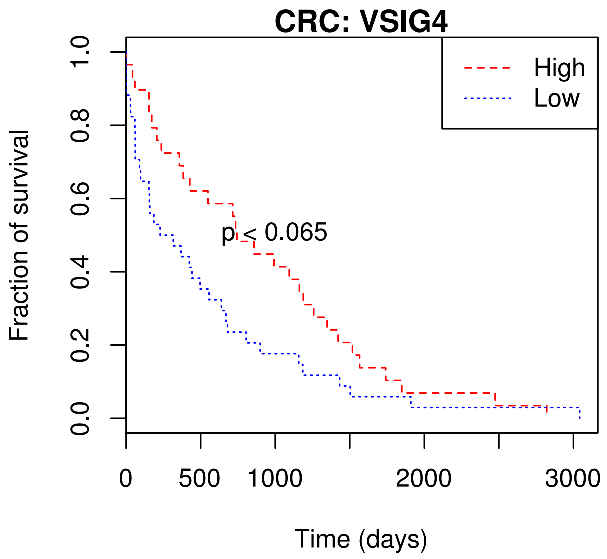
CRC: VSIG4
High
Low
p < 0.065
Fraction of survival
0.0 0.2 0.4 0.6 0.8 1.0
0 500 1000 2000 3000
Time (days)

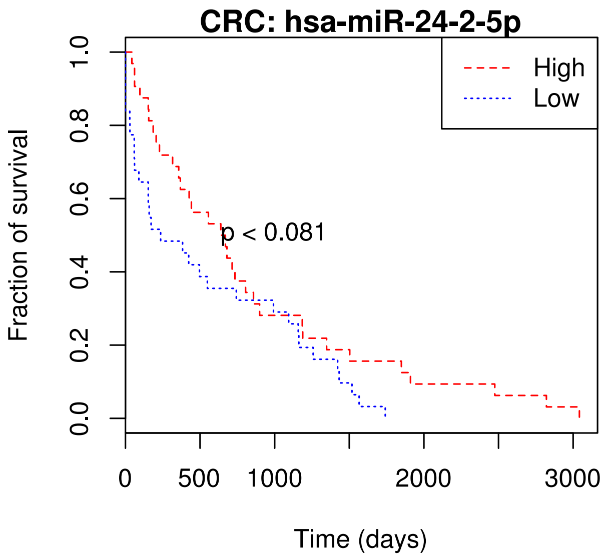

CRC: hsa-miR-24-2-5p
High
Low
p < 0.081
Fraction of survival
0.0 0.2 0.4 0.6 0.8 1.0
0 500 1000 2000 3000
Time (days)

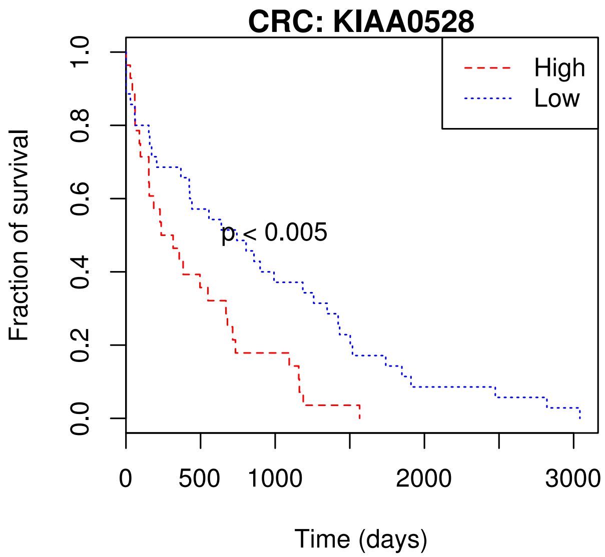

CRC: KIAA0528
High
Low
p < 0.005
Fraction of survival
0.0 0.2 0.4 0.6 0.8 1.0
0 500 1000 2000 3000
Time (days)

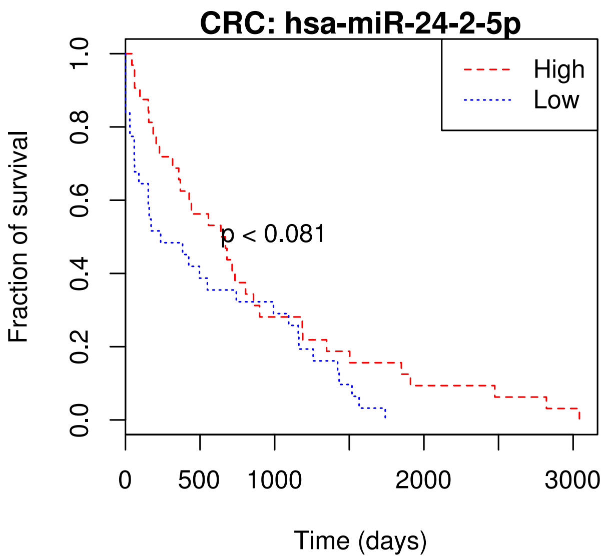

CRC: hsa-miR-24-2-5p
High
Low
p < 0.081
Fraction of survival
0.0 0.2 0.4 0.6 0.8 1.0
0 500 1000 2000 3000
Time (days)

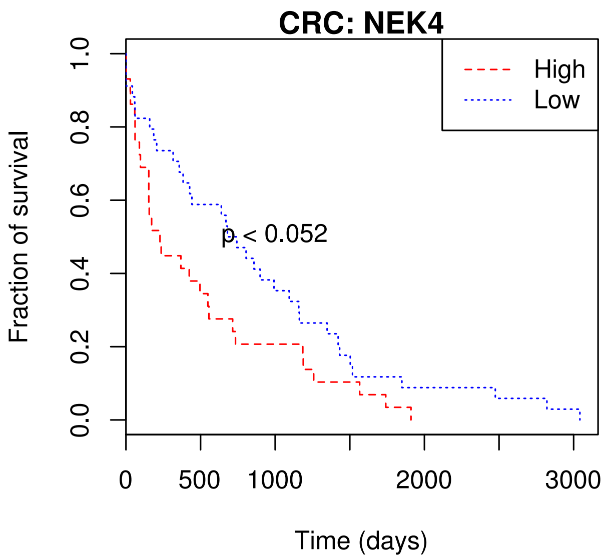

CRC: NEK4
High
Low
p < 0.052
Fraction of survival
0.0 0.2 0.4 0.6 0.8 1.0
0 500 1000 2000 3000
Time (days)

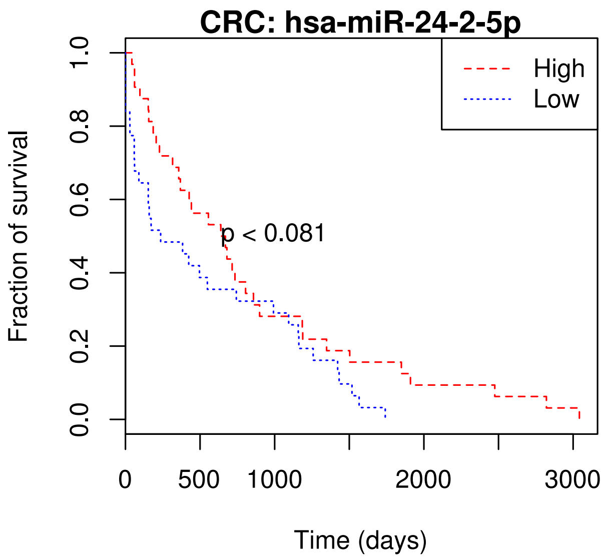

CRC: hsa-miR-24-2-5p
High
Low
p < 0.081
Fraction of survival
0.0 0.2 0.4 0.6 0.8 1.0
0 500 1000 2000 3000
Time (days)

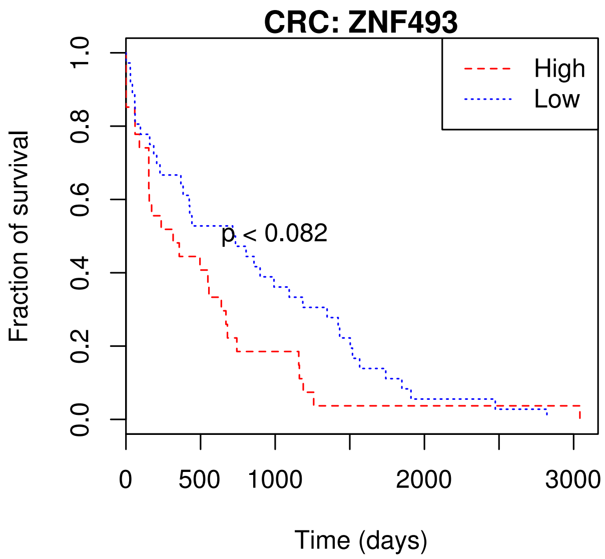

CRC: ZNF493
High
Low
p < 0.082
Fraction of survival
0.0 0.2 0.4 0.6 0.8 1.0
0 500 1000 2000 3000
Time (days)

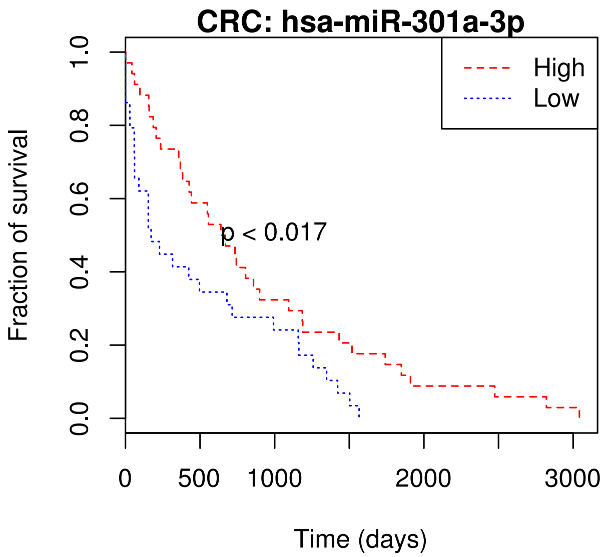
CRC: hsa-miR-301a-3p
High
Low
p < 0.017
Fraction of survival
Time (days)
0.0 0.2 0.4 0.6 0.8 1.0
0 500 1000 2000 3000

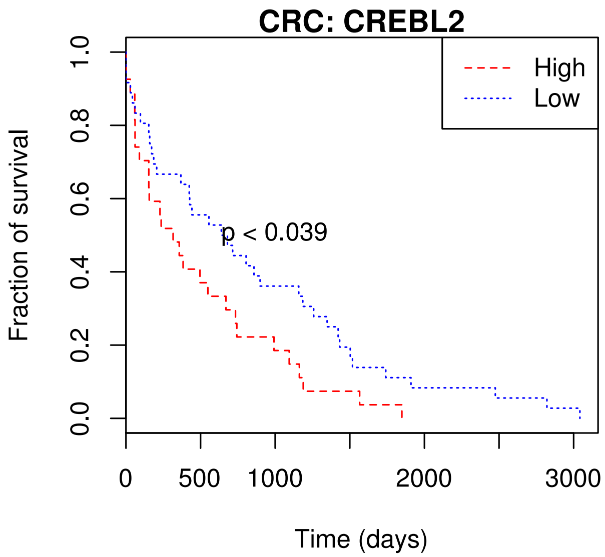
CRC: CREBL2
High
Low
p < 0.039
Fraction of survival
Time (days)
0.0 0.2 0.4 0.6 0.8 1.0
0 500 1000 2000 3000

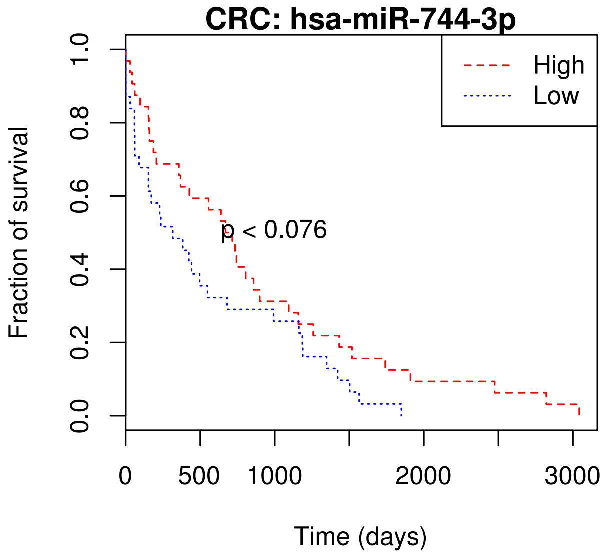
CRC: hsa-miR-744-3p
High
Low
p < 0.076
Fraction of survival
Time (days)
0.0 0.2 0.4 0.6 0.8 1.0
0 500 1000 2000 3000

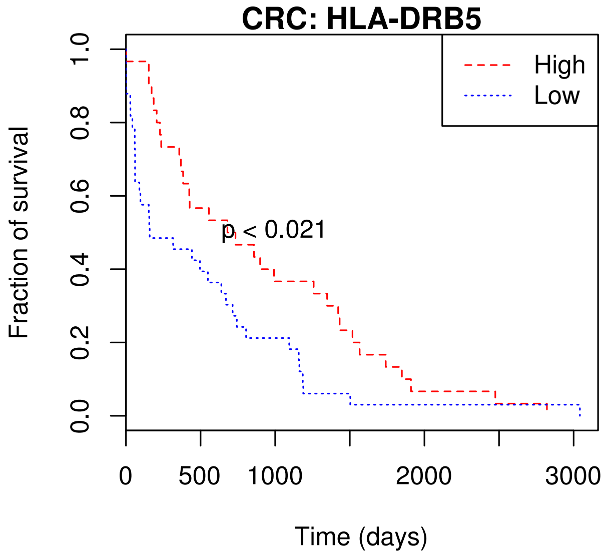
CRC: HLA-DRB5
High
Low
p < 0.021
Fraction of survival
Time (days)
0.0 0.2 0.4 0.6 0.8 1.0
0 500 1000 2000 3000

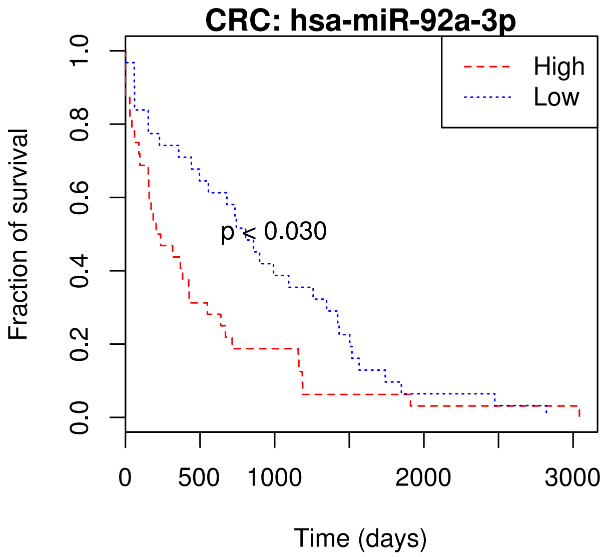
CRC: hsa-miR-92a-3p
High
Low
p < 0.030
Fraction of survival
Time (days)
0.0 0.2 0.4 0.6 0.8 1.0
0 500 1000 2000 3000

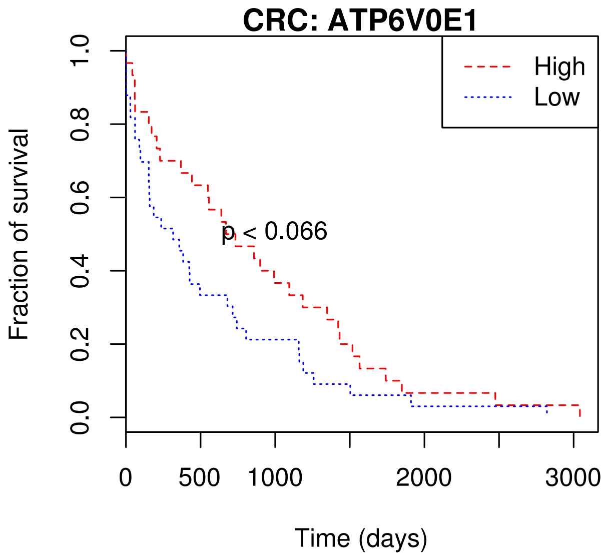
CRC: ATP6V0E1
High
Low
p < 0.066
Fraction of survival
Time (days)
0.0 0.2 0.4 0.6 0.8 1.0
0 500 1000 2000 3000

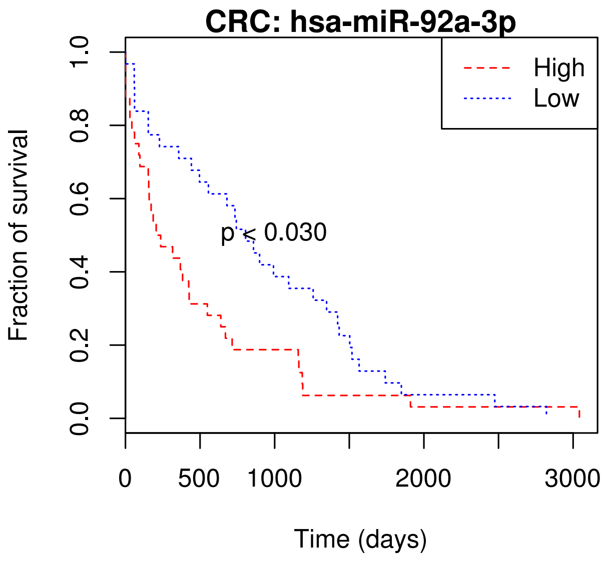
CRC: hsa-miR-92a-3p
High
Low
p < 0.030
Fraction of survival
Time (days)
0.0 0.2 0.4 0.6 0.8 1.0
0 500 1000 2000 3000

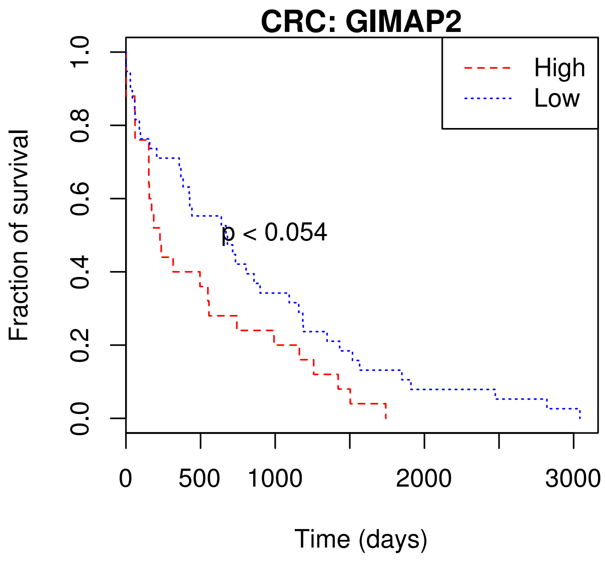
CRC: GIMAP2
High
Low
p < 0.054
Fraction of survival
Time (days)
0.0 0.2 0.4 0.6 0.8 1.0
0 500 1000 2000 3000

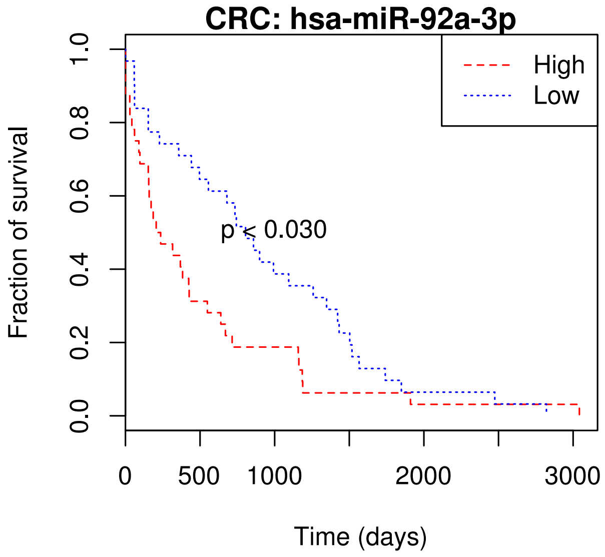
CRC: hsa-miR-92a-3p
High
Low
p < 0.030
Fraction of survival
Time (days)
0.0 0.2 0.4 0.6 0.8 1.0
0 500 1000 2000 3000

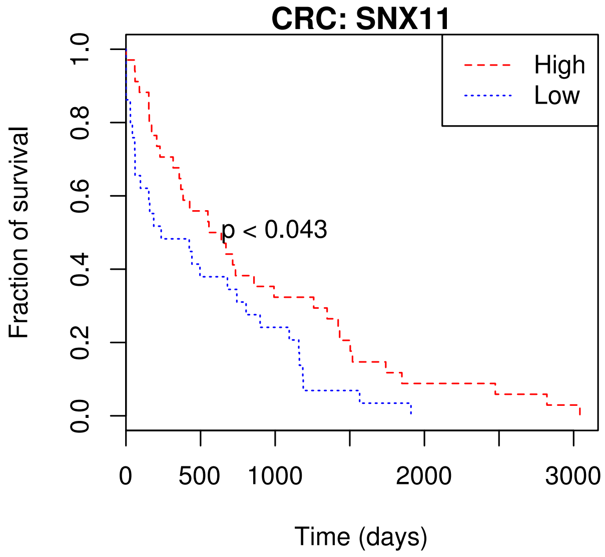
CRC: SNX11
High
Low
p < 0.043
Fraction of survival
Time (days)
0.0 0.2 0.4 0.6 0.8 1.0
0 500 1000 2000 3000

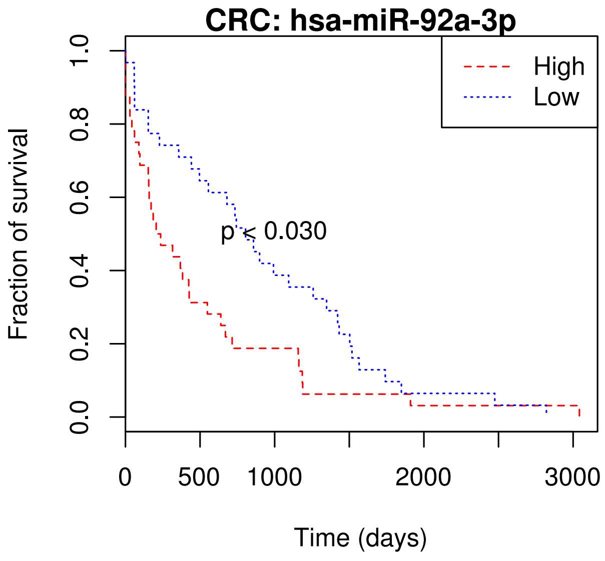
CRC: hsa-miR-92a-3p
High
Low
p < 0.030
Fraction of survival
Time (days)
0.0 0.2 0.4 0.6 0.8 1.0
0 500 1000 2000 3000

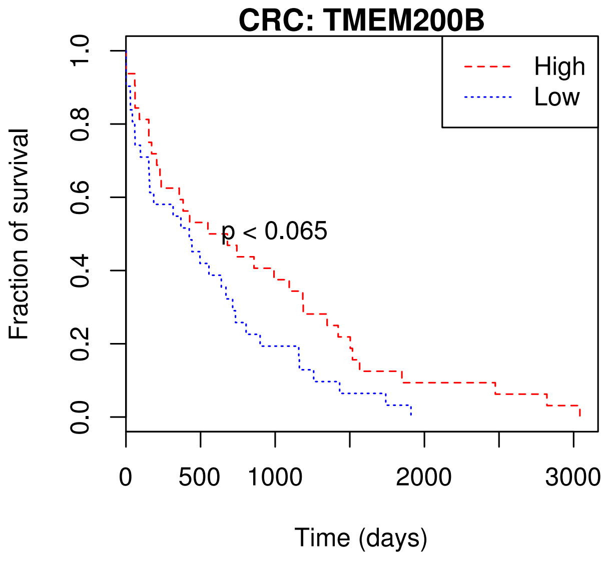
CRC: TMEM200B
High
Low
p < 0.065
Fraction of survival
Time (days)
0.0 0.2 0.4 0.6 0.8 1.0
0 500 1000 2000 3000

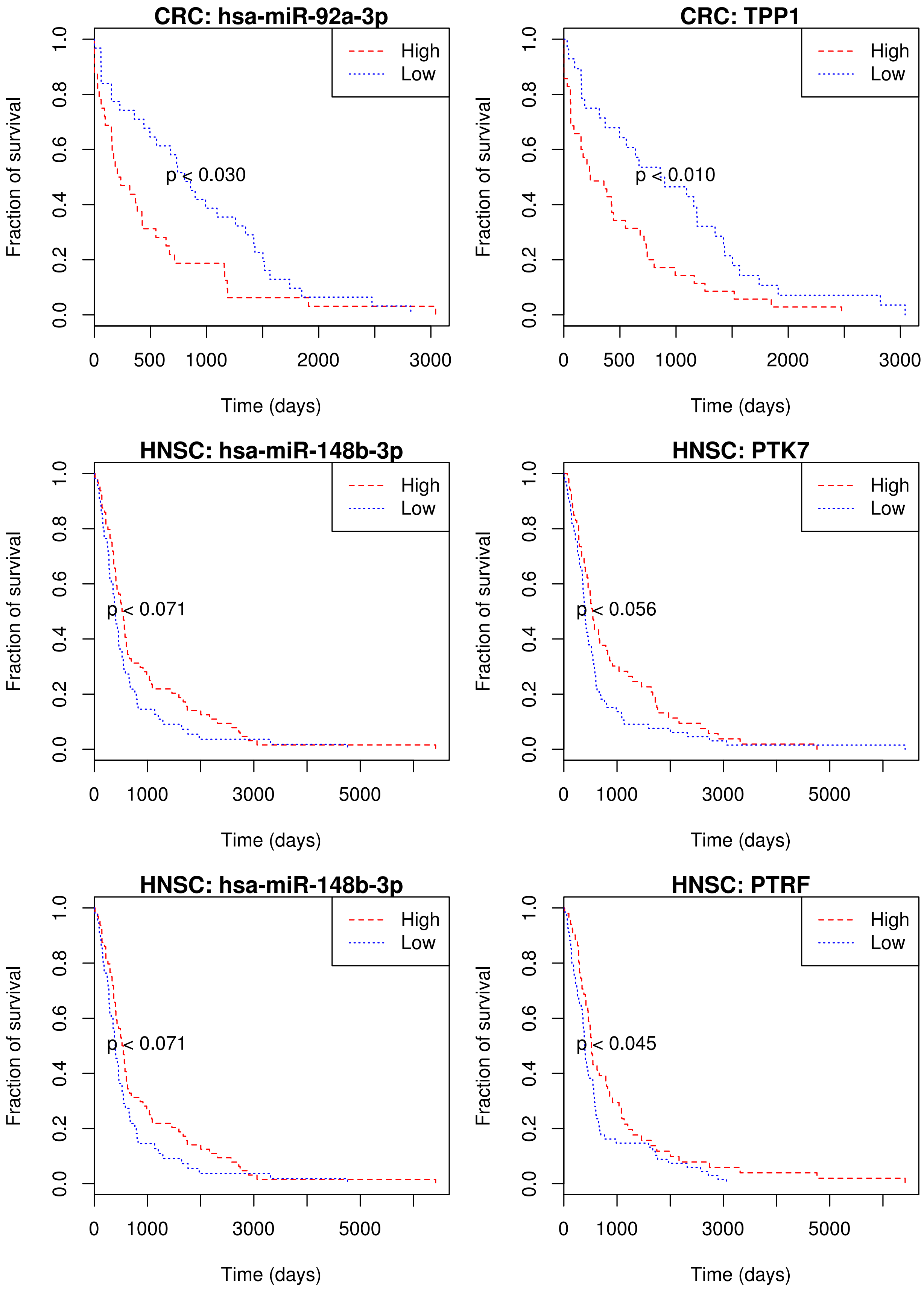
CRC: hsa-miR-92a-3p
High
Low
p < 0.030
Fraction of survival
Time (days)
CRC: TPP1
High
Low
p < 0.010
Fraction of survival
Time (days)
HNSC: hsa-miR-148b-3p
High
Low
p < 0.071
Fraction of survival
Time (days)
HNSC: PTK7
High
Low
p < 0.056
Fraction of survival
Time (days)
HNSC: hsa-miR-148b-3p
High
Low
p < 0.071
Fraction of survival
Time (days)
HNSC: PTRF
High
Low
p < 0.045
Fraction of survival
Time (days)

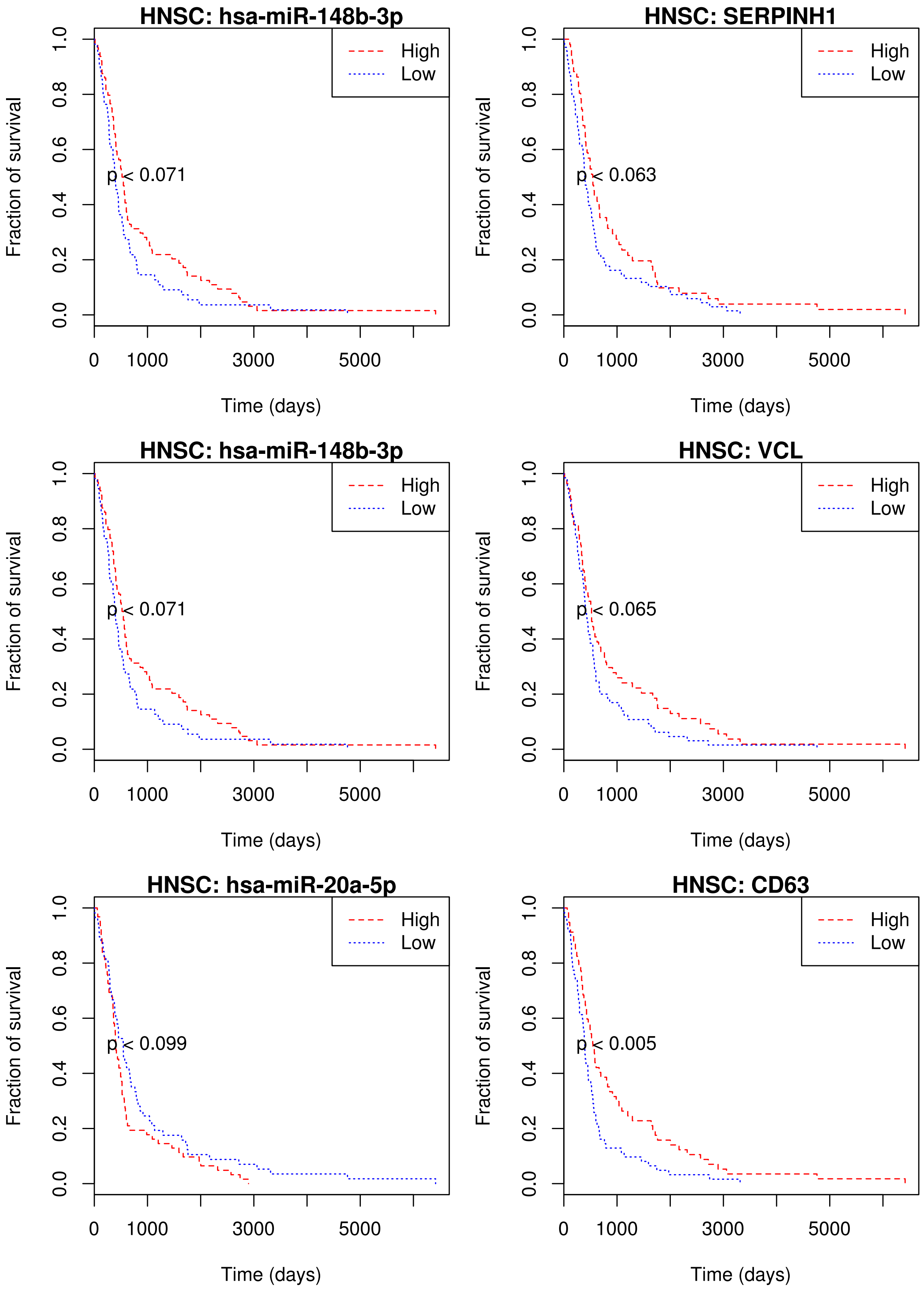
HNSC: hsa-miR-148b-3p
High
Low
p < 0.071
Fraction of survival
Time (days)
0.0
0.2
0.4
0.6
0.8
1.0
0
1000
3000
5000
HNSC: SERPINH1
High
Low
p < 0.063
Fraction of survival
Time (days)
HNSC: hsa-miR-148b-3p
High
Low
p < 0.071
Fraction of survival
Time (days)
HNSC: VCL
High
Low
p < 0.065
Fraction of survival
Time (days)
HNSC: hsa-miR-20a-5p
High
Low
p < 0.099
Fraction of survival
Time (days)
HNSC: CD63
High
Low
p < 0.005
Fraction of survival
Time (days)

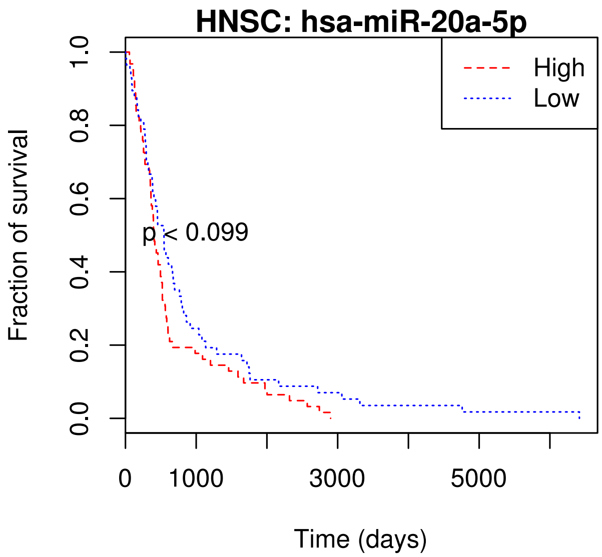
HNSC: hsa-miR-20a-5p
High
Low
p < 0.099
Fraction of survival
0.0 0.2 0.4 0.6 0.8 1.0
0 1000 3000 5000
Time (days)

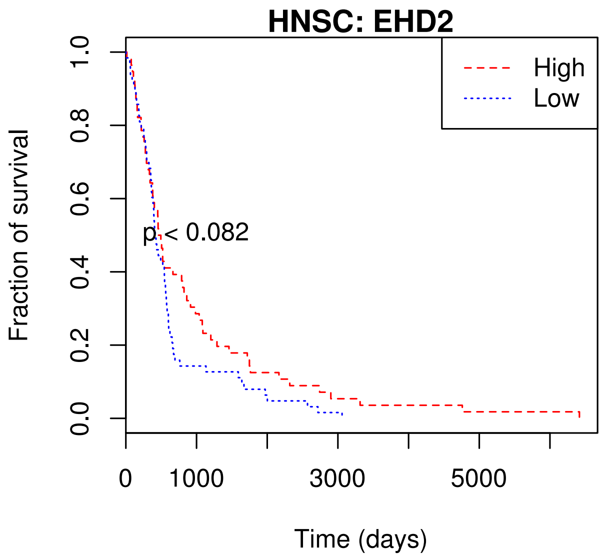
HNSC: EHD2
High
Low
p < 0.082
Fraction of survival
0.0 0.2 0.4 0.6 0.8 1.0
0 1000 3000 5000
Time (days)

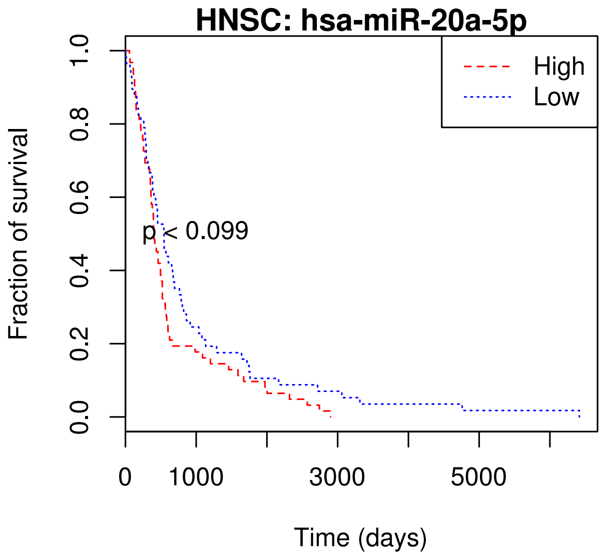
HNSC: hsa-miR-20a-5p
High
Low
p < 0.099
Fraction of survival
0.0 0.2 0.4 0.6 0.8 1.0
0 1000 3000 5000
Time (days)

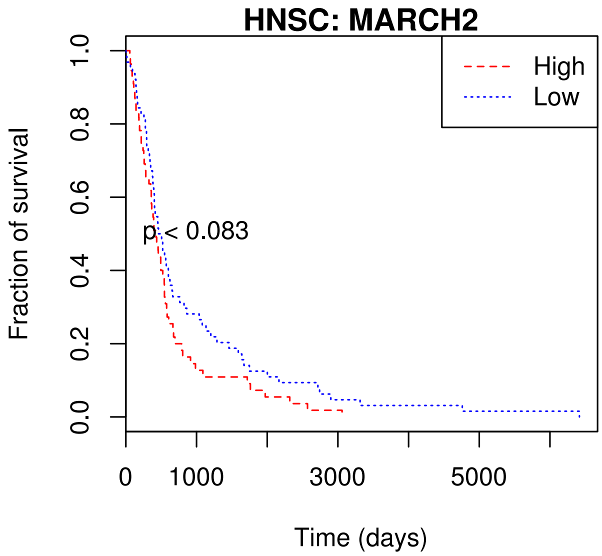
HNSC: MARCH2
High
Low
p < 0.083
Fraction of survival
0.0 0.2 0.4 0.6 0.8 1.0
0 1000 3000 5000
Time (days)

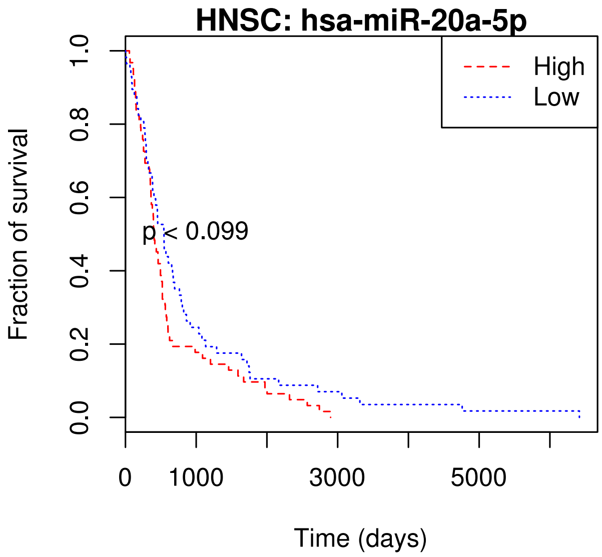
HNSC: hsa-miR-20a-5p
High
Low
p < 0.099
Fraction of survival
0.0 0.2 0.4 0.6 0.8 1.0
0 1000 3000 5000
Time (days)

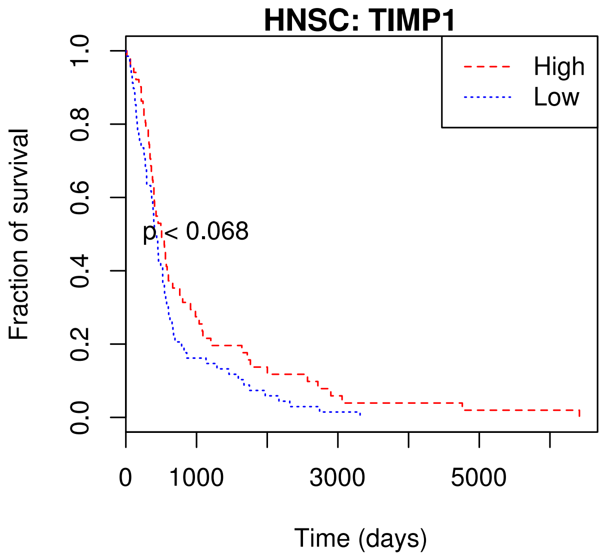
HNSC: TIMP1
High
Low
p < 0.068
Fraction of survival
0.0 0.2 0.4 0.6 0.8 1.0
0 1000 3000 5000
Time (days)

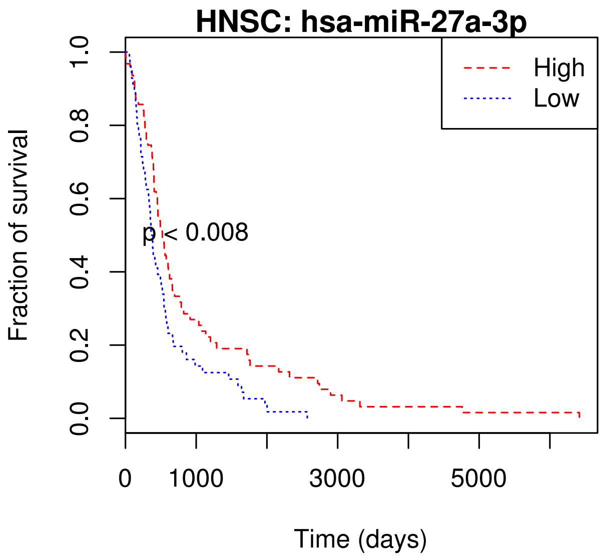
HNSC: hsa-miR-27a-3p
High
Low
p < 0.008
Fraction of survival
Time (days)
0.0 0.2 0.4 0.6 0.8 1.0
0 1000 3000 5000

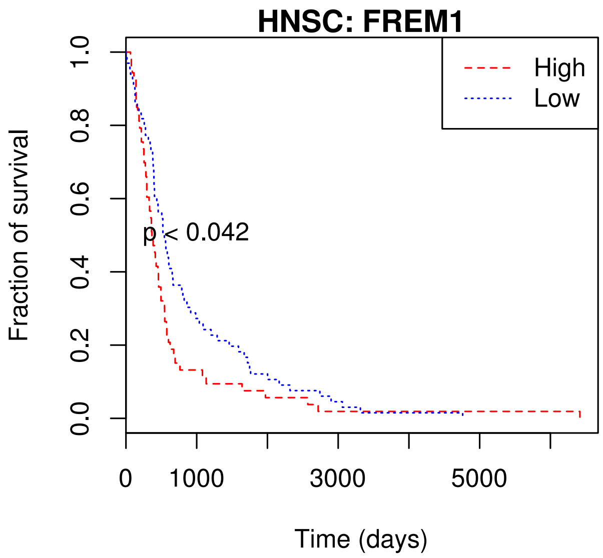
HNSC: FREM1
High
Low
p < 0.042
Fraction of survival
Time (days)
0.0 0.2 0.4 0.6 0.8 1.0
0 1000 3000 5000

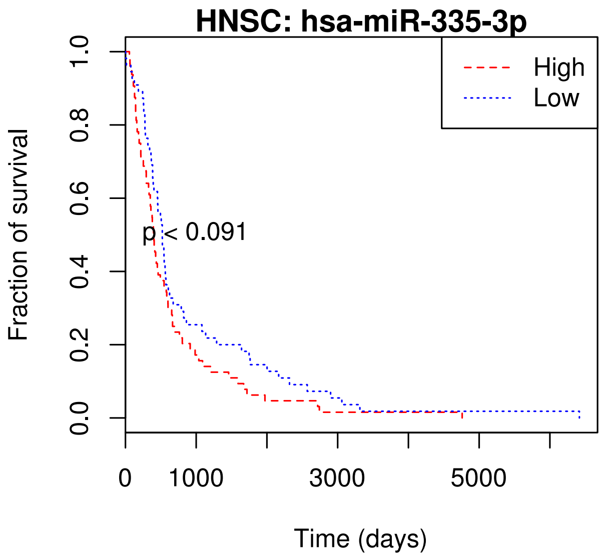
HNSC: hsa-miR-335-3p
High
Low
p < 0.091
Fraction of survival
Time (days)
0.0 0.2 0.4 0.6 0.8 1.0
0 1000 3000 5000

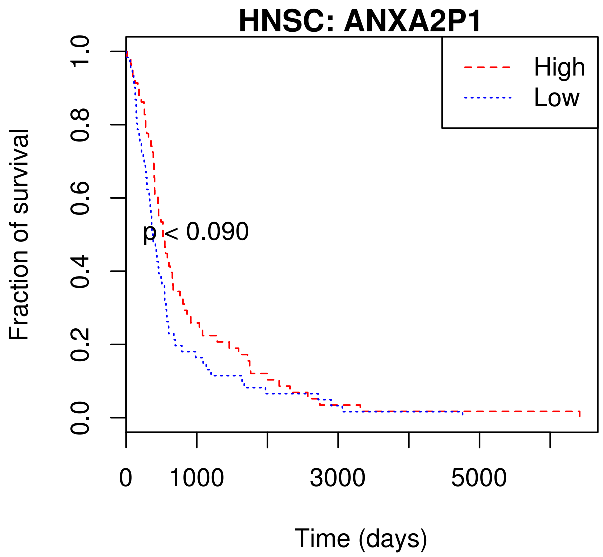
HNSC: ANXA2P1
High
Low
p < 0.090
Fraction of survival
Time (days)
0.0 0.2 0.4 0.6 0.8 1.0
0 1000 3000 5000

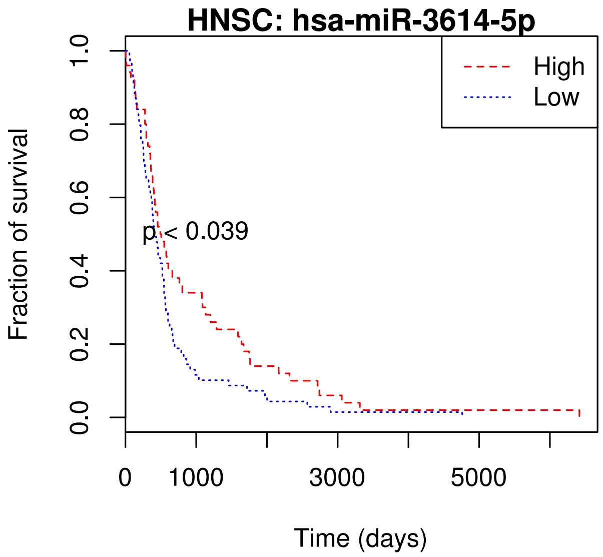
HNSC: hsa-miR-3614-5p
High
Low
p < 0.039
Fraction of survival
Time (days)
0.0 0.2 0.4 0.6 0.8 1.0
0 1000 3000 5000

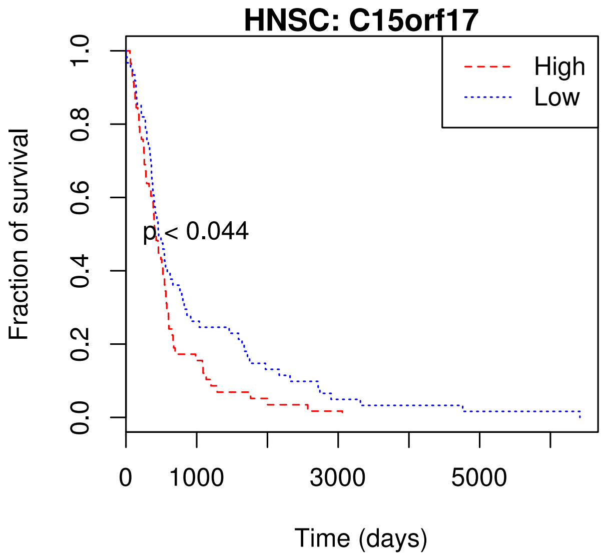
HNSC: C15orf17
High
Low
p < 0.044
Fraction of survival
Time (days)
0.0 0.2 0.4 0.6 0.8 1.0
0 1000 3000 5000

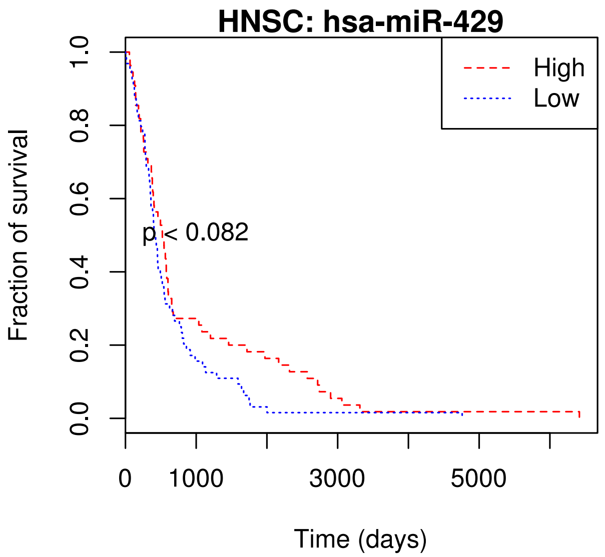
HNSC: hsa-miR-429
High
Low
p < 0.082
Fraction of survival
Time (days)
0.0 0.2 0.4 0.6 0.8 1.0
0 1000 3000 5000

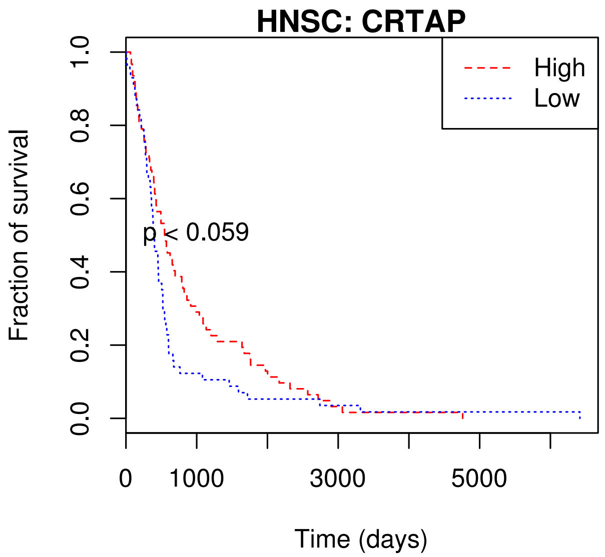
HNSC: CRTAP
High
Low
p < 0.059
Fraction of survival
Time (days)
0.0 0.2 0.4 0.6 0.8 1.0
0 1000 3000 5000

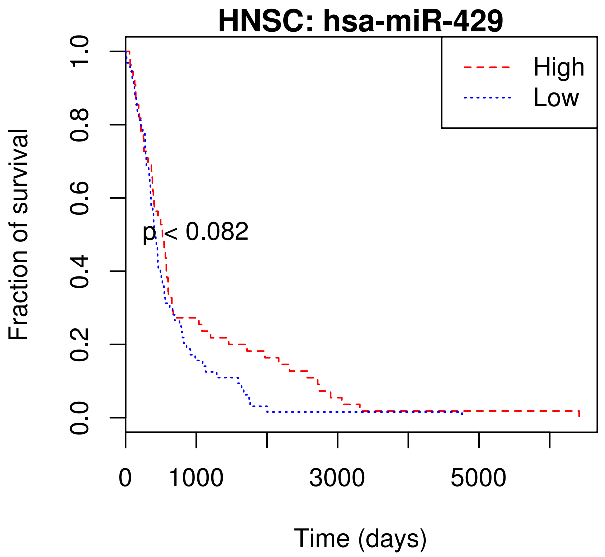
HNSC: hsa-miR-429
High
Low
p < 0.082
Fraction of survival
Time (days)
0.0 0.2 0.4 0.6 0.8 1.0
0 1000 3000 5000

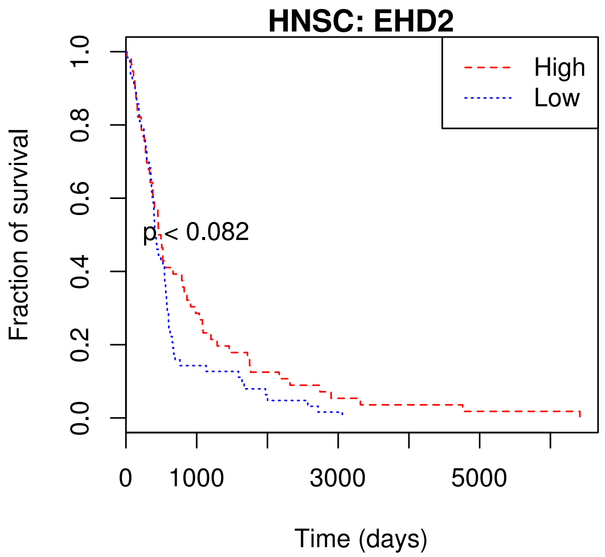
HNSC: EHD2
High
Low
p < 0.082
Fraction of survival
Time (days)
0.0 0.2 0.4 0.6 0.8 1.0
0 1000 3000 5000

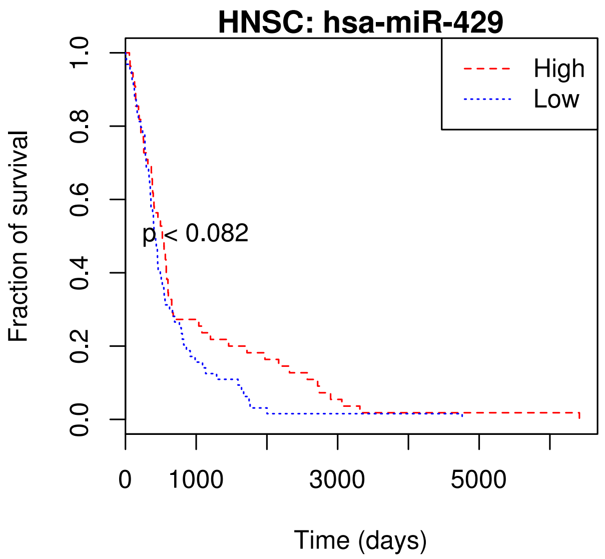
HNSC: hsa-miR-429
High
Low
p < 0.082
Fraction of survival
Time (days)
0.0 0.2 0.4 0.6 0.8 1.0
0 1000 3000 5000

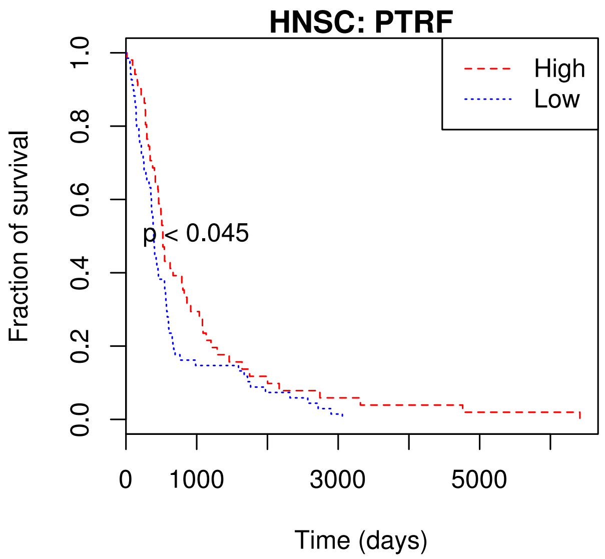
HNSC: PTRF
High
Low
p < 0.045
Fraction of survival
Time (days)
0.0 0.2 0.4 0.6 0.8 1.0
0 1000 3000 5000

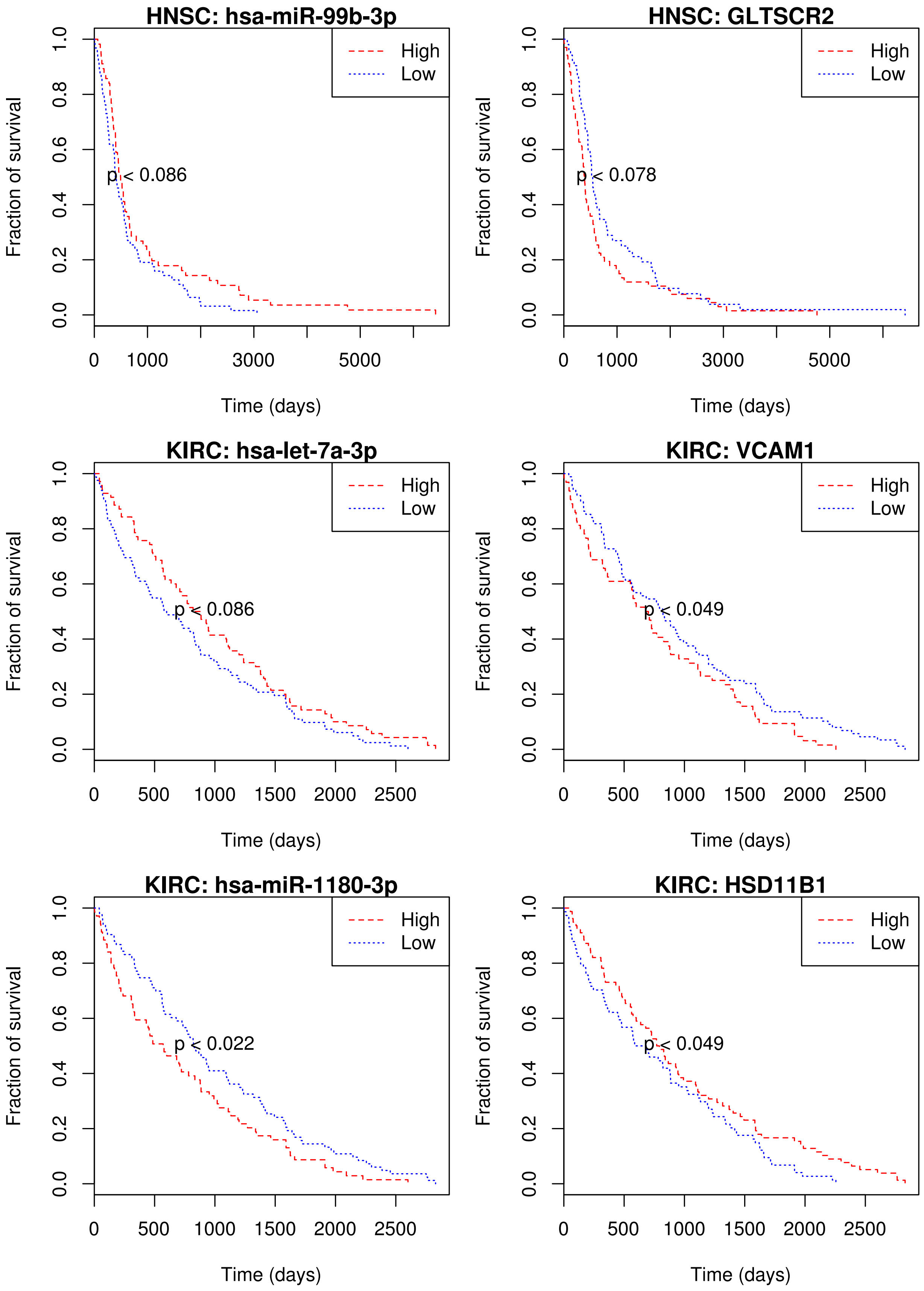
HNSC: hsa-miR-99b-3p
Fraction of survival
Time (days)
High
Low
p < 0.086
HNSC: GLTSCR2
Fraction of survival
Time (days)
High
Low
p < 0.078
KIRC: hsa-let-7a-3p
Fraction of survival
Time (days)
High
Low
p < 0.086
KIRC: VCAM1
Fraction of survival
Time (days)
High
Low
p < 0.049
KIRC: hsa-miR-1180-3p
Fraction of survival
Time (days)
High
Low
p < 0.022
KIRC: HSD11B1
Fraction of survival
Time (days)
High
Low
p < 0.049

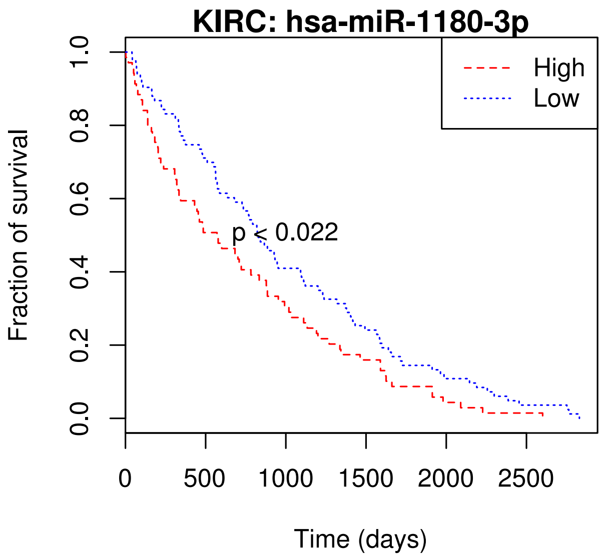
KIRC: hsa-miR-1180-3p
High
Low
p < 0.022
Fraction of survival
Time (days)
0.0 0.2 0.4 0.6 0.8 1.0
0 500 1000 1500 2000 2500

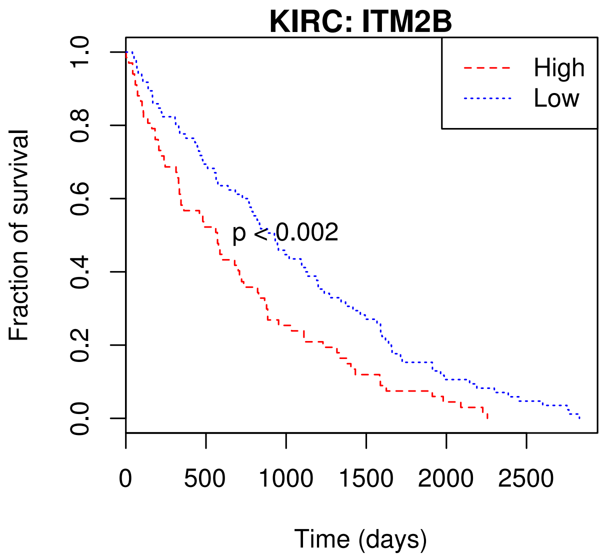
KIRC: ITM2B
High
Low
p < 0.002
Fraction of survival
Time (days)
0.0 0.2 0.4 0.6 0.8 1.0
0 500 1000 1500 2000 2500

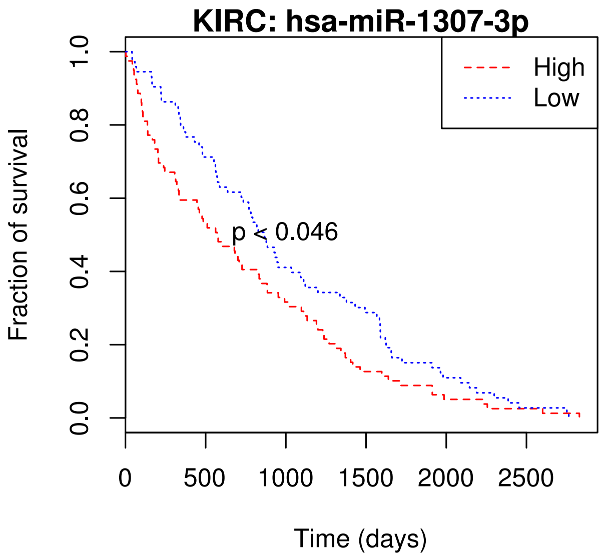
KIRC: hsa-miR-1307-3p
High
Low
p < 0.046
Fraction of survival
Time (days)
0.0 0.2 0.4 0.6 0.8 1.0
0 500 1000 1500 2000 2500

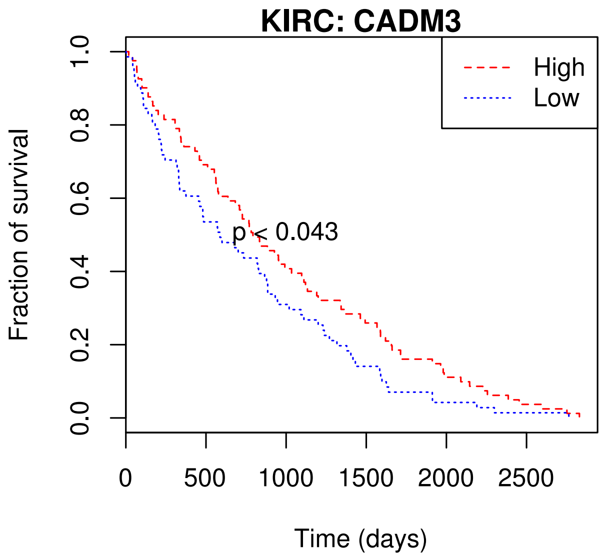
KIRC: CADM3
High
Low
p < 0.043
Fraction of survival
Time (days)
0.0 0.2 0.4 0.6 0.8 1.0
0 500 1000 1500 2000 2500

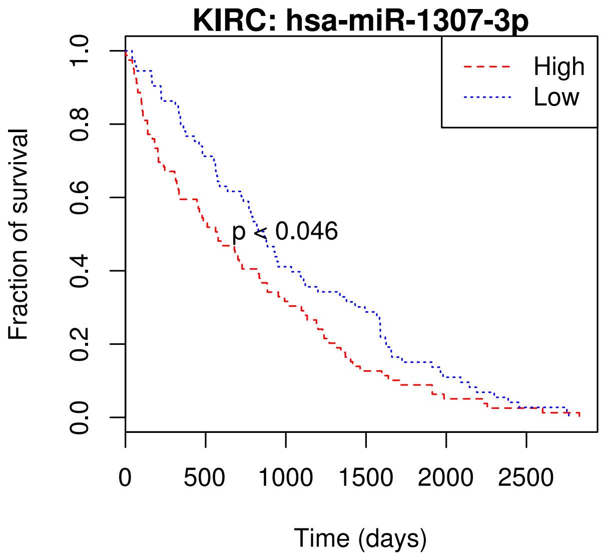
KIRC: hsa-miR-1307-3p
High
Low
p < 0.046
Fraction of survival
Time (days)
0.0 0.2 0.4 0.6 0.8 1.0
0 500 1000 1500 2000 2500

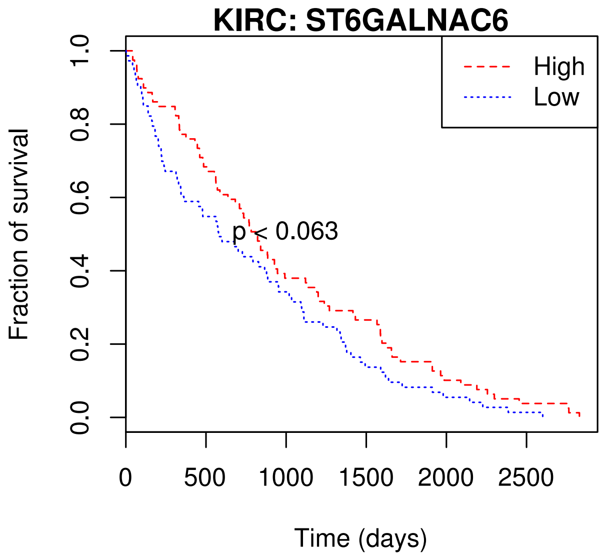
KIRC: ST6GALNAC6
High
Low
p < 0.063
Fraction of survival
Time (days)
0.0 0.2 0.4 0.6 0.8 1.0
0 500 1000 1500 2000 2500

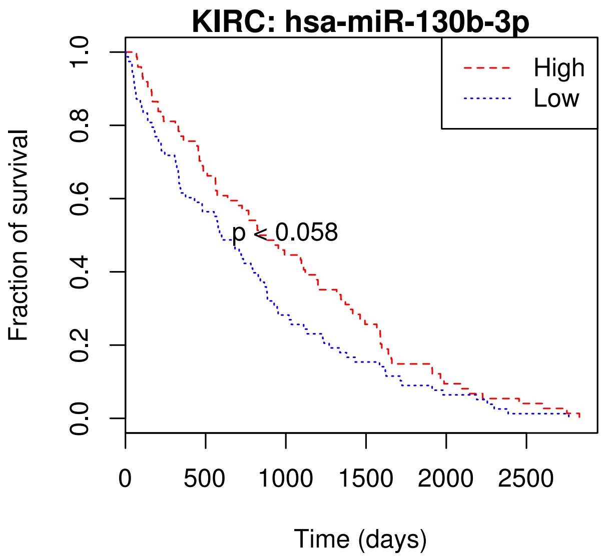
KIRC: hsa-miR-130b-3p
Fraction of survival
Time (days)
0.0
0.2
0.4
0.6
0.8
1.0
0
500
1000
1500
2000
2500
High
Low
p < 0.058

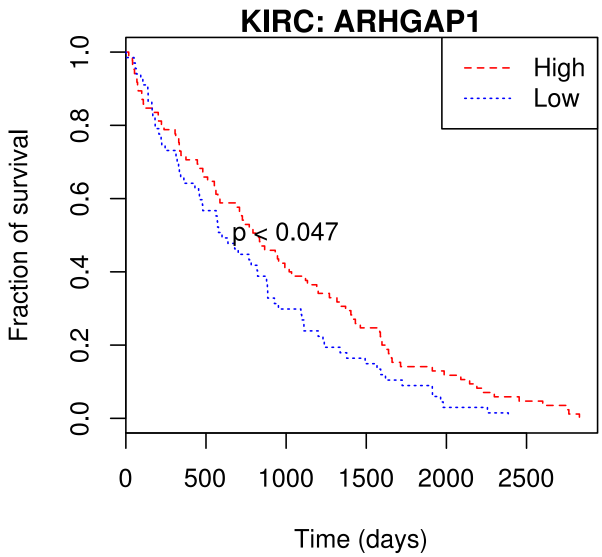
KIRC: ARHGAP1
Fraction of survival
Time (days)
0.0
0.2
0.4
0.6
0.8
1.0
0
500
1000
1500
2000
2500
High
Low
p < 0.047

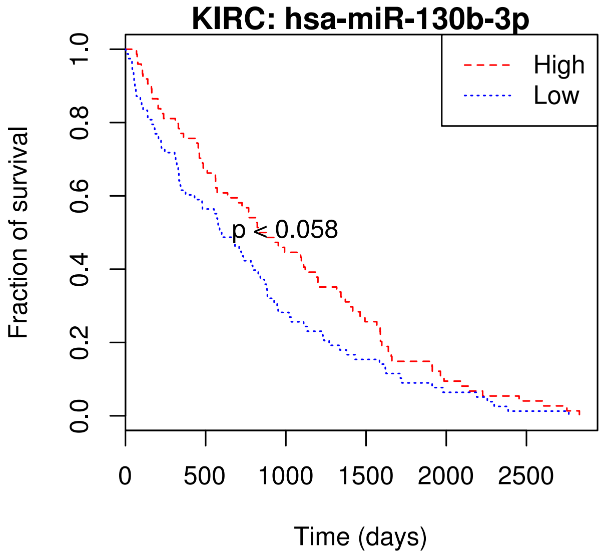
KIRC: hsa-miR-130b-3p
Fraction of survival
Time (days)
0.0
0.2
0.4
0.6
0.8
1.0
0
500
1000
1500
2000
2500
High
Low
p < 0.058

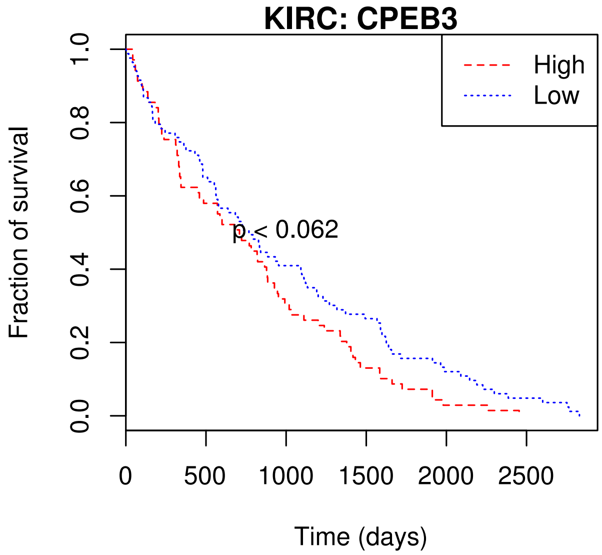
KIRC: CPEB3
Fraction of survival
Time (days)
0.0
0.2
0.4
0.6
0.8
1.0
0
500
1000
1500
2000
2500
High
Low
p < 0.062

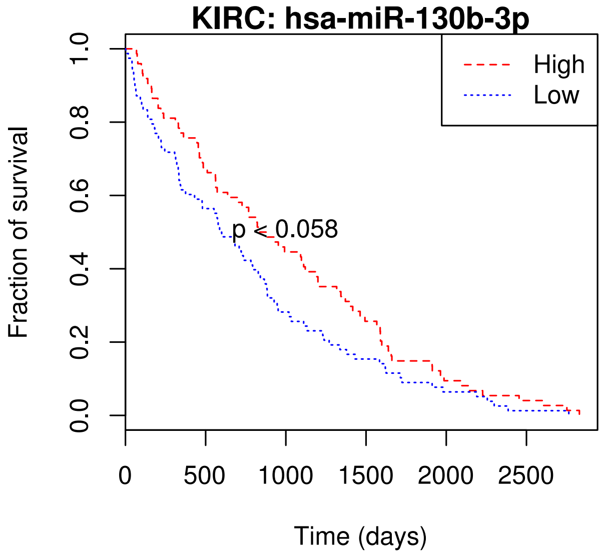
KIRC: hsa-miR-130b-3p
Fraction of survival
Time (days)
0.0
0.2
0.4
0.6
0.8
1.0
0
500
1000
1500
2000
2500
High
Low
p < 0.058

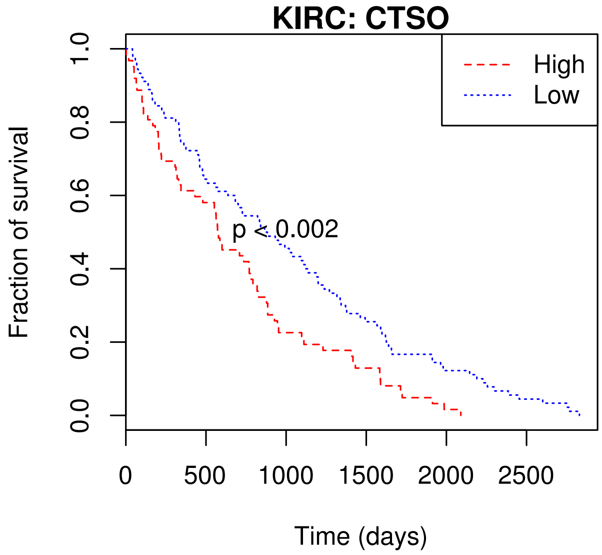
KIRC: CTSO
Fraction of survival
Time (days)
0.0
0.2
0.4
0.6
0.8
1.0
0
500
1000
1500
2000
2500
High
Low
p < 0.002

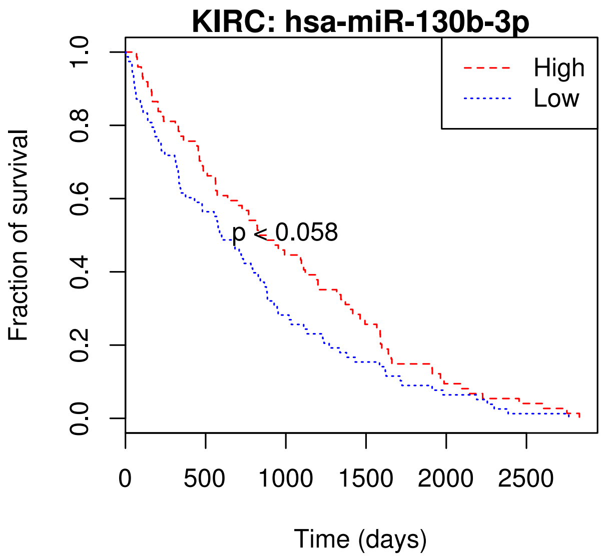
KIRC: hsa-miR-130b-3p
High
Low
p < 0.058
Fraction of survival
0.0
0.2
0.4
0.6
0.8
1.0
0
500
1000
1500
2000
2500
Time (days)

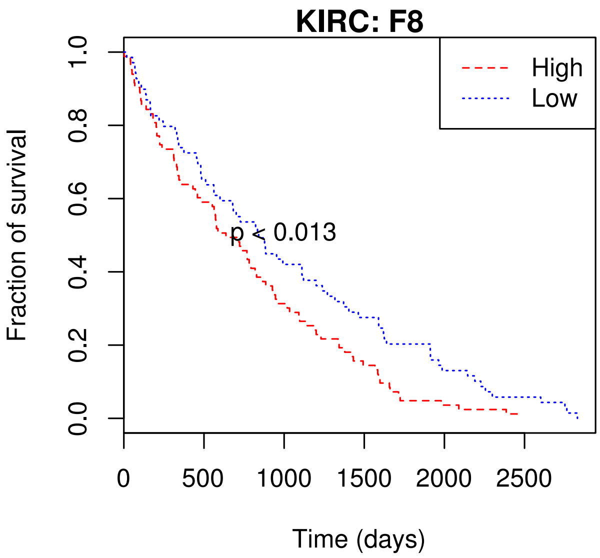
KIRC: F8
High
Low
p < 0.013
Fraction of survival
0.0
0.2
0.4
0.6
0.8
1.0
0
500
1000
1500
2000
2500
Time (days)

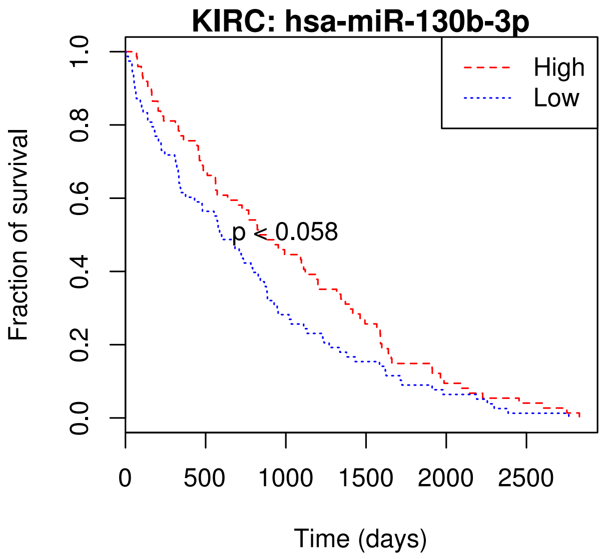
KIRC: hsa-miR-130b-3p
High
Low
p < 0.058
Fraction of survival
0.0
0.2
0.4
0.6
0.8
1.0
0
500
1000
1500
2000
2500
Time (days)

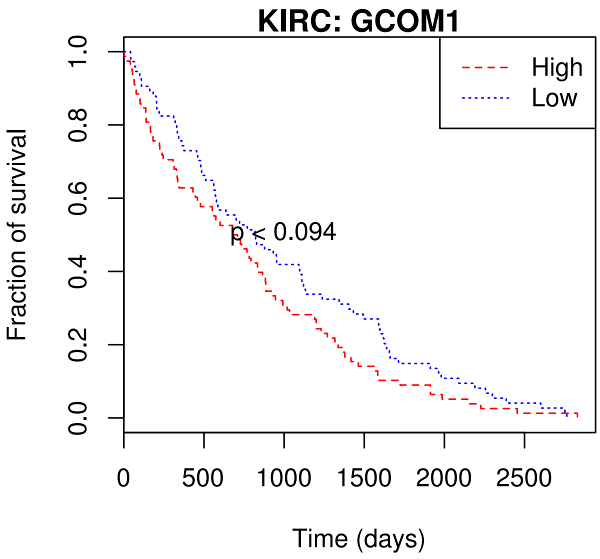
KIRC: GCOM1
High
Low
p < 0.094
Fraction of survival
0.0
0.2
0.4
0.6
0.8
1.0
0
500
1000
1500
2000
2500
Time (days)

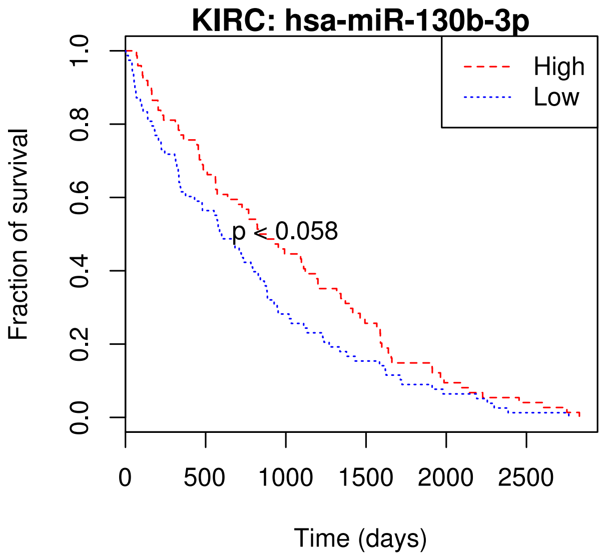
KIRC: hsa-miR-130b-3p
High
Low
p < 0.058
Fraction of survival
0.0
0.2
0.4
0.6
0.8
1.0
0
500
1000
1500
2000
2500
Time (days)

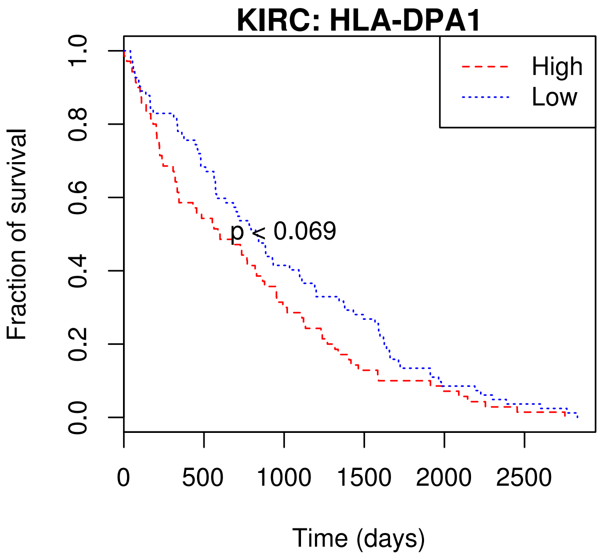
KIRC: HLA-DPA1
High
Low
p < 0.069
Fraction of survival
0.0
0.2
0.4
0.6
0.8
1.0
0
500
1000
1500
2000
2500
Time (days)

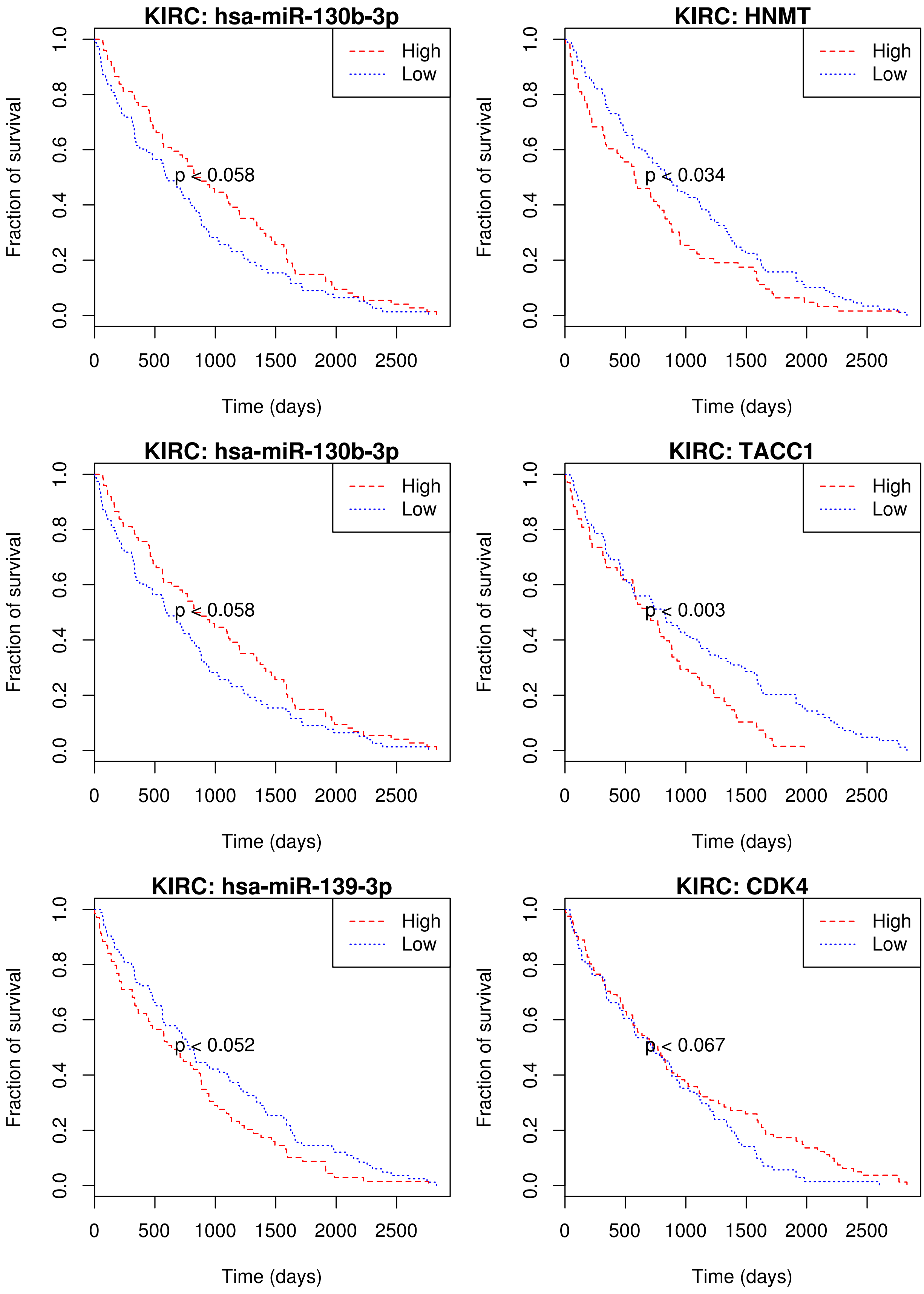
KIRC: hsa-miR-130b-3p
High
Low
p < 0.058
Fraction of survival
Time (days)
KIRC: HNMT
High
Low
p < 0.034
Fraction of survival
Time (days)
KIRC: hsa-miR-130b-3p
High
Low
p < 0.058
Fraction of survival
Time (days)
KIRC: TACC1
High
Low
p < 0.003
Fraction of survival
Time (days)
KIRC: hsa-miR-139-3p
High
Low
p < 0.052
Fraction of survival
Time (days)
KIRC: CDK4
High
Low
p < 0.067
Fraction of survival
Time (days)

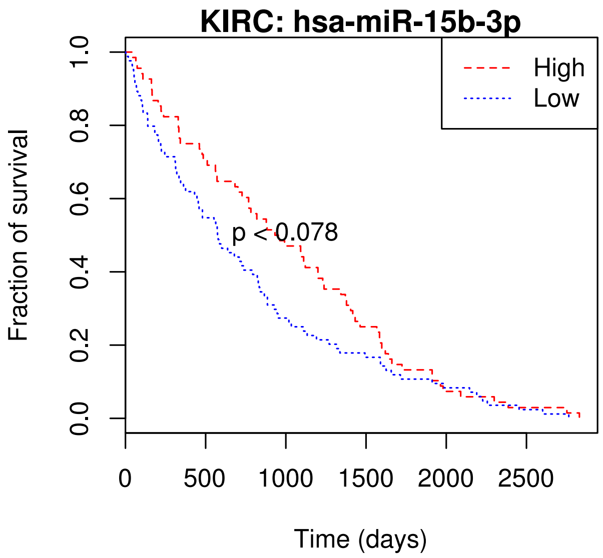
KIRC: hsa-miR-15b-3p
High
Low
p < 0.078
Fraction of survival
Time (days)
0.0 0.2 0.4 0.6 0.8 1.0
0 500 1000 1500 2000 2500

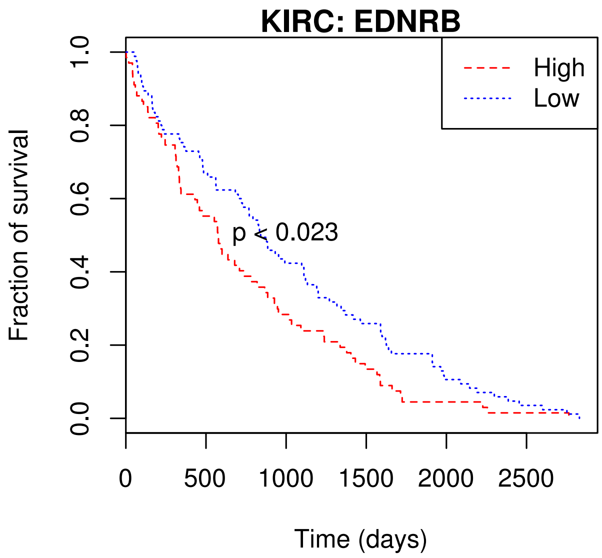
KIRC: EDNRB
High
Low
p < 0.023
Fraction of survival
Time (days)
0.0 0.2 0.4 0.6 0.8 1.0
0 500 1000 1500 2000 2500

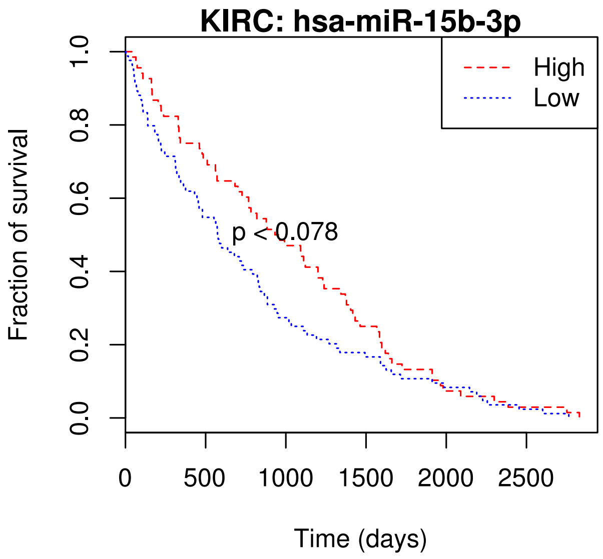
KIRC: hsa-miR-15b-3p
High
Low
p < 0.078
Fraction of survival
Time (days)
0.0 0.2 0.4 0.6 0.8 1.0
0 500 1000 1500 2000 2500

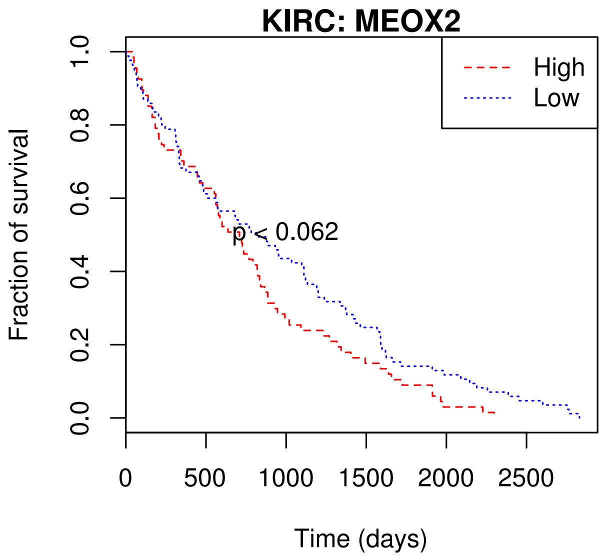
KIRC: MEOX2
High
Low
p < 0.062
Fraction of survival
Time (days)
0.0 0.2 0.4 0.6 0.8 1.0
0 500 1000 1500 2000 2500

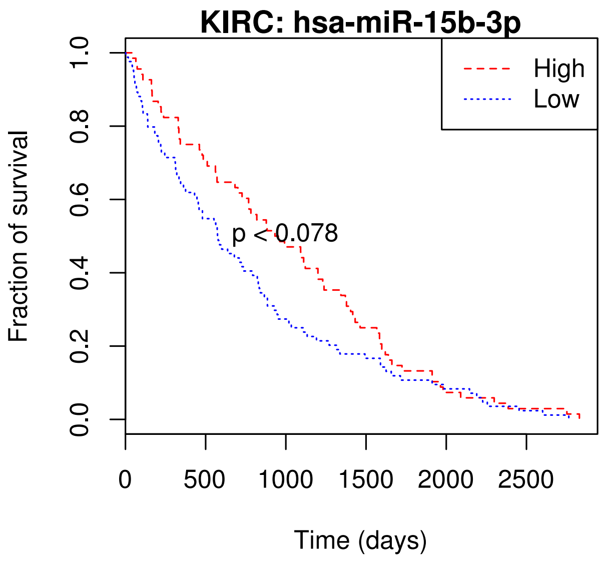
KIRC: hsa-miR-15b-3p
High
Low
p < 0.078
Fraction of survival
Time (days)
0.0 0.2 0.4 0.6 0.8 1.0
0 500 1000 1500 2000 2500

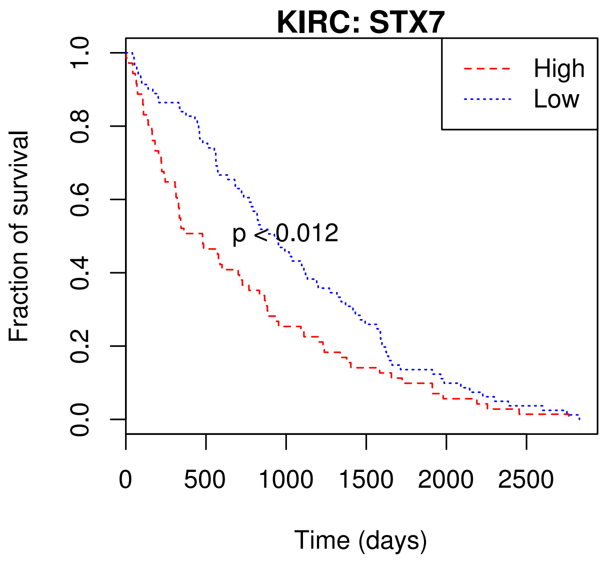
KIRC: STX7
High
Low
p < 0.012
Fraction of survival
Time (days)
0.0 0.2 0.4 0.6 0.8 1.0
0 500 1000 1500 2000 2500

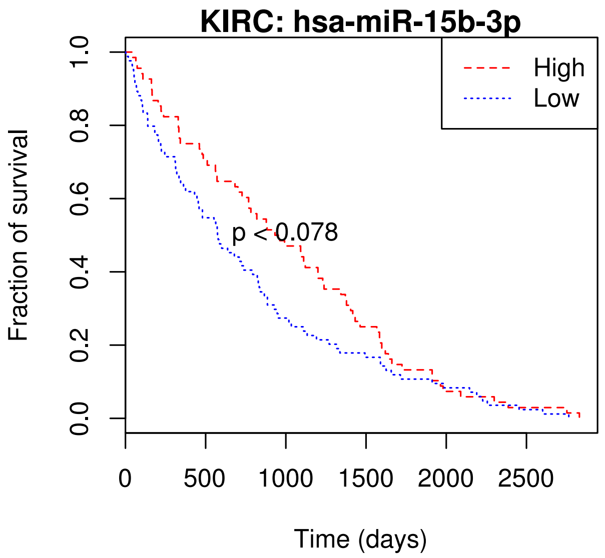
KIRC: hsa-miR-15b-3p
Fraction of survival
0.0
0.2
0.4
0.6
0.8
1.0
High
Low
p < 0.078
0
500
1000
1500
2000
2500
Time (days)

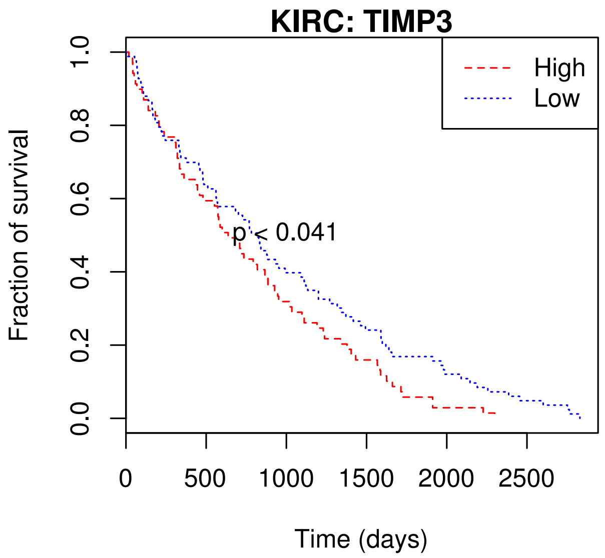
KIRC: TIMP3
Fraction of survival
0.0
0.2
0.4
0.6
0.8
1.0
High
Low
p < 0.041
0
500
1000
1500
2000
2500
Time (days)

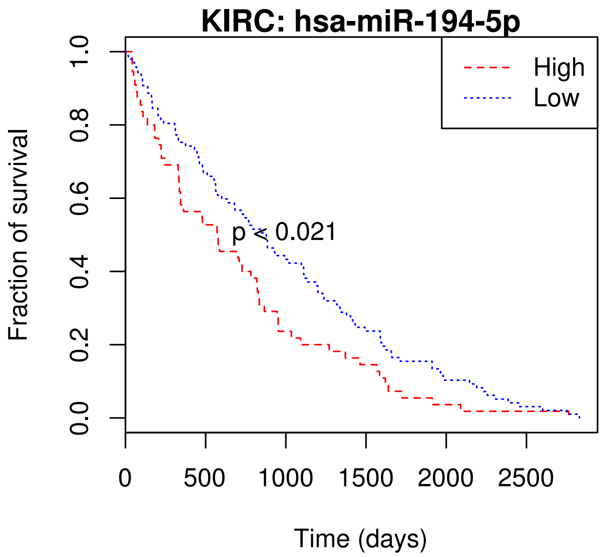
KIRC: hsa-miR-194-5p
Fraction of survival
0.0
0.2
0.4
0.6
0.8
1.0
High
Low
p < 0.021
0
500
1000
1500
2000
2500
Time (days)

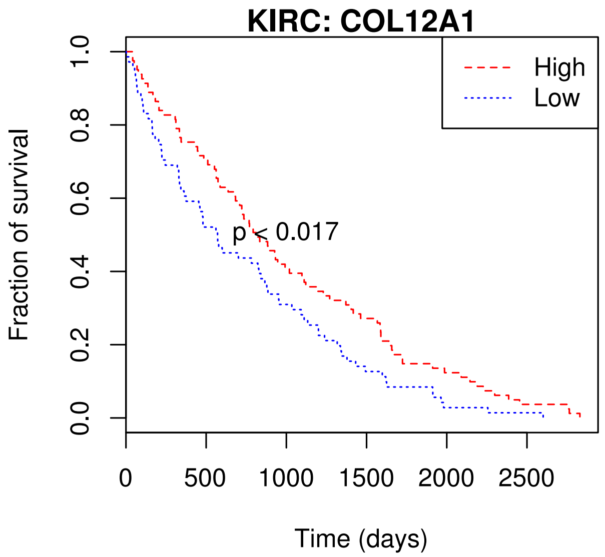
KIRC: COL12A1
Fraction of survival
0.0
0.2
0.4
0.6
0.8
1.0
High
Low
p < 0.017
0
500
1000
1500
2000
2500
Time (days)

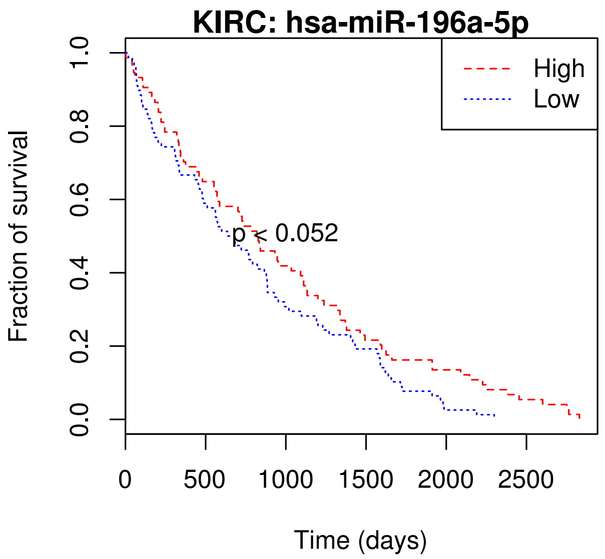
KIRC: hsa-miR-196a-5p
Fraction of survival
0.0
0.2
0.4
0.6
0.8
1.0
High
Low
p < 0.052
0
500
1000
1500
2000
2500
Time (days)

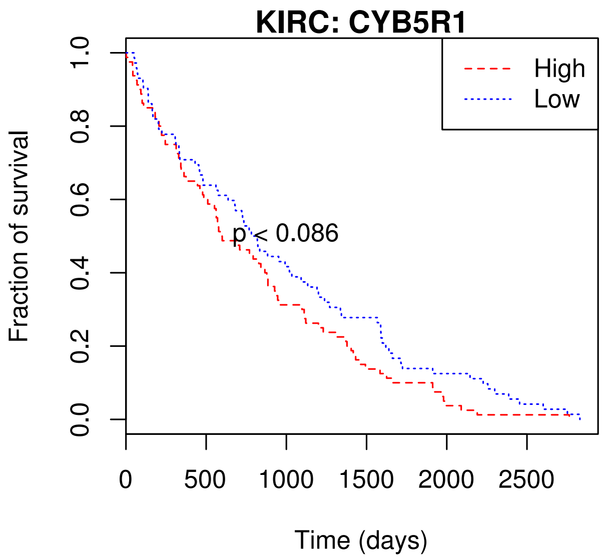
KIRC: CYB5R1
Fraction of survival
0.0
0.2
0.4
0.6
0.8
1.0
High
Low
p < 0.086
0
500
1000
1500
2000
2500
Time (days)

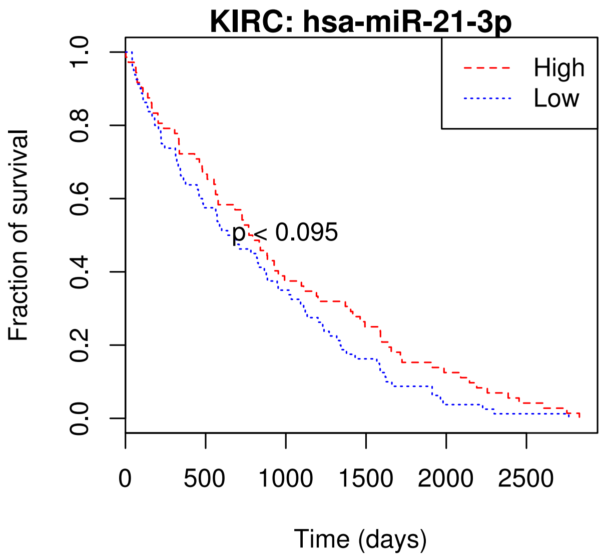
KIRC: hsa-miR-21-3p
High
Low
p < 0.095
Fraction of survival
Time (days)
0.0 0.2 0.4 0.6 0.8 1.0
0 500 1000 1500 2000 2500

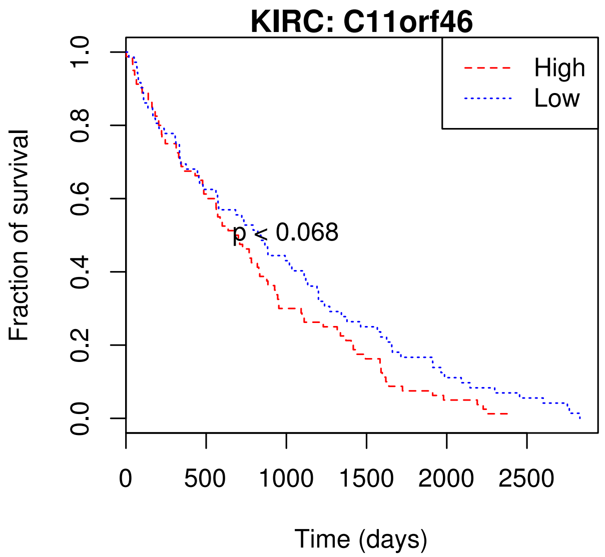
KIRC: C11orf46
High
Low
p < 0.068
Fraction of survival
Time (days)
0.0 0.2 0.4 0.6 0.8 1.0
0 500 1000 1500 2000 2500

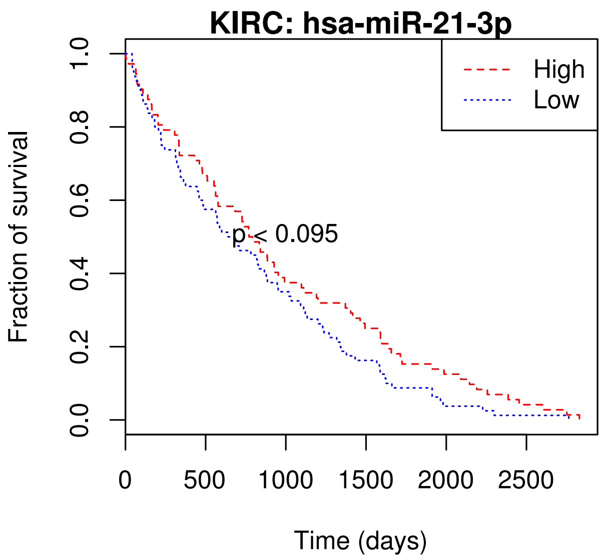
KIRC: hsa-miR-21-3p
High
Low
p < 0.095
Fraction of survival
Time (days)
0.0 0.2 0.4 0.6 0.8 1.0
0 500 1000 1500 2000 2500

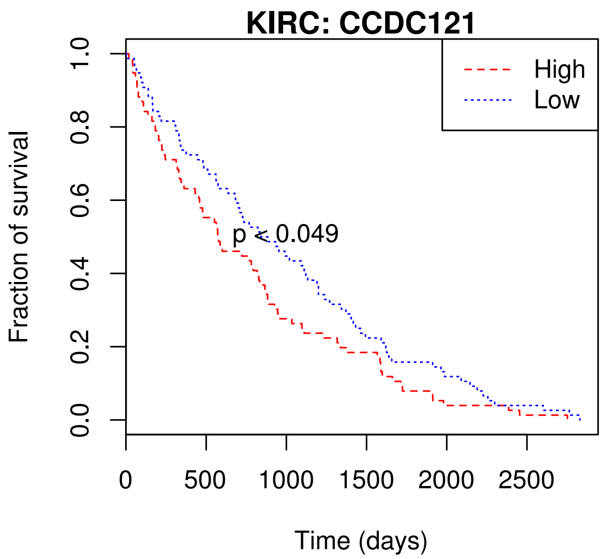
KIRC: CCDC121
High
Low
p < 0.049
Fraction of survival
Time (days)
0.0 0.2 0.4 0.6 0.8 1.0
0 500 1000 1500 2000 2500

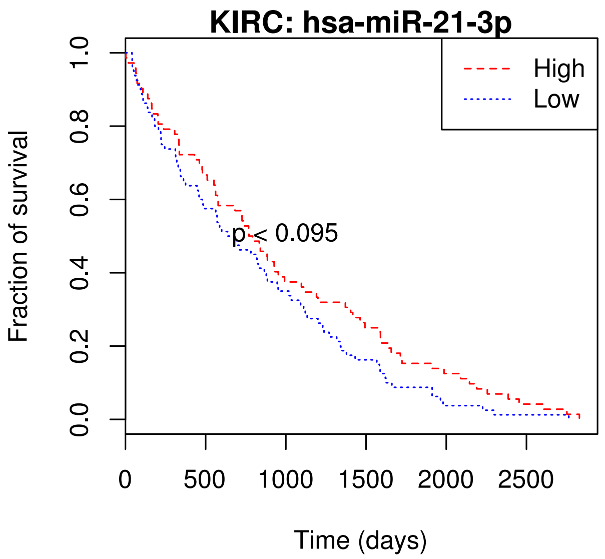
KIRC: hsa-miR-21-3p
High
Low
p < 0.095
Fraction of survival
Time (days)
0.0 0.2 0.4 0.6 0.8 1.0
0 500 1000 1500 2000 2500

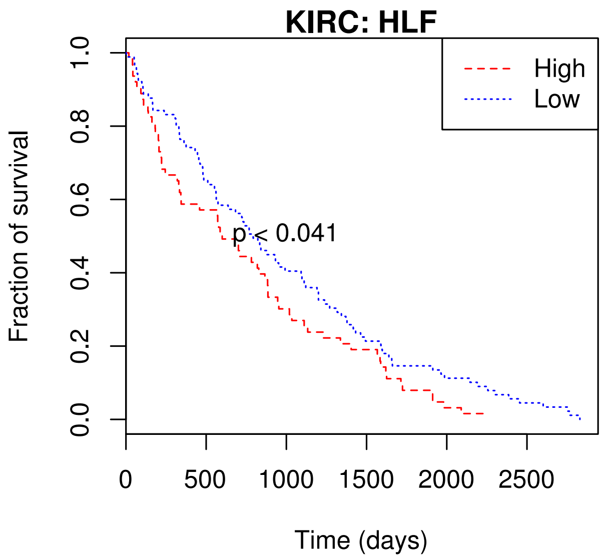
KIRC: HLF
High
Low
p < 0.041
Fraction of survival
Time (days)
0.0 0.2 0.4 0.6 0.8 1.0
0 500 1000 1500 2000 2500

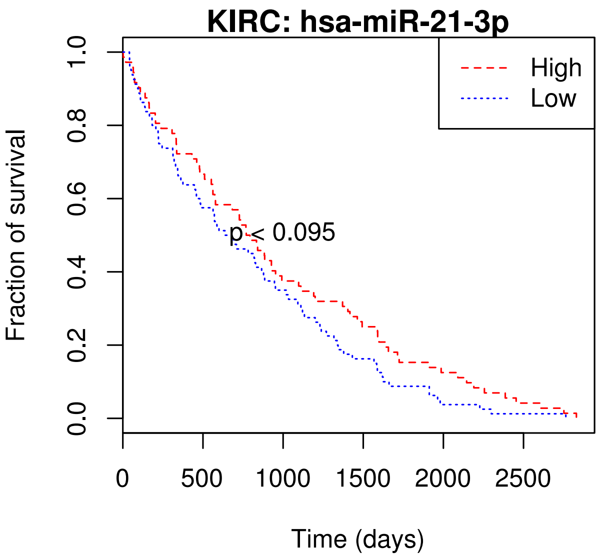
KIRC: hsa-miR-21-3p
High
Low
p < 0.095
Fraction of survival
Time (days)

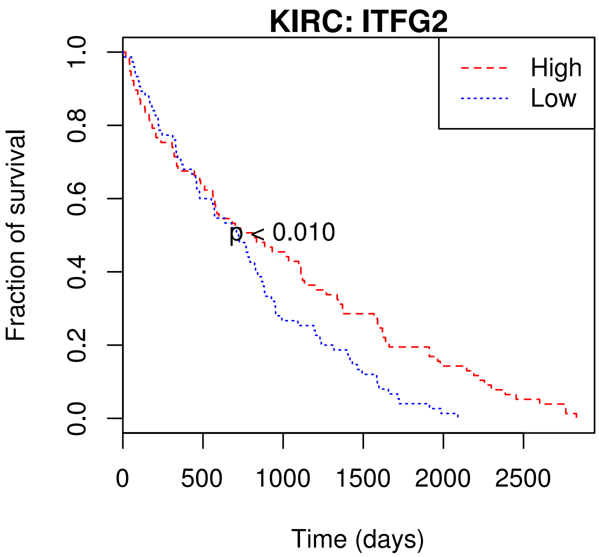
KIRC: ITFG2
High
Low
p < 0.010
Fraction of survival
Time (days)

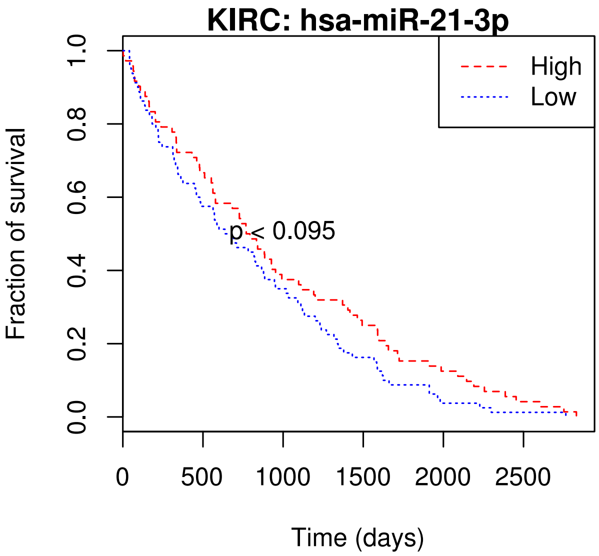
KIRC: hsa-miR-21-3p
High
Low
p < 0.095
Fraction of survival
Time (days)

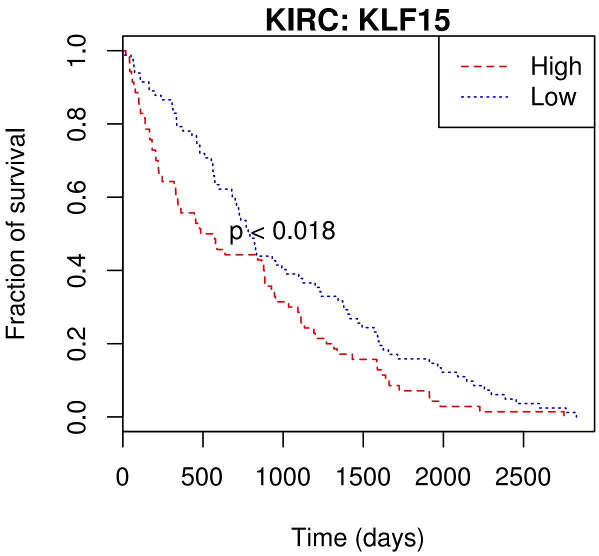
KIRC: KLF15
High
Low
p < 0.018
Fraction of survival
Time (days)

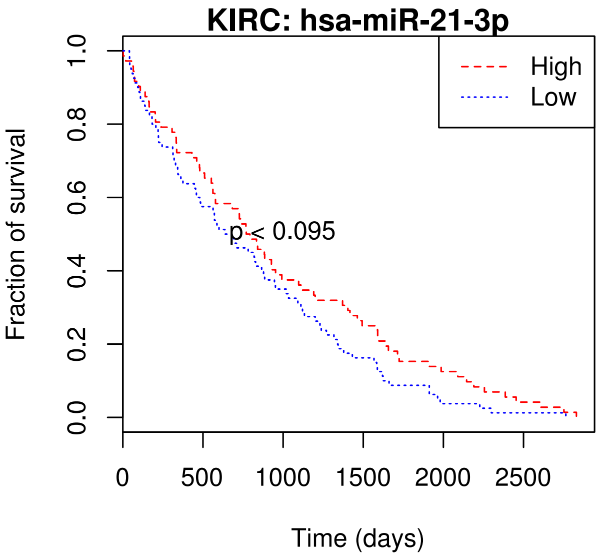
KIRC: hsa-miR-21-3p
High
Low
p < 0.095
Fraction of survival
Time (days)

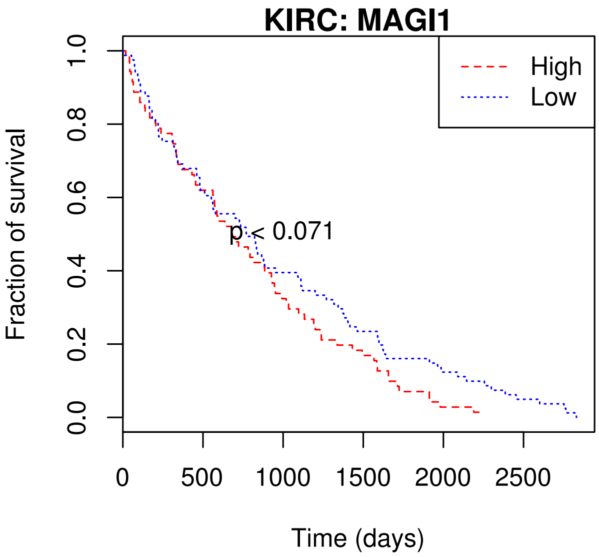
KIRC: MAGI1
High
Low
p < 0.071
Fraction of survival
Time (days)

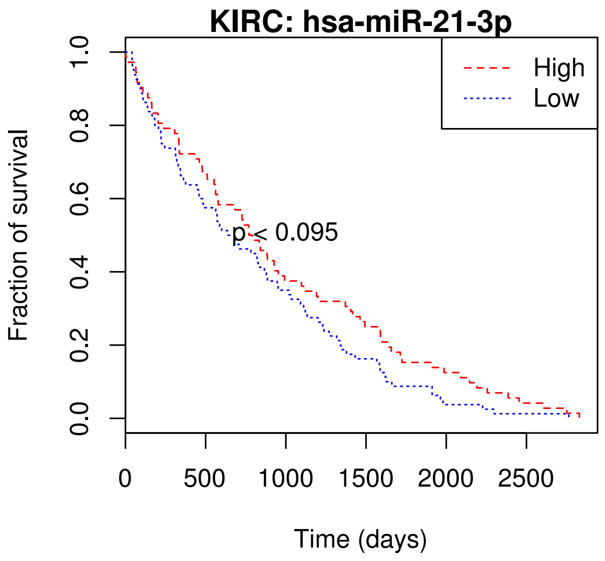
KIRC: hsa-miR-21-3p
High
Low
p < 0.095
Fraction of survival
Time (days)
0.0 0.2 0.4 0.6 0.8 1.0
0 500 1000 1500 2000 2500

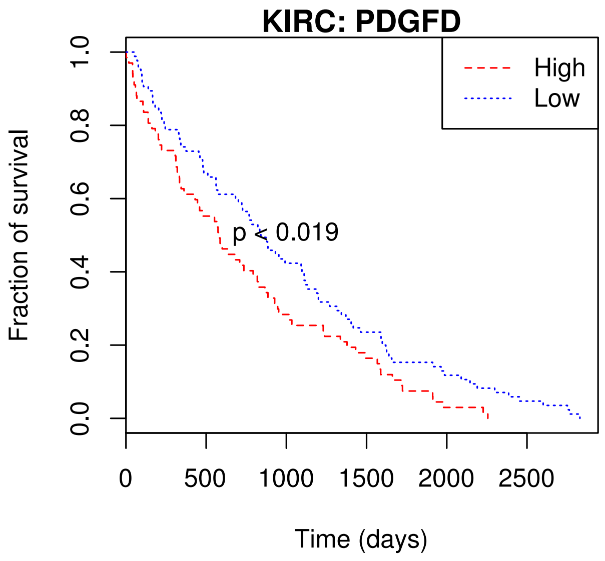
KIRC: PDGFD
High
Low
p < 0.019
Fraction of survival
Time (days)
0.0 0.2 0.4 0.6 0.8 1.0
0 500 1000 1500 2000 2500

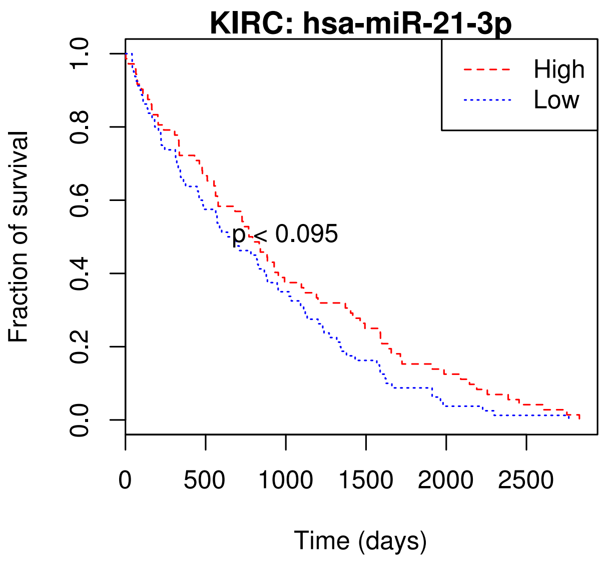
KIRC: hsa-miR-21-3p
High
Low
p < 0.095
Fraction of survival
Time (days)
0.0 0.2 0.4 0.6 0.8 1.0
0 500 1000 1500 2000 2500

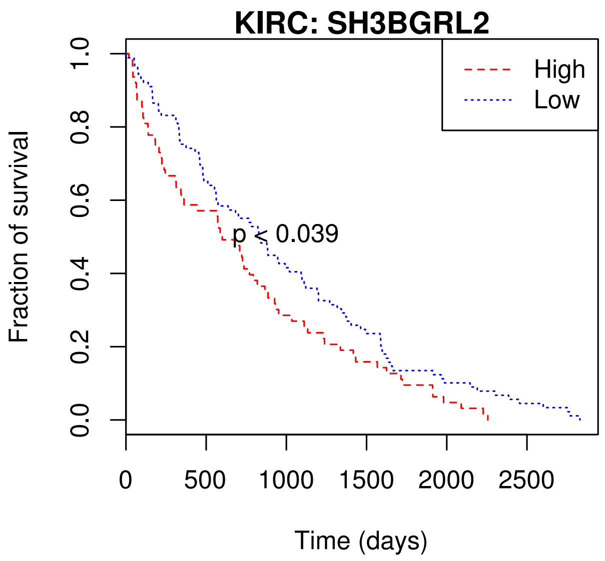
KIRC: SH3BGRL2
High
Low
p < 0.039
Fraction of survival
Time (days)
0.0 0.2 0.4 0.6 0.8 1.0
0 500 1000 1500 2000 2500

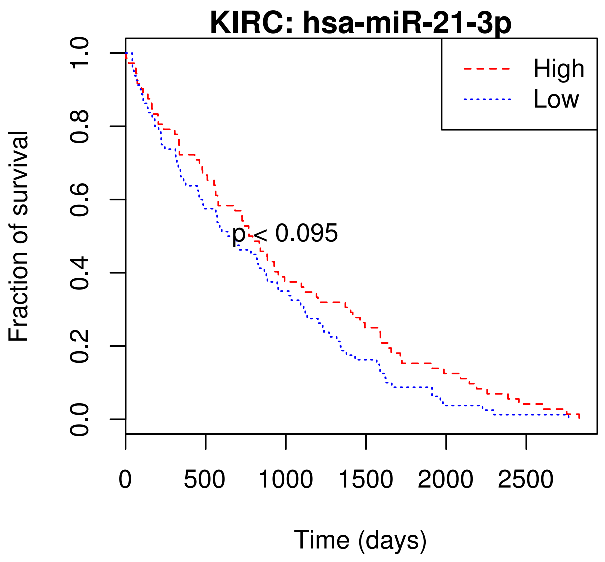
KIRC: hsa-miR-21-3p
High
Low
p < 0.095
Fraction of survival
Time (days)
0.0 0.2 0.4 0.6 0.8 1.0
0 500 1000 1500 2000 2500

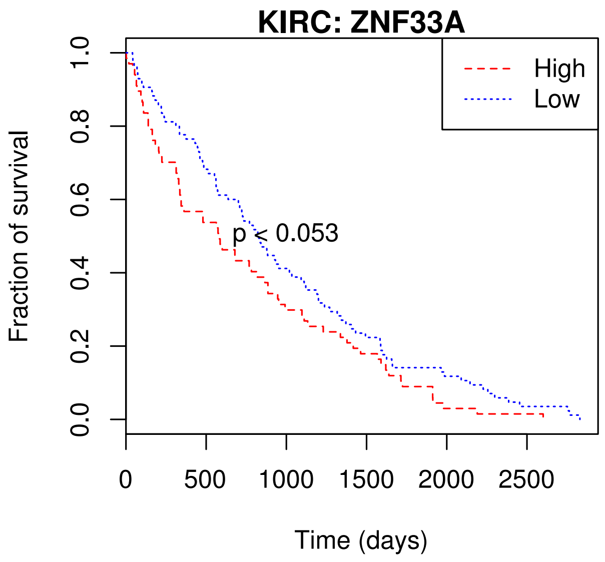
KIRC: ZNF33A
High
Low
p < 0.053
Fraction of survival
Time (days)
0.0 0.2 0.4 0.6 0.8 1.0
0 500 1000 1500 2000 2500

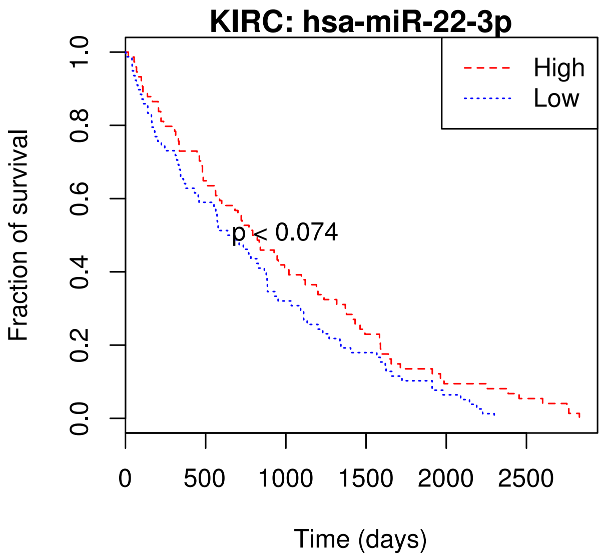
KIRC: hsa-miR-22-3p
High
Low
p < 0.074
Fraction of survival
Time (days)

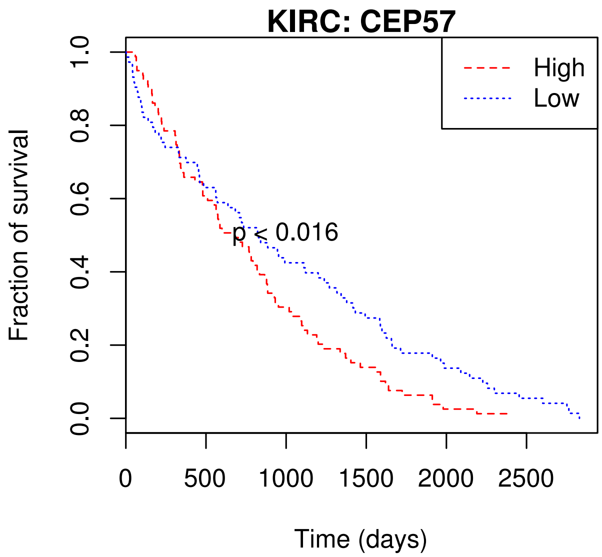
KIRC: CEP57
High
Low
p < 0.016
Fraction of survival
Time (days)

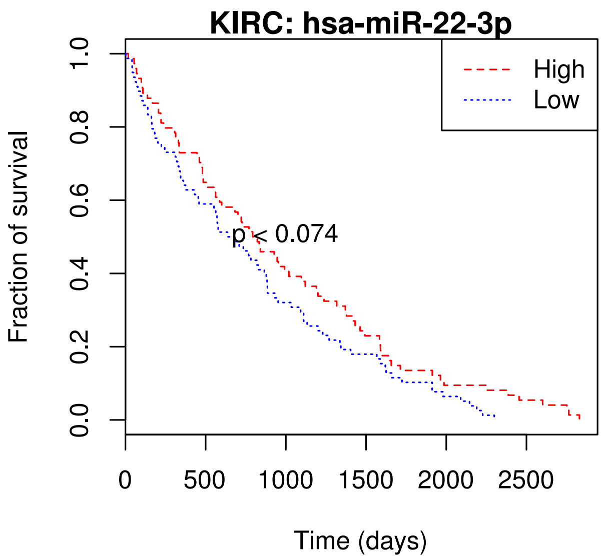
KIRC: hsa-miR-22-3p
High
Low
p < 0.074
Fraction of survival
Time (days)

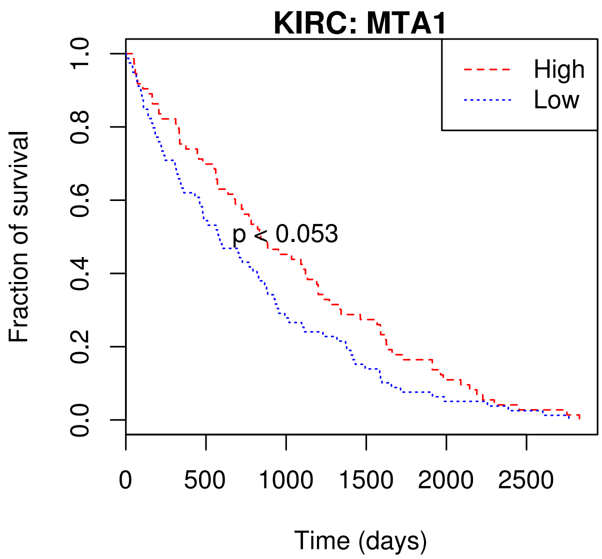
KIRC: MTA1
High
Low
p < 0.053
Fraction of survival
Time (days)

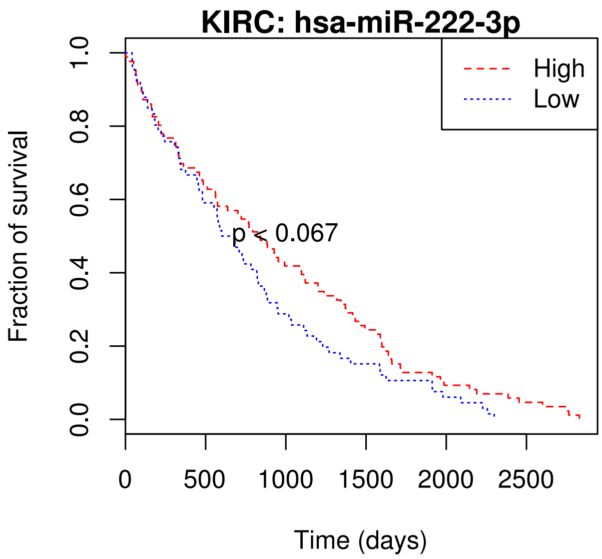
KIRC: hsa-miR-222-3p
High
Low
p < 0.067
Fraction of survival
Time (days)

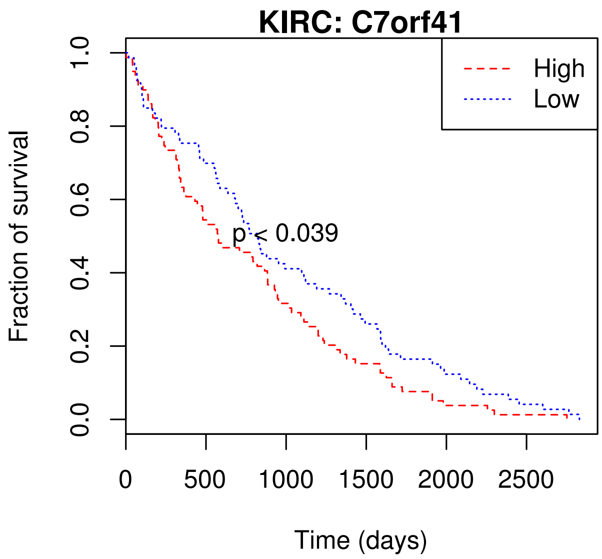
KIRC: C7orf41
High
Low
p < 0.039
Fraction of survival
Time (days)

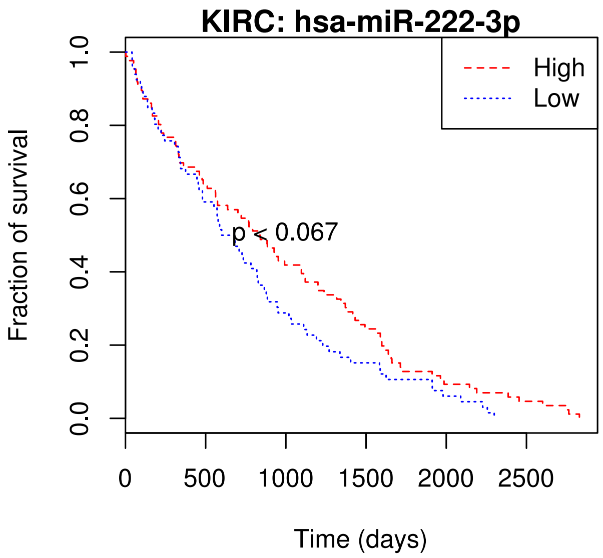
KIRC: hsa-miR-222-3p
High
Low
p < 0.067
Fraction of survival
Time (days)
0.0
0.2
0.4
0.6
0.8
1.0
0
500
1000
1500
2000
2500

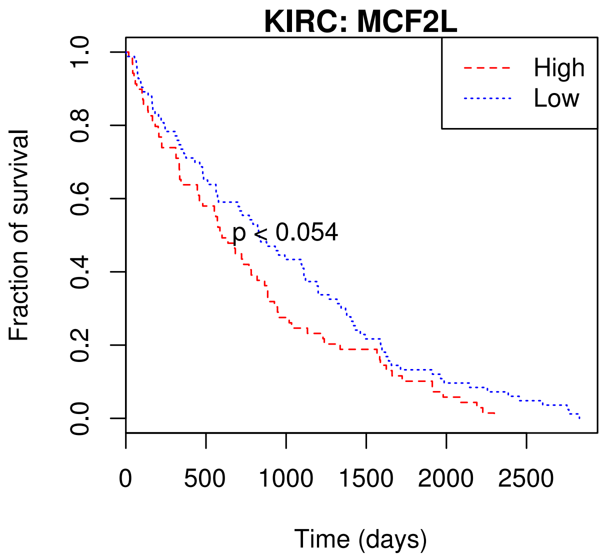
KIRC: MCF2L
High
Low
p < 0.054
Fraction of survival
Time (days)
0.0
0.2
0.4
0.6
0.8
1.0
0
500
1000
1500
2000
2500

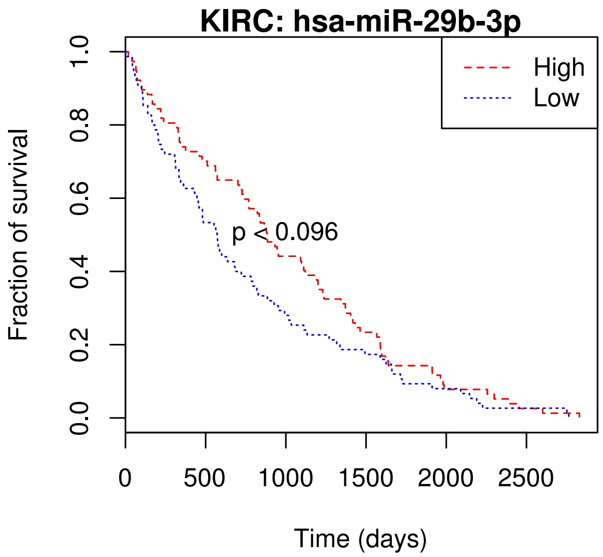
KIRC: hsa-miR-29b-3p
High
Low
p < 0.096
Fraction of survival
Time (days)
0.0
0.2
0.4
0.6
0.8
1.0
0
500
1000
1500
2000
2500

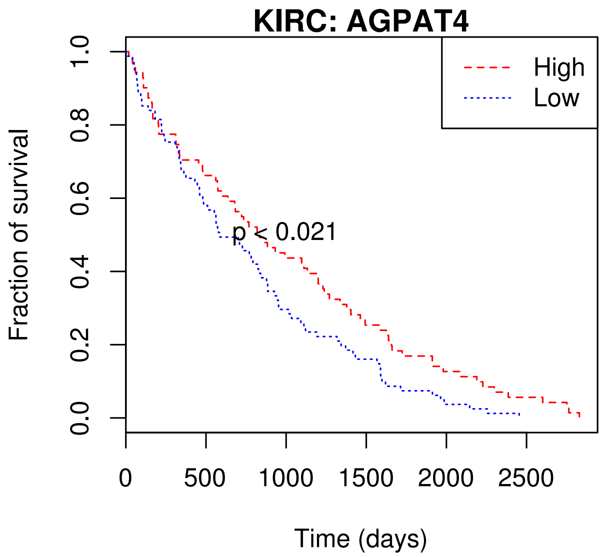
KIRC: AGPAT4
High
Low
p < 0.021
Fraction of survival
Time (days)
0.0
0.2
0.4
0.6
0.8
1.0
0
500
1000
1500
2000
2500

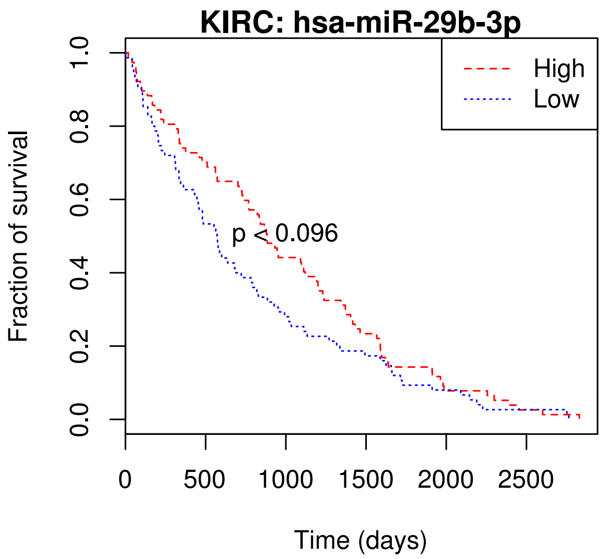
KIRC: hsa-miR-29b-3p
High
Low
p < 0.096
Fraction of survival
Time (days)
0.0
0.2
0.4
0.6
0.8
1.0
0
500
1000
1500
2000
2500

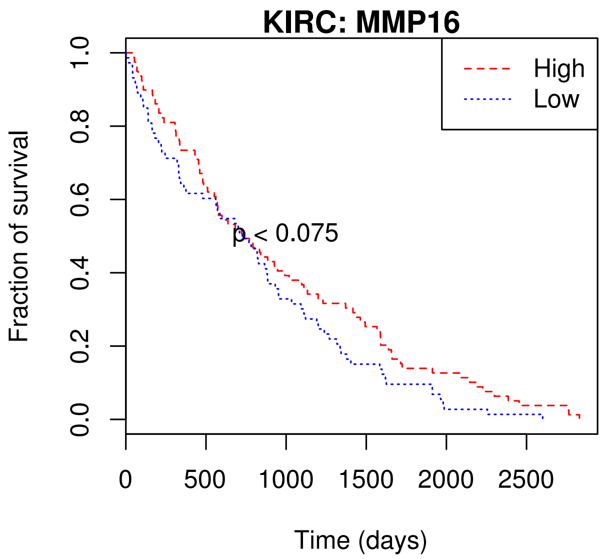
KIRC: MMP16
High
Low
p < 0.075
Fraction of survival
Time (days)
0.0
0.2
0.4
0.6
0.8
1.0
0
500
1000
1500
2000
2500

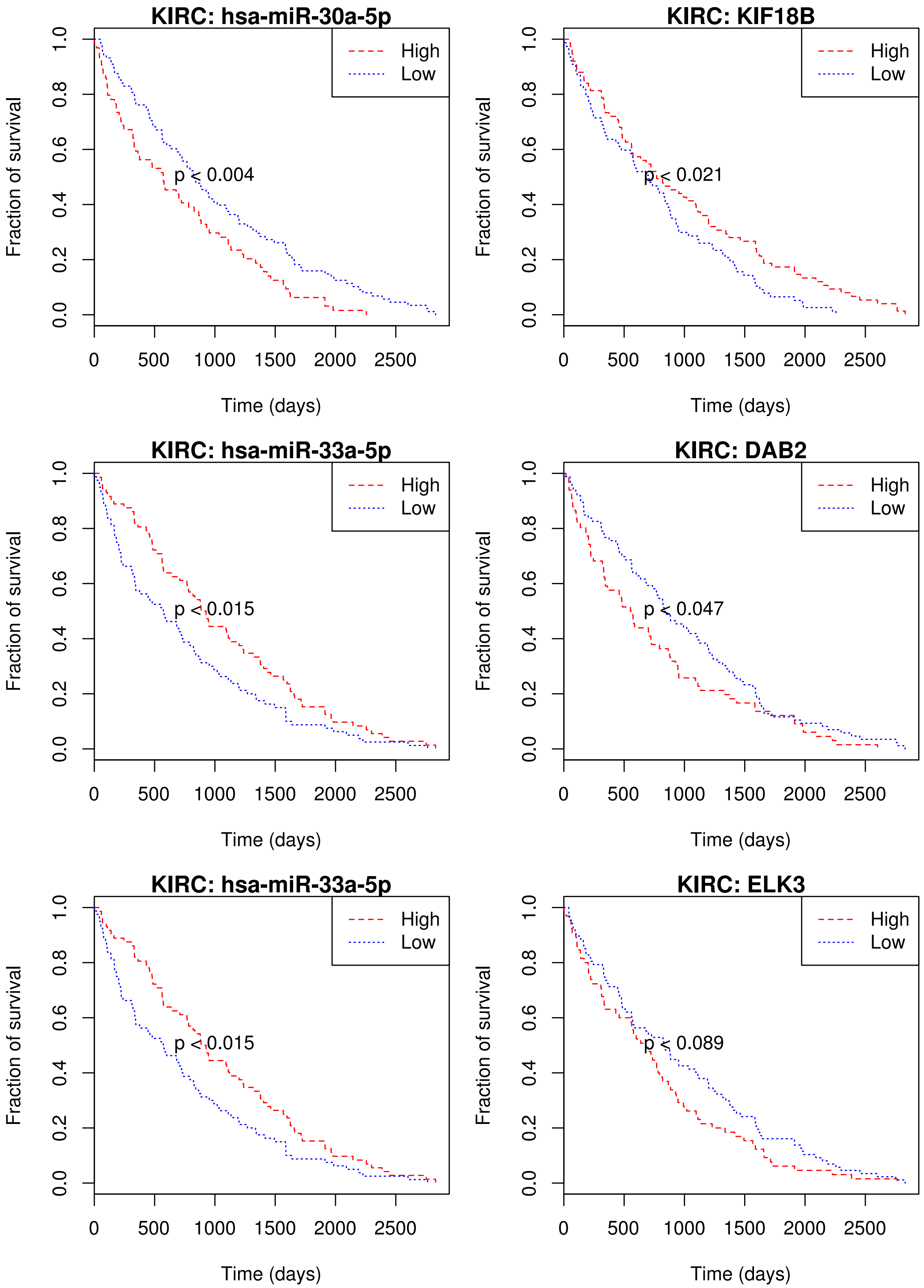
KIRC: hsa-miR-30a-5p
High
Low
p < 0.004
KIRC: KIF18B
High
Low
p < 0.021
KIRC: hsa-miR-33a-5p
High
Low
p < 0.015
KIRC: DAB2
High
Low
p < 0.047
KIRC: hsa-miR-33a-5p
High
Low
p < 0.015
KIRC: ELK3
High
Low
p < 0.089
Fraction of survival
Time (days)

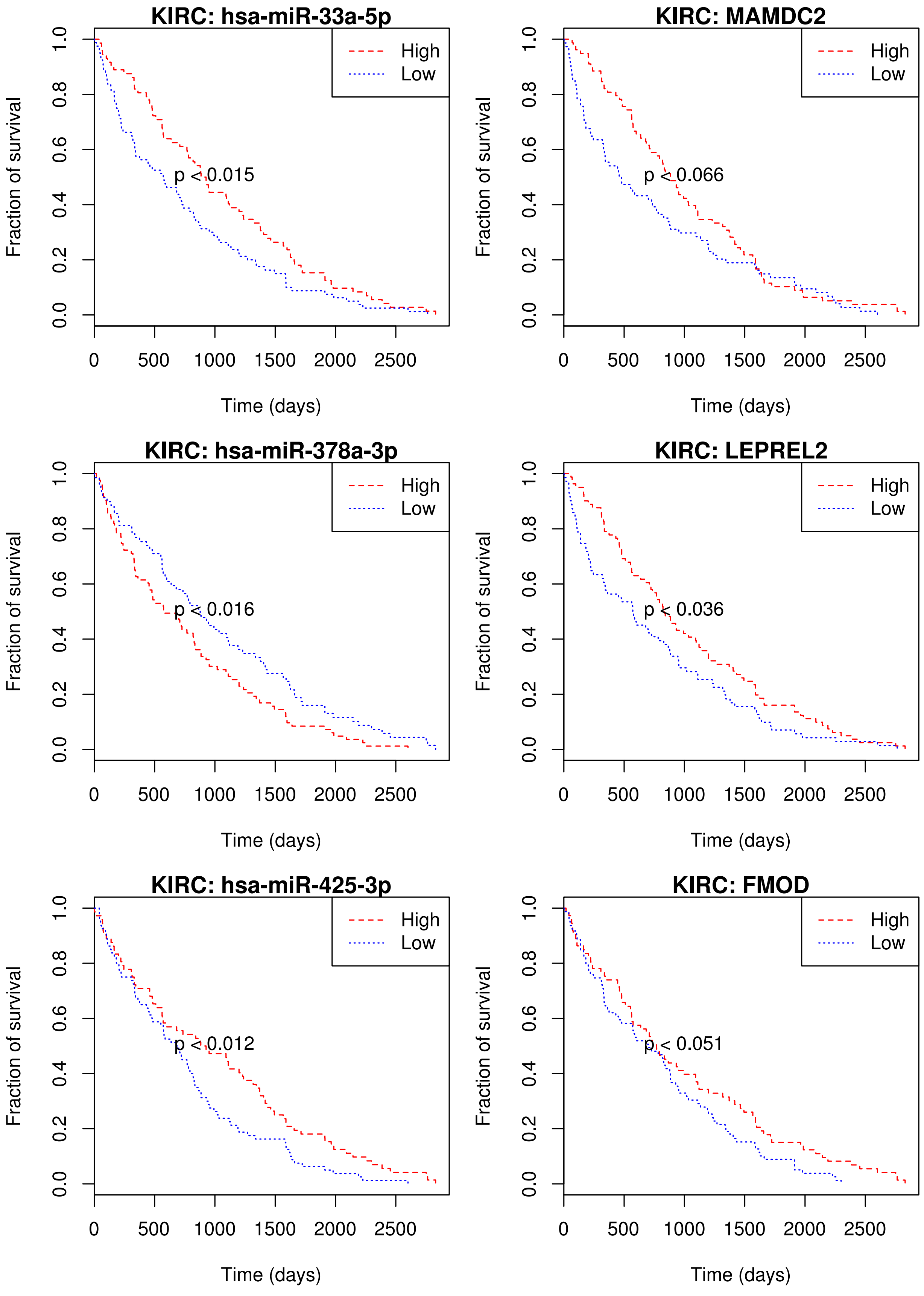
KIRC: hsa-miR-33a-5p
p < 0.015
KIRC: MAMDC2
p < 0.066
KIRC: hsa-miR-378a-3p
p < 0.016
KIRC: LEPREL2
p < 0.036
KIRC: hsa-miR-425-3p
p < 0.012
KIRC: FMOD
p < 0.051
High
Low
Fraction of survival
Time (days)
0.0
0.2
0.4
0.6
0.8
1.0
0
500
1000
1500
2000
2500

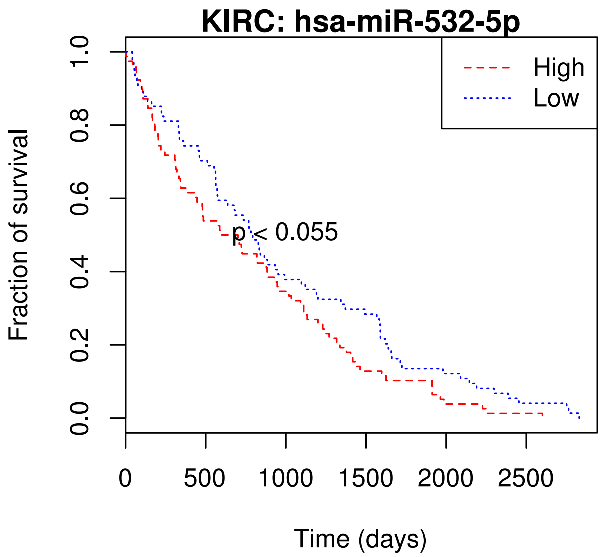
KIRC: hsa-miR-532-5p
High
Low
p < 0.055
Fraction of survival
Time (days)

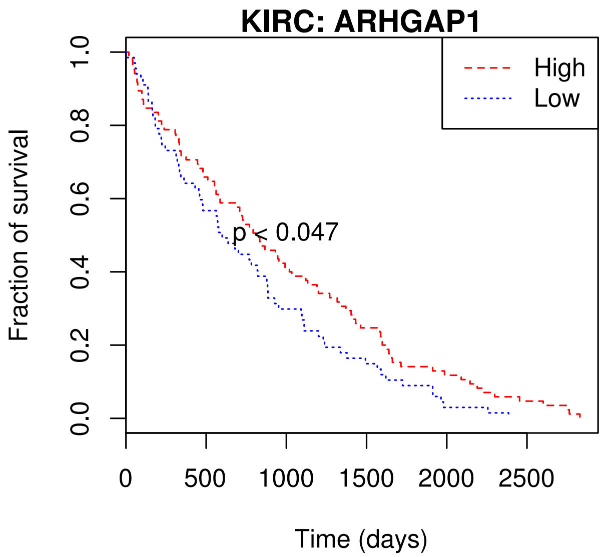
KIRC: ARHGAP1
High
Low
p < 0.047
Fraction of survival
Time (days)

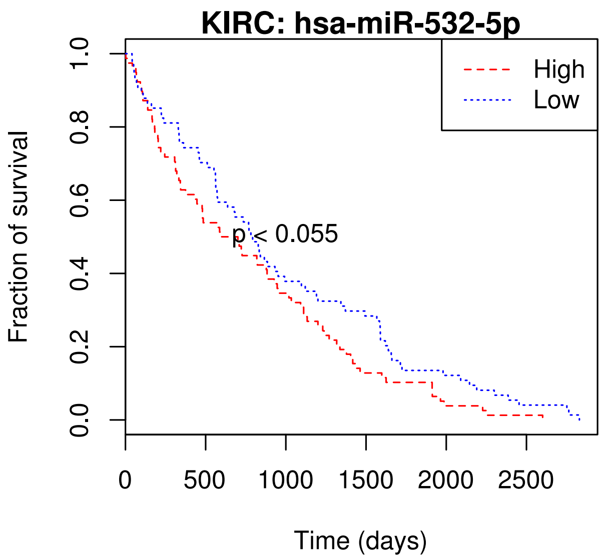
KIRC: hsa-miR-532-5p
High
Low
p < 0.055
Fraction of survival
Time (days)

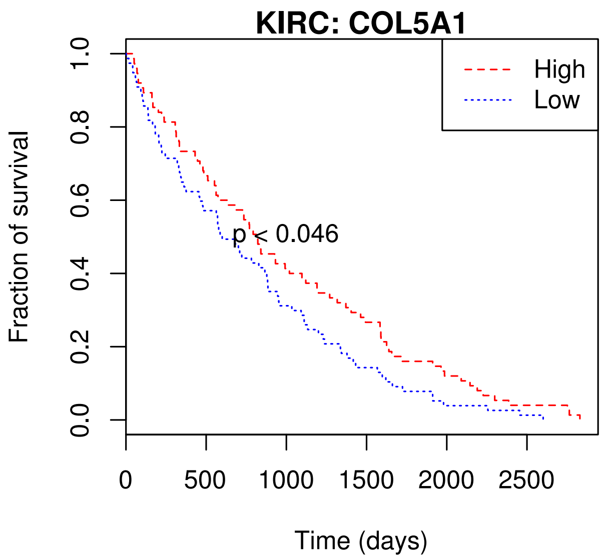
KIRC: COL5A1
High
Low
p < 0.046
Fraction of survival
Time (days)

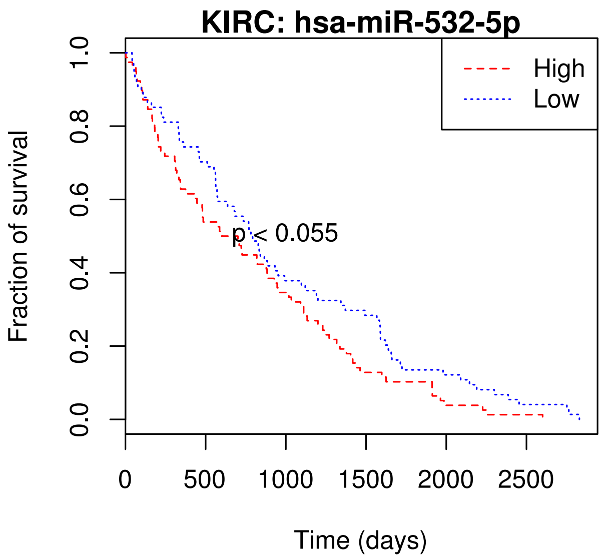
KIRC: hsa-miR-532-5p
High
Low
p < 0.055
Fraction of survival
Time (days)

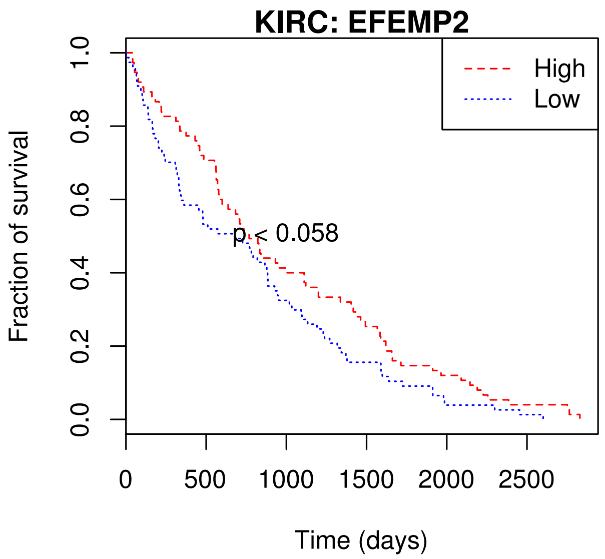
KIRC: EFEMP2
High
Low
p < 0.058
Fraction of survival
Time (days)

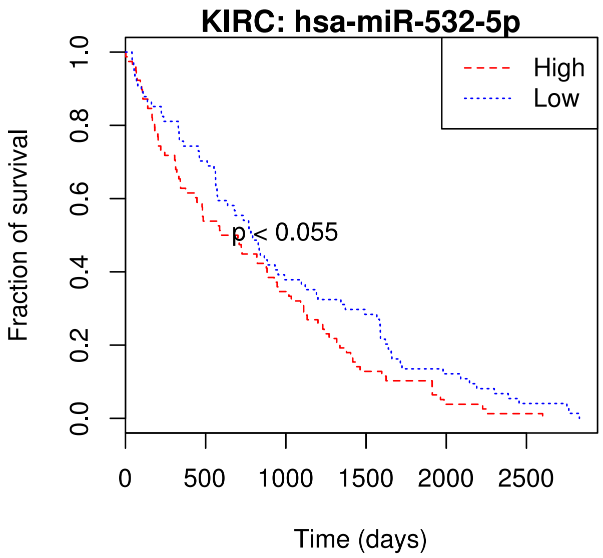
KIRC: hsa-miR-532-5p
High
Low
p < 0.055
Fraction of survival
Time (days)
0 500 1000 1500 2000 2500
0.0 0.2 0.4 0.6 0.8 1.0

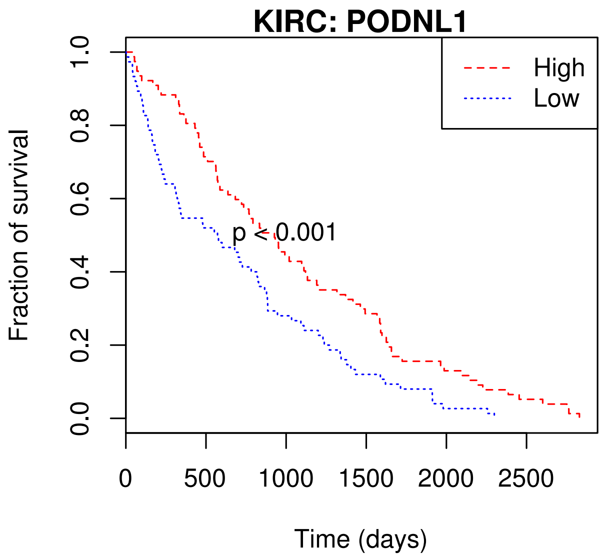
KIRC: PODNL1
High
Low
p < 0.001
Fraction of survival
Time (days)
0 500 1000 1500 2000 2500
0.0 0.2 0.4 0.6 0.8 1.0

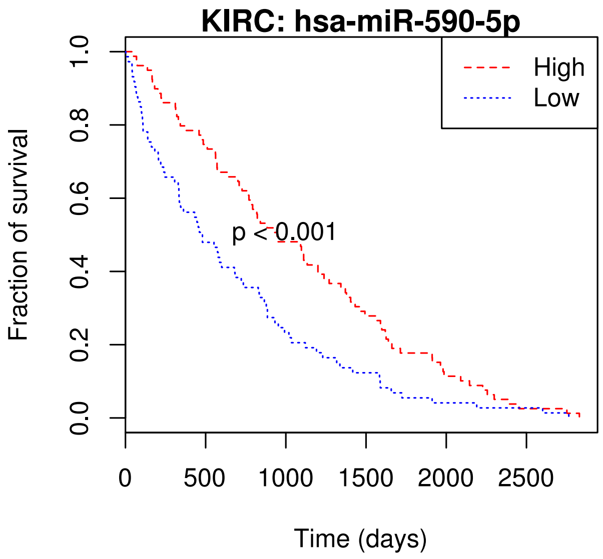
KIRC: hsa-miR-590-5p
High
Low
p < 0.001
Fraction of survival
Time (days)
0 500 1000 1500 2000 2500
0.0 0.2 0.4 0.6 0.8 1.0

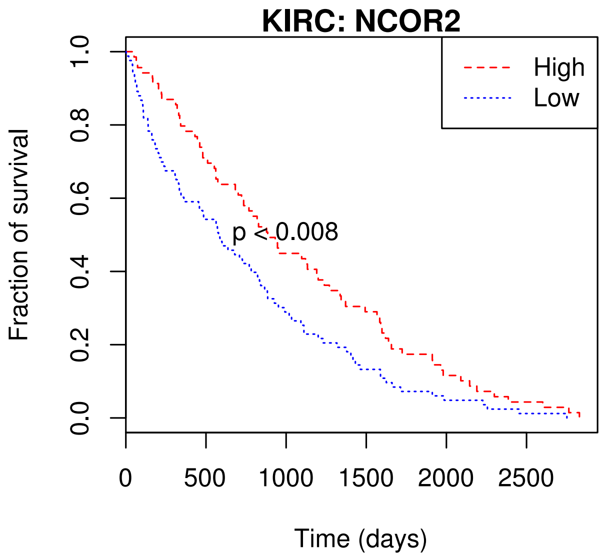
KIRC: NCOR2
High
Low
p < 0.008
Fraction of survival
Time (days)
0 500 1000 1500 2000 2500
0.0 0.2 0.4 0.6 0.8 1.0

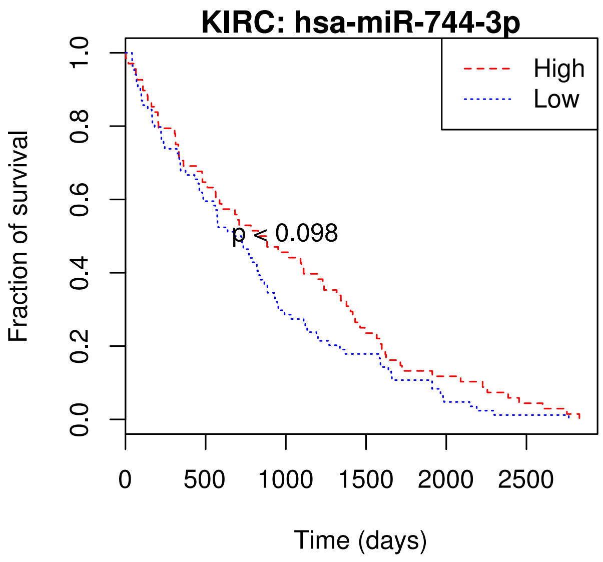
KIRC: hsa-miR-744-3p
High
Low
p < 0.098
Fraction of survival
Time (days)
0 500 1000 1500 2000 2500
0.0 0.2 0.4 0.6 0.8 1.0

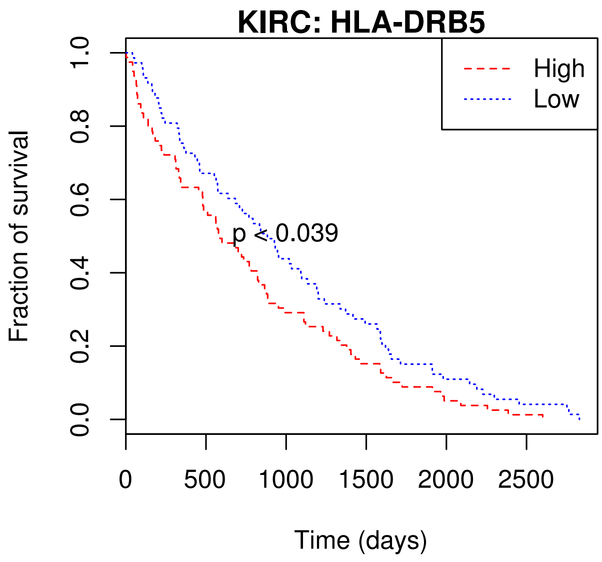
KIRC: HLA-DRB5
High
Low
p < 0.039
Fraction of survival
Time (days)
0 500 1000 1500 2000 2500
0.0 0.2 0.4 0.6 0.8 1.0

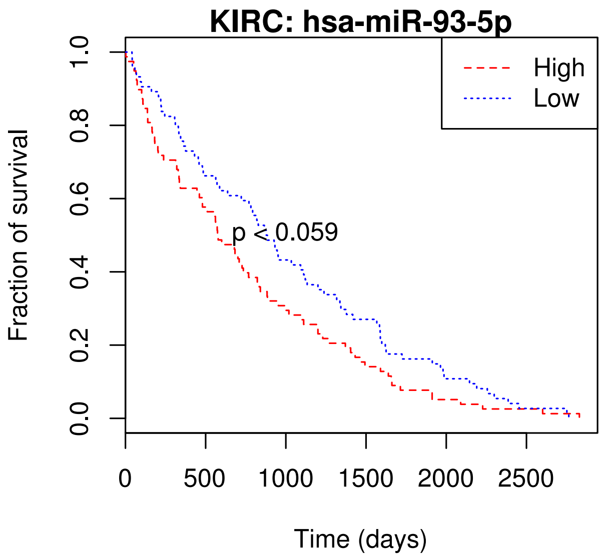
KIRC: hsa-miR-93-5p
High
Low
p < 0.059
Fraction of survival
Time (days)
0.0 0.2 0.4 0.6 0.8 1.0
0 500 1000 1500 2000 2500

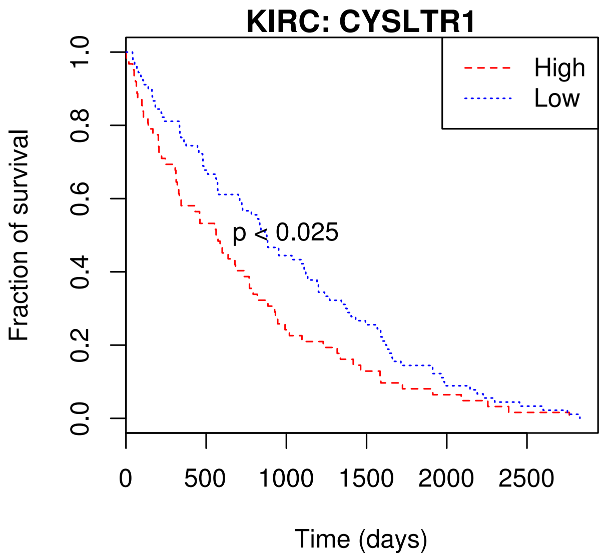
KIRC: CYSLTR1
High
Low
p < 0.025
Fraction of survival
Time (days)
0.0 0.2 0.4 0.6 0.8 1.0
0 500 1000 1500 2000 2500

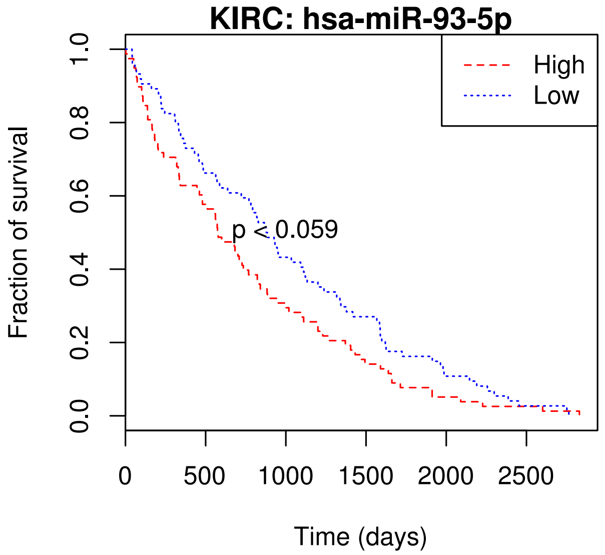
KIRC: hsa-miR-93-5p
High
Low
p < 0.059
Fraction of survival
Time (days)
0.0 0.2 0.4 0.6 0.8 1.0
0 500 1000 1500 2000 2500

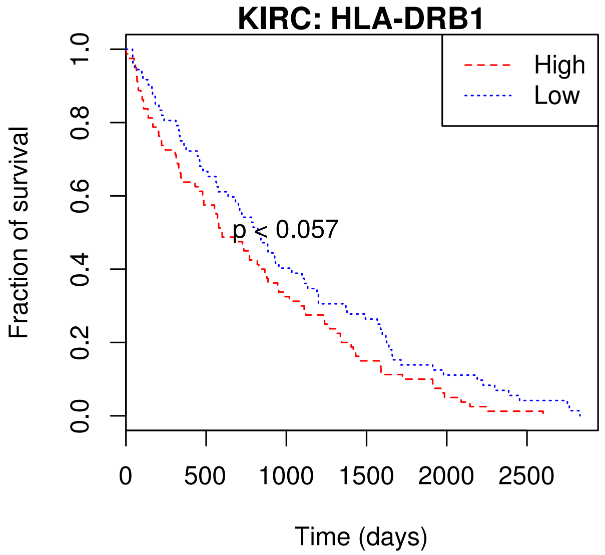
KIRC: HLA-DRB1
High
Low
p < 0.057
Fraction of survival
Time (days)
0.0 0.2 0.4 0.6 0.8 1.0
0 500 1000 1500 2000 2500

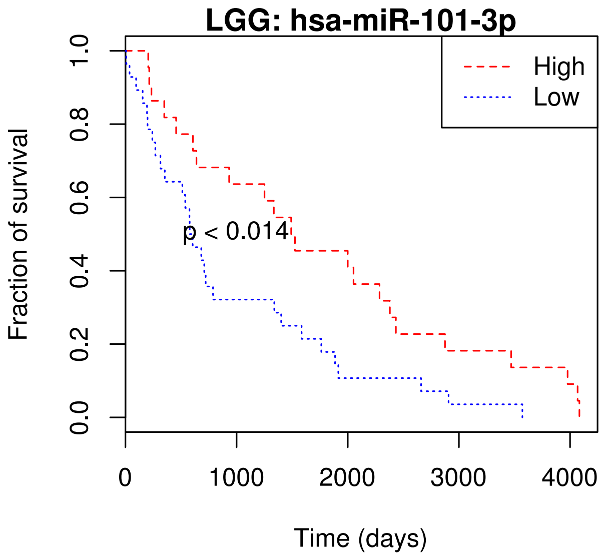
LGG: hsa-miR-101-3p
High
Low
p < 0.014
Fraction of survival
Time (days)
0.0 0.2 0.4 0.6 0.8 1.0
0 1000 2000 3000 4000

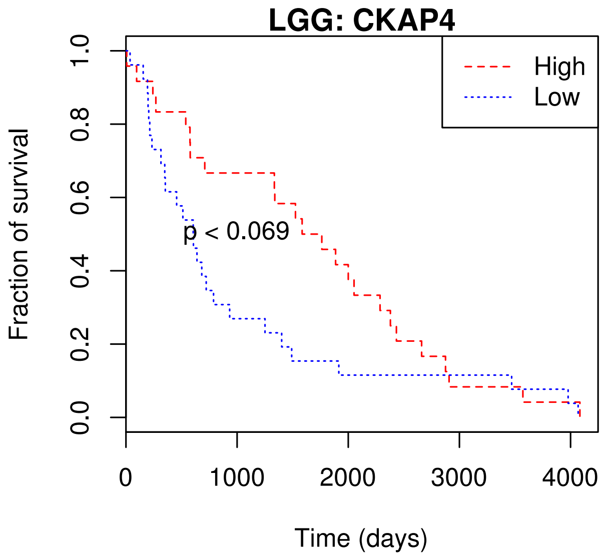
LGG: CKAP4
High
Low
p < 0.069
Fraction of survival
Time (days)
0.0 0.2 0.4 0.6 0.8 1.0
0 1000 2000 3000 4000

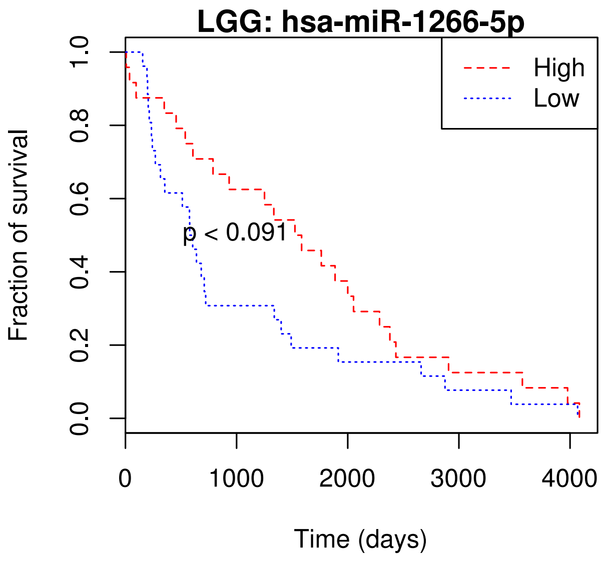
LGG: hsa-miR-1266-5p
High
Low
p < 0.091
Fraction of survival
0.0
0.2
0.4
0.6
0.8
1.0
0
1000
2000
3000
4000
Time (days)

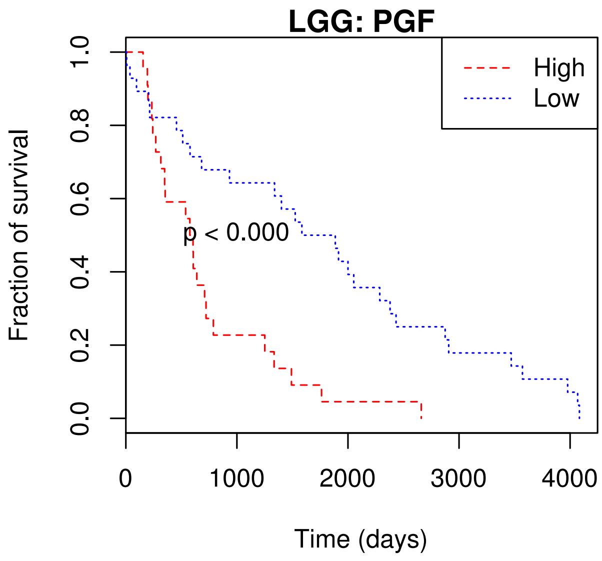
LGG: PGF
High
Low
p < 0.000
Fraction of survival
0.0
0.2
0.4
0.6
0.8
1.0
0
1000
2000
3000
4000
Time (days)

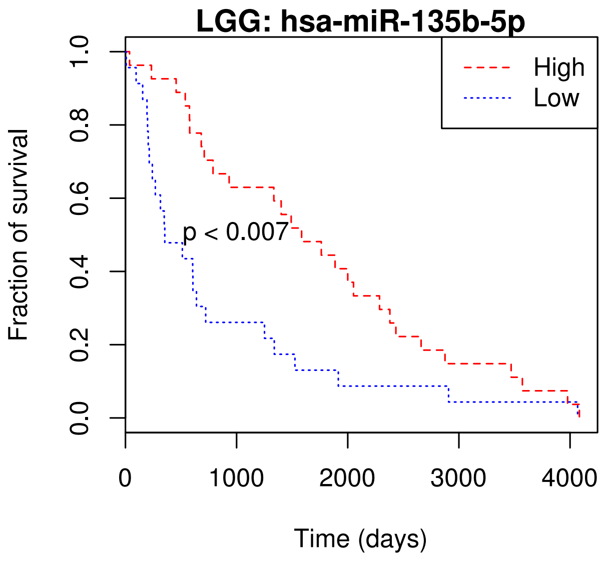
LGG: hsa-miR-135b-5p
High
Low
p < 0.007
Fraction of survival
0.0
0.2
0.4
0.6
0.8
1.0
0
1000
2000
3000
4000
Time (days)

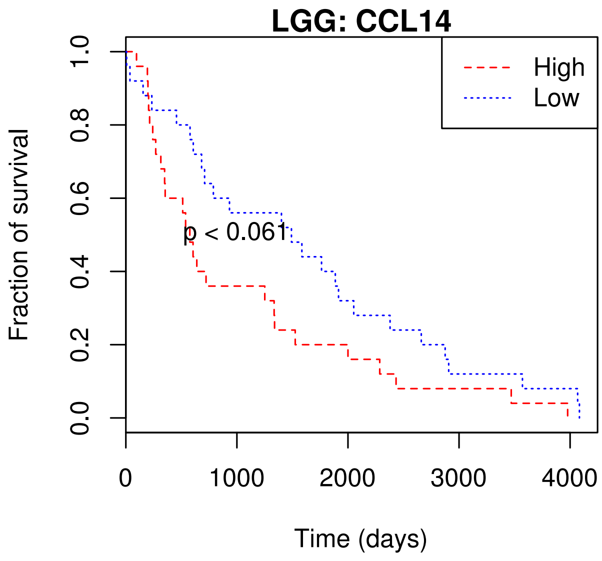
LGG: CCL14
High
Low
p < 0.061
Fraction of survival
0.0
0.2
0.4
0.6
0.8
1.0
0
1000
2000
3000
4000
Time (days)

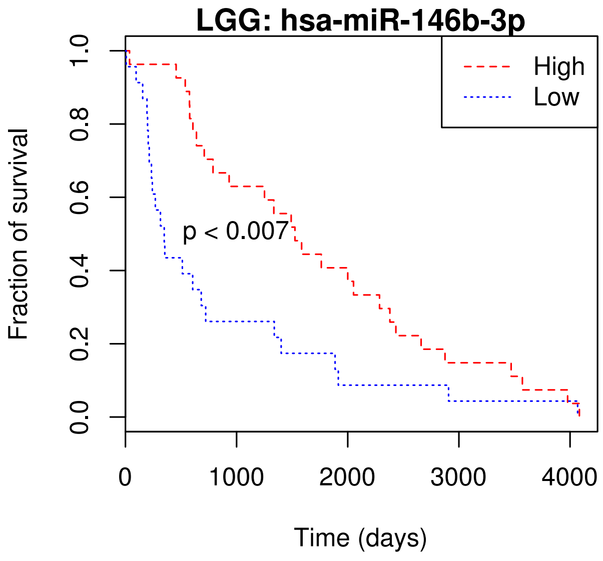
LGG: hsa-miR-146b-3p
High
Low
p < 0.007
Fraction of survival
0.0
0.2
0.4
0.6
0.8
1.0
0
1000
2000
3000
4000
Time (days)

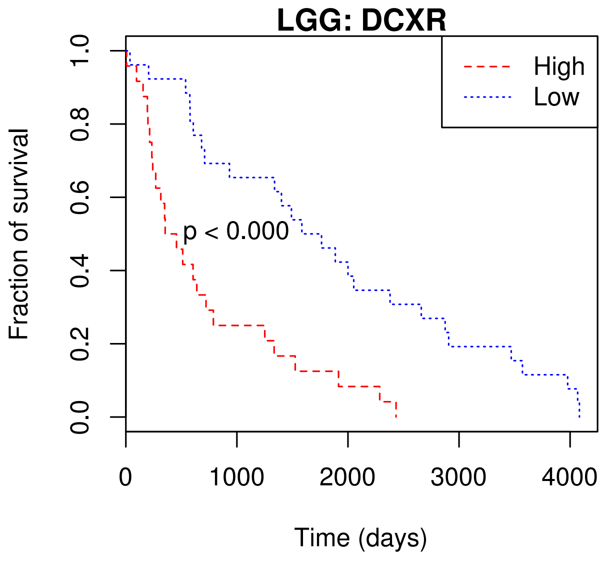
LGG: DCXR
High
Low
p < 0.000
Fraction of survival
0.0
0.2
0.4
0.6
0.8
1.0
0
1000
2000
3000
4000
Time (days)

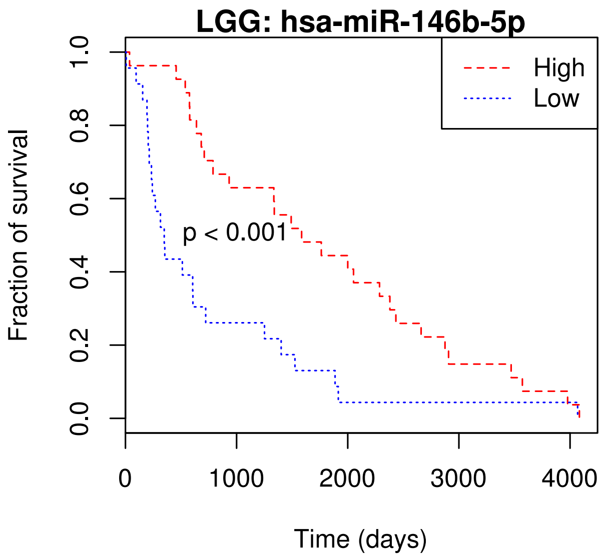
LGG: hsa-miR-146b-5p
High
Low
p < 0.001
Fraction of survival
0.0 0.2 0.4 0.6 0.8 1.0
0 1000 2000 3000 4000
Time (days)

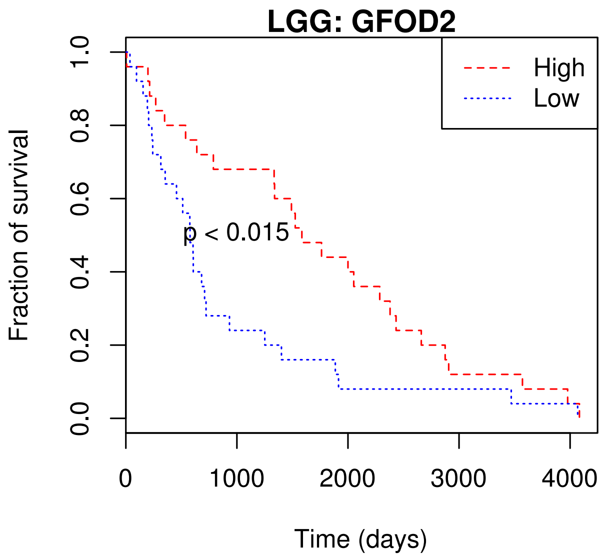
LGG: GFOD2
High
Low
p < 0.015
Fraction of survival
0.0 0.2 0.4 0.6 0.8 1.0
0 1000 2000 3000 4000
Time (days)

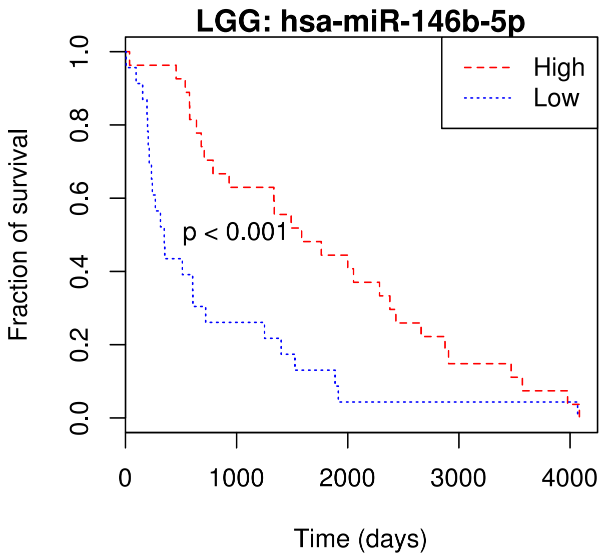
LGG: hsa-miR-146b-5p
High
Low
p < 0.001
Fraction of survival
0.0 0.2 0.4 0.6 0.8 1.0
0 1000 2000 3000 4000
Time (days)

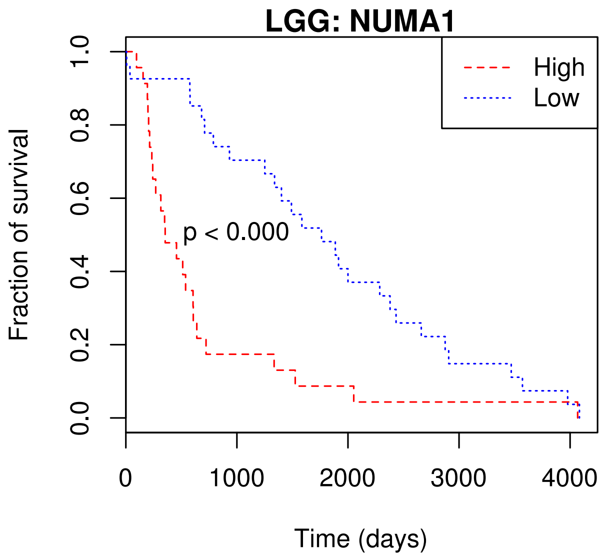
LGG: NUMA1
High
Low
p < 0.000
Fraction of survival
0.0 0.2 0.4 0.6 0.8 1.0
0 1000 2000 3000 4000
Time (days)

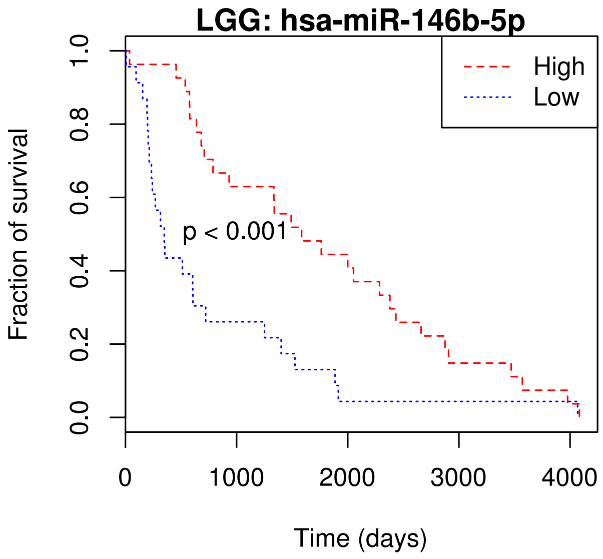
LGG: hsa-miR-146b-5p
High
Low
p < 0.001
Fraction of survival
0.0 0.2 0.4 0.6 0.8 1.0
0 1000 2000 3000 4000
Time (days)

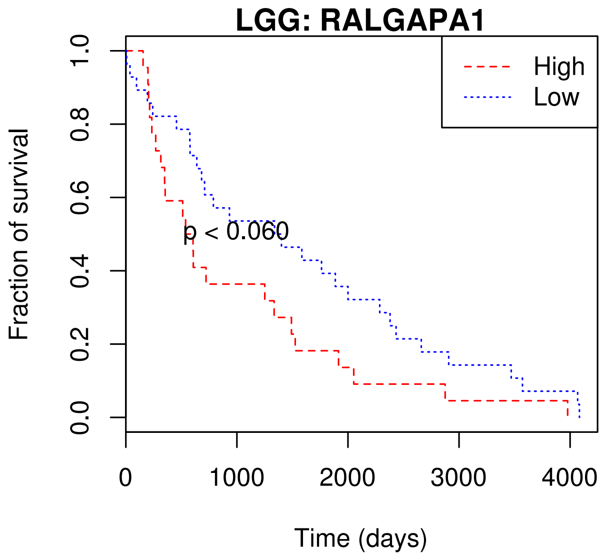
LGG: RALGAPA1
High
Low
p < 0.060
Fraction of survival
0.0 0.2 0.4 0.6 0.8 1.0
0 1000 2000 3000 4000
Time (days)

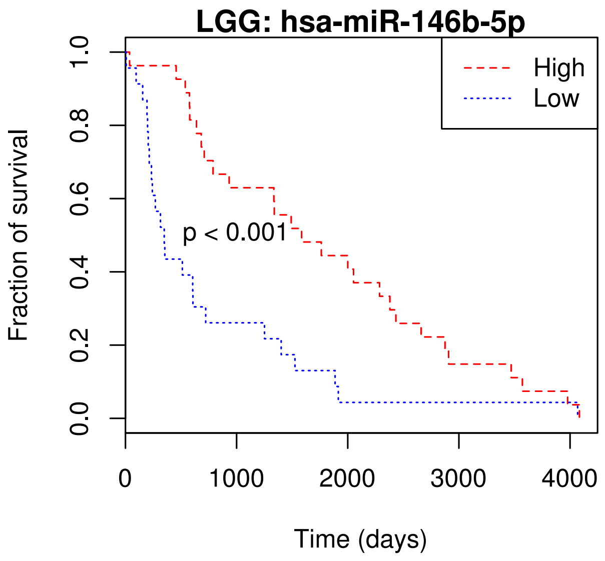
LGG: hsa-miR-146b-5p
High
Low
p < 0.001
Fraction of survival
Time (days)
0.0
0.2
0.4
0.6
0.8
1.0
0
1000
2000
3000
4000

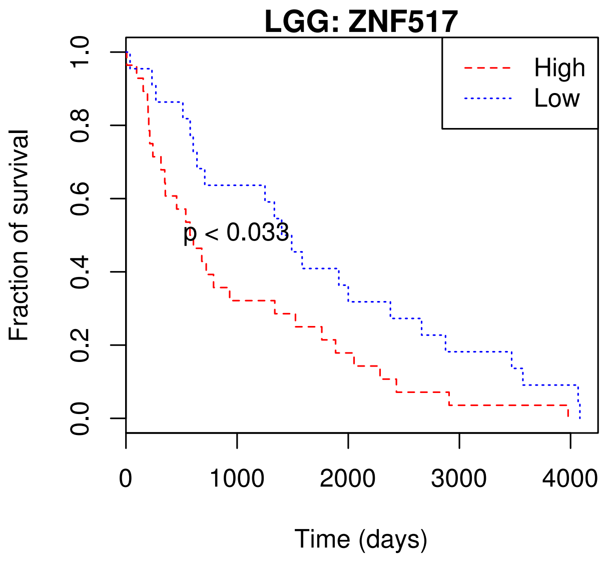
LGG: ZNF517
High
Low
p < 0.033
Fraction of survival
Time (days)
0.0
0.2
0.4
0.6
0.8
1.0
0
1000
2000
3000
4000

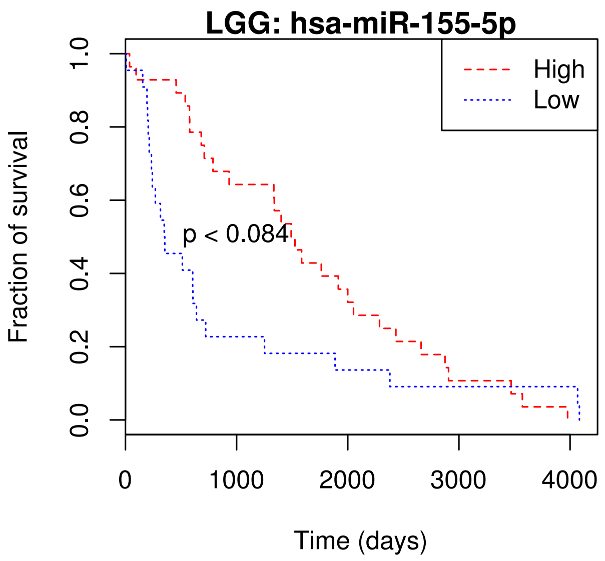
LGG: hsa-miR-155-5p
High
Low
p < 0.084
Fraction of survival
Time (days)
0.0
0.2
0.4
0.6
0.8
1.0
0
1000
2000
3000
4000

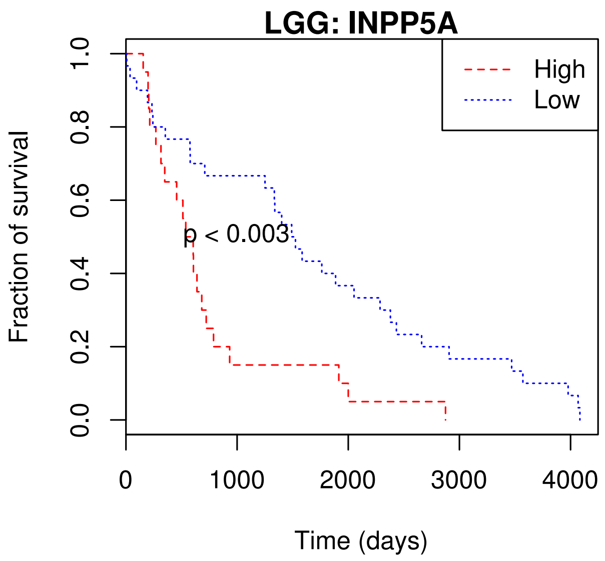
LGG: INPP5A
High
Low
p < 0.003
Fraction of survival
Time (days)
0.0
0.2
0.4
0.6
0.8
1.0
0
1000
2000
3000
4000

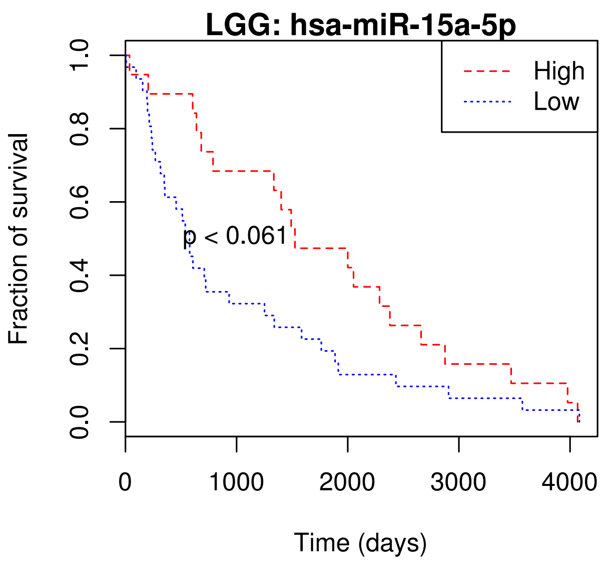
LGG: hsa-miR-15a-5p
High
Low
p < 0.061
Fraction of survival
Time (days)
0.0
0.2
0.4
0.6
0.8
1.0
0
1000
2000
3000
4000

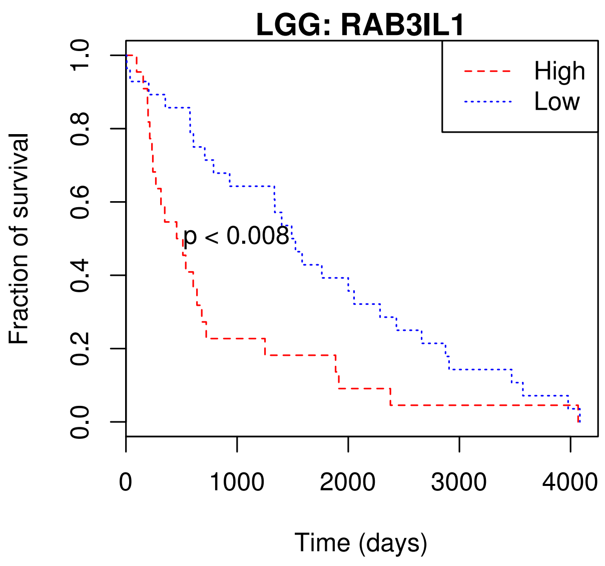
LGG: RAB3IL1
High
Low
p < 0.008
Fraction of survival
Time (days)
0.0
0.2
0.4
0.6
0.8
1.0
0
1000
2000
3000
4000

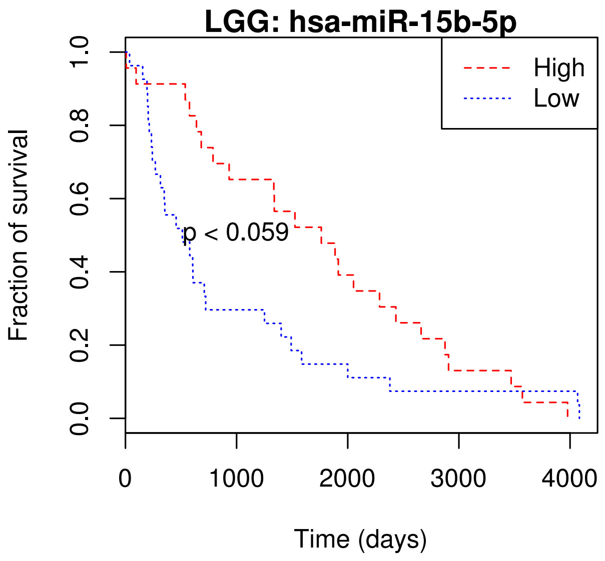
LGG: hsa-miR-15b-5p
High
Low
p < 0.059
Fraction of survival
0.0
0.2
0.4
0.6
0.8
1.0
0
1000
2000
3000
4000
Time (days)

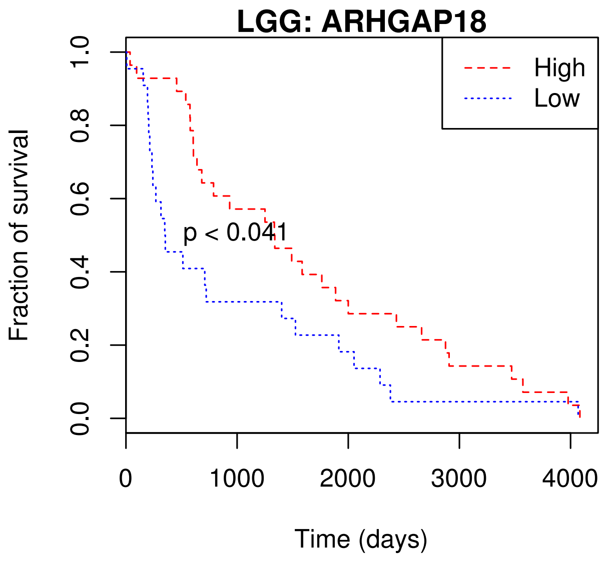
LGG: ARHGAP18
High
Low
p < 0.041
Fraction of survival
0.0
0.2
0.4
0.6
0.8
1.0
0
1000
2000
3000
4000
Time (days)

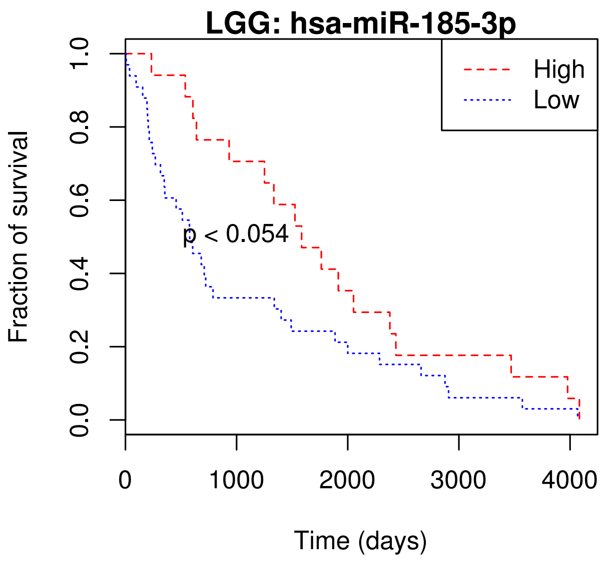
LGG: hsa-miR-185-3p
High
Low
p < 0.054
Fraction of survival
0.0
0.2
0.4
0.6
0.8
1.0
0
1000
2000
3000
4000
Time (days)

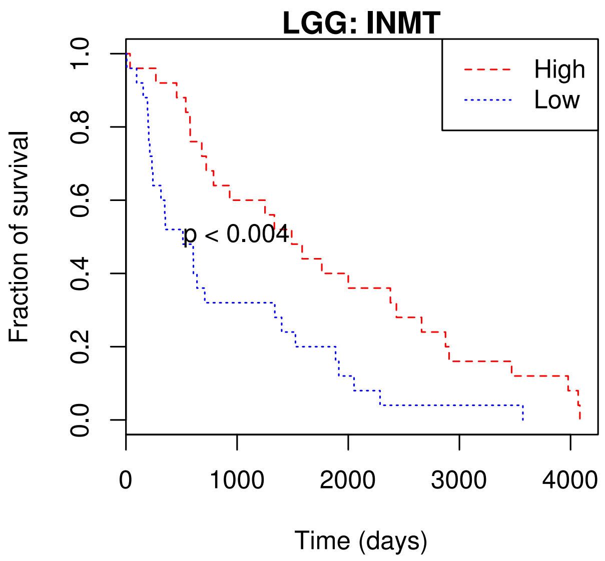
LGG: INMT
High
Low
p < 0.004
Fraction of survival
0.0
0.2
0.4
0.6
0.8
1.0
0
1000
2000
3000
4000
Time (days)

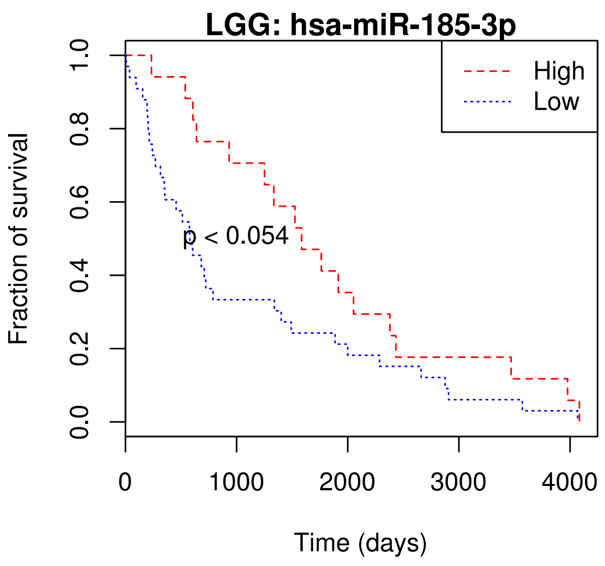
LGG: hsa-miR-185-3p
High
Low
p < 0.054
Fraction of survival
0.0
0.2
0.4
0.6
0.8
1.0
0
1000
2000
3000
4000
Time (days)

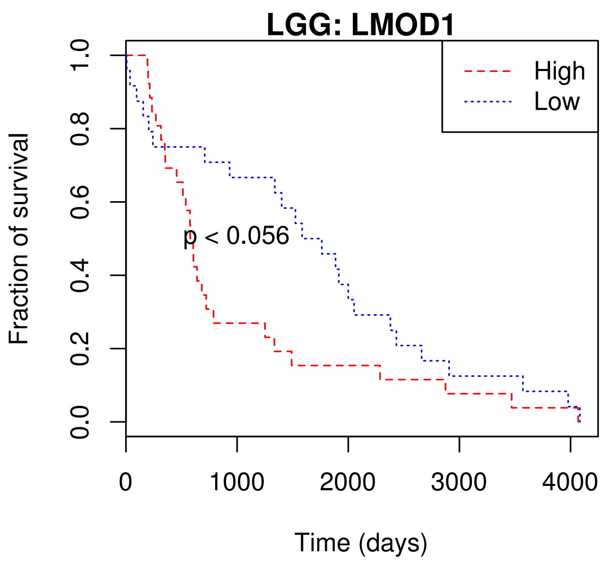
LGG: LMOD1
High
Low
p < 0.056
Fraction of survival
0.0
0.2
0.4
0.6
0.8
1.0
0
1000
2000
3000
4000
Time (days)

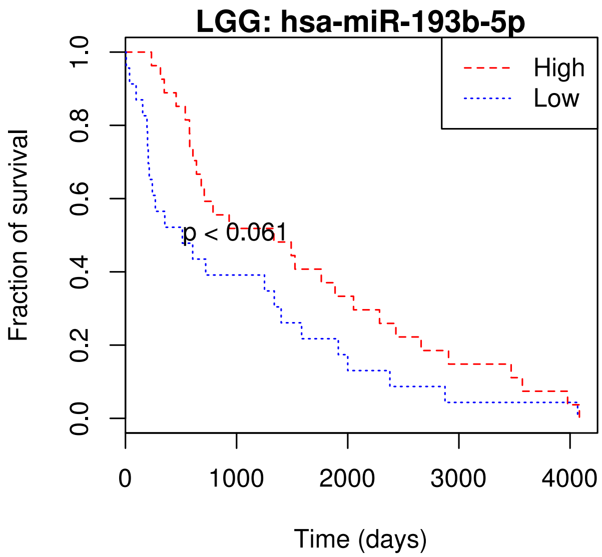
LGG: hsa-miR-193b-5p
High
Low
p < 0.061
Fraction of survival
Time (days)
0.0 0.2 0.4 0.6 0.8 1.0
0 1000 2000 3000 4000

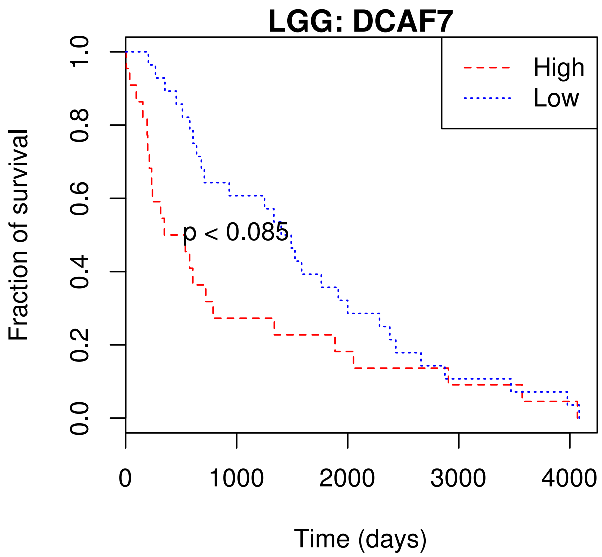
LGG: DCAF7
High
Low
p < 0.085
Fraction of survival
Time (days)
0.0 0.2 0.4 0.6 0.8 1.0
0 1000 2000 3000 4000

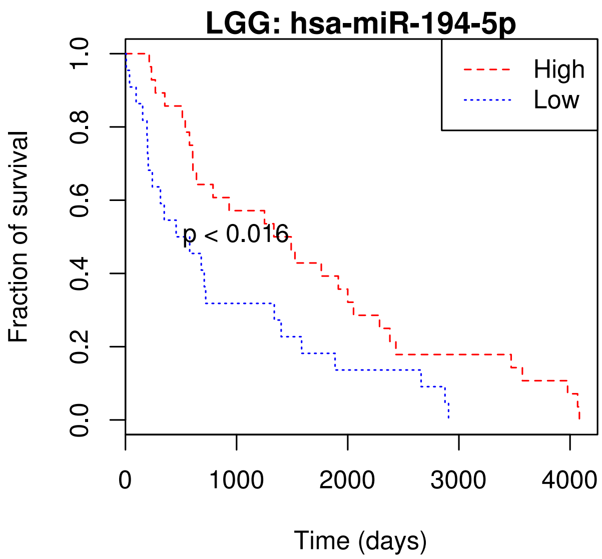
LGG: hsa-miR-194-5p
High
Low
p < 0.016
Fraction of survival
Time (days)
0.0 0.2 0.4 0.6 0.8 1.0
0 1000 2000 3000 4000

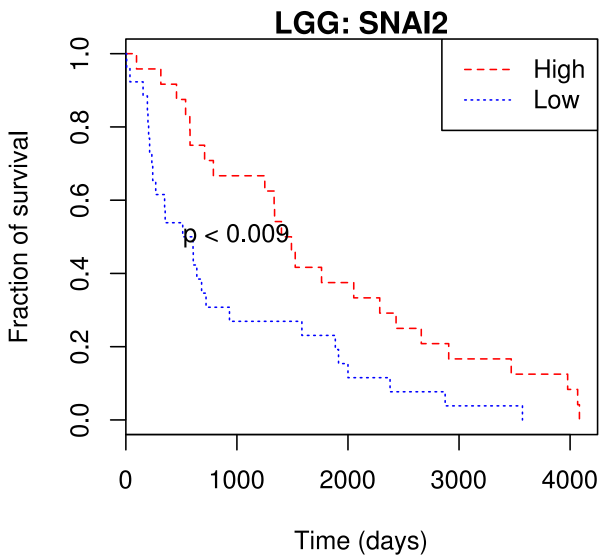
LGG: SNAI2
High
Low
p < 0.009
Fraction of survival
Time (days)
0.0 0.2 0.4 0.6 0.8 1.0
0 1000 2000 3000 4000

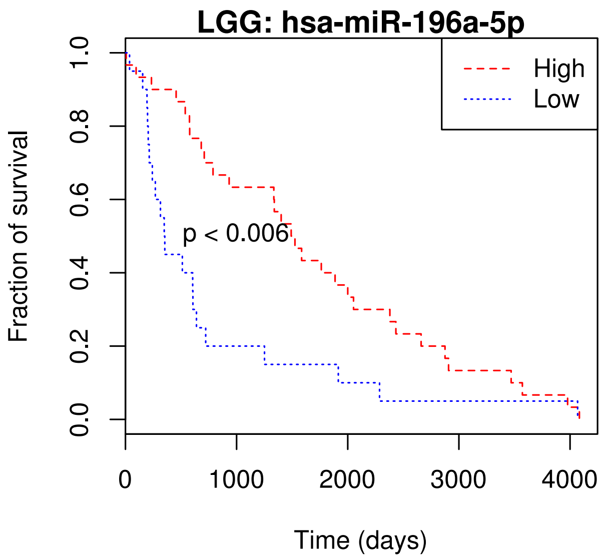
LGG: hsa-miR-196a-5p
High
Low
p < 0.006
Fraction of survival
Time (days)
0.0 0.2 0.4 0.6 0.8 1.0
0 1000 2000 3000 4000

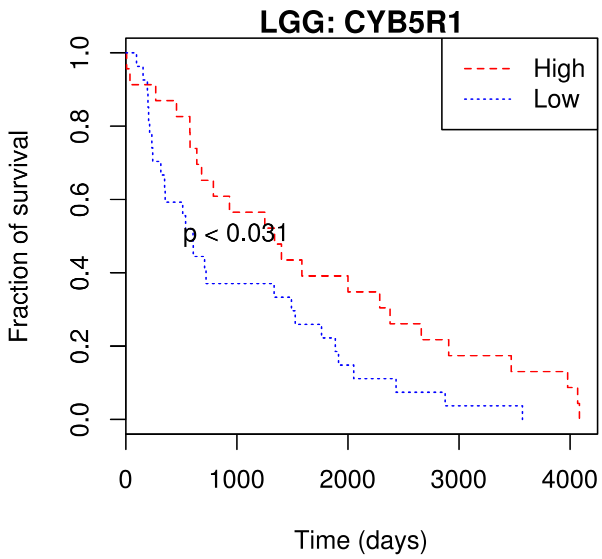
LGG: CYB5R1
High
Low
p < 0.031
Fraction of survival
Time (days)
0.0 0.2 0.4 0.6 0.8 1.0
0 1000 2000 3000 4000

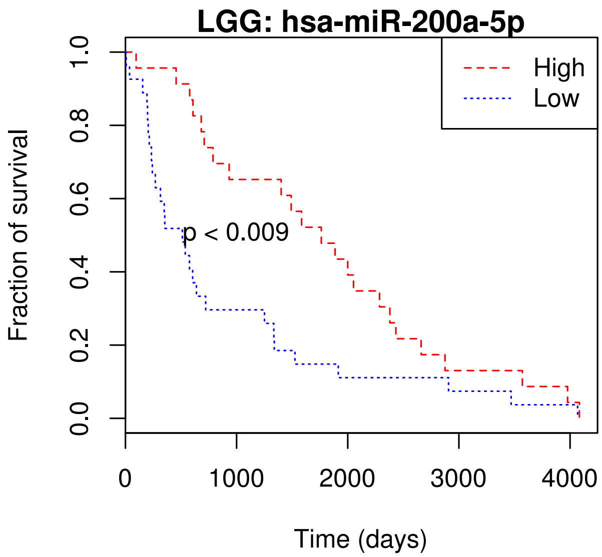
LGG: hsa-miR-200a-5p
High
Low
p < 0.009
Fraction of survival
Time (days)
0.0 0.2 0.4 0.6 0.8 1.0
0 1000 2000 3000 4000

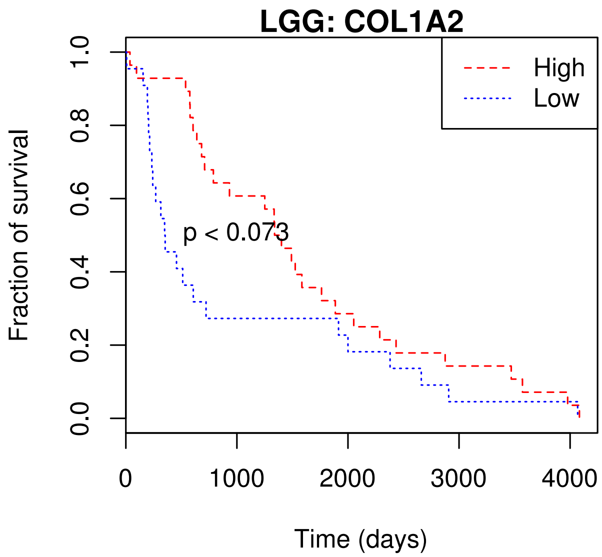
LGG: COL1A2
High
Low
p < 0.073
Fraction of survival
Time (days)
0.0 0.2 0.4 0.6 0.8 1.0
0 1000 2000 3000 4000

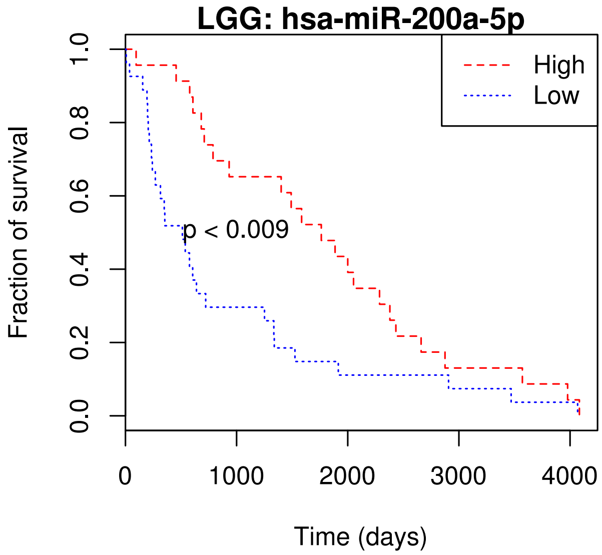
LGG: hsa-miR-200a-5p
High
Low
p < 0.009
Fraction of survival
Time (days)
0.0 0.2 0.4 0.6 0.8 1.0
0 1000 2000 3000 4000

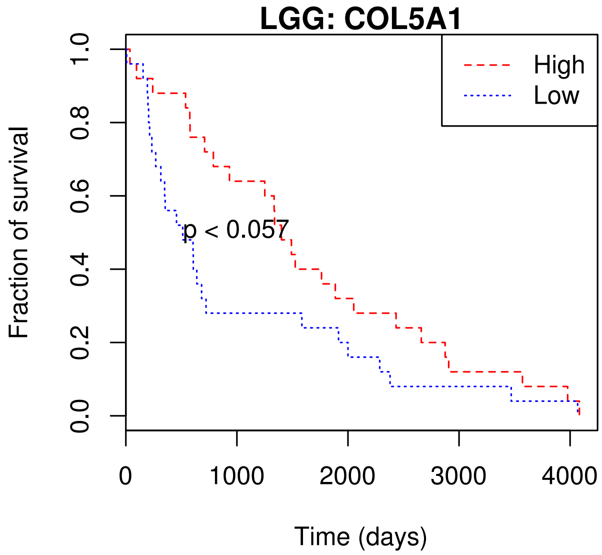
LGG: COL5A1
High
Low
p < 0.057
Fraction of survival
Time (days)
0.0 0.2 0.4 0.6 0.8 1.0
0 1000 2000 3000 4000

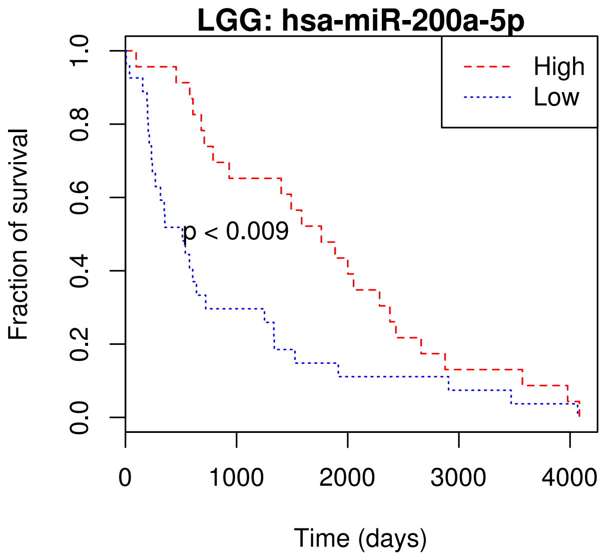
LGG: hsa-miR-200a-5p
High
Low
p < 0.009
Fraction of survival
Time (days)
0.0 0.2 0.4 0.6 0.8 1.0
0 1000 2000 3000 4000

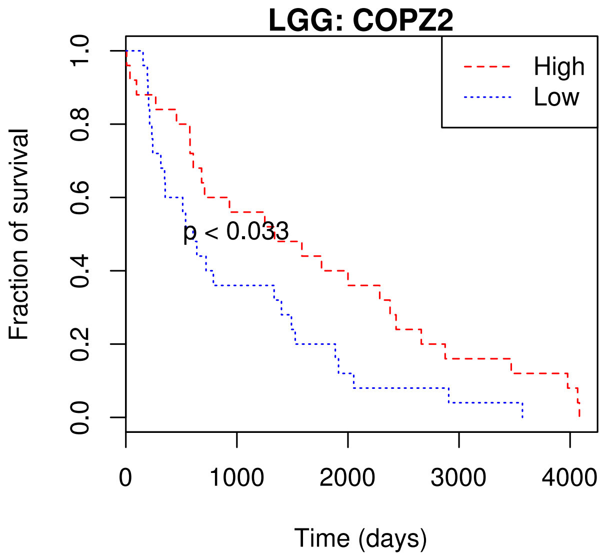
LGG: COPZ2
High
Low
p < 0.033
Fraction of survival
Time (days)
0.0 0.2 0.4 0.6 0.8 1.0
0 1000 2000 3000 4000

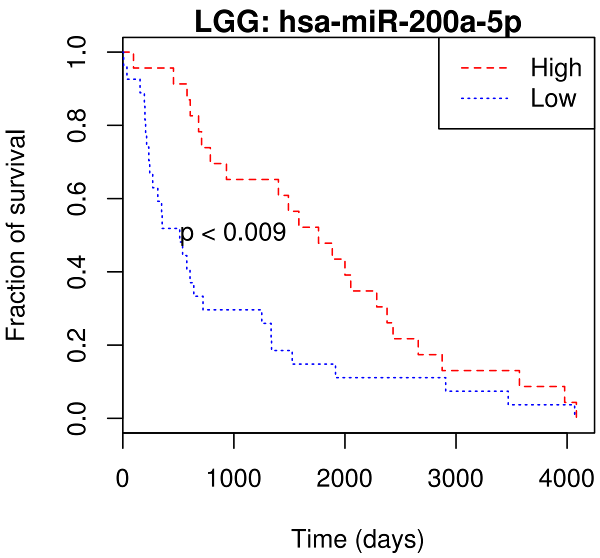
LGG: hsa-miR-200a-5p
High
Low
p < 0.009
Fraction of survival
0.0
0.2
0.4
0.6
0.8
1.0
0
1000
2000
3000
4000
Time (days)

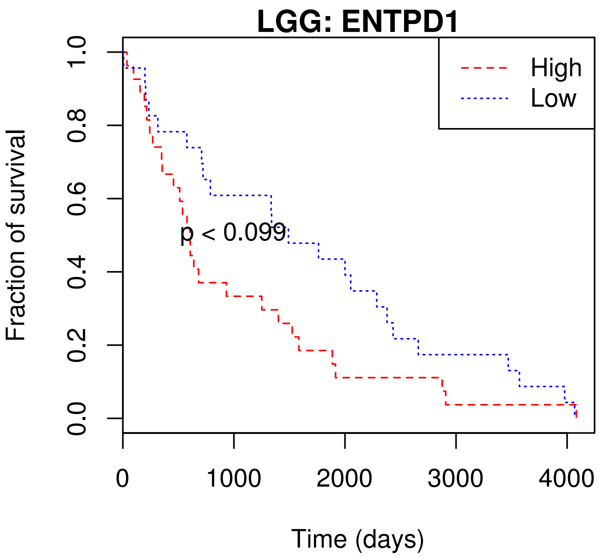
LGG: ENTPD1
High
Low
p < 0.099
Fraction of survival
0.0
0.2
0.4
0.6
0.8
1.0
0
1000
2000
3000
4000
Time (days)

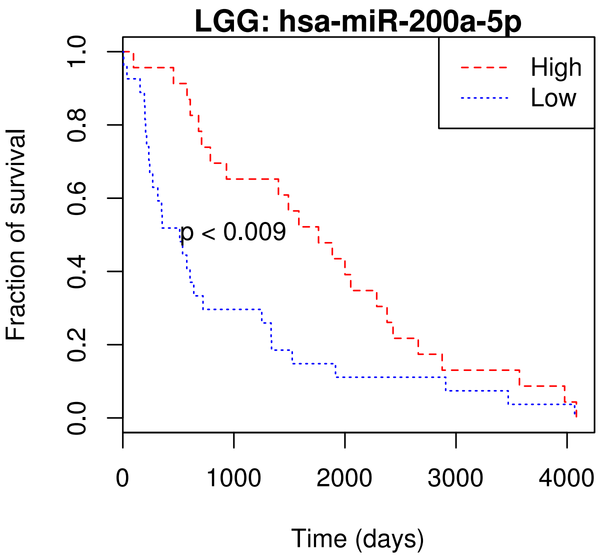
LGG: hsa-miR-200a-5p
High
Low
p < 0.009
Fraction of survival
0.0
0.2
0.4
0.6
0.8
1.0
0
1000
2000
3000
4000
Time (days)

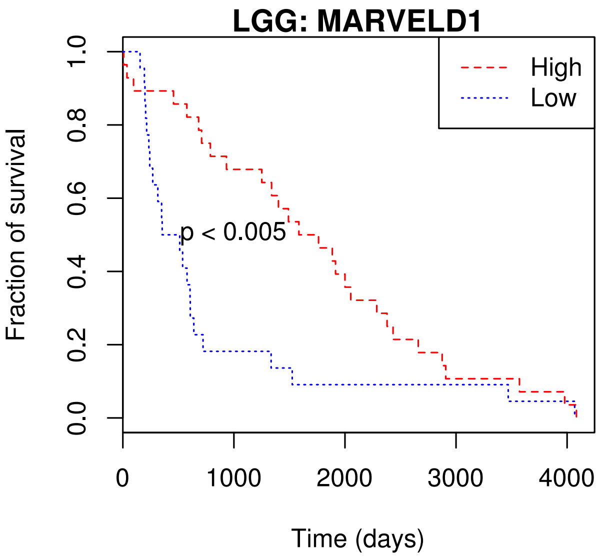
LGG: MARVELD1
High
Low
p < 0.005
Fraction of survival
0.0
0.2
0.4
0.6
0.8
1.0
0
1000
2000
3000
4000
Time (days)

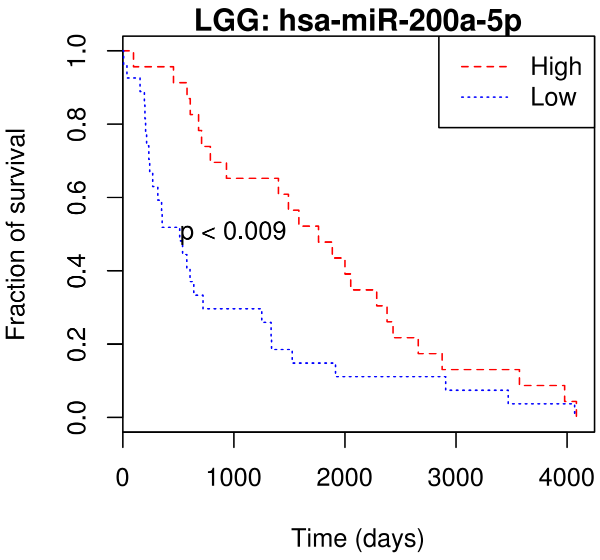
LGG: hsa-miR-200a-5p
High
Low
p < 0.009
Fraction of survival
0.0
0.2
0.4
0.6
0.8
1.0
0
1000
2000
3000
4000
Time (days)

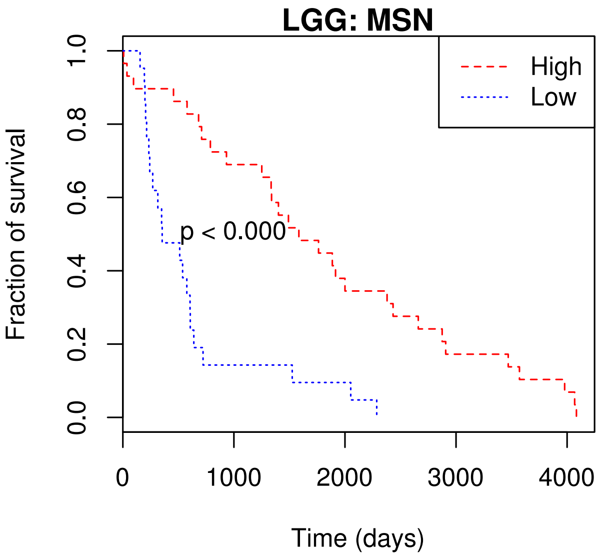
LGG: MSN
High
Low
p < 0.000
Fraction of survival
0.0
0.2
0.4
0.6
0.8
1.0
0
1000
2000
3000
4000
Time (days)

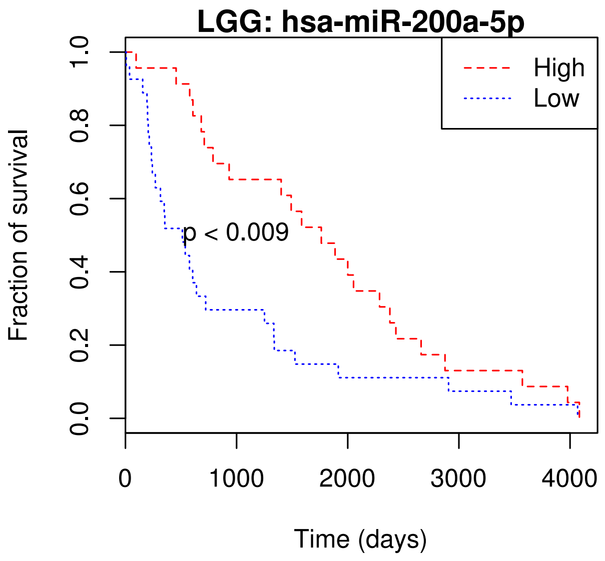
LGG: hsa-miR-200a-5p
High
Low
p < 0.009
Fraction of survival
Time (days)
0.0
0.2
0.4
0.6
0.8
1.0
0
1000
2000
3000
4000

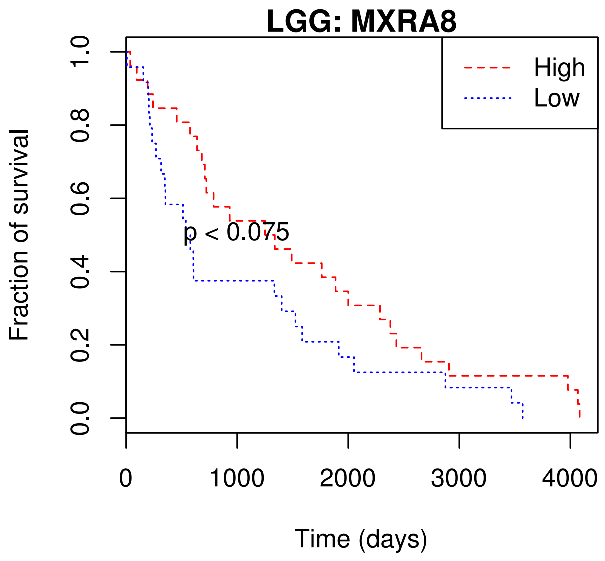
LGG: MXRA8
High
Low
p < 0.075
Fraction of survival
Time (days)
0.0
0.2
0.4
0.6
0.8
1.0
0
1000
2000
3000
4000

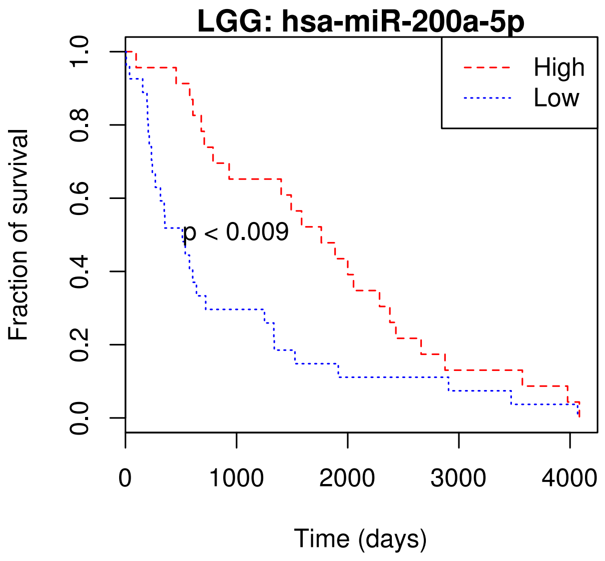
LGG: hsa-miR-200a-5p
High
Low
p < 0.009
Fraction of survival
Time (days)
0.0
0.2
0.4
0.6
0.8
1.0
0
1000
2000
3000
4000

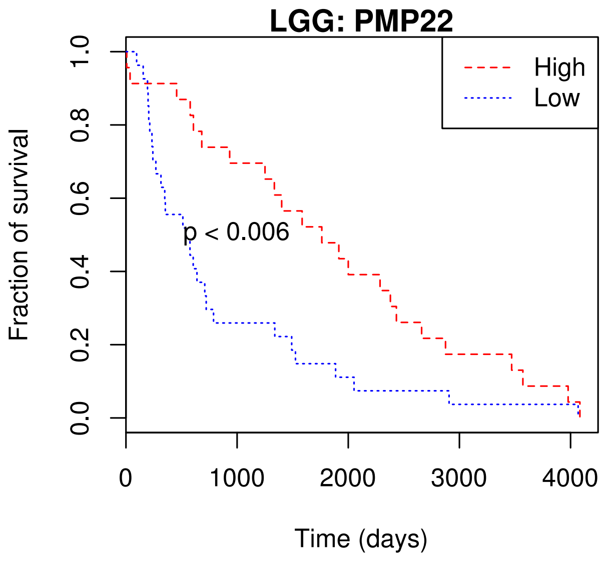
LGG: PMP22
High
Low
p < 0.006
Fraction of survival
Time (days)
0.0
0.2
0.4
0.6
0.8
1.0
0
1000
2000
3000
4000

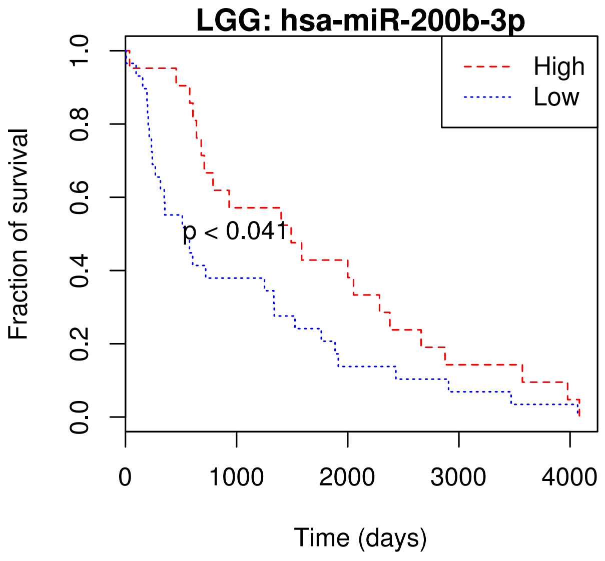
LGG: hsa-miR-200b-3p
High
Low
p < 0.041
Fraction of survival
Time (days)
0.0
0.2
0.4
0.6
0.8
1.0
0
1000
2000
3000
4000

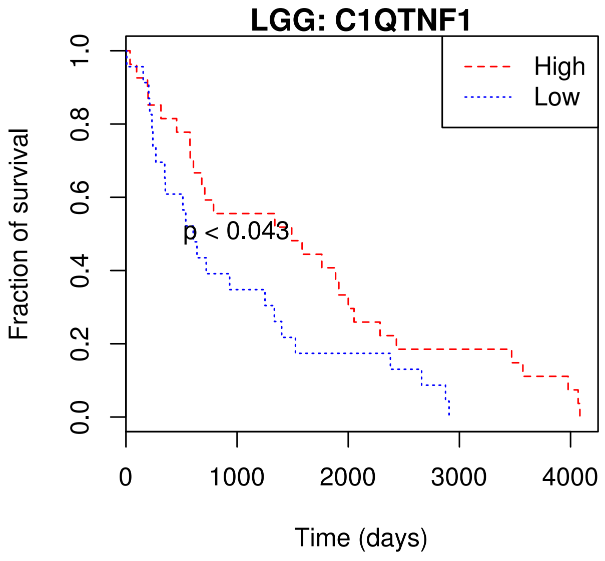
LGG: C1QTNF1
High
Low
p < 0.043
Fraction of survival
Time (days)
0.0
0.2
0.4
0.6
0.8
1.0
0
1000
2000
3000
4000

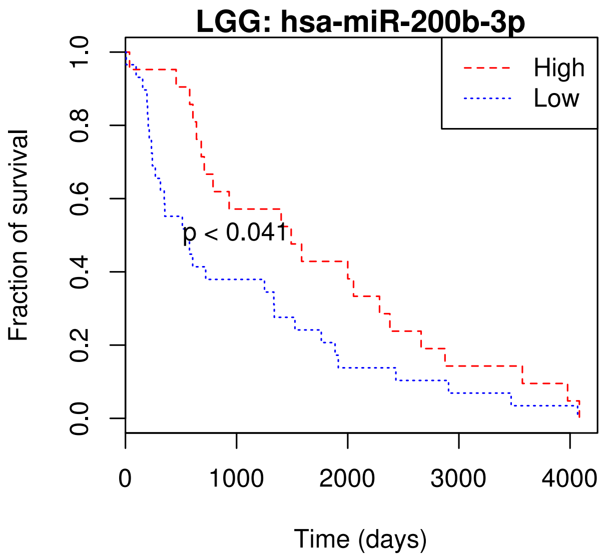
LGG: hsa-miR-200b-3p
High
Low
p < 0.041
Fraction of survival
Time (days)

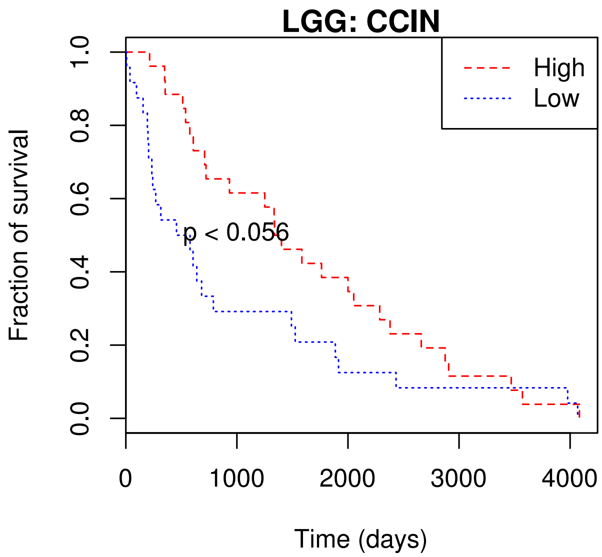
LGG: CCIN
High
Low
p < 0.056
Fraction of survival
Time (days)

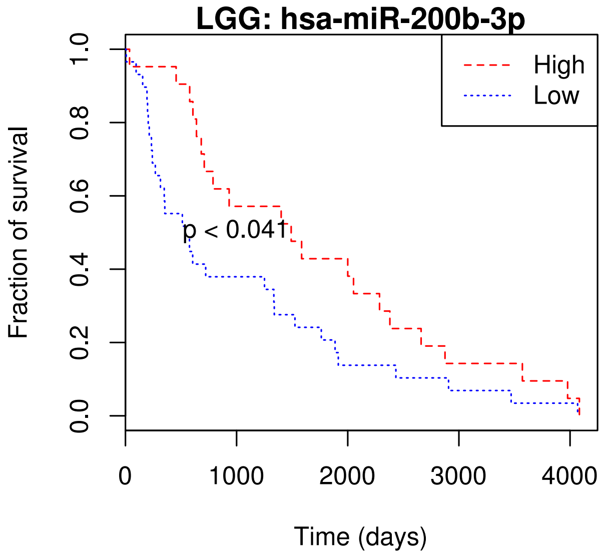
LGG: hsa-miR-200b-3p
High
Low
p < 0.041
Fraction of survival
Time (days)

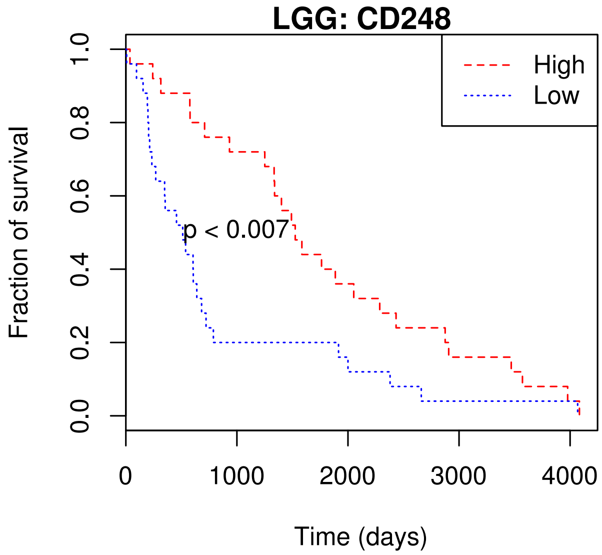
LGG: CD248
High
Low
p < 0.007
Fraction of survival
Time (days)

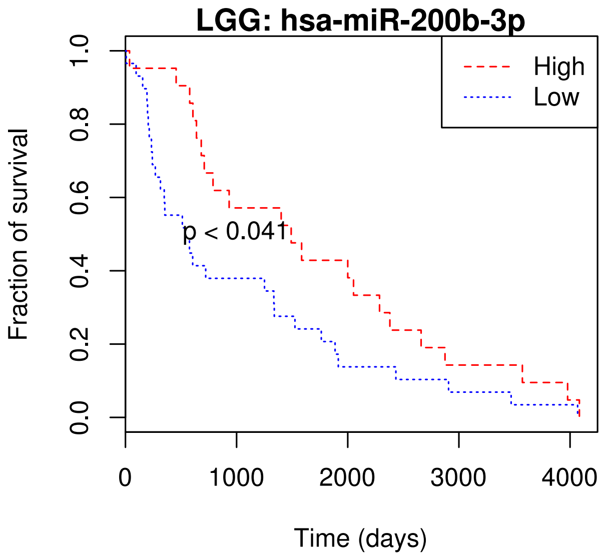
LGG: hsa-miR-200b-3p
High
Low
p < 0.041
Fraction of survival
Time (days)

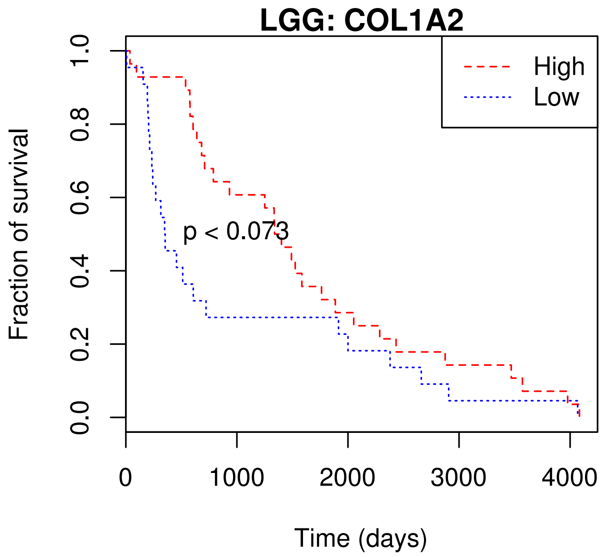
LGG: COL1A2
High
Low
p < 0.073
Fraction of survival
Time (days)

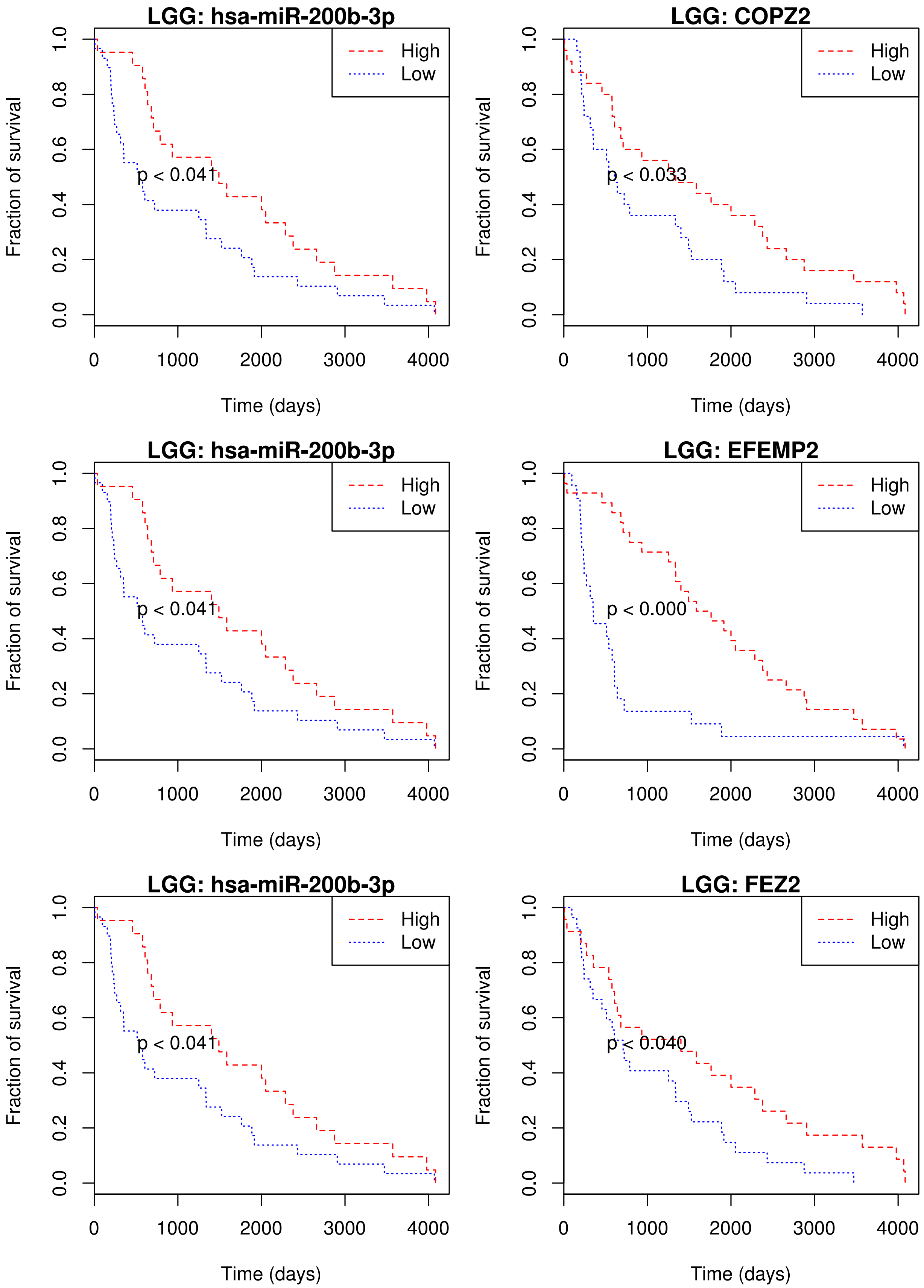

LGG: hsa-miR-200b-3p
High
Low
p < 0.041
Fraction of survival
Time (days)
0.0
0.2
0.4
0.6
0.8
1.0
0
1000
2000
3000
4000
LGG: COPZ2
High
Low
p < 0.033
Fraction of survival
Time (days)
LGG: hsa-miR-200b-3p
High
Low
p < 0.041
Fraction of survival
Time (days)
LGG: EFEMP2
High
Low
p < 0.000
Fraction of survival
Time (days)
LGG: hsa-miR-200b-3p
High
Low
p < 0.041
Fraction of survival
Time (days)
LGG: FEZ2
High
Low
p < 0.040
Fraction of survival
Time (days)

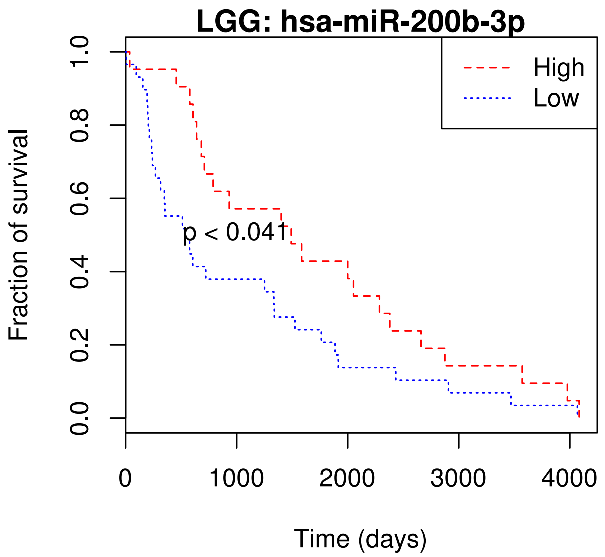

LGG: hsa-miR-200b-3p
High
Low
p < 0.041
Fraction of survival
Time (days)
0.0
0.2
0.4
0.6
0.8
1.0
0
1000
2000
3000
4000

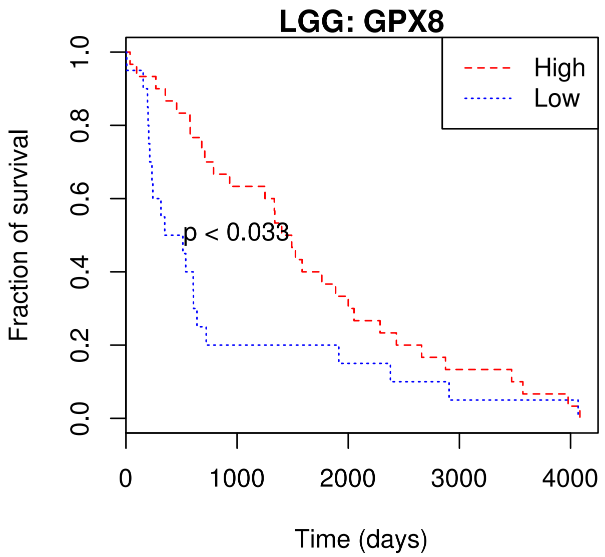

LGG: GPX8
High
Low
p < 0.033
Fraction of survival
Time (days)
0.0
0.2
0.4
0.6
0.8
1.0
0
1000
2000
3000
4000

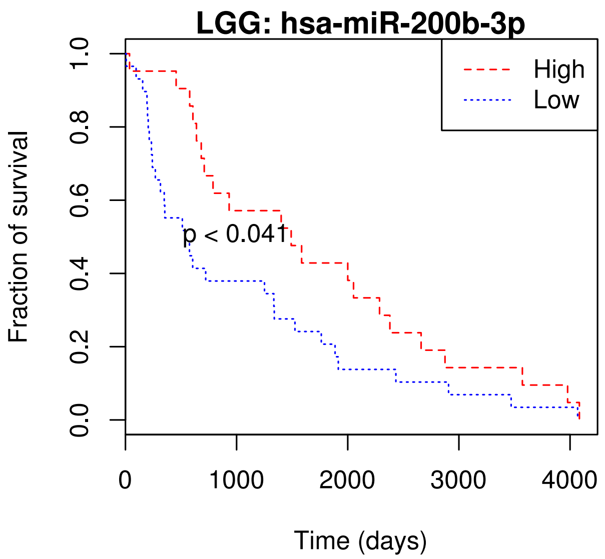

LGG: hsa-miR-200b-3p
High
Low
p < 0.041
Fraction of survival
Time (days)
0.0
0.2
0.4
0.6
0.8
1.0
0
1000
2000
3000
4000

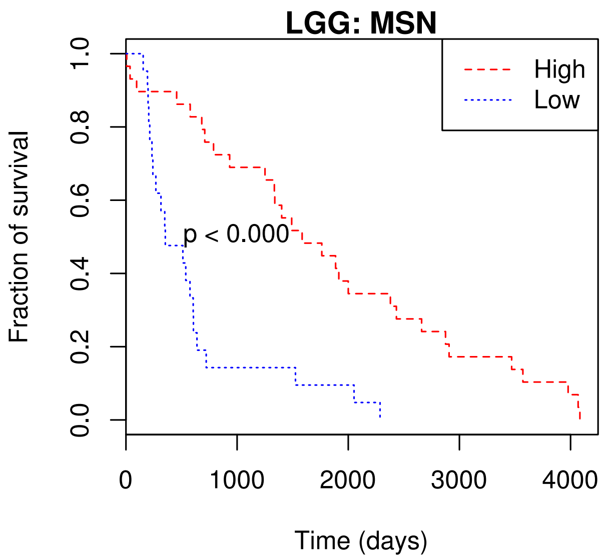

LGG: MSN
High
Low
p < 0.000
Fraction of survival
Time (days)
0.0
0.2
0.4
0.6
0.8
1.0
0
1000
2000
3000
4000

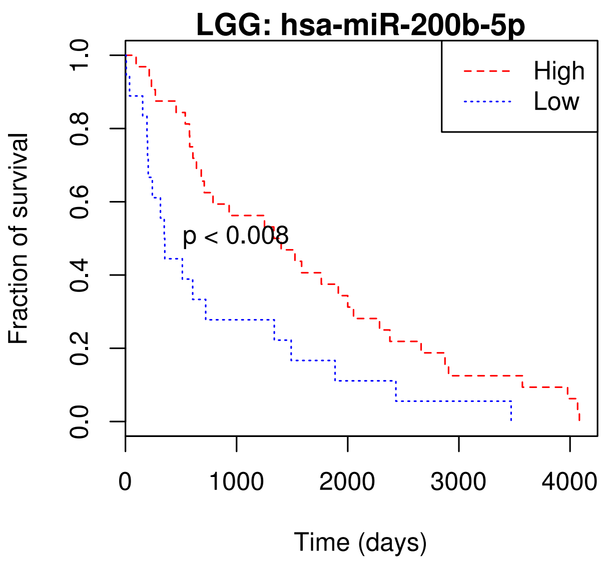

LGG: hsa-miR-200b-5p
High
Low
p < 0.008
Fraction of survival
Time (days)
0.0
0.2
0.4
0.6
0.8
1.0
0
1000
2000
3000
4000

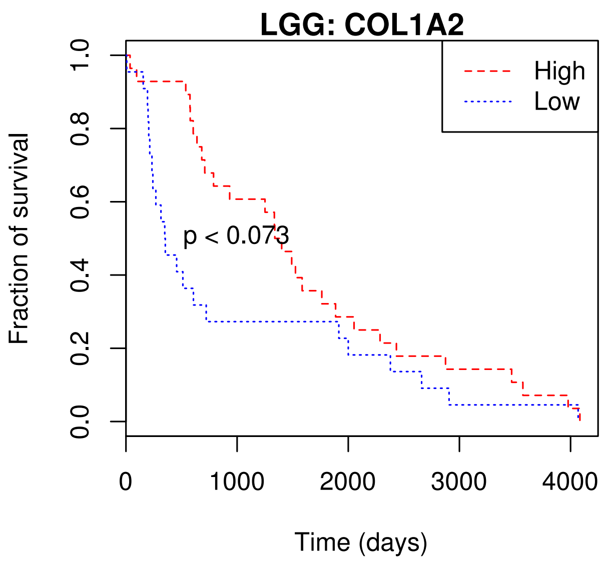

LGG: COL1A2
High
Low
p < 0.073
Fraction of survival
Time (days)
0.0
0.2
0.4
0.6
0.8
1.0
0
1000
2000
3000
4000

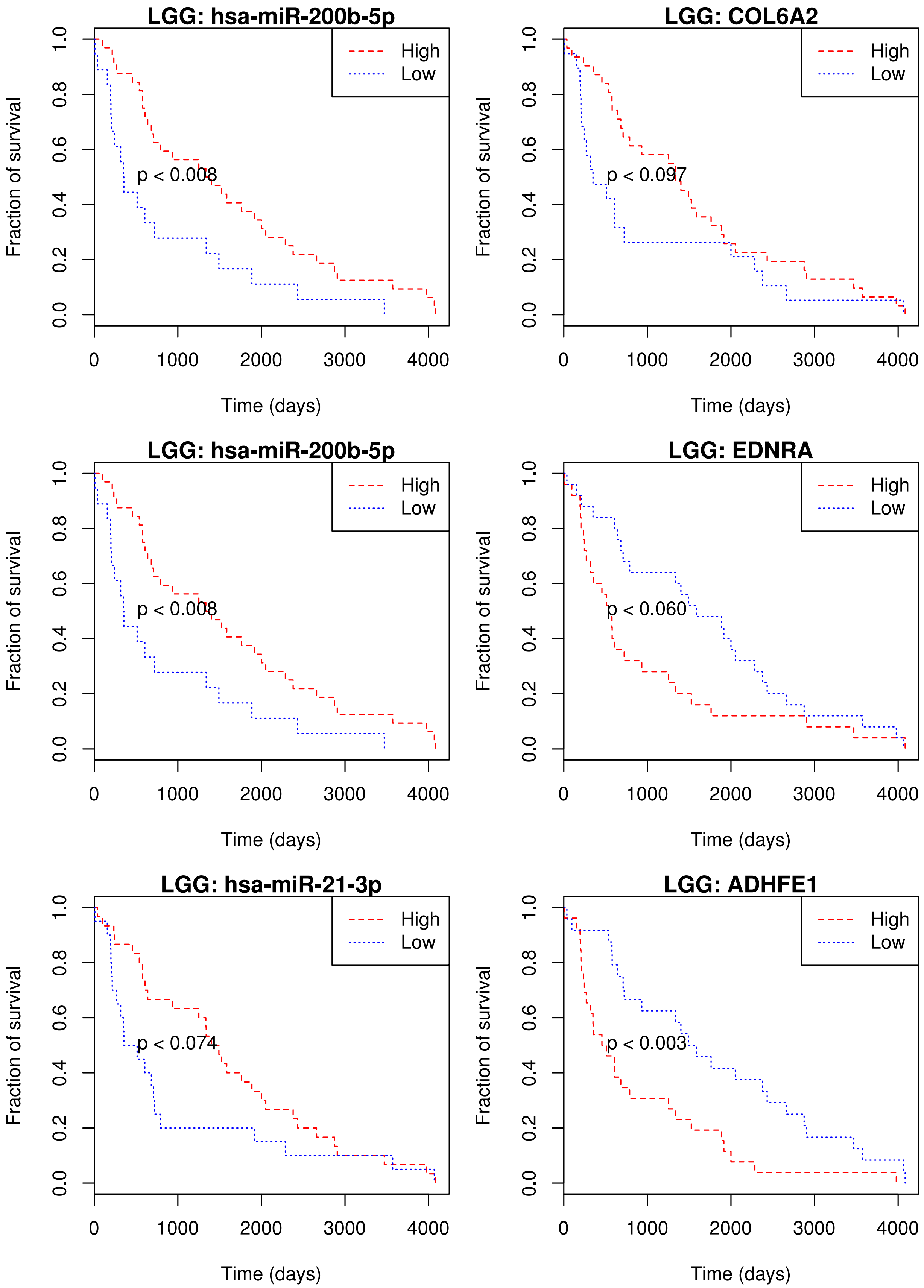
LGG: hsa-miR-200b-5p
High
Low
p < 0.008
Fraction of survival
Time (days)
LGG: COL6A2
High
Low
p < 0.097
Fraction of survival
Time (days)
LGG: hsa-miR-200b-5p
High
Low
p < 0.008
Fraction of survival
Time (days)
LGG: EDNRA
High
Low
p < 0.060
Fraction of survival
Time (days)
LGG: hsa-miR-21-3p
High
Low
p < 0.074
Fraction of survival
Time (days)
LGG: ADHFE1
High
Low
p < 0.003
Fraction of survival
Time (days)

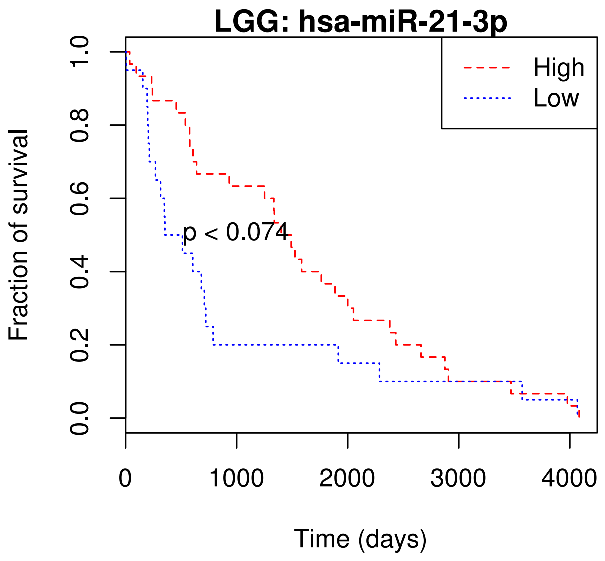
LGG: hsa-miR-21-3p
High
Low
p < 0.074
Fraction of survival
0.0 0.2 0.4 0.6 0.8 1.0
0 1000 2000 3000 4000
Time (days)

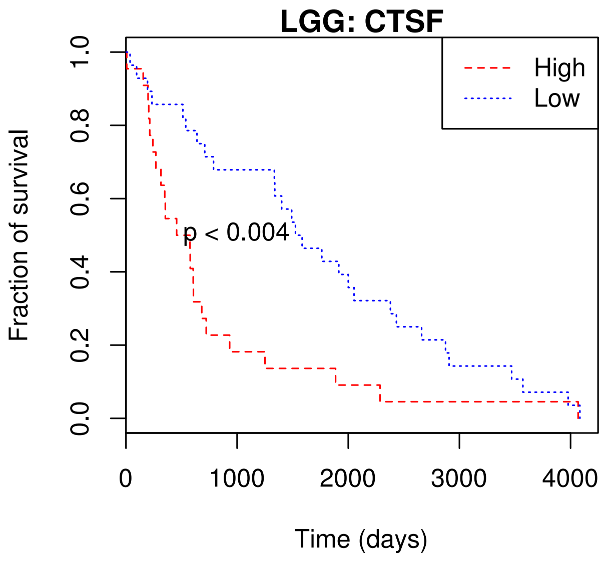
LGG: CTSF
High
Low
p < 0.004
Fraction of survival
0.0 0.2 0.4 0.6 0.8 1.0
0 1000 2000 3000 4000
Time (days)

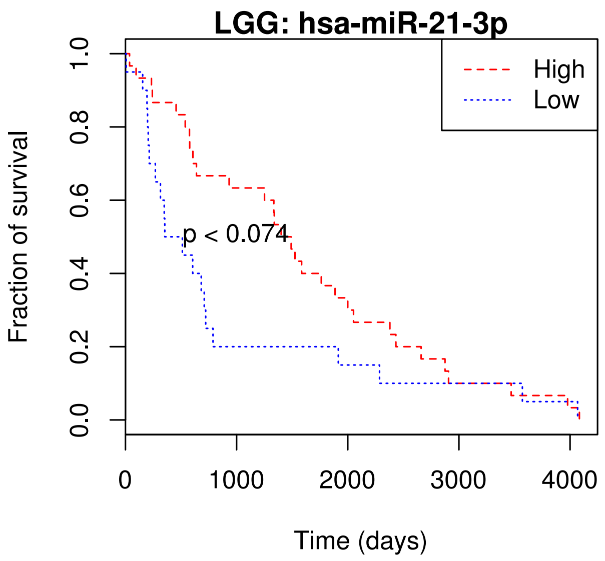
LGG: hsa-miR-21-3p
High
Low
p < 0.074
Fraction of survival
0.0 0.2 0.4 0.6 0.8 1.0
0 1000 2000 3000 4000
Time (days)

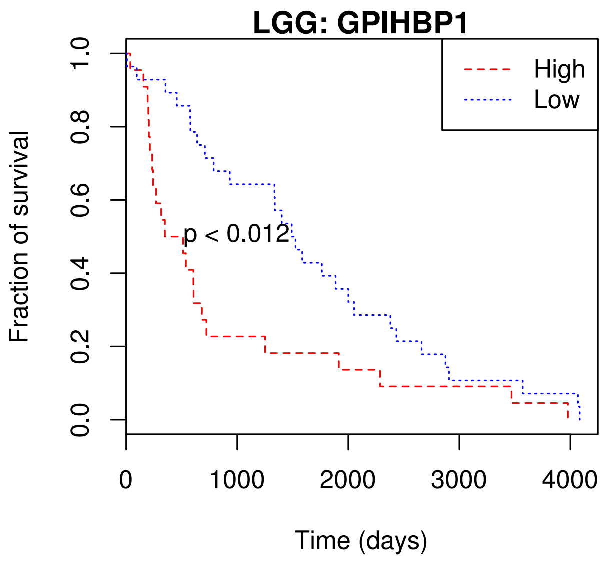
LGG: GPIHBP1
High
Low
p < 0.012
Fraction of survival
0.0 0.2 0.4 0.6 0.8 1.0
0 1000 2000 3000 4000
Time (days)

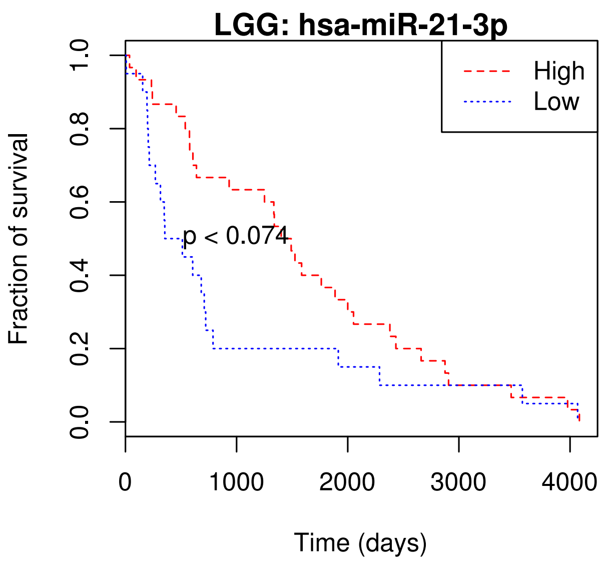
LGG: hsa-miR-21-3p
High
Low
p < 0.074
Fraction of survival
0.0 0.2 0.4 0.6 0.8 1.0
0 1000 2000 3000 4000
Time (days)

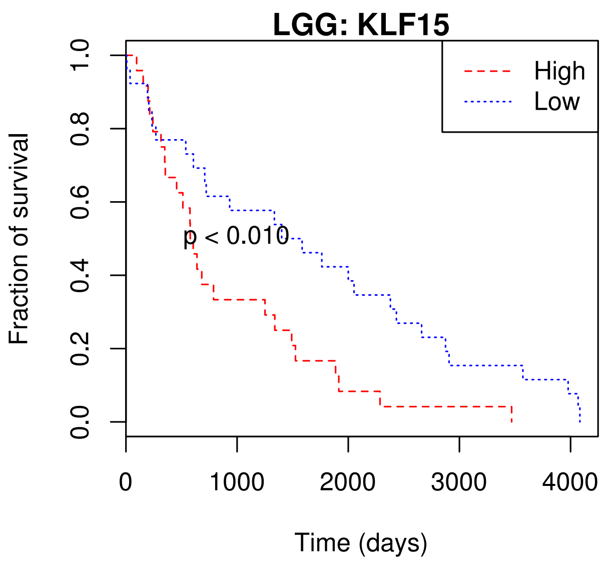
LGG: KLF15
High
Low
p < 0.010
Fraction of survival
0.0 0.2 0.4 0.6 0.8 1.0
0 1000 2000 3000 4000
Time (days)

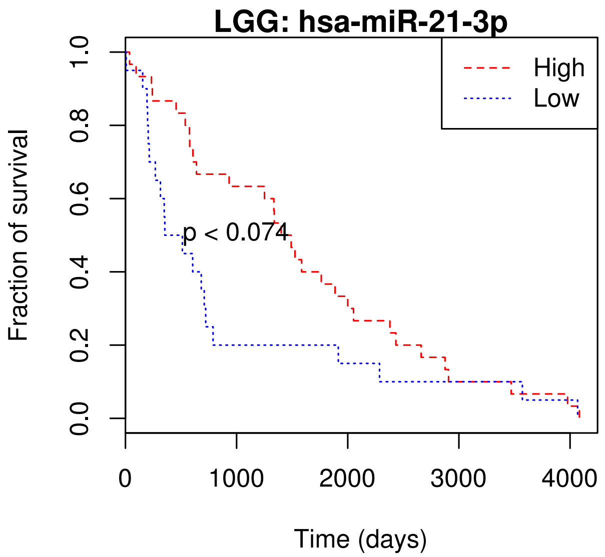
LGG: hsa-miR-21-3p
High
Low
p < 0.074
Fraction of survival
Time (days)
0.0 0.2 0.4 0.6 0.8 1.0
0 1000 2000 3000 4000

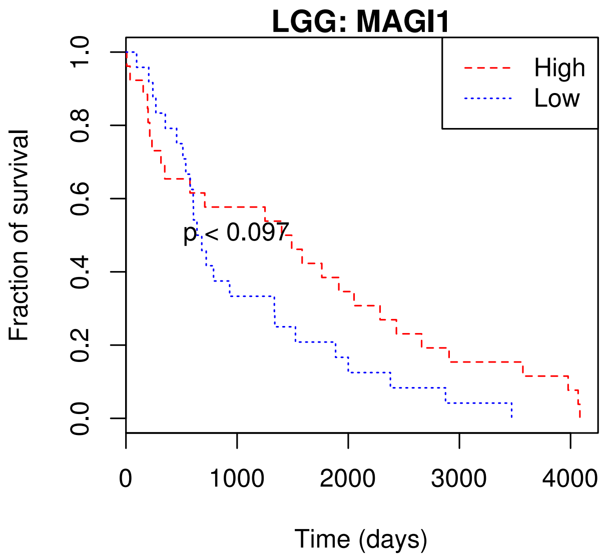
LGG: MAGI1
High
Low
p < 0.097
Fraction of survival
Time (days)
0.0 0.2 0.4 0.6 0.8 1.0
0 1000 2000 3000 4000

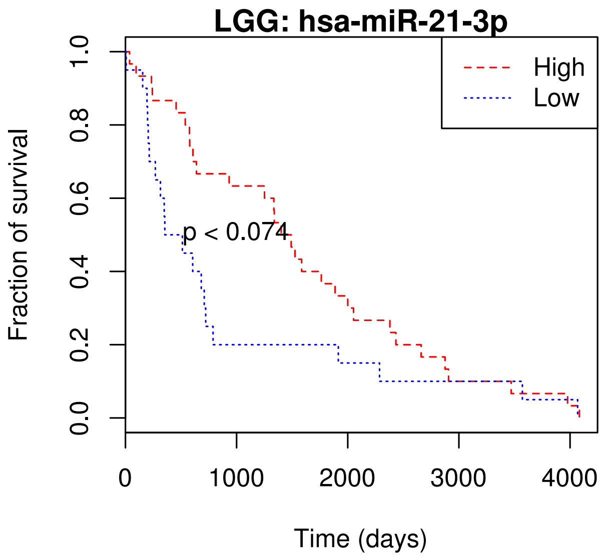
LGG: hsa-miR-21-3p
High
Low
p < 0.074
Fraction of survival
Time (days)
0.0 0.2 0.4 0.6 0.8 1.0
0 1000 2000 3000 4000

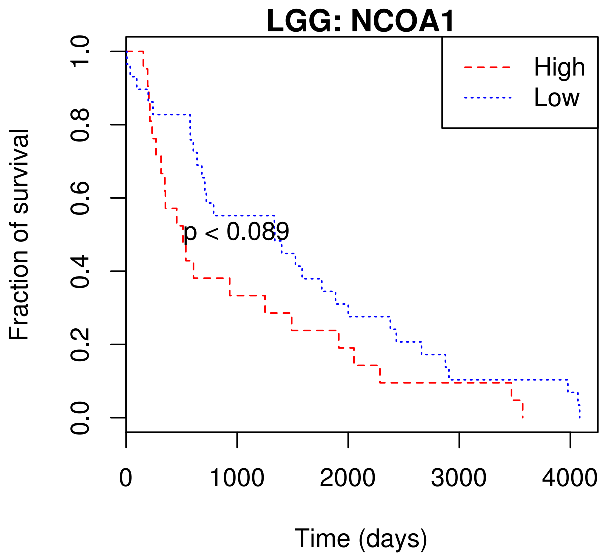
LGG: NCOA1
High
Low
p < 0.089
Fraction of survival
Time (days)
0.0 0.2 0.4 0.6 0.8 1.0
0 1000 2000 3000 4000

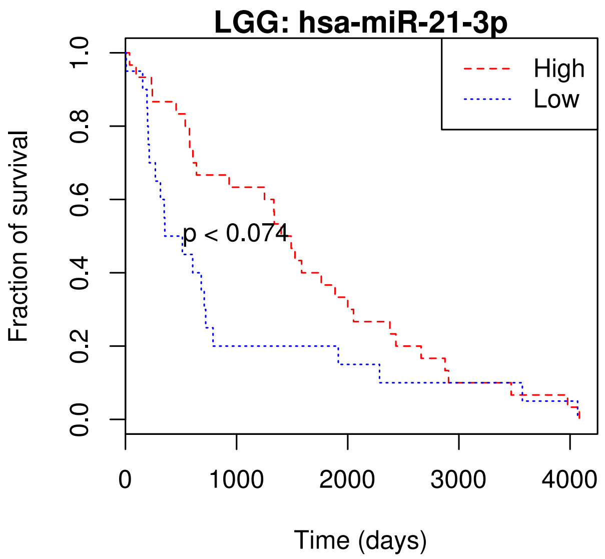
LGG: hsa-miR-21-3p
High
Low
p < 0.074
Fraction of survival
Time (days)
0.0 0.2 0.4 0.6 0.8 1.0
0 1000 2000 3000 4000

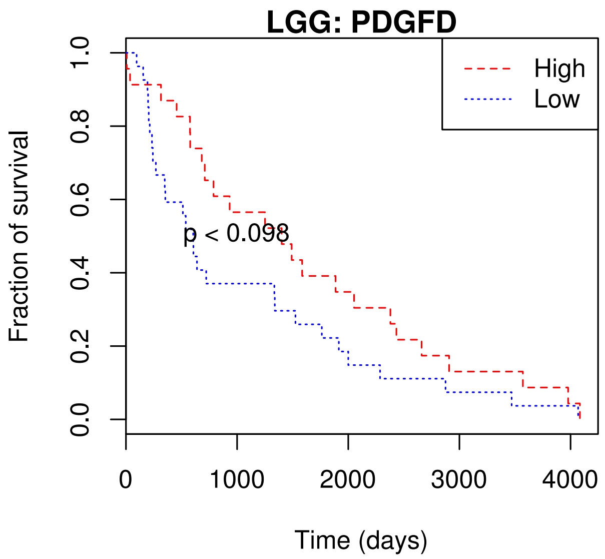
LGG: PDGFD
High
Low
p < 0.098
Fraction of survival
Time (days)
0.0 0.2 0.4 0.6 0.8 1.0
0 1000 2000 3000 4000

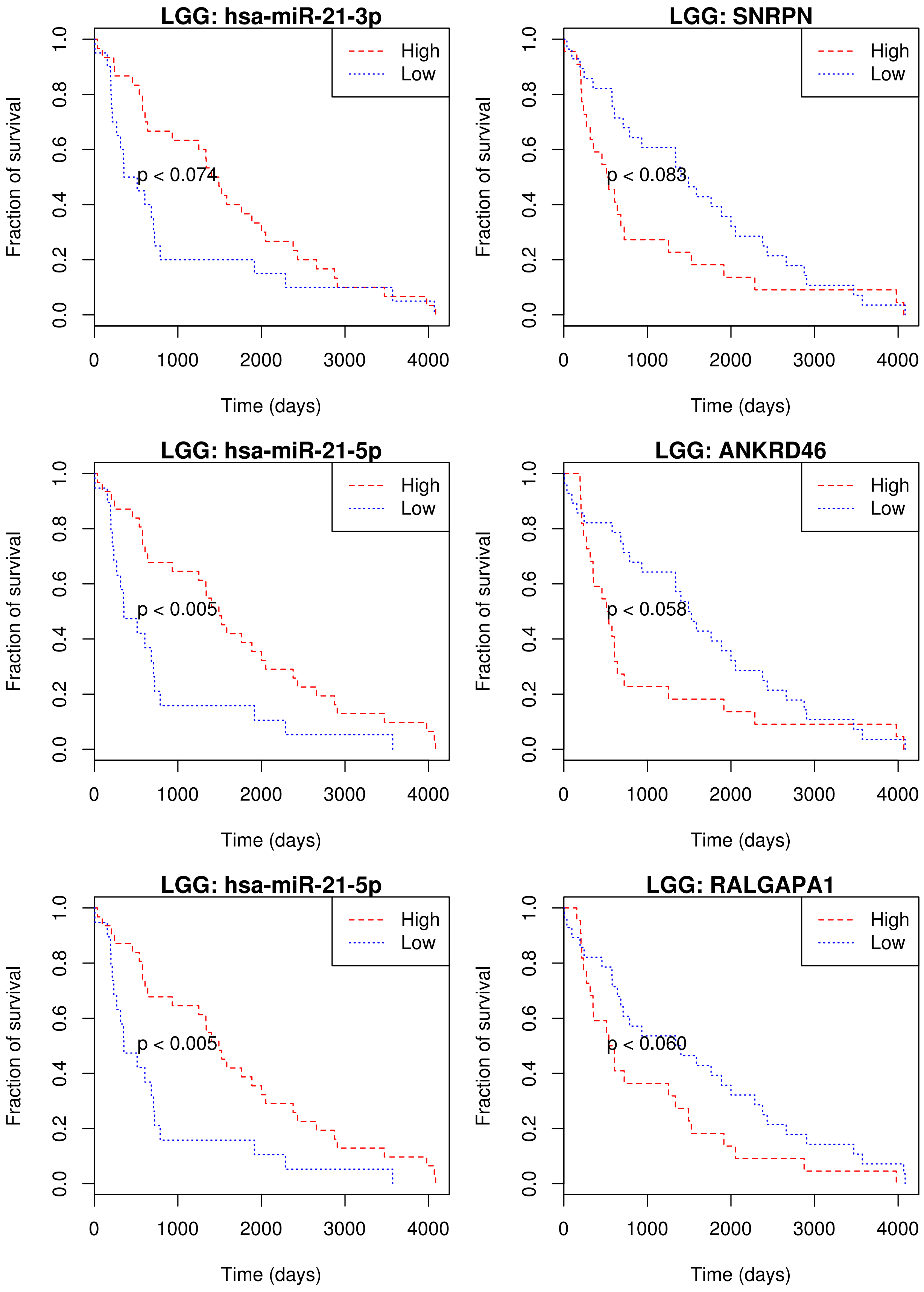
LGG: hsa-miR-21-3p
p < 0.074
LGG: SNRPN
p < 0.083
LGG: hsa-miR-21-5p
p < 0.005
LGG: ANKRD46
p < 0.058
LGG: hsa-miR-21-5p
p < 0.005
LGG: RALGAPA1
p < 0.060
High
Low
Fraction of survival
0.0
0.2
0.4
0.6
0.8
1.0
Time (days)
0
1000
2000
3000
4000

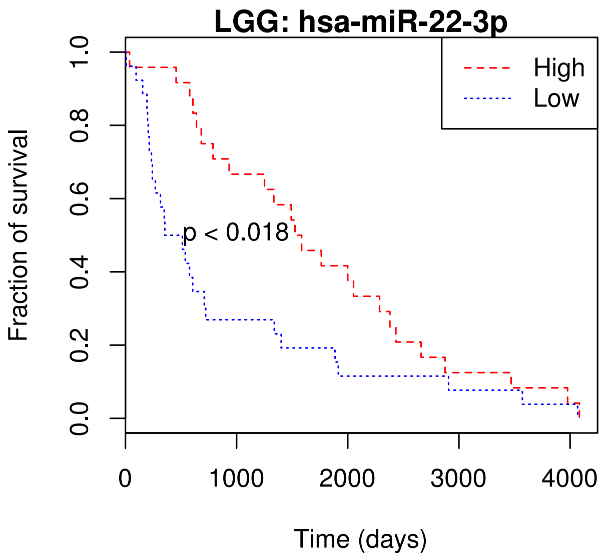
LGG: hsa-miR-22-3p
High
Low
p < 0.018
Fraction of survival
0.0
0.2
0.4
0.6
0.8
1.0
0
1000
2000
3000
4000
Time (days)

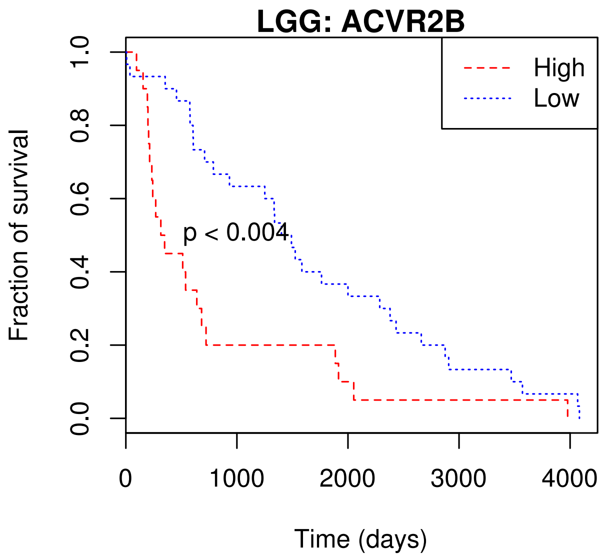
LGG: ACVR2B
High
Low
p < 0.004
Fraction of survival
0.0
0.2
0.4
0.6
0.8
1.0
0
1000
2000
3000
4000
Time (days)

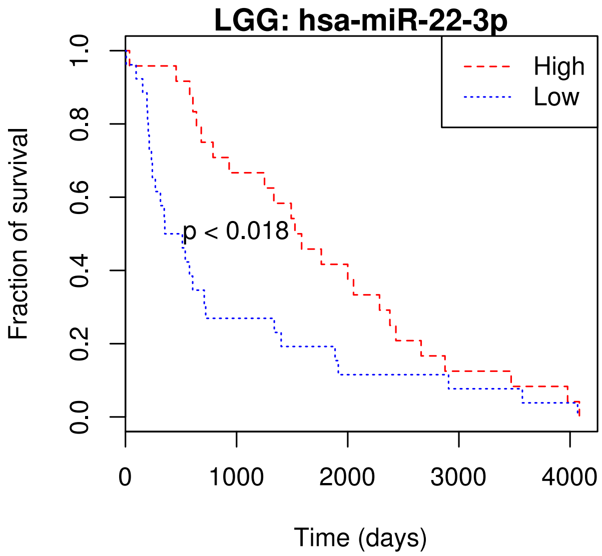
LGG: hsa-miR-22-3p
High
Low
p < 0.018
Fraction of survival
0.0
0.2
0.4
0.6
0.8
1.0
0
1000
2000
3000
4000
Time (days)

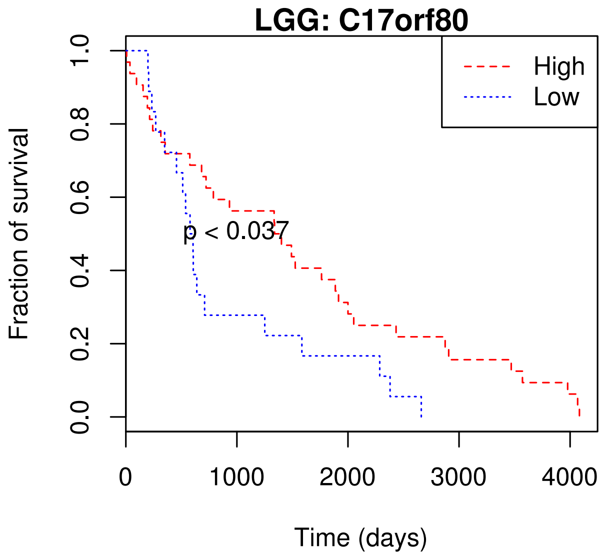
LGG: C17orf80
High
Low
p < 0.037
Fraction of survival
0.0
0.2
0.4
0.6
0.8
1.0
0
1000
2000
3000
4000
Time (days)

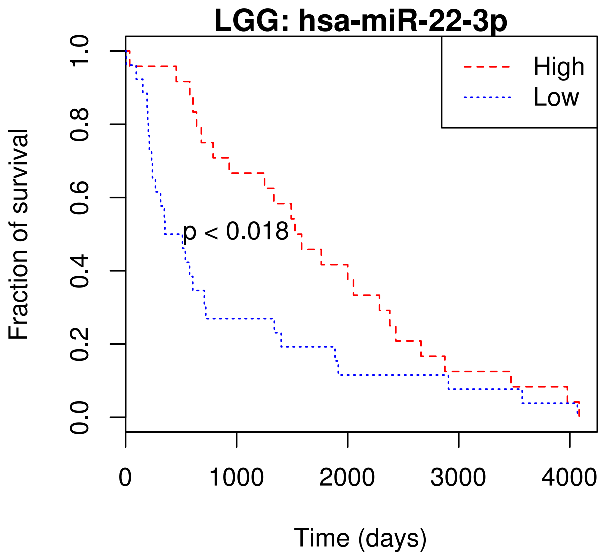
LGG: hsa-miR-22-3p
High
Low
p < 0.018
Fraction of survival
0.0
0.2
0.4
0.6
0.8
1.0
0
1000
2000
3000
4000
Time (days)

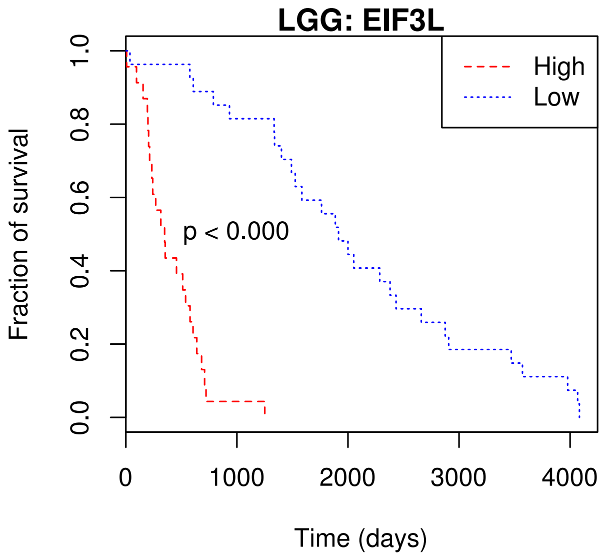
LGG: EIF3L
High
Low
p < 0.000
Fraction of survival
0.0
0.2
0.4
0.6
0.8
1.0
0
1000
2000
3000
4000
Time (days)

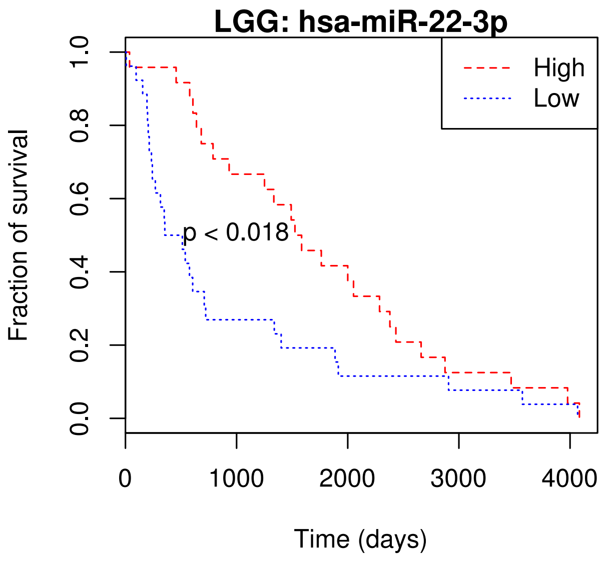
LGG: hsa-miR-22-3p
High
Low
p < 0.018
Fraction of survival
0.0
0.2
0.4
0.6
0.8
1.0
0
1000
2000
3000
4000
Time (days)

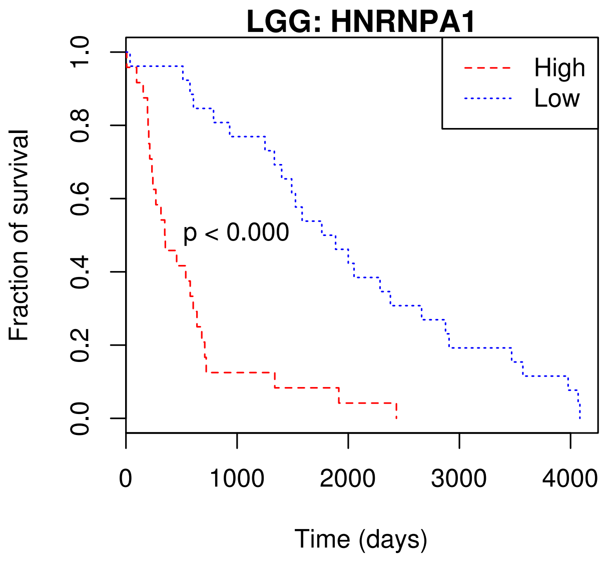
LGG: HNRNPA1
High
Low
p < 0.000
Fraction of survival
0.0
0.2
0.4
0.6
0.8
1.0
0
1000
2000
3000
4000
Time (days)

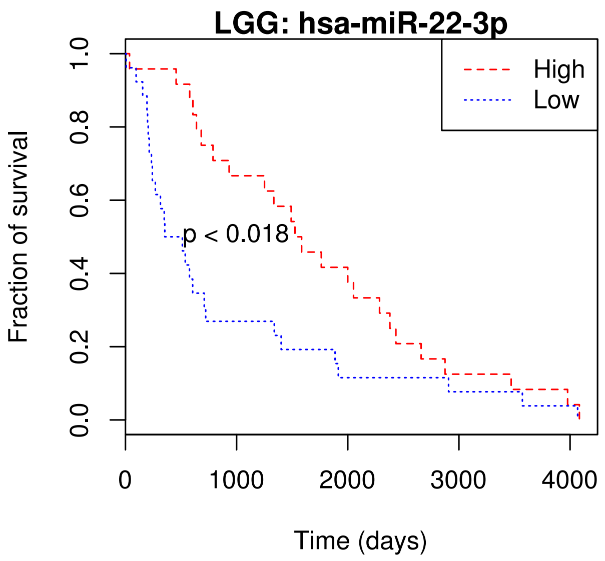
LGG: hsa-miR-22-3p
High
Low
p < 0.018
Fraction of survival
0.0
0.2
0.4
0.6
0.8
1.0
0
1000
2000
3000
4000
Time (days)

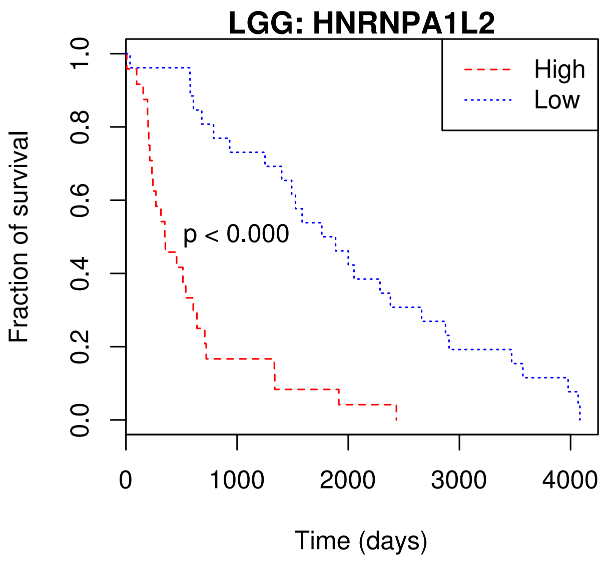
LGG: HNRNPA1L2
High
Low
p < 0.000
Fraction of survival
0.0
0.2
0.4
0.6
0.8
1.0
0
1000
2000
3000
4000
Time (days)

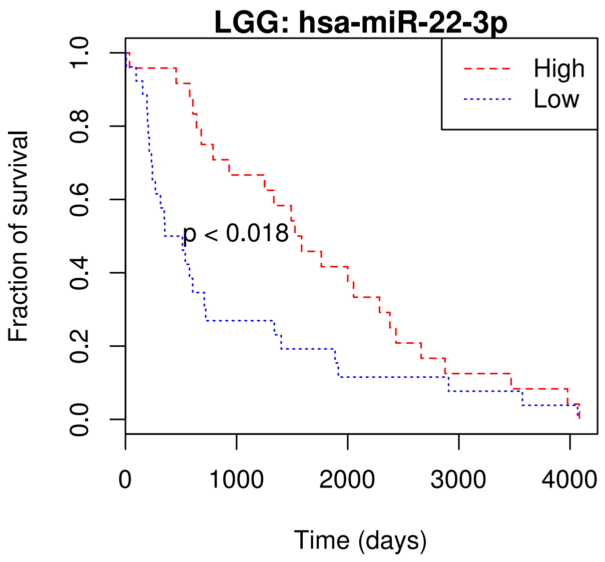
LGG: hsa-miR-22-3p
High
Low
p < 0.018
Fraction of survival
0.0
0.2
0.4
0.6
0.8
1.0
0
1000
2000
3000
4000
Time (days)

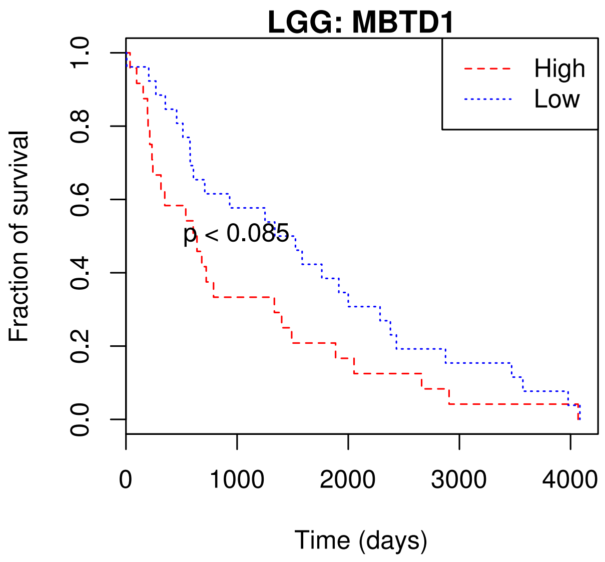
LGG: MBTD1
High
Low
p < 0.085
Fraction of survival
0.0
0.2
0.4
0.6
0.8
1.0
0
1000
2000
3000
4000
Time (days)

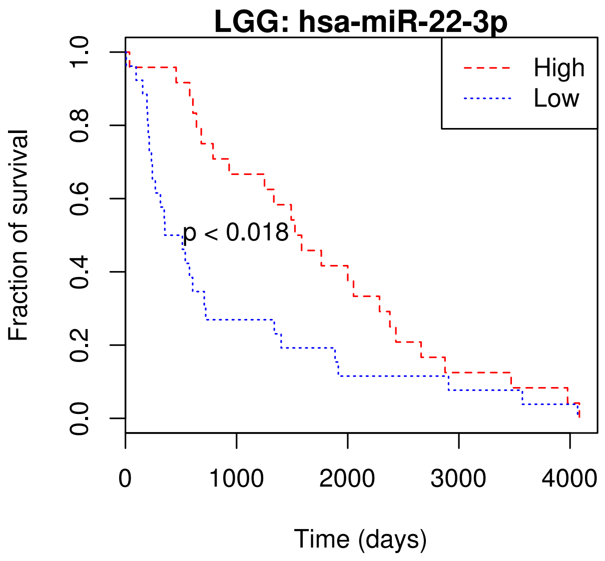
LGG: hsa-miR-22-3p
High
Low
p < 0.018
Fraction of survival
Time (days)

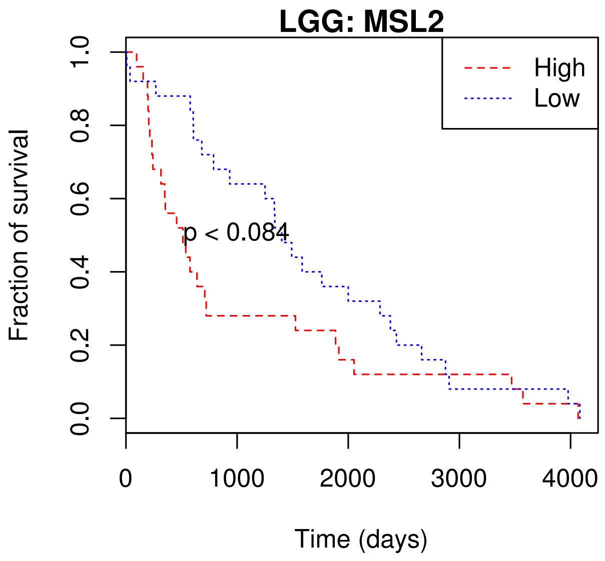
LGG: MSL2
High
Low
p < 0.084
Fraction of survival
Time (days)

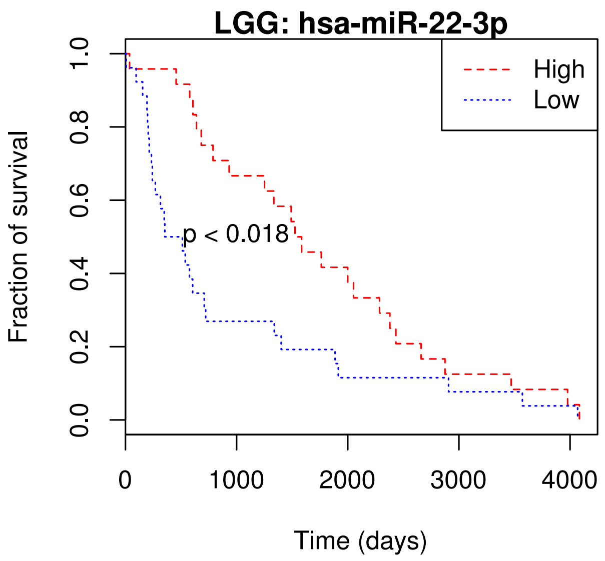
LGG: hsa-miR-22-3p
High
Low
p < 0.018
Fraction of survival
Time (days)

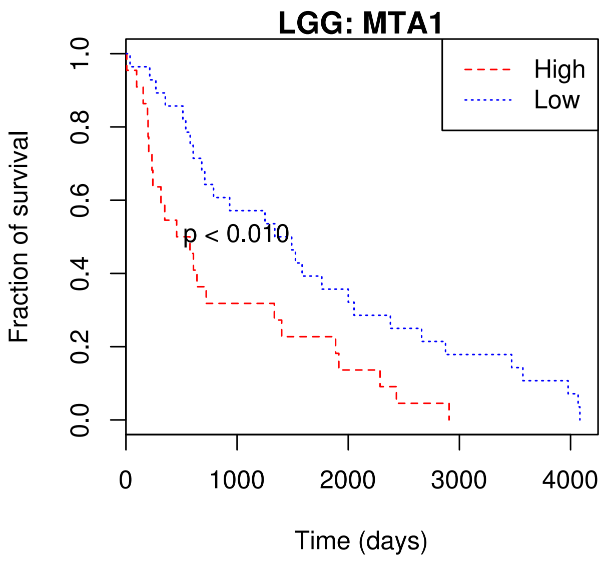
LGG: MTA1
High
Low
p < 0.010
Fraction of survival
Time (days)

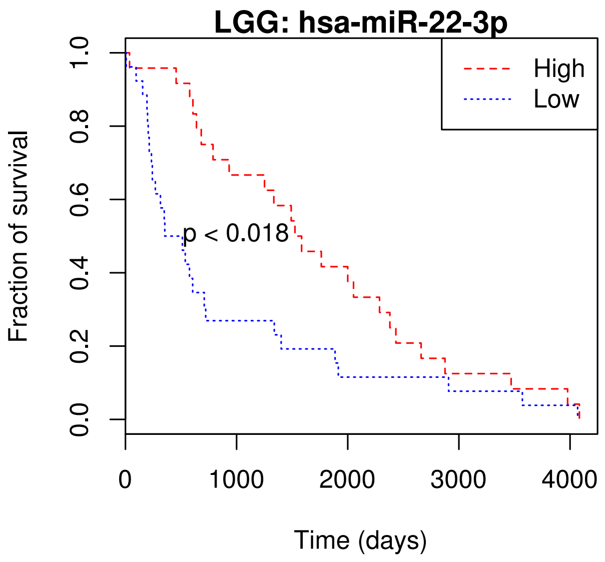
LGG: hsa-miR-22-3p
High
Low
p < 0.018
Fraction of survival
Time (days)

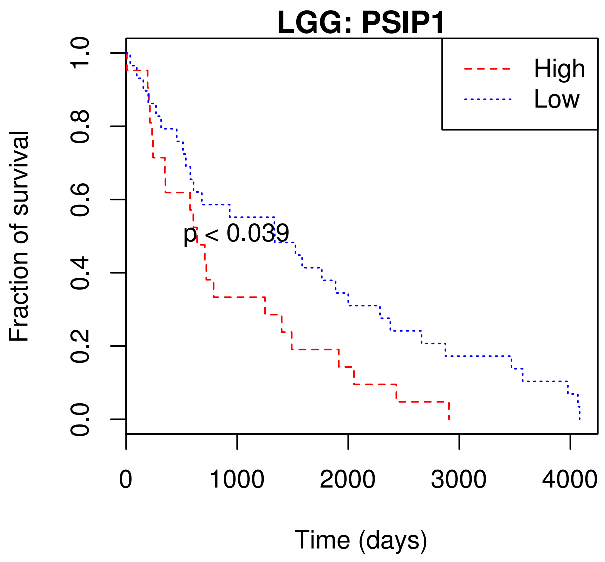
LGG: PSIP1
High
Low
p < 0.039
Fraction of survival
Time (days)

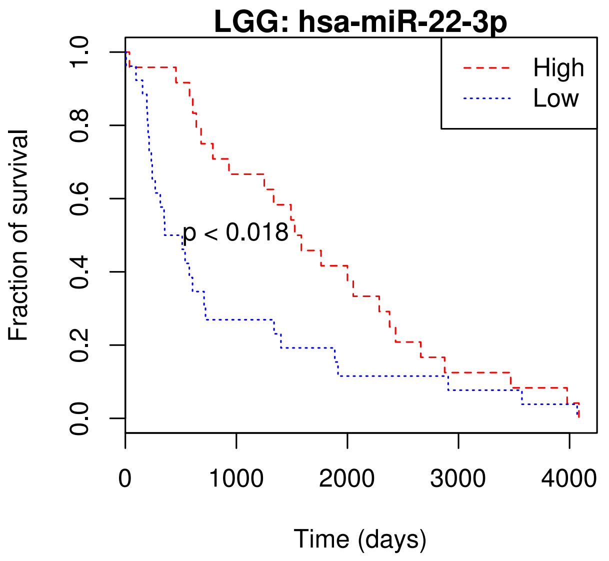
LGG: hsa-miR-22-3p
High
Low
p < 0.018
Fraction of survival
0.0
0.2
0.4
0.6
0.8
1.0
0
1000
2000
3000
4000
Time (days)

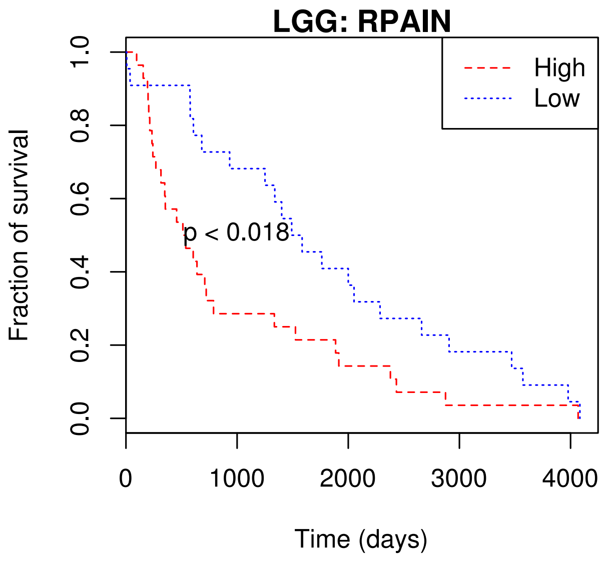
LGG: RPAIN
High
Low
p < 0.018
Fraction of survival
0.0
0.2
0.4
0.6
0.8
1.0
0
1000
2000
3000
4000
Time (days)

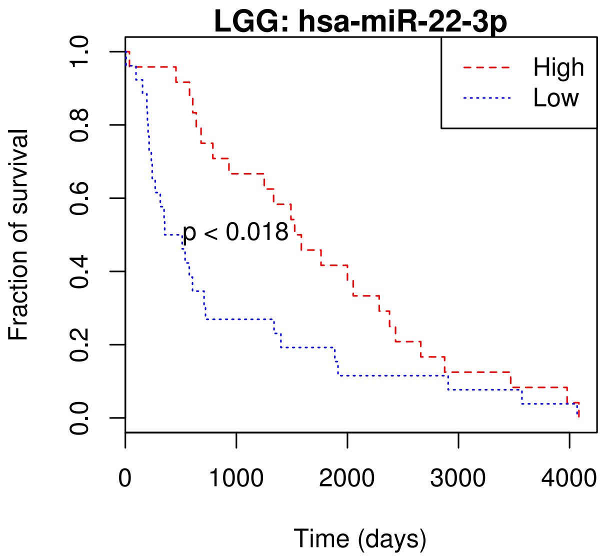
LGG: hsa-miR-22-3p
High
Low
p < 0.018
Fraction of survival
0.0
0.2
0.4
0.6
0.8
1.0
0
1000
2000
3000
4000
Time (days)

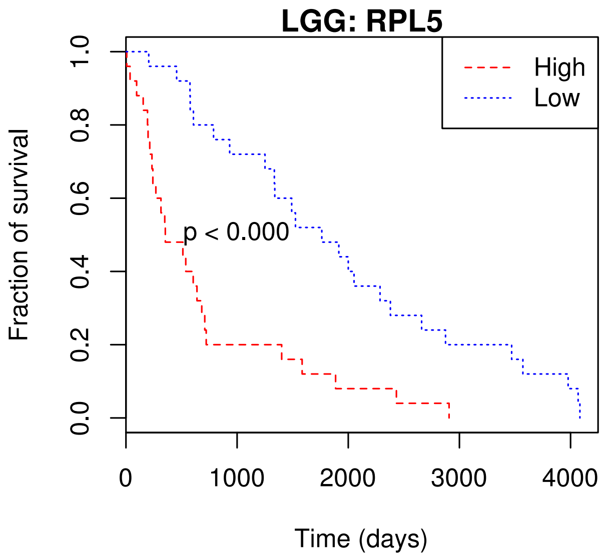
LGG: RPL5
High
Low
p < 0.000
Fraction of survival
0.0
0.2
0.4
0.6
0.8
1.0
0
1000
2000
3000
4000
Time (days)

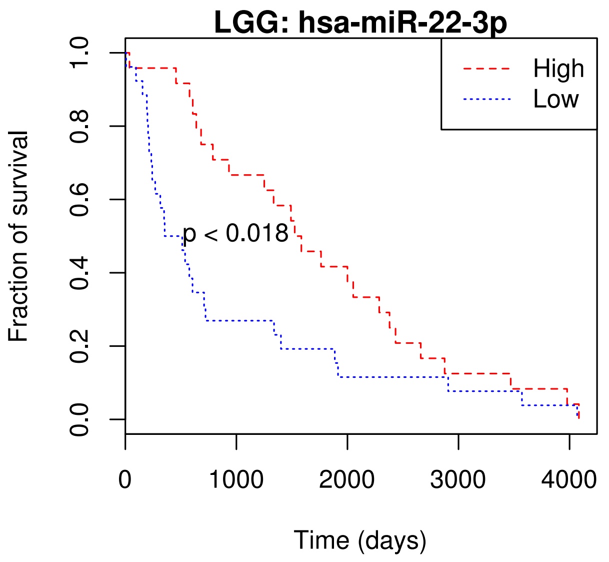
LGG: hsa-miR-22-3p
High
Low
p < 0.018
Fraction of survival
0.0
0.2
0.4
0.6
0.8
1.0
0
1000
2000
3000
4000
Time (days)

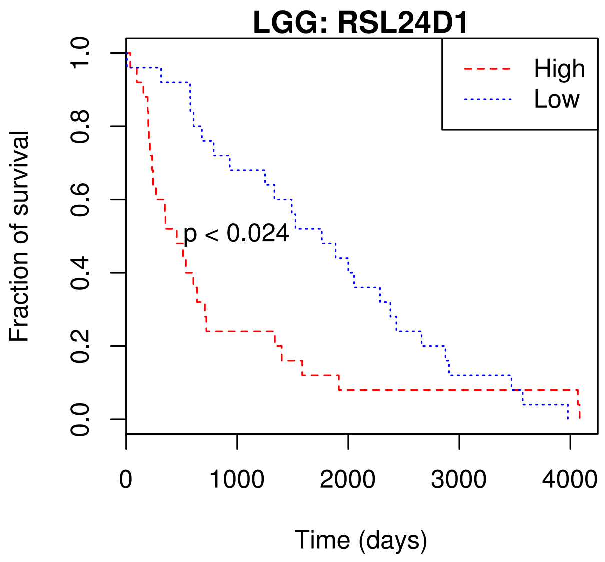
LGG: RSL24D1
High
Low
p < 0.024
Fraction of survival
0.0
0.2
0.4
0.6
0.8
1.0
0
1000
2000
3000
4000
Time (days)

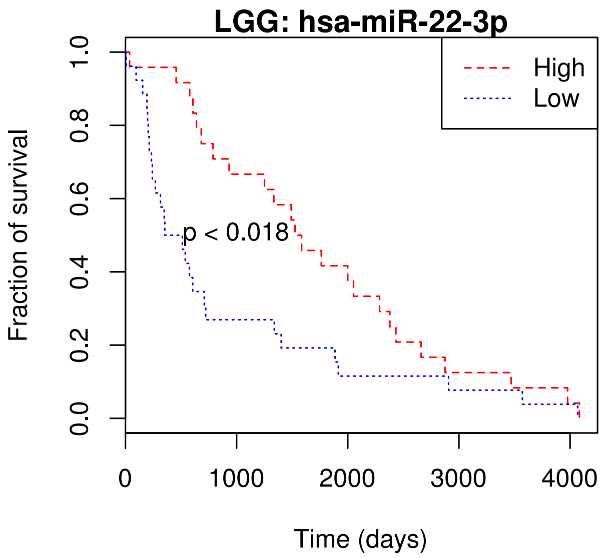
LGG: hsa-miR-22-3p
High
Low
p < 0.018
Fraction of survival
0.0
0.2
0.4
0.6
0.8
1.0
0
1000
2000
3000
4000
Time (days)

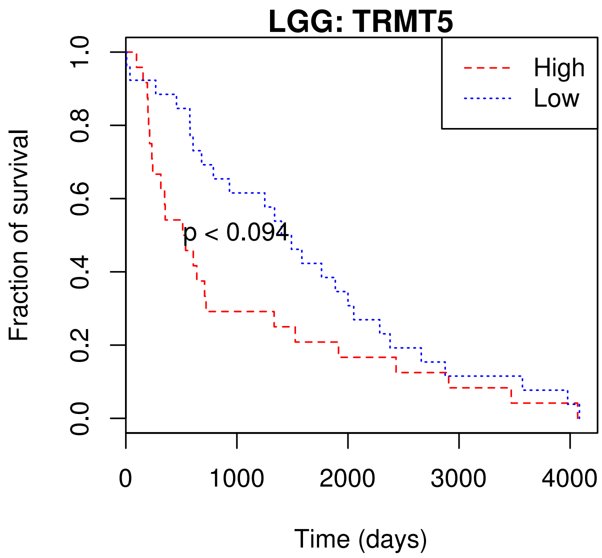
LGG: TRMT5
High
Low
p < 0.094
Fraction of survival
0.0
0.2
0.4
0.6
0.8
1.0
0
1000
2000
3000
4000
Time (days)

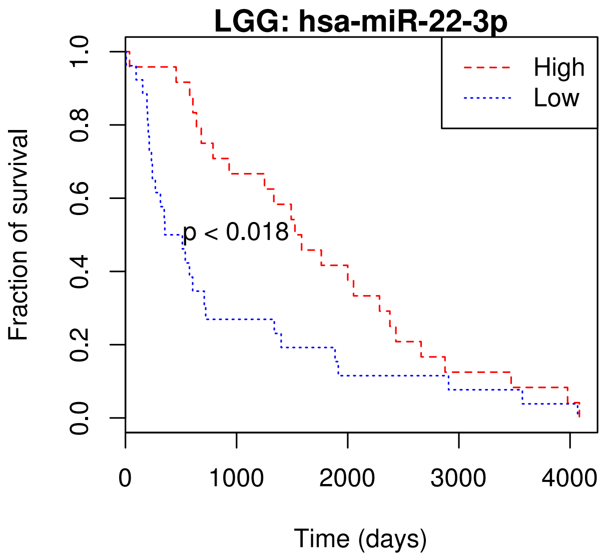
LGG: hsa-miR-22-3p
High
Low
p < 0.018
Fraction of survival
0.0
0.2
0.4
0.6
0.8
1.0
0
1000
2000
3000
4000
Time (days)

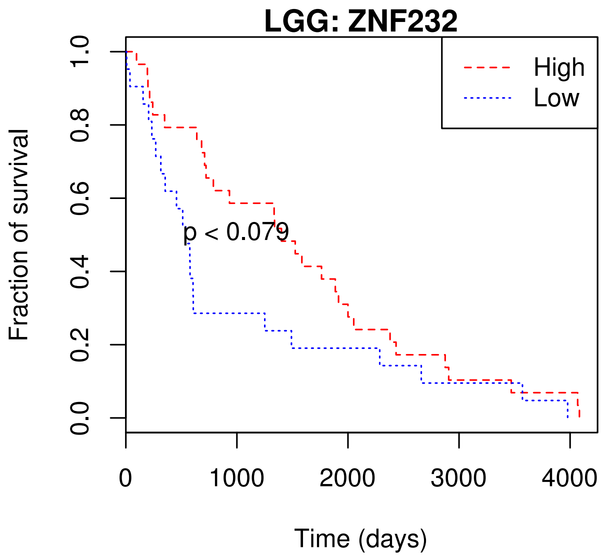
LGG: ZNF232
High
Low
p < 0.079
Fraction of survival
0.0
0.2
0.4
0.6
0.8
1.0
0
1000
2000
3000
4000
Time (days)

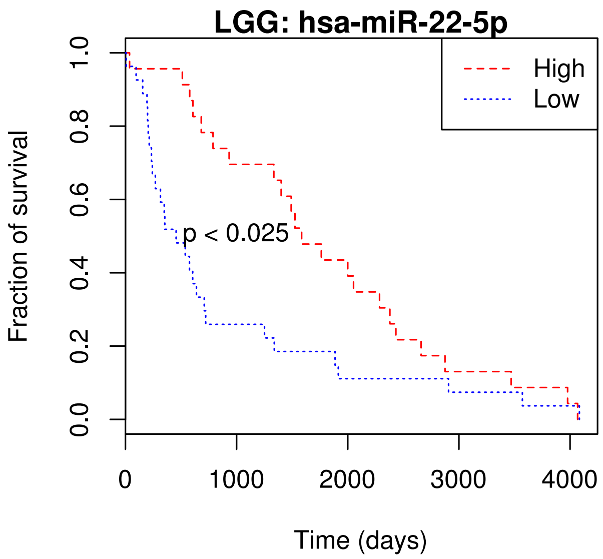
LGG: hsa-miR-22-5p
High
Low
p < 0.025
Fraction of survival
0.0
0.2
0.4
0.6
0.8
1.0
0
1000
2000
3000
4000
Time (days)

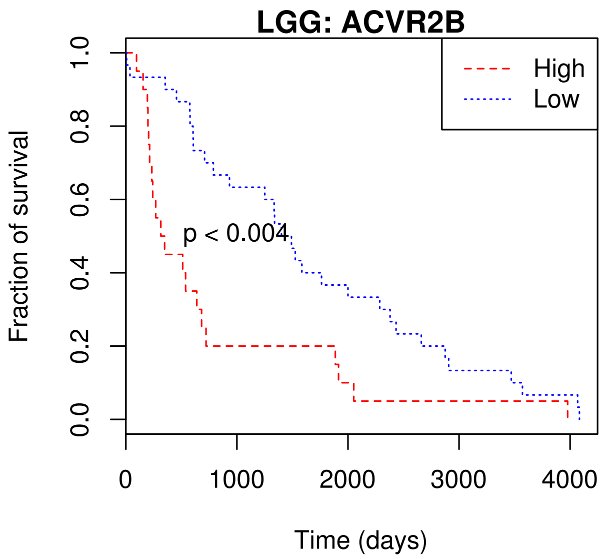
LGG: ACVR2B
High
Low
p < 0.004
Fraction of survival
0.0
0.2
0.4
0.6
0.8
1.0
0
1000
2000
3000
4000
Time (days)

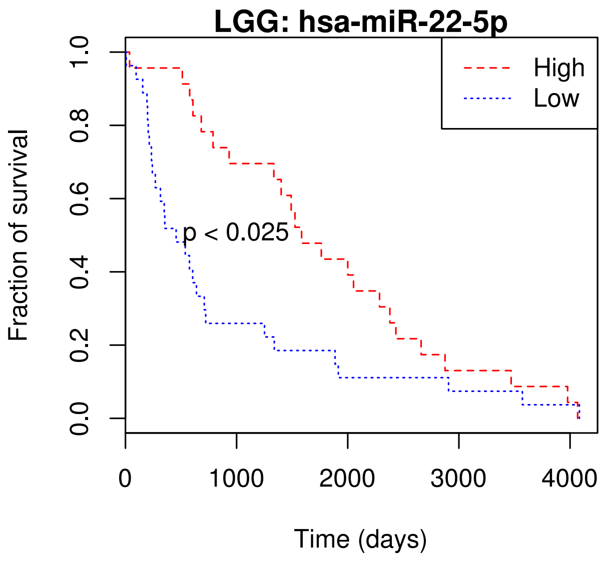
LGG: hsa-miR-22-5p
High
Low
p < 0.025
Fraction of survival
0.0
0.2
0.4
0.6
0.8
1.0
0
1000
2000
3000
4000
Time (days)

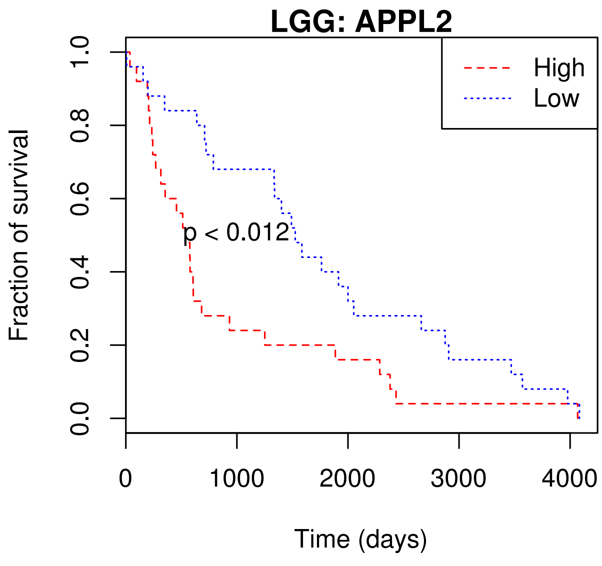
LGG: APPL2
High
Low
p < 0.012
Fraction of survival
0.0
0.2
0.4
0.6
0.8
1.0
0
1000
2000
3000
4000
Time (days)

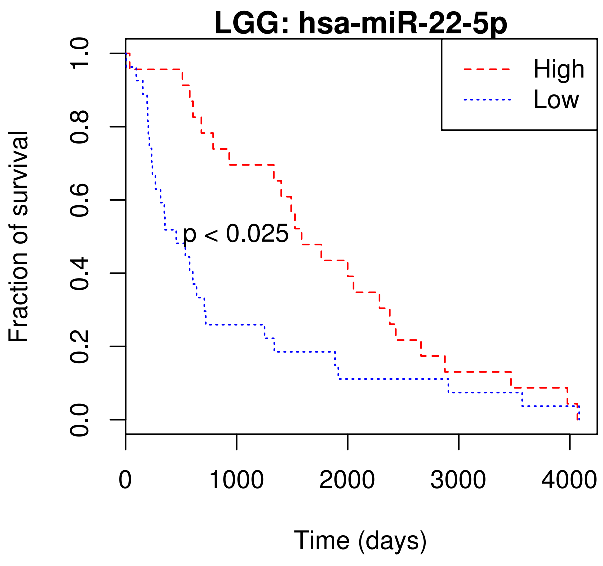
LGG: hsa-miR-22-5p
High
Low
p < 0.025
Fraction of survival
0.0
0.2
0.4
0.6
0.8
1.0
0
1000
2000
3000
4000
Time (days)

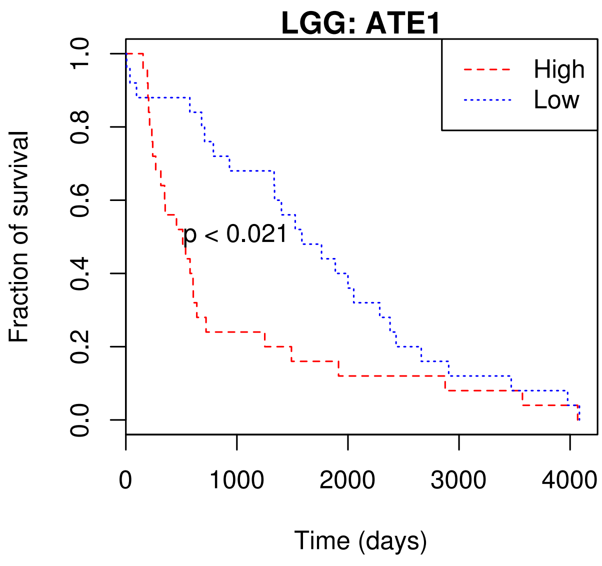
LGG: ATE1
High
Low
p < 0.021
Fraction of survival
0.0
0.2
0.4
0.6
0.8
1.0
0
1000
2000
3000
4000
Time (days)

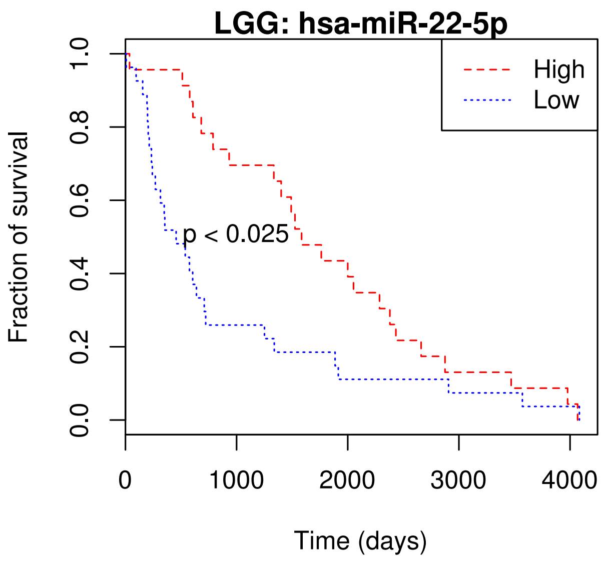
LGG: hsa-miR-22-5p
High
Low
p < 0.025
Fraction of survival
0.0
0.2
0.4
0.6
0.8
1.0
0
1000
2000
3000
4000
Time (days)

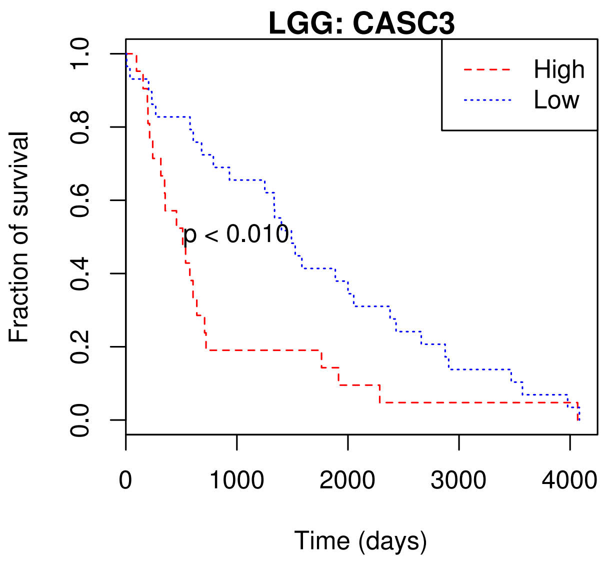
LGG: CASC3
High
Low
p < 0.010
Fraction of survival
0.0
0.2
0.4
0.6
0.8
1.0
0
1000
2000
3000
4000
Time (days)

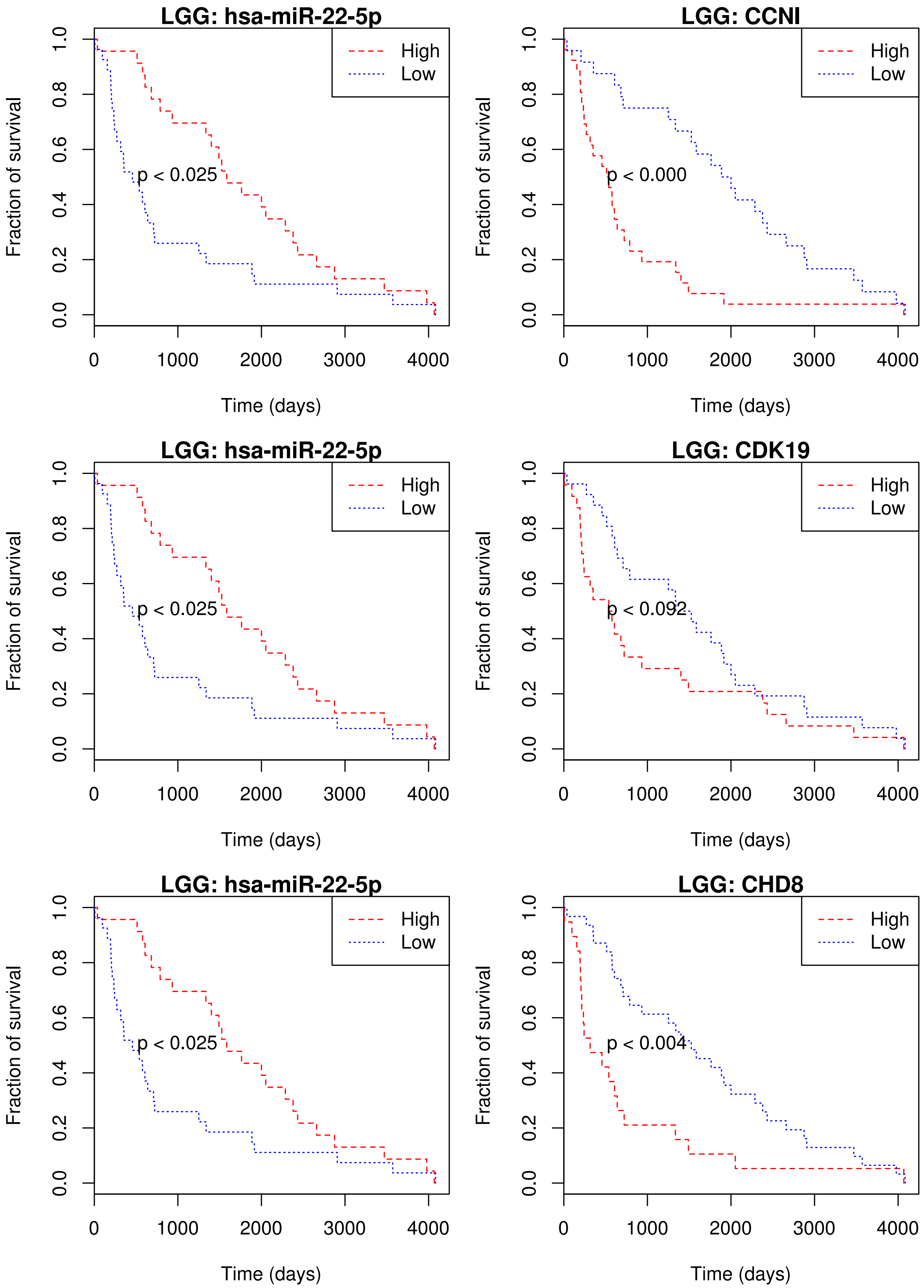
LGG: hsa-miR-22-5p
High
Low
p < 0.025
Fraction of survival
Time (days)
LGG: CCNI
High
Low
p < 0.000
Fraction of survival
Time (days)
LGG: hsa-miR-22-5p
High
Low
p < 0.025
Fraction of survival
Time (days)
LGG: CDK19
High
Low
p < 0.092
Fraction of survival
Time (days)
LGG: hsa-miR-22-5p
High
Low
p < 0.025
Fraction of survival
Time (days)
LGG: CHD8
High
Low
p < 0.004
Fraction of survival
Time (days)
0.0
0.2
0.4
0.6
0.8
1.0
0
1000
2000
3000
4000

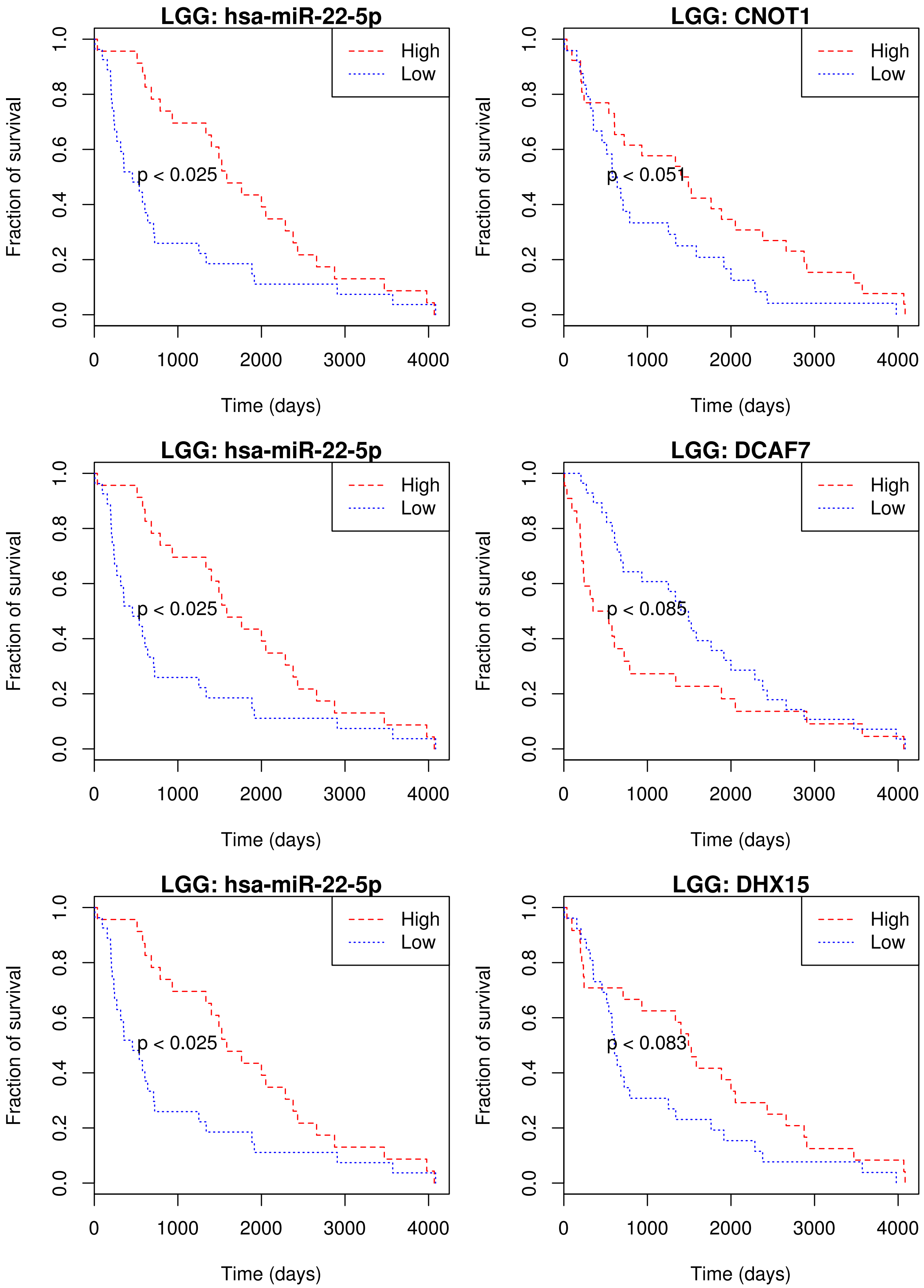

LGG: hsa-miR-22-5p
High
Low
p < 0.025
Fraction of survival
Time (days)
LGG: CNOT1
High
Low
p < 0.051
Fraction of survival
Time (days)
LGG: hsa-miR-22-5p
High
Low
p < 0.025
Fraction of survival
Time (days)
LGG: DCAF7
High
Low
p < 0.085
Fraction of survival
Time (days)
LGG: hsa-miR-22-5p
High
Low
p < 0.025
Fraction of survival
Time (days)
LGG: DHX15
High
Low
p < 0.083
Fraction of survival
Time (days)

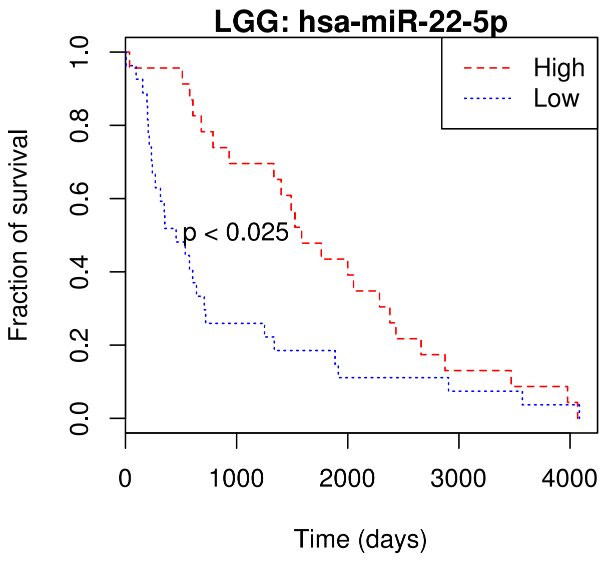
LGG: hsa-miR-22-5p
High
Low
p < 0.025
Fraction of survival
Time (days)

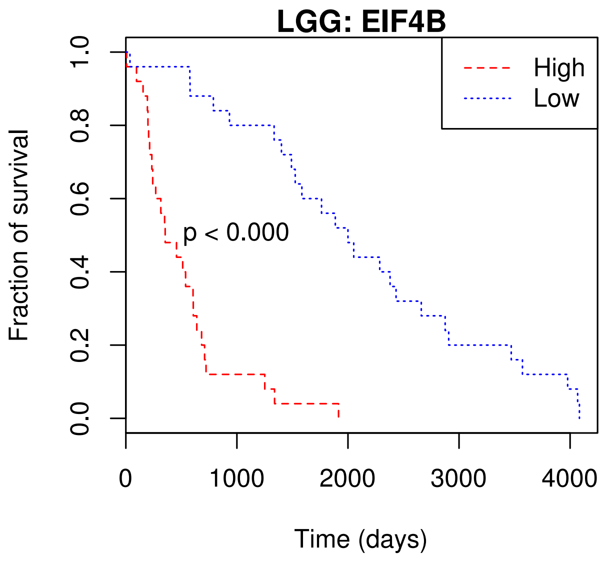
LGG: EIF4B
High
Low
p < 0.000
Fraction of survival
Time (days)

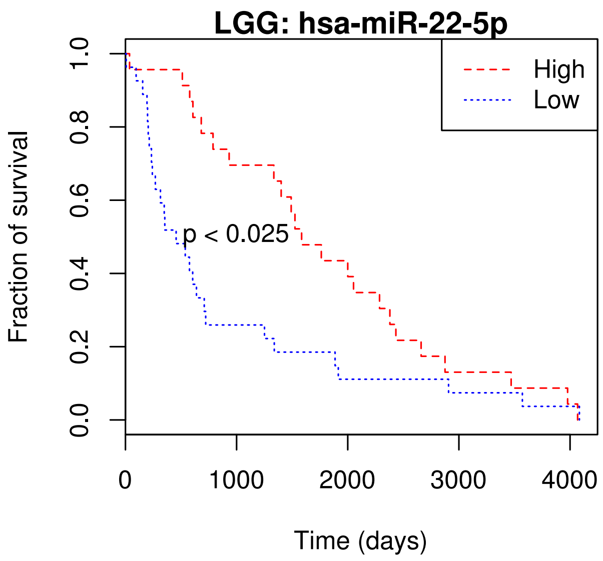
LGG: hsa-miR-22-5p
High
Low
p < 0.025
Fraction of survival
Time (days)

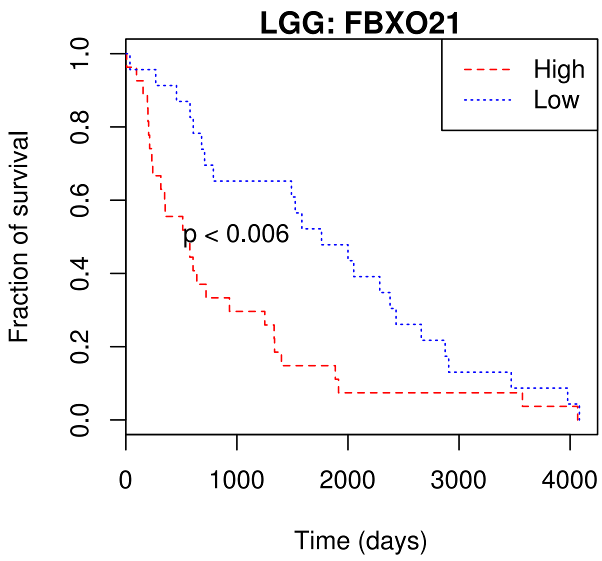
LGG: FBXO21
High
Low
p < 0.006
Fraction of survival
Time (days)

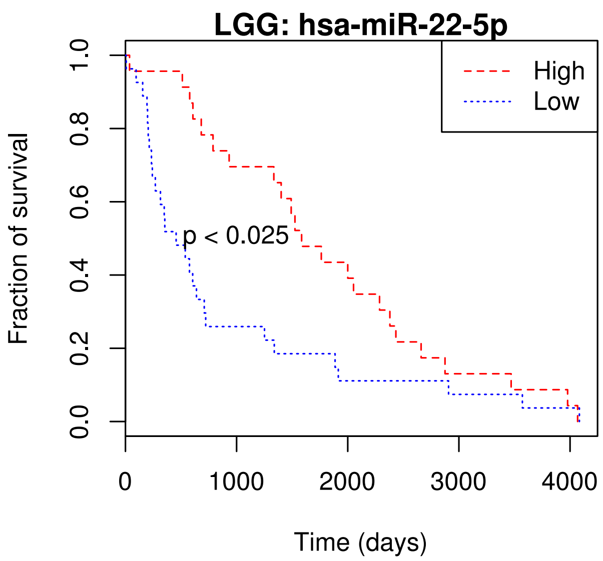
LGG: hsa-miR-22-5p
High
Low
p < 0.025
Fraction of survival
Time (days)

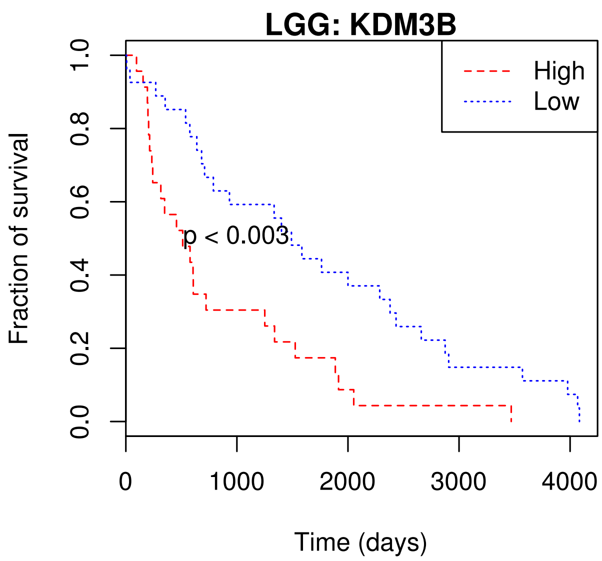
LGG: KDM3B
High
Low
p < 0.003
Fraction of survival
Time (days)

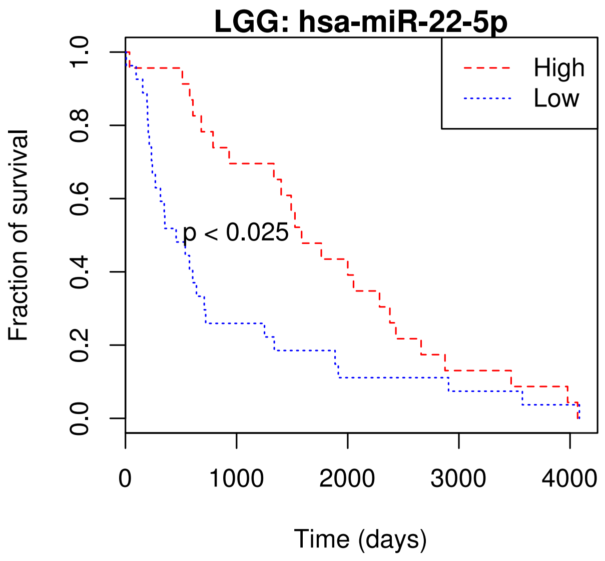
LGG: hsa-miR-22-5p
High
Low
p < 0.025
Fraction of survival
0.0 0.2 0.4 0.6 0.8 1.0
0 1000 2000 3000 4000
Time (days)

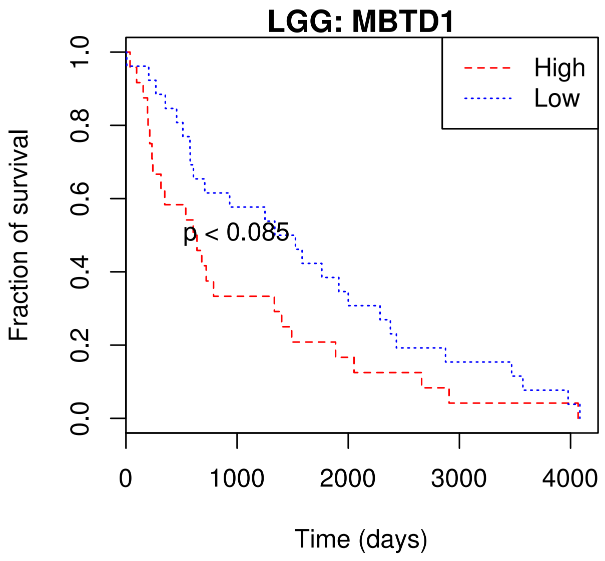
LGG: MBTD1
High
Low
p < 0.085
Fraction of survival
0.0 0.2 0.4 0.6 0.8 1.0
0 1000 2000 3000 4000
Time (days)

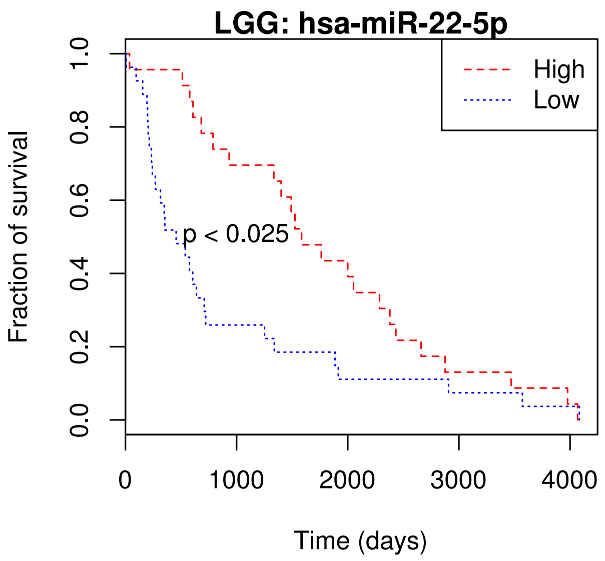
LGG: hsa-miR-22-5p
High
Low
p < 0.025
Fraction of survival
0.0 0.2 0.4 0.6 0.8 1.0
0 1000 2000 3000 4000
Time (days)

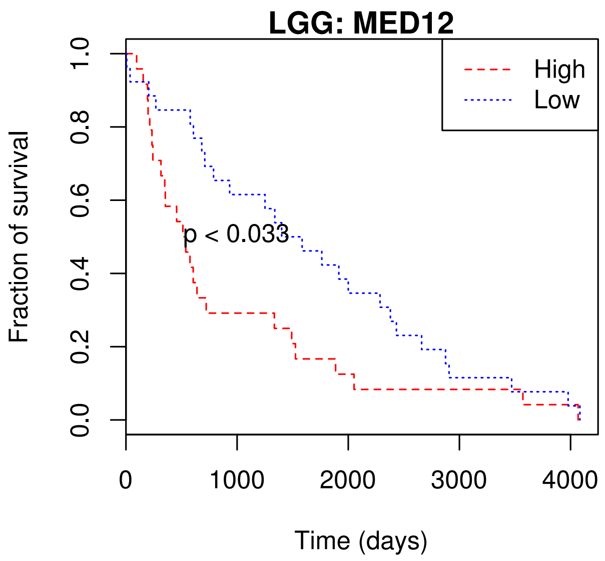
LGG: MED12
High
Low
p < 0.033
Fraction of survival
0.0 0.2 0.4 0.6 0.8 1.0
0 1000 2000 3000 4000
Time (days)

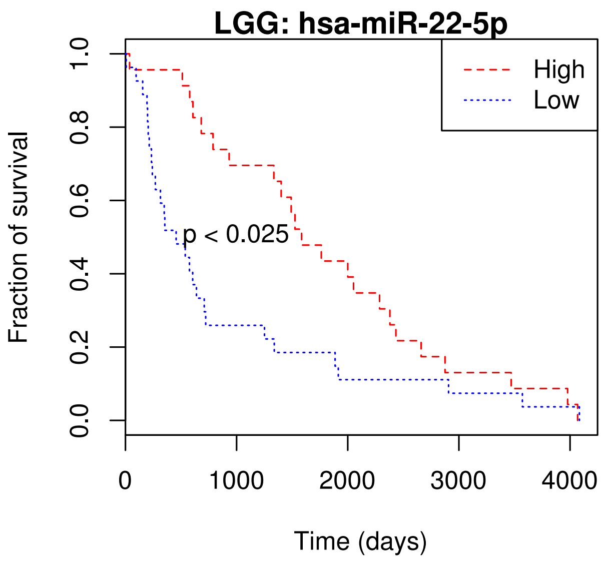
LGG: hsa-miR-22-5p
High
Low
p < 0.025
Fraction of survival
0.0 0.2 0.4 0.6 0.8 1.0
0 1000 2000 3000 4000
Time (days)

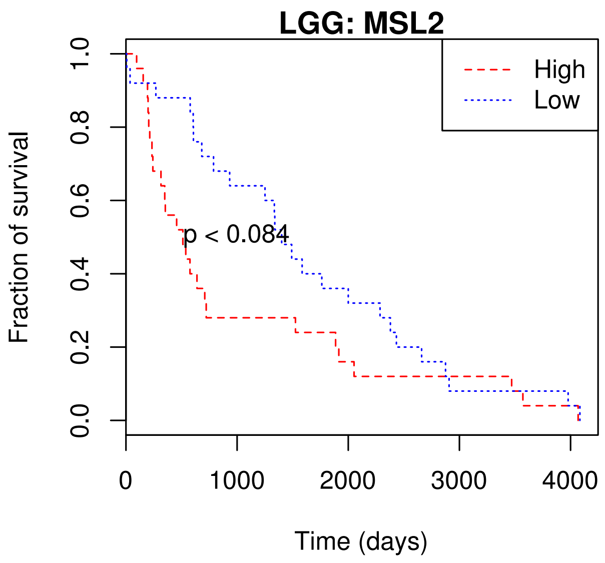
LGG: MSL2
High
Low
p < 0.084
Fraction of survival
0.0 0.2 0.4 0.6 0.8 1.0
0 1000 2000 3000 4000
Time (days)

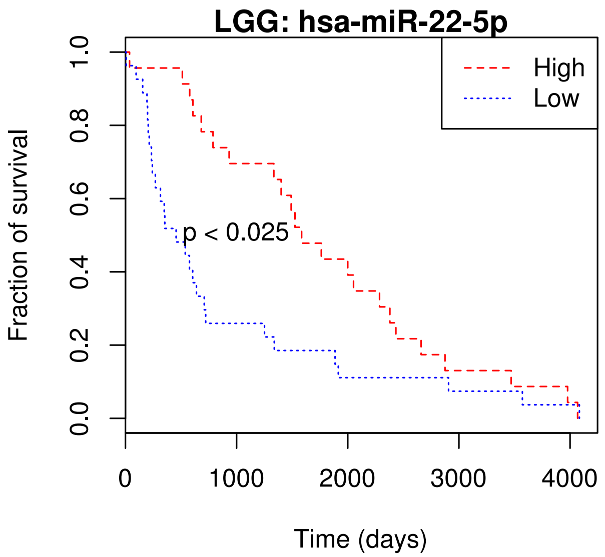
LGG: hsa-miR-22-5p
High
Low
p < 0.025
Fraction of survival
Time (days)
0.0 0.2 0.4 0.6 0.8 1.0
0 1000 2000 3000 4000

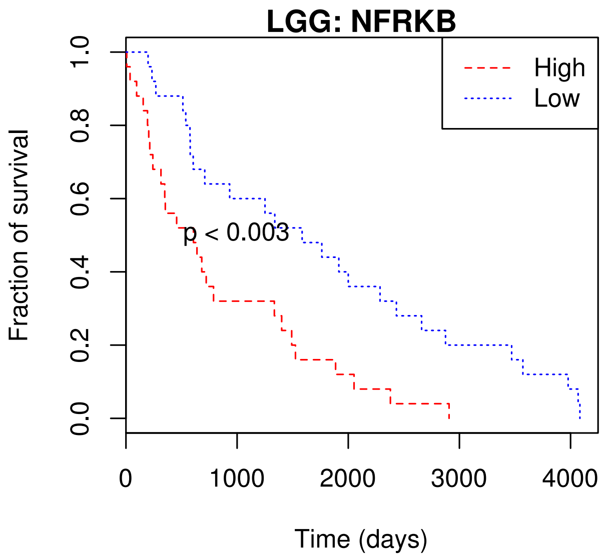
LGG: NFRKB
High
Low
p < 0.003
Fraction of survival
Time (days)
0.0 0.2 0.4 0.6 0.8 1.0
0 1000 2000 3000 4000

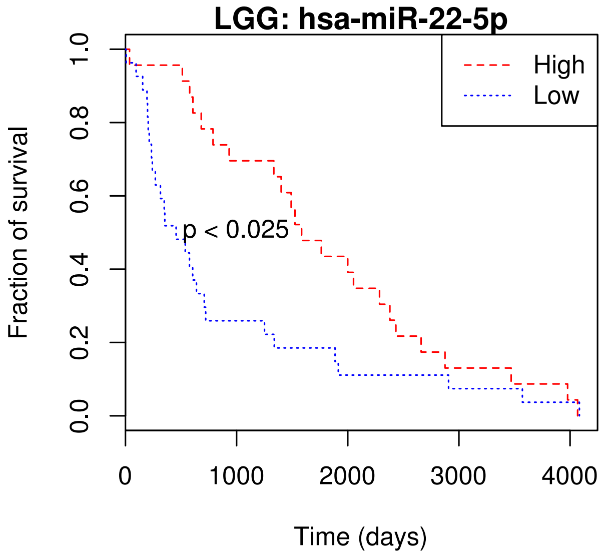
LGG: hsa-miR-22-5p
High
Low
p < 0.025
Fraction of survival
Time (days)
0.0 0.2 0.4 0.6 0.8 1.0
0 1000 2000 3000 4000

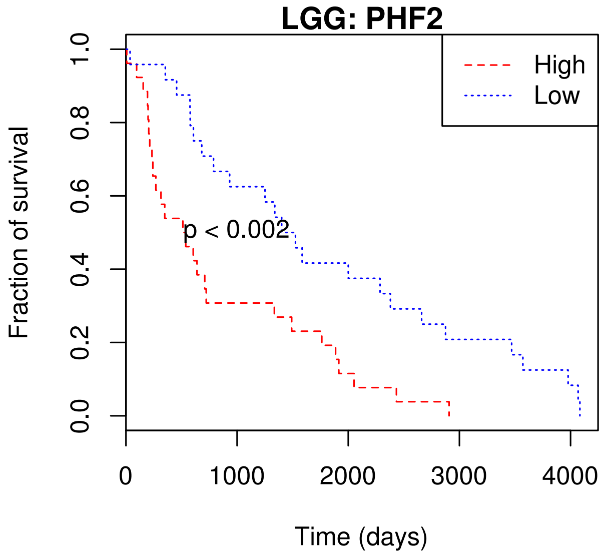
LGG: PHF2
High
Low
p < 0.002
Fraction of survival
Time (days)
0.0 0.2 0.4 0.6 0.8 1.0
0 1000 2000 3000 4000

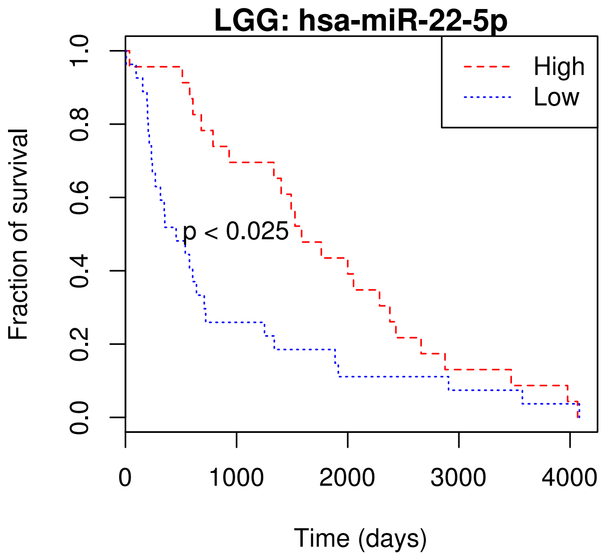
LGG: hsa-miR-22-5p
High
Low
p < 0.025
Fraction of survival
Time (days)
0.0 0.2 0.4 0.6 0.8 1.0
0 1000 2000 3000 4000

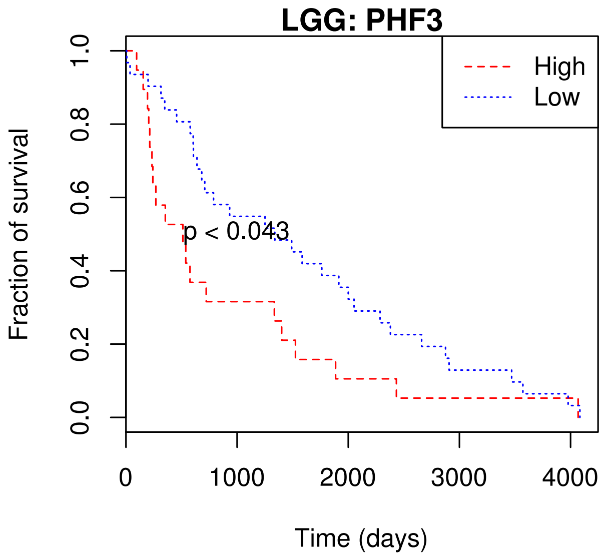
LGG: PHF3
High
Low
p < 0.043
Fraction of survival
Time (days)
0.0 0.2 0.4 0.6 0.8 1.0
0 1000 2000 3000 4000

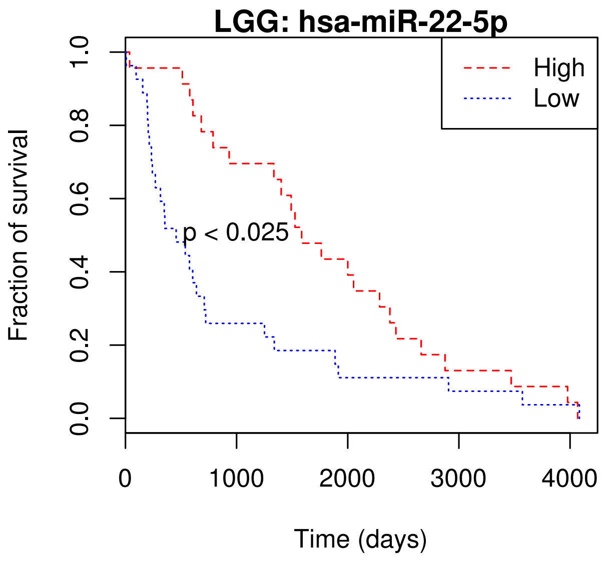
LGG: hsa-miR-22-5p
High
Low
p < 0.025
Fraction of survival
Time (days)
0.0 0.2 0.4 0.6 0.8 1.0
0 1000 2000 3000 4000

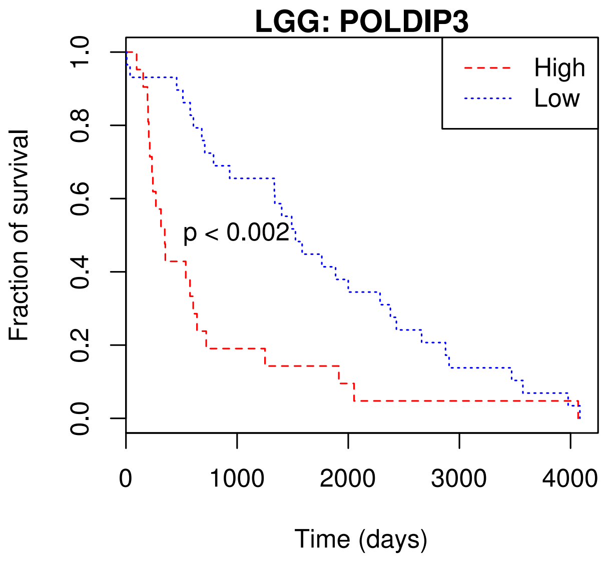
LGG: POLDIP3
High
Low
p < 0.002
Fraction of survival
Time (days)
0.0 0.2 0.4 0.6 0.8 1.0
0 1000 2000 3000 4000

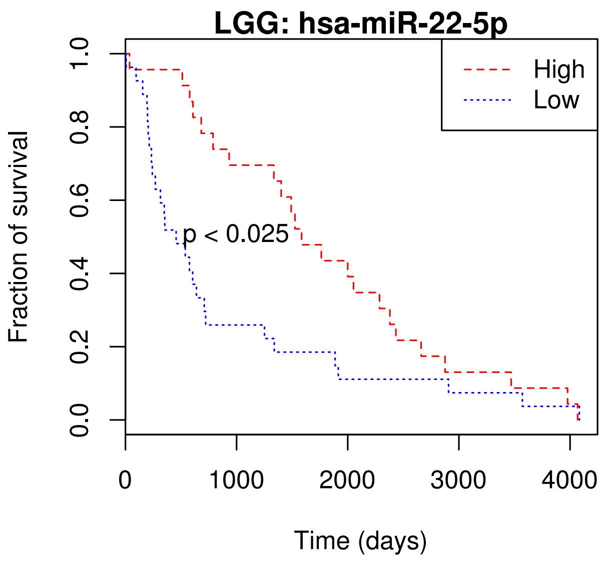
LGG: hsa-miR-22-5p
High
Low
p < 0.025
Fraction of survival
Time (days)
0.0 0.2 0.4 0.6 0.8 1.0
0 1000 2000 3000 4000

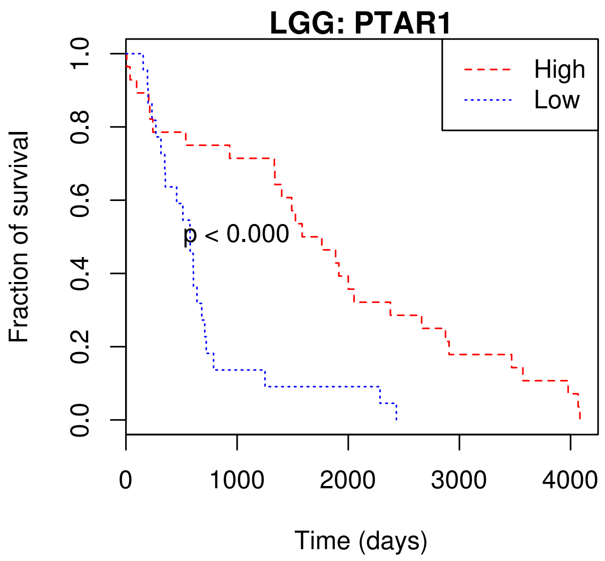
LGG: PTAR1
High
Low
p < 0.000
Fraction of survival
Time (days)
0.0 0.2 0.4 0.6 0.8 1.0
0 1000 2000 3000 4000

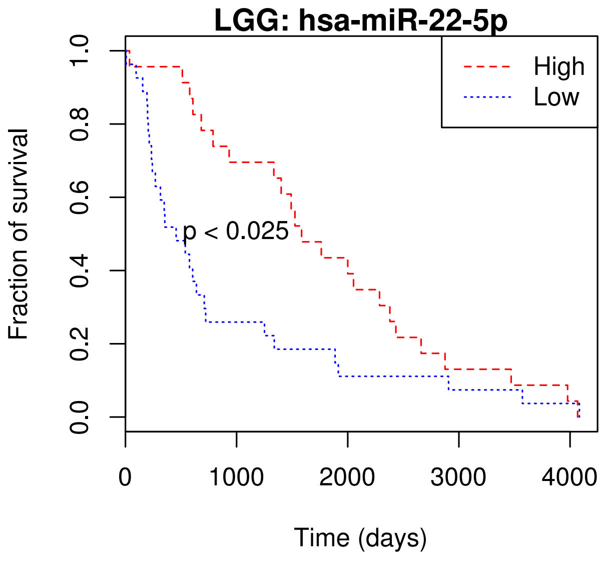
LGG: hsa-miR-22-5p
High
Low
p < 0.025
Fraction of survival
Time (days)
0.0 0.2 0.4 0.6 0.8 1.0
0 1000 2000 3000 4000

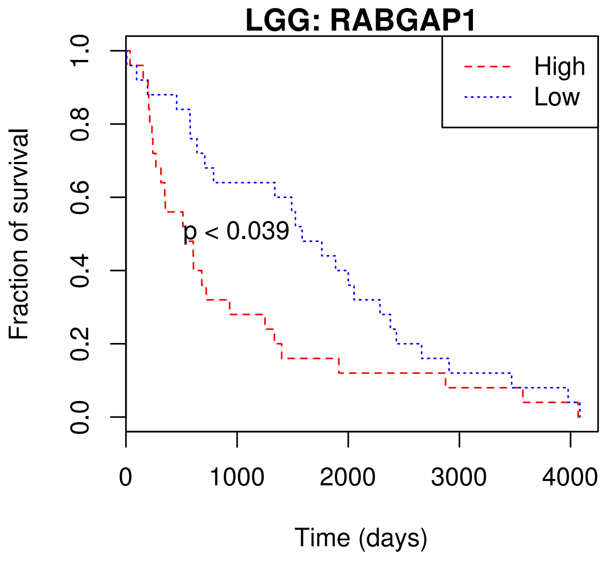
LGG: RABGAP1
High
Low
p < 0.039
Fraction of survival
Time (days)
0.0 0.2 0.4 0.6 0.8 1.0
0 1000 2000 3000 4000

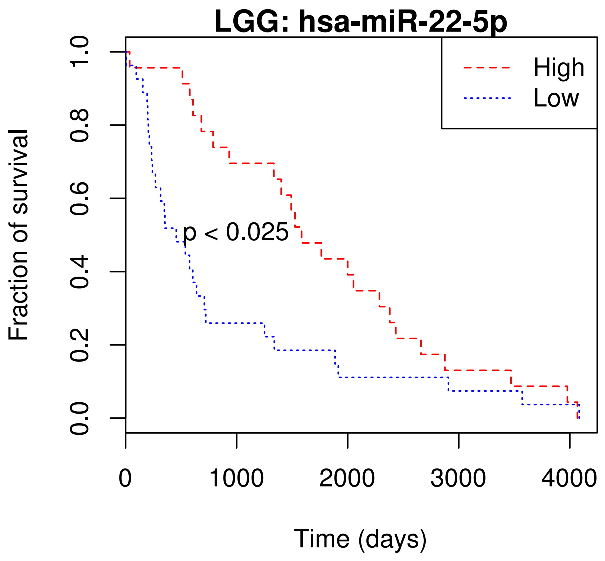
LGG: hsa-miR-22-5p
High
Low
p < 0.025
Fraction of survival
Time (days)
0.0
0.2
0.4
0.6
0.8
1.0
0
1000
2000
3000
4000

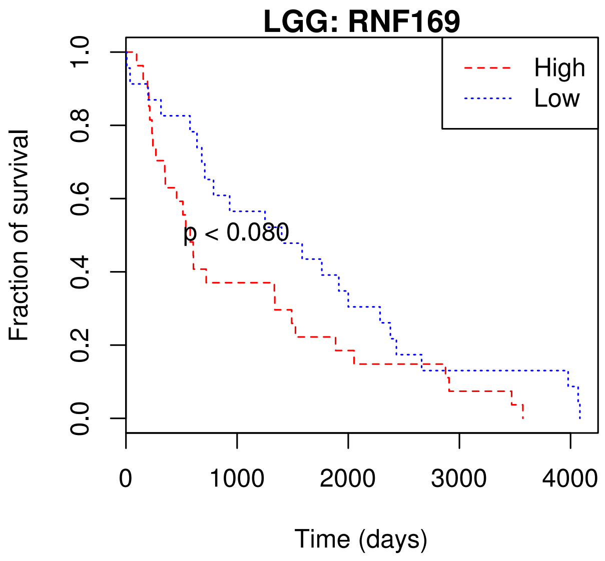
LGG: RNF169
High
Low
p < 0.080
Fraction of survival
Time (days)
0.0
0.2
0.4
0.6
0.8
1.0
0
1000
2000
3000
4000

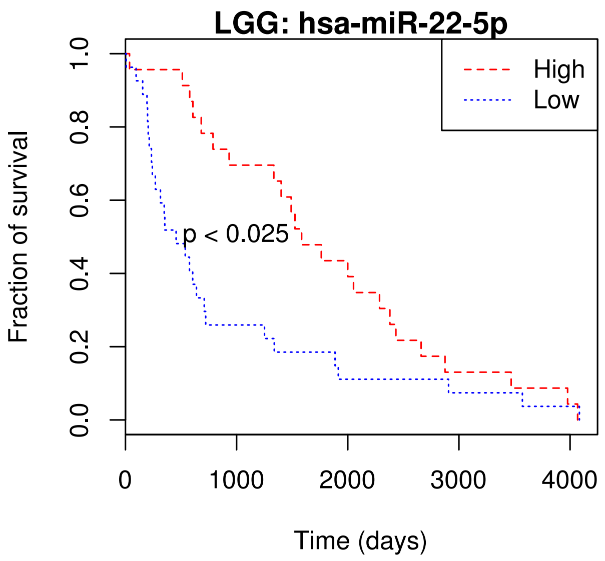
LGG: hsa-miR-22-5p
High
Low
p < 0.025
Fraction of survival
Time (days)
0.0
0.2
0.4
0.6
0.8
1.0
0
1000
2000
3000
4000

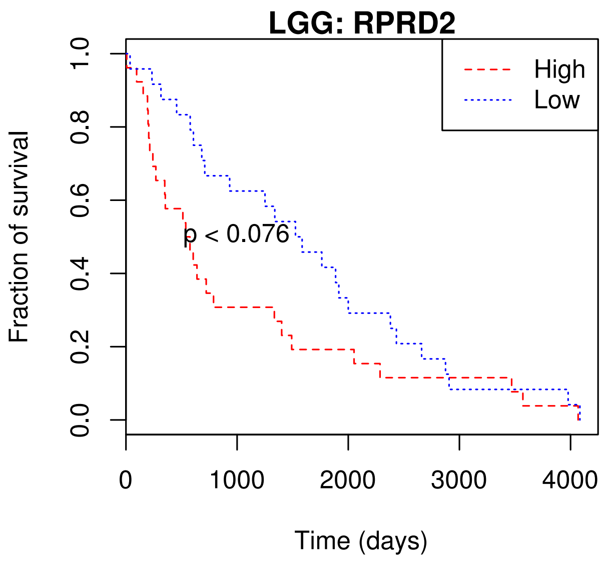
LGG: RPRD2
High
Low
p < 0.076
Fraction of survival
Time (days)
0.0
0.2
0.4
0.6
0.8
1.0
0
1000
2000
3000
4000

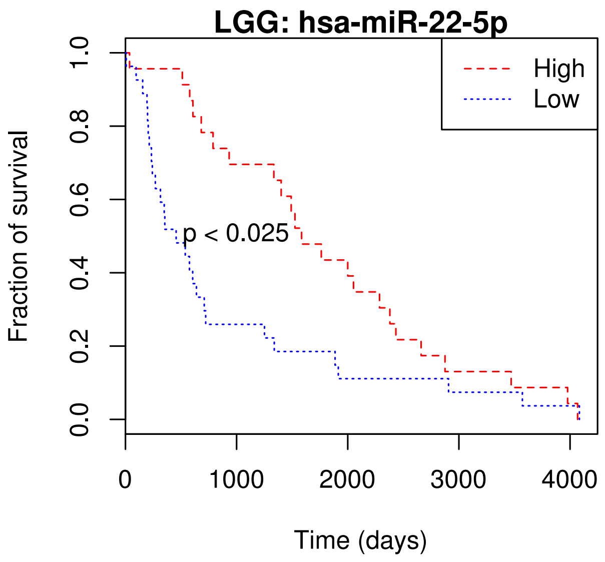
LGG: hsa-miR-22-5p
High
Low
p < 0.025
Fraction of survival
Time (days)
0.0
0.2
0.4
0.6
0.8
1.0
0
1000
2000
3000
4000

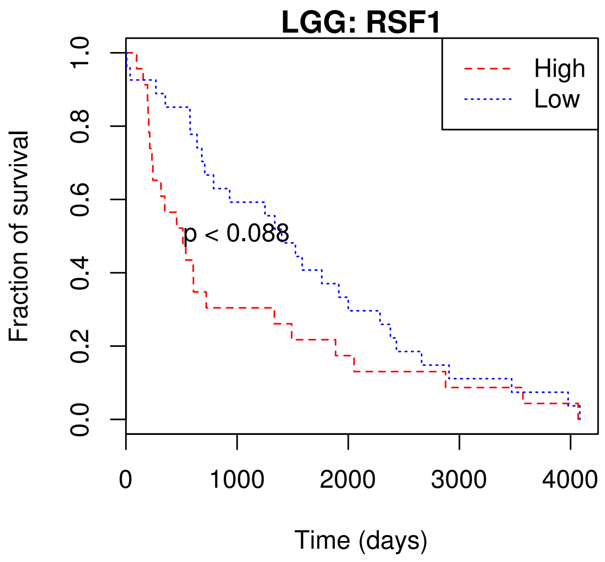
LGG: RSF1
High
Low
p < 0.088
Fraction of survival
Time (days)
0.0
0.2
0.4
0.6
0.8
1.0
0
1000
2000
3000
4000

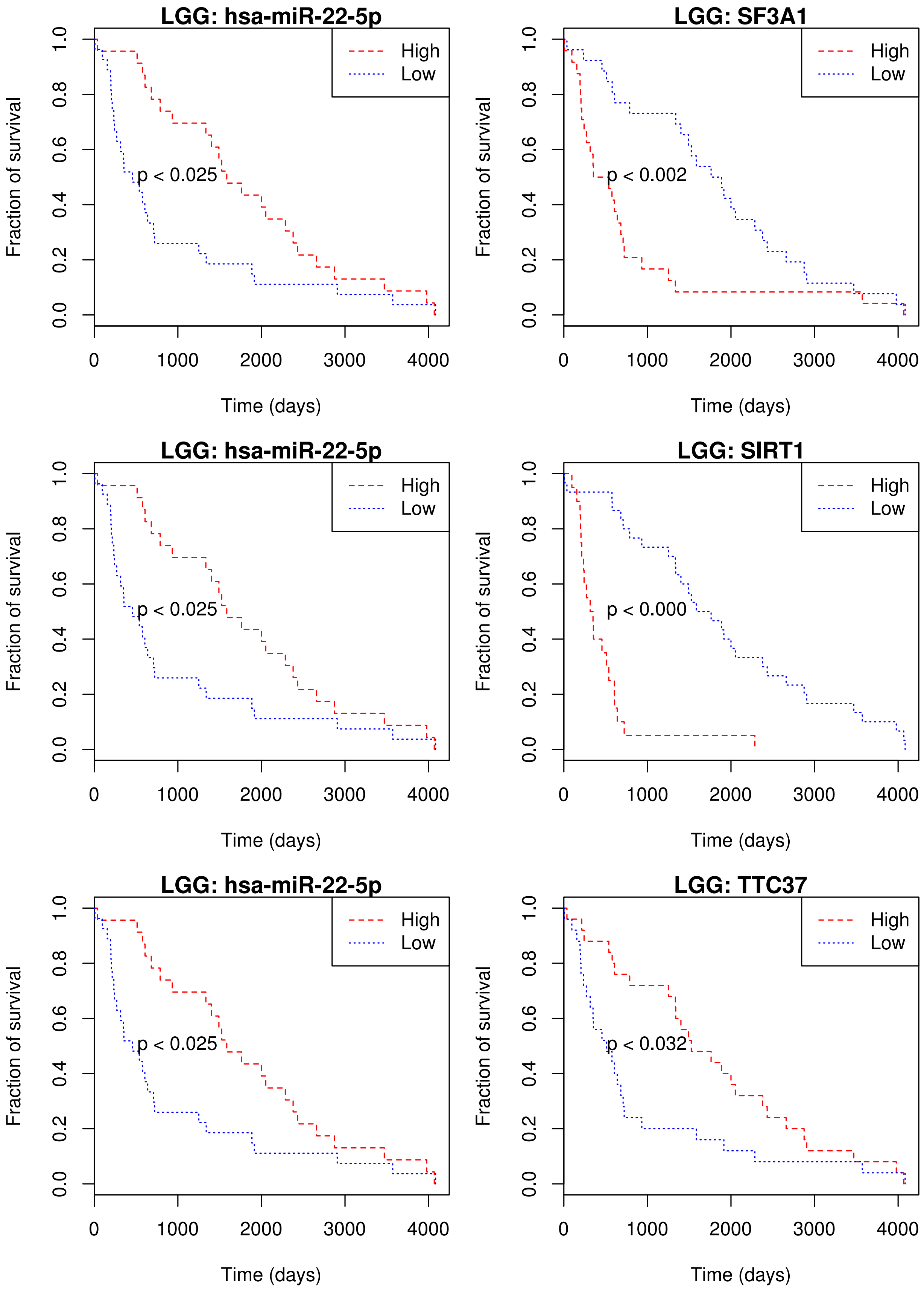
LGG: hsa-miR-22-5p
High
Low
p < 0.025
Fraction of survival
Time (days)
LGG: SF3A1
High
Low
p < 0.002
Fraction of survival
Time (days)
LGG: hsa-miR-22-5p
High
Low
p < 0.025
Fraction of survival
Time (days)
LGG: SIRT1
High
Low
p < 0.000
Fraction of survival
Time (days)
LGG: hsa-miR-22-5p
High
Low
p < 0.025
Fraction of survival
Time (days)
LGG: TTC37
High
Low
p < 0.032
Fraction of survival
Time (days)

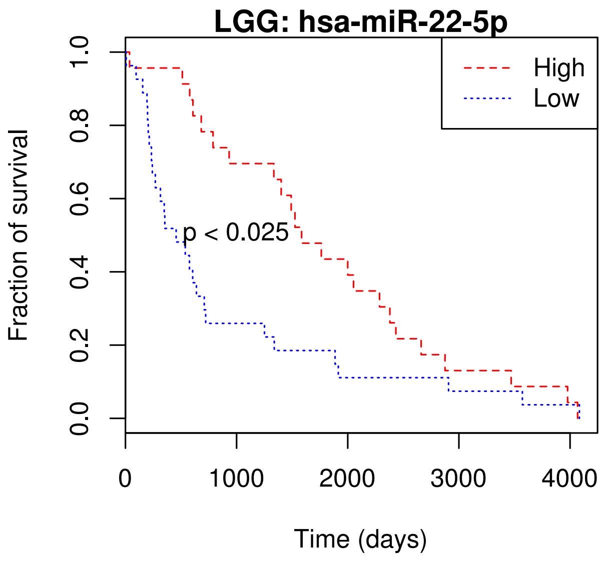
LGG: hsa-miR-22-5p
High
Low
p < 0.025
Fraction of survival
Time (days)
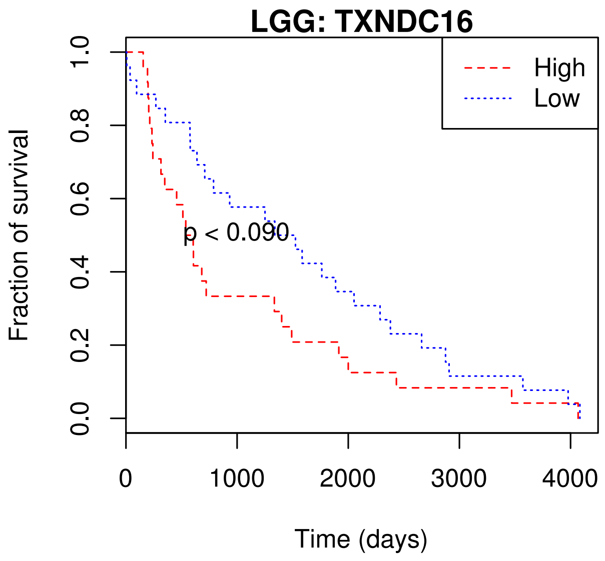
LGG: TXNDC16
High
Low
p < 0.090
Fraction of survival
Time (days)
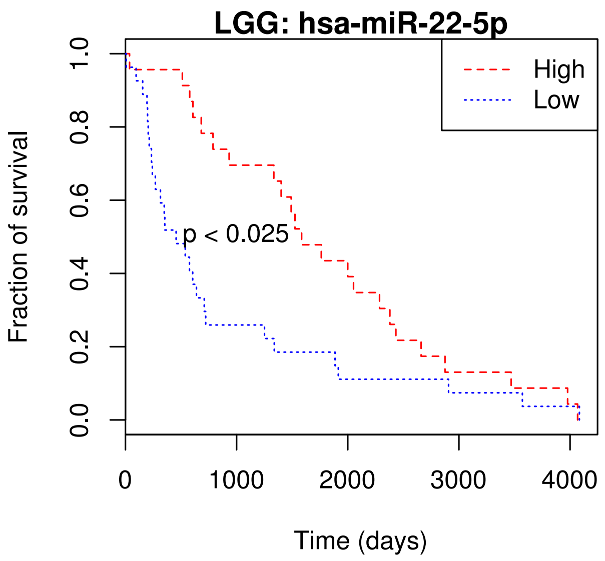
LGG: hsa-miR-22-5p
High
Low
p < 0.025
Fraction of survival
Time (days)
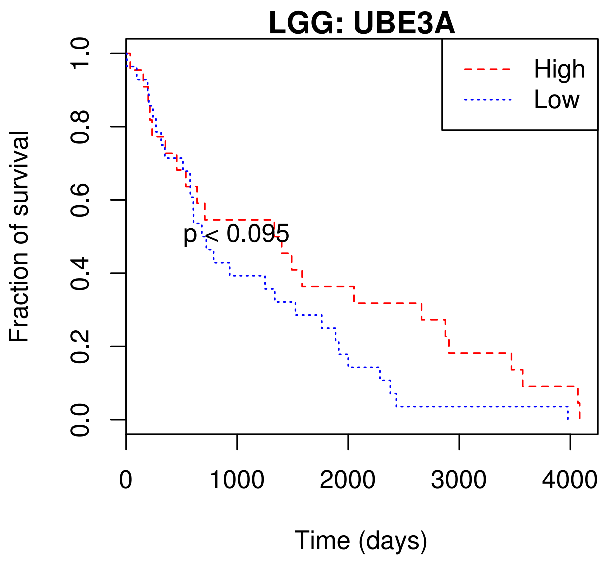
LGG: UBE3A
High
Low
p < 0.095
Fraction of survival
Time (days)
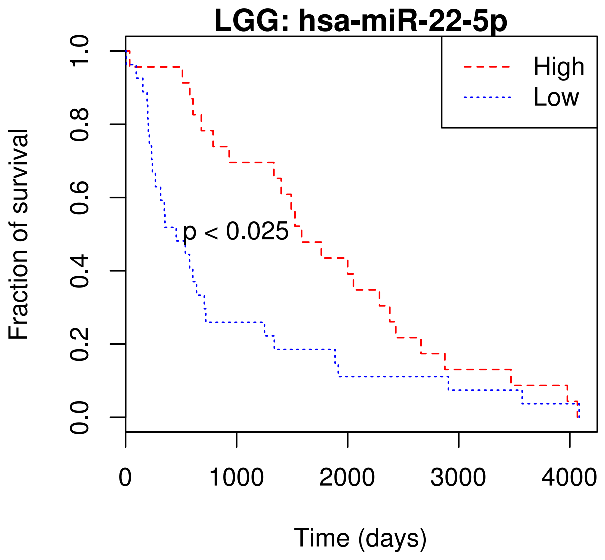
LGG: hsa-miR-22-5p
High
Low
p < 0.025
Fraction of survival
Time (days)
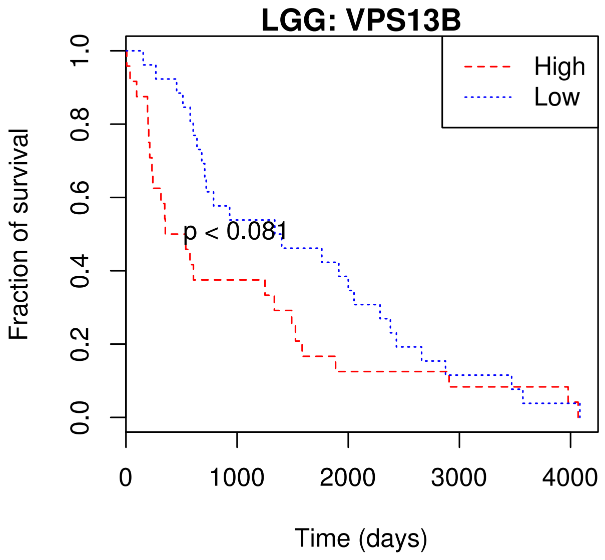
LGG: VPS13B
High
Low
p < 0.081
Fraction of survival
Time (days)

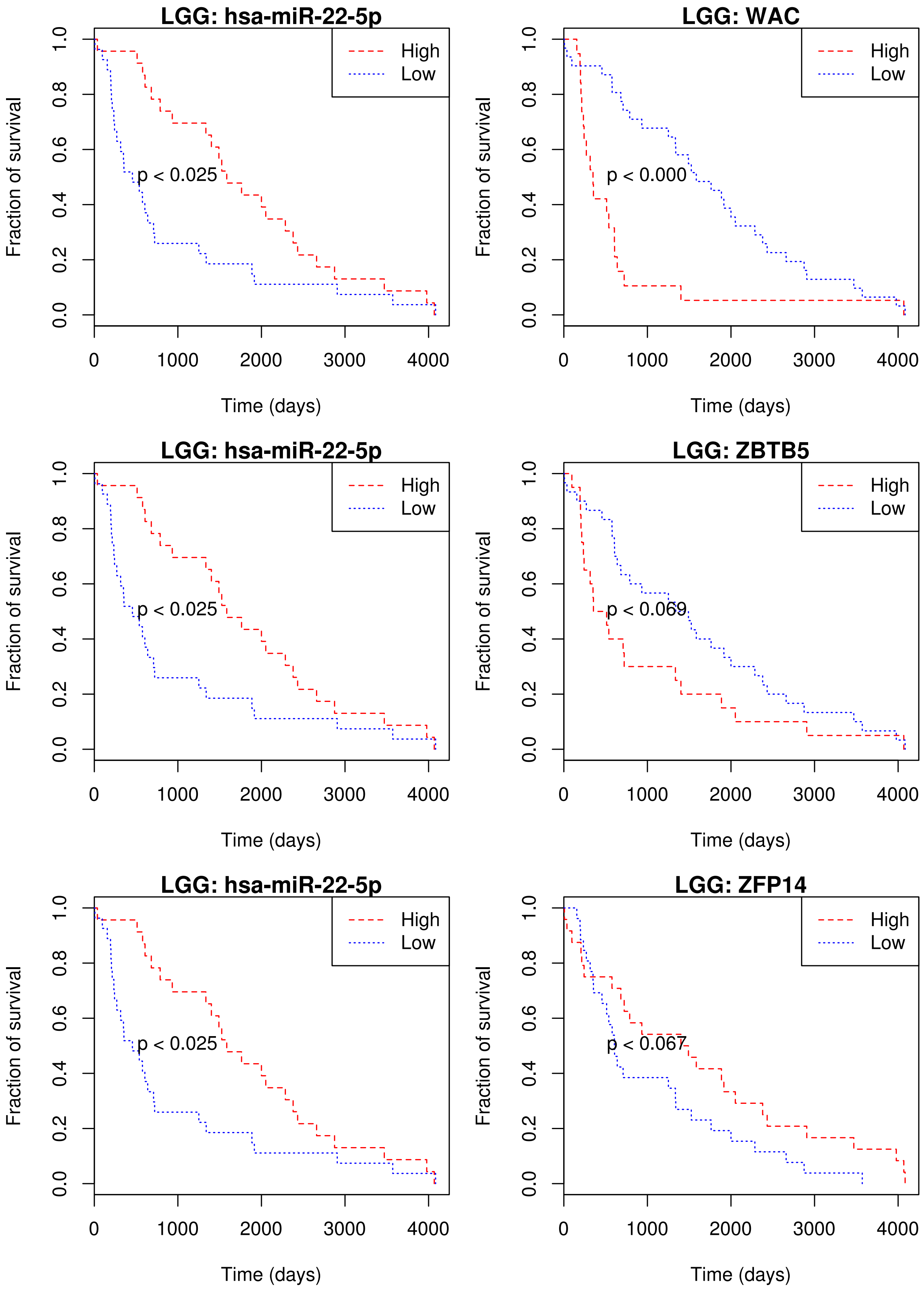
LGG: hsa-miR-22-5p
High
Low
p < 0.025
Fraction of survival
Time (days)
LGG: WAC
High
Low
p < 0.000
Fraction of survival
Time (days)
LGG: hsa-miR-22-5p
High
Low
p < 0.025
Fraction of survival
Time (days)
LGG: ZBTB5
High
Low
p < 0.069
Fraction of survival
Time (days)
LGG: hsa-miR-22-5p
High
Low
p < 0.025
Fraction of survival
Time (days)
LGG: ZFP14
High
Low
p < 0.067
Fraction of survival
Time (days)

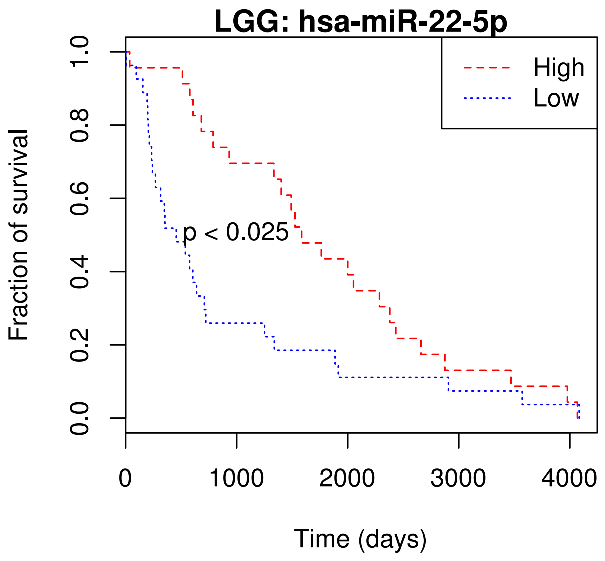
LGG: hsa-miR-22-5p
High
Low
p < 0.025
Fraction of survival
0.0
0.2
0.4
0.6
0.8
1.0
0
1000
2000
3000
4000
Time (days)

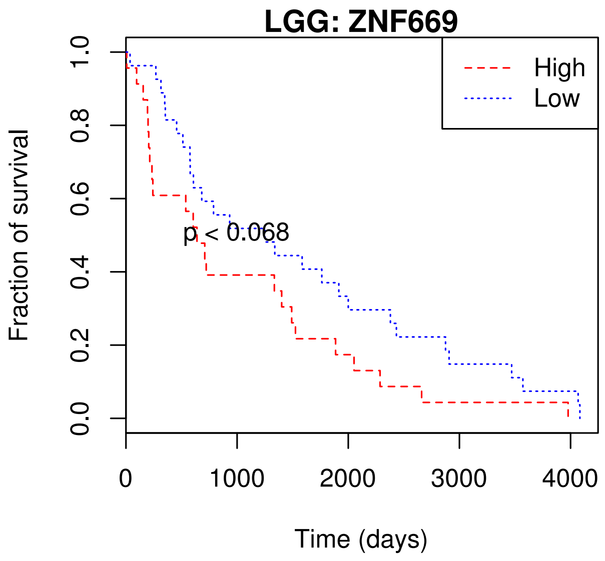
LGG: ZNF669
High
Low
p < 0.068
Fraction of survival
0.0
0.2
0.4
0.6
0.8
1.0
0
1000
2000
3000
4000
Time (days)

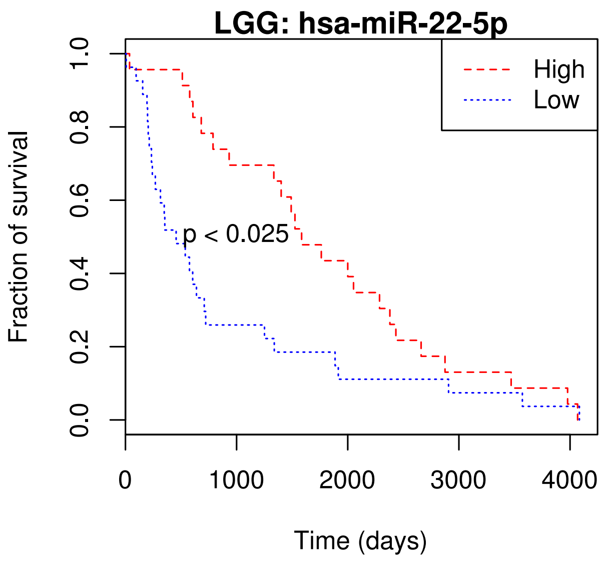
LGG: hsa-miR-22-5p
High
Low
p < 0.025
Fraction of survival
0.0
0.2
0.4
0.6
0.8
1.0
0
1000
2000
3000
4000
Time (days)

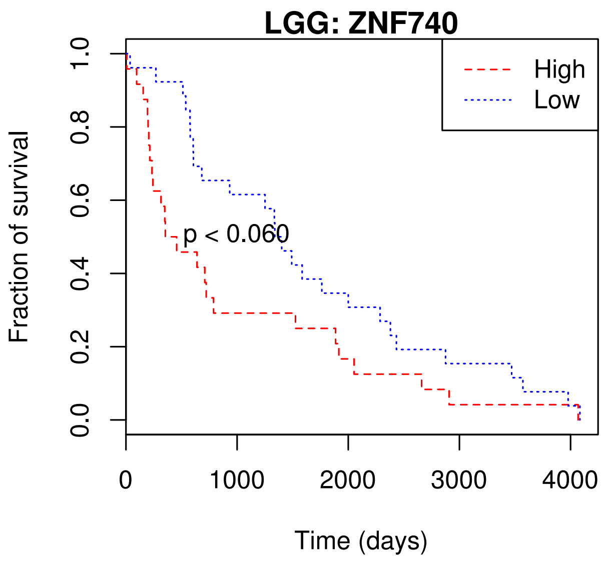
LGG: ZNF740
High
Low
p < 0.060
Fraction of survival
0.0
0.2
0.4
0.6
0.8
1.0
0
1000
2000
3000
4000
Time (days)

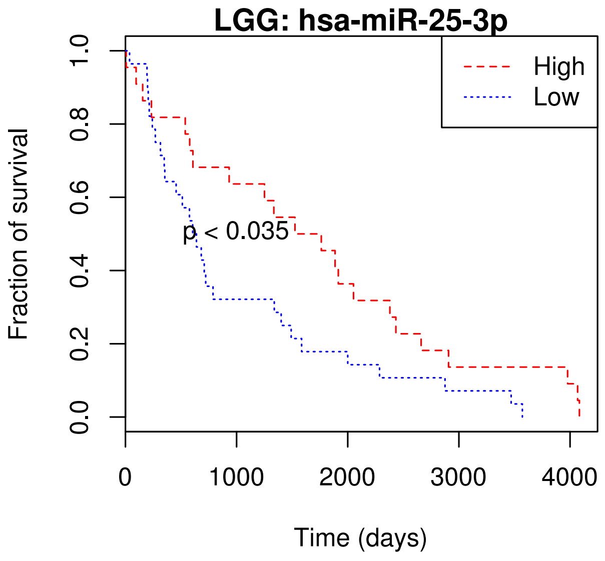
LGG: hsa-miR-25-3p
High
Low
p < 0.035
Fraction of survival
0.0
0.2
0.4
0.6
0.8
1.0
0
1000
2000
3000
4000
Time (days)

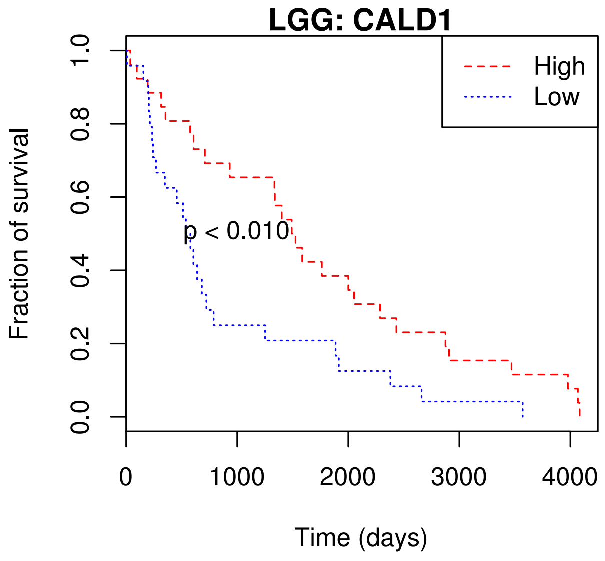
LGG: CALD1
High
Low
p < 0.010
Fraction of survival
0.0
0.2
0.4
0.6
0.8
1.0
0
1000
2000
3000
4000
Time (days)

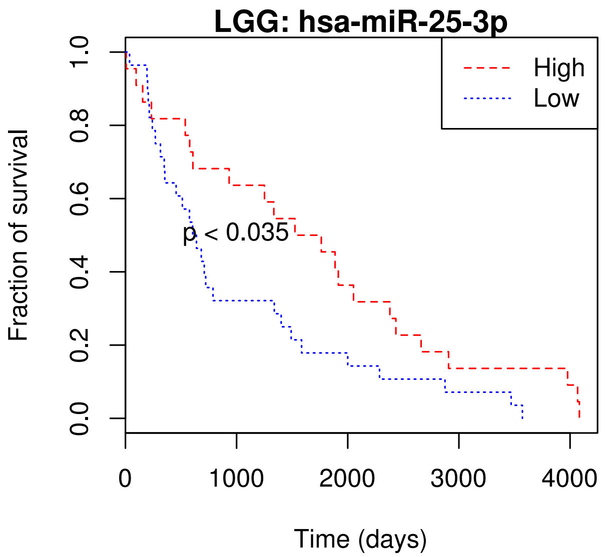
LGG: hsa-miR-25-3p
High
Low
p < 0.035
Fraction of survival
Time (days)

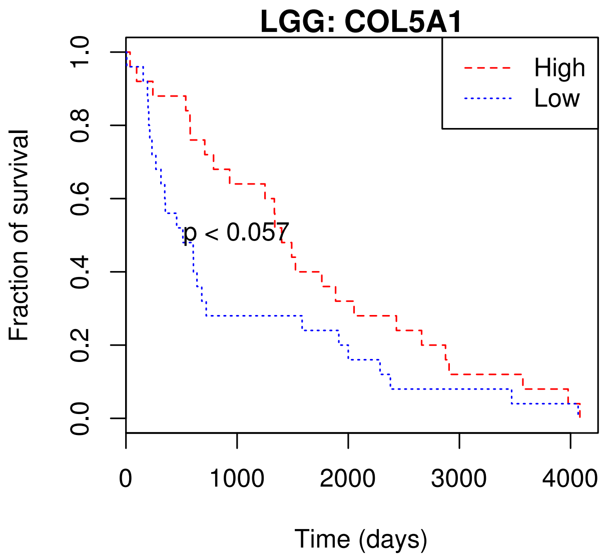
LGG: COL5A1
High
Low
p < 0.057
Fraction of survival
Time (days)

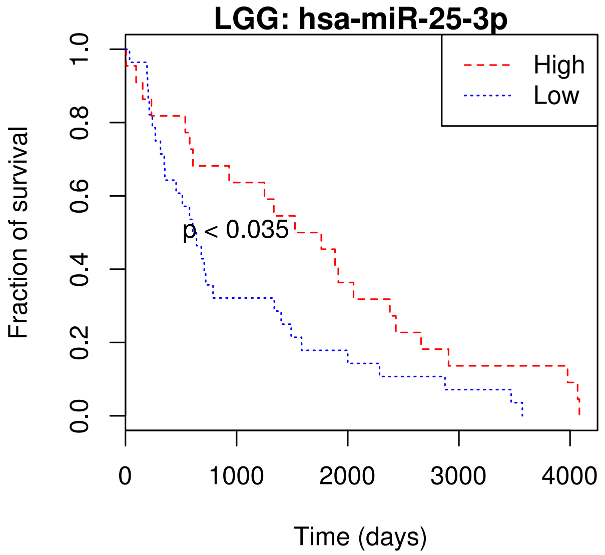
LGG: hsa-miR-25-3p
High
Low
p < 0.035
Fraction of survival
Time (days)

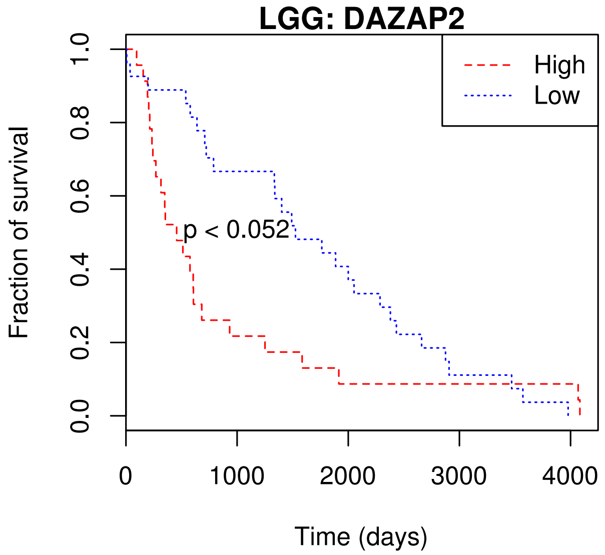
LGG: DAZAP2
High
Low
p < 0.052
Fraction of survival
Time (days)

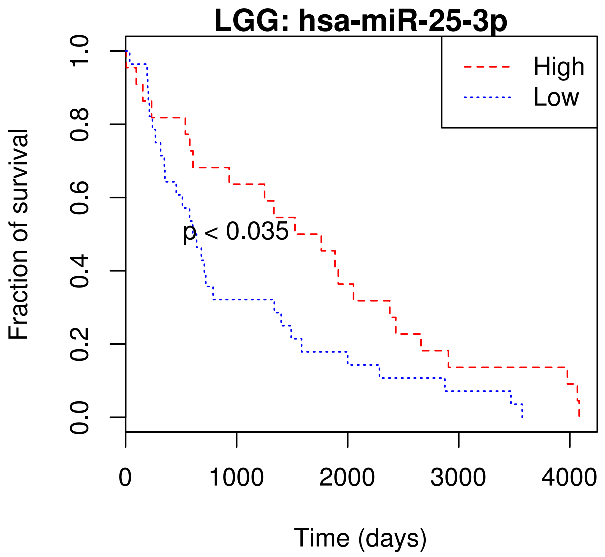
LGG: hsa-miR-25-3p
High
Low
p < 0.035
Fraction of survival
Time (days)

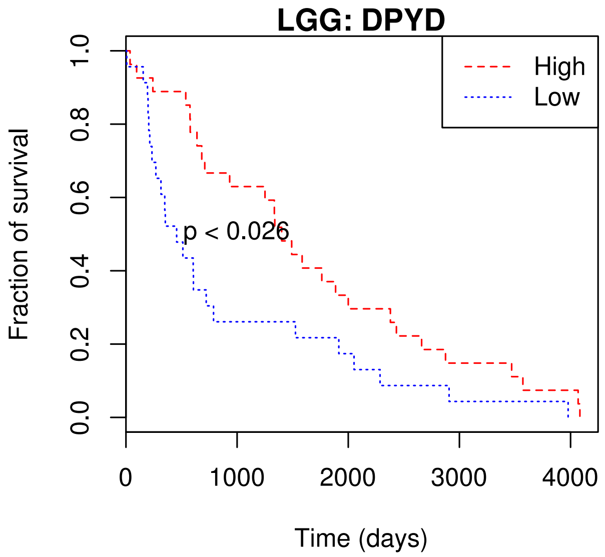
LGG: DPYD
High
Low
p < 0.026
Fraction of survival
Time (days)

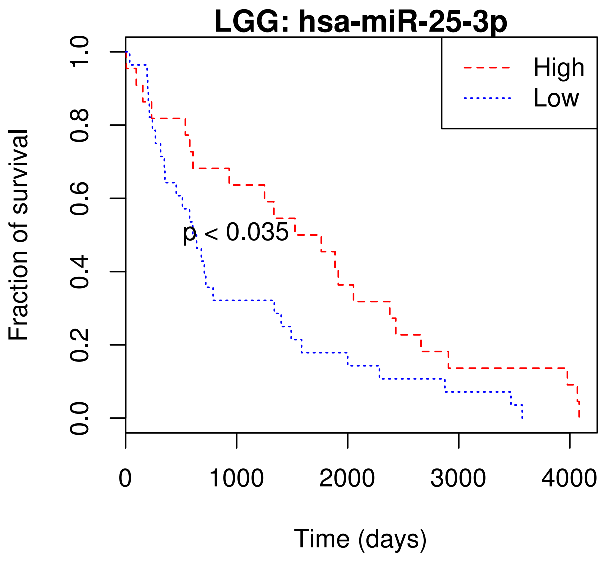
LGG: hsa-miR-25-3p
High
Low
p < 0.035
Fraction of survival
0.0
0.2
0.4
0.6
0.8
1.0
0
1000
2000
3000
4000
Time (days)

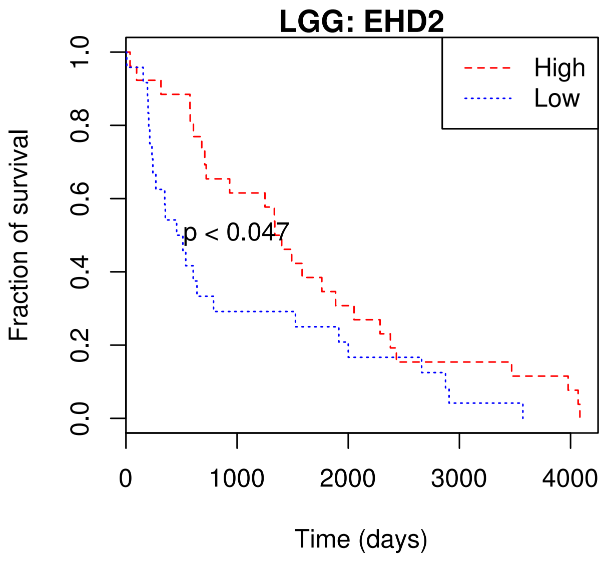
LGG: EHD2
High
Low
p < 0.047
Fraction of survival
0.0
0.2
0.4
0.6
0.8
1.0
0
1000
2000
3000
4000
Time (days)

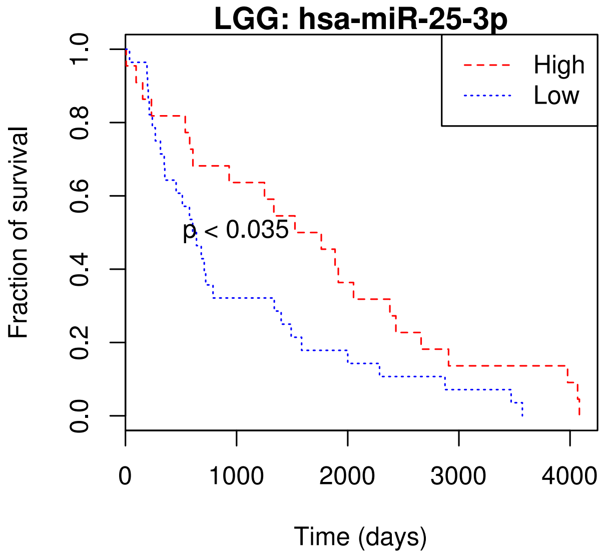
LGG: hsa-miR-25-3p
High
Low
p < 0.035
Fraction of survival
0.0
0.2
0.4
0.6
0.8
1.0
0
1000
2000
3000
4000
Time (days)

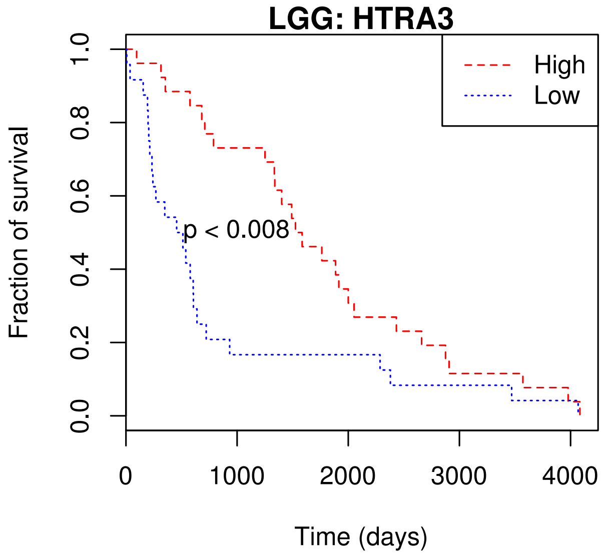
LGG: HTRA3
High
Low
p < 0.008
Fraction of survival
0.0
0.2
0.4
0.6
0.8
1.0
0
1000
2000
3000
4000
Time (days)

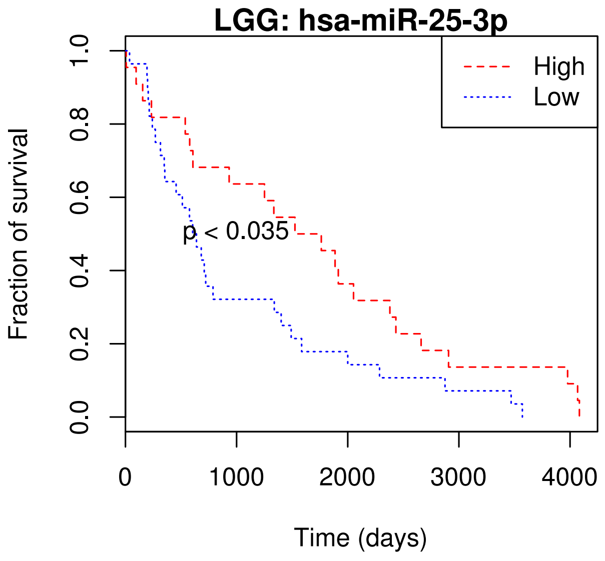
LGG: hsa-miR-25-3p
High
Low
p < 0.035
Fraction of survival
0.0
0.2
0.4
0.6
0.8
1.0
0
1000
2000
3000
4000
Time (days)

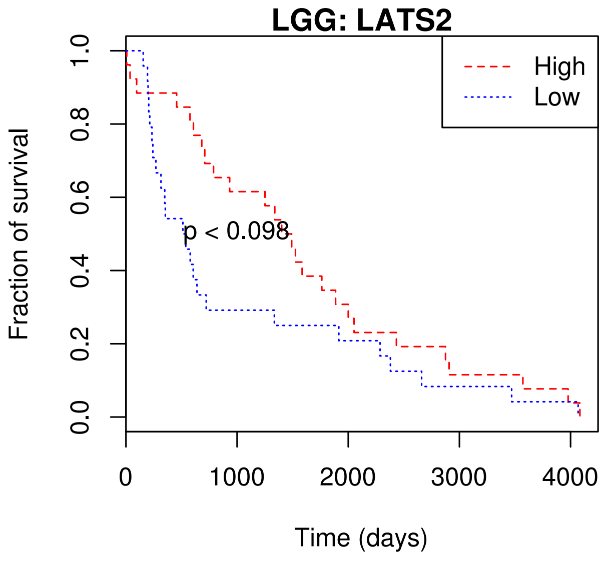
LGG: LATS2
High
Low
p < 0.098
Fraction of survival
0.0
0.2
0.4
0.6
0.8
1.0
0
1000
2000
3000
4000
Time (days)

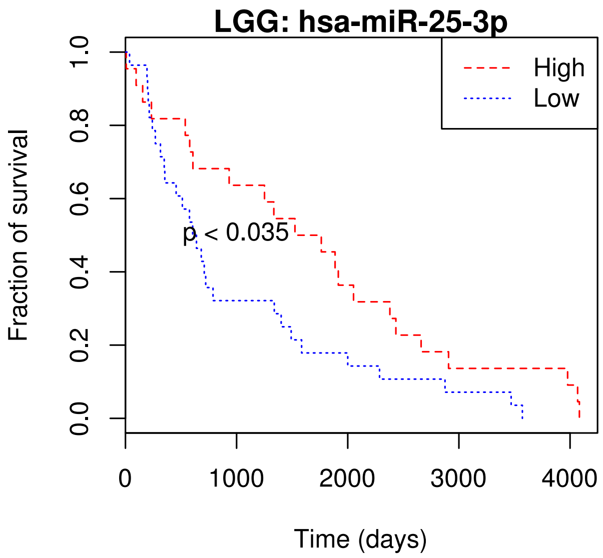
LGG: hsa-miR-25-3p
High
Low
p < 0.035
Fraction of survival
0.0
0.2
0.4
0.6
0.8
1.0
0
1000
2000
3000
4000
Time (days)

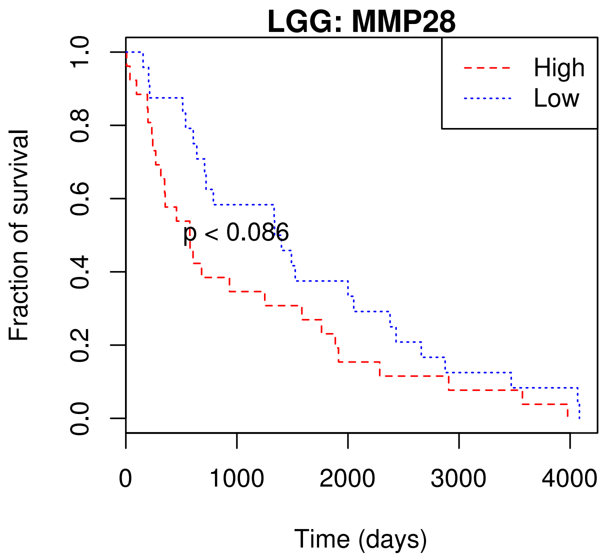
LGG: MMP28
High
Low
p < 0.086
Fraction of survival
0.0
0.2
0.4
0.6
0.8
1.0
0
1000
2000
3000
4000
Time (days)

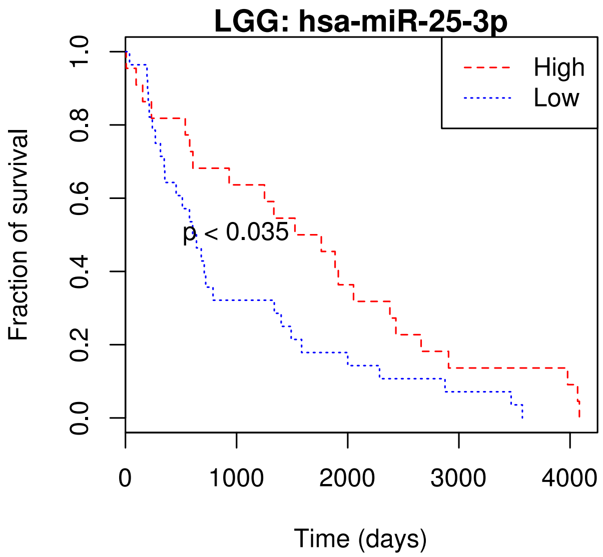
LGG: hsa-miR-25-3p
High
Low
p < 0.035
Fraction of survival
0.0
0.2
0.4
0.6
0.8
1.0
0
1000
2000
3000
4000
Time (days)

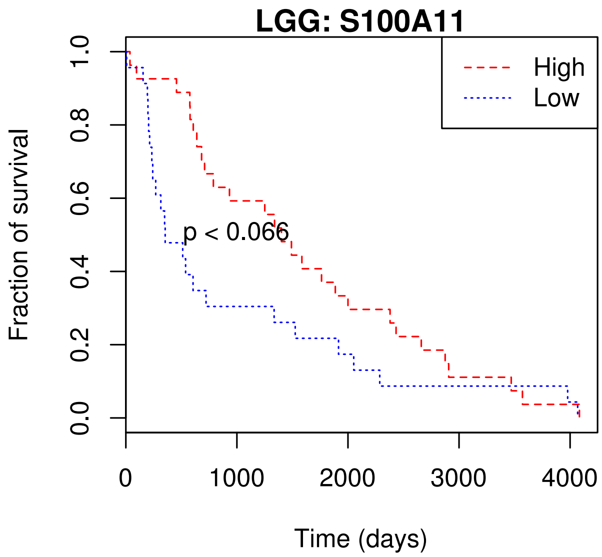
LGG: S100A11
High
Low
p < 0.066
Fraction of survival
0.0
0.2
0.4
0.6
0.8
1.0
0
1000
2000
3000
4000
Time (days)

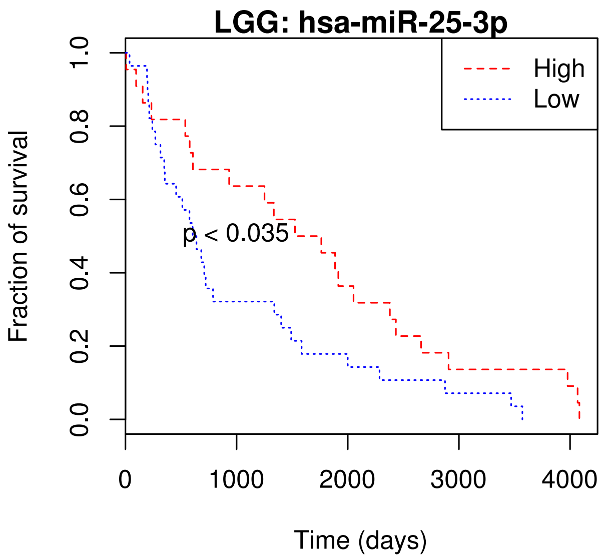
LGG: hsa-miR-25-3p
High
Low
p < 0.035
Fraction of survival
0.0
0.2
0.4
0.6
0.8
1.0
0
1000
2000
3000
4000
Time (days)

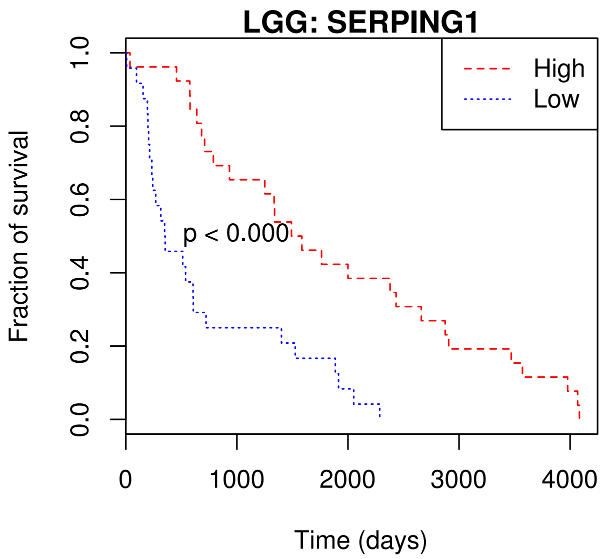
LGG: SERPING1
High
Low
p < 0.000
Fraction of survival
0.0
0.2
0.4
0.6
0.8
1.0
0
1000
2000
3000
4000
Time (days)

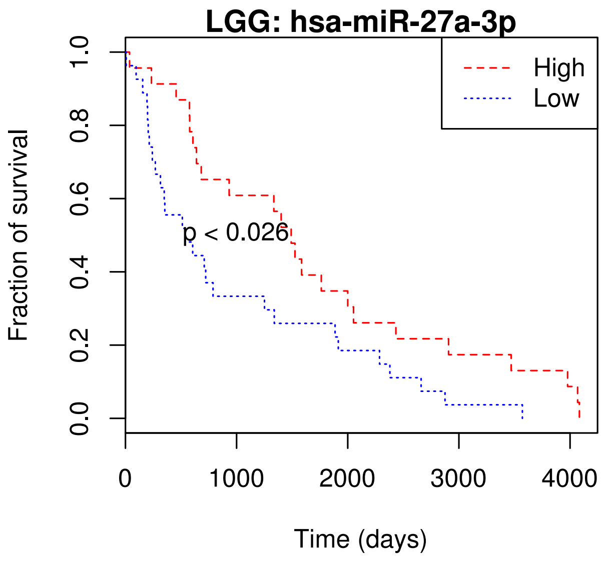
LGG: hsa-miR-27a-3p
High
Low
p < 0.026
Fraction of survival
Time (days)
0.0 0.2 0.4 0.6 0.8 1.0
0 1000 2000 3000 4000

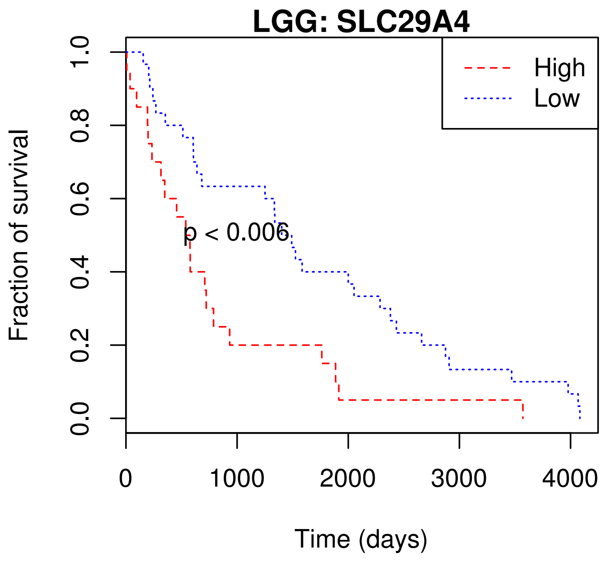
LGG: SLC29A4
High
Low
p < 0.006
Fraction of survival
Time (days)
0.0 0.2 0.4 0.6 0.8 1.0
0 1000 2000 3000 4000

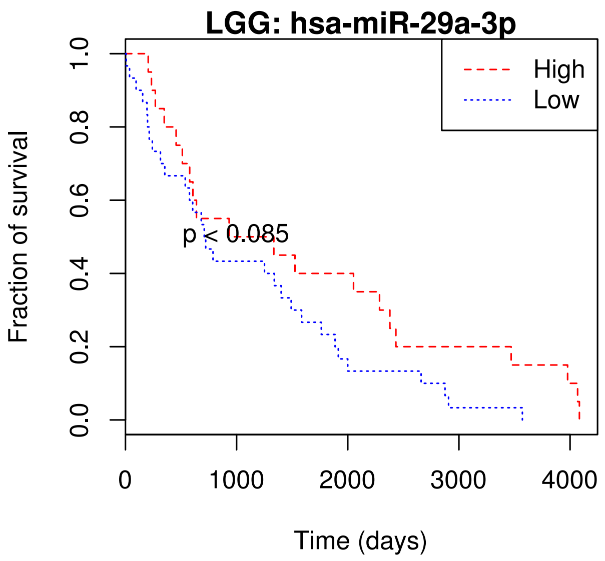
LGG: hsa-miR-29a-3p
High
Low
p < 0.085
Fraction of survival
Time (days)
0.0 0.2 0.4 0.6 0.8 1.0
0 1000 2000 3000 4000

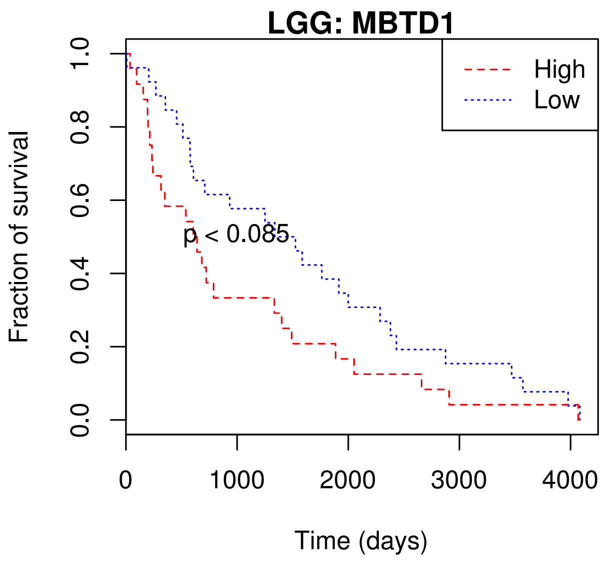
LGG: MBTD1
High
Low
p < 0.085
Fraction of survival
Time (days)
0.0 0.2 0.4 0.6 0.8 1.0
0 1000 2000 3000 4000

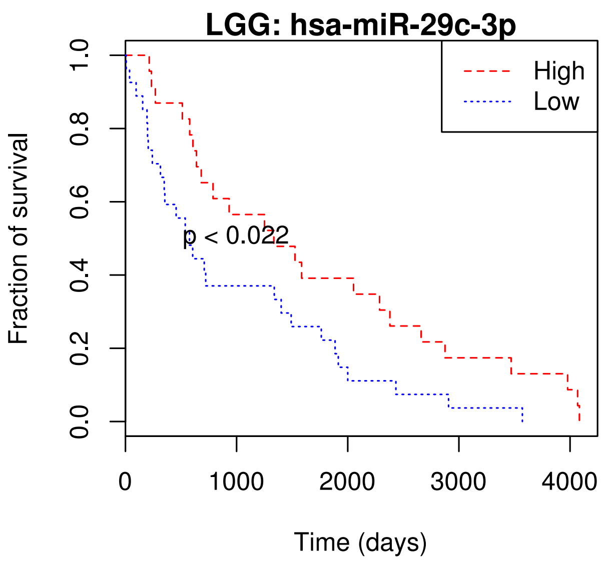
LGG: hsa-miR-29c-3p
High
Low
p < 0.022
Fraction of survival
Time (days)
0.0 0.2 0.4 0.6 0.8 1.0
0 1000 2000 3000 4000

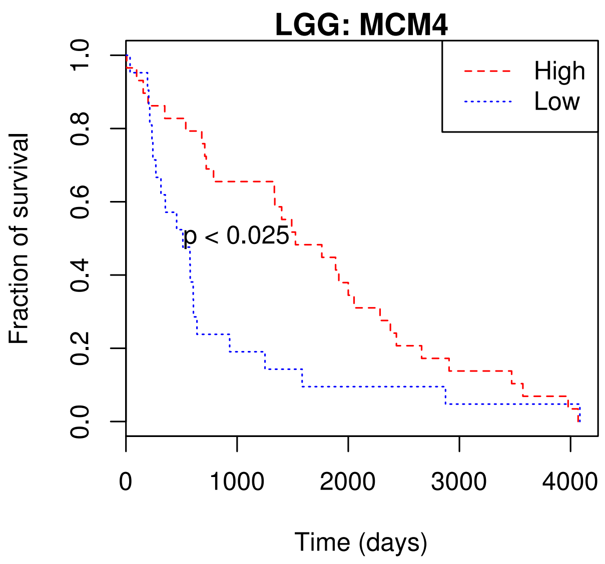
LGG: MCM4
High
Low
p < 0.025
Fraction of survival
Time (days)
0.0 0.2 0.4 0.6 0.8 1.0
0 1000 2000 3000 4000

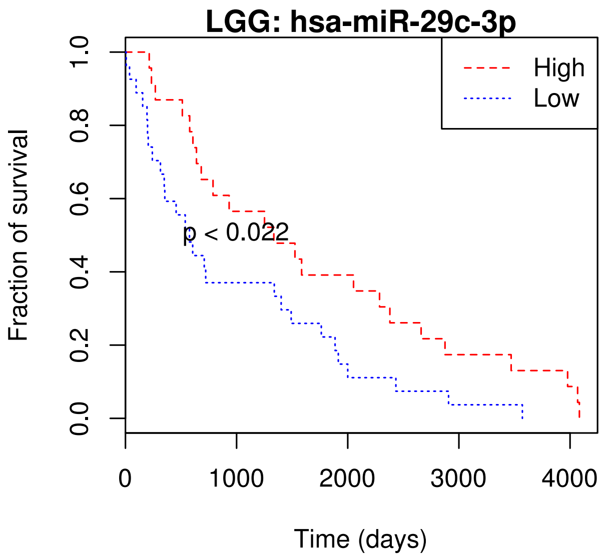
LGG: hsa-miR-29c-3p
High
Low
p < 0.022
Fraction of survival
Time (days)
0.0 0.2 0.4 0.6 0.8 1.0
0 1000 2000 3000 4000

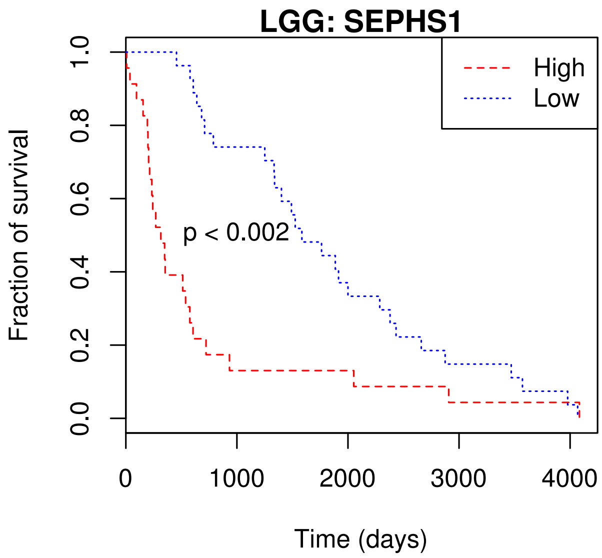
LGG: SEPHS1
High
Low
p < 0.002
Fraction of survival
Time (days)
0.0 0.2 0.4 0.6 0.8 1.0
0 1000 2000 3000 4000

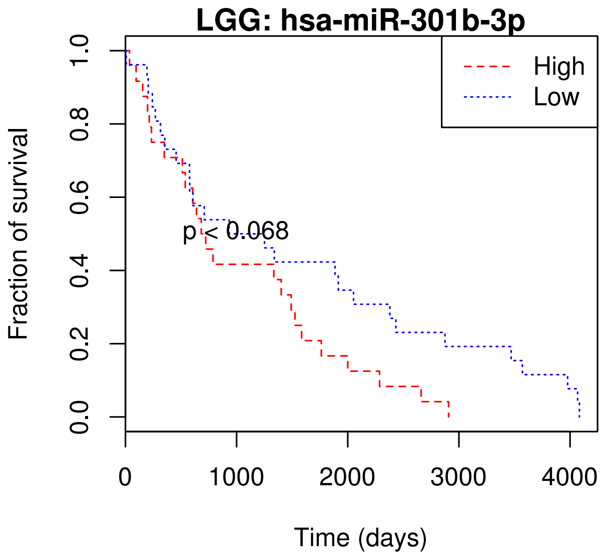
LGG: hsa-miR-301b-3p
High
Low
p < 0.068
Fraction of survival
Time (days)
0.0 0.2 0.4 0.6 0.8 1.0
0 1000 2000 3000 4000

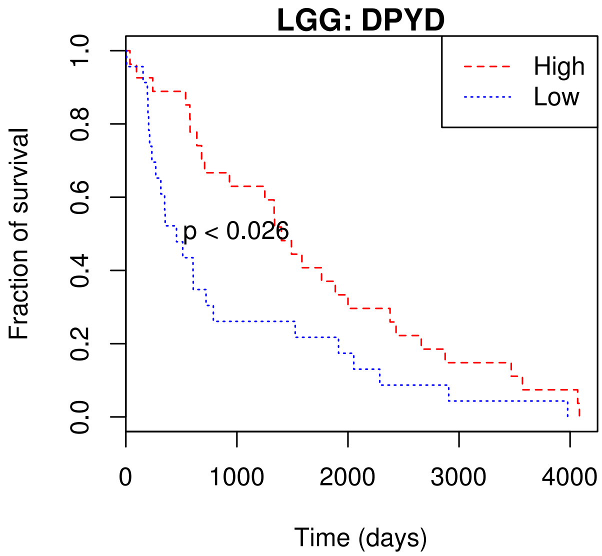
LGG: DPYD
High
Low
p < 0.026
Fraction of survival
Time (days)
0.0 0.2 0.4 0.6 0.8 1.0
0 1000 2000 3000 4000

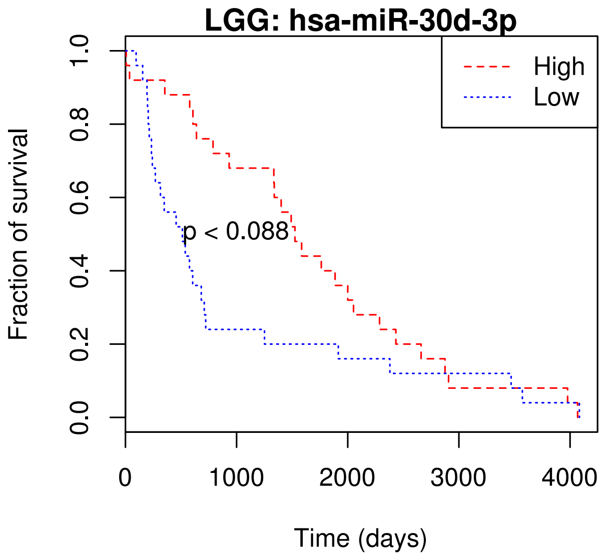
LGG: hsa-miR-30d-3p
High
Low
p < 0.088
Fraction of survival
Time (days)
0.0 0.2 0.4 0.6 0.8 1.0
0 1000 2000 3000 4000

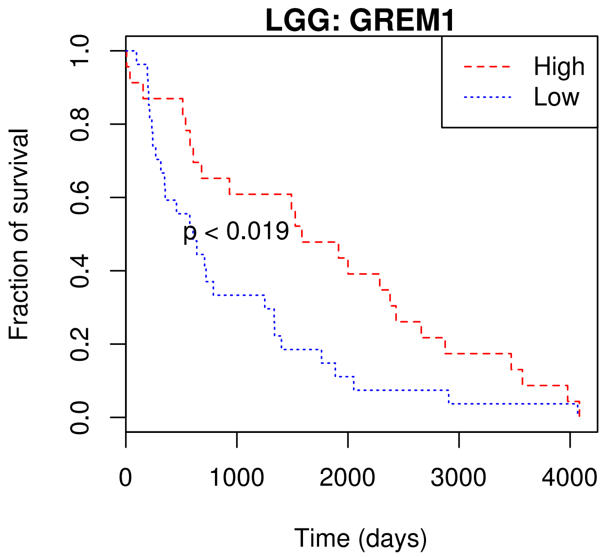
LGG: GREM1
High
Low
p < 0.019
Fraction of survival
Time (days)
0.0 0.2 0.4 0.6 0.8 1.0
0 1000 2000 3000 4000

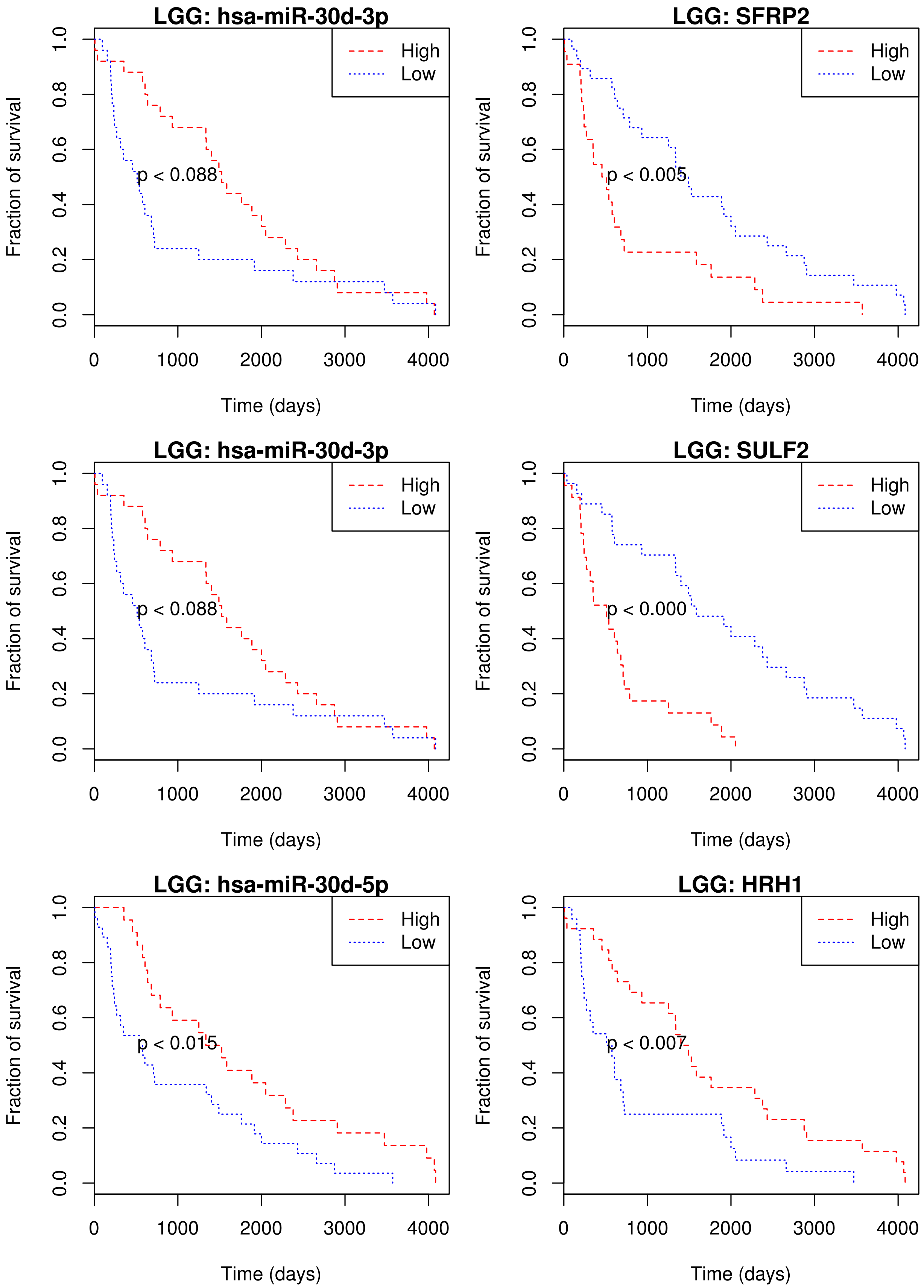

LGG: hsa-miR-30d-3p
High
Low
p < 0.088
Fraction of survival
Time (days)
LGG: SFRP2
High
Low
p < 0.005
Fraction of survival
Time (days)
LGG: hsa-miR-30d-3p
High
Low
p < 0.088
Fraction of survival
Time (days)
LGG: SULF2
High
Low
p < 0.000
Fraction of survival
Time (days)
LGG: hsa-miR-30d-5p
High
Low
p < 0.015
Fraction of survival
Time (days)
LGG: HRH1
High
Low
p < 0.007
Fraction of survival
Time (days)

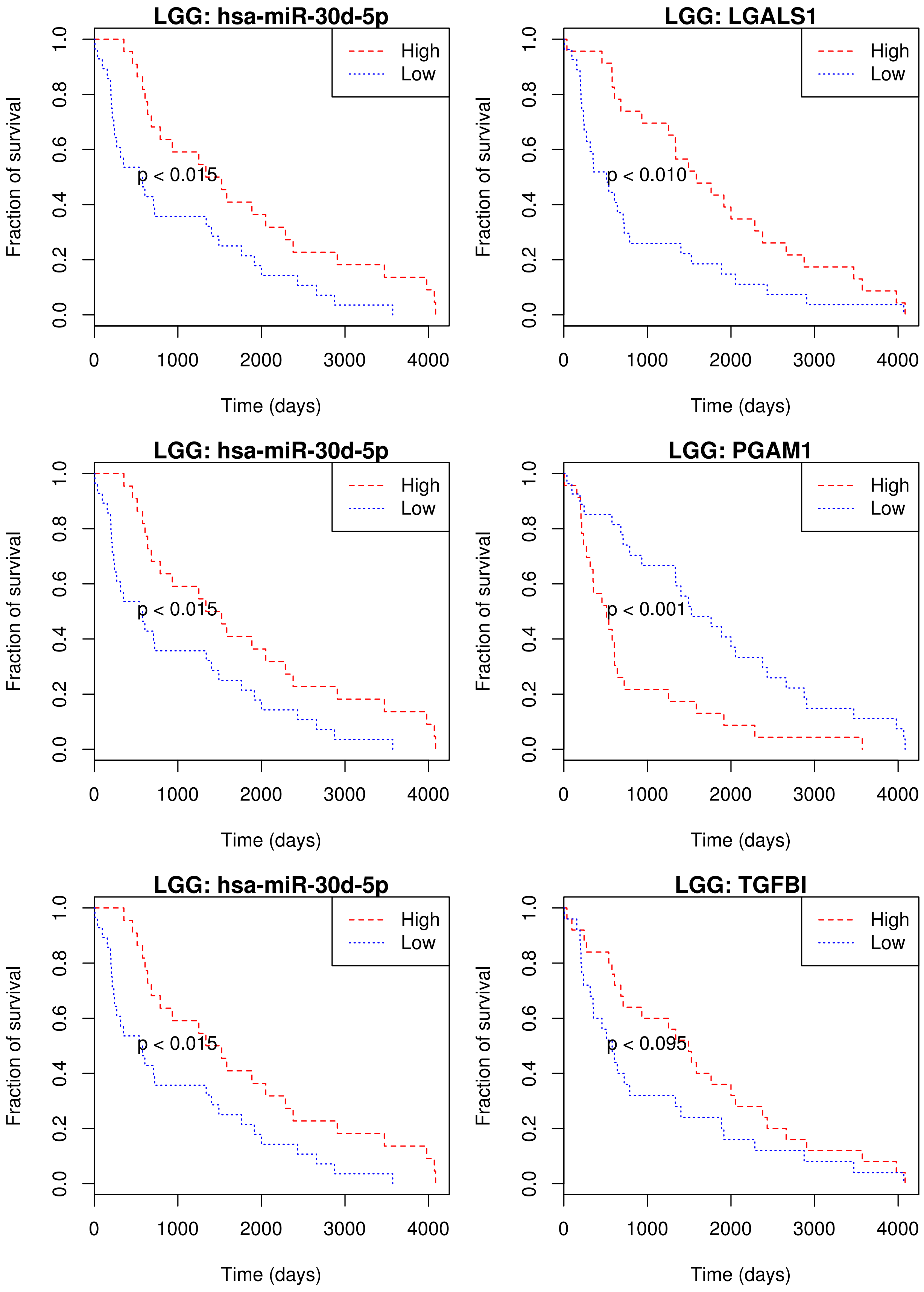
LGG: hsa-miR-30d-5p
High
Low
p < 0.015
Fraction of survival
Time (days)
LGG: LGALS1
High
Low
p < 0.010
Fraction of survival
Time (days)
LGG: hsa-miR-30d-5p
High
Low
p < 0.015
Fraction of survival
Time (days)
LGG: PGAM1
High
Low
p < 0.001
Fraction of survival
Time (days)
LGG: hsa-miR-30d-5p
High
Low
p < 0.015
Fraction of survival
Time (days)
LGG: TGFBI
High
Low
p < 0.095
Fraction of survival
Time (days)

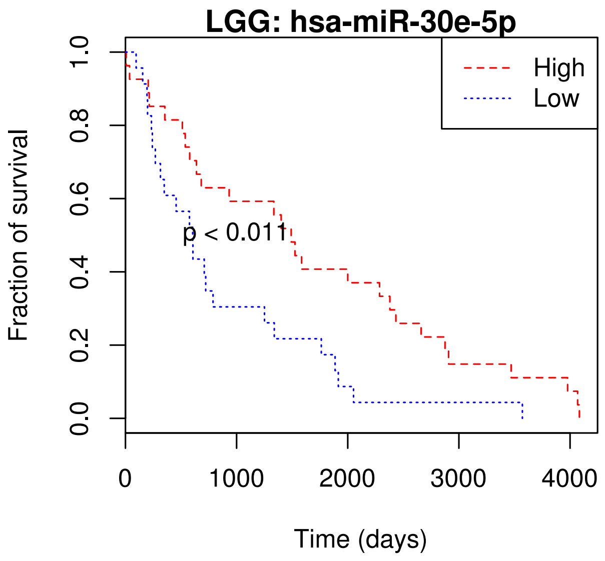
LGG: hsa-miR-30e-5p
High
Low
p < 0.011
Fraction of survival
Time (days)
0.0 0.2 0.4 0.6 0.8 1.0
0 1000 2000 3000 4000

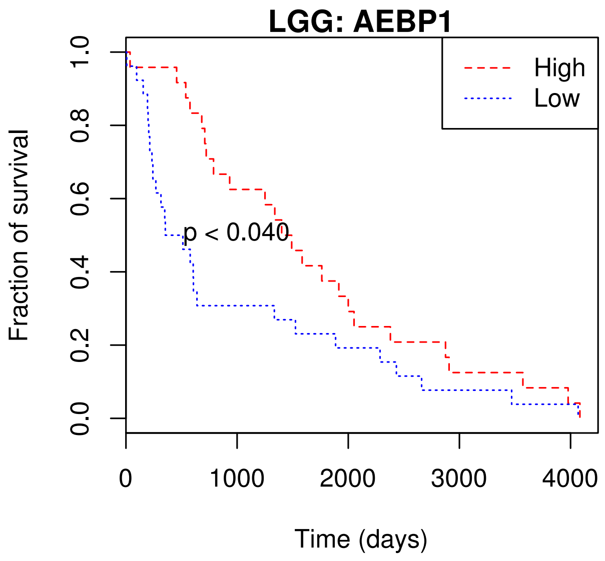
LGG: AEBP1
High
Low
p < 0.040
Fraction of survival
Time (days)
0.0 0.2 0.4 0.6 0.8 1.0
0 1000 2000 3000 4000

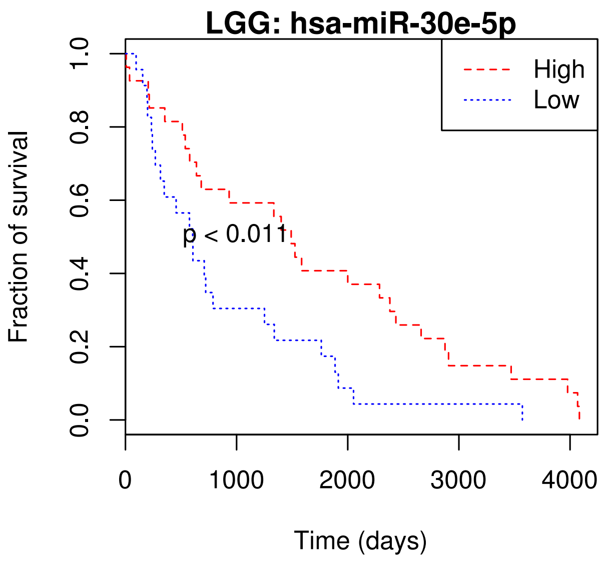
LGG: hsa-miR-30e-5p
High
Low
p < 0.011
Fraction of survival
Time (days)
0.0 0.2 0.4 0.6 0.8 1.0
0 1000 2000 3000 4000

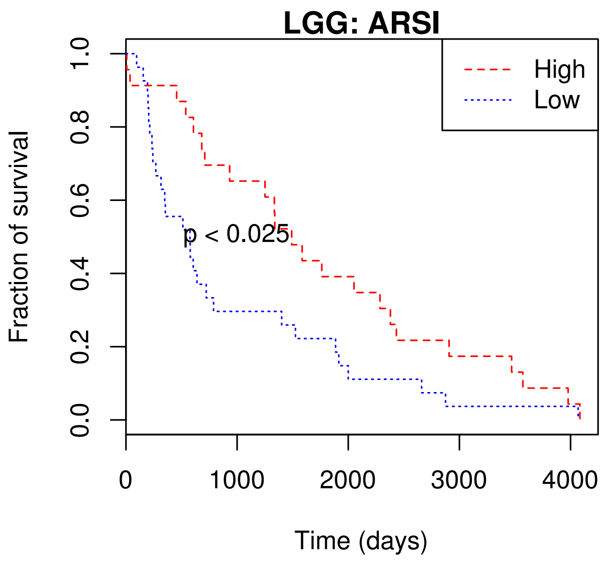
LGG: ARSI
High
Low
p < 0.025
Fraction of survival
Time (days)
0.0 0.2 0.4 0.6 0.8 1.0
0 1000 2000 3000 4000

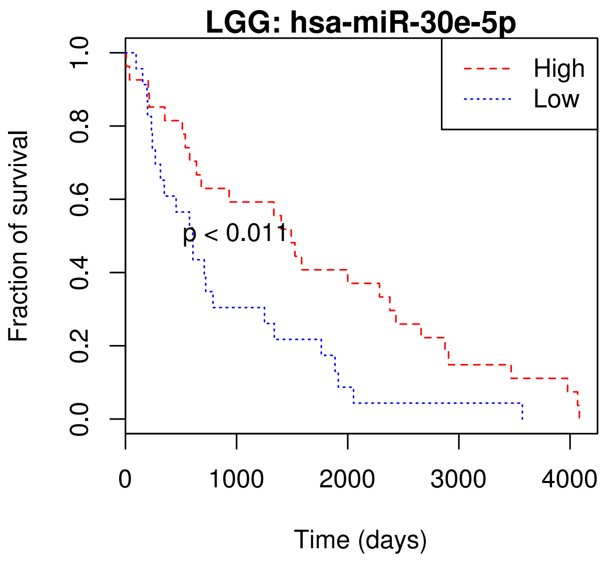
LGG: hsa-miR-30e-5p
High
Low
p < 0.011
Fraction of survival
Time (days)
0.0 0.2 0.4 0.6 0.8 1.0
0 1000 2000 3000 4000

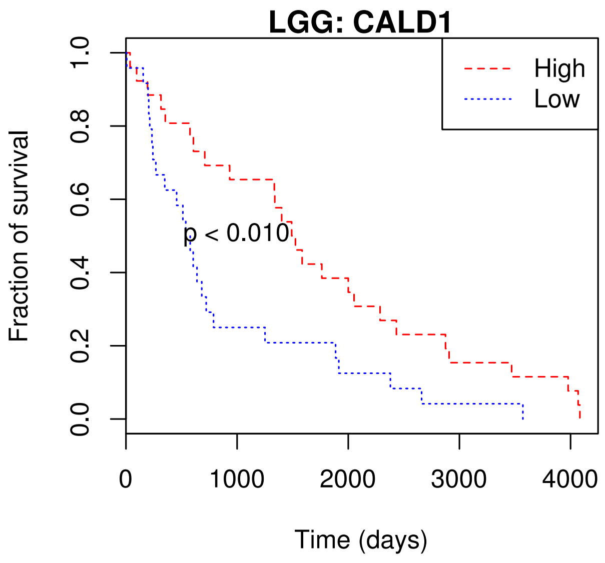
LGG: CALD1
High
Low
p < 0.010
Fraction of survival
Time (days)
0.0 0.2 0.4 0.6 0.8 1.0
0 1000 2000 3000 4000

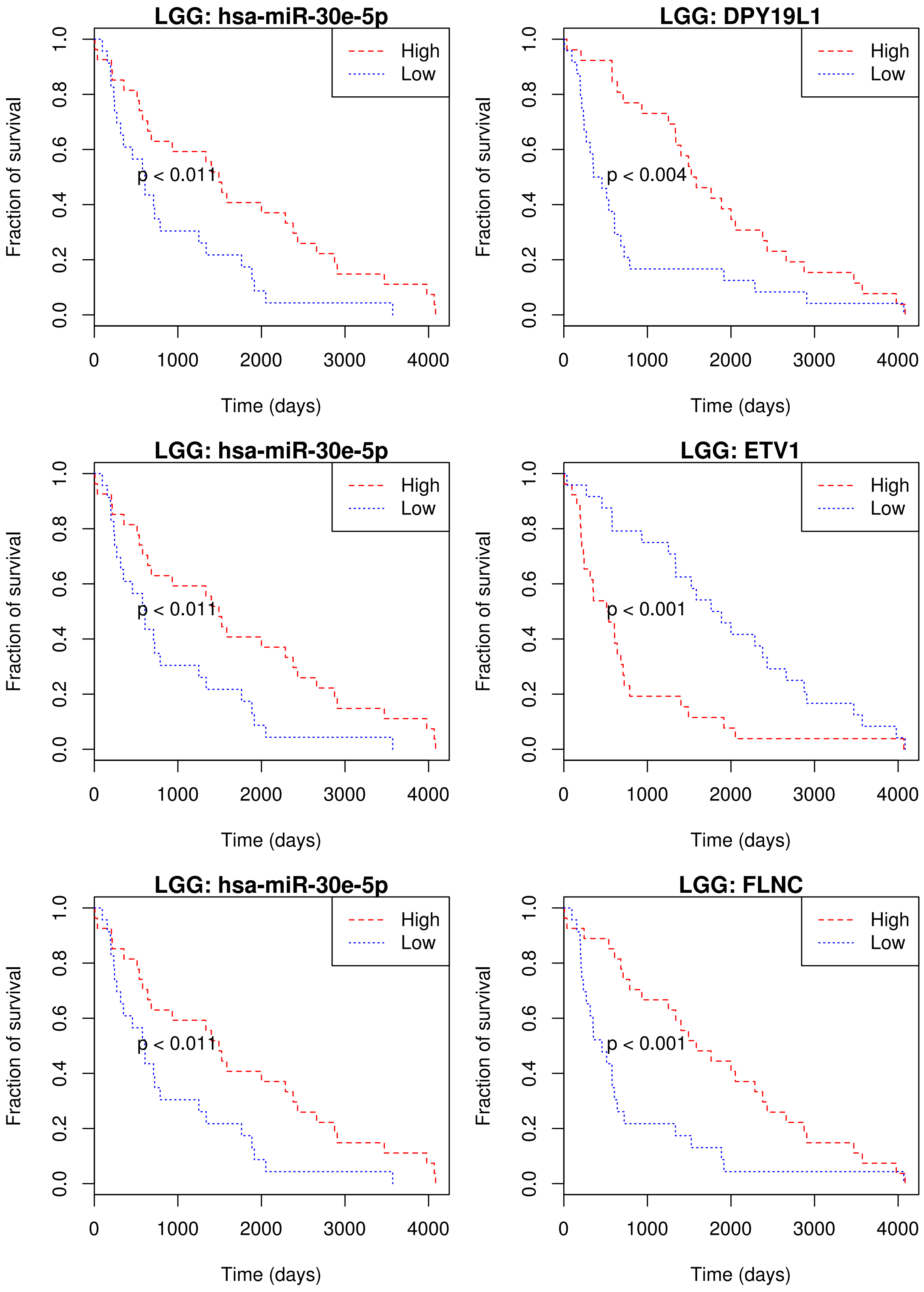
LGG: hsa-miR-30e-5p
High
Low
p < 0.011
Fraction of survival
Time (days)
LGG: DPY19L1
High
Low
p < 0.004
Fraction of survival
Time (days)
LGG: hsa-miR-30e-5p
High
Low
p < 0.011
Fraction of survival
Time (days)
LGG: ETV1
High
Low
p < 0.001
Fraction of survival
Time (days)
LGG: hsa-miR-30e-5p
High
Low
p < 0.011
Fraction of survival
Time (days)
LGG: FLNC
High
Low
p < 0.001
Fraction of survival
Time (days)

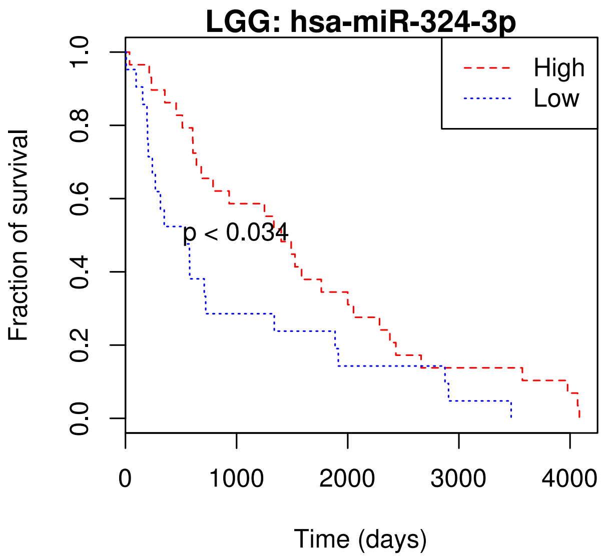
LGG: hsa-miR-324-3p
High
Low
p < 0.034
Fraction of survival
Time (days)

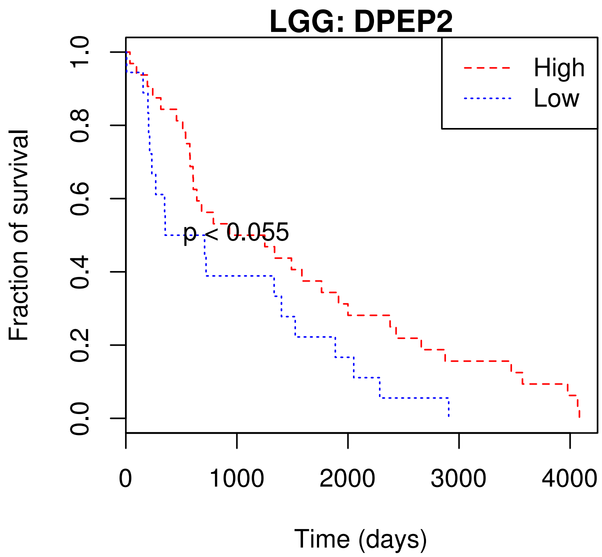
LGG: DPEP2
High
Low
p < 0.055
Fraction of survival
Time (days)

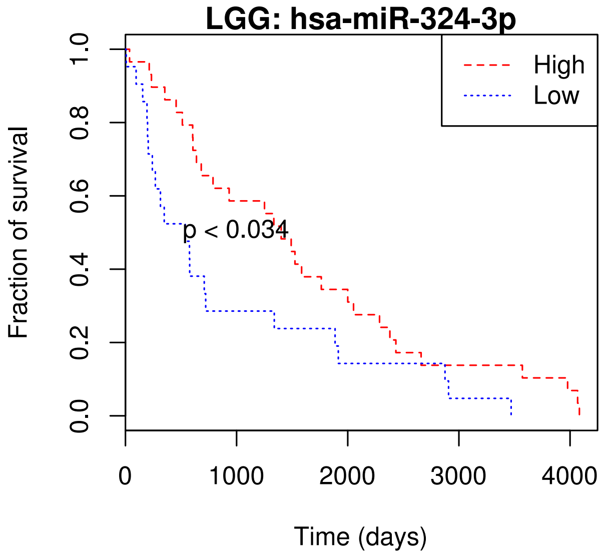
LGG: hsa-miR-324-3p
High
Low
p < 0.034
Fraction of survival
Time (days)

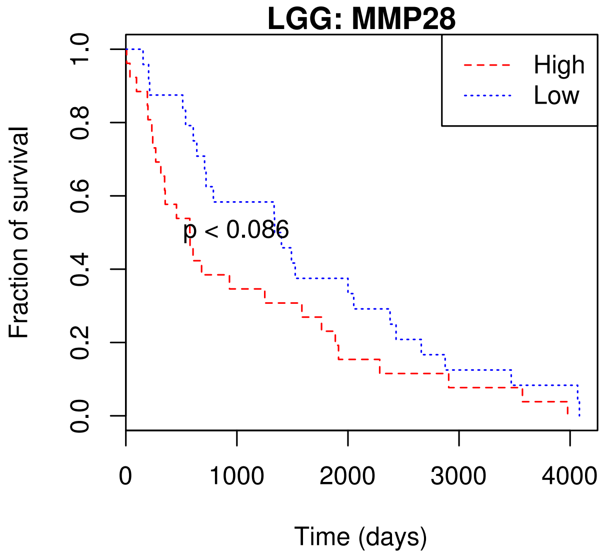
LGG: MMP28
High
Low
p < 0.086
Fraction of survival
Time (days)

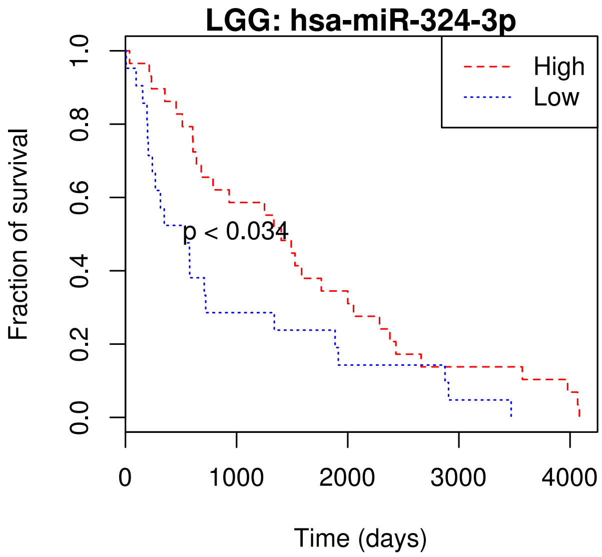
LGG: hsa-miR-324-3p
High
Low
p < 0.034
Fraction of survival
Time (days)

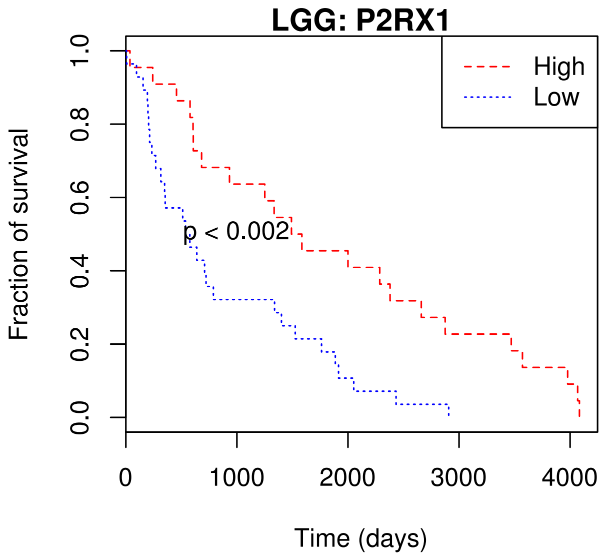
LGG: P2RX1
High
Low
p < 0.002
Fraction of survival
Time (days)

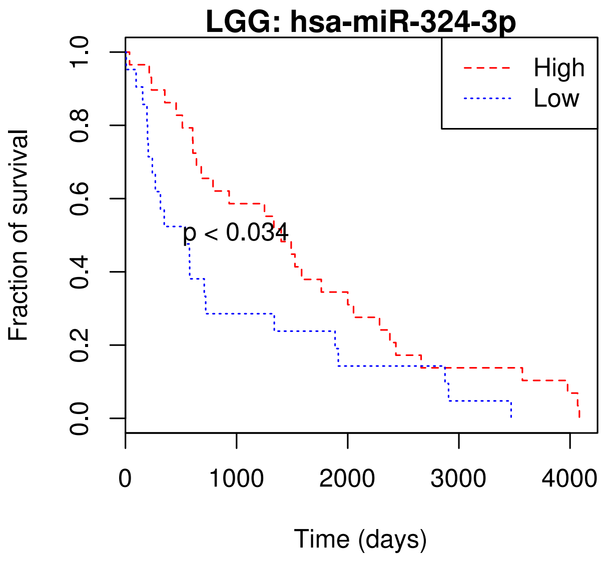
LGG: hsa-miR-324-3p
High
Low
p < 0.034
Fraction of survival
Time (days)

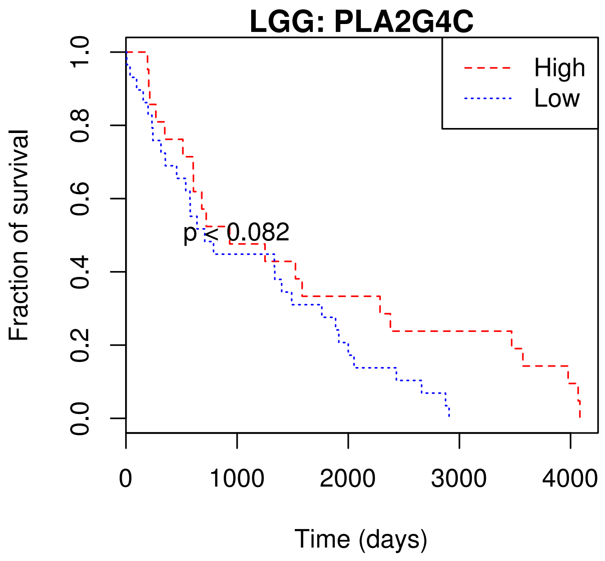
LGG: PLA2G4C
High
Low
p < 0.082
Fraction of survival
Time (days)

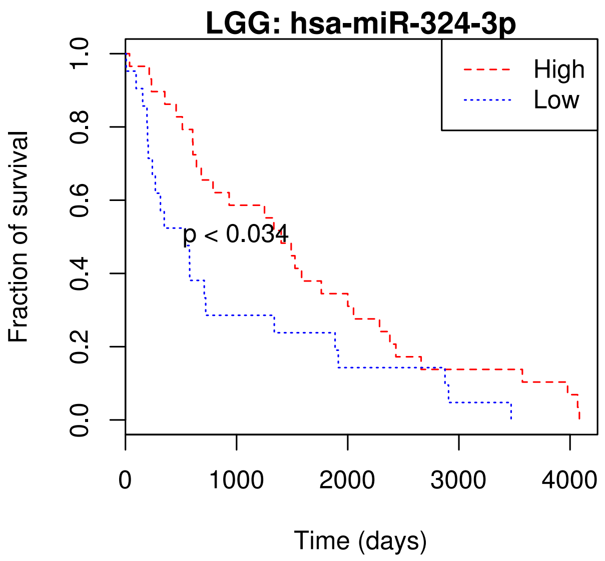
LGG: hsa-miR-324-3p
High
Low
p < 0.034
Fraction of survival
Time (days)

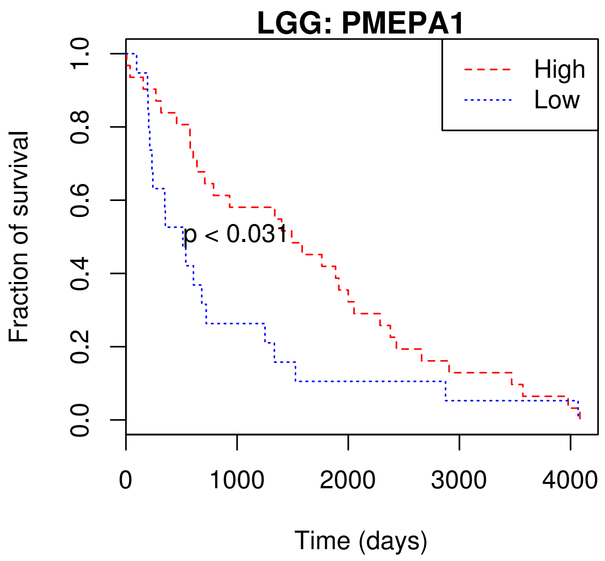
LGG: PMEPA1
High
Low
p < 0.031
Fraction of survival
Time (days)

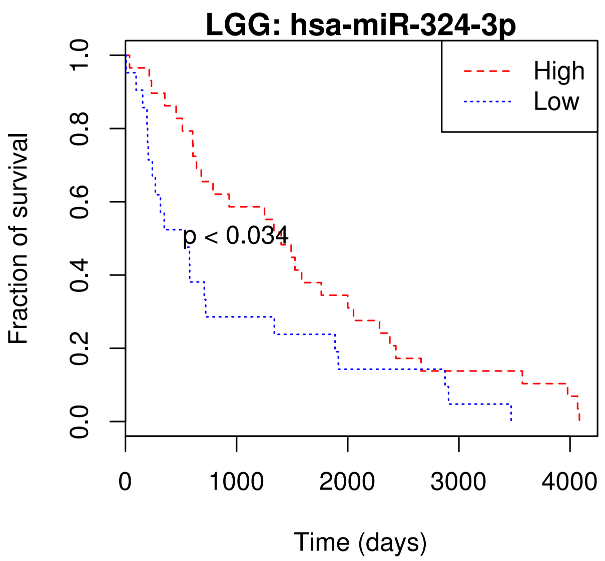
LGG: hsa-miR-324-3p
High
Low
p < 0.034
Fraction of survival
Time (days)

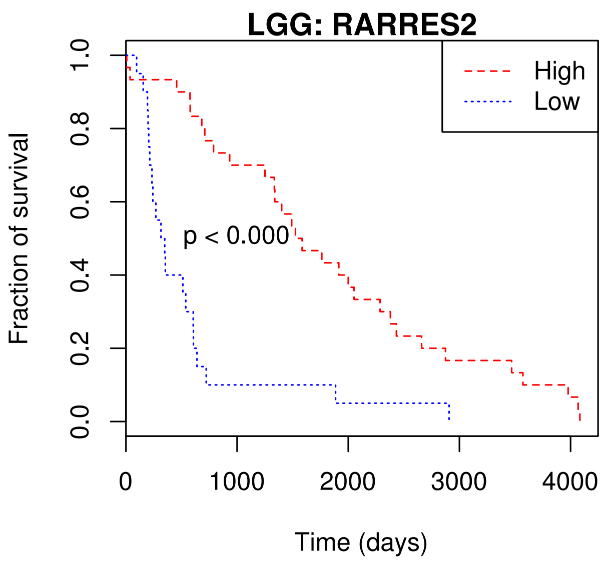
LGG: RARRES2
High
Low
p < 0.000
Fraction of survival
Time (days)

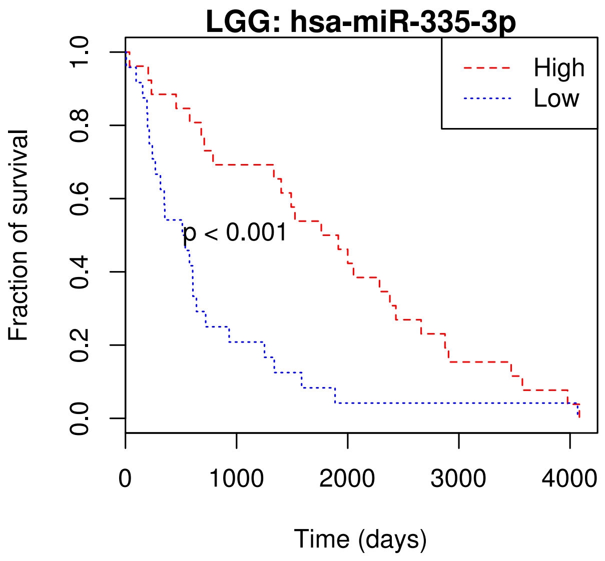
LGG: hsa-miR-335-3p
High
Low
p < 0.001
Fraction of survival
0.0
0.2
0.4
0.6
0.8
1.0
0
1000
2000
3000
4000
Time (days)

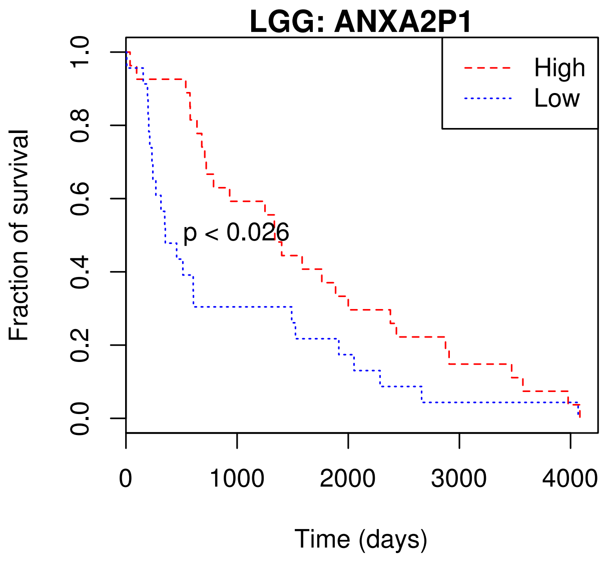
LGG: ANXA2P1
High
Low
p < 0.026
Fraction of survival
0.0
0.2
0.4
0.6
0.8
1.0
0
1000
2000
3000
4000
Time (days)

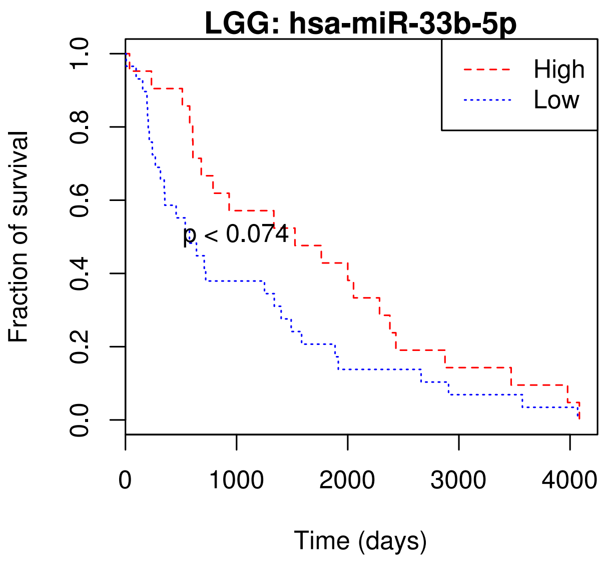
LGG: hsa-miR-33b-5p
High
Low
p < 0.074
Fraction of survival
0.0
0.2
0.4
0.6
0.8
1.0
0
1000
2000
3000
4000
Time (days)

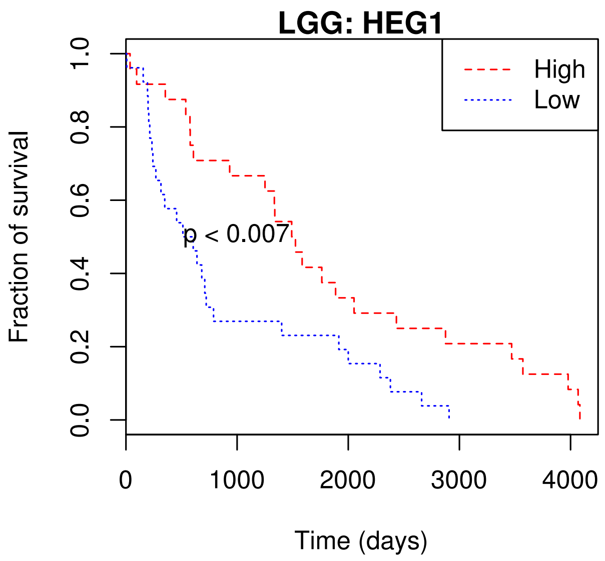
LGG: HEG1
High
Low
p < 0.007
Fraction of survival
0.0
0.2
0.4
0.6
0.8
1.0
0
1000
2000
3000
4000
Time (days)

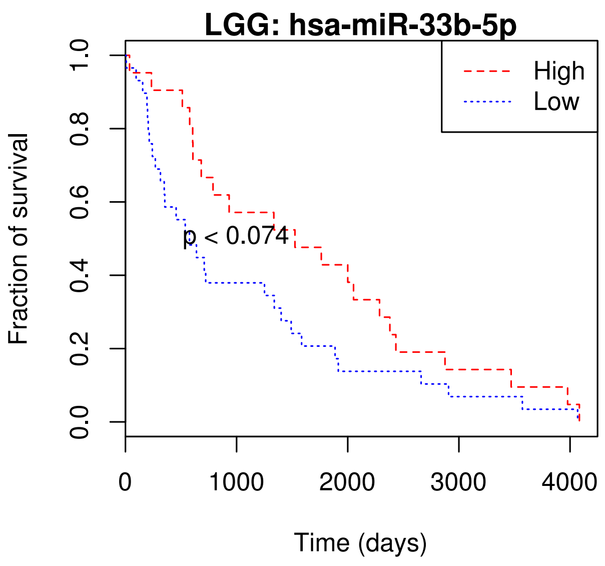
LGG: hsa-miR-33b-5p
High
Low
p < 0.074
Fraction of survival
0.0
0.2
0.4
0.6
0.8
1.0
0
1000
2000
3000
4000
Time (days)

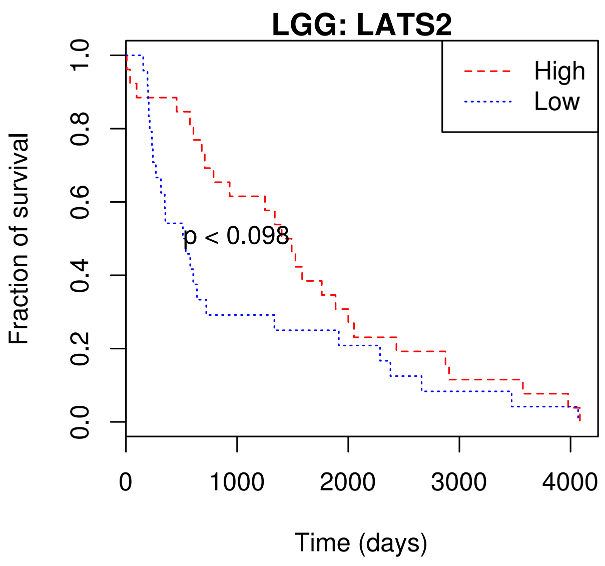
LGG: LATS2
High
Low
p < 0.098
Fraction of survival
0.0
0.2
0.4
0.6
0.8
1.0
0
1000
2000
3000
4000
Time (days)

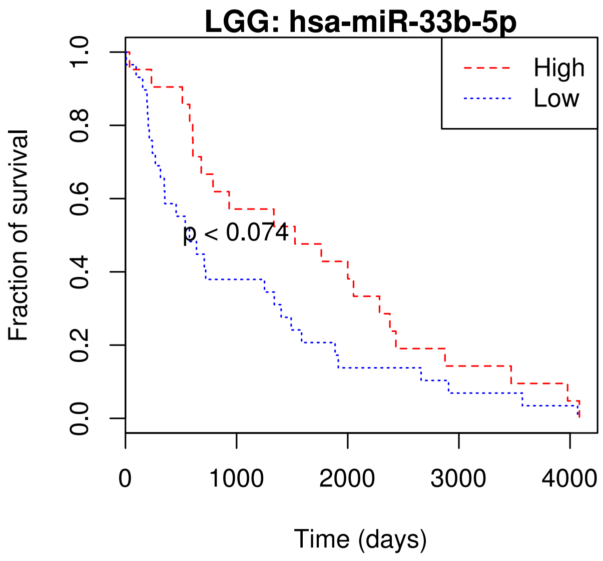
LGG: hsa-miR-33b-5p
High
Low
p < 0.074
Fraction of survival
Time (days)
0.0
0.2
0.4
0.6
0.8
1.0
0
1000
2000
3000
4000

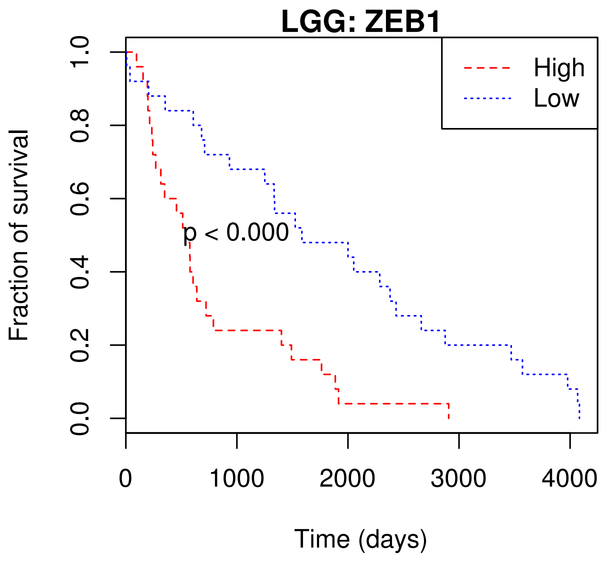
LGG: ZEB1
High
Low
p < 0.000
Fraction of survival
Time (days)
0.0
0.2
0.4
0.6
0.8
1.0
0
1000
2000
3000
4000

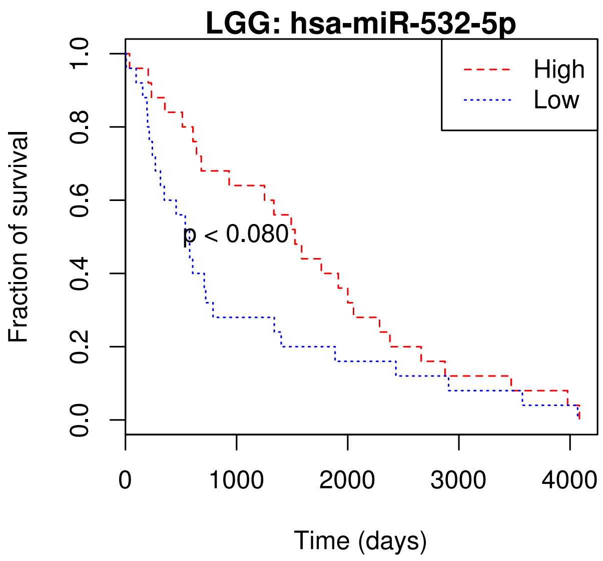
LGG: hsa-miR-532-5p
High
Low
p < 0.080
Fraction of survival
Time (days)
0.0
0.2
0.4
0.6
0.8
1.0
0
1000
2000
3000
4000

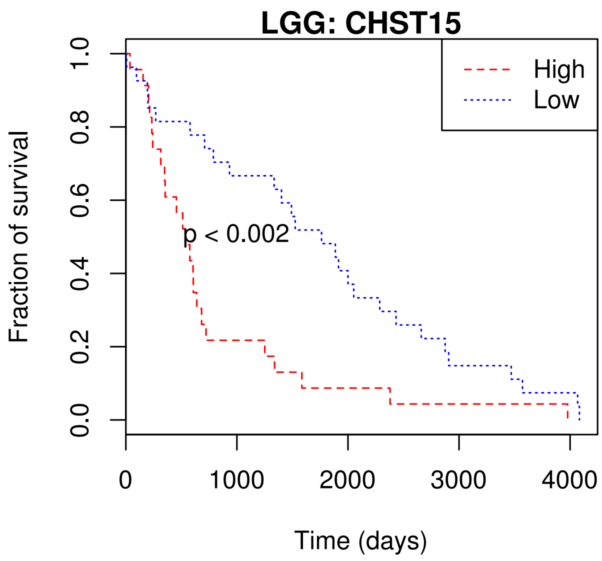
LGG: CHST15
High
Low
p < 0.002
Fraction of survival
Time (days)
0.0
0.2
0.4
0.6
0.8
1.0
0
1000
2000
3000
4000

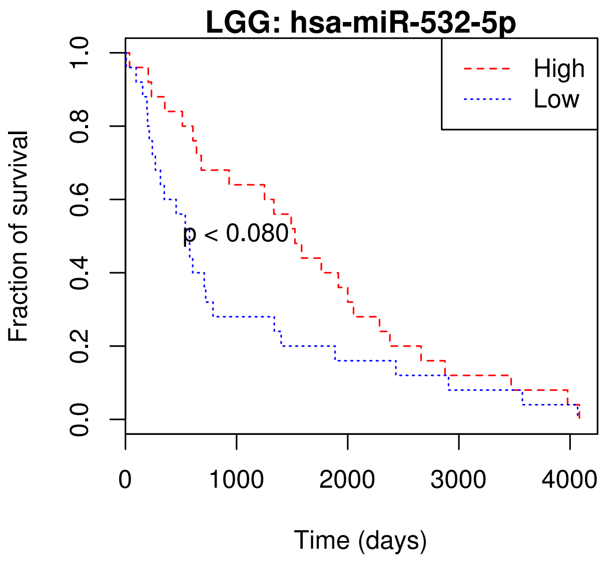
LGG: hsa-miR-532-5p
High
Low
p < 0.080
Fraction of survival
Time (days)
0.0
0.2
0.4
0.6
0.8
1.0
0
1000
2000
3000
4000

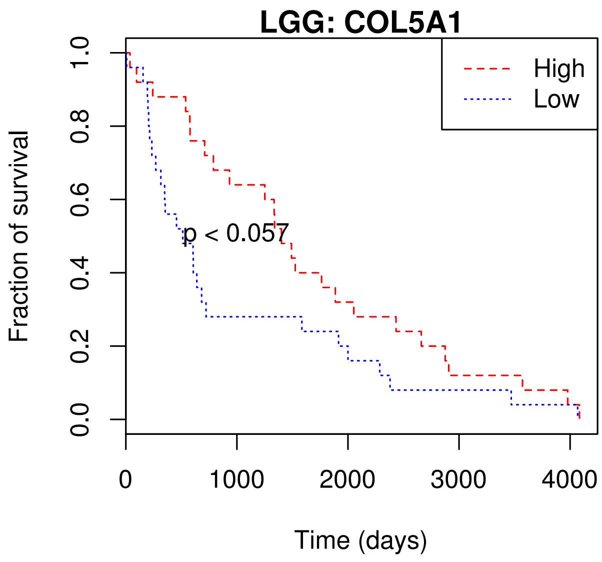
LGG: COL5A1
High
Low
p < 0.057
Fraction of survival
Time (days)
0.0
0.2
0.4
0.6
0.8
1.0
0
1000
2000
3000
4000

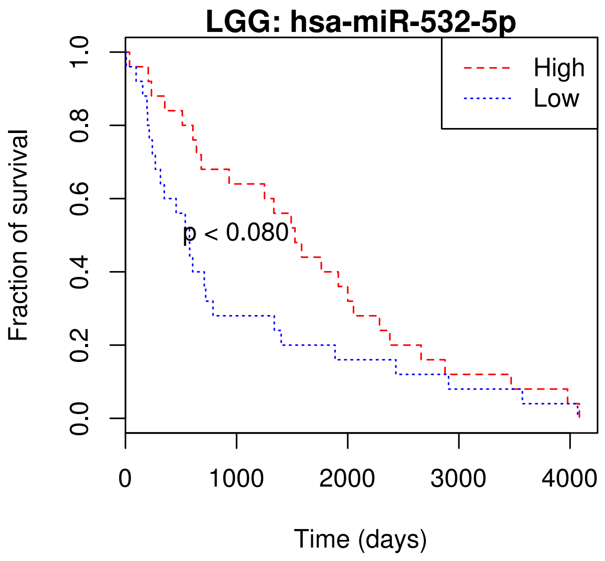
LGG: hsa-miR-532-5p
High
Low
p < 0.080
Fraction of survival
Time (days)
0.0 0.2 0.4 0.6 0.8 1.0
0 1000 2000 3000 4000

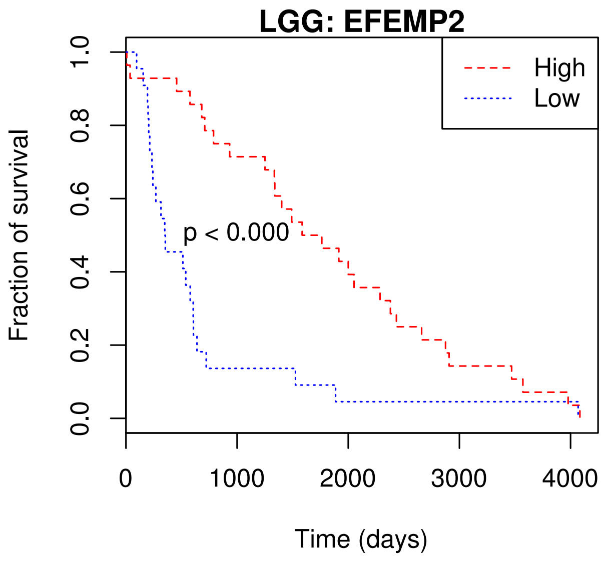
LGG: EFEMP2
High
Low
p < 0.000
Fraction of survival
Time (days)
0.0 0.2 0.4 0.6 0.8 1.0
0 1000 2000 3000 4000

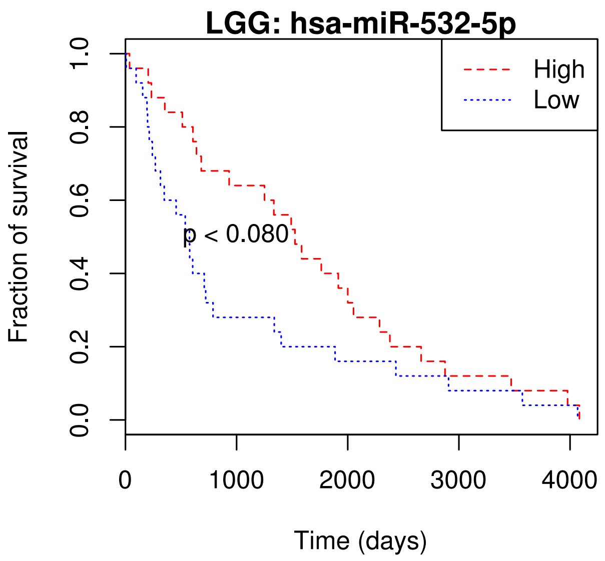
LGG: hsa-miR-532-5p
High
Low
p < 0.080
Fraction of survival
Time (days)
0.0 0.2 0.4 0.6 0.8 1.0
0 1000 2000 3000 4000

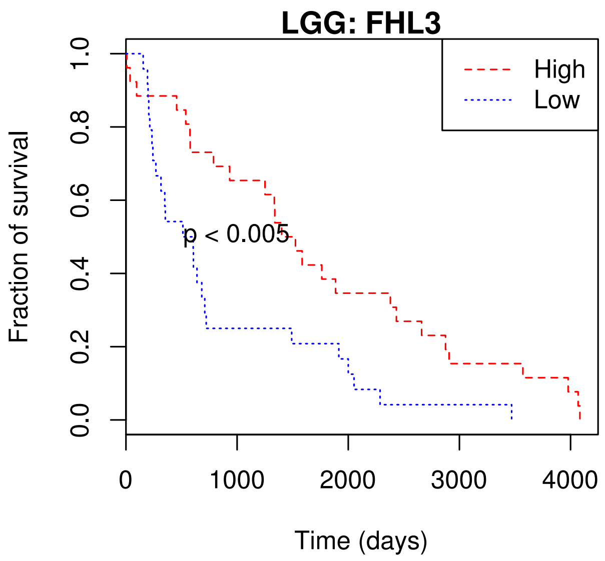
LGG: FHL3
High
Low
p < 0.005
Fraction of survival
Time (days)
0.0 0.2 0.4 0.6 0.8 1.0
0 1000 2000 3000 4000

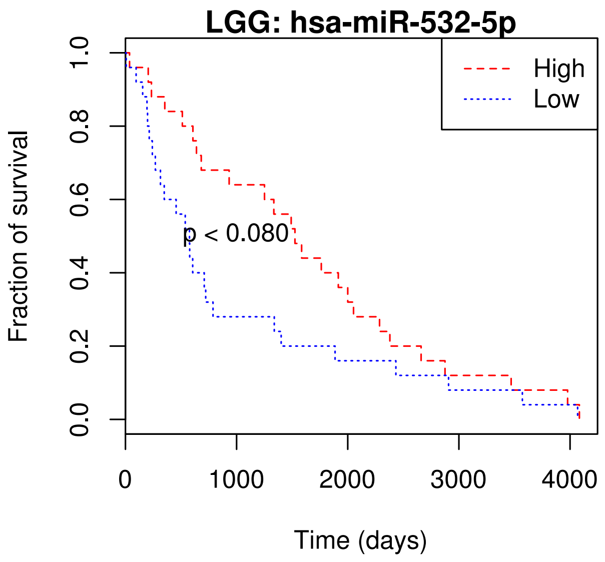
LGG: hsa-miR-532-5p
High
Low
p < 0.080
Fraction of survival
Time (days)
0.0 0.2 0.4 0.6 0.8 1.0
0 1000 2000 3000 4000

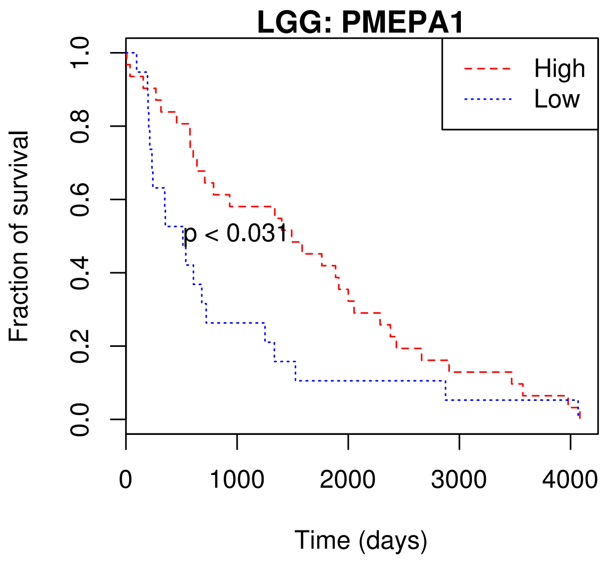
LGG: PMEPA1
High
Low
p < 0.031
Fraction of survival
Time (days)
0.0 0.2 0.4 0.6 0.8 1.0
0 1000 2000 3000 4000

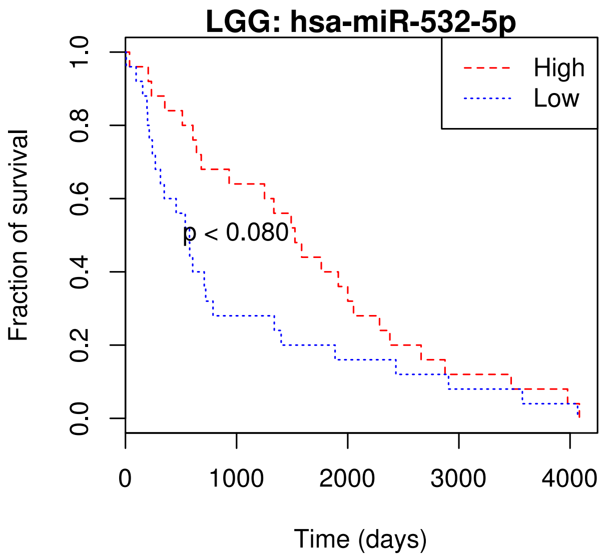
LGG: hsa-miR-532-5p
High
Low
p < 0.080
Fraction of survival
Time (days)
0.0 0.2 0.4 0.6 0.8 1.0
0 1000 2000 3000 4000

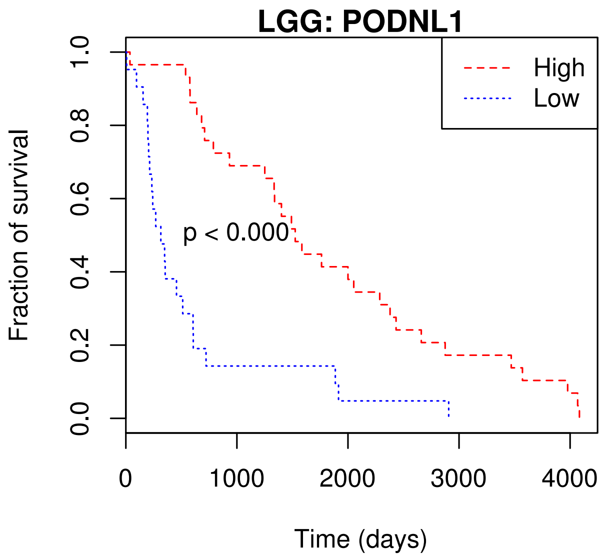
LGG: PODNL1
High
Low
p < 0.000
Fraction of survival
Time (days)
0.0 0.2 0.4 0.6 0.8 1.0
0 1000 2000 3000 4000

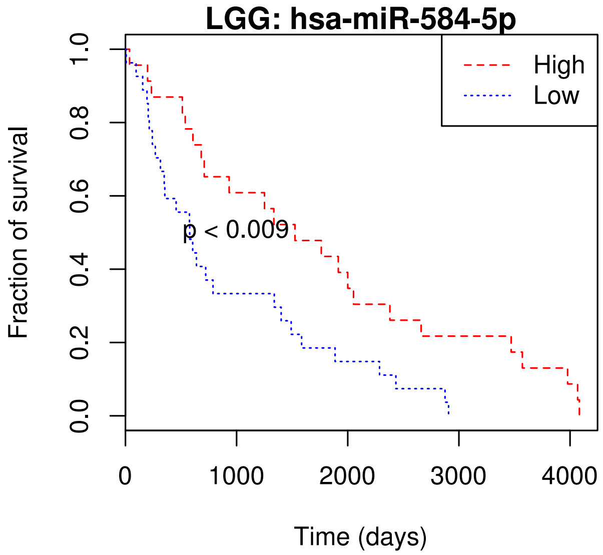
LGG: hsa-miR-584-5p
High
Low
p < 0.009
Fraction of survival
Time (days)
0.0 0.2 0.4 0.6 0.8 1.0
0 1000 2000 3000 4000

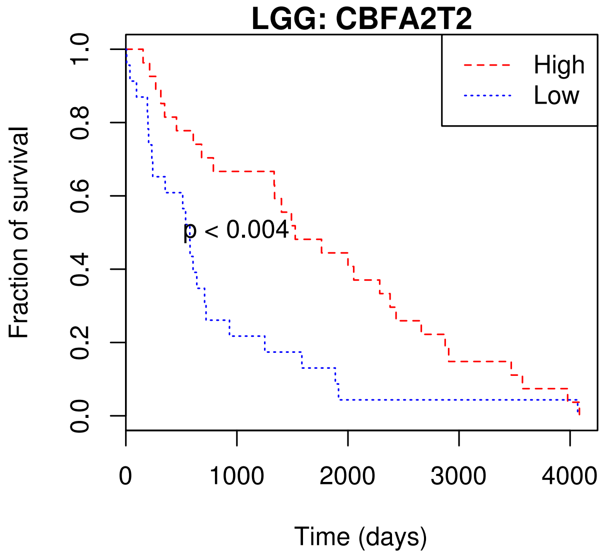
LGG: CBFA2T2
High
Low
p < 0.004
Fraction of survival
Time (days)
0.0 0.2 0.4 0.6 0.8 1.0
0 1000 2000 3000 4000

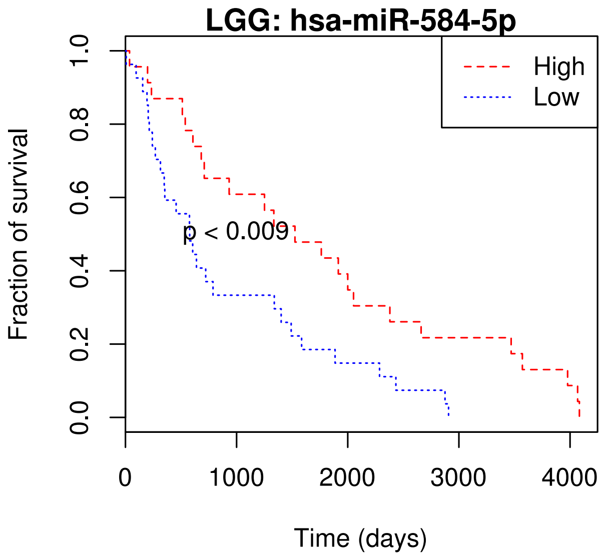
LGG: hsa-miR-584-5p
High
Low
p < 0.009
Fraction of survival
Time (days)
0.0 0.2 0.4 0.6 0.8 1.0
0 1000 2000 3000 4000

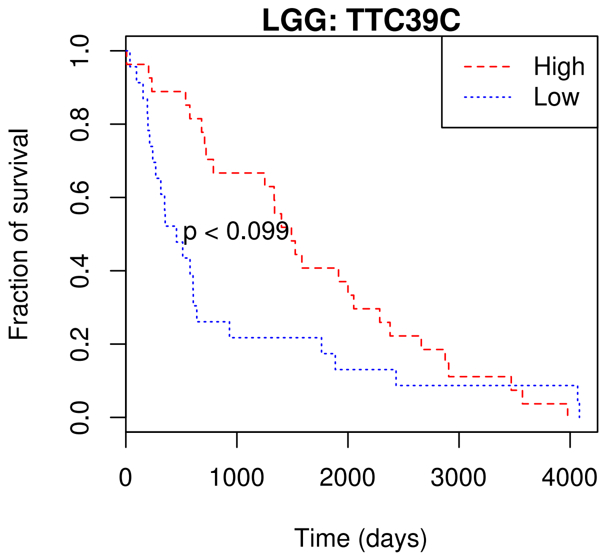
LGG: TTC39C
High
Low
p < 0.099
Fraction of survival
Time (days)
0.0 0.2 0.4 0.6 0.8 1.0
0 1000 2000 3000 4000

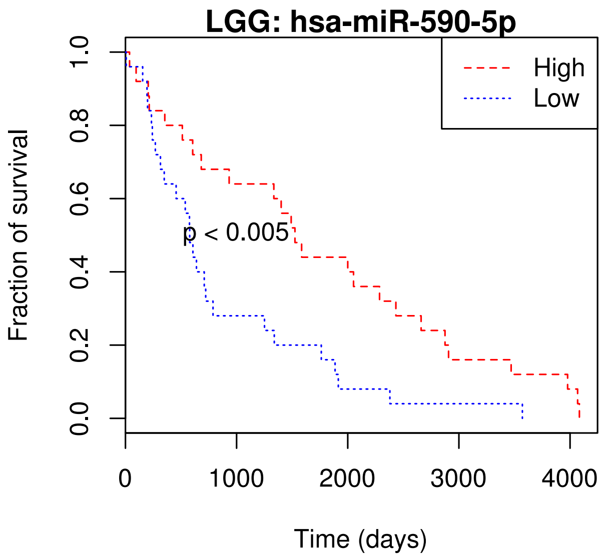
LGG: hsa-miR-590-5p
High
Low
p < 0.005
Fraction of survival
0.0
0.2
0.4
0.6
0.8
1.0
0
1000
2000
3000
4000
Time (days)

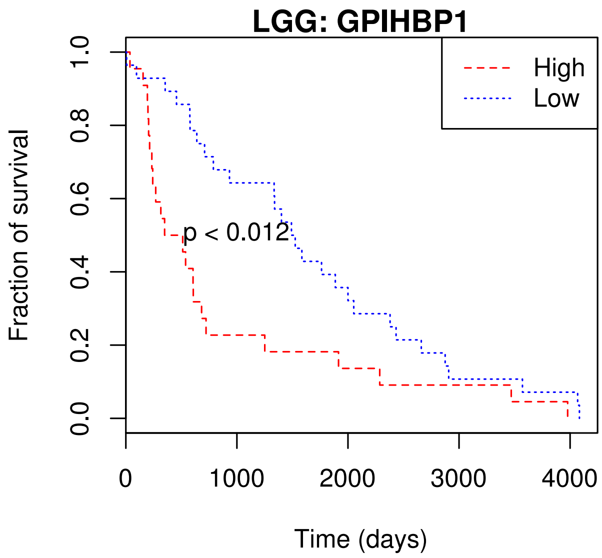
LGG: GPIHBP1
High
Low
p < 0.012
Fraction of survival
0.0
0.2
0.4
0.6
0.8
1.0
0
1000
2000
3000
4000
Time (days)

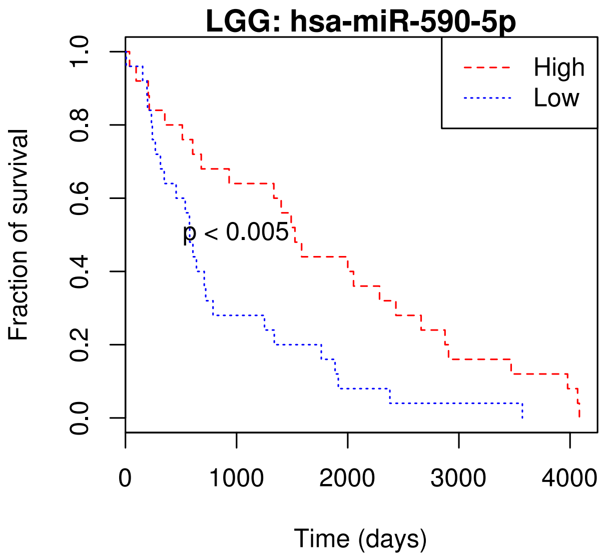
LGG: hsa-miR-590-5p
High
Low
p < 0.005
Fraction of survival
0.0
0.2
0.4
0.6
0.8
1.0
0
1000
2000
3000
4000
Time (days)

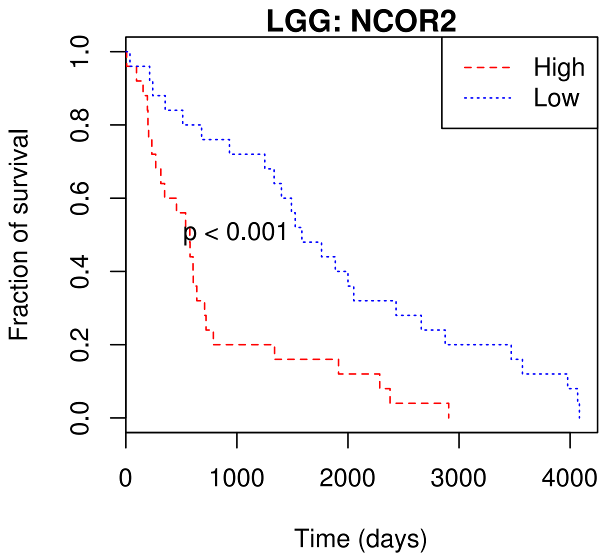
LGG: NCOR2
High
Low
p < 0.001
Fraction of survival
0.0
0.2
0.4
0.6
0.8
1.0
0
1000
2000
3000
4000
Time (days)

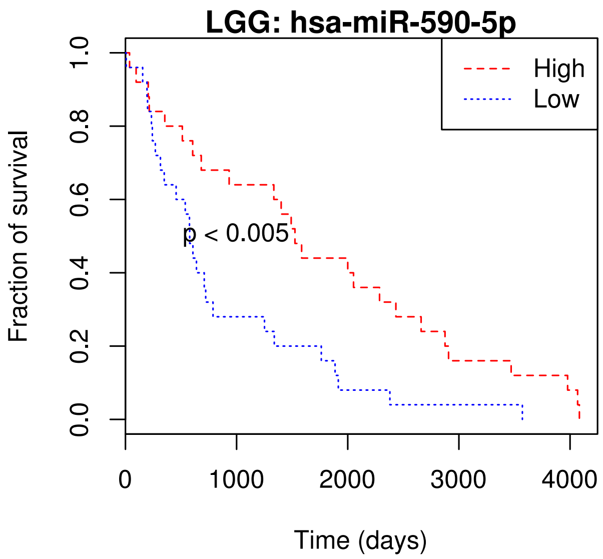
LGG: hsa-miR-590-5p
High
Low
p < 0.005
Fraction of survival
0.0
0.2
0.4
0.6
0.8
1.0
0
1000
2000
3000
4000
Time (days)

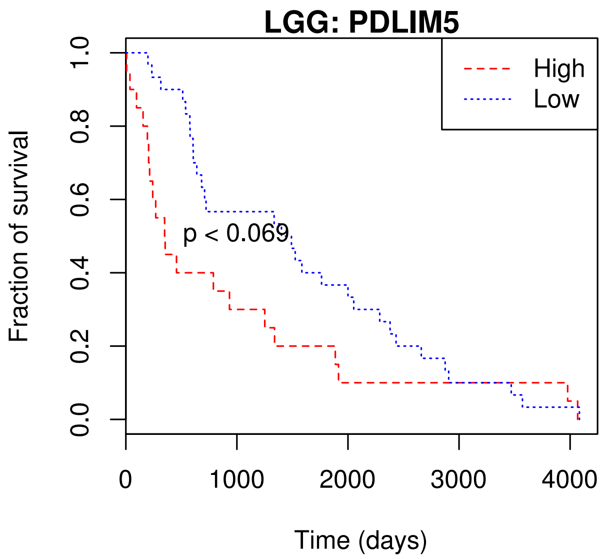
LGG: PDLIM5
High
Low
p < 0.069
Fraction of survival
0.0
0.2
0.4
0.6
0.8
1.0
0
1000
2000
3000
4000
Time (days)

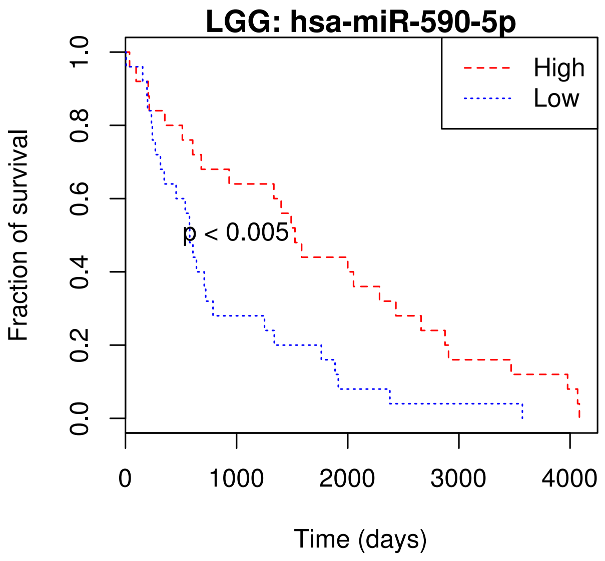
LGG: hsa-miR-590-5p
High
Low
p < 0.005
Fraction of survival
Time (days)
0.0 0.2 0.4 0.6 0.8 1.0
0 1000 2000 3000 4000

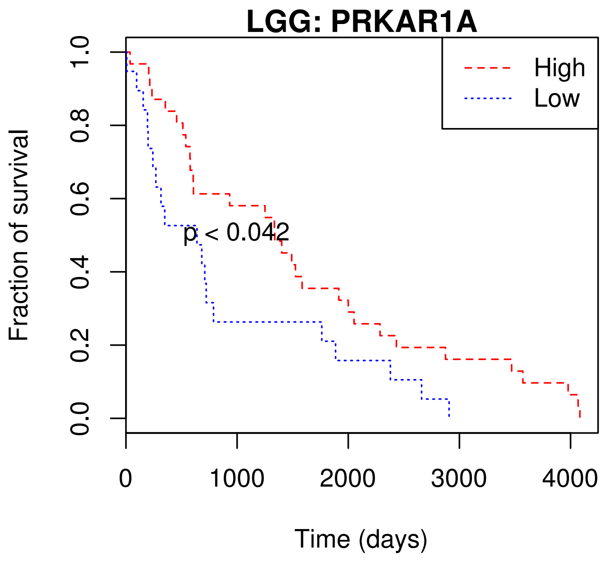
LGG: PRKAR1A
High
Low
p < 0.042
Fraction of survival
Time (days)
0.0 0.2 0.4 0.6 0.8 1.0
0 1000 2000 3000 4000

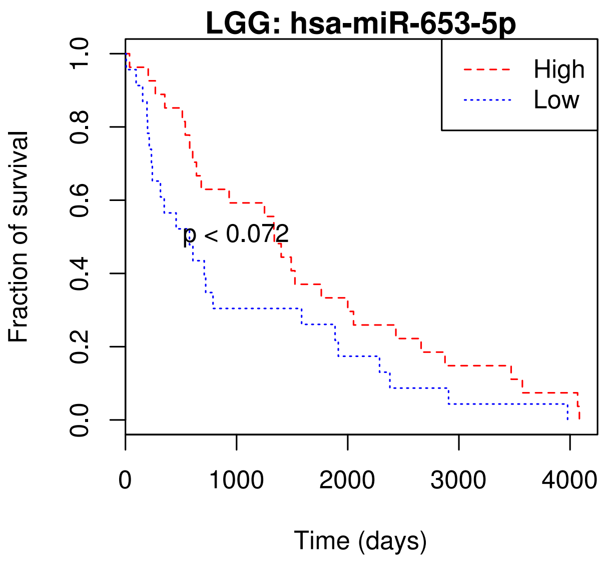
LGG: hsa-miR-653-5p
High
Low
p < 0.072
Fraction of survival
Time (days)
0.0 0.2 0.4 0.6 0.8 1.0
0 1000 2000 3000 4000

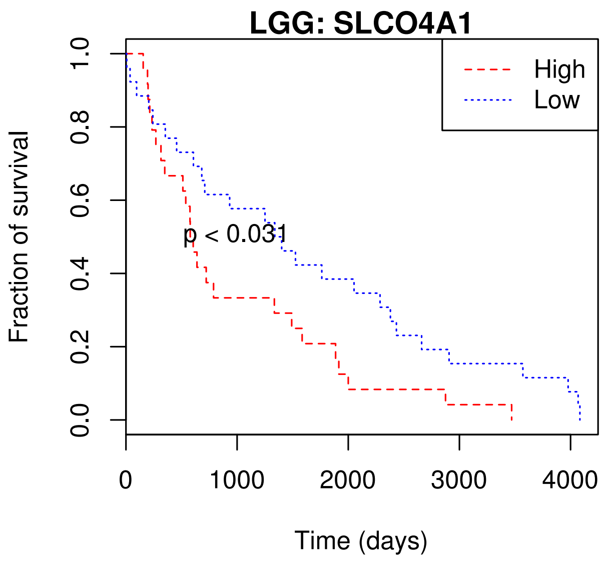
LGG: SLCO4A1
High
Low
p < 0.031
Fraction of survival
Time (days)
0.0 0.2 0.4 0.6 0.8 1.0
0 1000 2000 3000 4000

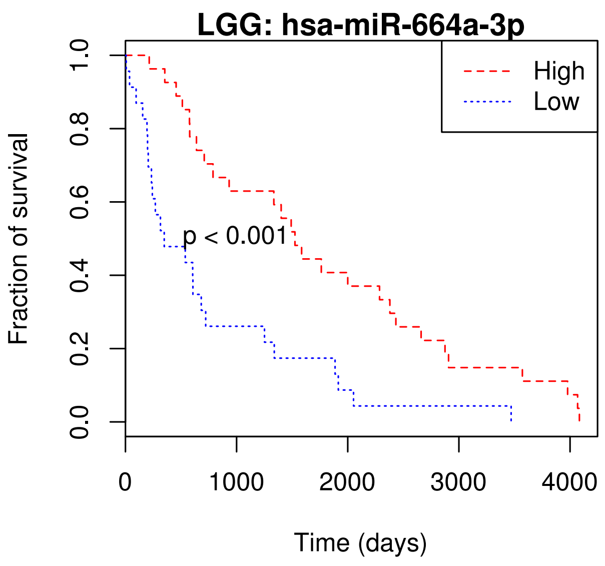
LGG: hsa-miR-664a-3p
High
Low
p < 0.001
Fraction of survival
Time (days)
0.0 0.2 0.4 0.6 0.8 1.0
0 1000 2000 3000 4000

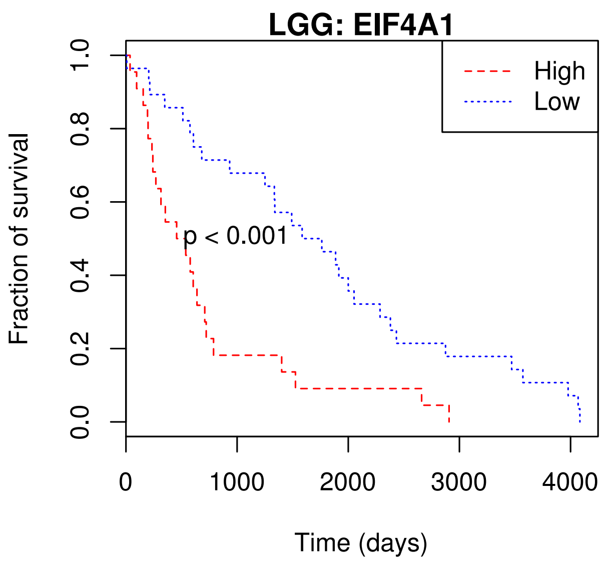
LGG: EIF4A1
High
Low
p < 0.001
Fraction of survival
Time (days)
0.0 0.2 0.4 0.6 0.8 1.0
0 1000 2000 3000 4000

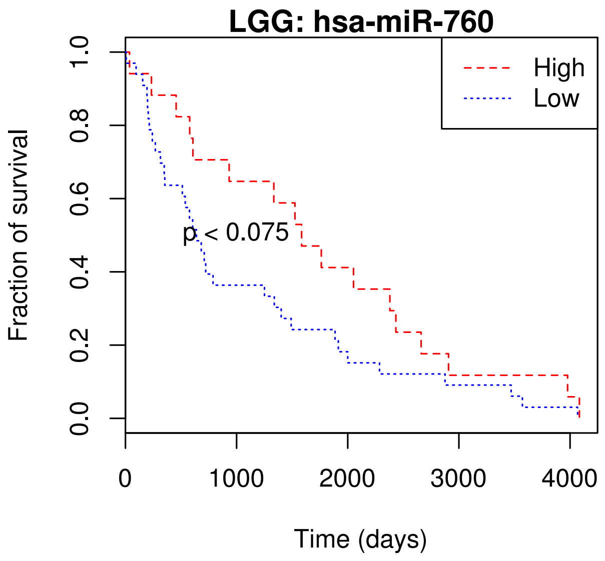
LGG: hsa-miR-760
High
Low
p < 0.075
Fraction of survival
Time (days)
0.0
0.2
0.4
0.6
0.8
1.0
0
1000
2000
3000
4000

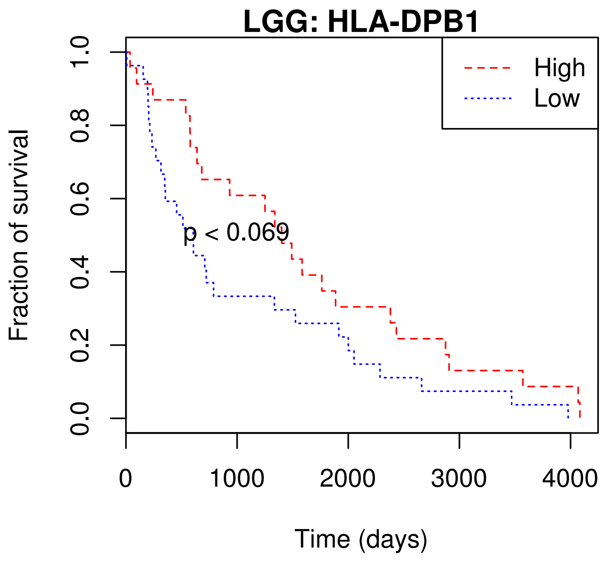
LGG: HLA-DPB1
High
Low
p < 0.069
Fraction of survival
Time (days)
0.0
0.2
0.4
0.6
0.8
1.0
0
1000
2000
3000
4000

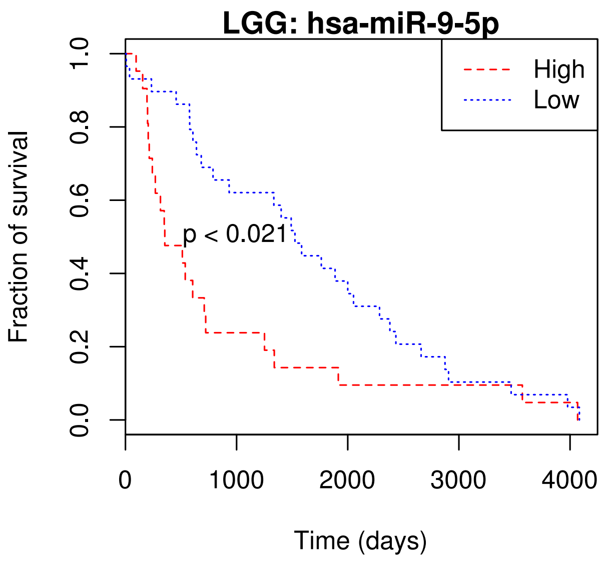
LGG: hsa-miR-9-5p
High
Low
p < 0.021
Fraction of survival
Time (days)
0.0
0.2
0.4
0.6
0.8
1.0
0
1000
2000
3000
4000

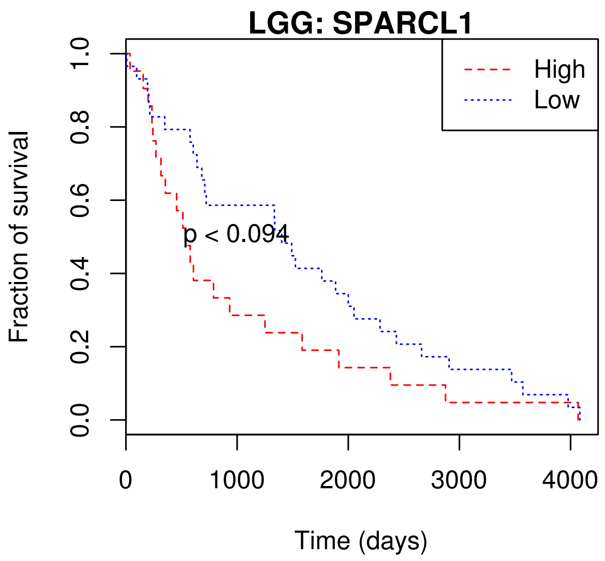
LGG: SPARCL1
High
Low
p < 0.094
Fraction of survival
Time (days)
0.0
0.2
0.4
0.6
0.8
1.0
0
1000
2000
3000
4000

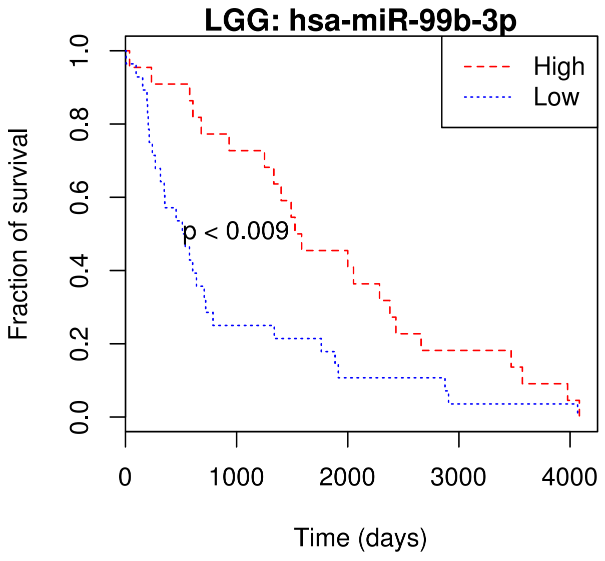
LGG: hsa-miR-99b-3p
High
Low
p < 0.009
Fraction of survival
Time (days)
0.0
0.2
0.4
0.6
0.8
1.0
0
1000
2000
3000
4000

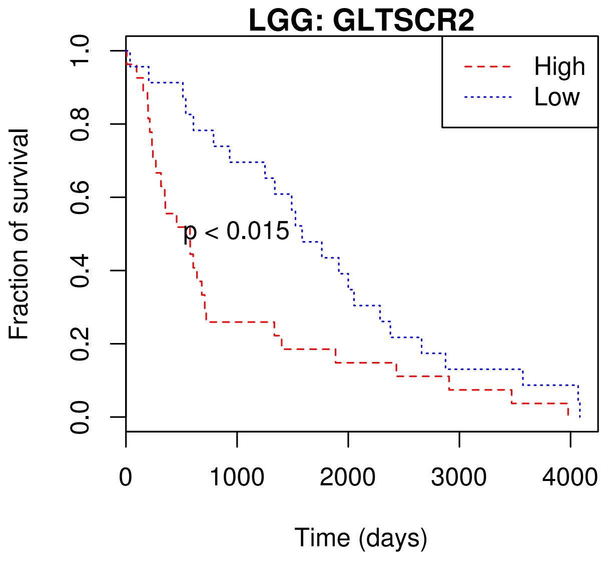
LGG: GLTSCR2
High
Low
p < 0.015
Fraction of survival
Time (days)
0.0
0.2
0.4
0.6
0.8
1.0
0
1000
2000
3000
4000

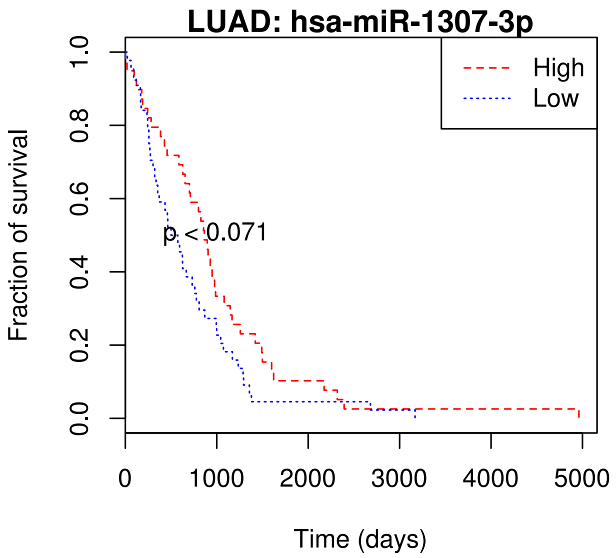
LUAD: hsa-miR-1307-3p
High
Low
p < 0.071
Fraction of survival
Time (days)

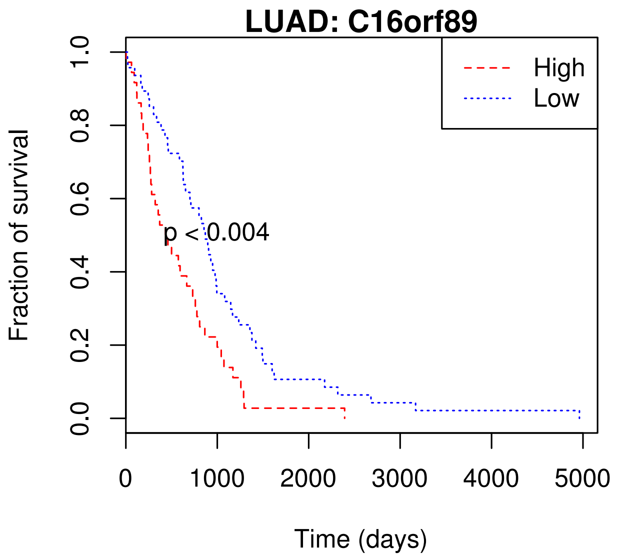
LUAD: C16orf89
High
Low
p < 0.004
Fraction of survival
Time (days)

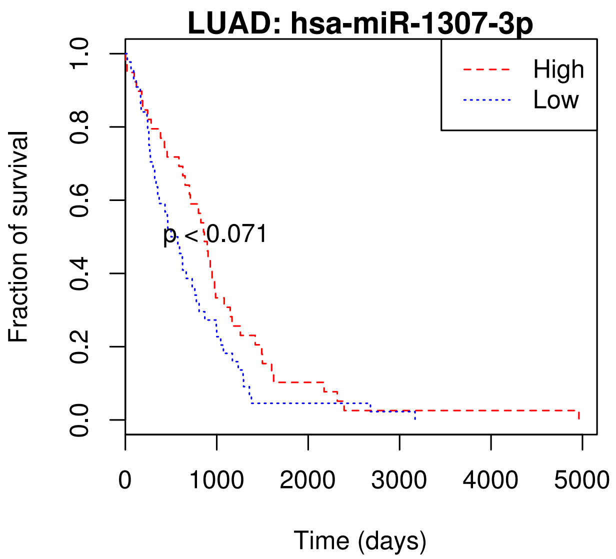
LUAD: hsa-miR-1307-3p
High
Low
p < 0.071
Fraction of survival
Time (days)

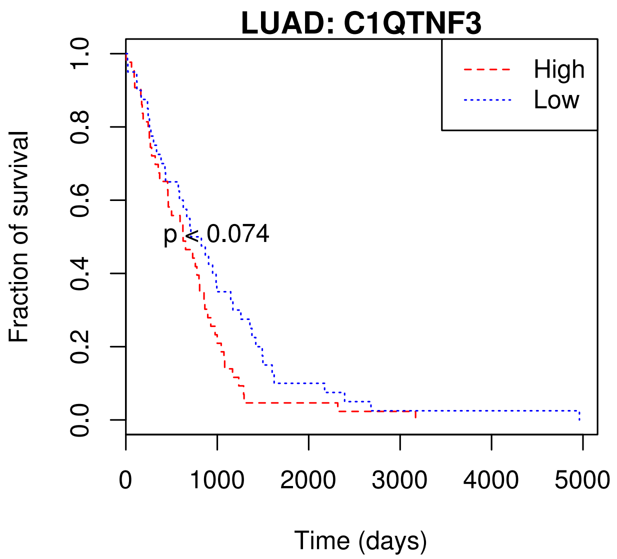
LUAD: C1QTNF3
High
Low
p < 0.074
Fraction of survival
Time (days)

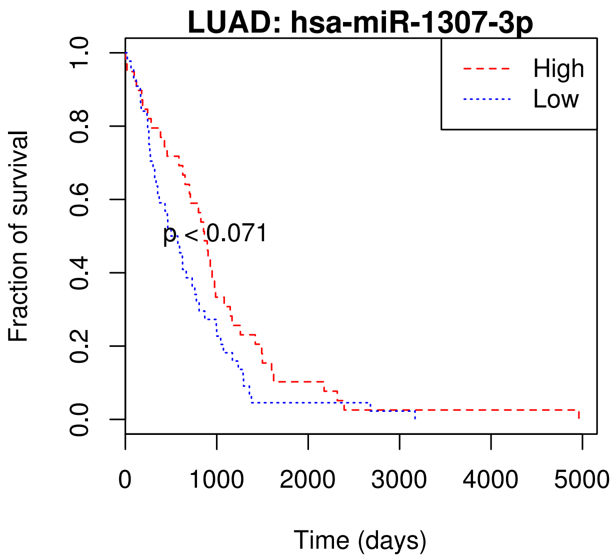
LUAD: hsa-miR-1307-3p
High
Low
p < 0.071
Fraction of survival
Time (days)

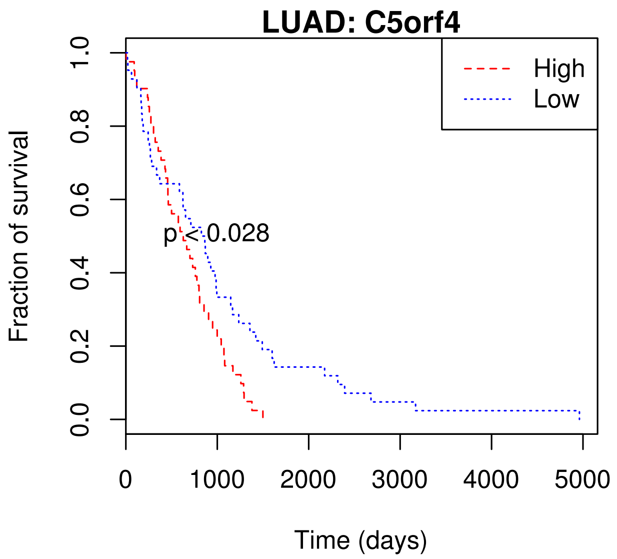
LUAD: C5orf4
High
Low
p < 0.028
Fraction of survival
Time (days)

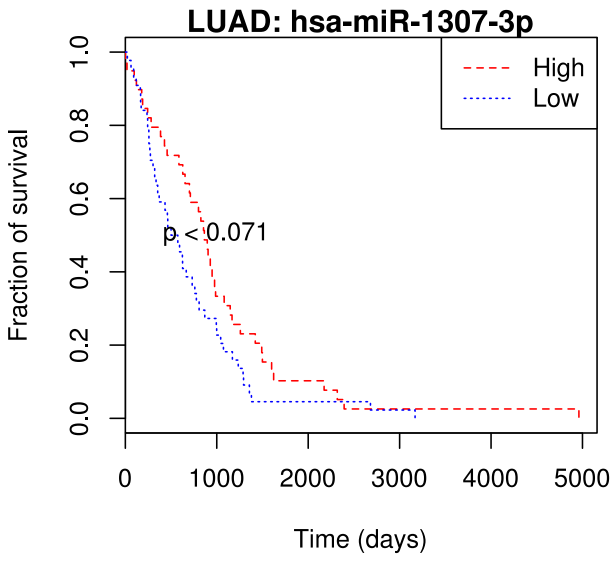
LUAD: hsa-miR-1307-3p
High
Low
p < 0.071
Fraction of survival
0.0
0.2
0.4
0.6
0.8
1.0
0
1000
2000
3000
4000
5000
Time (days)

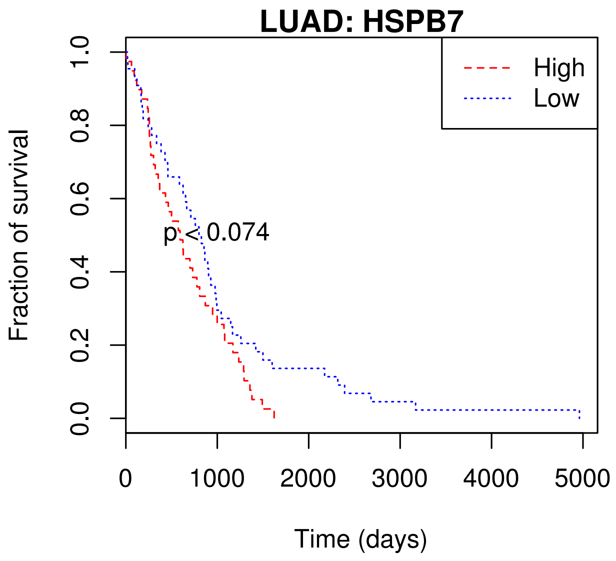
LUAD: HSPB7
High
Low
p < 0.074
Fraction of survival
0.0
0.2
0.4
0.6
0.8
1.0
0
1000
2000
3000
4000
5000
Time (days)

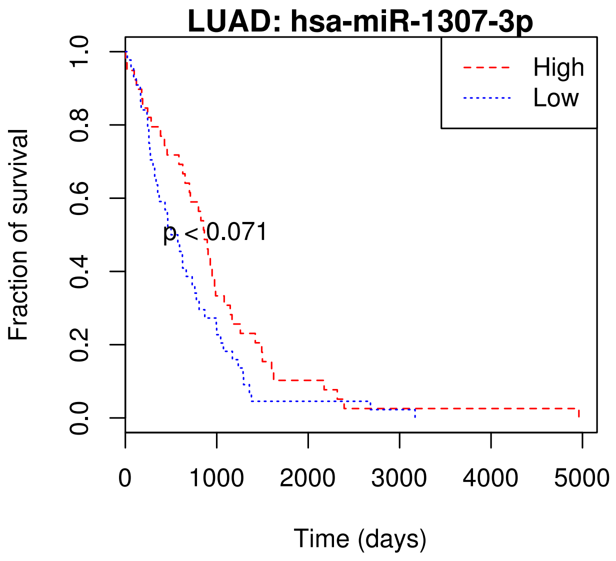
LUAD: hsa-miR-1307-3p
High
Low
p < 0.071
Fraction of survival
0.0
0.2
0.4
0.6
0.8
1.0
0
1000
2000
3000
4000
5000
Time (days)

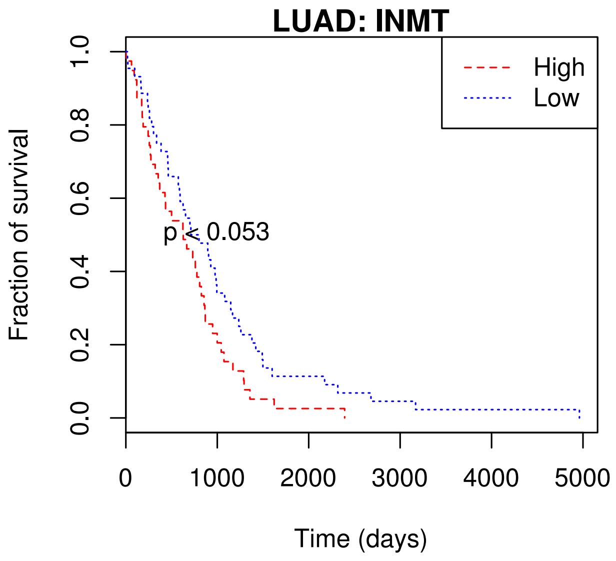
LUAD: INMT
High
Low
p < 0.053
Fraction of survival
0.0
0.2
0.4
0.6
0.8
1.0
0
1000
2000
3000
4000
5000
Time (days)

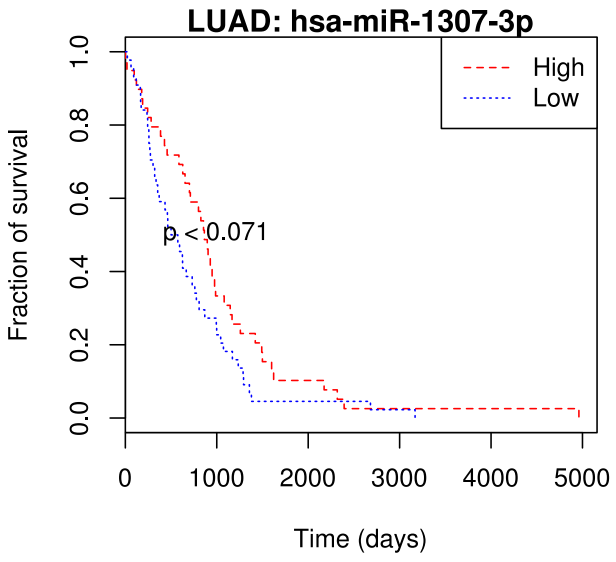
LUAD: hsa-miR-1307-3p
High
Low
p < 0.071
Fraction of survival
0.0
0.2
0.4
0.6
0.8
1.0
0
1000
2000
3000
4000
5000
Time (days)

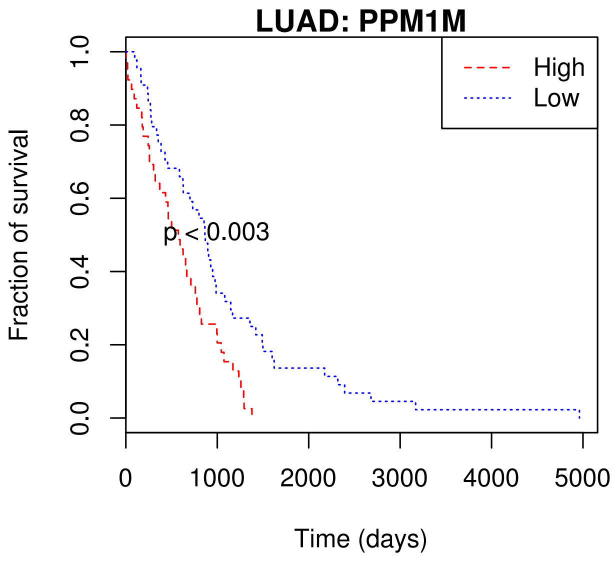
LUAD: PPM1M
High
Low
p < 0.003
Fraction of survival
0.0
0.2
0.4
0.6
0.8
1.0
0
1000
2000
3000
4000
5000
Time (days)

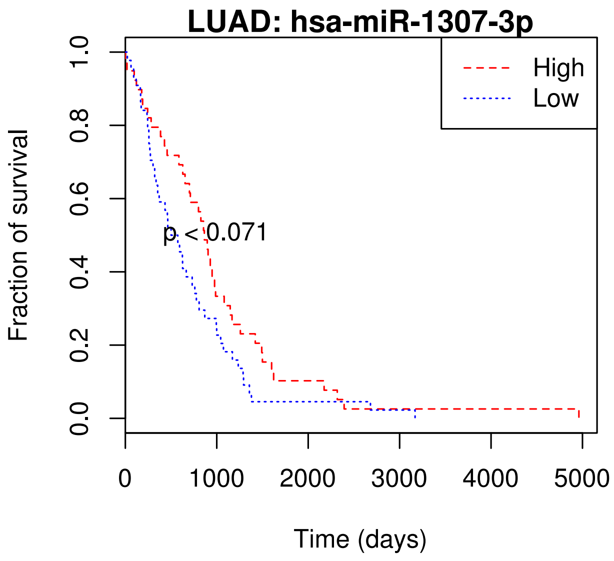
LUAD: hsa-miR-1307-3p
High
Low
p < 0.071
Fraction of survival
0.0
0.2
0.4
0.6
0.8
1.0
0
1000
2000
3000
4000
5000
Time (days)

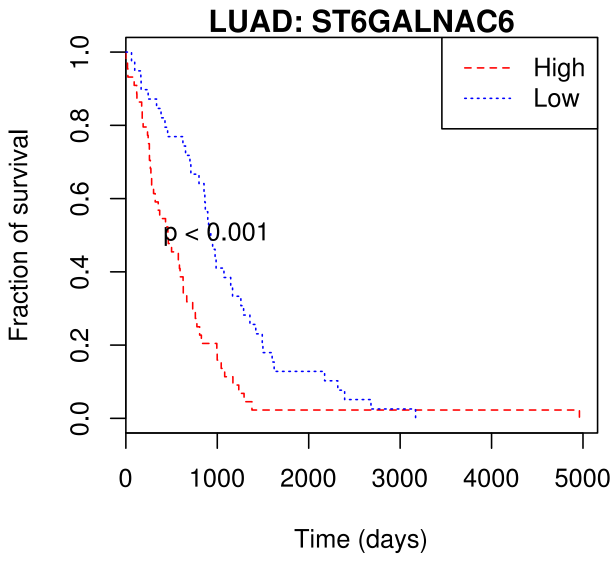
LUAD: ST6GALNAC6
High
Low
p < 0.001
Fraction of survival
0.0
0.2
0.4
0.6
0.8
1.0
0
1000
2000
3000
4000
5000
Time (days)

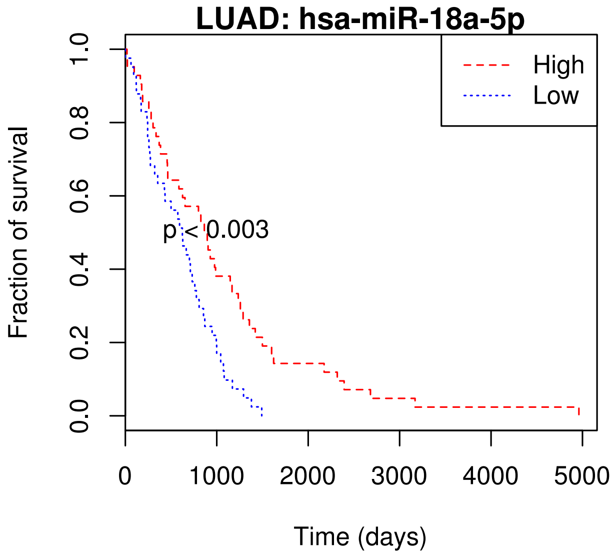
LUAD: hsa-miR-18a-5p
High
Low
p < 0.003
Fraction of survival
0.0
0.2
0.4
0.6
0.8
1.0
0
1000
2000
3000
4000
5000
Time (days)

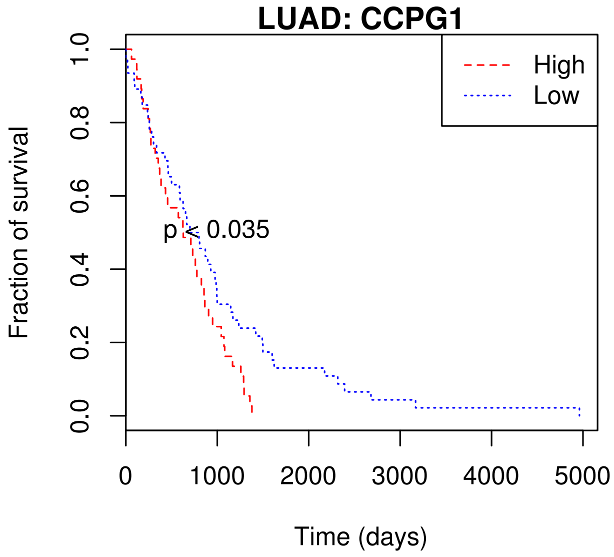
LUAD: CCPG1
High
Low
p < 0.035
Fraction of survival
0.0
0.2
0.4
0.6
0.8
1.0
0
1000
2000
3000
4000
5000
Time (days)

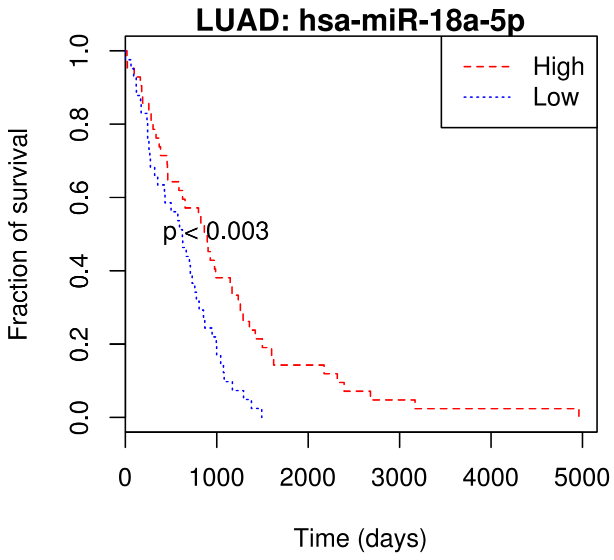
LUAD: hsa-miR-18a-5p
High
Low
p < 0.003
Fraction of survival
0.0
0.2
0.4
0.6
0.8
1.0
0
1000
2000
3000
4000
5000
Time (days)

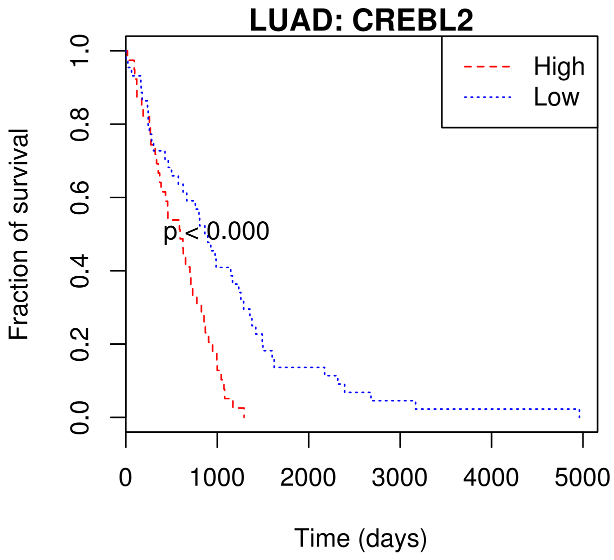
LUAD: CREBL2
High
Low
p < 0.000
Fraction of survival
0.0
0.2
0.4
0.6
0.8
1.0
0
1000
2000
3000
4000
5000
Time (days)

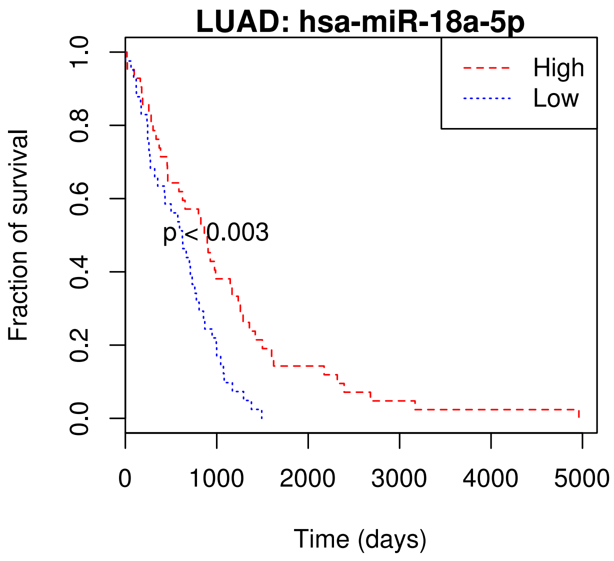
LUAD: hsa-miR-18a-5p
High
Low
p < 0.003
Fraction of survival
1.0
0.8
0.6
0.4
0.2
0.0
0
1000
2000
3000
4000
5000
Time (days)

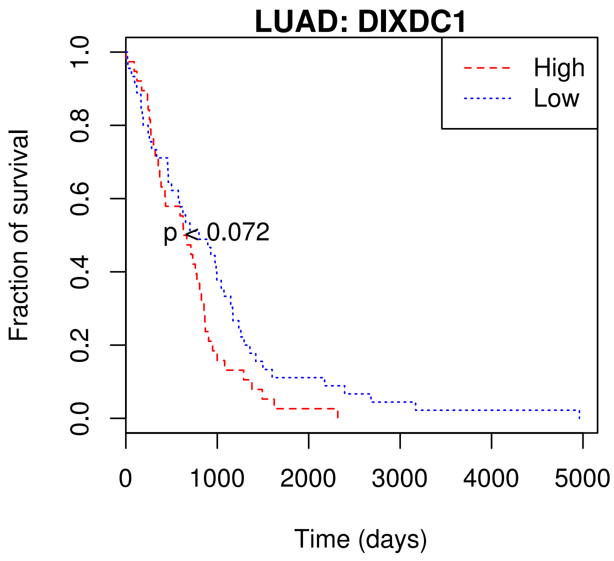
LUAD: DIXDC1
High
Low
p < 0.072
Fraction of survival
1.0
0.8
0.6
0.4
0.2
0.0
0
1000
2000
3000
4000
5000
Time (days)

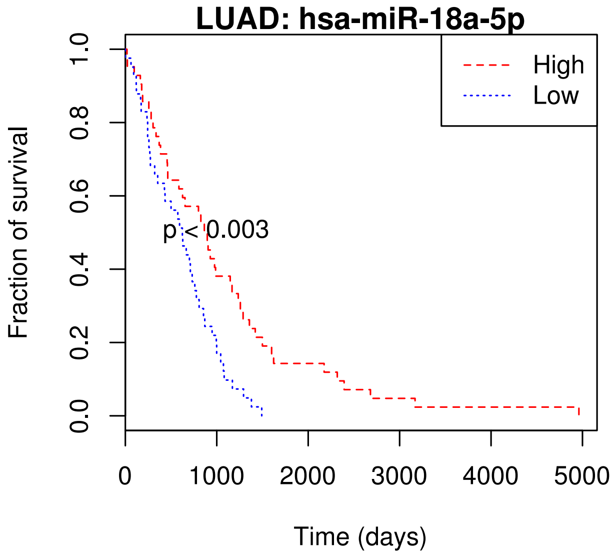
LUAD: hsa-miR-18a-5p
High
Low
p < 0.003
Fraction of survival
1.0
0.8
0.6
0.4
0.2
0.0
0
1000
2000
3000
4000
5000
Time (days)

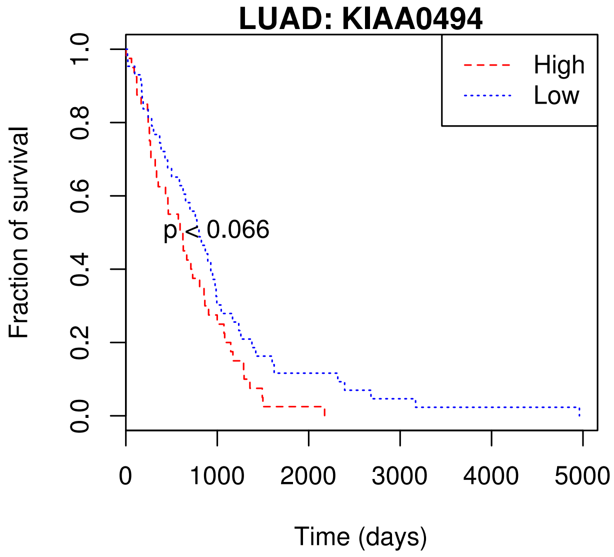
LUAD: KIAA0494
High
Low
p < 0.066
Fraction of survival
1.0
0.8
0.6
0.4
0.2
0.0
0
1000
2000
3000
4000
5000
Time (days)

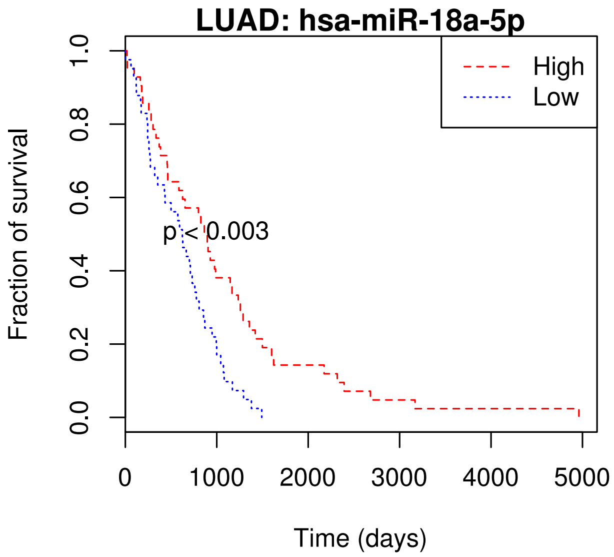
LUAD: hsa-miR-18a-5p
High
Low
p < 0.003
Fraction of survival
1.0
0.8
0.6
0.4
0.2
0.0
0
1000
2000
3000
4000
5000
Time (days)

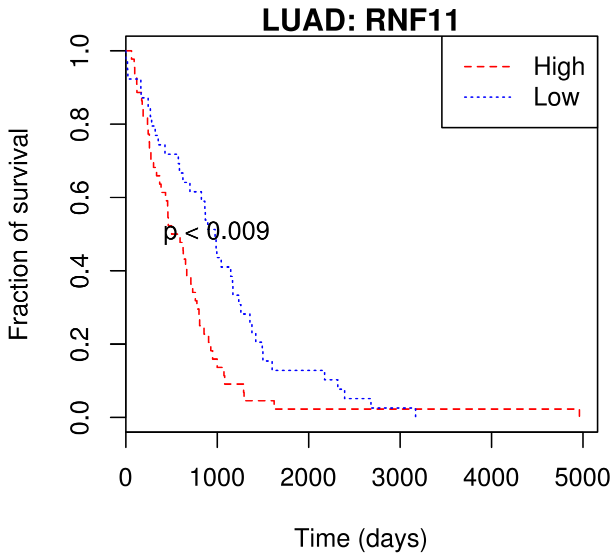
LUAD: RNF11
High
Low
p < 0.009
Fraction of survival
1.0
0.8
0.6
0.4
0.2
0.0
0
1000
2000
3000
4000
5000
Time (days)

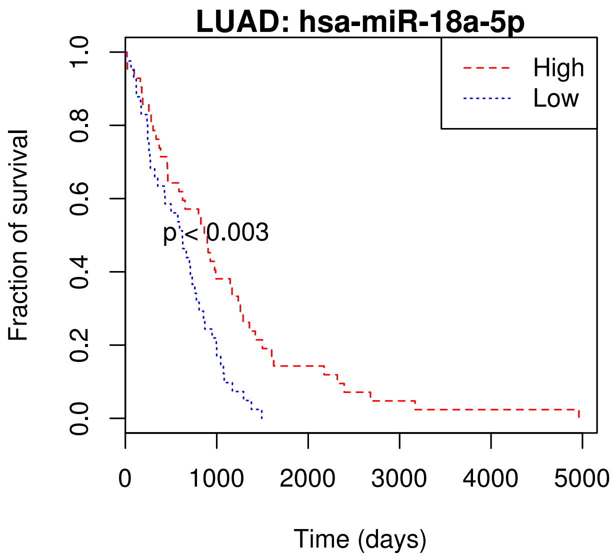
LUAD: hsa-miR-18a-5p
High
Low
p < 0.003
Fraction of survival
0.0 0.2 0.4 0.6 0.8 1.0
0 1000 2000 3000 4000 5000
Time (days)

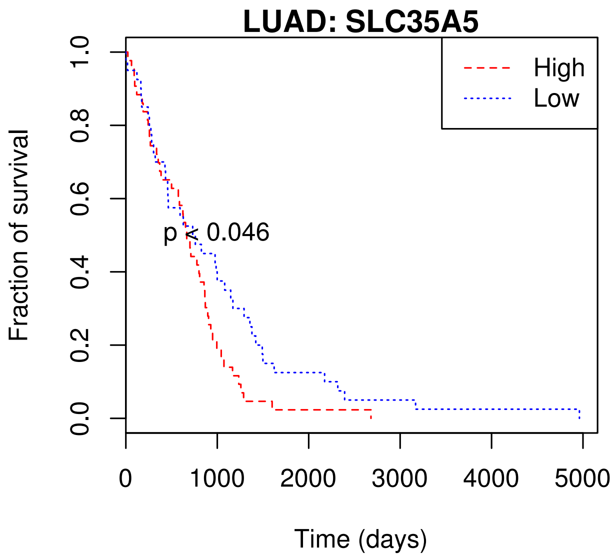
LUAD: SLC35A5
High
Low
p < 0.046
Fraction of survival
0.0 0.2 0.4 0.6 0.8 1.0
0 1000 2000 3000 4000 5000
Time (days)

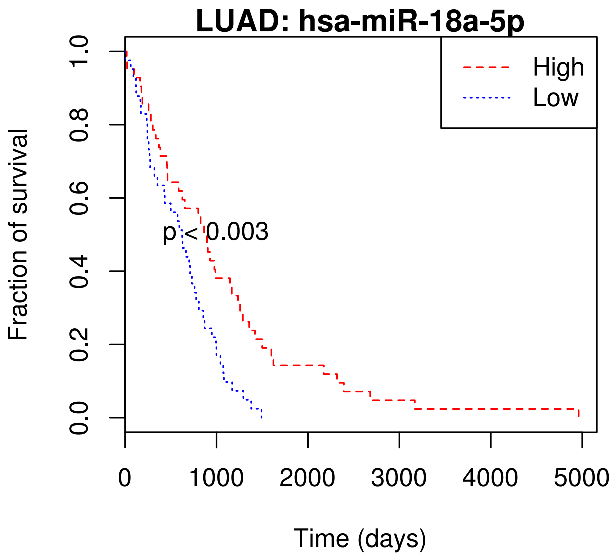
LUAD: hsa-miR-18a-5p
High
Low
p < 0.003
Fraction of survival
0.0 0.2 0.4 0.6 0.8 1.0
0 1000 2000 3000 4000 5000
Time (days)

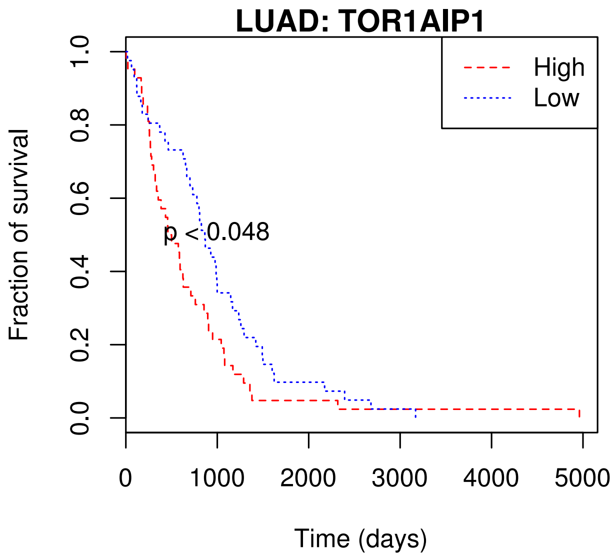
LUAD: TOR1AIP1
High
Low
p < 0.048
Fraction of survival
0.0 0.2 0.4 0.6 0.8 1.0
0 1000 2000 3000 4000 5000
Time (days)

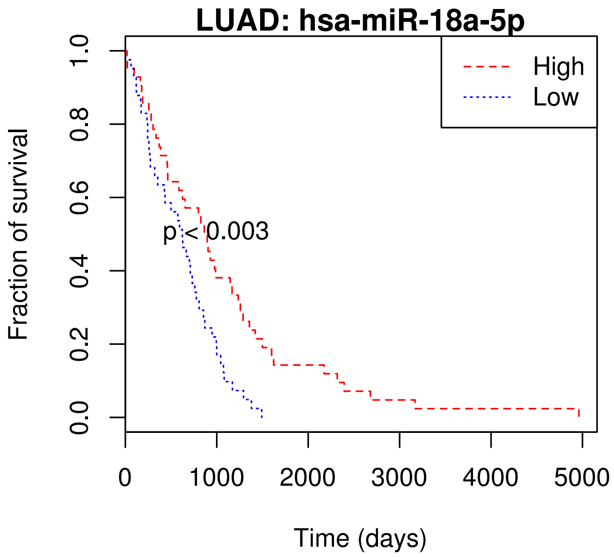
LUAD: hsa-miR-18a-5p
High
Low
p < 0.003
Fraction of survival
0.0 0.2 0.4 0.6 0.8 1.0
0 1000 2000 3000 4000 5000
Time (days)

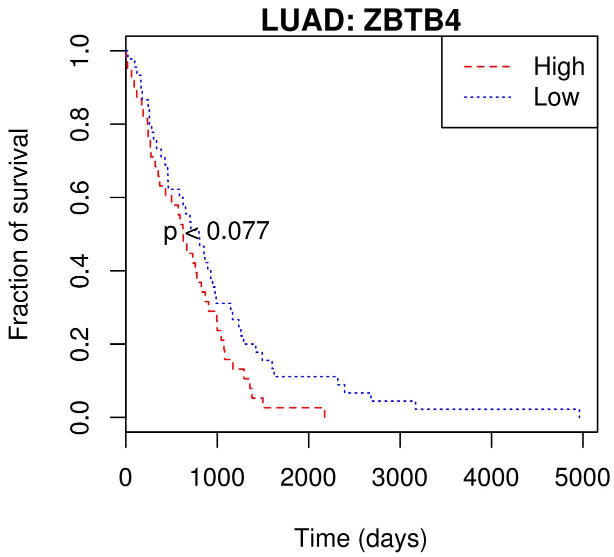
LUAD: ZBTB4
High
Low
p < 0.077
Fraction of survival
0.0 0.2 0.4 0.6 0.8 1.0
0 1000 2000 3000 4000 5000
Time (days)

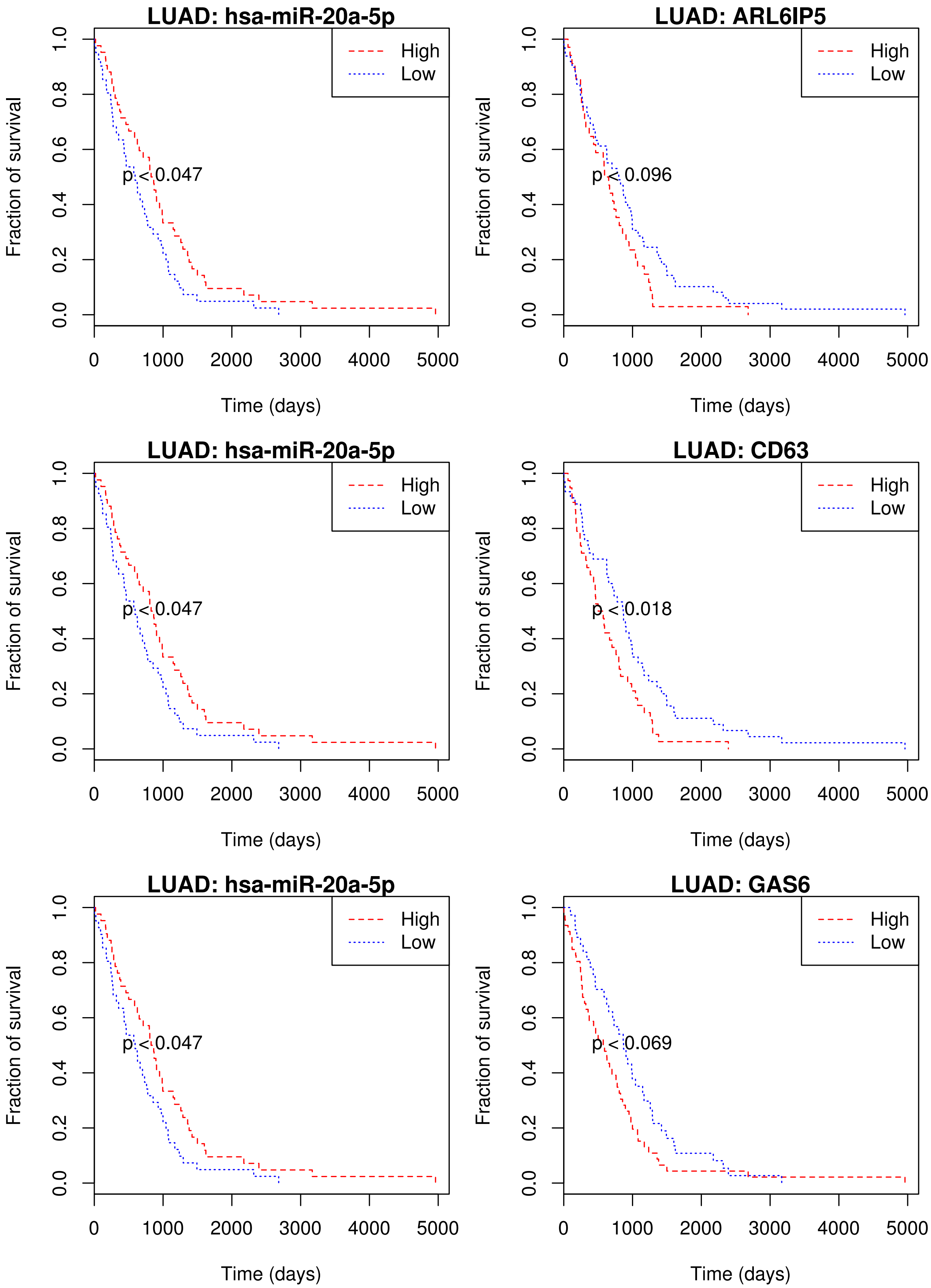
LUAD: hsa-miR-20a-5p
Fraction of survival
Time (days)
High
Low
p < 0.047
LUAD: ARL6IP5
Fraction of survival
Time (days)
High
Low
p < 0.096
LUAD: hsa-miR-20a-5p
Fraction of survival
Time (days)
High
Low
p < 0.047
LUAD: CD63
Fraction of survival
Time (days)
High
Low
p < 0.018
LUAD: hsa-miR-20a-5p
Fraction of survival
Time (days)
High
Low
p < 0.047
LUAD: GAS6
Fraction of survival
Time (days)
High
Low
p < 0.069

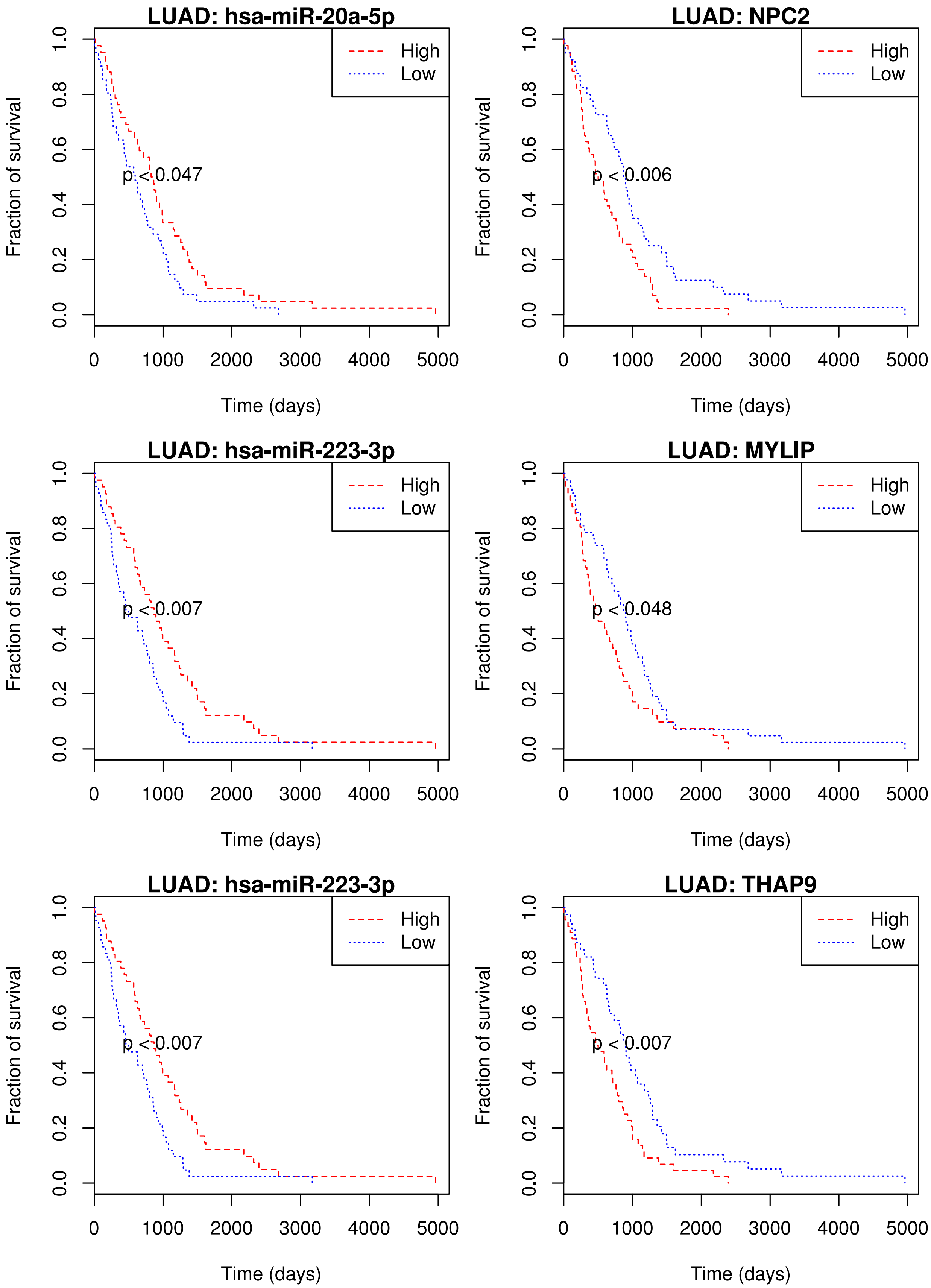
LUAD: hsa-miR-20a-5p
High
Low
p < 0.047
Fraction of survival
Time (days)
LUAD: NPC2
High
Low
p < 0.006
Fraction of survival
Time (days)
LUAD: hsa-miR-223-3p
High
Low
p < 0.007
Fraction of survival
Time (days)
LUAD: MYLIP
High
Low
p < 0.048
Fraction of survival
Time (days)
LUAD: hsa-miR-223-3p
High
Low
p < 0.007
Fraction of survival
Time (days)
LUAD: THAP9
High
Low
p < 0.007
Fraction of survival
Time (days)

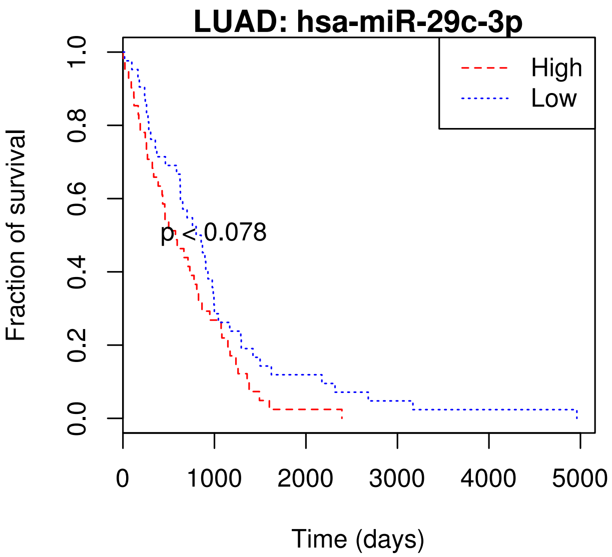
LUAD: hsa-miR-29c-3p
High
Low
p < 0.078
Fraction of survival
Time (days)

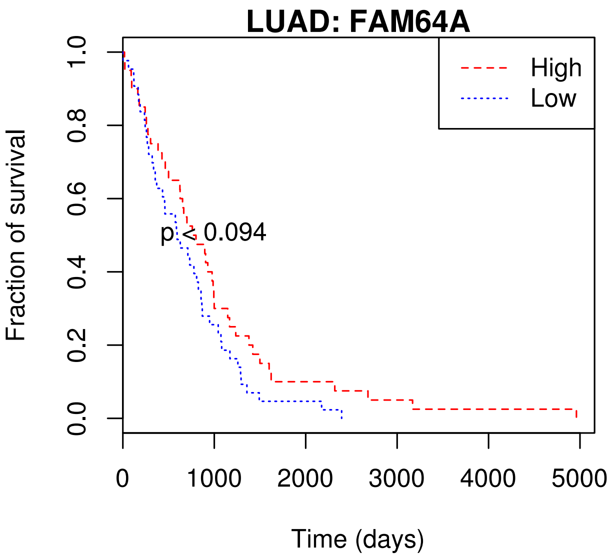
LUAD: FAM64A
High
Low
p < 0.094
Fraction of survival
Time (days)

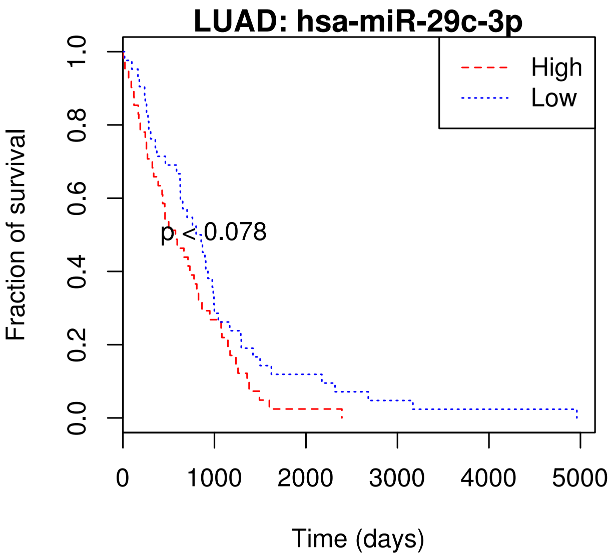
LUAD: hsa-miR-29c-3p
High
Low
p < 0.078
Fraction of survival
Time (days)

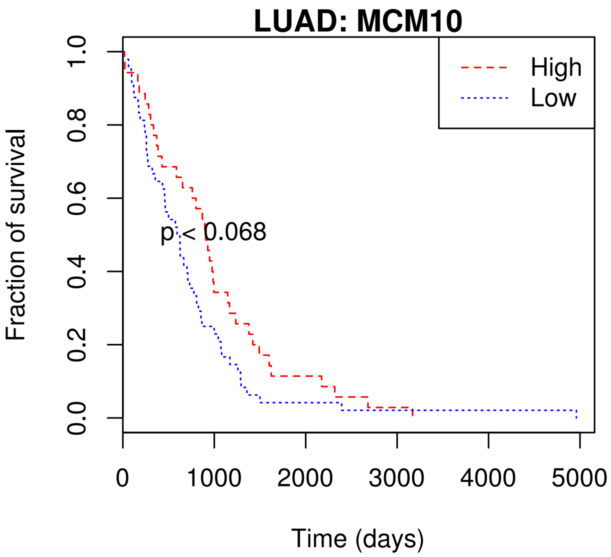
LUAD: MCM10
High
Low
p < 0.068
Fraction of survival
Time (days)

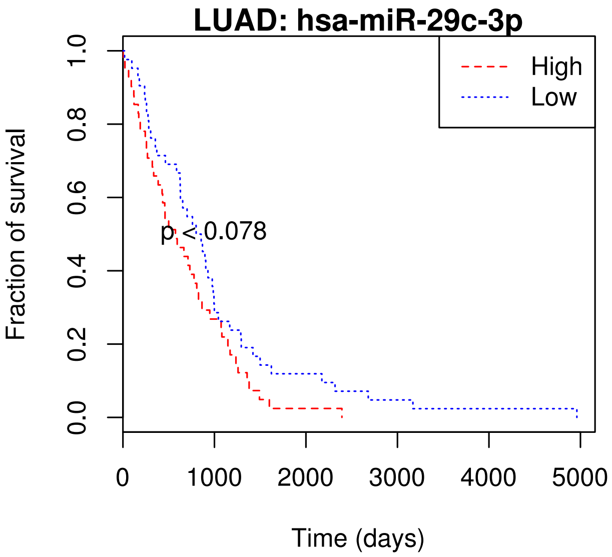
LUAD: hsa-miR-29c-3p
High
Low
p < 0.078
Fraction of survival
Time (days)

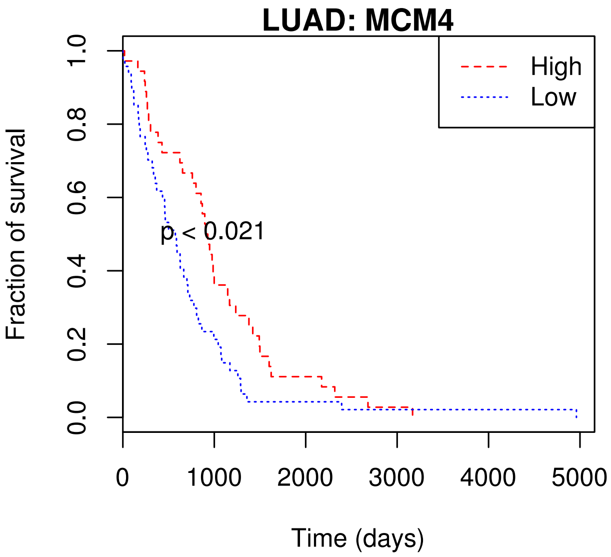
LUAD: MCM4
High
Low
p < 0.021
Fraction of survival
Time (days)

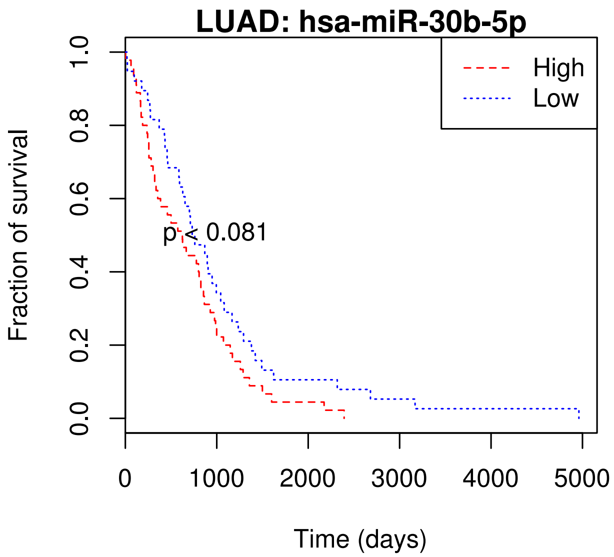
LUAD: hsa-miR-30b-5p
High
Low
p < 0.081
Fraction of survival
Time (days)
0.0
0.2
0.4
0.6
0.8
1.0
0
1000
2000
3000
4000
5000

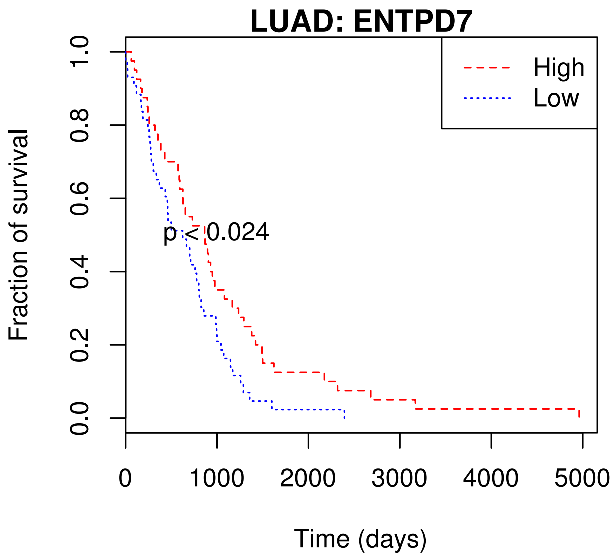
LUAD: ENTPD7
High
Low
p < 0.024
Fraction of survival
Time (days)
0.0
0.2
0.4
0.6
0.8
1.0
0
1000
2000
3000
4000
5000

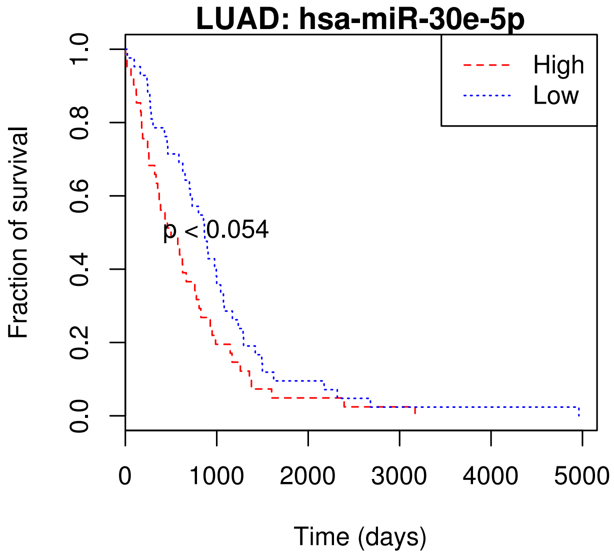
LUAD: hsa-miR-30e-5p
High
Low
p < 0.054
Fraction of survival
Time (days)
0.0
0.2
0.4
0.6
0.8
1.0
0
1000
2000
3000
4000
5000

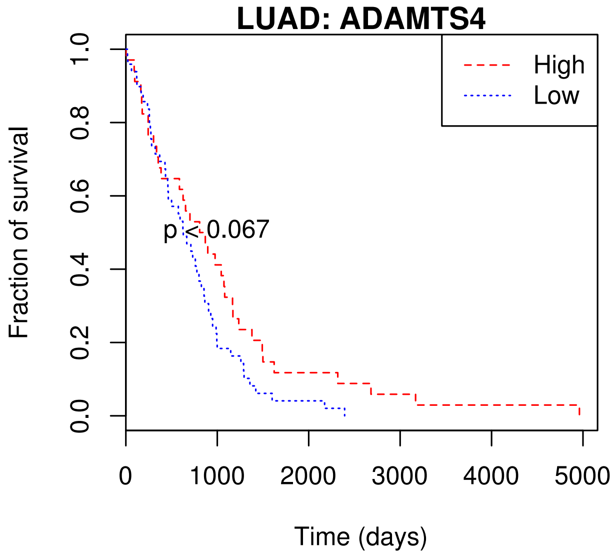
LUAD: ADAMTS4
High
Low
p < 0.067
Fraction of survival
Time (days)
0.0
0.2
0.4
0.6
0.8
1.0
0
1000
2000
3000
4000
5000

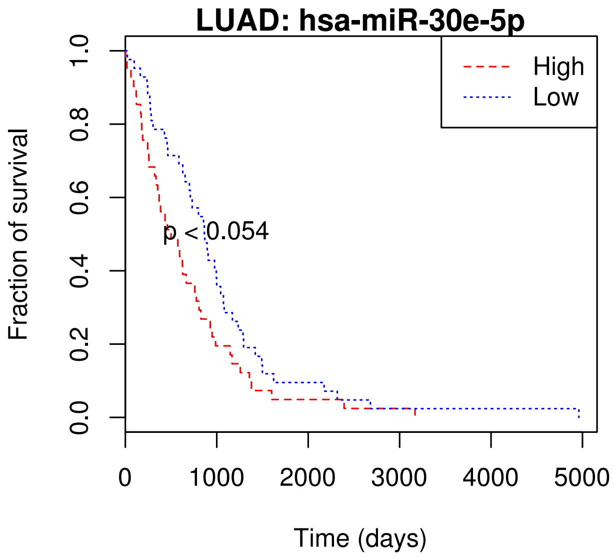
LUAD: hsa-miR-30e-5p
High
Low
p < 0.054
Fraction of survival
Time (days)
0.0
0.2
0.4
0.6
0.8
1.0
0
1000
2000
3000
4000
5000

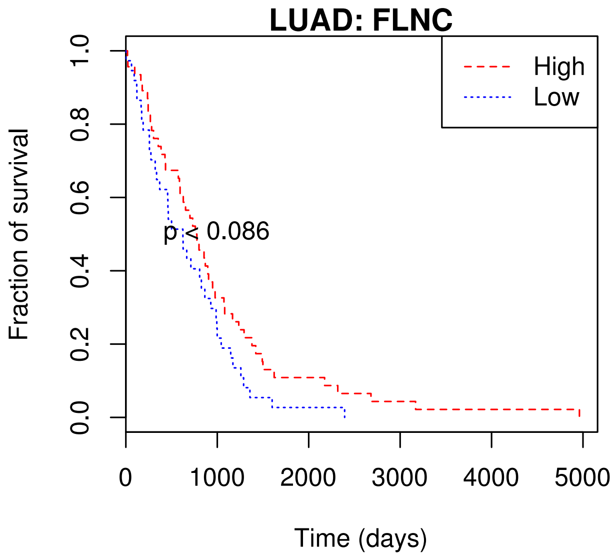
LUAD: FLNC
High
Low
p < 0.086
Fraction of survival
Time (days)
0.0
0.2
0.4
0.6
0.8
1.0
0
1000
2000
3000
4000
5000

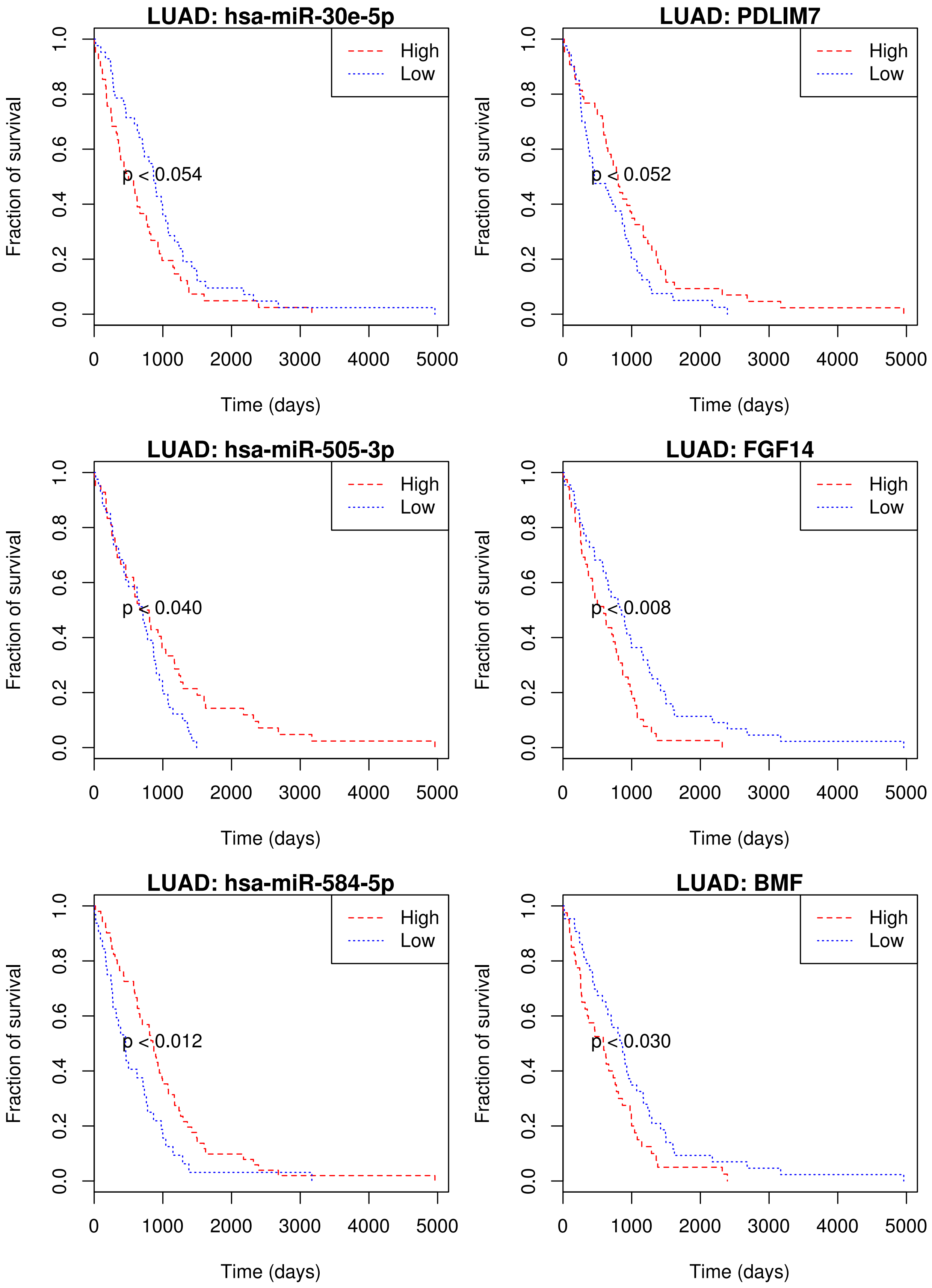
LUAD: hsa-miR-30e-5p
High
Low
p < 0.054
Fraction of survival
Time (days)
0.0 0.2 0.4 0.6 0.8 1.0
0 1000 2000 3000 4000 5000
LUAD: PDLIM7
High
Low
p < 0.052
Fraction of survival
Time (days)
0.0 0.2 0.4 0.6 0.8 1.0
0 1000 2000 3000 4000 5000
LUAD: hsa-miR-505-3p
High
Low
p < 0.040
Fraction of survival
Time (days)
0.0 0.2 0.4 0.6 0.8 1.0
0 1000 2000 3000 4000 5000
LUAD: FGF14
High
Low
p < 0.008
Fraction of survival
Time (days)
0.0 0.2 0.4 0.6 0.8 1.0
0 1000 2000 3000 4000 5000
LUAD: hsa-miR-584-5p
High
Low
p < 0.012
Fraction of survival
Time (days)
0.0 0.2 0.4 0.6 0.8 1.0
0 1000 2000 3000 4000 5000
LUAD: BMF
High
Low
p < 0.030
Fraction of survival
Time (days)
0.0 0.2 0.4 0.6 0.8 1.0
0 1000 2000 3000 4000 5000

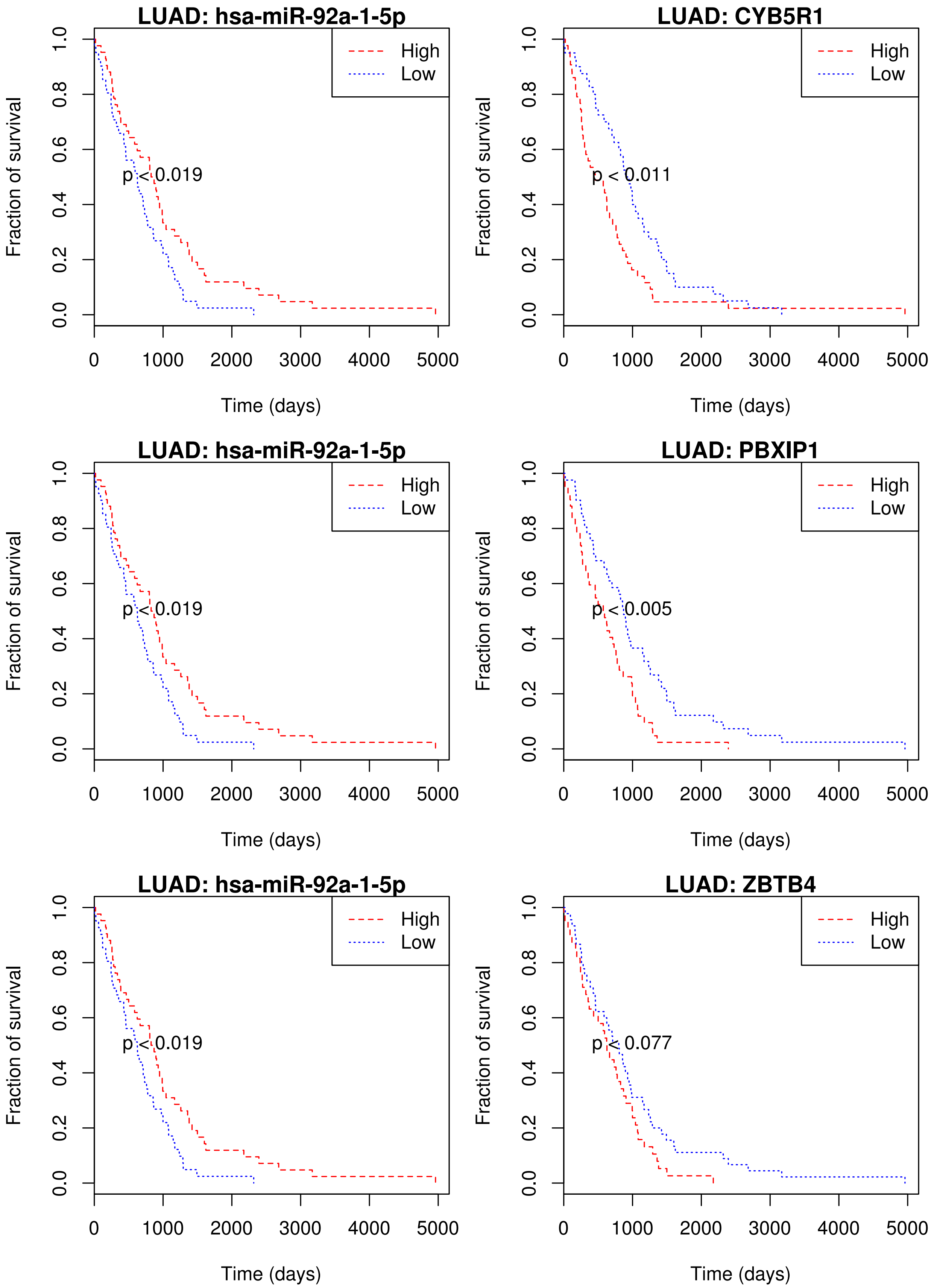
LUAD: hsa-miR-92a-1-5p
p < 0.019
LUAD: CYB5R1
p < 0.011
LUAD: hsa-miR-92a-1-5p
p < 0.019
LUAD: PBXIP1
p < 0.005
LUAD: hsa-miR-92a-1-5p
p < 0.019
LUAD: ZBTB4
p < 0.077
High
Low
Fraction of survival
Time (days)

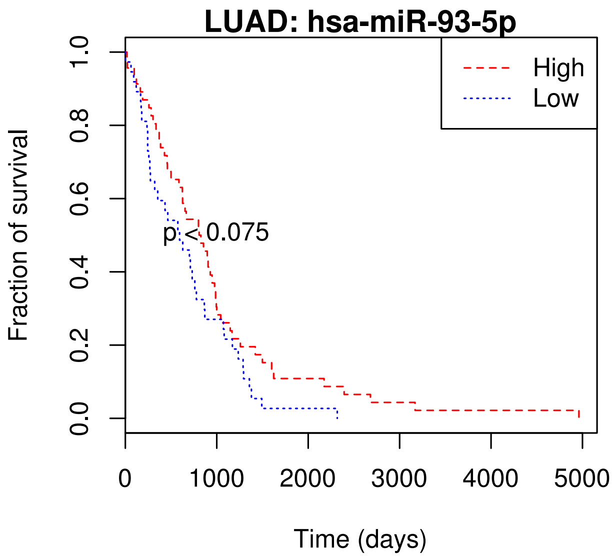
LUAD: hsa-miR-93-5p
High
Low
p < 0.075
Fraction of survival
Time (days)
0.0 0.2 0.4 0.6 0.8 1.0
0 1000 2000 3000 4000 5000

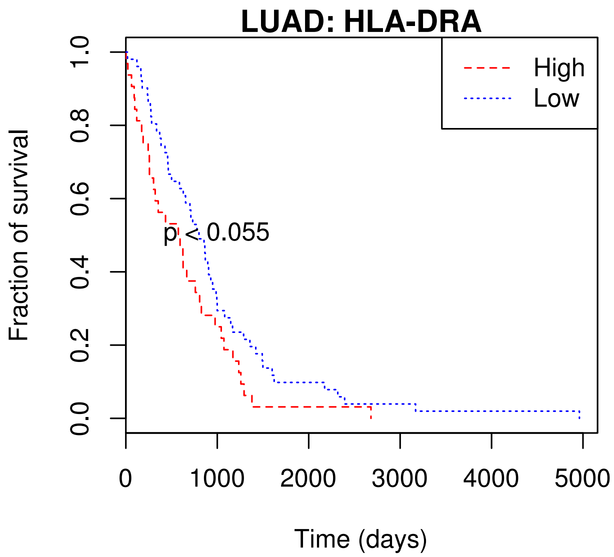
LUAD: HLA-DRA
High
Low
p < 0.055
Fraction of survival
Time (days)
0.0 0.2 0.4 0.6 0.8 1.0
0 1000 2000 3000 4000 5000

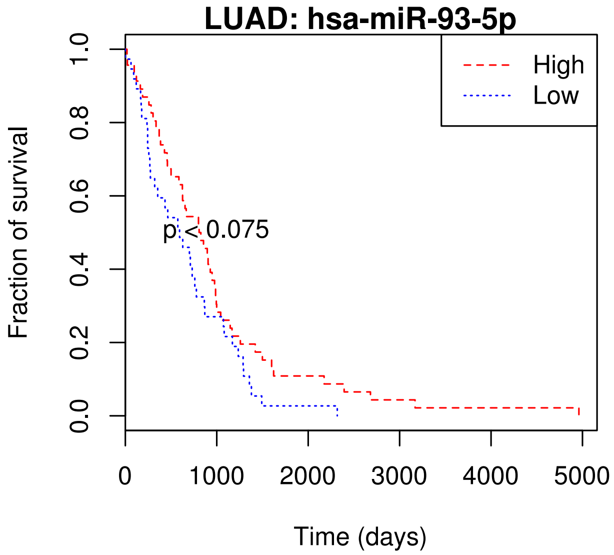
LUAD: hsa-miR-93-5p
High
Low
p < 0.075
Fraction of survival
Time (days)
0.0 0.2 0.4 0.6 0.8 1.0
0 1000 2000 3000 4000 5000

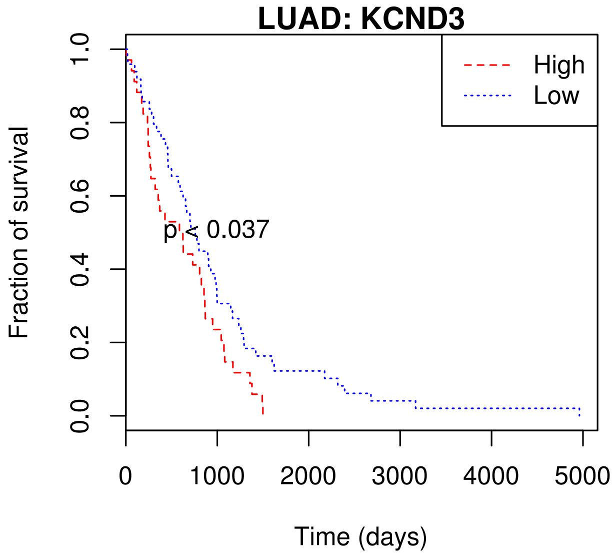
LUAD: KCND3
High
Low
p < 0.037
Fraction of survival
Time (days)
0.0 0.2 0.4 0.6 0.8 1.0
0 1000 2000 3000 4000 5000

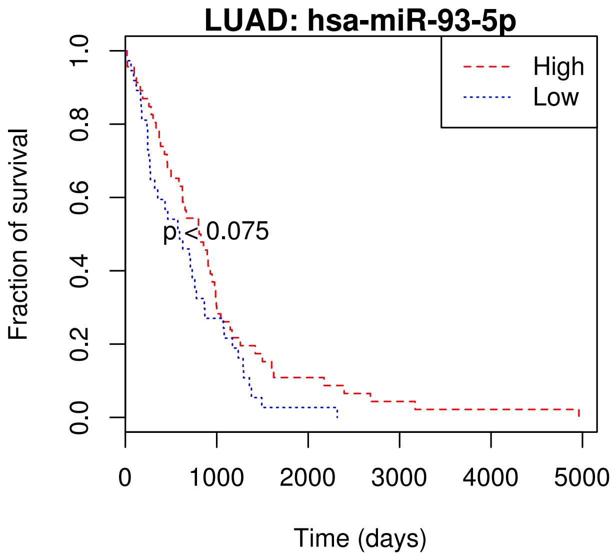
LUAD: hsa-miR-93-5p
High
Low
p < 0.075
Fraction of survival
Time (days)
0.0 0.2 0.4 0.6 0.8 1.0
0 1000 2000 3000 4000 5000

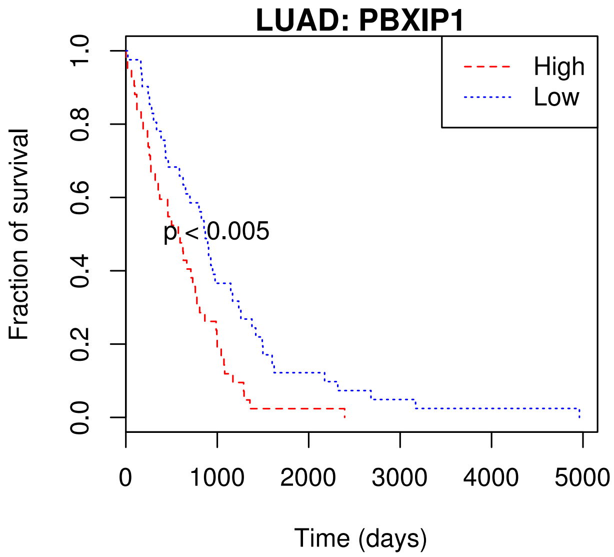
LUAD: PBXIP1
High
Low
p < 0.005
Fraction of survival
Time (days)
0.0 0.2 0.4 0.6 0.8 1.0
0 1000 2000 3000 4000 5000

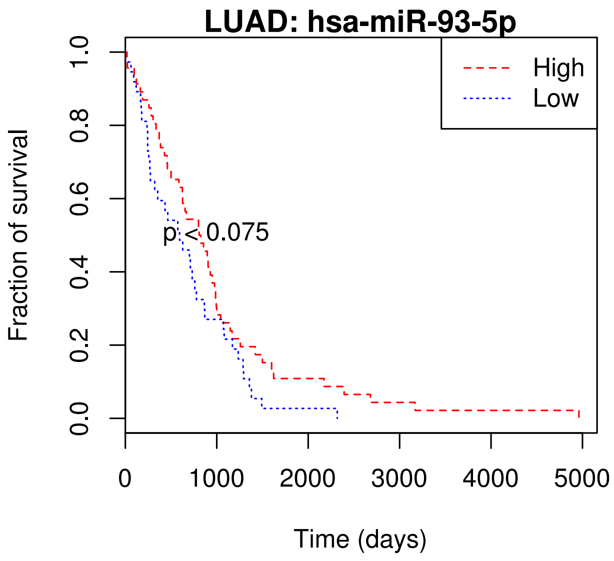
LUAD: hsa-miR-93-5p
High
Low
p < 0.075
Fraction of survival
Time (days)

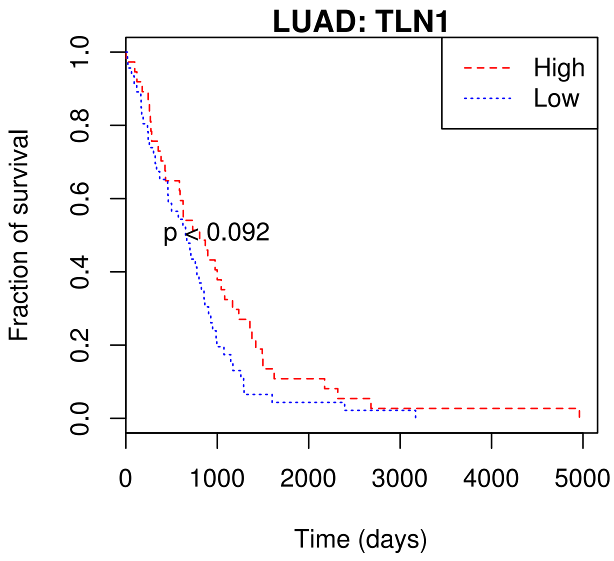
LUAD: TLN1
High
Low
p < 0.092
Fraction of survival
Time (days)

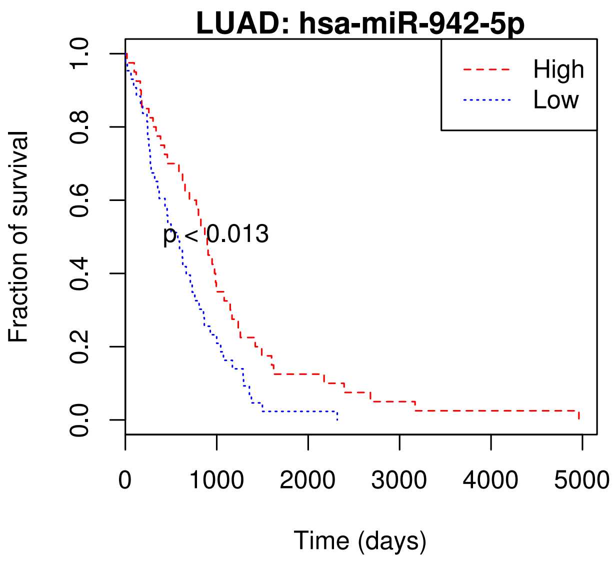
LUAD: hsa-miR-942-5p
High
Low
p < 0.013
Fraction of survival
Time (days)

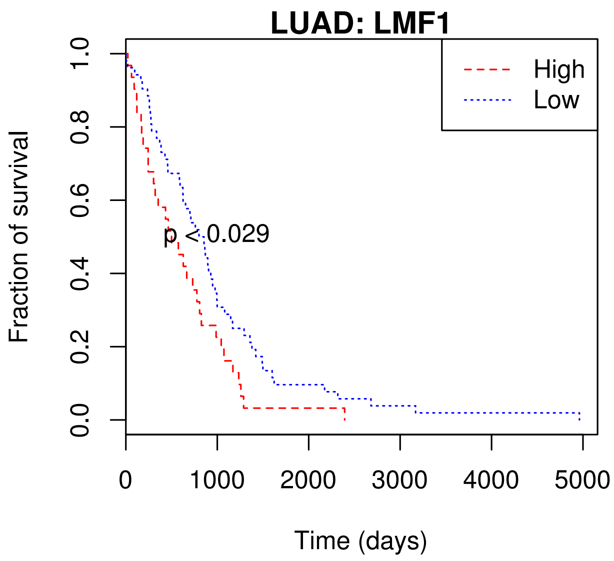
LUAD: LMF1
High
Low
p < 0.029
Fraction of survival
Time (days)

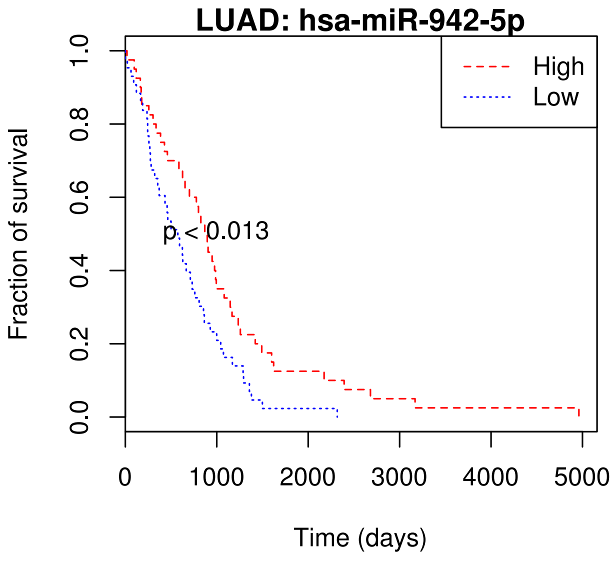
LUAD: hsa-miR-942-5p
High
Low
p < 0.013
Fraction of survival
Time (days)

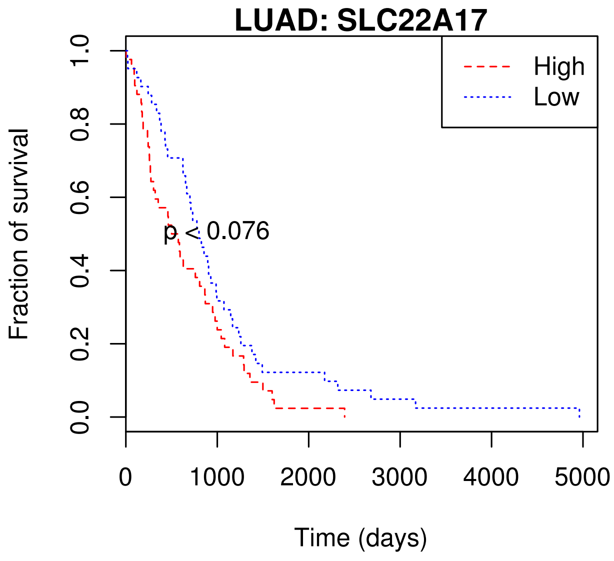
LUAD: SLC22A17
High
Low
p < 0.076
Fraction of survival
Time (days)

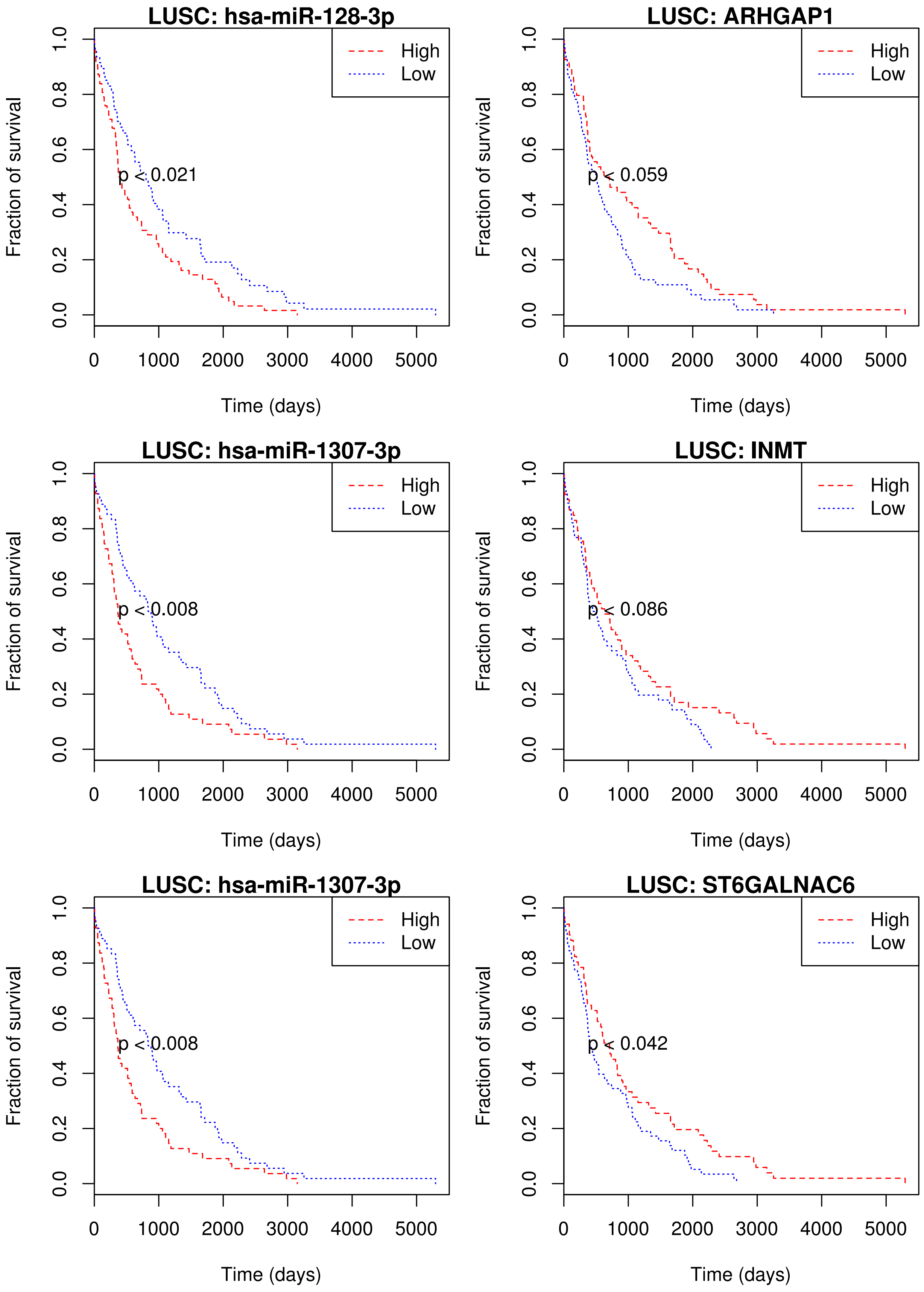
LUSC: hsa-miR-128-3p
p < 0.021
LUSC: ARHGAP1
p < 0.059
LUSC: hsa-miR-1307-3p
p < 0.008
LUSC: INMT
p < 0.086
LUSC: hsa-miR-1307-3p
p < 0.008
LUSC: ST6GALNAC6
p < 0.042
High
Low
Fraction of survival
Time (days)
0.0
0.2
0.4
0.6
0.8
1.0
0
1000
2000
3000
4000
5000

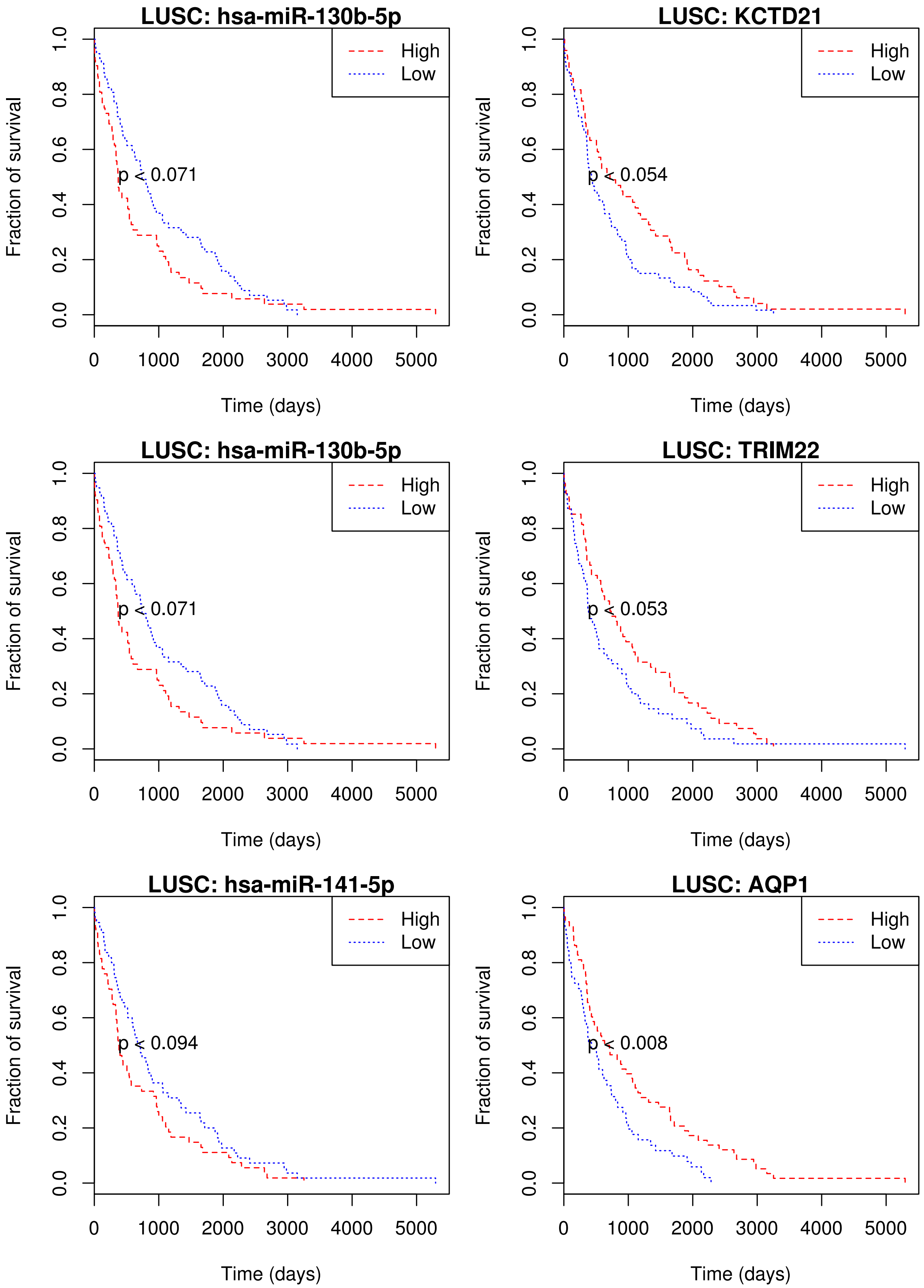
LUSC: hsa-miR-130b-5p
High
Low
p < 0.071
LUSC: KCTD21
High
Low
p < 0.054
LUSC: hsa-miR-130b-5p
High
Low
p < 0.071
LUSC: TRIM22
High
Low
p < 0.053
LUSC: hsa-miR-141-5p
High
Low
p < 0.094
LUSC: AQP1
High
Low
p < 0.008
Fraction of survival
Time (days)

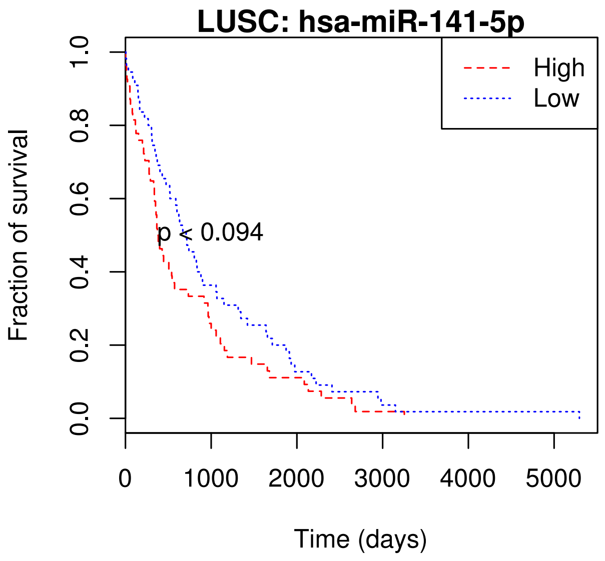
LUSC: hsa-miR-141-5p
High
Low
p < 0.094
Fraction of survival
Time (days)

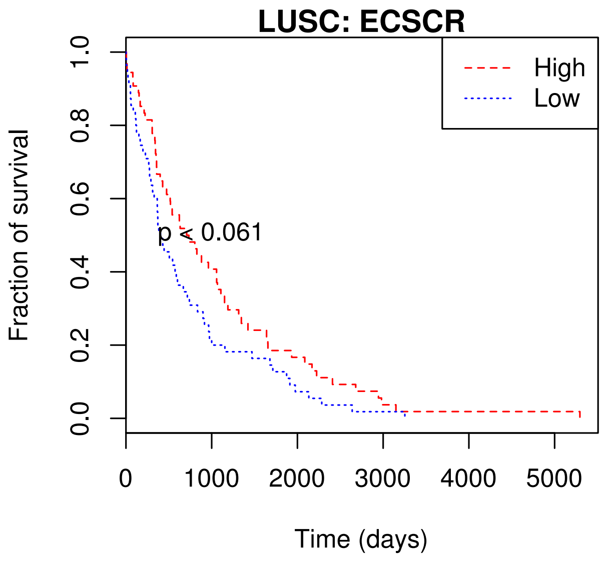
LUSC: ECSCR
High
Low
p < 0.061
Fraction of survival
Time (days)

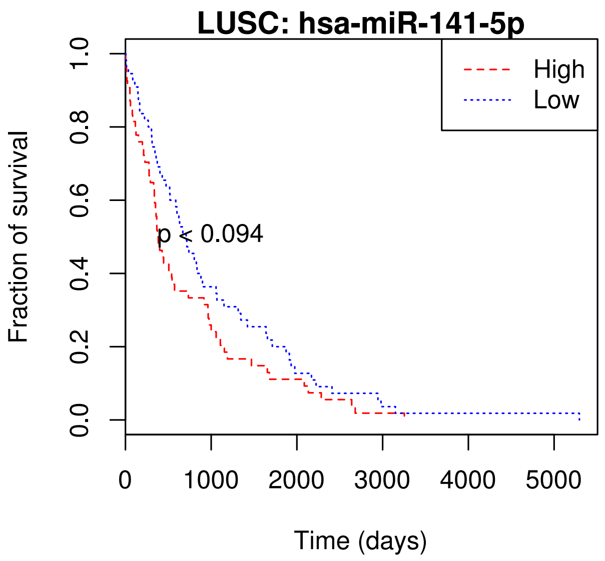
LUSC: hsa-miR-141-5p
High
Low
p < 0.094
Fraction of survival
Time (days)

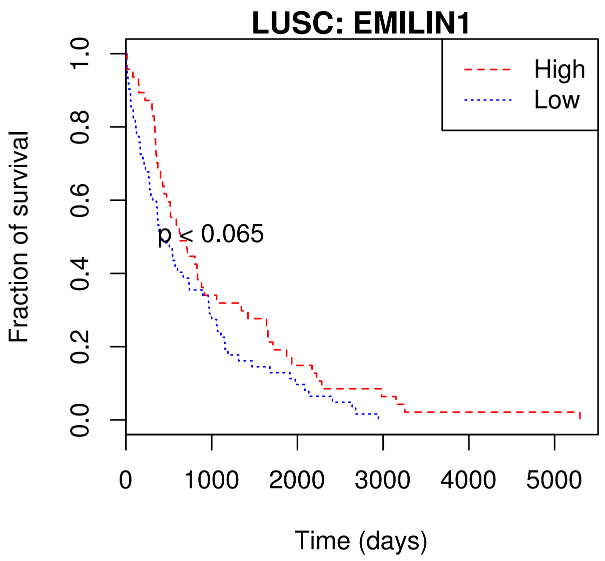
LUSC: EMILIN1
High
Low
p < 0.065
Fraction of survival
Time (days)

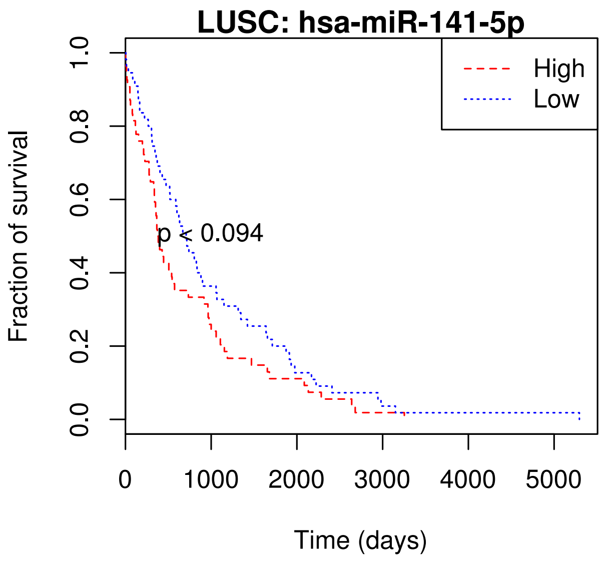
LUSC: hsa-miR-141-5p
High
Low
p < 0.094
Fraction of survival
Time (days)

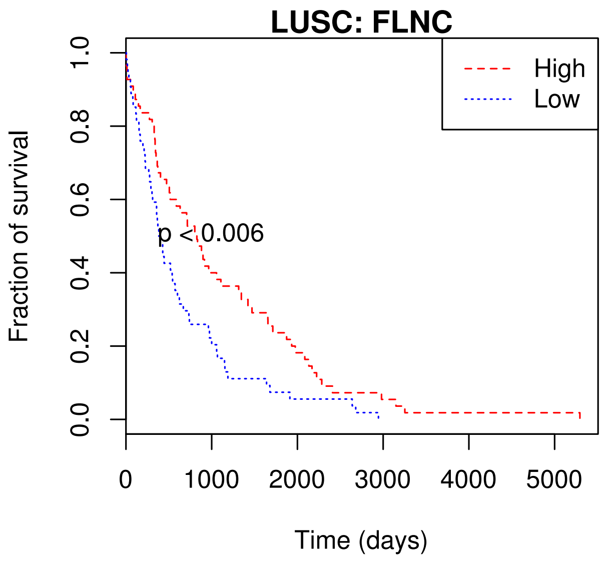
LUSC: FLNC
High
Low
p < 0.006
Fraction of survival
Time (days)

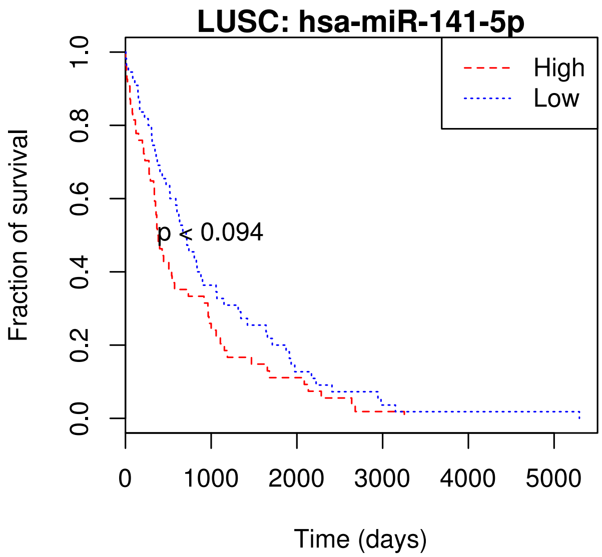
LUSC: hsa-miR-141-5p
High
Low
p < 0.094
Fraction of survival
Time (days)

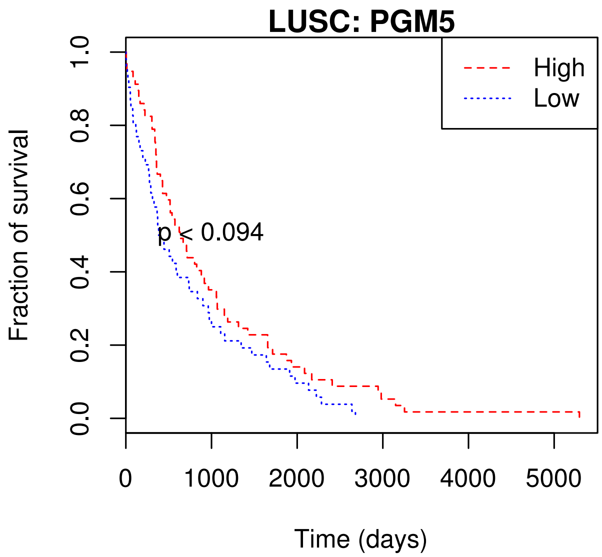
LUSC: PGM5
High
Low
p < 0.094
Fraction of survival
Time (days)

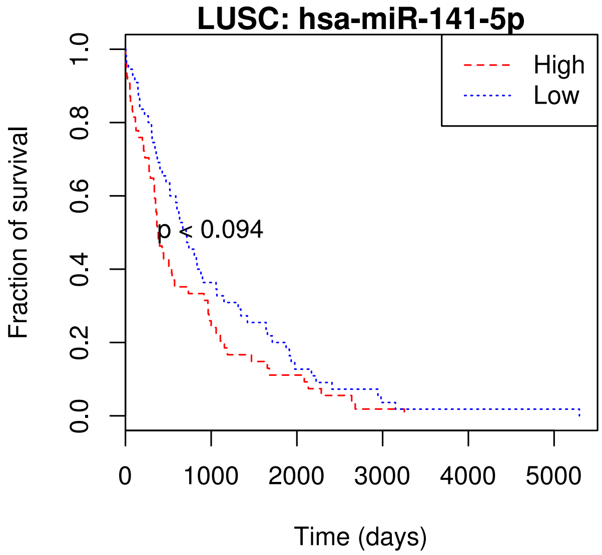
LUSC: hsa-miR-141-5p
High
Low
p < 0.094
Fraction of survival
Time (days)

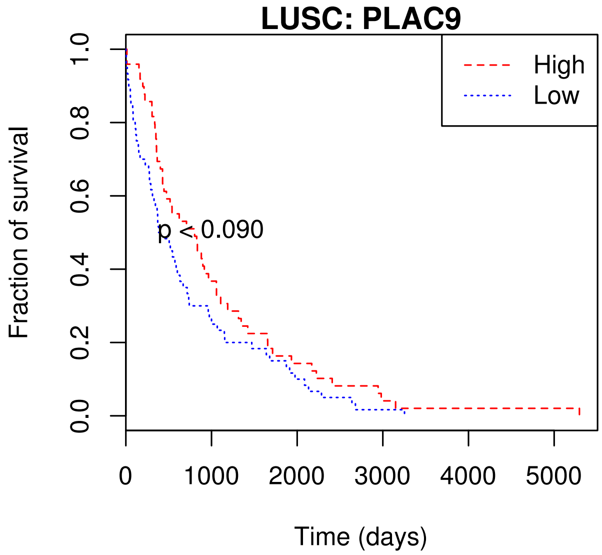
LUSC: PLAC9
High
Low
p < 0.090
Fraction of survival
Time (days)

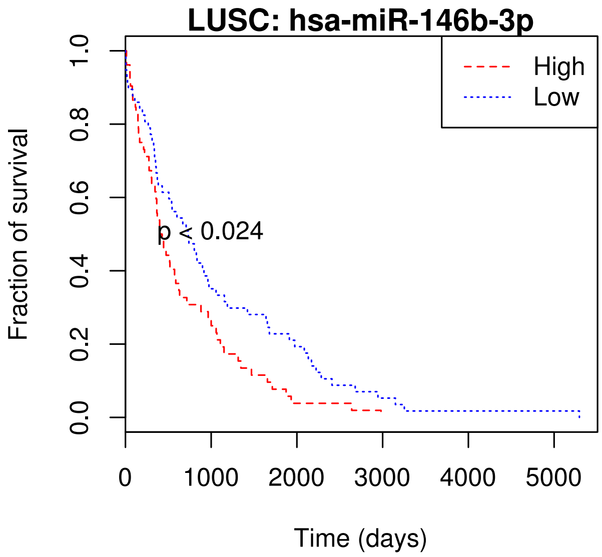
LUSC: hsa-miR-146b-3p
High
Low
p < 0.024
Fraction of survival
Time (days)

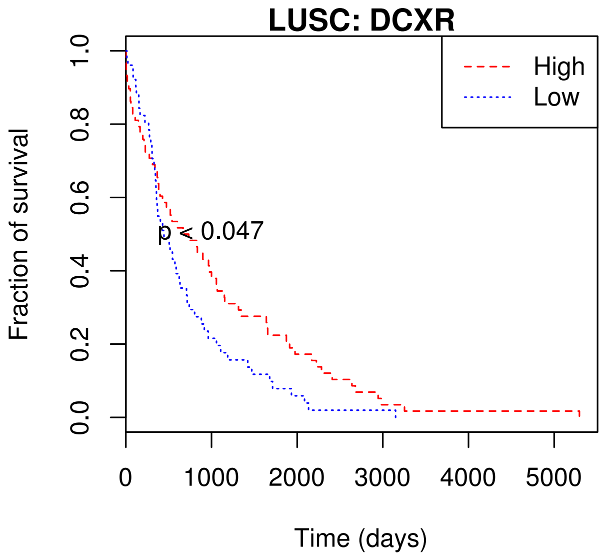
LUSC: DCXR
High
Low
p < 0.047
Fraction of survival
Time (days)

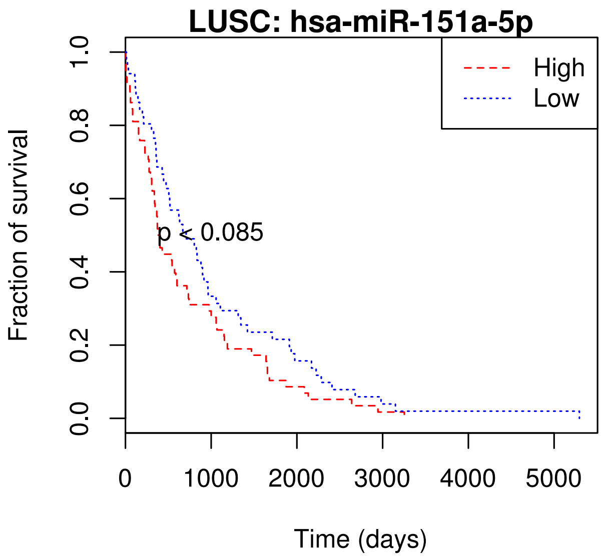
LUSC: hsa-miR-151a-5p
High
Low
p < 0.085
Fraction of survival
0.0
0.2
0.4
0.6
0.8
1.0
0
1000
2000
3000
4000
5000
Time (days)

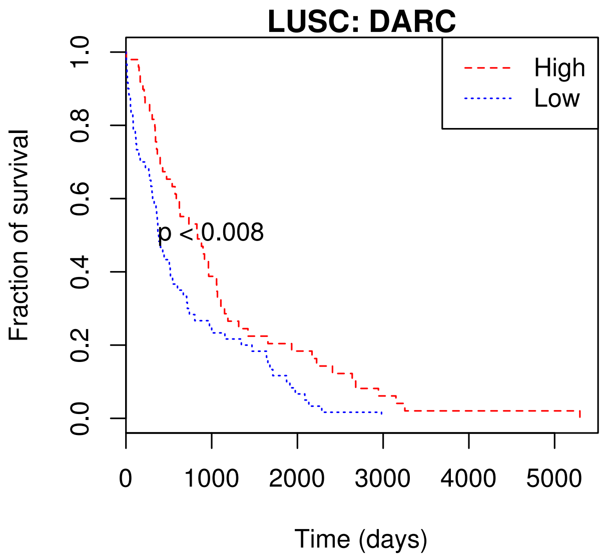
LUSC: DARC
High
Low
p < 0.008
Fraction of survival
0.0
0.2
0.4
0.6
0.8
1.0
0
1000
2000
3000
4000
5000
Time (days)

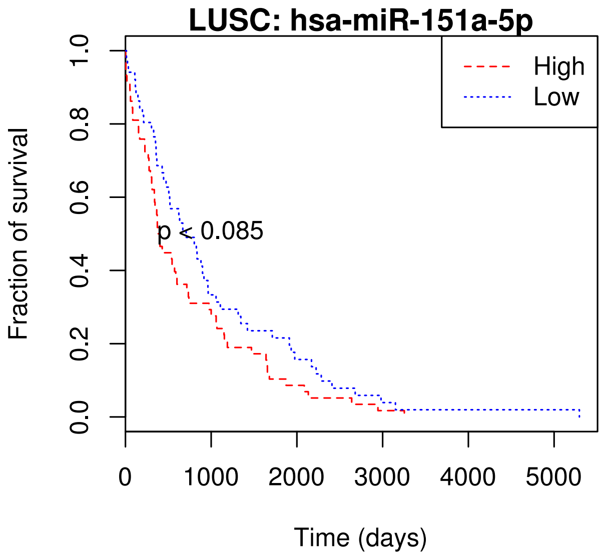
LUSC: hsa-miR-151a-5p
High
Low
p < 0.085
Fraction of survival
0.0
0.2
0.4
0.6
0.8
1.0
0
1000
2000
3000
4000
5000
Time (days)

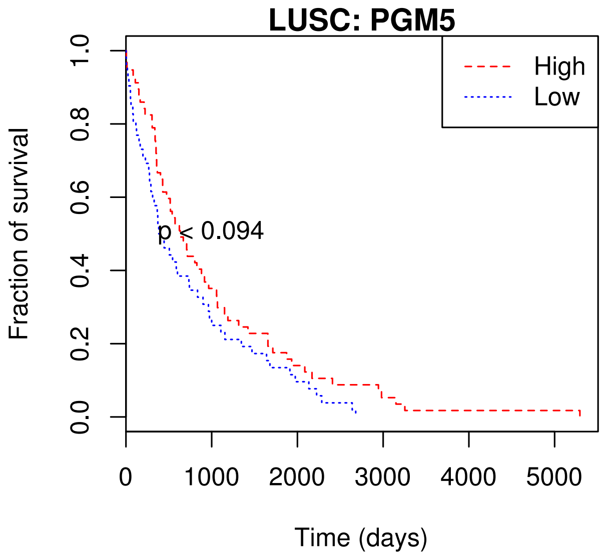
LUSC: PGM5
High
Low
p < 0.094
Fraction of survival
0.0
0.2
0.4
0.6
0.8
1.0
0
1000
2000
3000
4000
5000
Time (days)

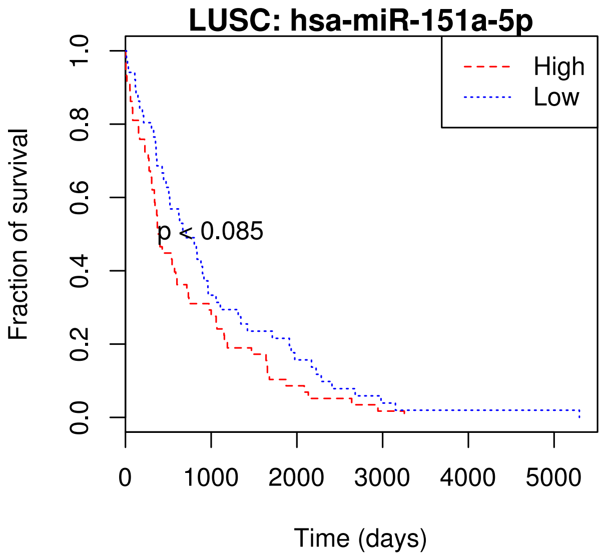
LUSC: hsa-miR-151a-5p
High
Low
p < 0.085
Fraction of survival
0.0
0.2
0.4
0.6
0.8
1.0
0
1000
2000
3000
4000
5000
Time (days)

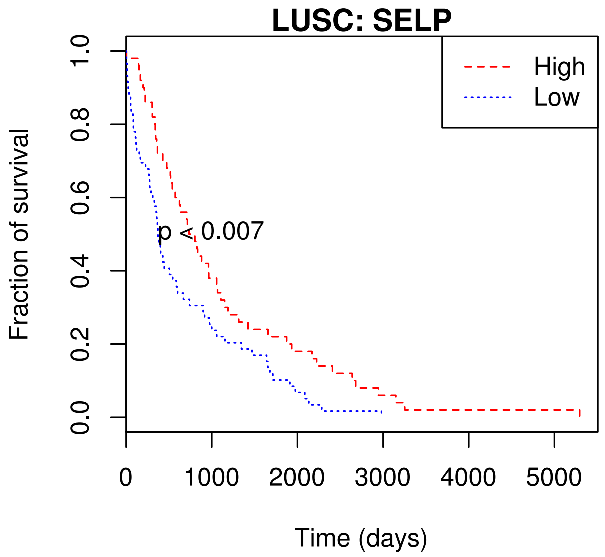
LUSC: SELP
High
Low
p < 0.007
Fraction of survival
0.0
0.2
0.4
0.6
0.8
1.0
0
1000
2000
3000
4000
5000
Time (days)

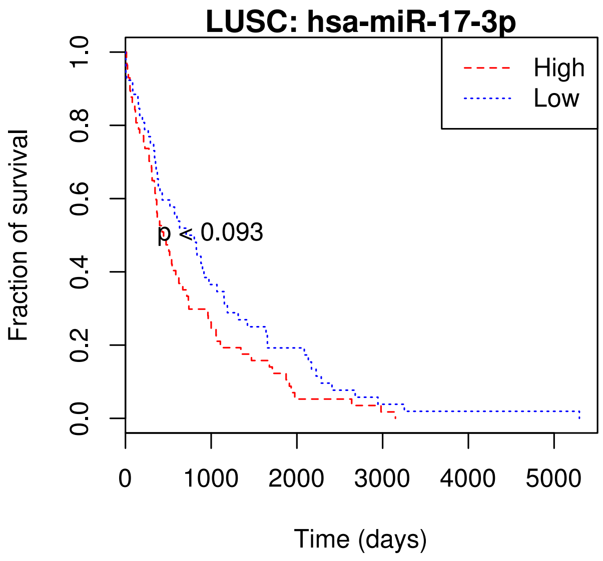
LUSC: hsa-miR-17-3p
High
Low
p < 0.093
Fraction of survival
Time (days)
0.0
0.2
0.4
0.6
0.8
1.0
0
1000
2000
3000
4000
5000

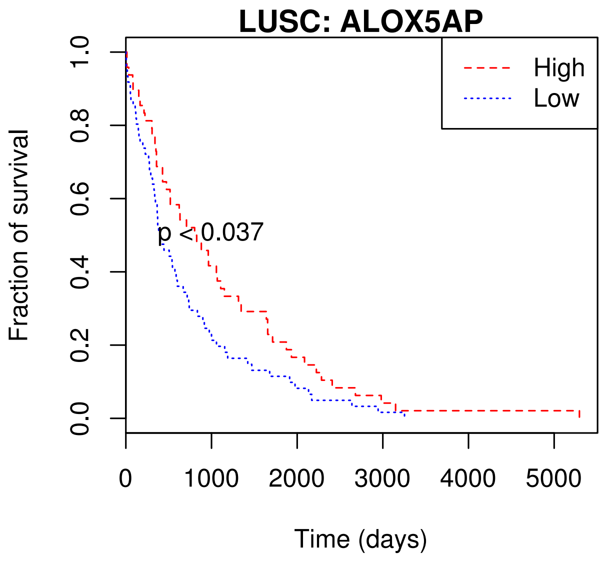
LUSC: ALOX5AP
High
Low
p < 0.037
Fraction of survival
Time (days)
0.0
0.2
0.4
0.6
0.8
1.0
0
1000
2000
3000
4000
5000

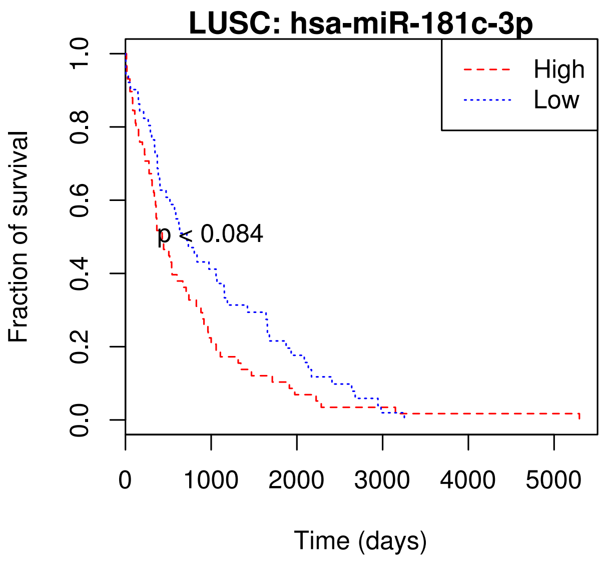
LUSC: hsa-miR-181c-3p
High
Low
p < 0.084
Fraction of survival
Time (days)
0.0
0.2
0.4
0.6
0.8
1.0
0
1000
2000
3000
4000
5000

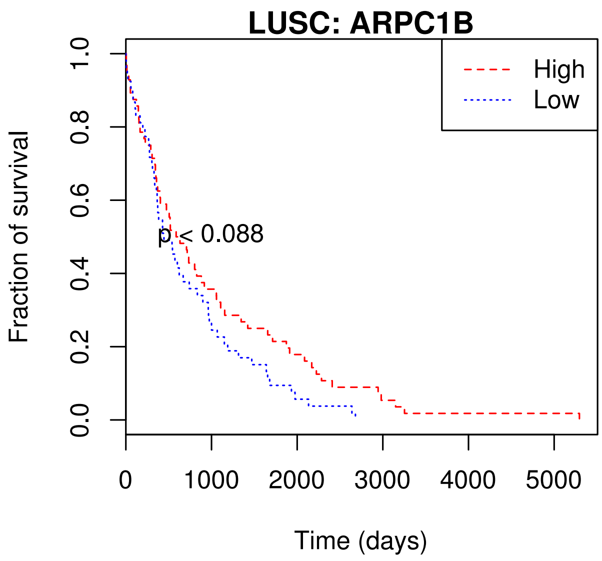
LUSC: ARPC1B
High
Low
p < 0.088
Fraction of survival
Time (days)
0.0
0.2
0.4
0.6
0.8
1.0
0
1000
2000
3000
4000
5000

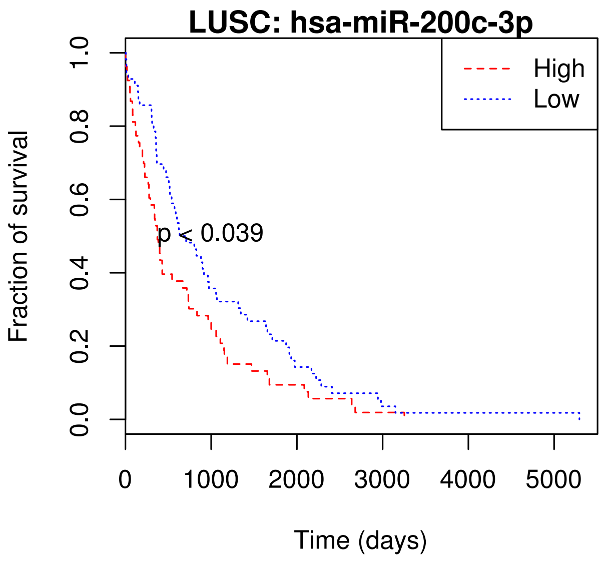
LUSC: hsa-miR-200c-3p
High
Low
p < 0.039
Fraction of survival
Time (days)
0.0
0.2
0.4
0.6
0.8
1.0
0
1000
2000
3000
4000
5000

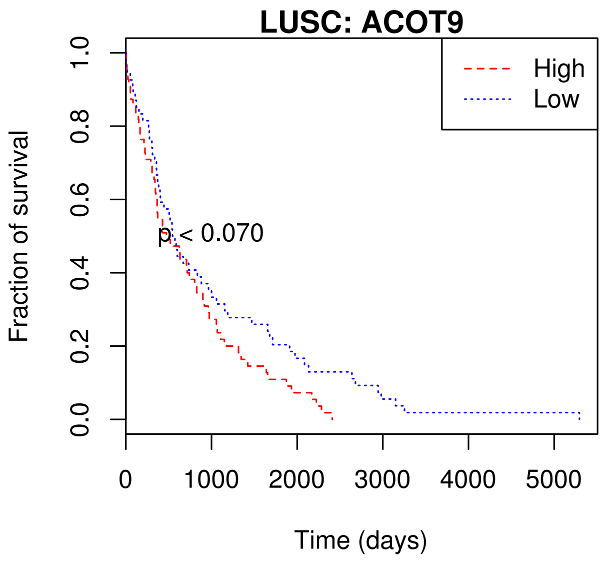
LUSC: ACOT9
High
Low
p < 0.070
Fraction of survival
Time (days)
0.0
0.2
0.4
0.6
0.8
1.0
0
1000
2000
3000
4000
5000

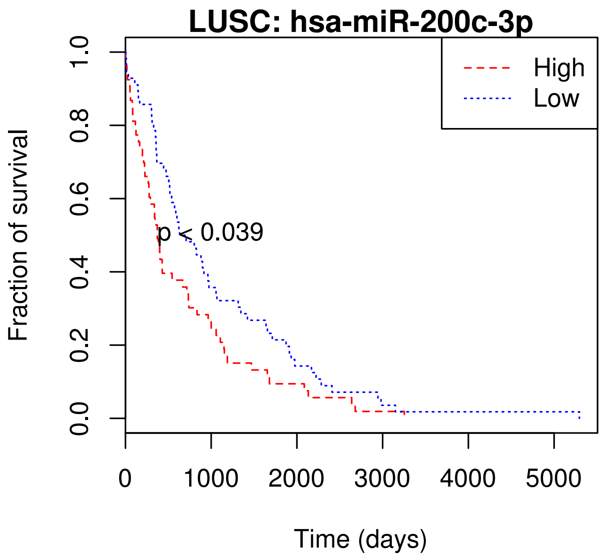
LUSC: hsa-miR-200c-3p
High
Low
p < 0.039
Fraction of survival
Time (days)
0.0 0.2 0.4 0.6 0.8 1.0
0 1000 2000 3000 4000 5000

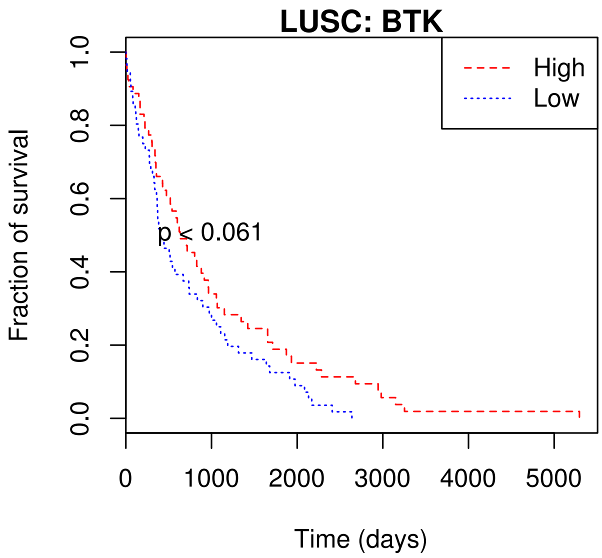
LUSC: BTK
High
Low
p < 0.061
Fraction of survival
Time (days)
0.0 0.2 0.4 0.6 0.8 1.0
0 1000 2000 3000 4000 5000

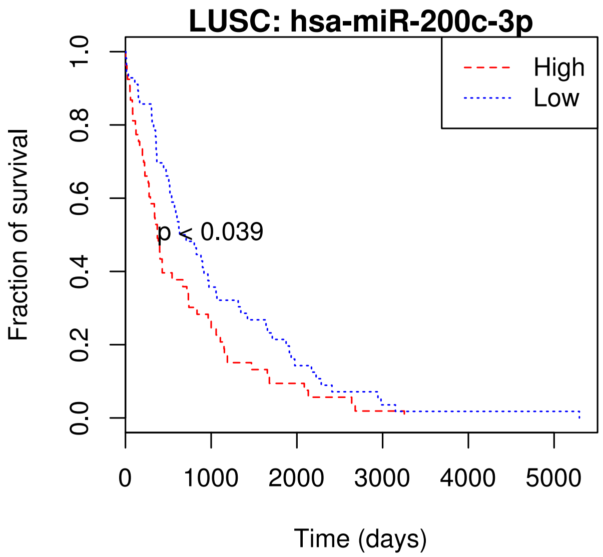
LUSC: hsa-miR-200c-3p
High
Low
p < 0.039
Fraction of survival
Time (days)
0.0 0.2 0.4 0.6 0.8 1.0
0 1000 2000 3000 4000 5000

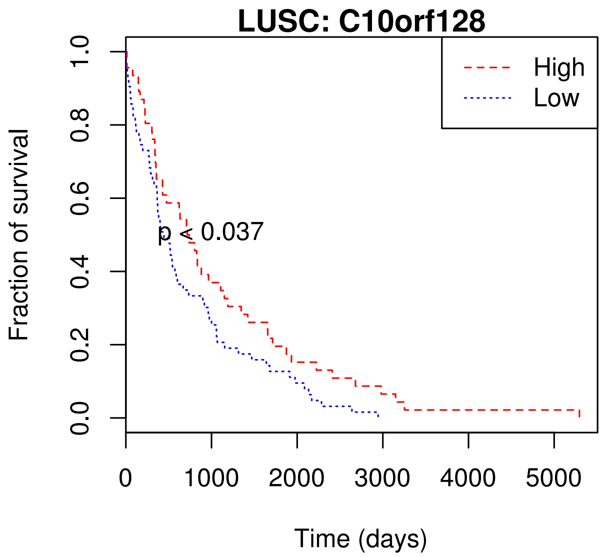
LUSC: C10orf128
High
Low
p < 0.037
Fraction of survival
Time (days)
0.0 0.2 0.4 0.6 0.8 1.0
0 1000 2000 3000 4000 5000

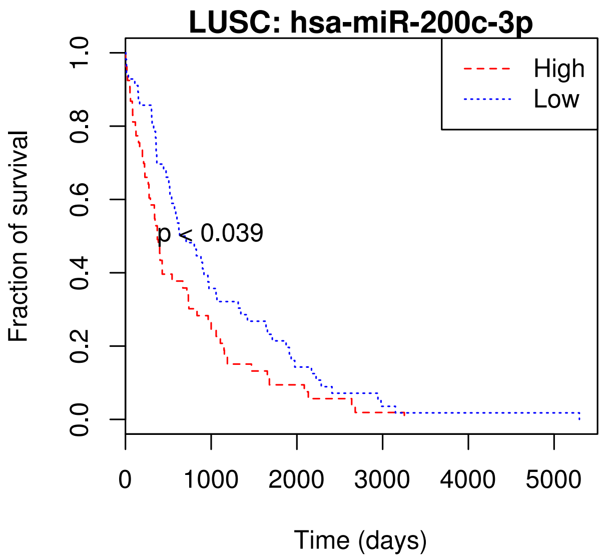
LUSC: hsa-miR-200c-3p
High
Low
p < 0.039
Fraction of survival
Time (days)
0.0 0.2 0.4 0.6 0.8 1.0
0 1000 2000 3000 4000 5000

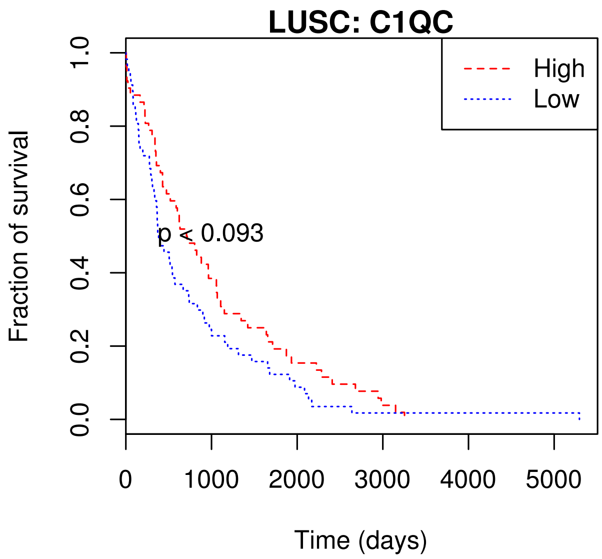
LUSC: C1QC
High
Low
p < 0.093
Fraction of survival
Time (days)
0.0 0.2 0.4 0.6 0.8 1.0
0 1000 2000 3000 4000 5000

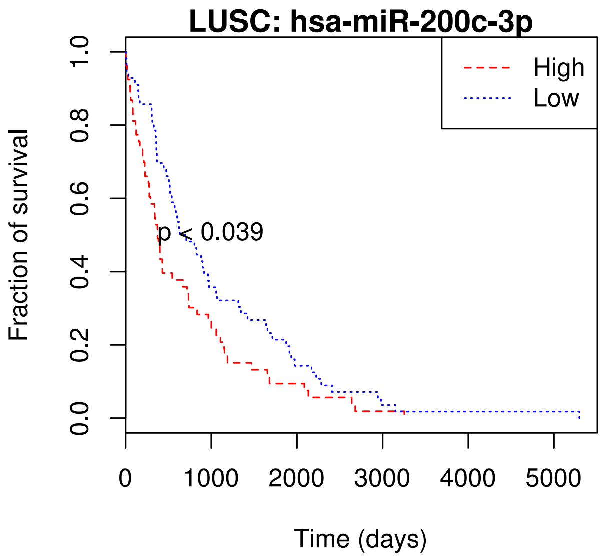
LUSC: hsa-miR-200c-3p
High
Low
p < 0.039
Fraction of survival
Time (days)
0.0 0.2 0.4 0.6 0.8 1.0
0 1000 2000 3000 4000 5000

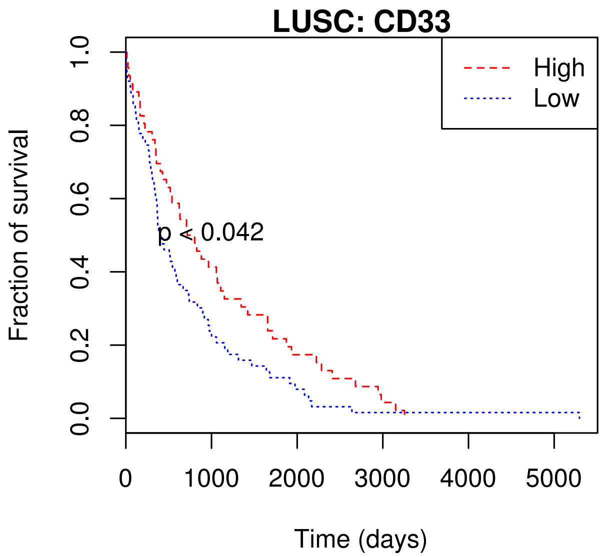
LUSC: CD33
High
Low
p < 0.042
Fraction of survival
Time (days)
0.0 0.2 0.4 0.6 0.8 1.0
0 1000 2000 3000 4000 5000

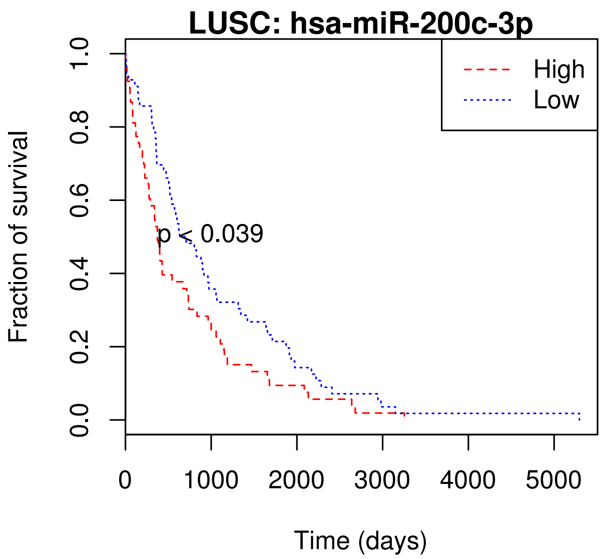
LUSC: hsa-miR-200c-3p
High
Low
p < 0.039
Fraction of survival
Time (days)
0.0 0.2 0.4 0.6 0.8 1.0
0 1000 2000 3000 4000 5000

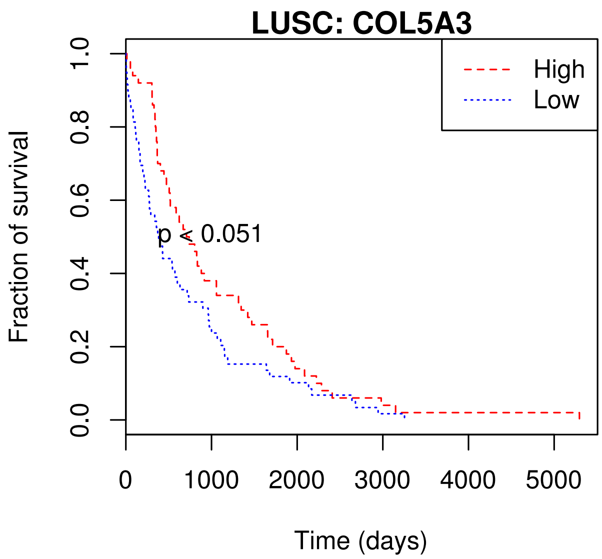
LUSC: COL5A3
High
Low
p < 0.051
Fraction of survival
Time (days)
0.0 0.2 0.4 0.6 0.8 1.0
0 1000 2000 3000 4000 5000

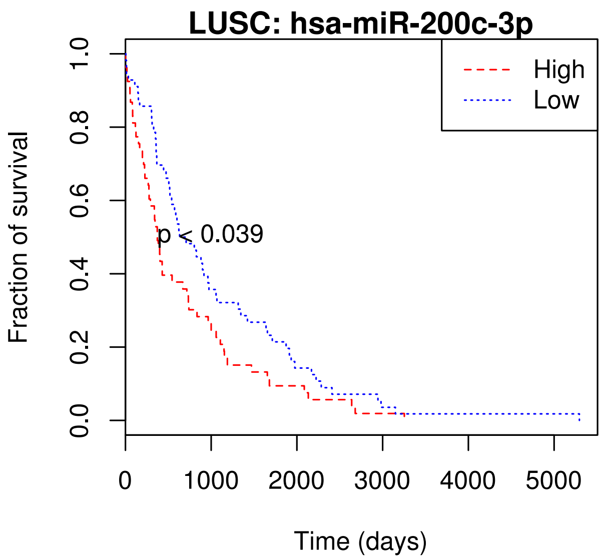
LUSC: hsa-miR-200c-3p
High
Low
p < 0.039
Fraction of survival
Time (days)
0.0 0.2 0.4 0.6 0.8 1.0
0 1000 2000 3000 4000 5000

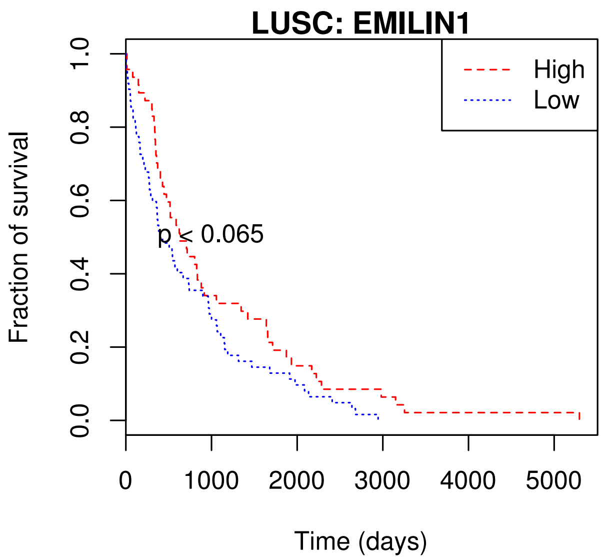
LUSC: EMILIN1
High
Low
p < 0.065
Fraction of survival
Time (days)
0.0 0.2 0.4 0.6 0.8 1.0
0 1000 2000 3000 4000 5000

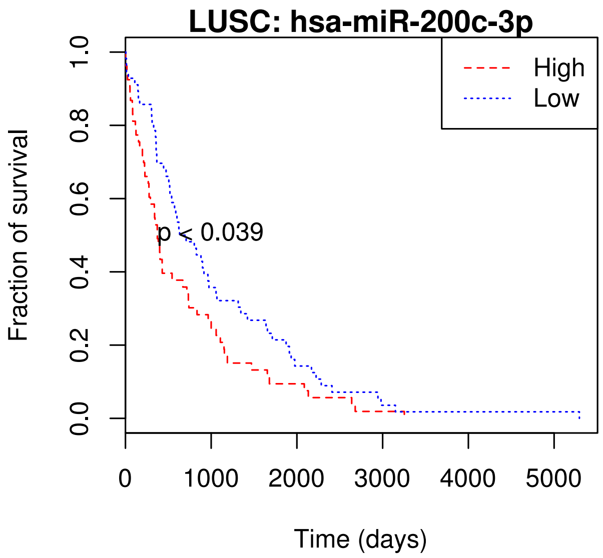
LUSC: hsa-miR-200c-3p
High
Low
p < 0.039
Fraction of survival
Time (days)
0.0
0.2
0.4
0.6
0.8
1.0
0
1000
2000
3000
4000
5000

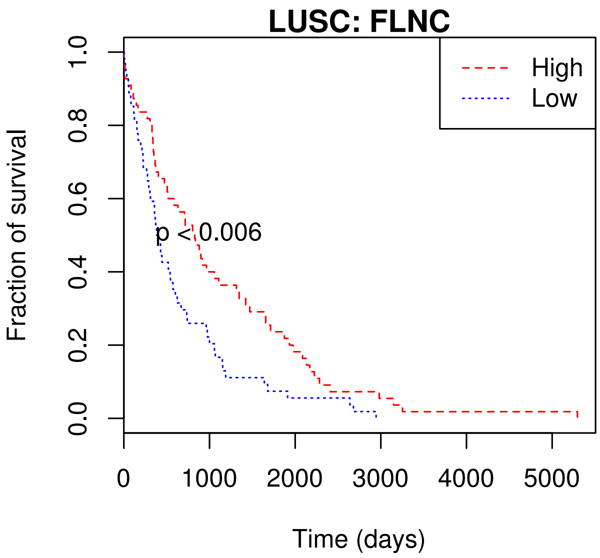
LUSC: FLNC
High
Low
p < 0.006
Fraction of survival
Time (days)
0.0
0.2
0.4
0.6
0.8
1.0
0
1000
2000
3000
4000
5000

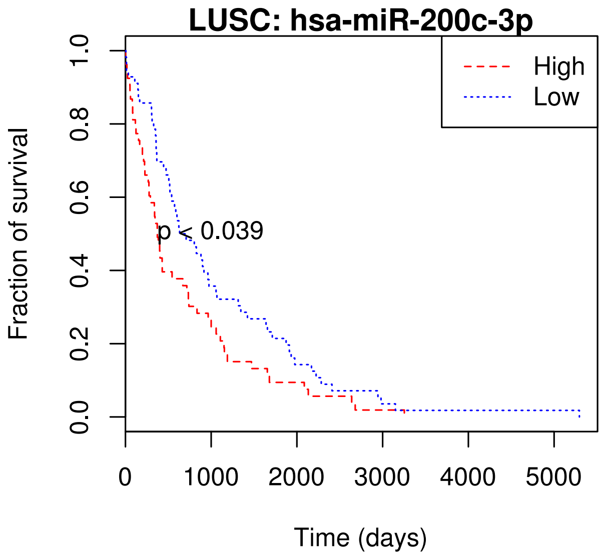
LUSC: hsa-miR-200c-3p
High
Low
p < 0.039
Fraction of survival
Time (days)
0.0
0.2
0.4
0.6
0.8
1.0
0
1000
2000
3000
4000
5000

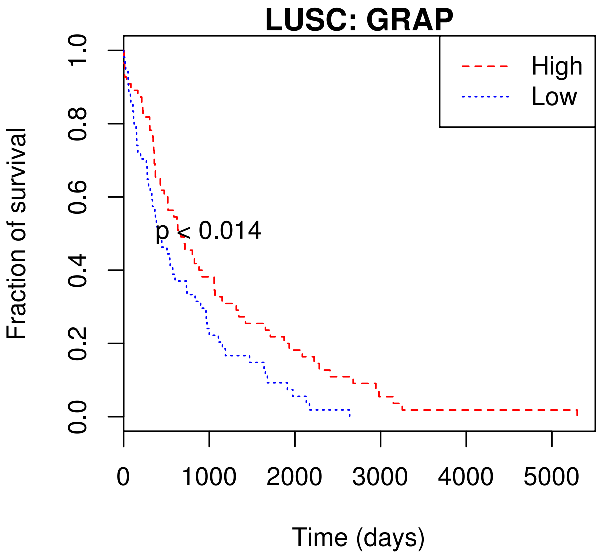
LUSC: GRAP
High
Low
p < 0.014
Fraction of survival
Time (days)
0.0
0.2
0.4
0.6
0.8
1.0
0
1000
2000
3000
4000
5000

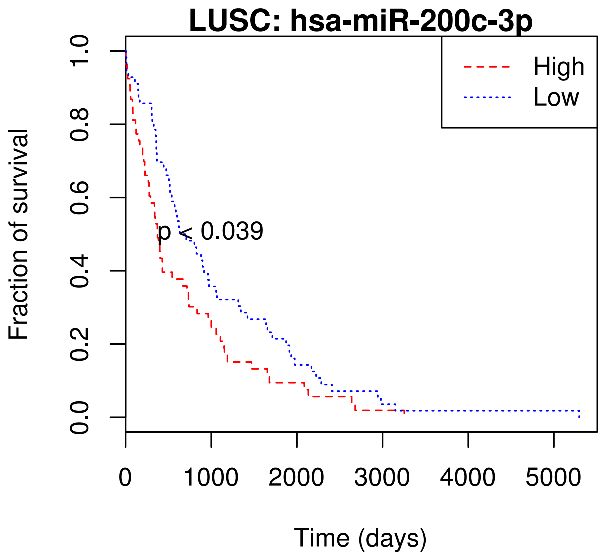
LUSC: hsa-miR-200c-3p
High
Low
p < 0.039
Fraction of survival
Time (days)
0.0
0.2
0.4
0.6
0.8
1.0
0
1000
2000
3000
4000
5000

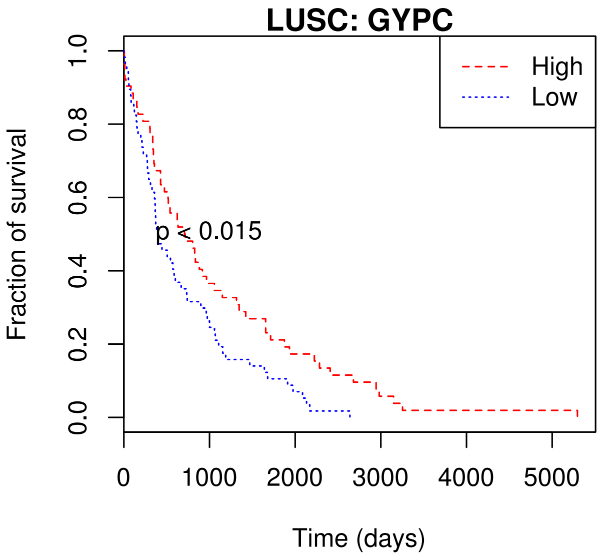
LUSC: GYPC
High
Low
p < 0.015
Fraction of survival
Time (days)
0.0
0.2
0.4
0.6
0.8
1.0
0
1000
2000
3000
4000
5000

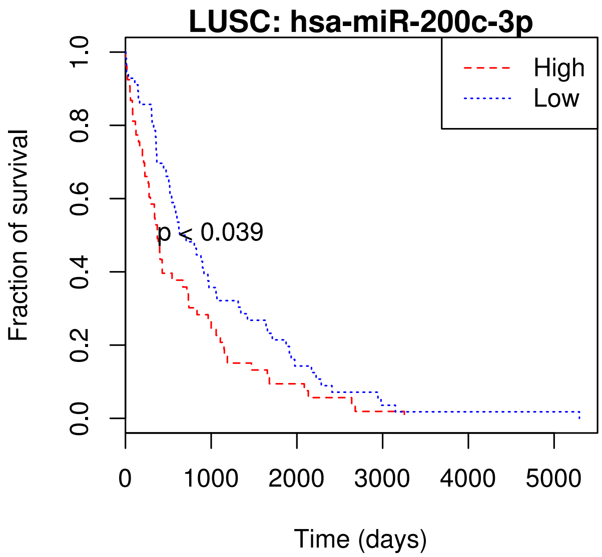
LUSC: hsa-miR-200c-3p
High
Low
p < 0.039
Fraction of survival
Time (days)
0.0 0.2 0.4 0.6 0.8 1.0
0 1000 2000 3000 4000 5000

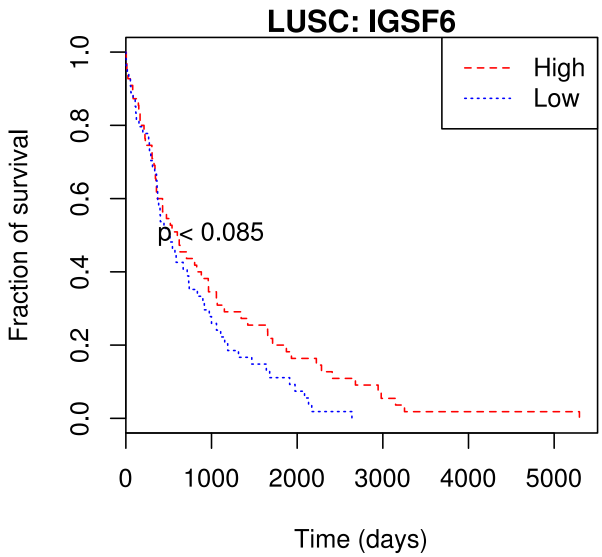
LUSC: IGSF6
High
Low
p < 0.085
Fraction of survival
Time (days)
0.0 0.2 0.4 0.6 0.8 1.0
0 1000 2000 3000 4000 5000

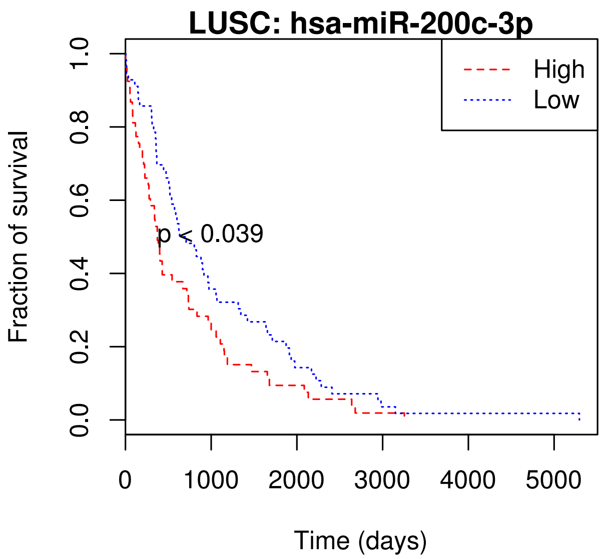
LUSC: hsa-miR-200c-3p
High
Low
p < 0.039
Fraction of survival
Time (days)
0.0 0.2 0.4 0.6 0.8 1.0
0 1000 2000 3000 4000 5000

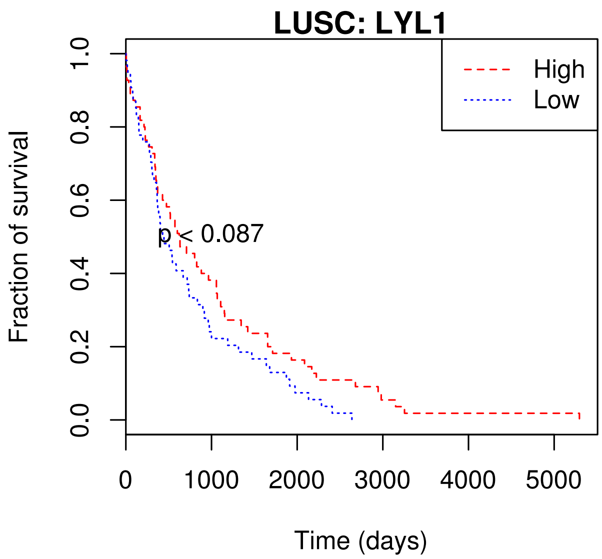
LUSC: LYL1
High
Low
p < 0.087
Fraction of survival
Time (days)
0.0 0.2 0.4 0.6 0.8 1.0
0 1000 2000 3000 4000 5000

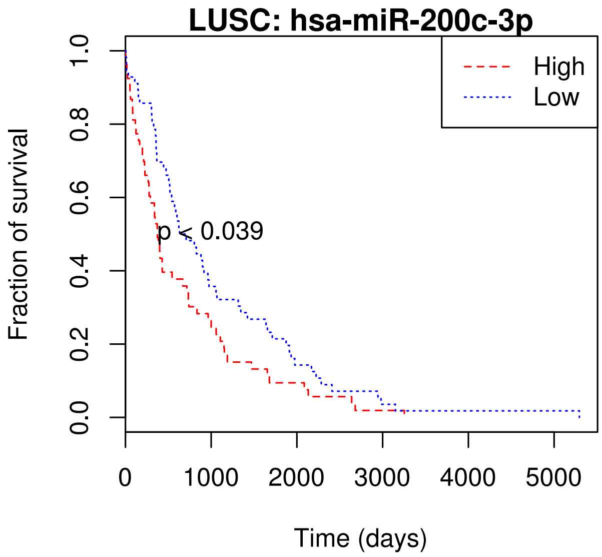
LUSC: hsa-miR-200c-3p
High
Low
p < 0.039
Fraction of survival
Time (days)
0.0 0.2 0.4 0.6 0.8 1.0
0 1000 2000 3000 4000 5000

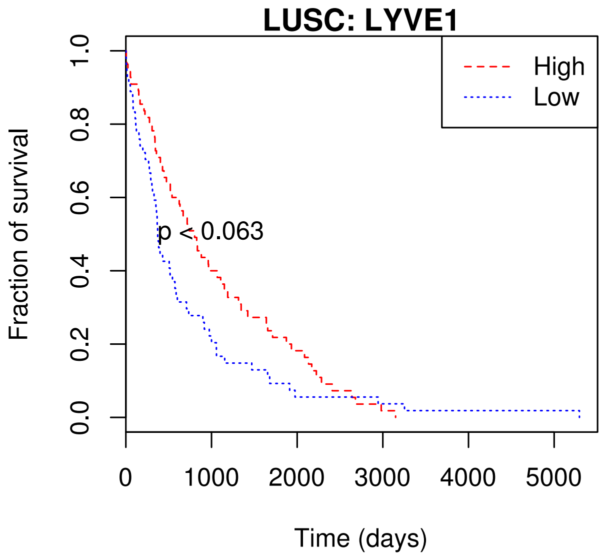
LUSC: LYVE1
High
Low
p < 0.063
Fraction of survival
Time (days)
0.0 0.2 0.4 0.6 0.8 1.0
0 1000 2000 3000 4000 5000

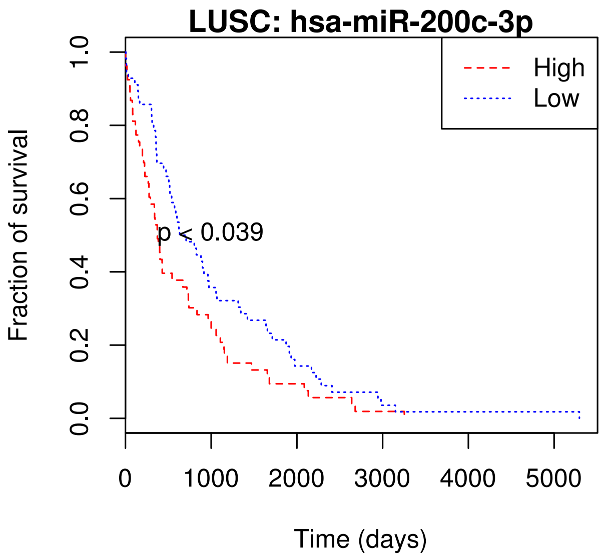
LUSC: hsa-miR-200c-3p
High
Low
p < 0.039
Fraction of survival
Time (days)
0.0 0.2 0.4 0.6 0.8 1.0
0 1000 2000 3000 4000 5000

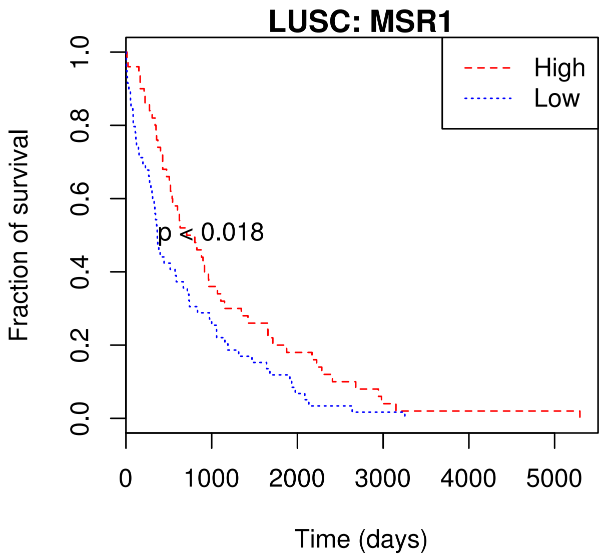
LUSC: MSR1
High
Low
p < 0.018
Fraction of survival
Time (days)
0.0 0.2 0.4 0.6 0.8 1.0
0 1000 2000 3000 4000 5000

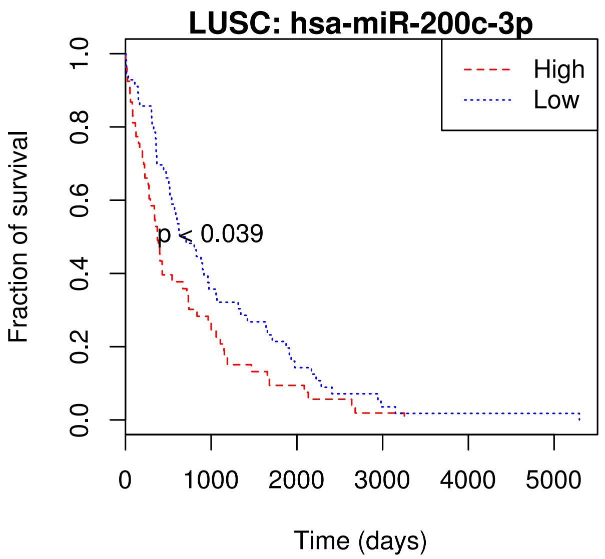
LUSC: hsa-miR-200c-3p
High
Low
p < 0.039
Fraction of survival
Time (days)
0.0 0.2 0.4 0.6 0.8 1.0
0 1000 2000 3000 4000 5000

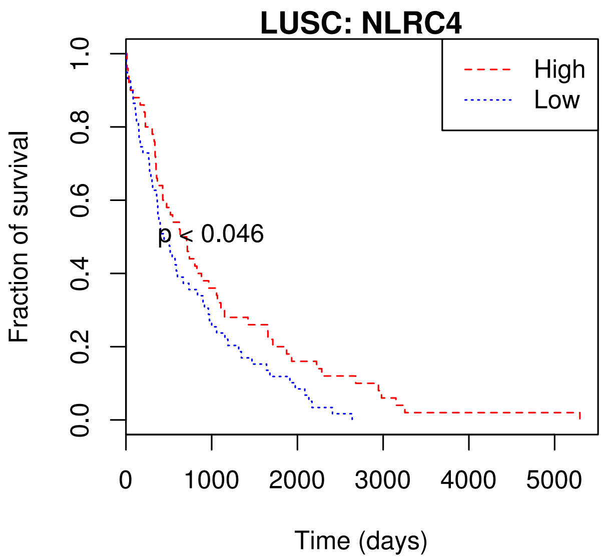
LUSC: NLRC4
High
Low
p < 0.046
Fraction of survival
Time (days)
0.0 0.2 0.4 0.6 0.8 1.0
0 1000 2000 3000 4000 5000

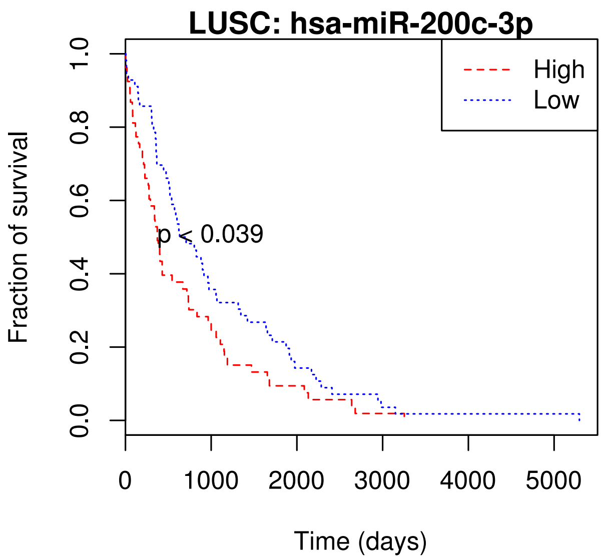
LUSC: hsa-miR-200c-3p
High
Low
p < 0.039
Fraction of survival
Time (days)
0.0 0.2 0.4 0.6 0.8 1.0
0 1000 2000 3000 4000 5000

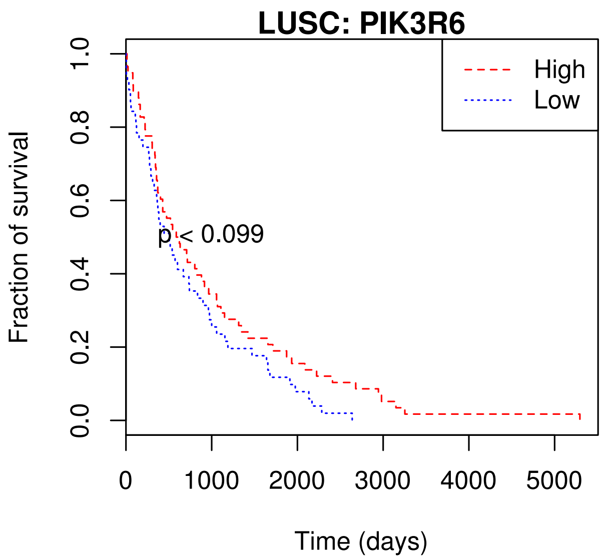
LUSC: PIK3R6
High
Low
p < 0.099
Fraction of survival
Time (days)
0.0 0.2 0.4 0.6 0.8 1.0
0 1000 2000 3000 4000 5000

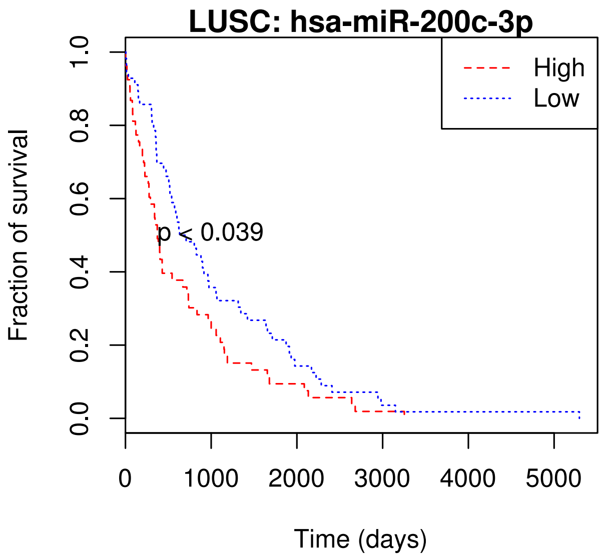
LUSC: hsa-miR-200c-3p
High
Low
p < 0.039
Fraction of survival
0.0
0.2
0.4
0.6
0.8
1.0
0
1000
2000
3000
4000
5000
Time (days)

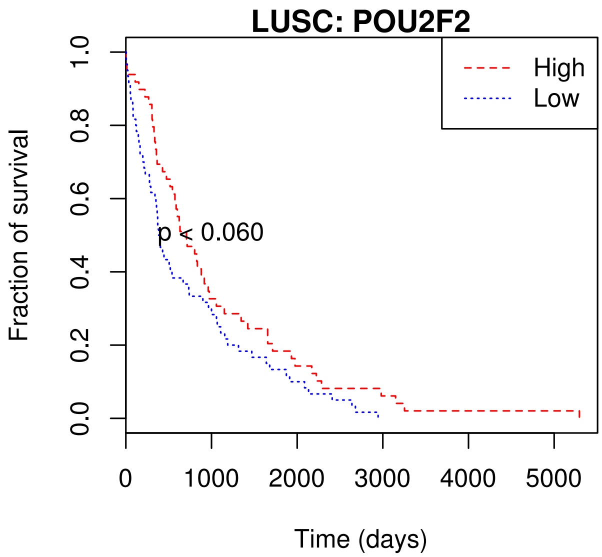
LUSC: POU2F2
High
Low
p < 0.060
Fraction of survival
0.0
0.2
0.4
0.6
0.8
1.0
0
1000
2000
3000
4000
5000
Time (days)

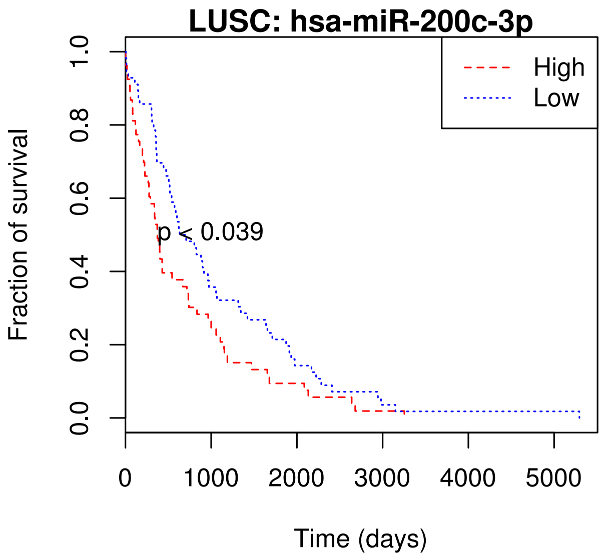
LUSC: hsa-miR-200c-3p
High
Low
p < 0.039
Fraction of survival
0.0
0.2
0.4
0.6
0.8
1.0
0
1000
2000
3000
4000
5000
Time (days)

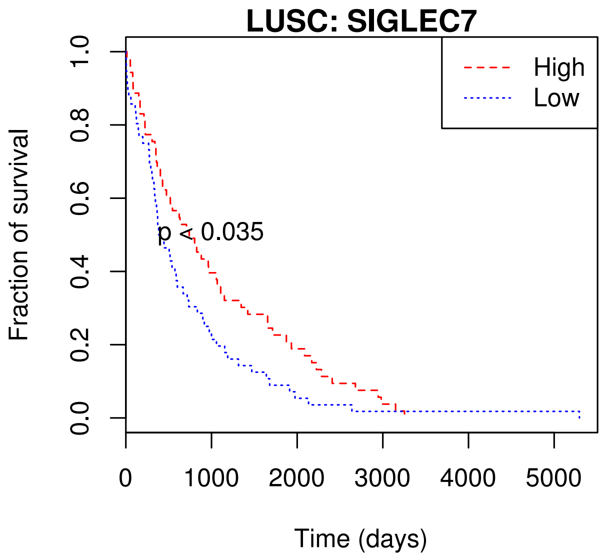
LUSC: SIGLEC7
High
Low
p < 0.035
Fraction of survival
0.0
0.2
0.4
0.6
0.8
1.0
0
1000
2000
3000
4000
5000
Time (days)

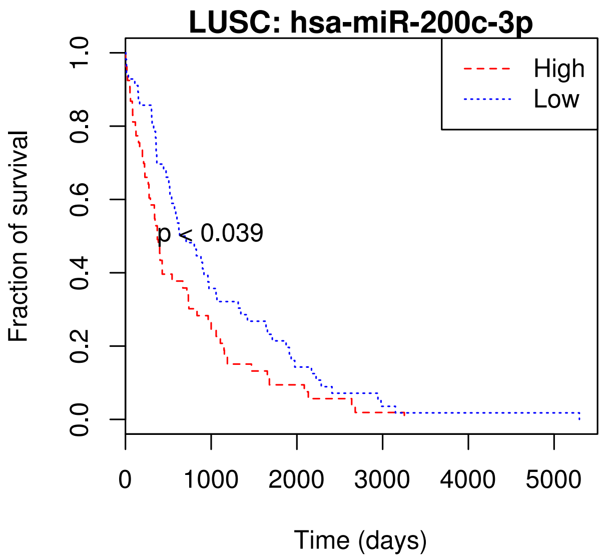
LUSC: hsa-miR-200c-3p
High
Low
p < 0.039
Fraction of survival
0.0
0.2
0.4
0.6
0.8
1.0
0
1000
2000
3000
4000
5000
Time (days)

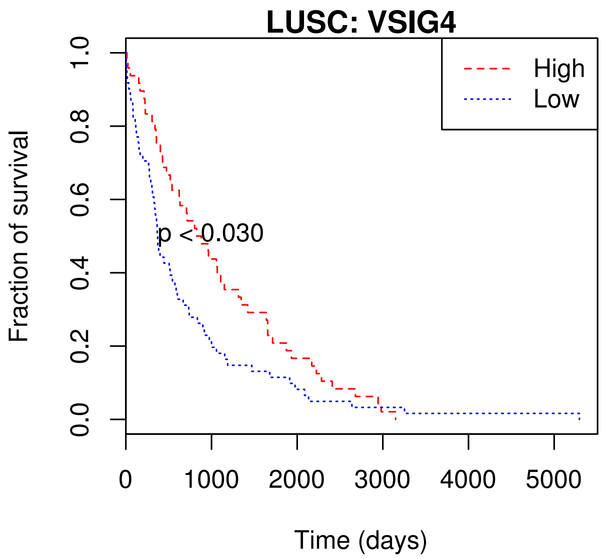
LUSC: VSIG4
High
Low
p < 0.030
Fraction of survival
0.0
0.2
0.4
0.6
0.8
1.0
0
1000
2000
3000
4000
5000
Time (days)

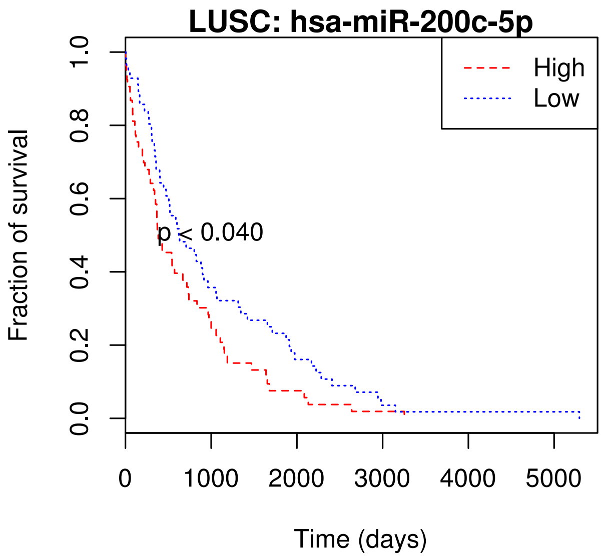
LUSC: hsa-miR-200c-5p
High
Low
p < 0.040
Fraction of survival
Time (days)
0.0 0.2 0.4 0.6 0.8 1.0
0 1000 2000 3000 4000 5000

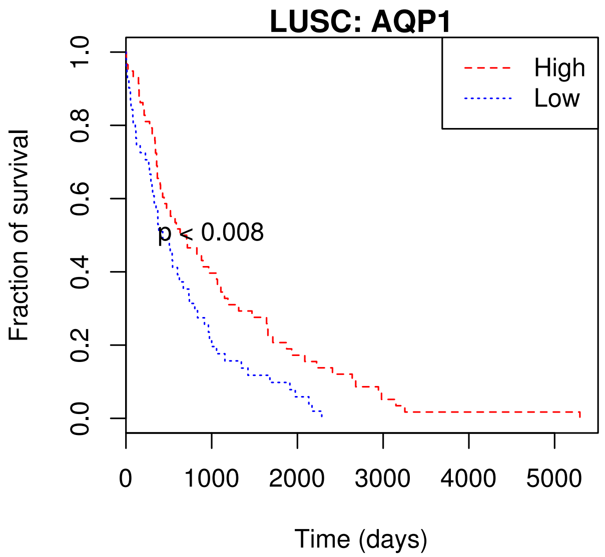
LUSC: AQP1
High
Low
p < 0.008
Fraction of survival
Time (days)
0.0 0.2 0.4 0.6 0.8 1.0
0 1000 2000 3000 4000 5000

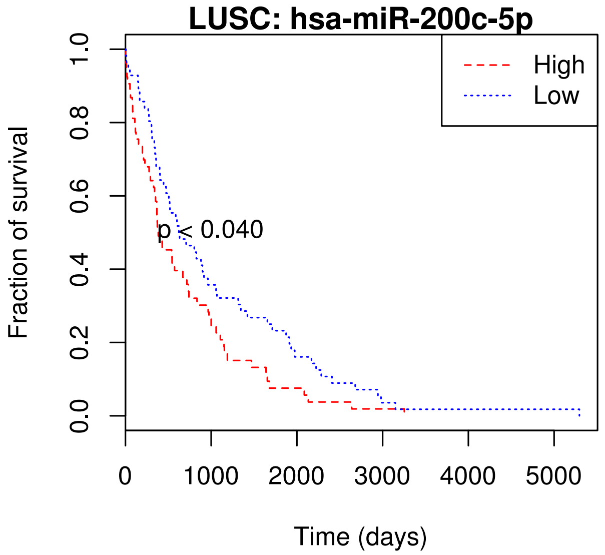
LUSC: hsa-miR-200c-5p
High
Low
p < 0.040
Fraction of survival
Time (days)
0.0 0.2 0.4 0.6 0.8 1.0
0 1000 2000 3000 4000 5000

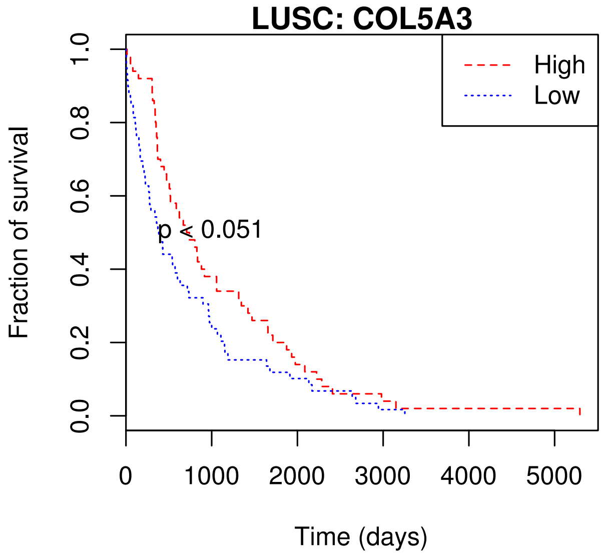
LUSC: COL5A3
High
Low
p < 0.051
Fraction of survival
Time (days)
0.0 0.2 0.4 0.6 0.8 1.0
0 1000 2000 3000 4000 5000

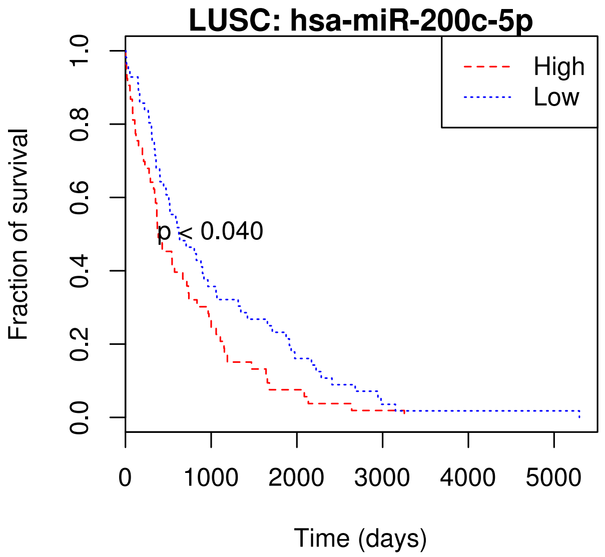
LUSC: hsa-miR-200c-5p
High
Low
p < 0.040
Fraction of survival
Time (days)
0.0 0.2 0.4 0.6 0.8 1.0
0 1000 2000 3000 4000 5000

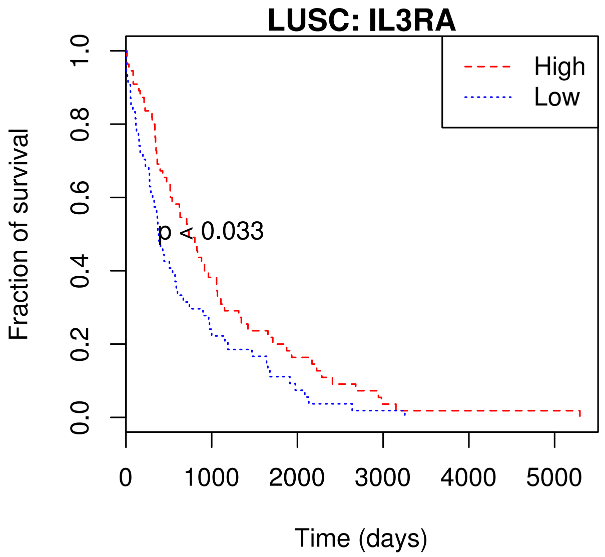
LUSC: IL3RA
High
Low
p < 0.033
Fraction of survival
Time (days)
0.0 0.2 0.4 0.6 0.8 1.0
0 1000 2000 3000 4000 5000

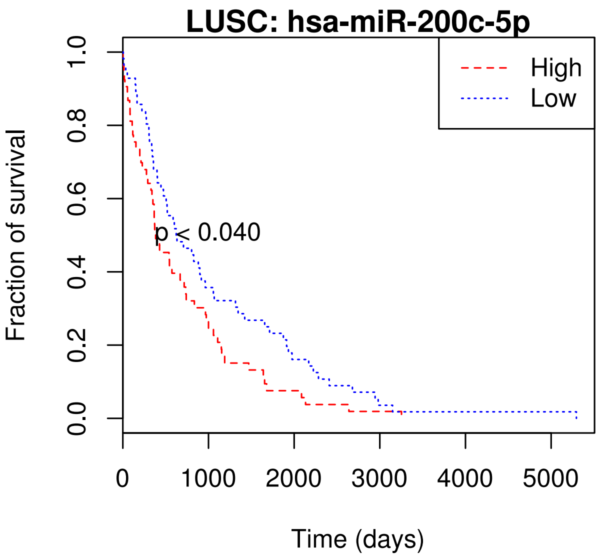
LUSC: hsa-miR-200c-5p
Fraction of survival
0.0 0.2 0.4 0.6 0.8 1.0
Time (days)
0 1000 2000 3000 4000 5000
High
Low
p < 0.040

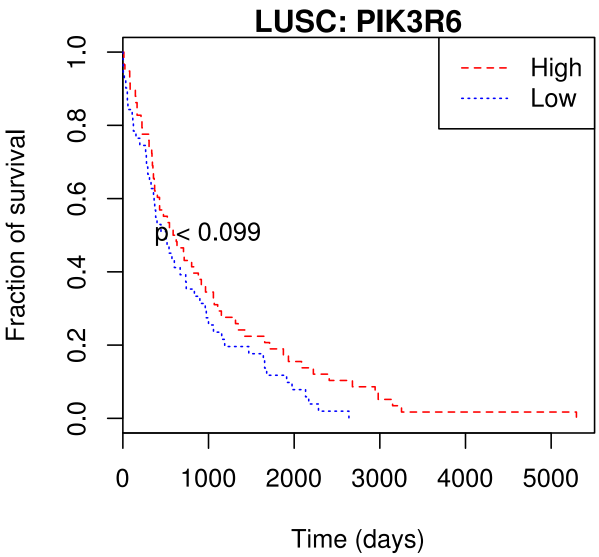
LUSC: PIK3R6
Fraction of survival
0.0 0.2 0.4 0.6 0.8 1.0
Time (days)
0 1000 2000 3000 4000 5000
High
Low
p < 0.099

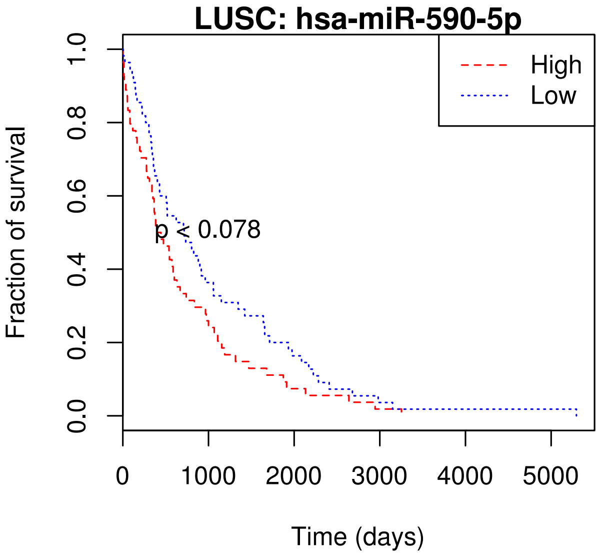
LUSC: hsa-miR-590-5p
Fraction of survival
0.0 0.2 0.4 0.6 0.8 1.0
Time (days)
0 1000 2000 3000 4000 5000
High
Low
p < 0.078

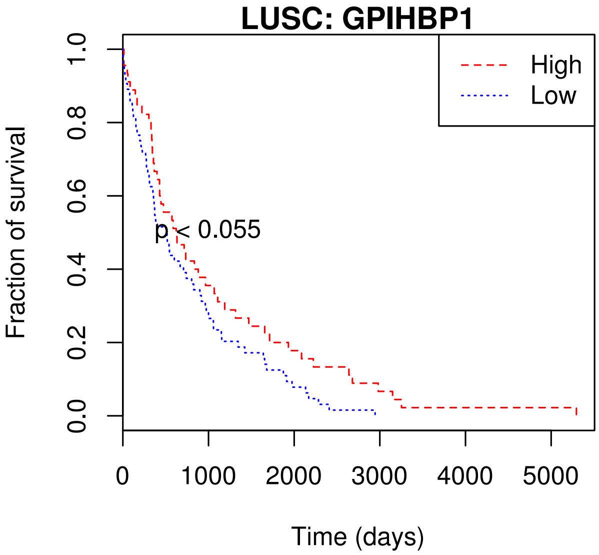
LUSC: GPIHBP1
Fraction of survival
0.0 0.2 0.4 0.6 0.8 1.0
Time (days)
0 1000 2000 3000 4000 5000
High
Low
p < 0.055

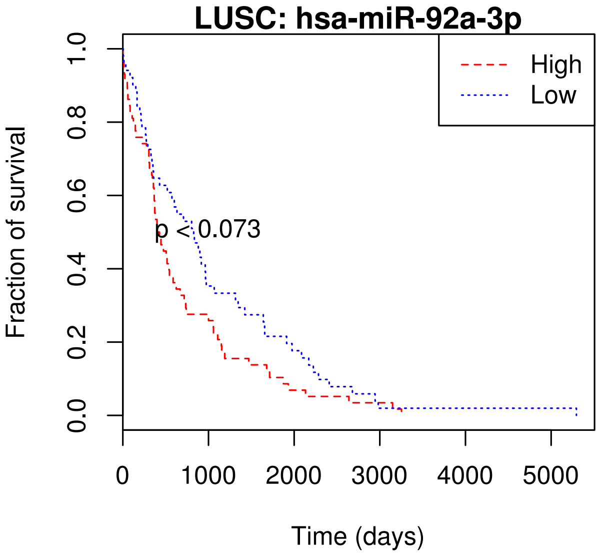
LUSC: hsa-miR-92a-3p
Fraction of survival
0.0 0.2 0.4 0.6 0.8 1.0
Time (days)
0 1000 2000 3000 4000 5000
High
Low
p < 0.073

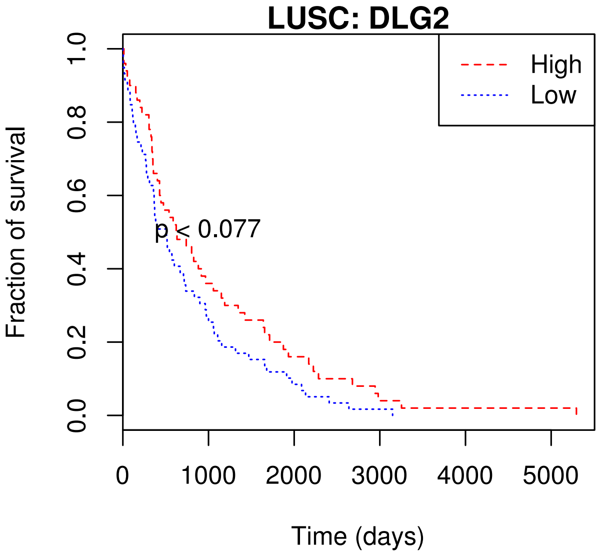
LUSC: DLG2
Fraction of survival
0.0 0.2 0.4 0.6 0.8 1.0
Time (days)
0 1000 2000 3000 4000 5000
High
Low
p < 0.077

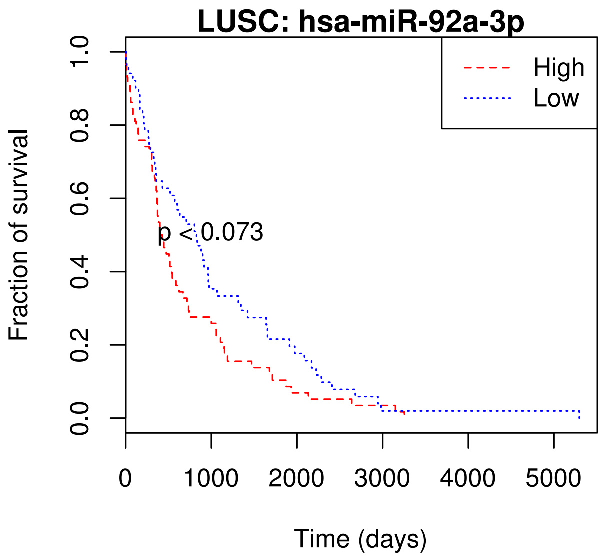
LUSC: hsa-miR-92a-3p
High
Low
p < 0.073
Fraction of survival
Time (days)
0.0 0.2 0.4 0.6 0.8 1.0
0 1000 2000 3000 4000 5000

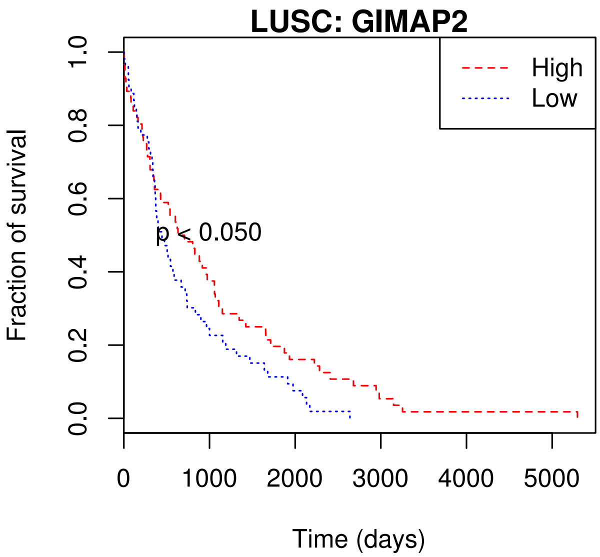
LUSC: GIMAP2
High
Low
p < 0.050
Fraction of survival
Time (days)
0.0 0.2 0.4 0.6 0.8 1.0
0 1000 2000 3000 4000 5000

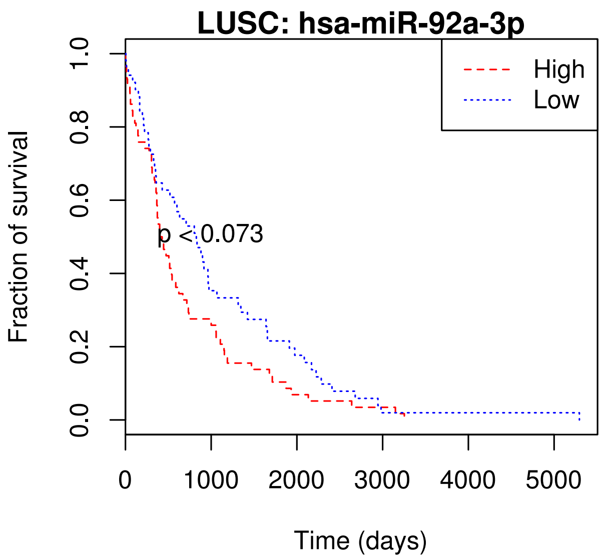
LUSC: hsa-miR-92a-3p
High
Low
p < 0.073
Fraction of survival
Time (days)
0.0 0.2 0.4 0.6 0.8 1.0
0 1000 2000 3000 4000 5000

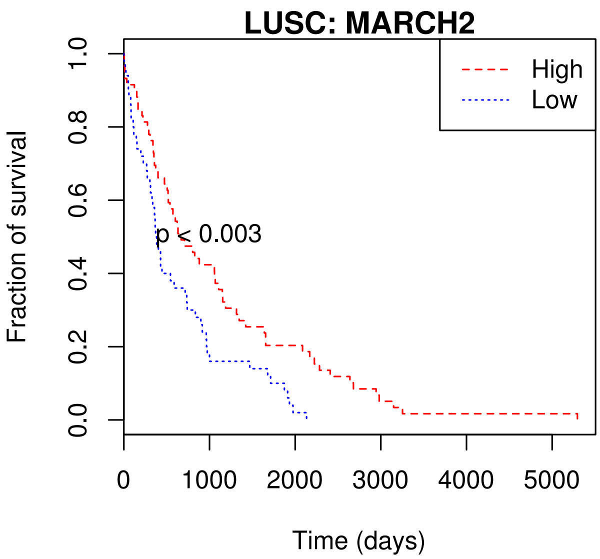
LUSC: MARCH2
High
Low
p < 0.003
Fraction of survival
Time (days)
0.0 0.2 0.4 0.6 0.8 1.0
0 1000 2000 3000 4000 5000

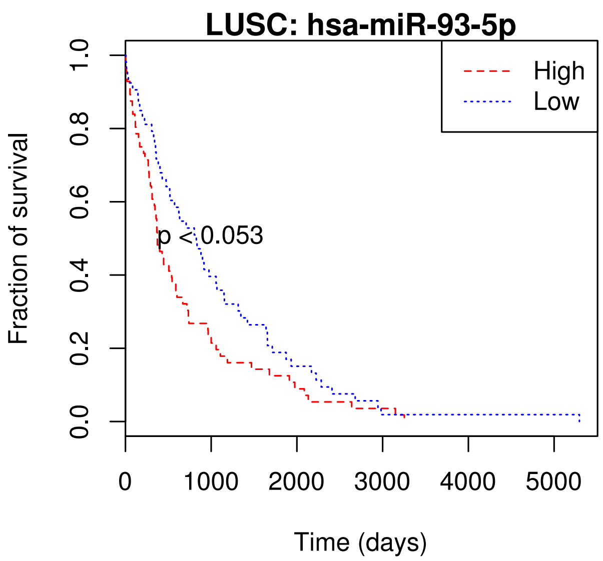
LUSC: hsa-miR-93-5p
High
Low
p < 0.053
Fraction of survival
Time (days)
0.0 0.2 0.4 0.6 0.8 1.0
0 1000 2000 3000 4000 5000

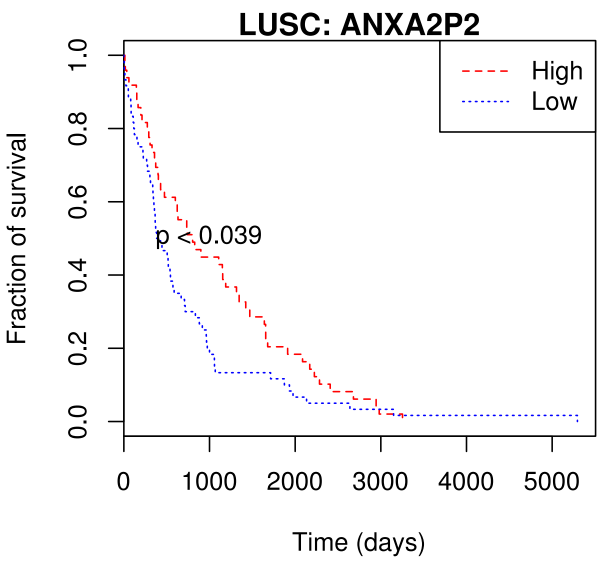
LUSC: ANXA2P2
High
Low
p < 0.039
Fraction of survival
Time (days)
0.0 0.2 0.4 0.6 0.8 1.0
0 1000 2000 3000 4000 5000

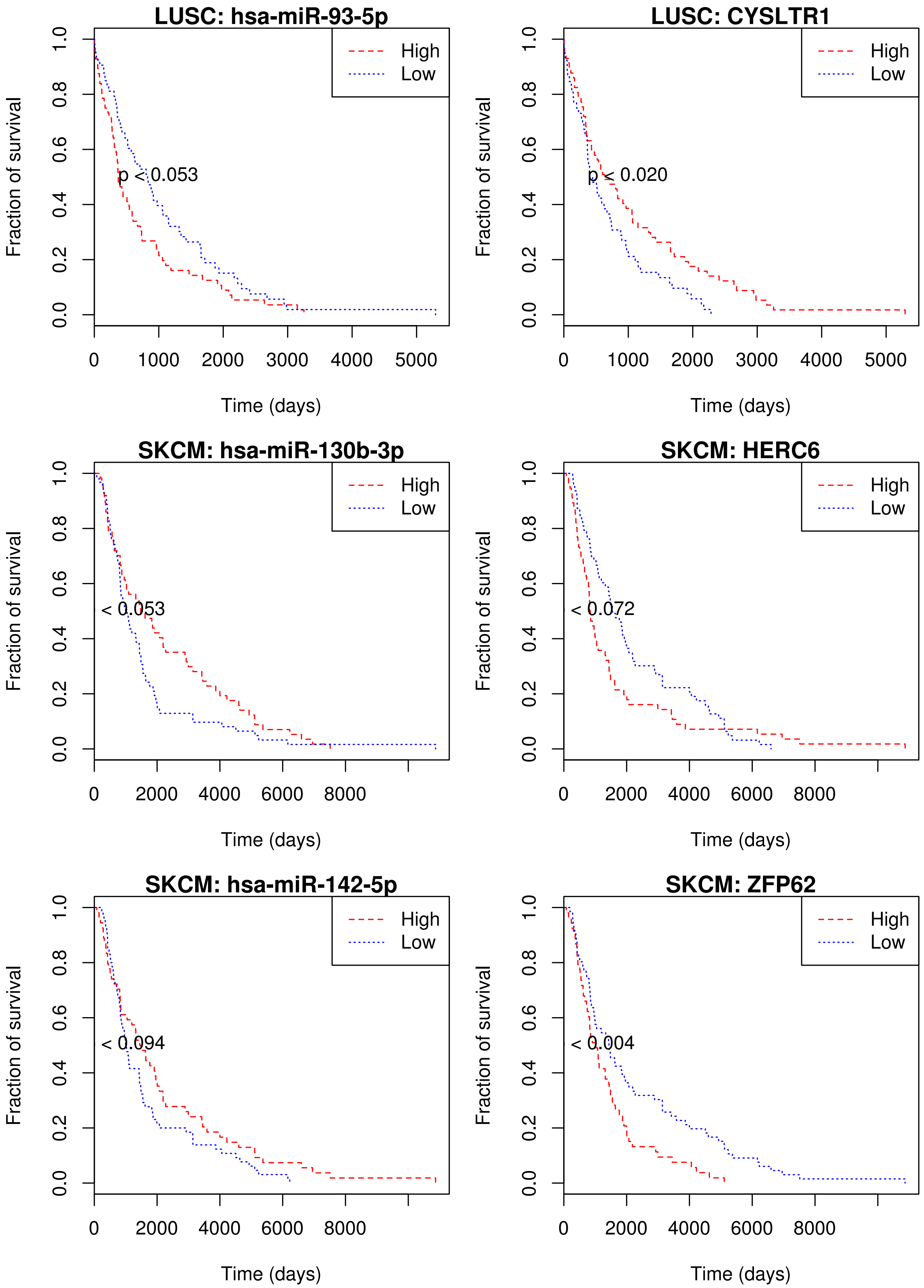
LUSC: hsa-miR-93-5p
High
Low
p < 0.053
LUSC: CYSLTR1
High
Low
p < 0.020
SKCM: hsa-miR-130b-3p
High
Low
< 0.053
SKCM: HERC6
High
Low
< 0.072
SKCM: hsa-miR-142-5p
High
Low
< 0.094
SKCM: ZFP62
High
Low
< 0.004
Fraction of survival
Time (days)

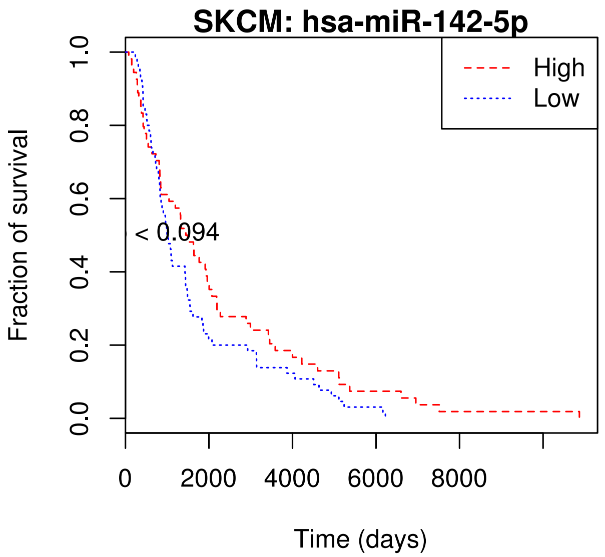
SKCM: hsa-miR-142-5p
High
Low
< 0.094
Fraction of survival
Time (days)

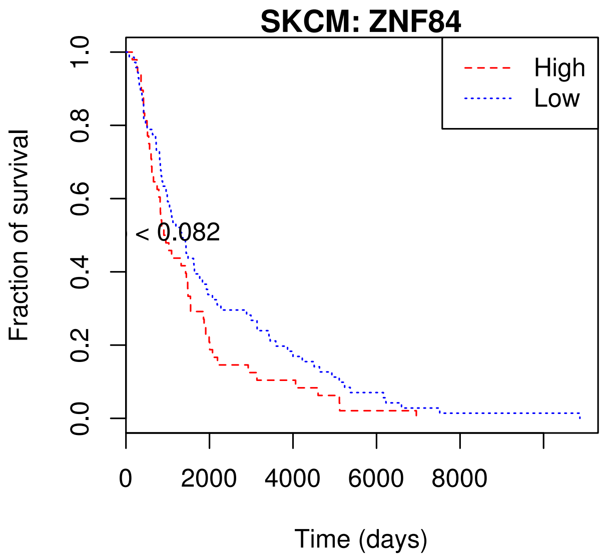
SKCM: ZNF84
High
Low
< 0.082
Fraction of survival
Time (days)

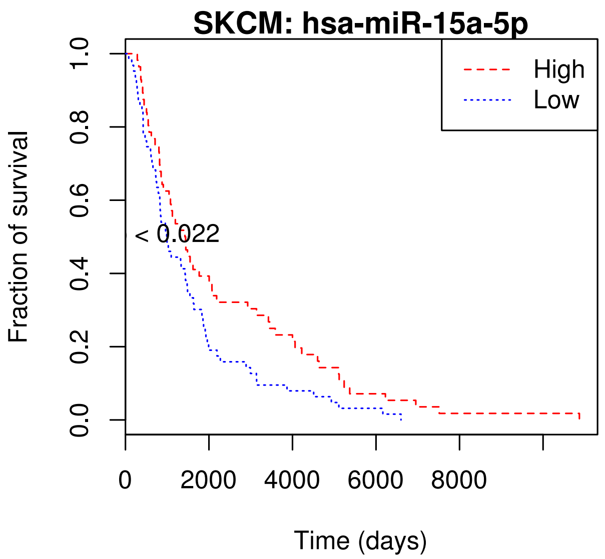
SKCM: hsa-miR-15a-5p
High
Low
< 0.022
Fraction of survival
Time (days)

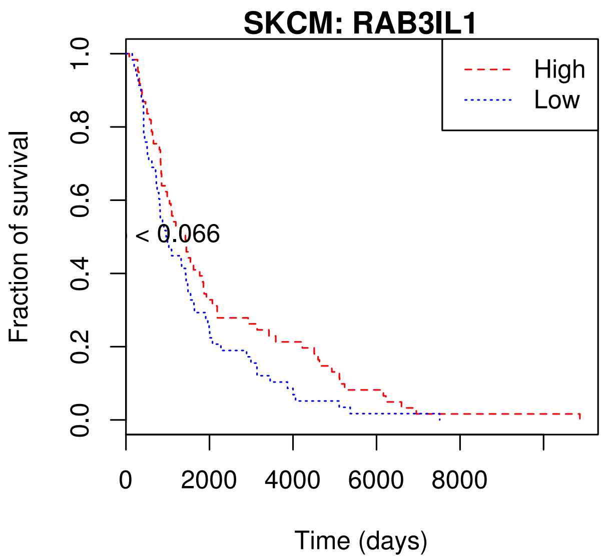
SKCM: RAB3IL1
High
Low
< 0.066
Fraction of survival
Time (days)

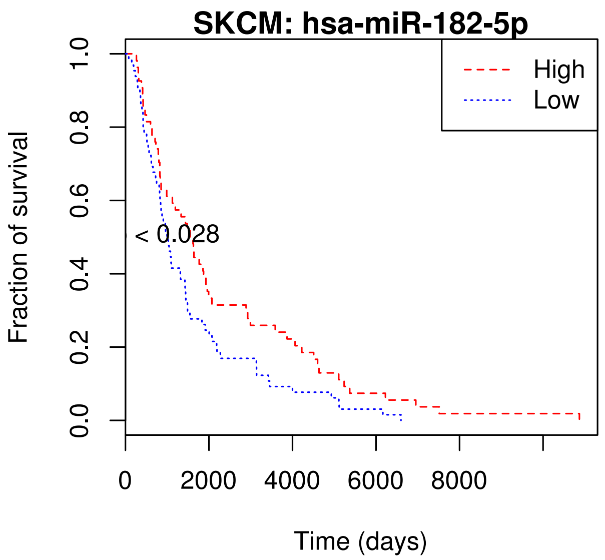
SKCM: hsa-miR-182-5p
High
Low
< 0.028
Fraction of survival
Time (days)

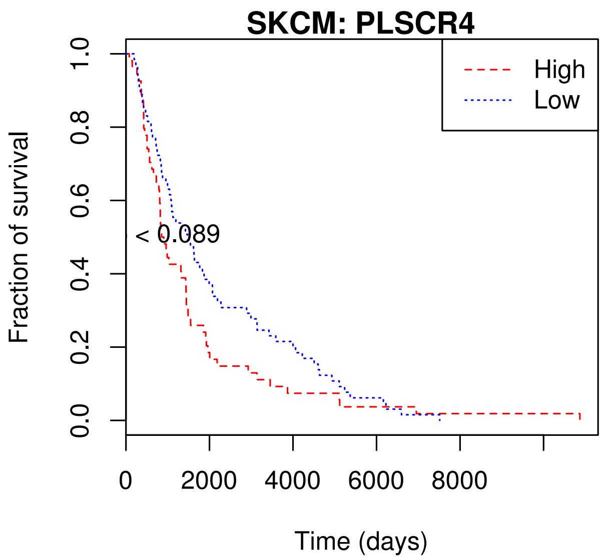
SKCM: PLSCR4
High
Low
< 0.089
Fraction of survival
Time (days)

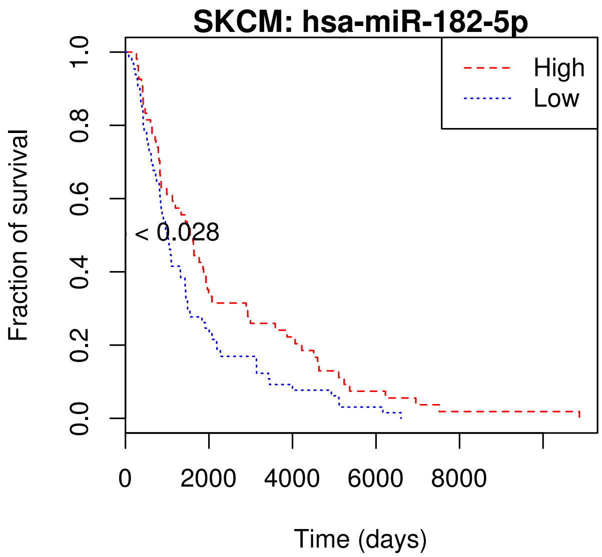
SKCM: hsa-miR-182-5p
High
Low
< 0.028
Fraction of survival
Time (days)
0.0
0.2
0.4
0.6
0.8
1.0
0
2000
4000
6000
8000

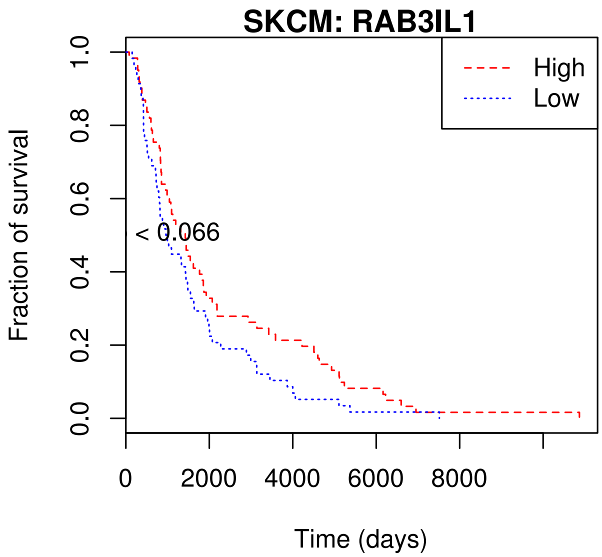
SKCM: RAB3IL1
High
Low
< 0.066
Fraction of survival
Time (days)
0.0
0.2
0.4
0.6
0.8
1.0
0
2000
4000
6000
8000

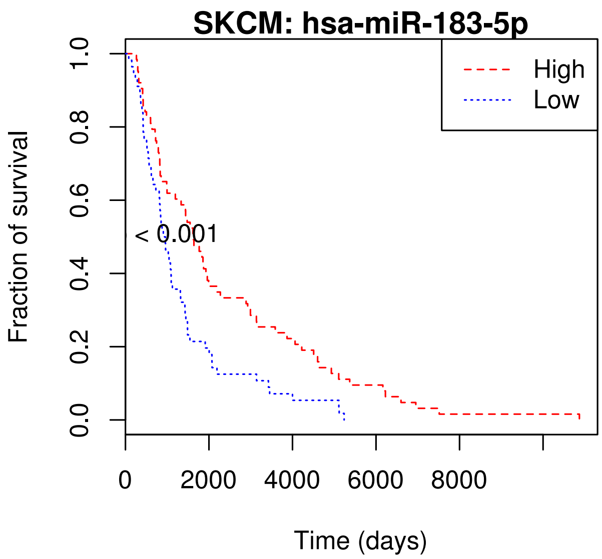
SKCM: hsa-miR-183-5p
High
Low
< 0.001
Fraction of survival
Time (days)
0.0
0.2
0.4
0.6
0.8
1.0
0
2000
4000
6000
8000

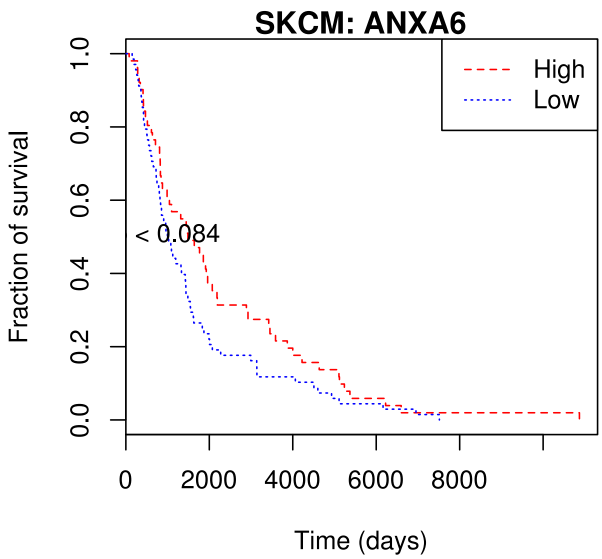
SKCM: ANXA6
High
Low
< 0.084
Fraction of survival
Time (days)
0.0
0.2
0.4
0.6
0.8
1.0
0
2000
4000
6000
8000

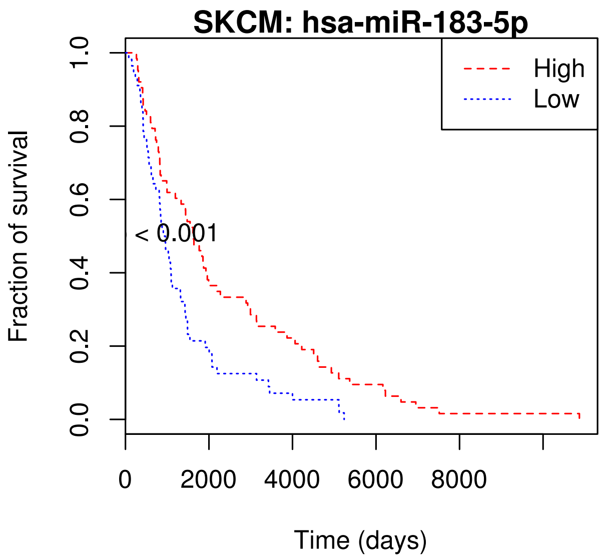
SKCM: hsa-miR-183-5p
High
Low
< 0.001
Fraction of survival
Time (days)
0.0
0.2
0.4
0.6
0.8
1.0
0
2000
4000
6000
8000

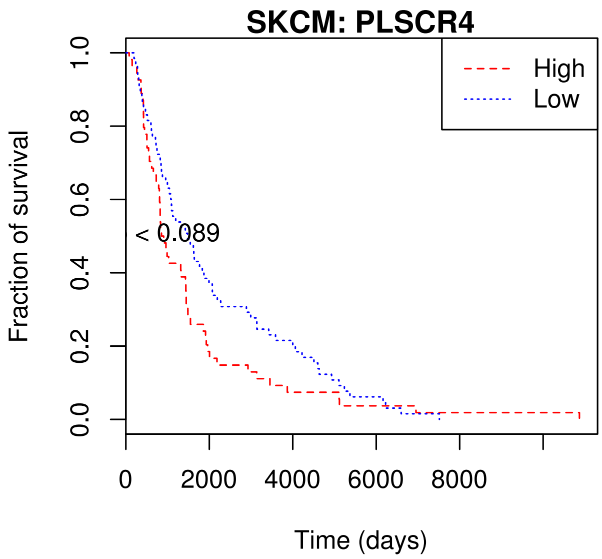
SKCM: PLSCR4
High
Low
< 0.089
Fraction of survival
Time (days)
0.0
0.2
0.4
0.6
0.8
1.0
0
2000
4000
6000
8000

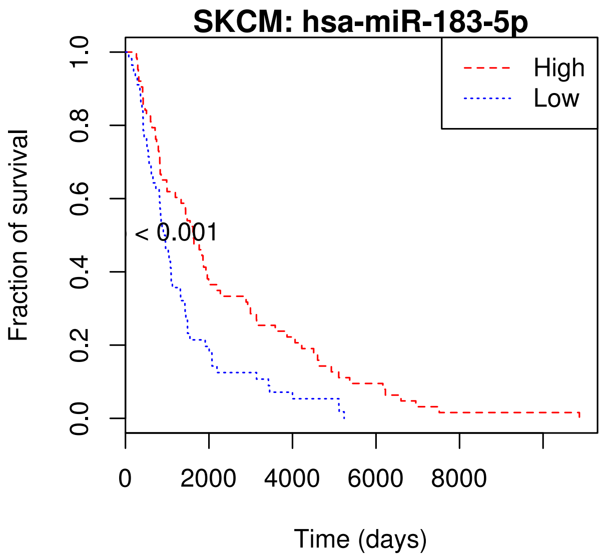
SKCM: hsa-miR-183-5p
Fraction of survival
1.0
0.8
0.6
0.4
0.2
0.0
High
Low
< 0.001
0
2000
4000
6000
8000
Time (days)

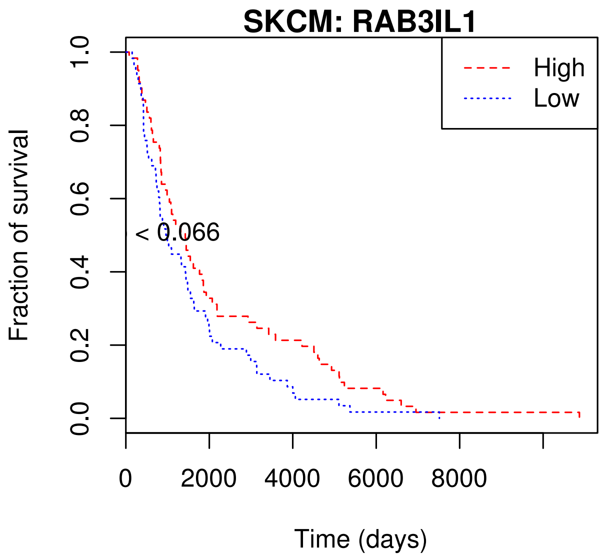
SKCM: RAB3IL1
Fraction of survival
1.0
0.8
0.6
0.4
0.2
0.0
High
Low
< 0.066
0
2000
4000
6000
8000
Time (days)

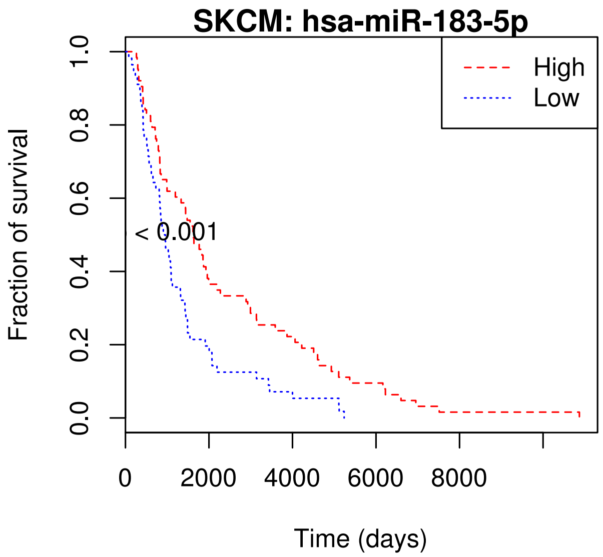
SKCM: hsa-miR-183-5p
Fraction of survival
1.0
0.8
0.6
0.4
0.2
0.0
High
Low
< 0.001
0
2000
4000
6000
8000
Time (days)

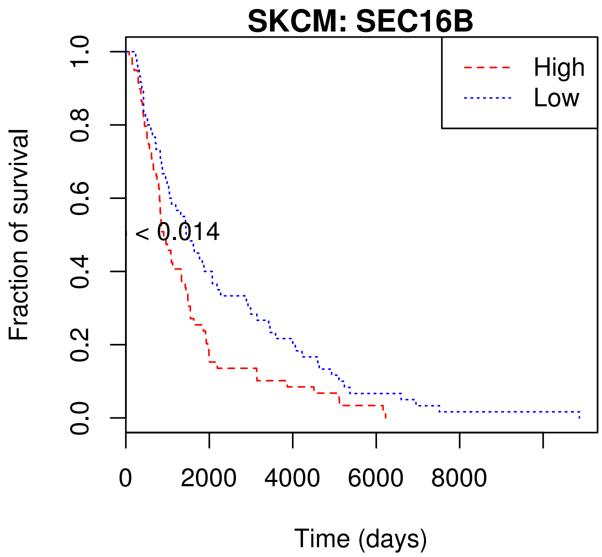
SKCM: SEC16B
Fraction of survival
1.0
0.8
0.6
0.4
0.2
0.0
High
Low
< 0.014
0
2000
4000
6000
8000
Time (days)

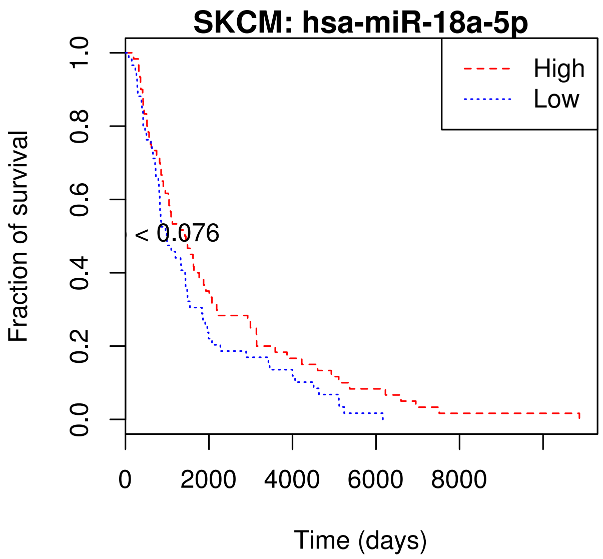
SKCM: hsa-miR-18a-5p
Fraction of survival
1.0
0.8
0.6
0.4
0.2
0.0
High
Low
< 0.076
0
2000
4000
6000
8000
Time (days)

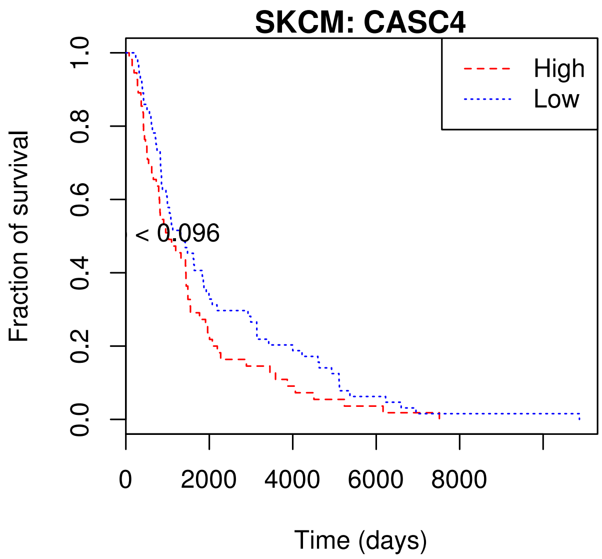
SKCM: CASC4
Fraction of survival
1.0
0.8
0.6
0.4
0.2
0.0
High
Low
< 0.096
0
2000
4000
6000
8000
Time (days)

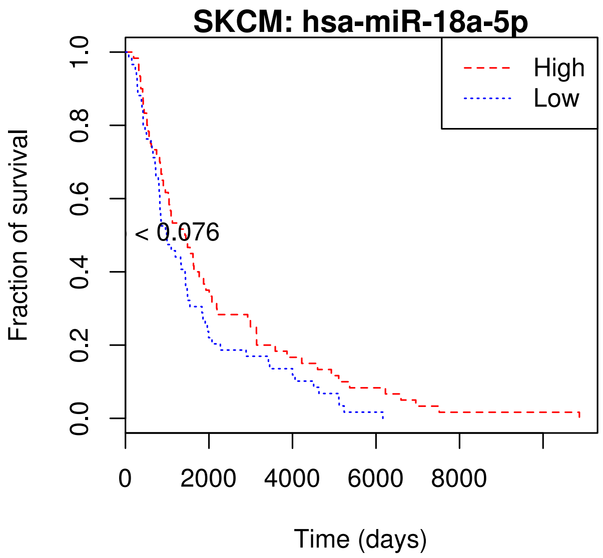
SKCM: hsa-miR-18a-5p
High
Low
< 0.076
Fraction of survival
Time (days)
0 2000 4000 6000 8000
0.0 0.2 0.4 0.6 0.8 1.0

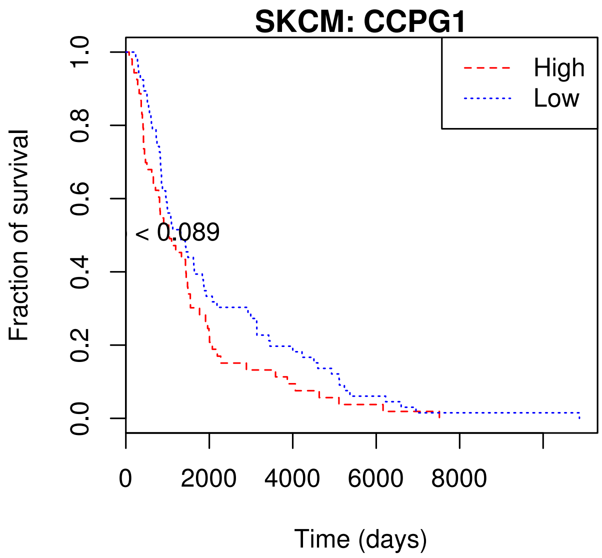
SKCM: CCPG1
High
Low
< 0.089
Fraction of survival
Time (days)
0 2000 4000 6000 8000
0.0 0.2 0.4 0.6 0.8 1.0

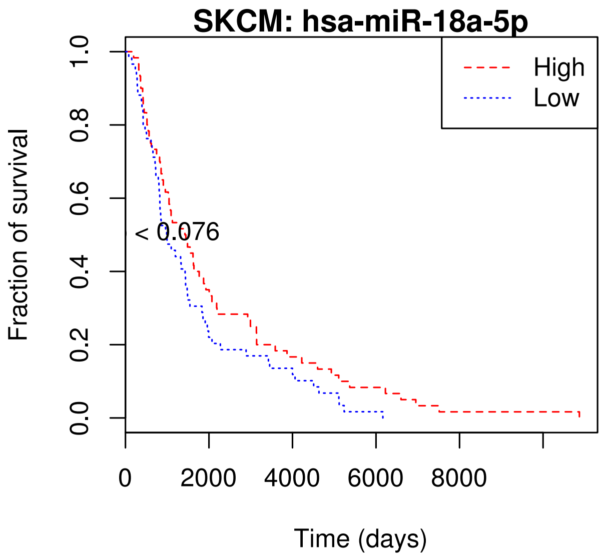
SKCM: hsa-miR-18a-5p
High
Low
< 0.076
Fraction of survival
Time (days)
0 2000 4000 6000 8000
0.0 0.2 0.4 0.6 0.8 1.0

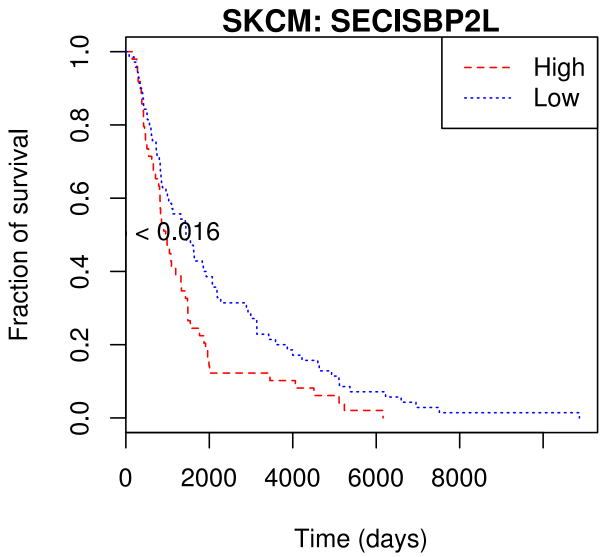
SKCM: SECISBP2L
High
Low
< 0.016
Fraction of survival
Time (days)
0 2000 4000 6000 8000
0.0 0.2 0.4 0.6 0.8 1.0

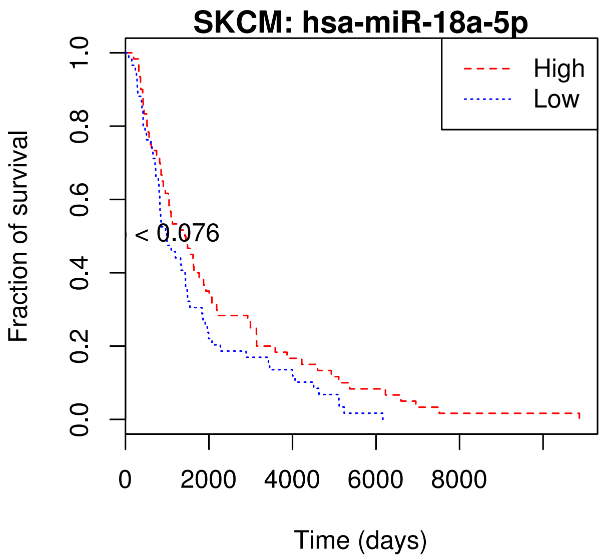
SKCM: hsa-miR-18a-5p
High
Low
< 0.076
Fraction of survival
Time (days)
0 2000 4000 6000 8000
0.0 0.2 0.4 0.6 0.8 1.0

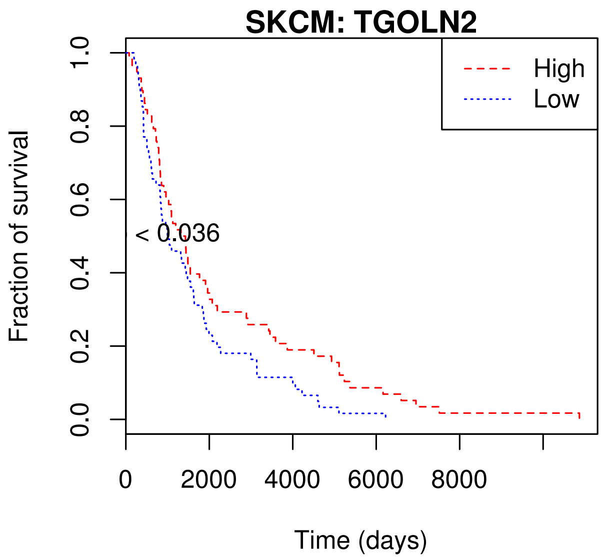
SKCM: TGOLN2
High
Low
< 0.036
Fraction of survival
Time (days)
0 2000 4000 6000 8000
0.0 0.2 0.4 0.6 0.8 1.0

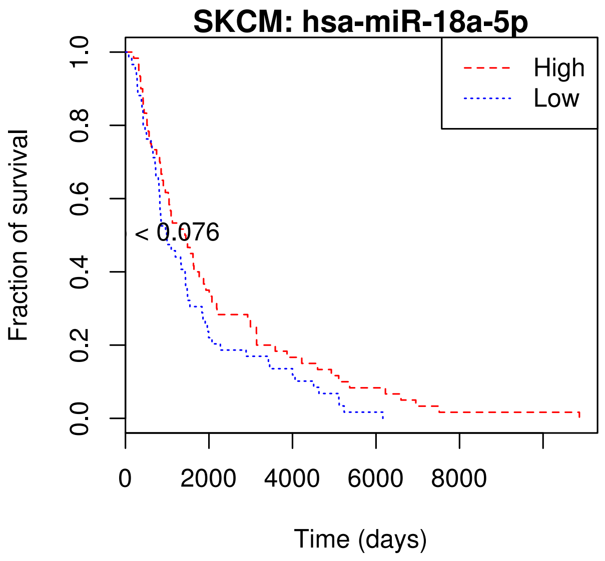
SKCM: hsa-miR-18a-5p
High
Low
< 0.076
Fraction of survival
0.0 0.2 0.4 0.6 0.8 1.0
0 2000 4000 6000 8000
Time (days)

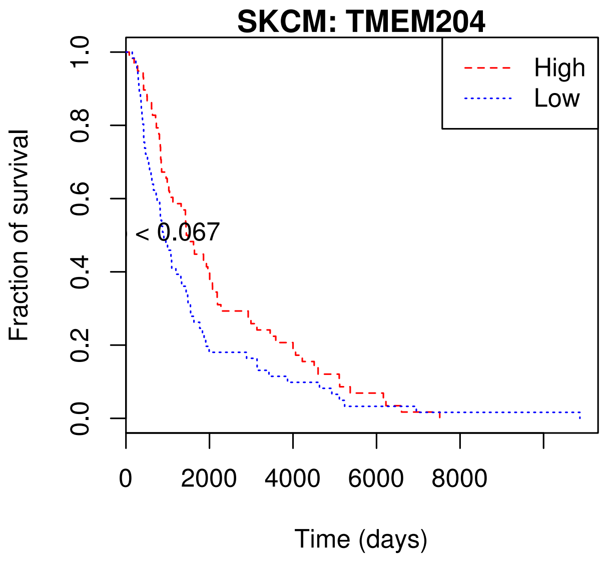
SKCM: TMEM204
High
Low
< 0.067
Fraction of survival
0.0 0.2 0.4 0.6 0.8 1.0
0 2000 4000 6000 8000
Time (days)

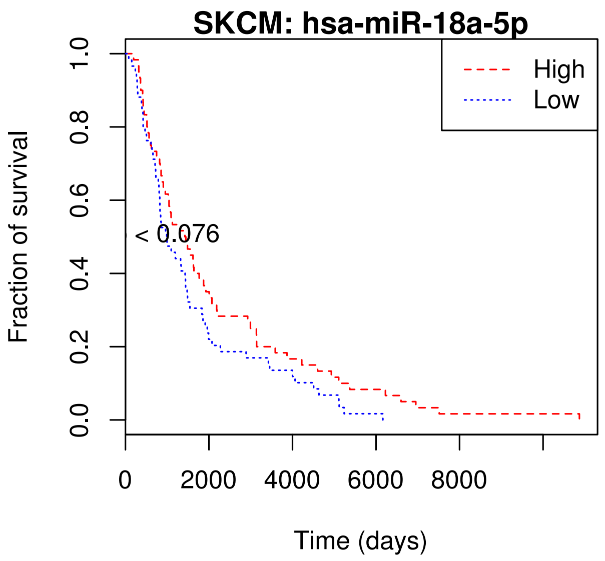
SKCM: hsa-miR-18a-5p
High
Low
< 0.076
Fraction of survival
0.0 0.2 0.4 0.6 0.8 1.0
0 2000 4000 6000 8000
Time (days)

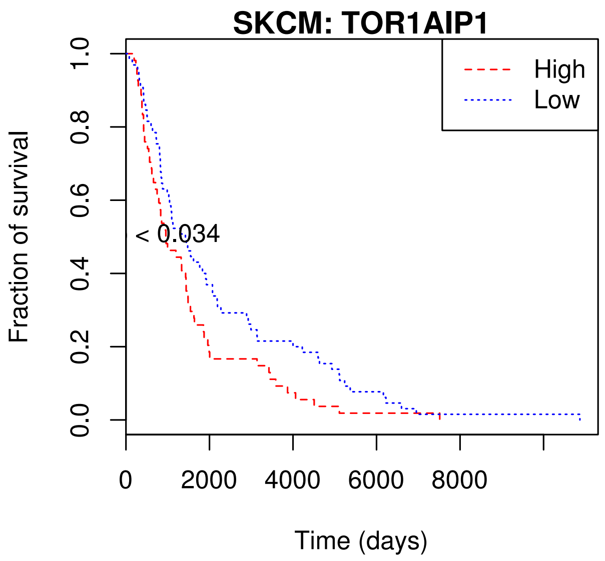
SKCM: TOR1AIP1
High
Low
< 0.034
Fraction of survival
0.0 0.2 0.4 0.6 0.8 1.0
0 2000 4000 6000 8000
Time (days)

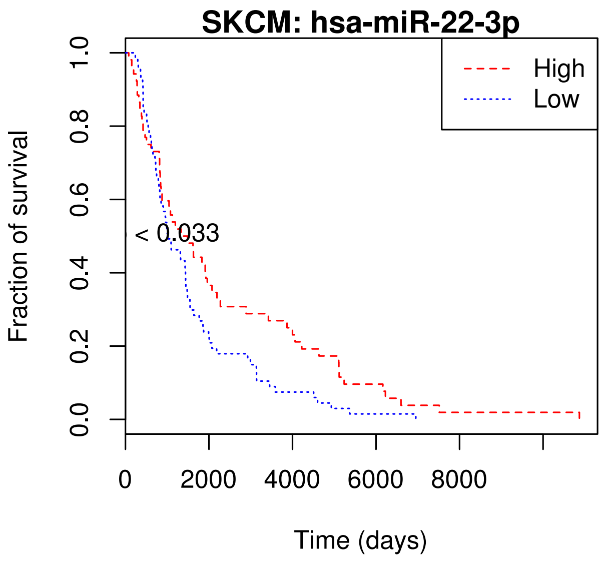
SKCM: hsa-miR-22-3p
High
Low
< 0.033
Fraction of survival
0.0 0.2 0.4 0.6 0.8 1.0
0 2000 4000 6000 8000
Time (days)

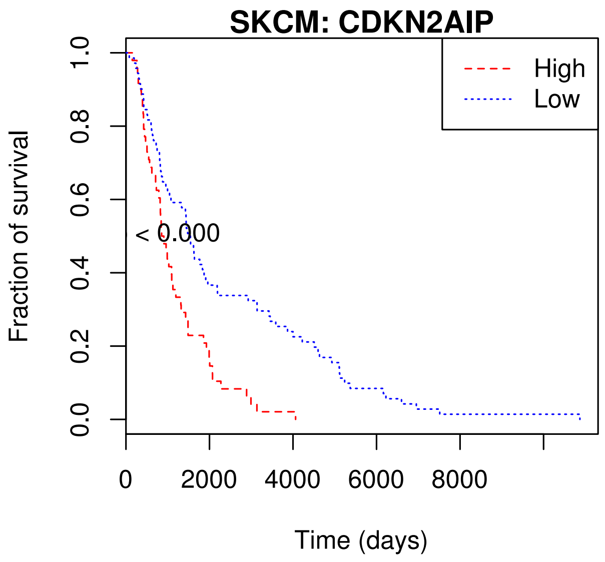
SKCM: CDKN2AIP
High
Low
< 0.000
Fraction of survival
0.0 0.2 0.4 0.6 0.8 1.0
0 2000 4000 6000 8000
Time (days)

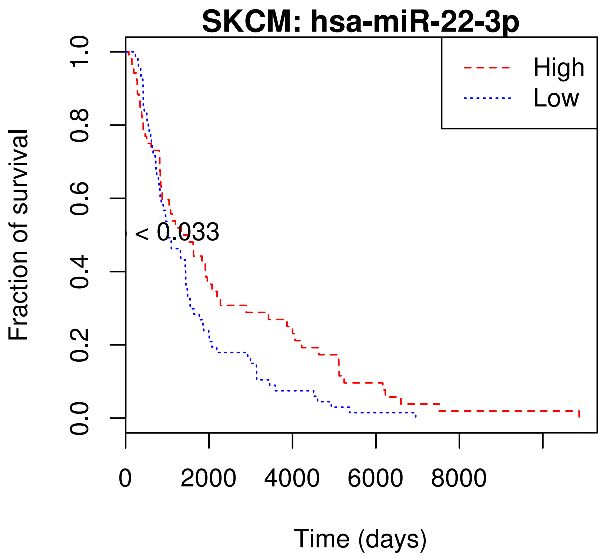
SKCM: hsa-miR-22-3p
High
Low
< 0.033
Fraction of survival
0.0 0.2 0.4 0.6 0.8 1.0
0 2000 4000 6000 8000
Time (days)

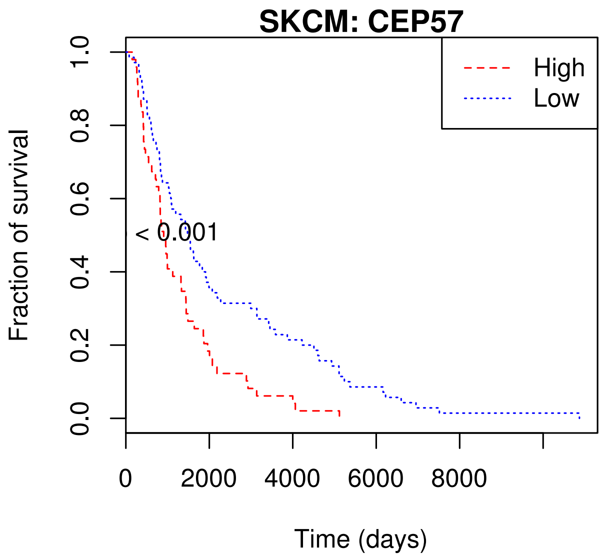
SKCM: CEP57
High
Low
< 0.001
Fraction of survival
0.0 0.2 0.4 0.6 0.8 1.0
0 2000 4000 6000 8000
Time (days)

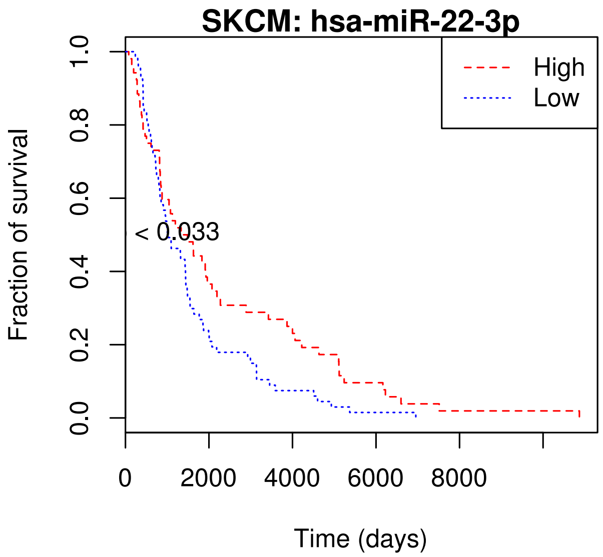
SKCM: hsa-miR-22-3p
High
Low
< 0.033
Fraction of survival
0.0 0.2 0.4 0.6 0.8 1.0
0 2000 4000 6000 8000
Time (days)

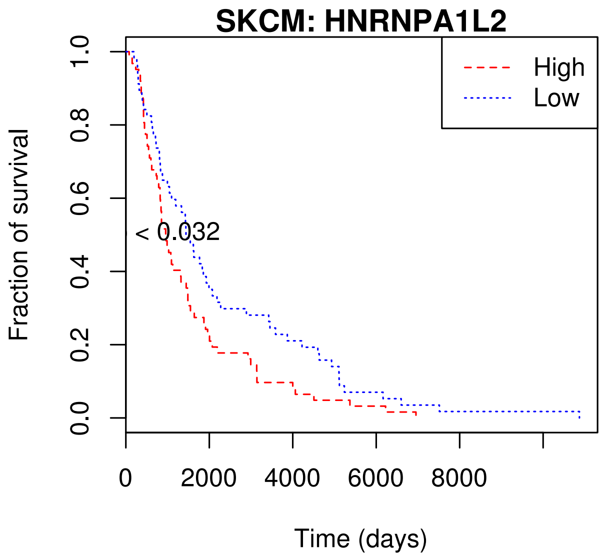
SKCM: HNRNPA1L2
High
Low
< 0.032
Fraction of survival
0.0 0.2 0.4 0.6 0.8 1.0
0 2000 4000 6000 8000
Time (days)

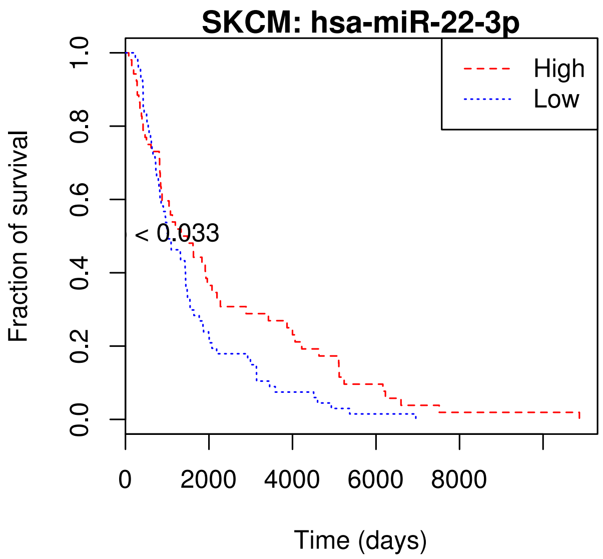
SKCM: hsa-miR-22-3p
High
Low
< 0.033
Fraction of survival
0.0 0.2 0.4 0.6 0.8 1.0
0 2000 4000 6000 8000
Time (days)

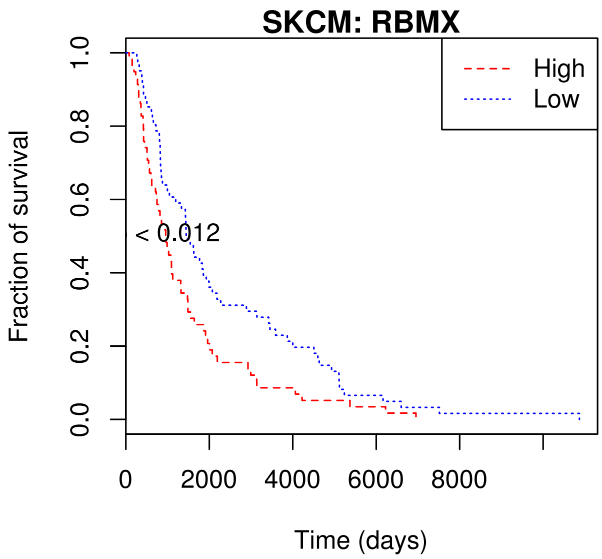
SKCM: RBMX
High
Low
< 0.012
Fraction of survival
0.0 0.2 0.4 0.6 0.8 1.0
0 2000 4000 6000 8000
Time (days)

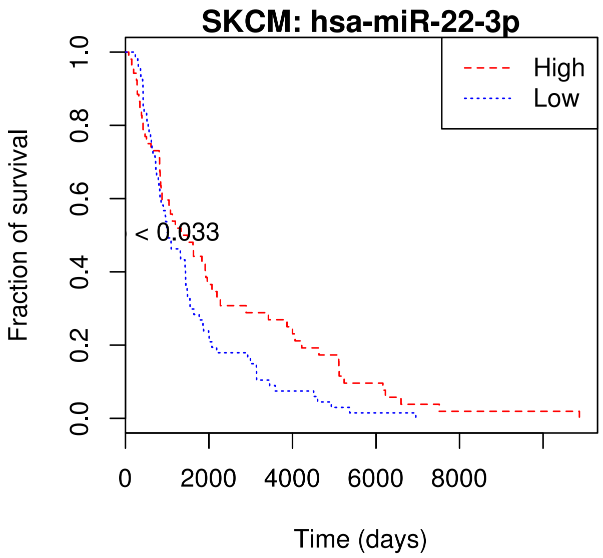
SKCM: hsa-miR-22-3p
High
Low
< 0.033
Fraction of survival
0.0
0.2
0.4
0.6
0.8
1.0
0
2000
4000
6000
8000
Time (days)

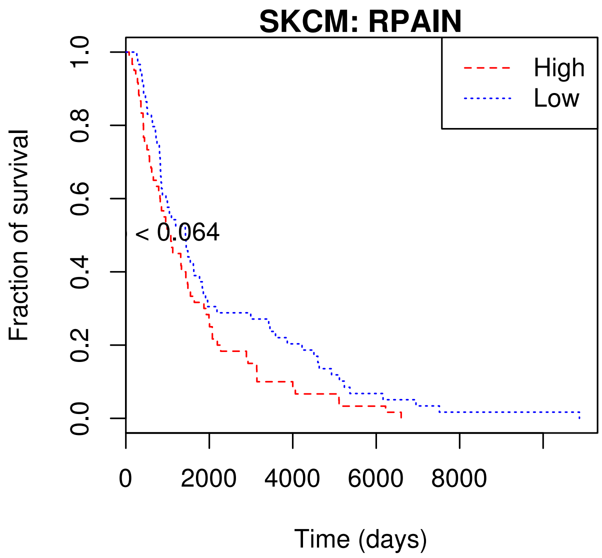
SKCM: RPAIN
High
Low
< 0.064
Fraction of survival
0.0
0.2
0.4
0.6
0.8
1.0
0
2000
4000
6000
8000
Time (days)

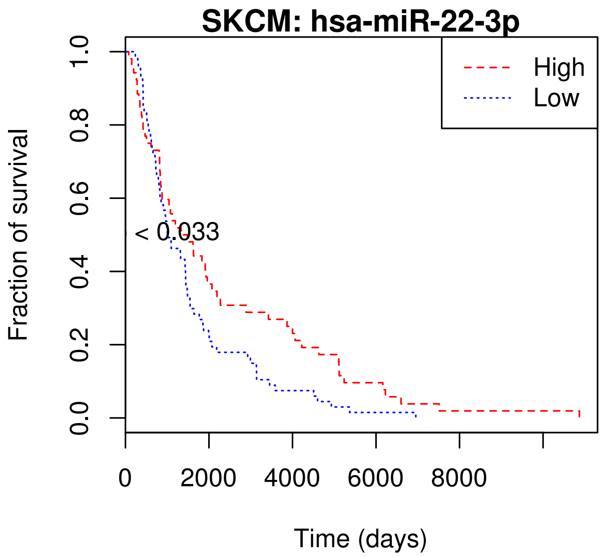
SKCM: hsa-miR-22-3p
High
Low
< 0.033
Fraction of survival
0.0
0.2
0.4
0.6
0.8
1.0
0
2000
4000
6000
8000
Time (days)

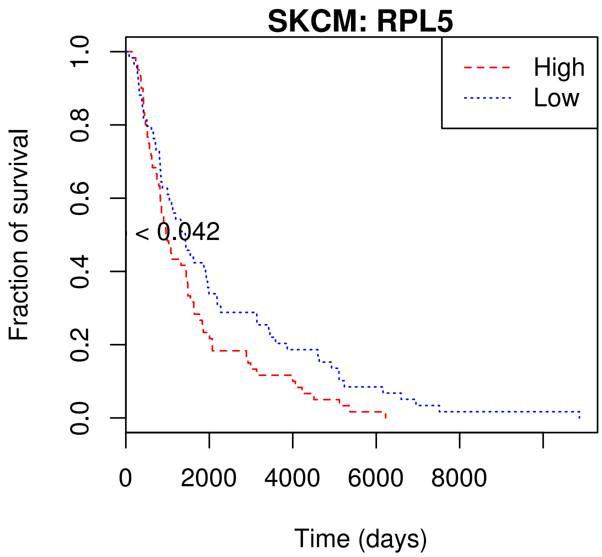
SKCM: RPL5
High
Low
< 0.042
Fraction of survival
0.0
0.2
0.4
0.6
0.8
1.0
0
2000
4000
6000
8000
Time (days)

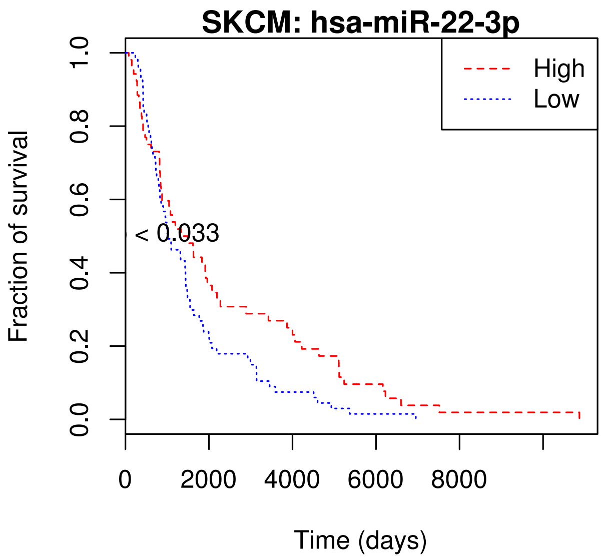
SKCM: hsa-miR-22-3p
High
Low
< 0.033
Fraction of survival
0.0
0.2
0.4
0.6
0.8
1.0
0
2000
4000
6000
8000
Time (days)

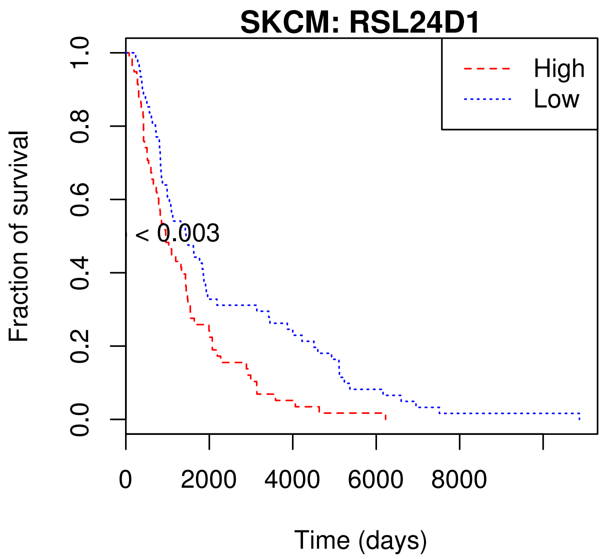
SKCM: RSL24D1
High
Low
< 0.003
Fraction of survival
0.0
0.2
0.4
0.6
0.8
1.0
0
2000
4000
6000
8000
Time (days)

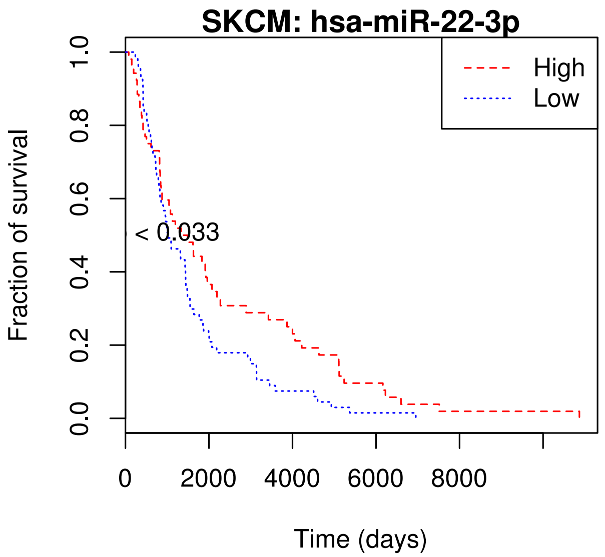
SKCM: hsa-miR-22-3p
High
Low
< 0.033
Fraction of survival
0.0
0.2
0.4
0.6
0.8
1.0
0
2000
4000
6000
8000
Time (days)

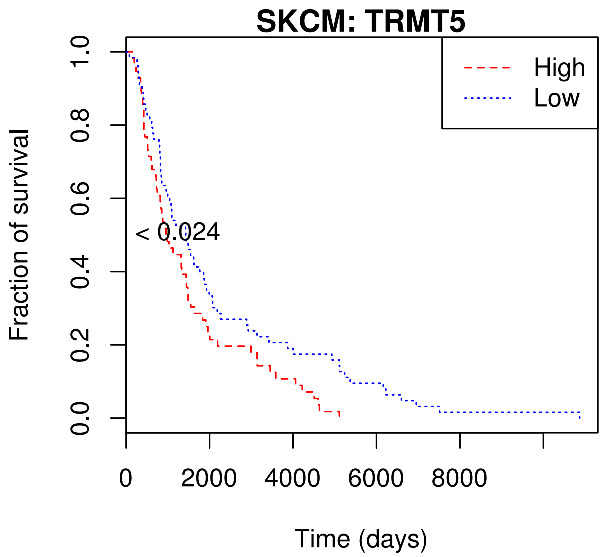
SKCM: TRMT5
High
Low
< 0.024
Fraction of survival
0.0
0.2
0.4
0.6
0.8
1.0
0
2000
4000
6000
8000
Time (days)

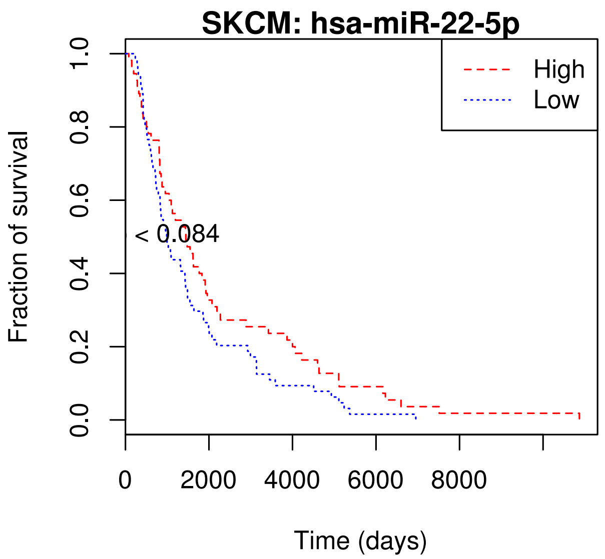
SKCM: hsa-miR-22-5p
High
Low
< 0.084
Fraction of survival
0.0
0.2
0.4
0.6
0.8
1.0
0
2000
4000
6000
8000
Time (days)

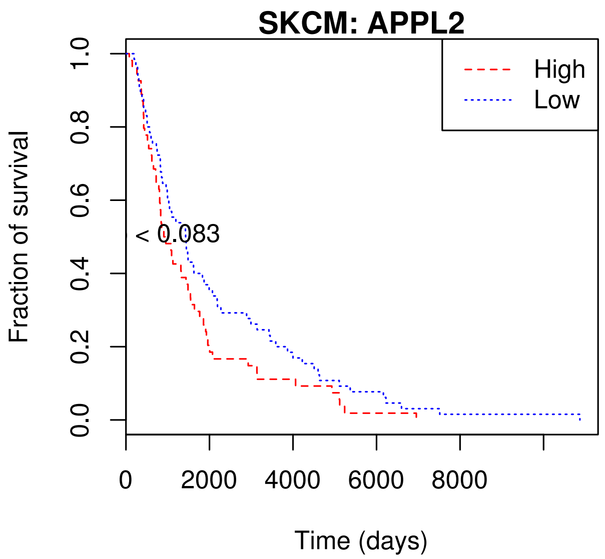
SKCM: APPL2
High
Low
< 0.083
Fraction of survival
0.0
0.2
0.4
0.6
0.8
1.0
0
2000
4000
6000
8000
Time (days)

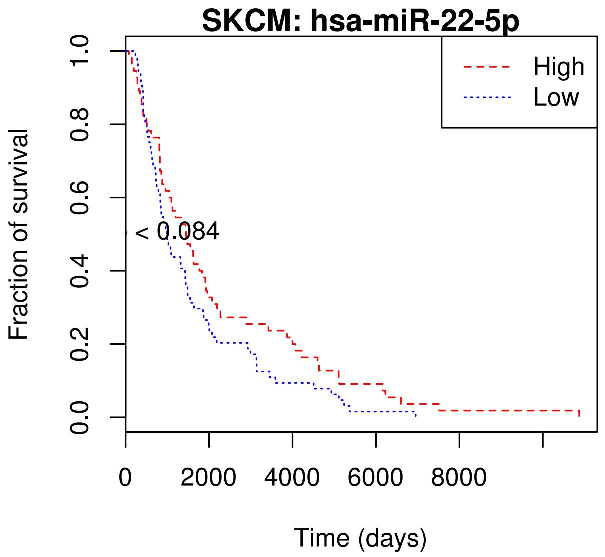
SKCM: hsa-miR-22-5p
High
Low
< 0.084
Fraction of survival
0.0
0.2
0.4
0.6
0.8
1.0
0
2000
4000
6000
8000
Time (days)

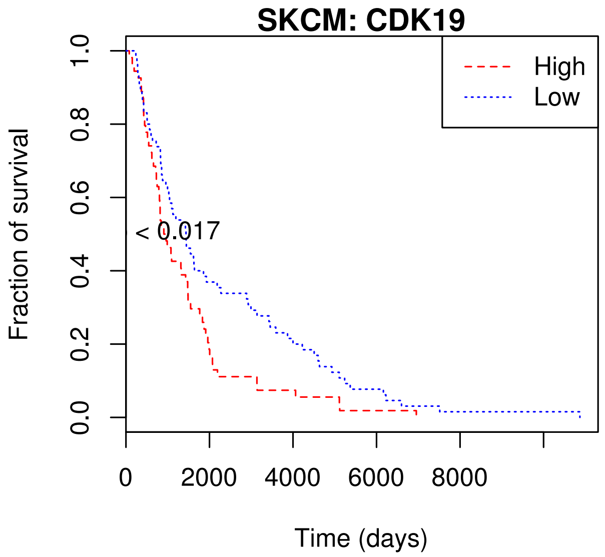
SKCM: CDK19
High
Low
< 0.017
Fraction of survival
0.0
0.2
0.4
0.6
0.8
1.0
0
2000
4000
6000
8000
Time (days)

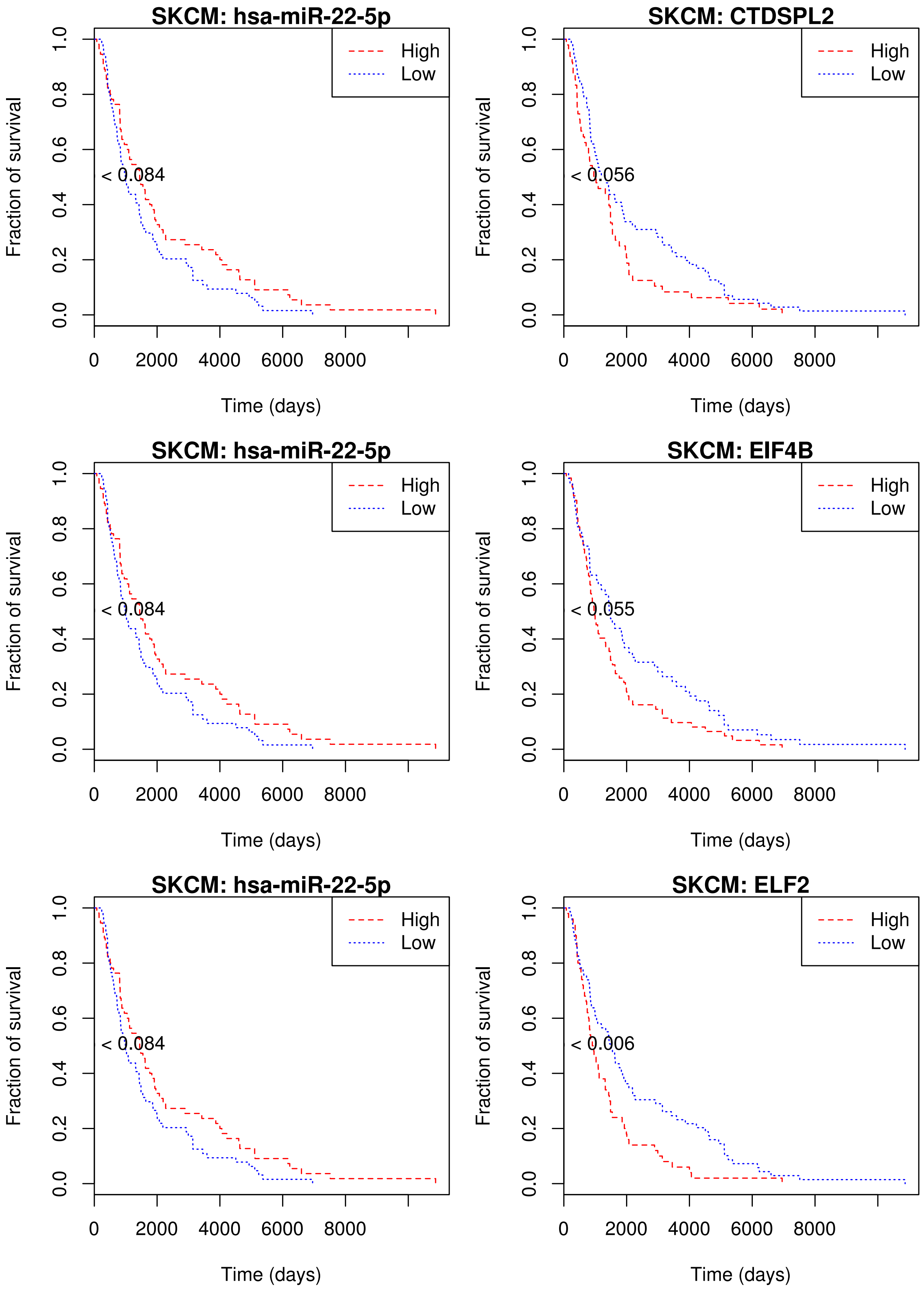

SKCM: hsa-miR-22-5p
High
Low
< 0.084
Fraction of survival
Time (days)
SKCM: CTDSPL2
High
Low
< 0.056
Fraction of survival
Time (days)
SKCM: hsa-miR-22-5p
High
Low
< 0.084
Fraction of survival
Time (days)
SKCM: EIF4B
High
Low
< 0.055
Fraction of survival
Time (days)
SKCM: hsa-miR-22-5p
High
Low
< 0.084
Fraction of survival
Time (days)
SKCM: ELF2
High
Low
< 0.006
Fraction of survival
Time (days)

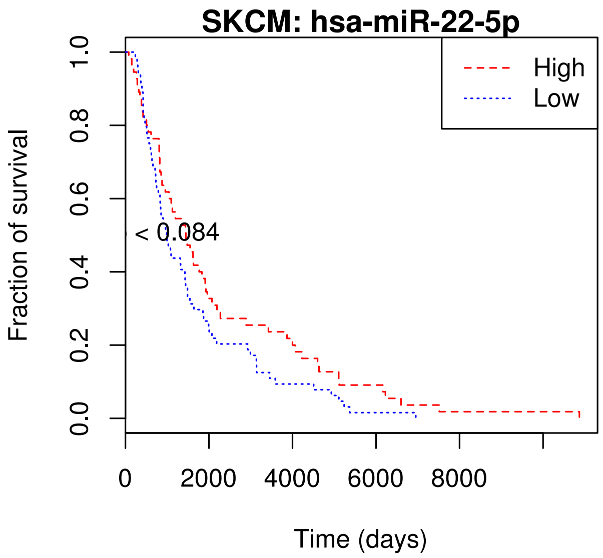
SKCM: hsa-miR-22-5p
High
Low
< 0.084
Fraction of survival
0.0 0.2 0.4 0.6 0.8 1.0
0 2000 4000 6000 8000
Time (days)

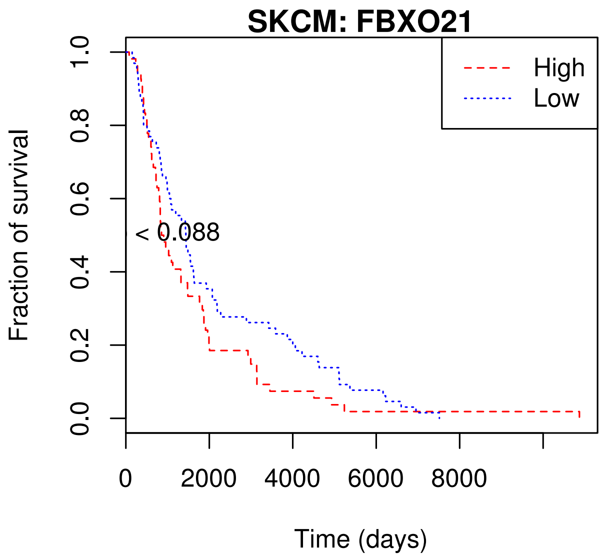
SKCM: FBXO21
High
Low
< 0.088
Fraction of survival
0.0 0.2 0.4 0.6 0.8 1.0
0 2000 4000 6000 8000
Time (days)

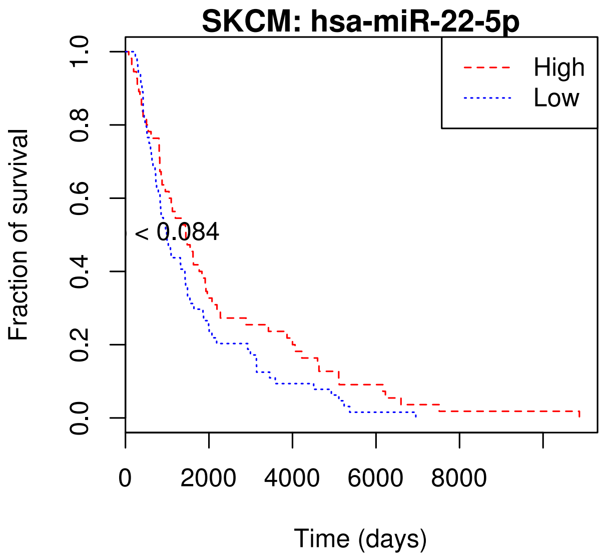
SKCM: hsa-miR-22-5p
High
Low
< 0.084
Fraction of survival
0.0 0.2 0.4 0.6 0.8 1.0
0 2000 4000 6000 8000
Time (days)

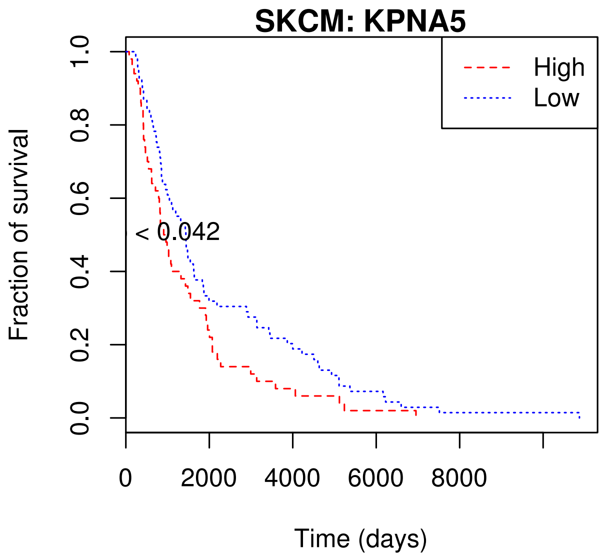
SKCM: KPNA5
High
Low
< 0.042
Fraction of survival
0.0 0.2 0.4 0.6 0.8 1.0
0 2000 4000 6000 8000
Time (days)

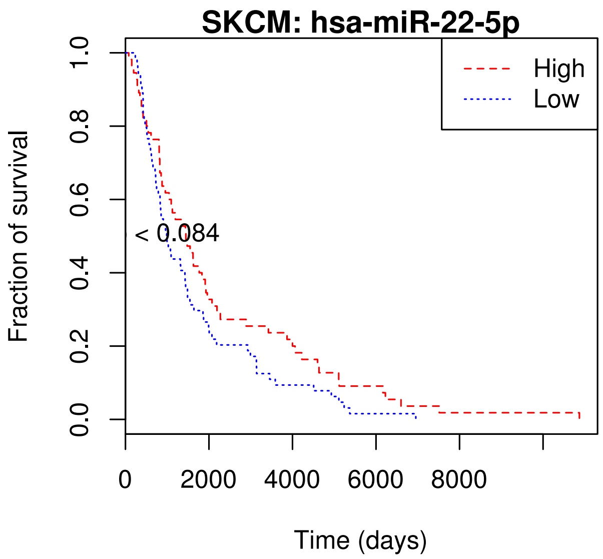
SKCM: hsa-miR-22-5p
High
Low
< 0.084
Fraction of survival
0.0 0.2 0.4 0.6 0.8 1.0
0 2000 4000 6000 8000
Time (days)

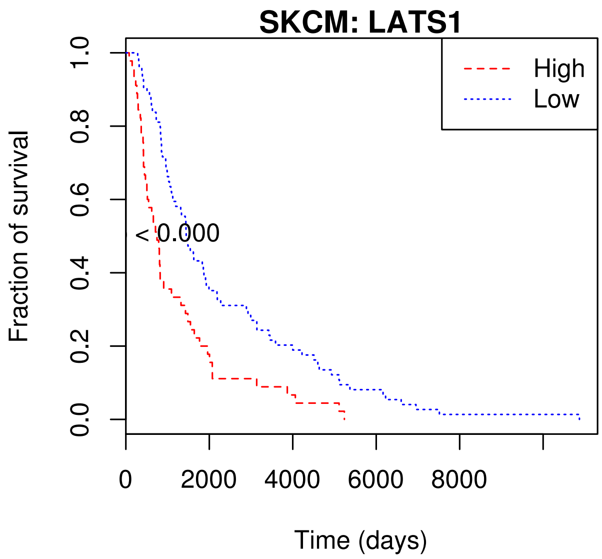
SKCM: LATS1
High
Low
< 0.000
Fraction of survival
0.0 0.2 0.4 0.6 0.8 1.0
0 2000 4000 6000 8000
Time (days)

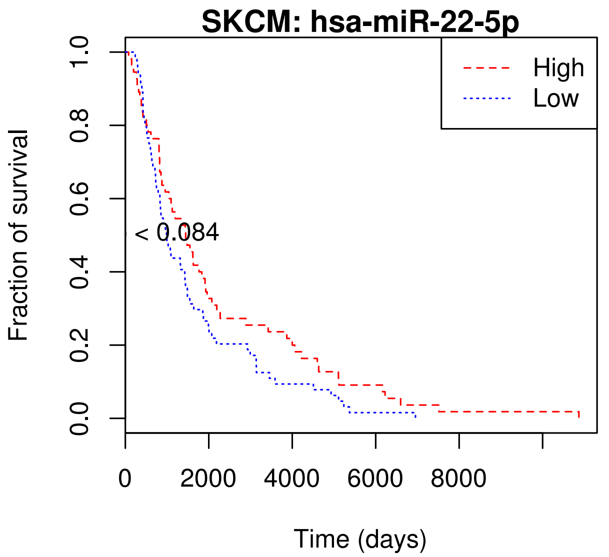
SKCM: hsa-miR-22-5p
High
Low
< 0.084
Fraction of survival
0.0 0.2 0.4 0.6 0.8 1.0
0 2000 4000 6000 8000
Time (days)

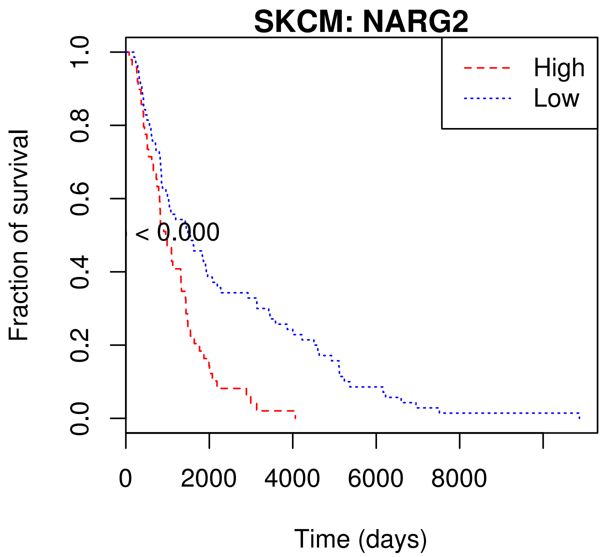
SKCM: NARG2
High
Low
< 0.000
Fraction of survival
0.0 0.2 0.4 0.6 0.8 1.0
0 2000 4000 6000 8000
Time (days)

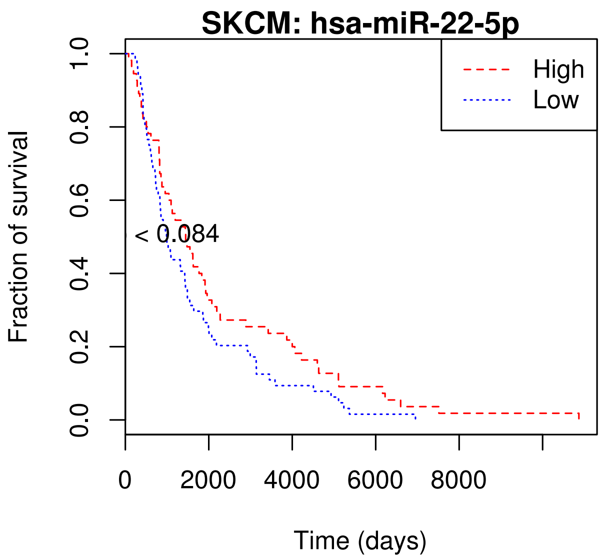
SKCM: hsa-miR-22-5p
High
Low
< 0.084
Fraction of survival
0.0 0.2 0.4 0.6 0.8 1.0
0 2000 4000 6000 8000
Time (days)

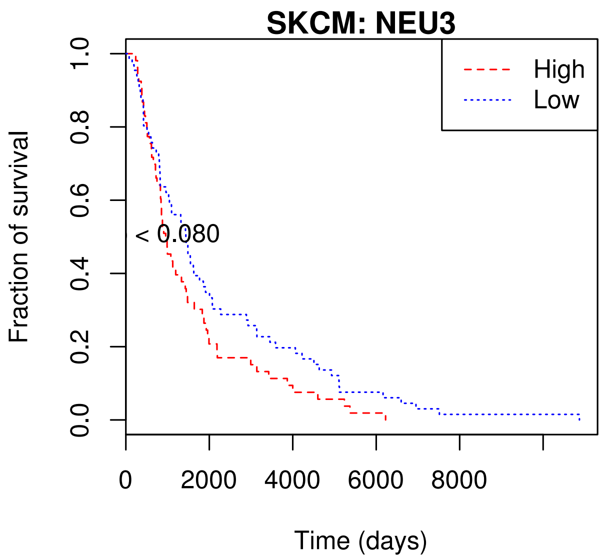
SKCM: NEU3
High
Low
< 0.080
Fraction of survival
0.0 0.2 0.4 0.6 0.8 1.0
0 2000 4000 6000 8000
Time (days)

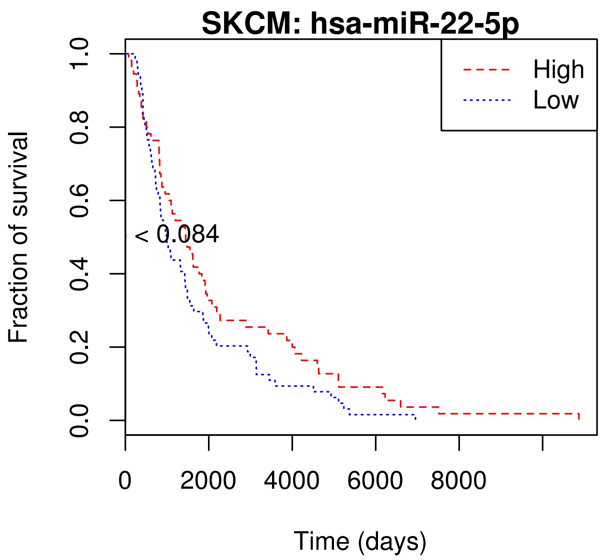
SKCM: hsa-miR-22-5p
High
Low
< 0.084
Fraction of survival
0.0 0.2 0.4 0.6 0.8 1.0
0 2000 4000 6000 8000
Time (days)

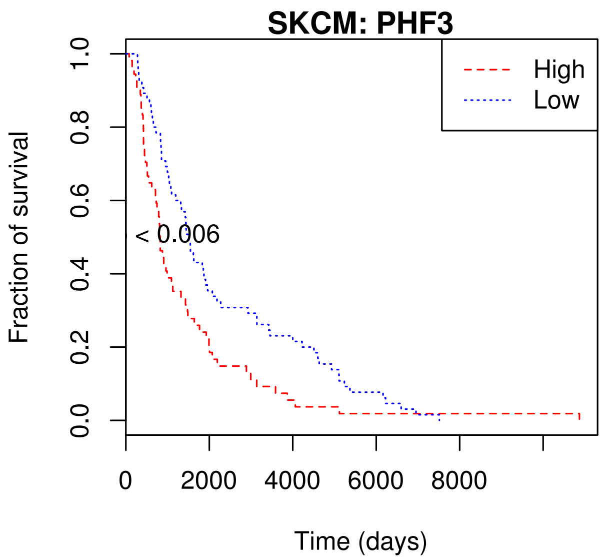
SKCM: PHF3
High
Low
< 0.006
Fraction of survival
0.0 0.2 0.4 0.6 0.8 1.0
0 2000 4000 6000 8000
Time (days)

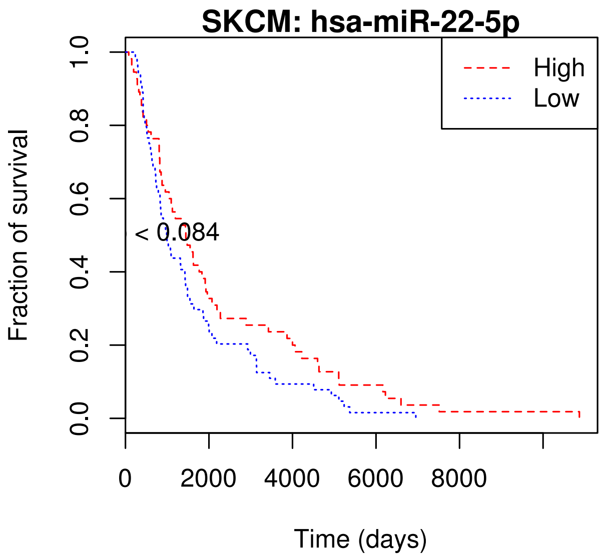
SKCM: hsa-miR-22-5p
High
Low
< 0.084
Fraction of survival
0.0
0.2
0.4
0.6
0.8
1.0
0
2000
4000
6000
8000
Time (days)

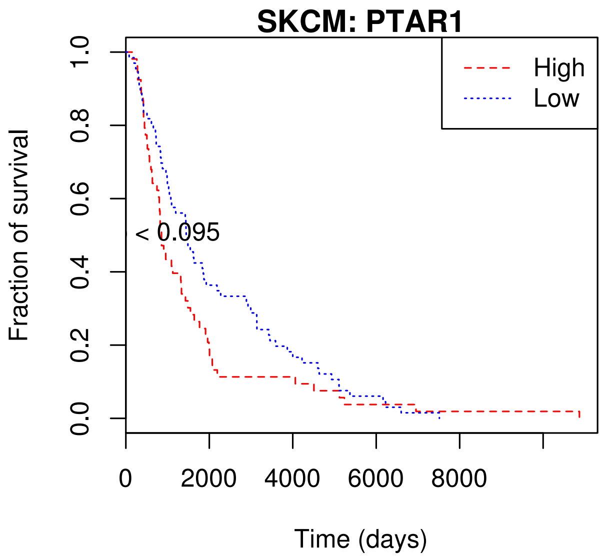
SKCM: PTAR1
High
Low
< 0.095
Fraction of survival
0.0
0.2
0.4
0.6
0.8
1.0
0
2000
4000
6000
8000
Time (days)

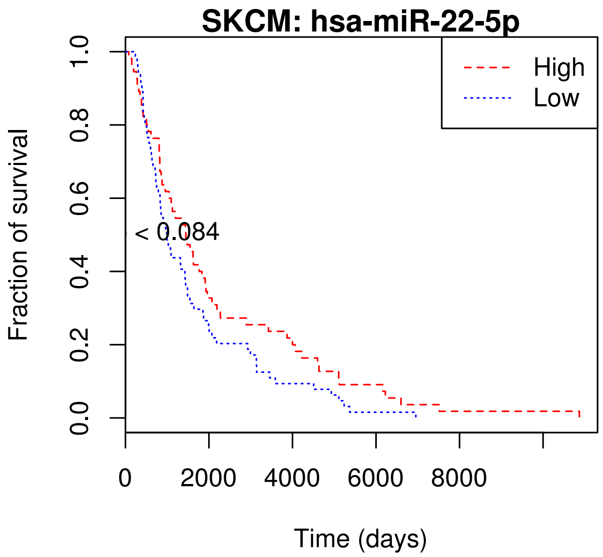
SKCM: hsa-miR-22-5p
High
Low
< 0.084
Fraction of survival
0.0
0.2
0.4
0.6
0.8
1.0
0
2000
4000
6000
8000
Time (days)

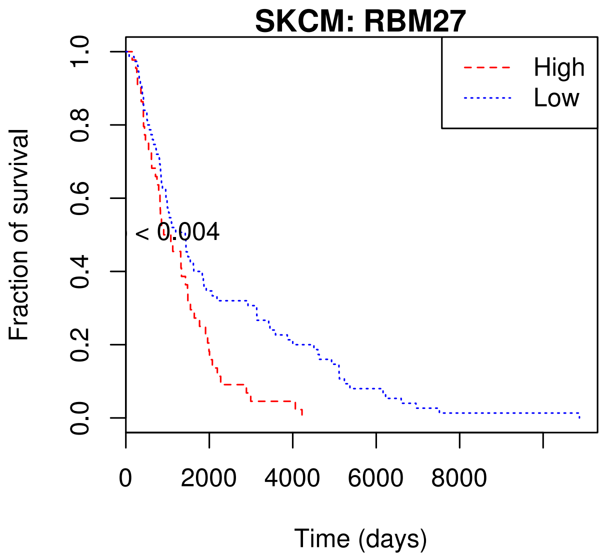
SKCM: RBM27
High
Low
< 0.004
Fraction of survival
0.0
0.2
0.4
0.6
0.8
1.0
0
2000
4000
6000
8000
Time (days)

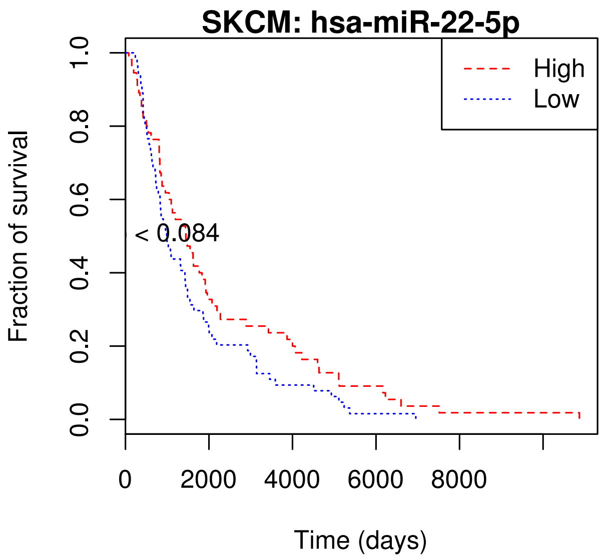
SKCM: hsa-miR-22-5p
High
Low
< 0.084
Fraction of survival
0.0
0.2
0.4
0.6
0.8
1.0
0
2000
4000
6000
8000
Time (days)

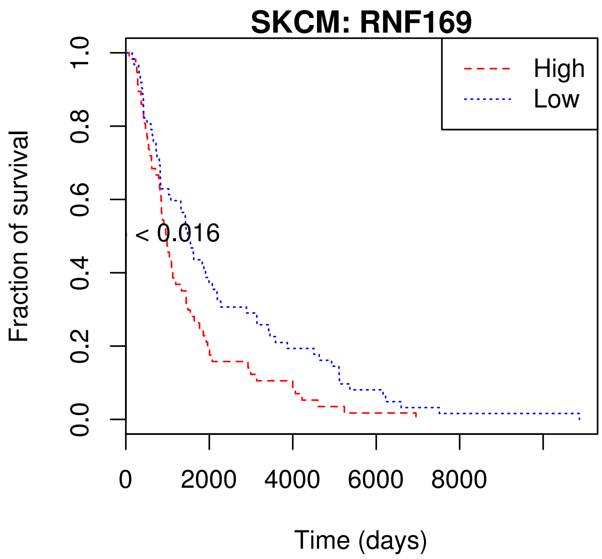
SKCM: RNF169
High
Low
< 0.016
Fraction of survival
0.0
0.2
0.4
0.6
0.8
1.0
0
2000
4000
6000
8000
Time (days)

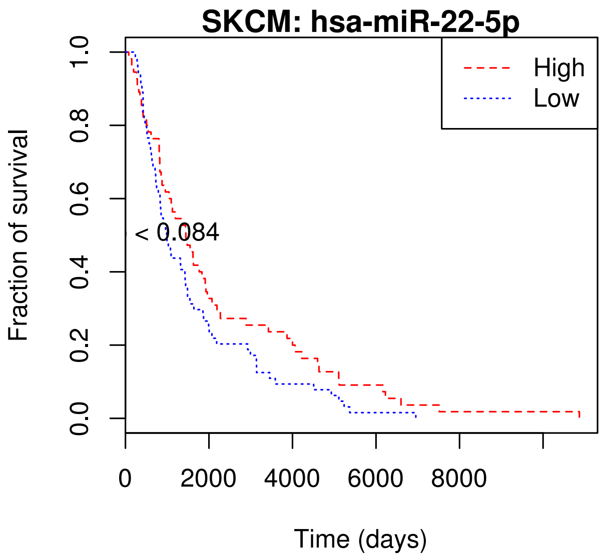
SKCM: hsa-miR-22-5p
High
Low
< 0.084
Fraction of survival
Time (days)

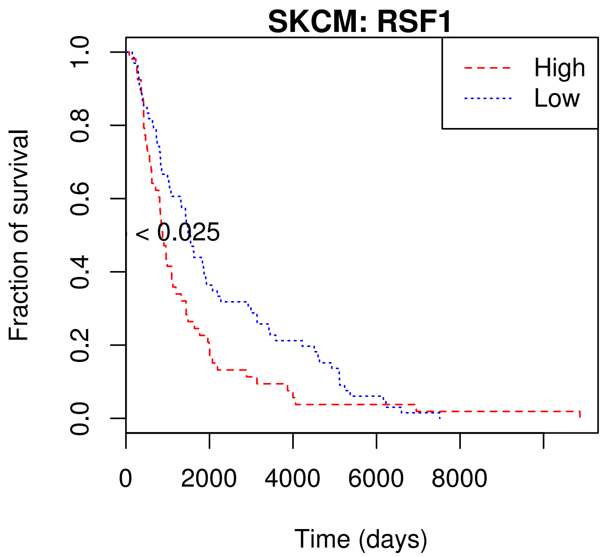
SKCM: RSF1
High
Low
< 0.025
Fraction of survival
Time (days)

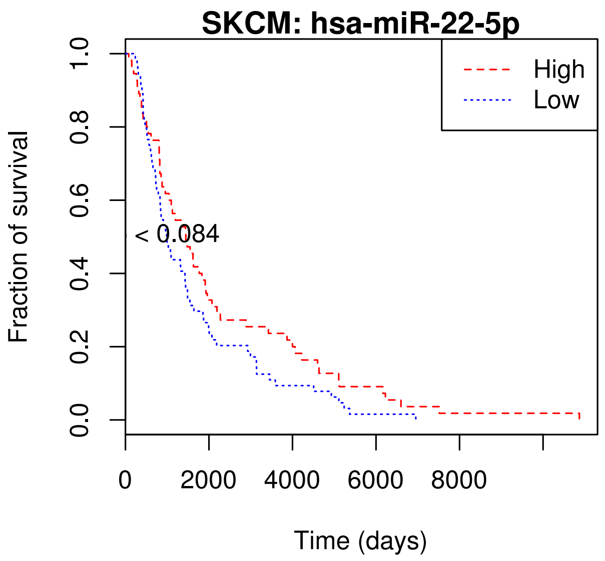
SKCM: hsa-miR-22-5p
High
Low
< 0.084
Fraction of survival
Time (days)

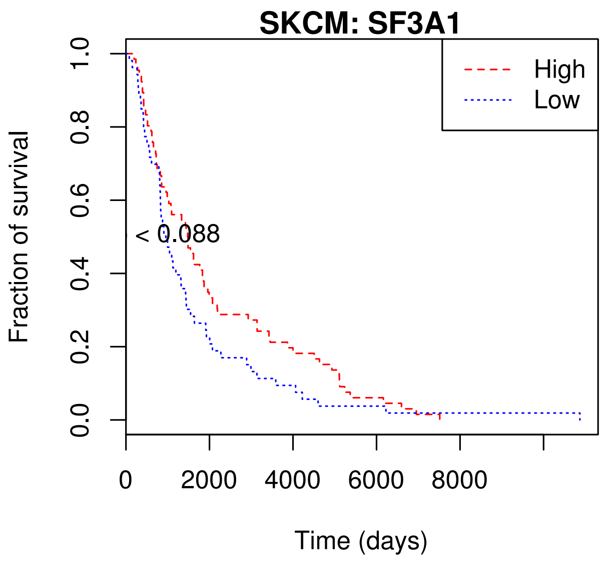
SKCM: SF3A1
High
Low
< 0.088
Fraction of survival
Time (days)

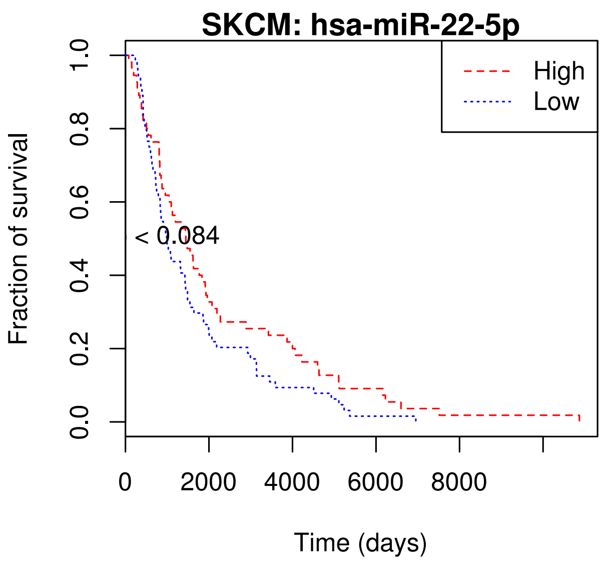
SKCM: hsa-miR-22-5p
High
Low
< 0.084
Fraction of survival
Time (days)

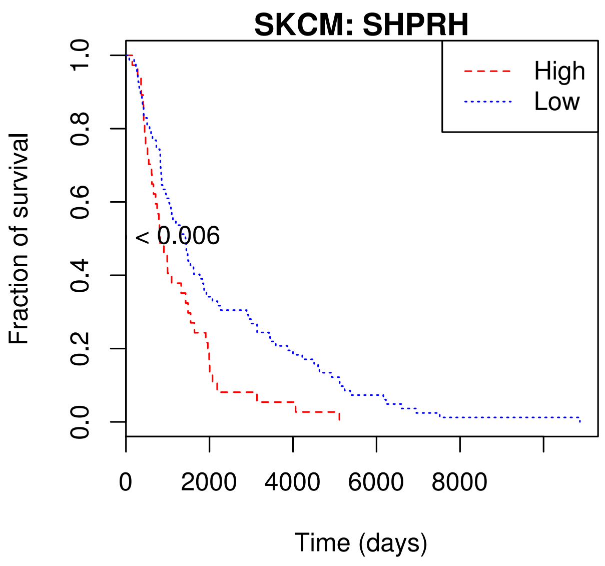
SKCM: SHPRH
High
Low
< 0.006
Fraction of survival
Time (days)

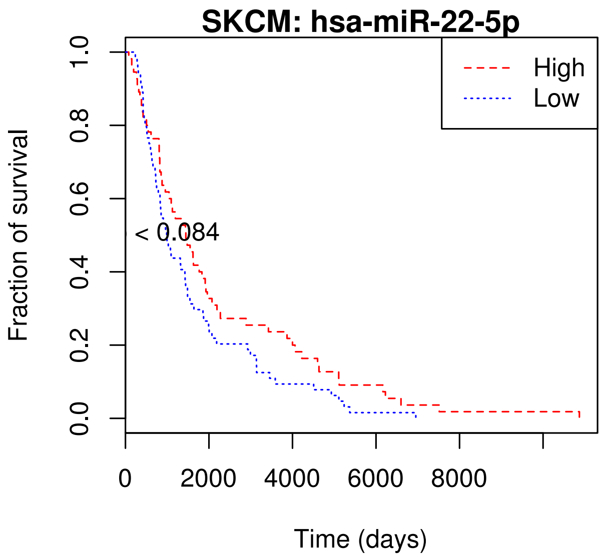
SKCM: hsa-miR-22-5p
High
Low
< 0.084
Fraction of survival
Time (days)
0.0 0.2 0.4 0.6 0.8 1.0
0 2000 4000 6000 8000

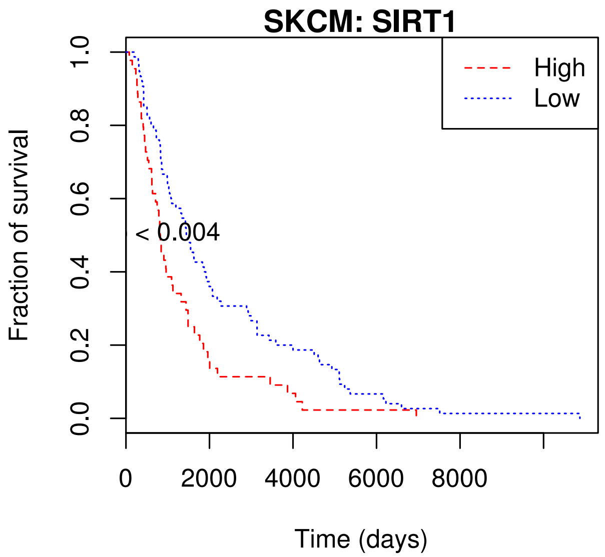
SKCM: SIRT1
High
Low
< 0.004
Fraction of survival
Time (days)
0.0 0.2 0.4 0.6 0.8 1.0
0 2000 4000 6000 8000

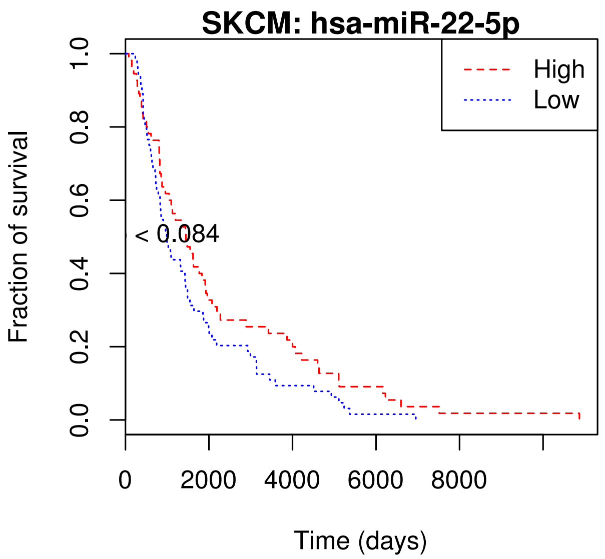
SKCM: hsa-miR-22-5p
High
Low
< 0.084
Fraction of survival
Time (days)
0.0 0.2 0.4 0.6 0.8 1.0
0 2000 4000 6000 8000

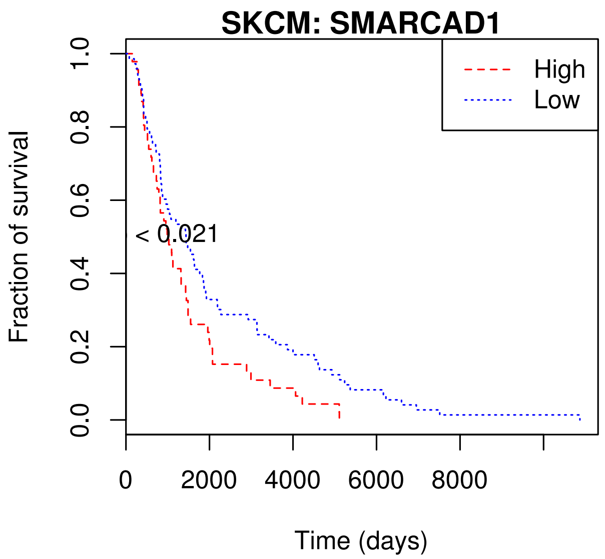
SKCM: SMARCAD1
High
Low
< 0.021
Fraction of survival
Time (days)
0.0 0.2 0.4 0.6 0.8 1.0
0 2000 4000 6000 8000

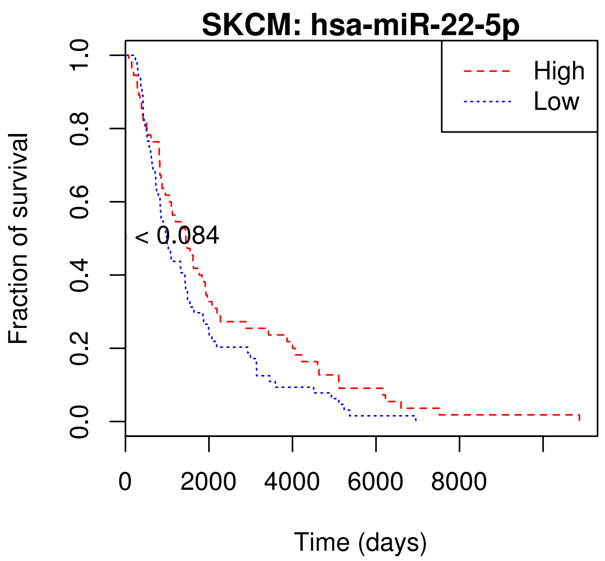
SKCM: hsa-miR-22-5p
High
Low
< 0.084
Fraction of survival
Time (days)
0.0 0.2 0.4 0.6 0.8 1.0
0 2000 4000 6000 8000

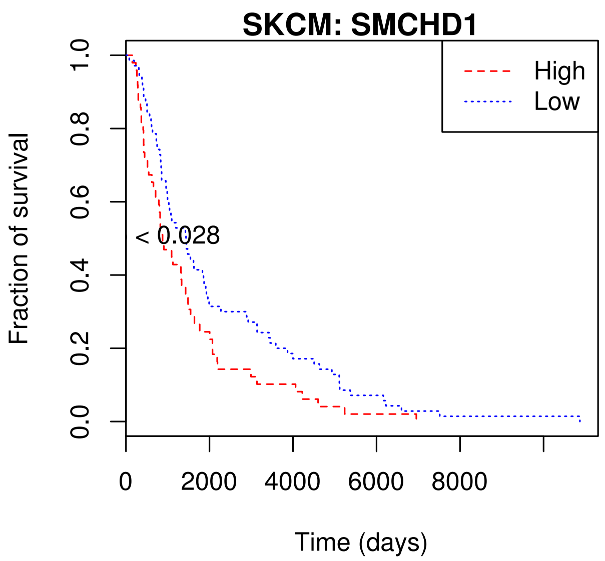
SKCM: SMCHD1
High
Low
< 0.028
Fraction of survival
Time (days)
0.0 0.2 0.4 0.6 0.8 1.0
0 2000 4000 6000 8000

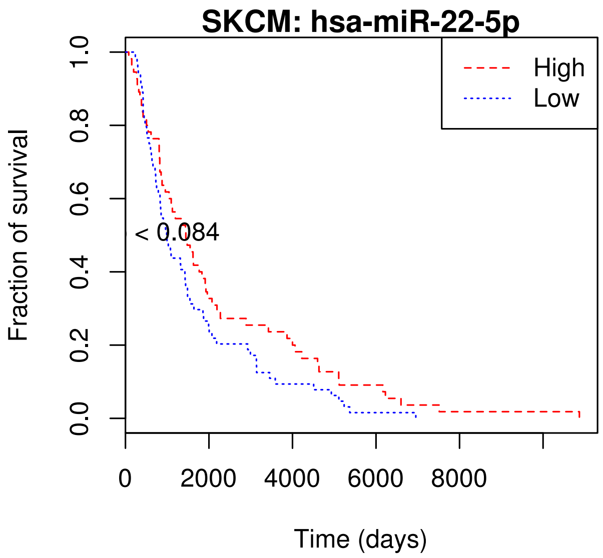
SKCM: hsa-miR-22-5p
High
Low
< 0.084
Fraction of survival
0.0 0.2 0.4 0.6 0.8 1.0
0 2000 4000 6000 8000
Time (days)

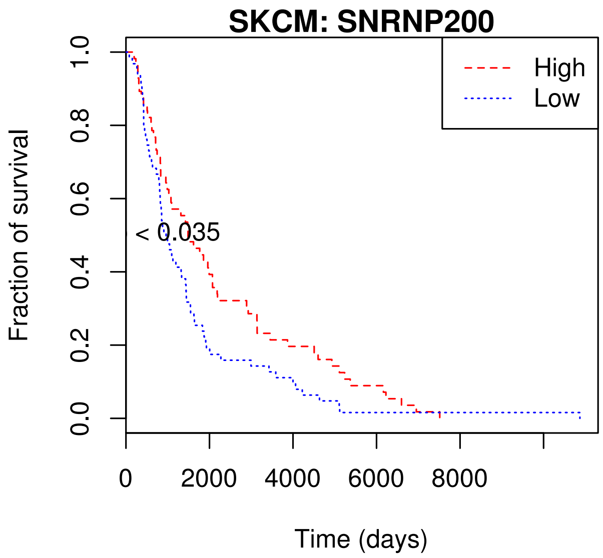
SKCM: SNRNP200
High
Low
< 0.035
Fraction of survival
0.0 0.2 0.4 0.6 0.8 1.0
0 2000 4000 6000 8000
Time (days)

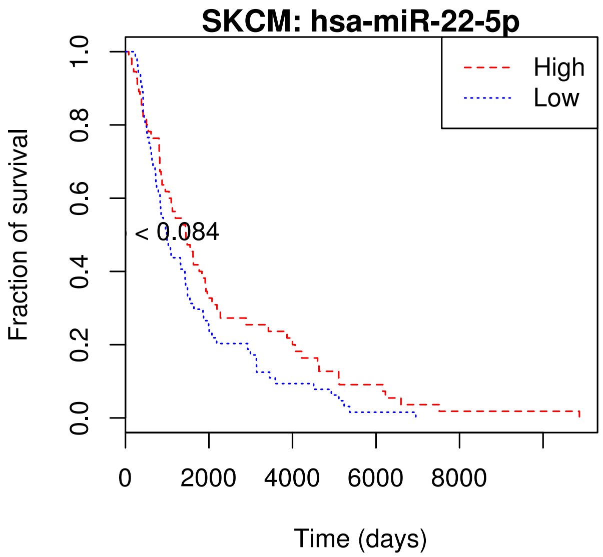
SKCM: hsa-miR-22-5p
High
Low
< 0.084
Fraction of survival
0.0 0.2 0.4 0.6 0.8 1.0
0 2000 4000 6000 8000
Time (days)

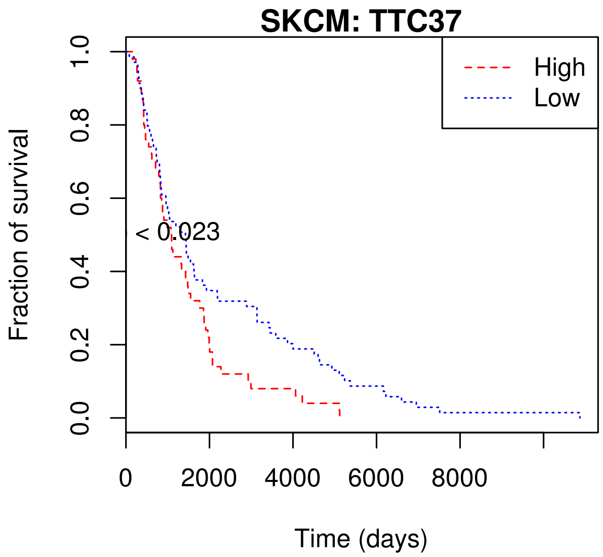
SKCM: TTC37
High
Low
< 0.023
Fraction of survival
0.0 0.2 0.4 0.6 0.8 1.0
0 2000 4000 6000 8000
Time (days)

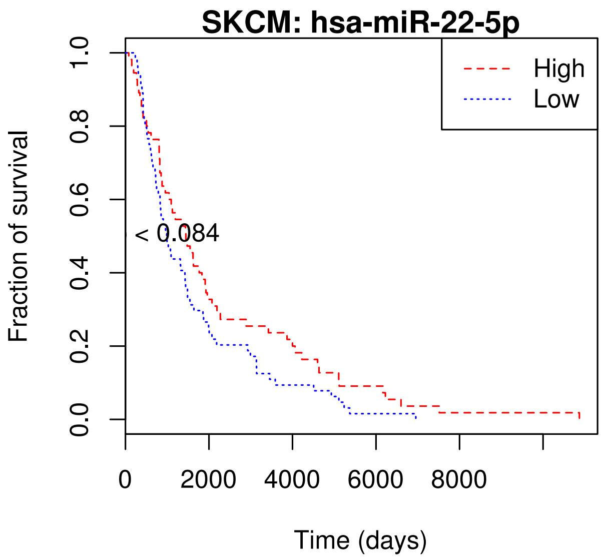
SKCM: hsa-miR-22-5p
High
Low
< 0.084
Fraction of survival
0.0 0.2 0.4 0.6 0.8 1.0
0 2000 4000 6000 8000
Time (days)

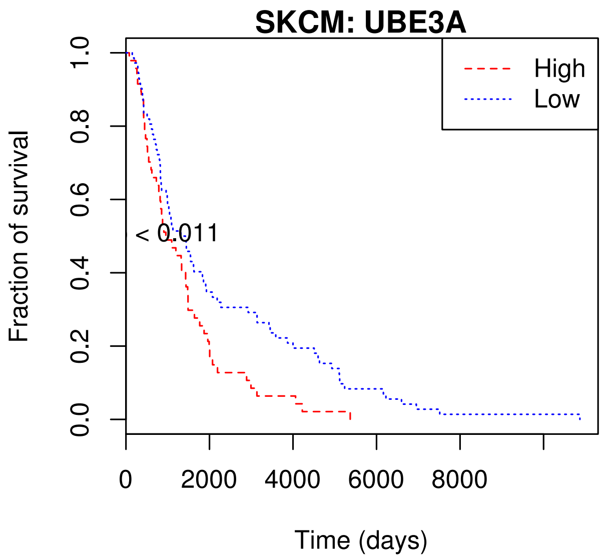
SKCM: UBE3A
High
Low
< 0.011
Fraction of survival
0.0 0.2 0.4 0.6 0.8 1.0
0 2000 4000 6000 8000
Time (days)

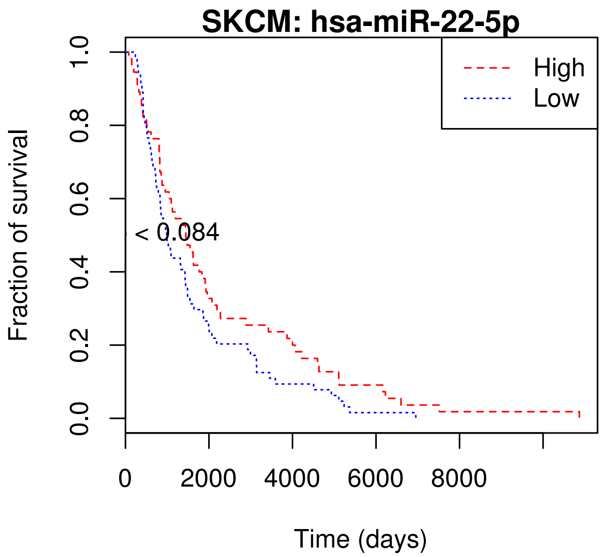
SKCM: hsa-miR-22-5p
High
Low
< 0.084
Fraction of survival
0.0
0.2
0.4
0.6
0.8
1.0
0
2000
4000
6000
8000
Time (days)

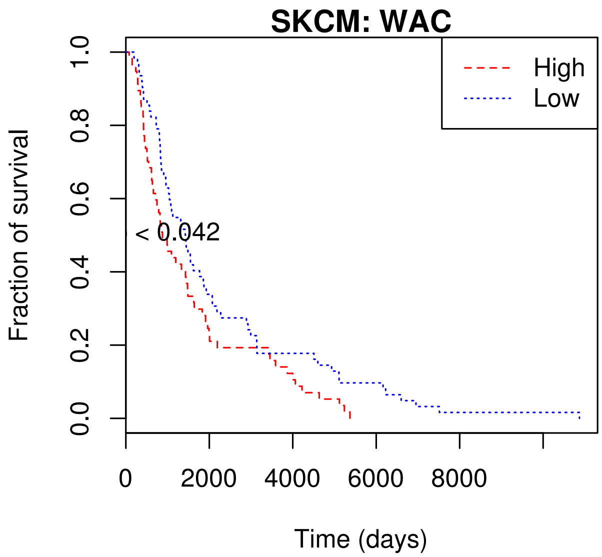
SKCM: WAC
High
Low
< 0.042
Fraction of survival
0.0
0.2
0.4
0.6
0.8
1.0
0
2000
4000
6000
8000
Time (days)

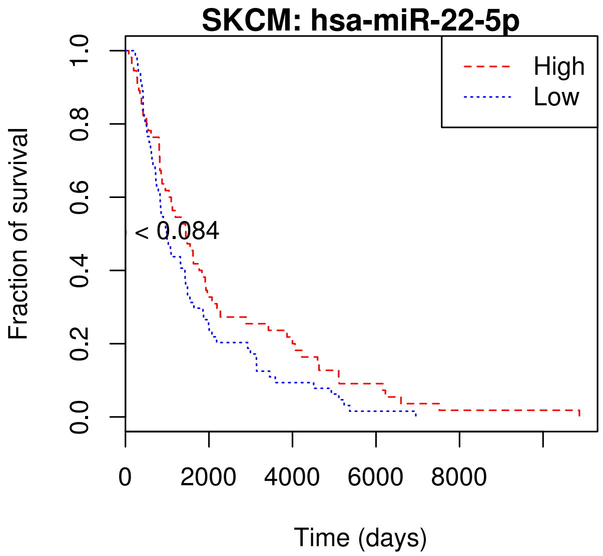
SKCM: hsa-miR-22-5p
High
Low
< 0.084
Fraction of survival
0.0
0.2
0.4
0.6
0.8
1.0
0
2000
4000
6000
8000
Time (days)

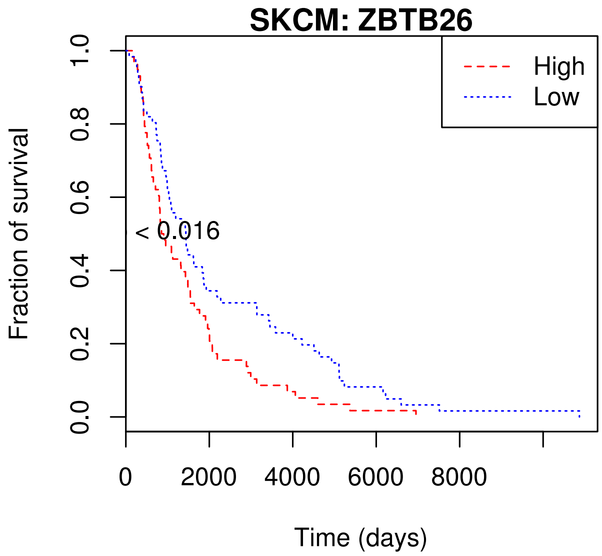
SKCM: ZBTB26
High
Low
< 0.016
Fraction of survival
0.0
0.2
0.4
0.6
0.8
1.0
0
2000
4000
6000
8000
Time (days)

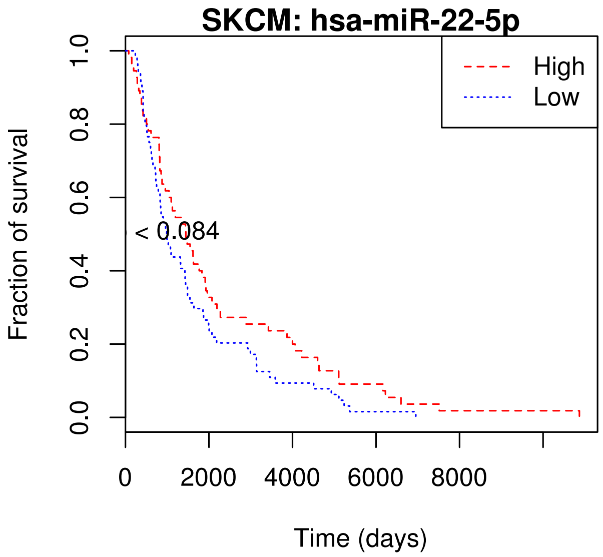
SKCM: hsa-miR-22-5p
High
Low
< 0.084
Fraction of survival
0.0
0.2
0.4
0.6
0.8
1.0
0
2000
4000
6000
8000
Time (days)

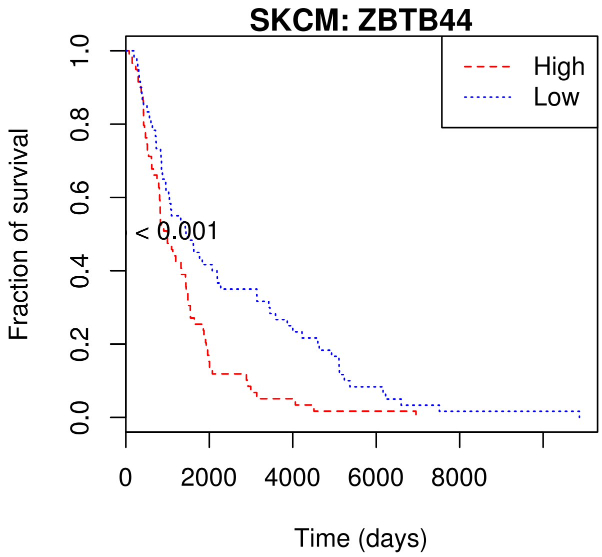
SKCM: ZBTB44
High
Low
< 0.001
Fraction of survival
0.0
0.2
0.4
0.6
0.8
1.0
0
2000
4000
6000
8000
Time (days)

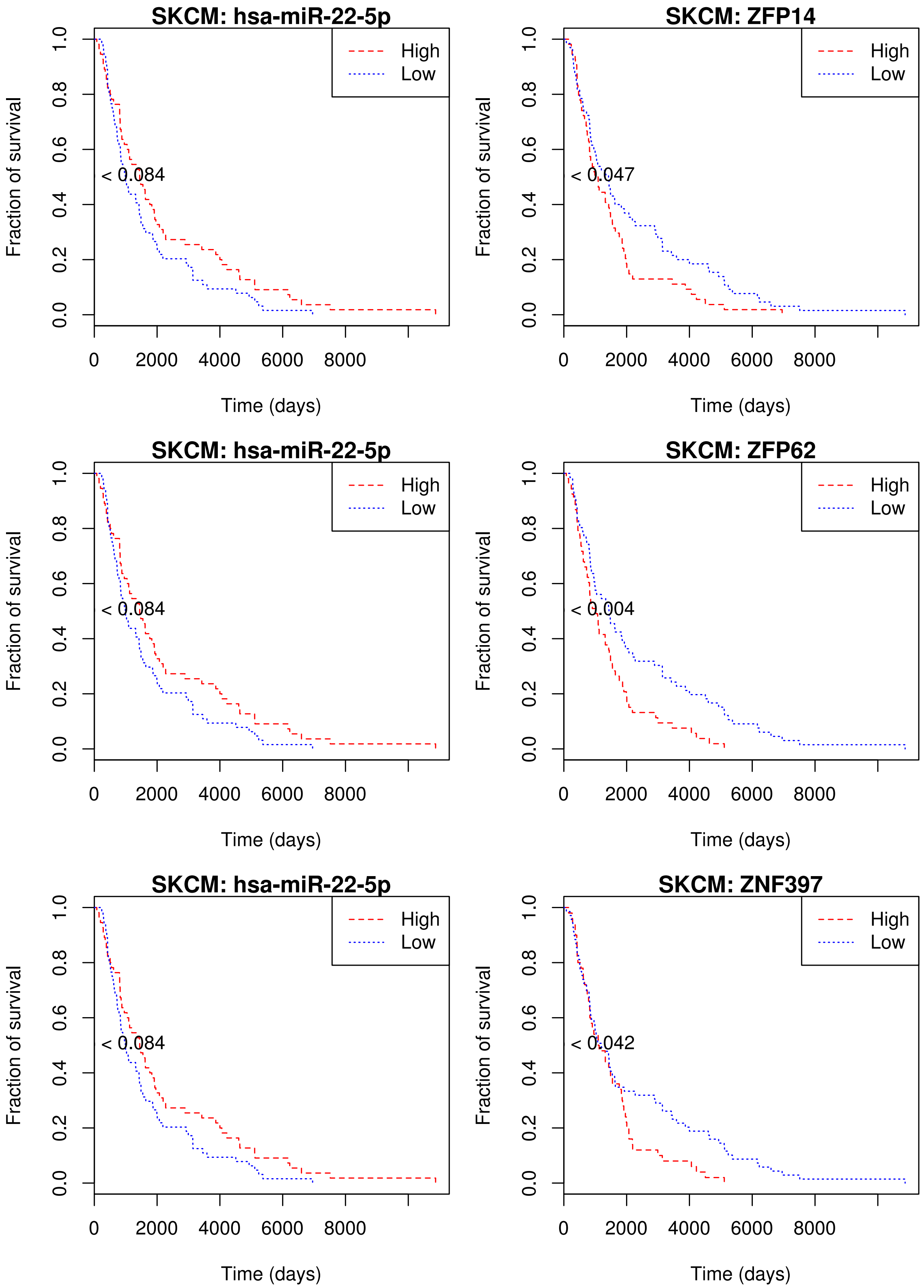
SKCM: hsa-miR-22-5p
High
Low
< 0.084
Fraction of survival
Time (days)
SKCM: ZFP14
High
Low
< 0.047
Fraction of survival
Time (days)
SKCM: hsa-miR-22-5p
High
Low
< 0.084
Fraction of survival
Time (days)
SKCM: ZFP62
High
Low
< 0.004
Fraction of survival
Time (days)
SKCM: hsa-miR-22-5p
High
Low
< 0.084
Fraction of survival
Time (days)
SKCM: ZNF397
High
Low
< 0.042
Fraction of survival
Time (days)

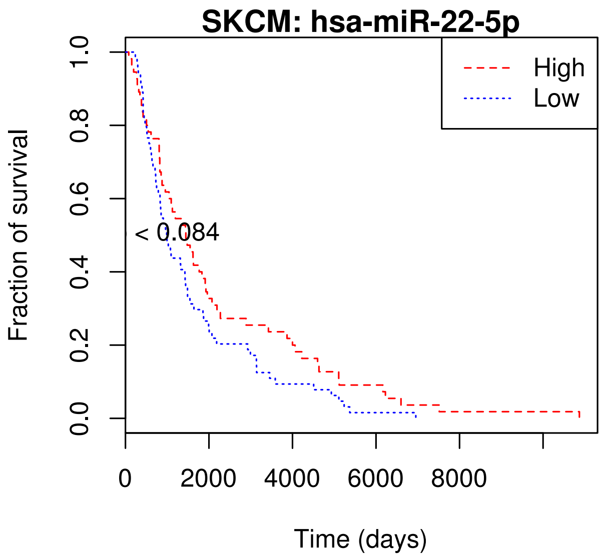
SKCM: hsa-miR-22-5p
High
Low
< 0.084
Fraction of survival
Time (days)

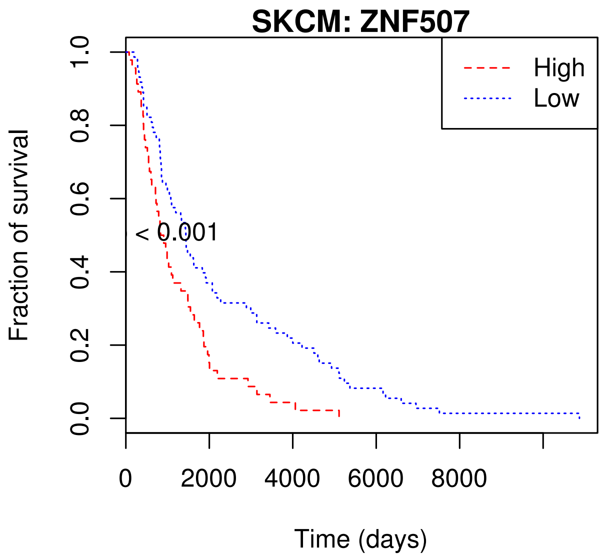
SKCM: ZNF507
High
Low
< 0.001
Fraction of survival
Time (days)

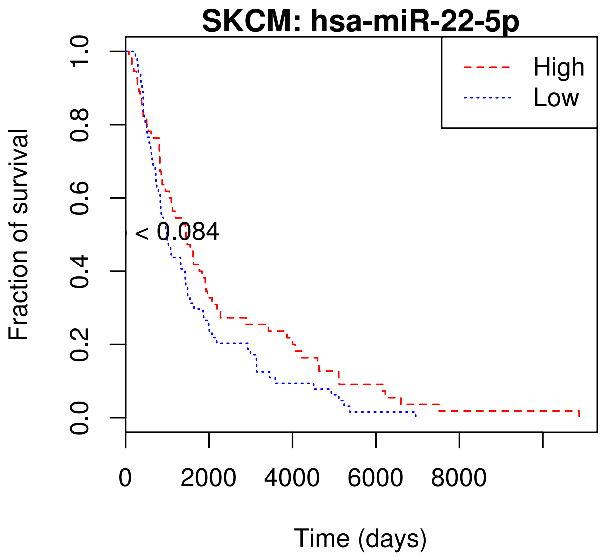
SKCM: hsa-miR-22-5p
High
Low
< 0.084
Fraction of survival
Time (days)

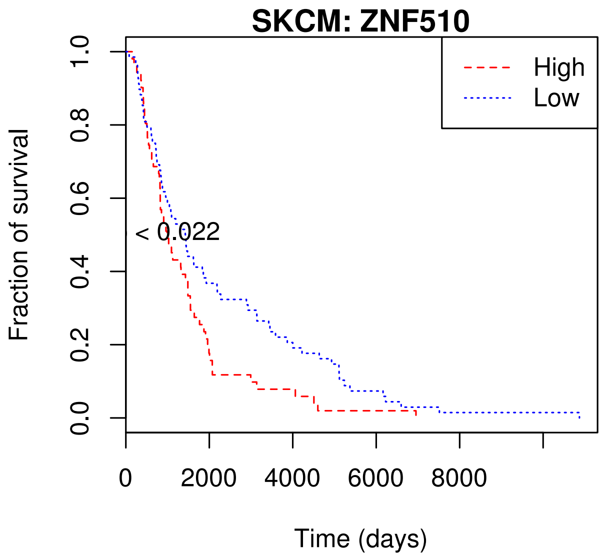
SKCM: ZNF510
High
Low
< 0.022
Fraction of survival
Time (days)

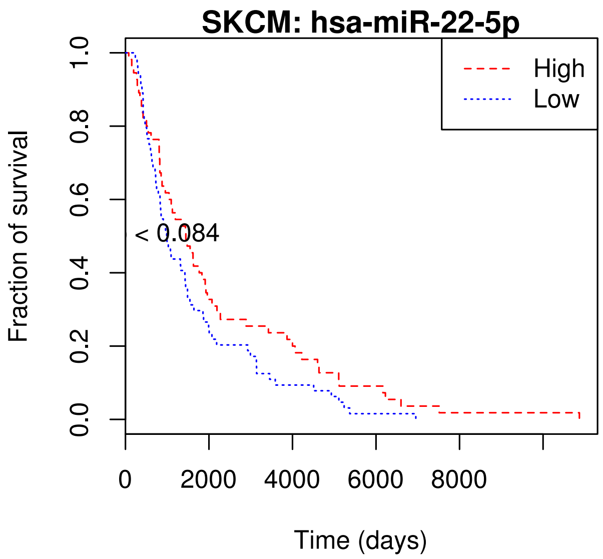
SKCM: hsa-miR-22-5p
High
Low
< 0.084
Fraction of survival
Time (days)

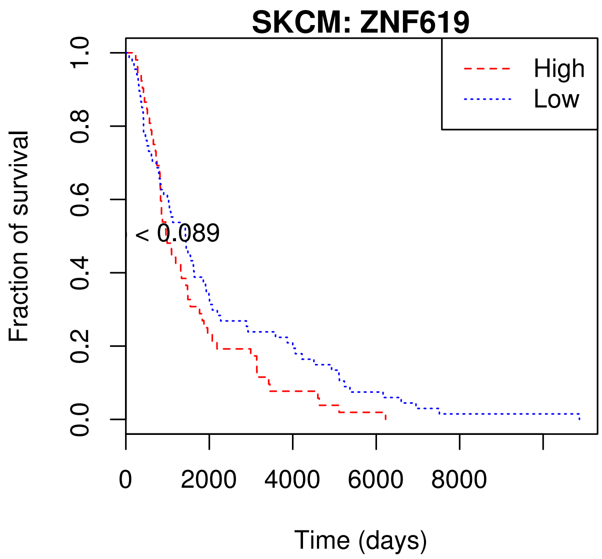
SKCM: ZNF619
High
Low
< 0.089
Fraction of survival
Time (days)

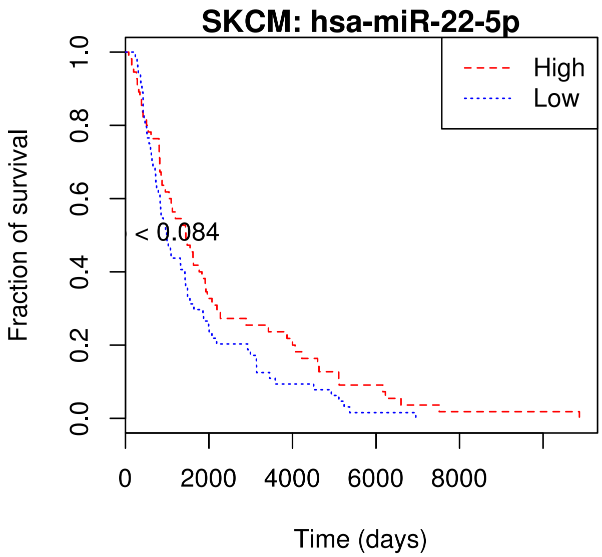
SKCM: hsa-miR-22-5p
High
Low
< 0.084
Fraction of survival
Time (days)

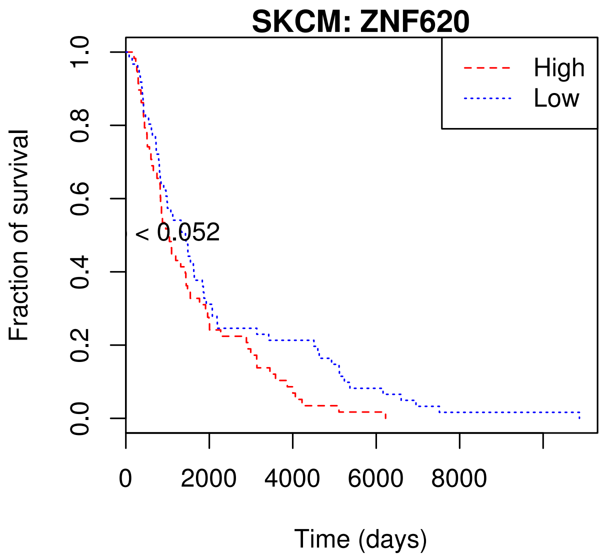
SKCM: ZNF620
High
Low
< 0.052
Fraction of survival
Time (days)

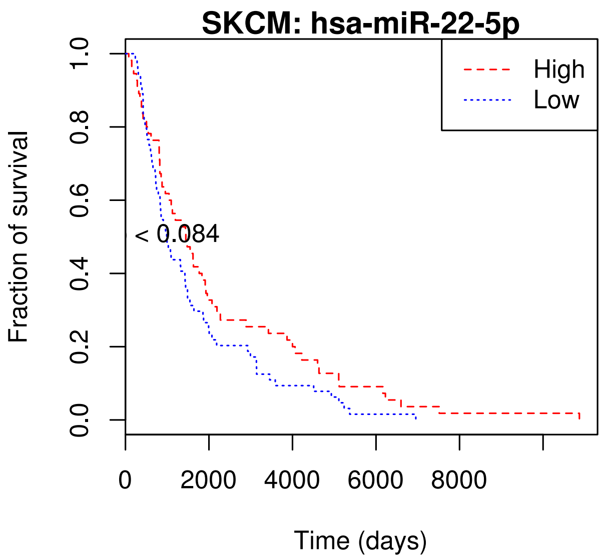
SKCM: hsa-miR-22-5p
High
Low
< 0.084
Fraction of survival
Time (days)

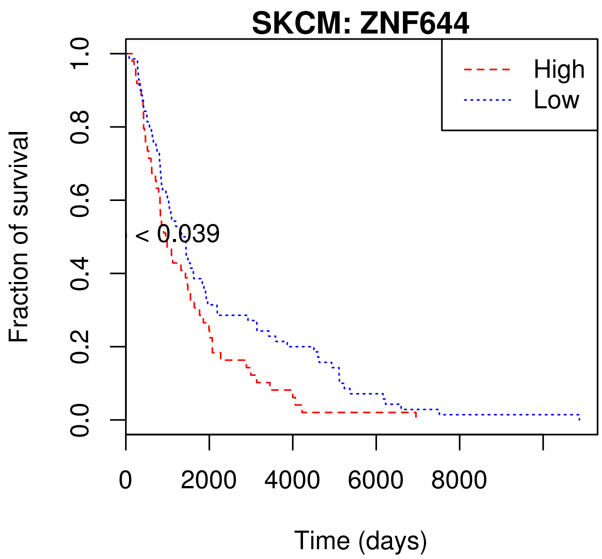
SKCM: ZNF644
High
Low
< 0.039
Fraction of survival
Time (days)

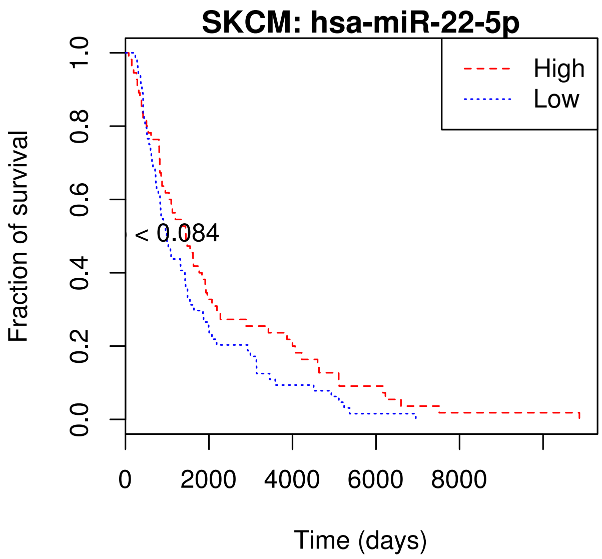
SKCM: hsa-miR-22-5p
High
Low
< 0.084
Fraction of survival
Time (days)

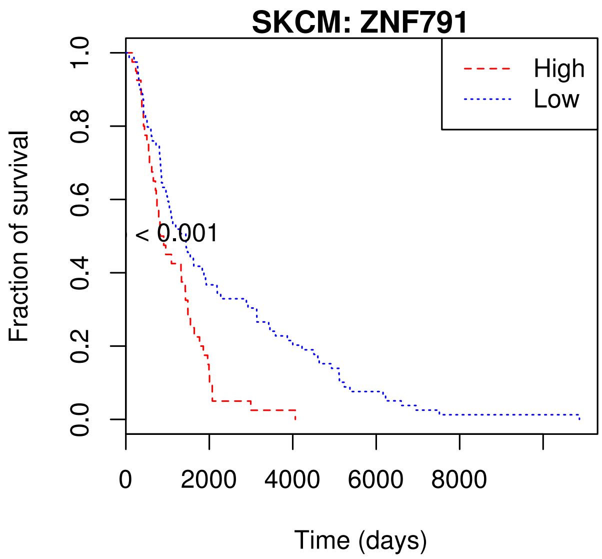
SKCM: ZNF791
High
Low
< 0.001
Fraction of survival
Time (days)

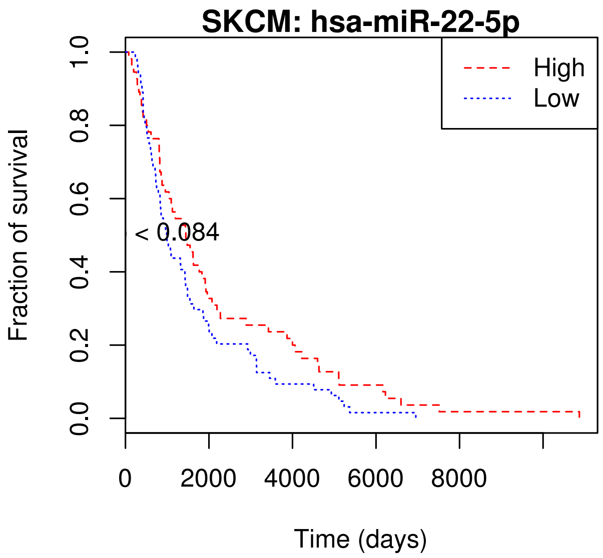
SKCM: hsa-miR-22-5p
High
Low
< 0.084
Fraction of survival
0.0
0.2
0.4
0.6
0.8
1.0
0
2000
4000
6000
8000
Time (days)

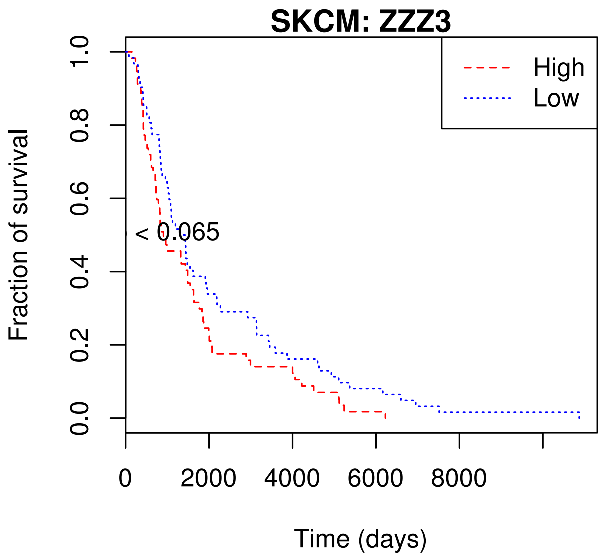
SKCM: ZZZ3
High
Low
< 0.065
Fraction of survival
0.0
0.2
0.4
0.6
0.8
1.0
0
2000
4000
6000
8000
Time (days)

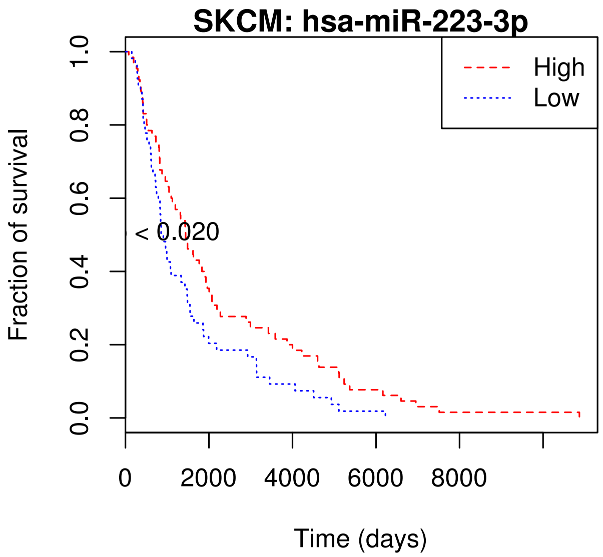
SKCM: hsa-miR-223-3p
High
Low
< 0.020
Fraction of survival
0.0
0.2
0.4
0.6
0.8
1.0
0
2000
4000
6000
8000
Time (days)

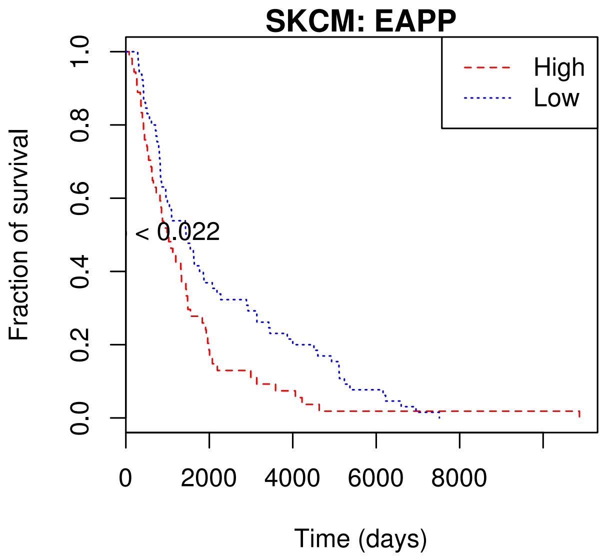
SKCM: EAPP
High
Low
< 0.022
Fraction of survival
0.0
0.2
0.4
0.6
0.8
1.0
0
2000
4000
6000
8000
Time (days)

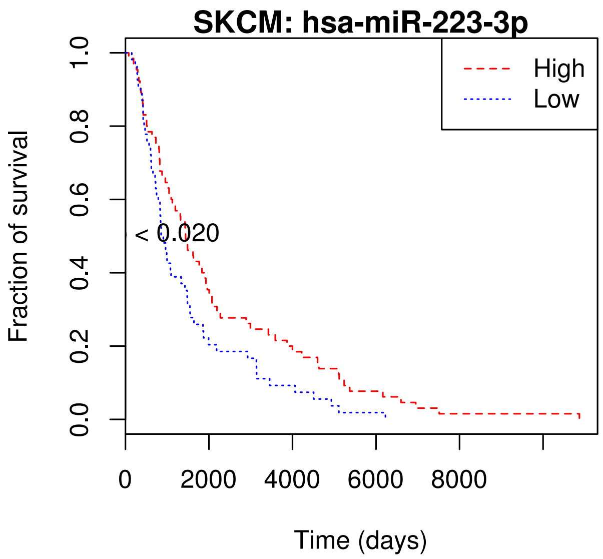
SKCM: hsa-miR-223-3p
High
Low
< 0.020
Fraction of survival
0.0
0.2
0.4
0.6
0.8
1.0
0
2000
4000
6000
8000
Time (days)

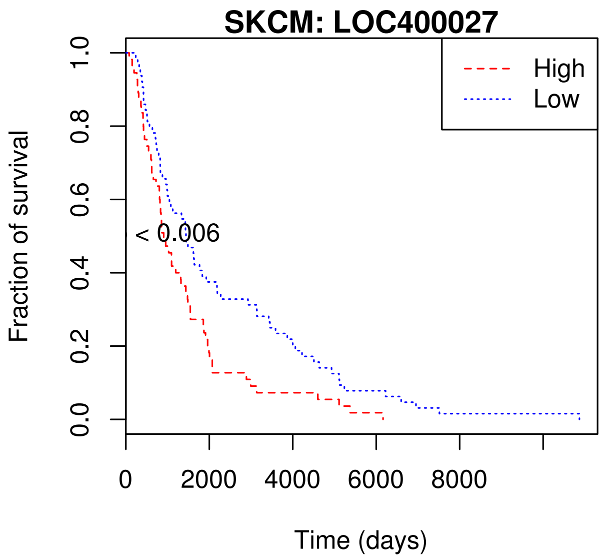
SKCM: LOC400027
High
Low
< 0.006
Fraction of survival
0.0
0.2
0.4
0.6
0.8
1.0
0
2000
4000
6000
8000
Time (days)

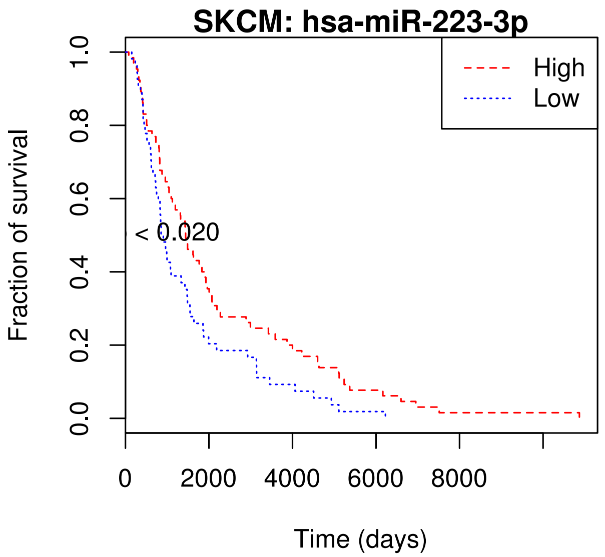
SKCM: hsa-miR-223-3p
High
Low
< 0.020
Fraction of survival
Time (days)

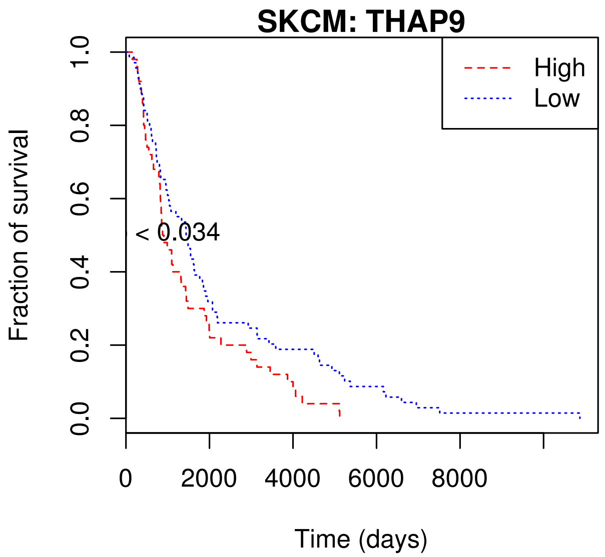
SKCM: THAP9
High
Low
< 0.034
Fraction of survival
Time (days)

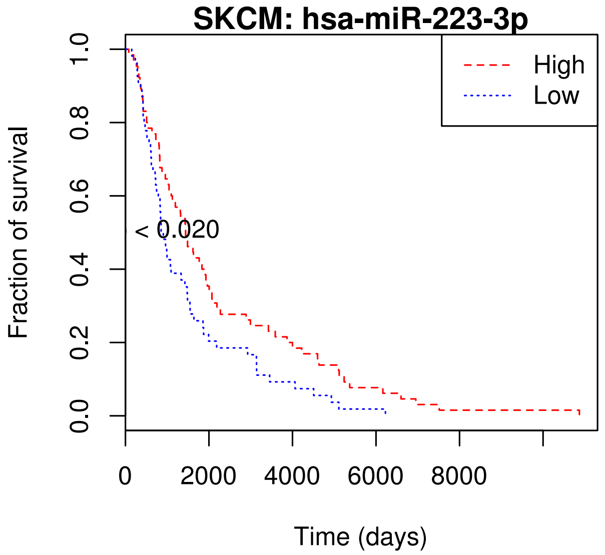
SKCM: hsa-miR-223-3p
High
Low
< 0.020
Fraction of survival
Time (days)

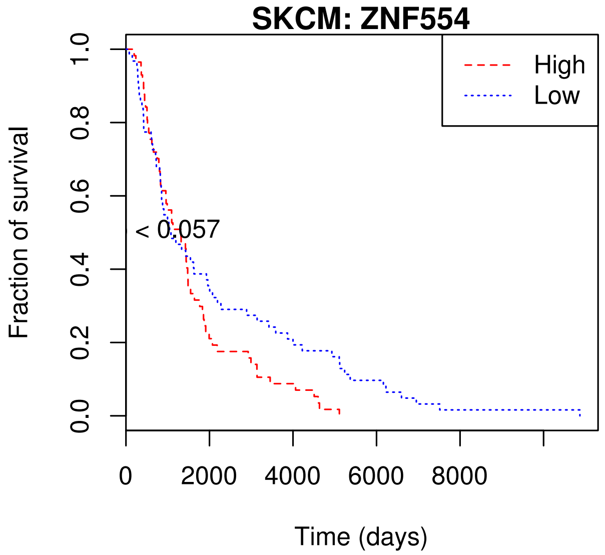
SKCM: ZNF554
High
Low
< 0.057
Fraction of survival
Time (days)

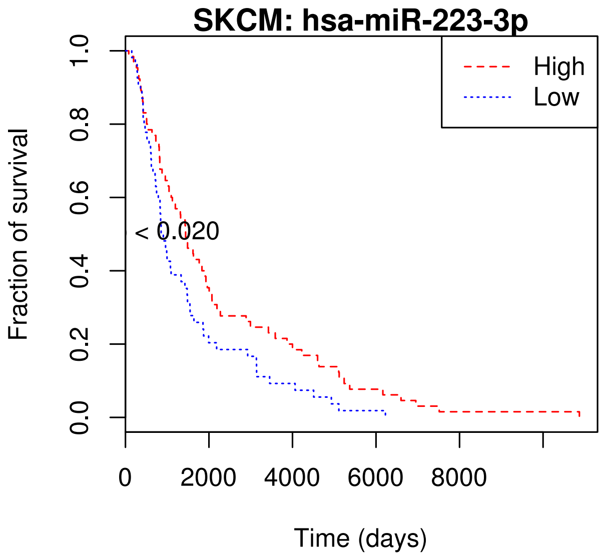
SKCM: hsa-miR-223-3p
High
Low
< 0.020
Fraction of survival
Time (days)

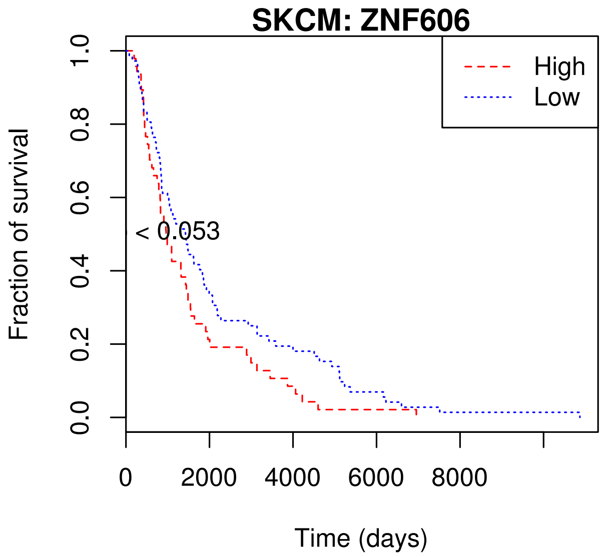
SKCM: ZNF606
High
Low
< 0.053
Fraction of survival
Time (days)

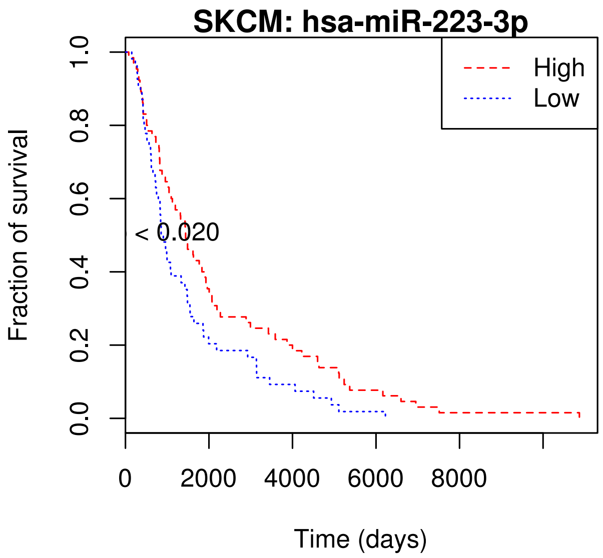
SKCM: hsa-miR-223-3p
High
Low
< 0.020
Fraction of survival
0.0 0.2 0.4 0.6 0.8 1.0
0 2000 4000 6000 8000
Time (days)

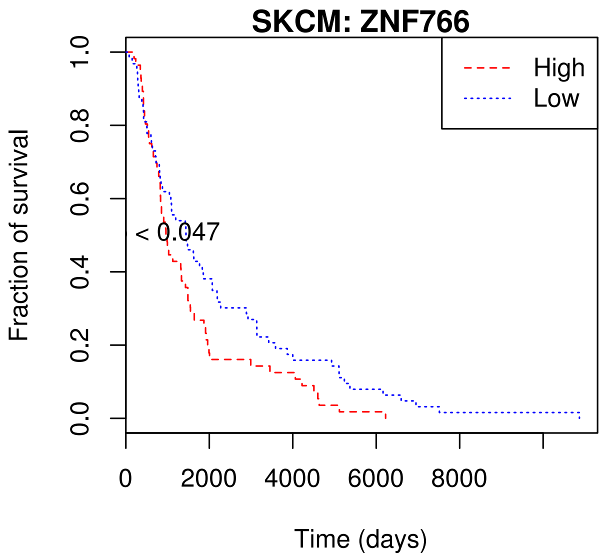
SKCM: ZNF766
High
Low
< 0.047
Fraction of survival
0.0 0.2 0.4 0.6 0.8 1.0
0 2000 4000 6000 8000
Time (days)

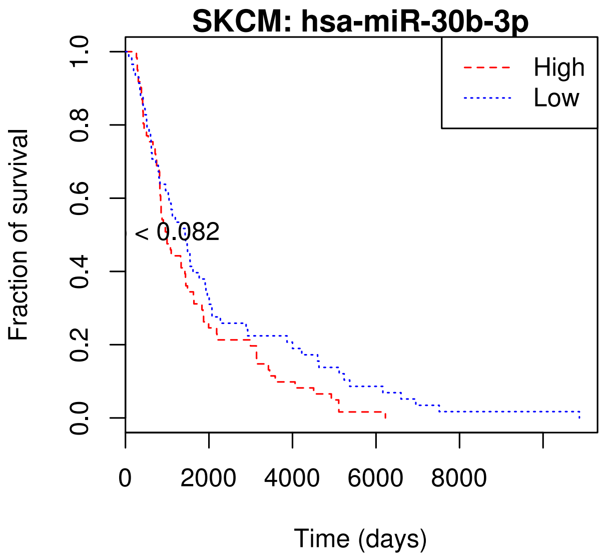
SKCM: hsa-miR-30b-3p
High
Low
< 0.082
Fraction of survival
0.0 0.2 0.4 0.6 0.8 1.0
0 2000 4000 6000 8000
Time (days)

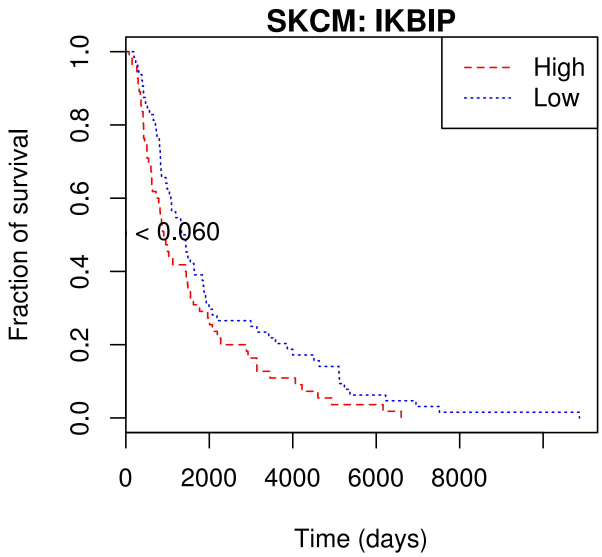
SKCM: IKBIP
High
Low
< 0.060
Fraction of survival
0.0 0.2 0.4 0.6 0.8 1.0
0 2000 4000 6000 8000
Time (days)

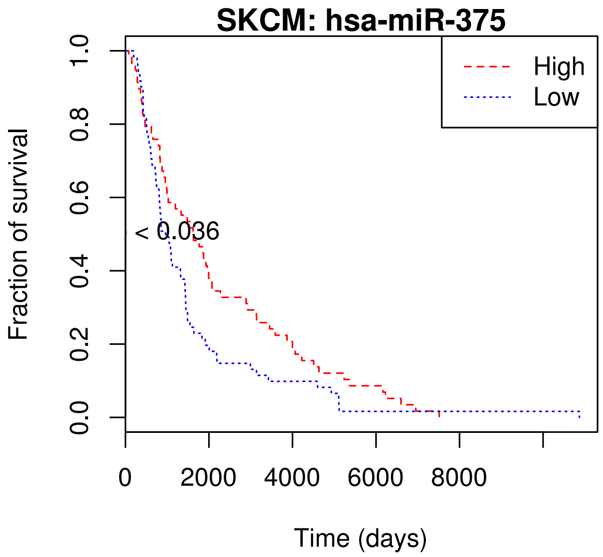
SKCM: hsa-miR-375
High
Low
< 0.036
Fraction of survival
0.0 0.2 0.4 0.6 0.8 1.0
0 2000 4000 6000 8000
Time (days)

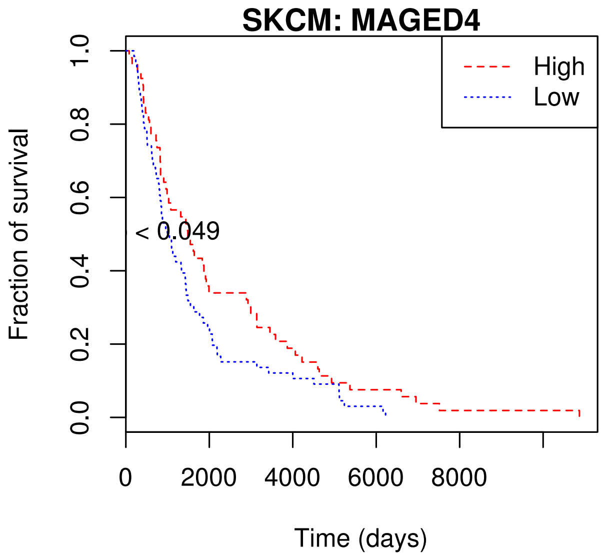
SKCM: MAGED4
High
Low
< 0.049
Fraction of survival
0.0 0.2 0.4 0.6 0.8 1.0
0 2000 4000 6000 8000
Time (days)

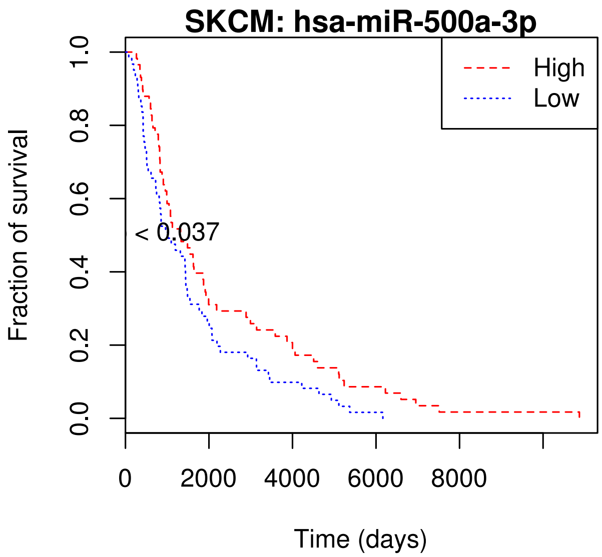
SKCM: hsa-miR-500a-3p
High
Low
< 0.037
Fraction of survival
0.0 0.2 0.4 0.6 0.8 1.0
0 2000 4000 6000 8000
Time (days)

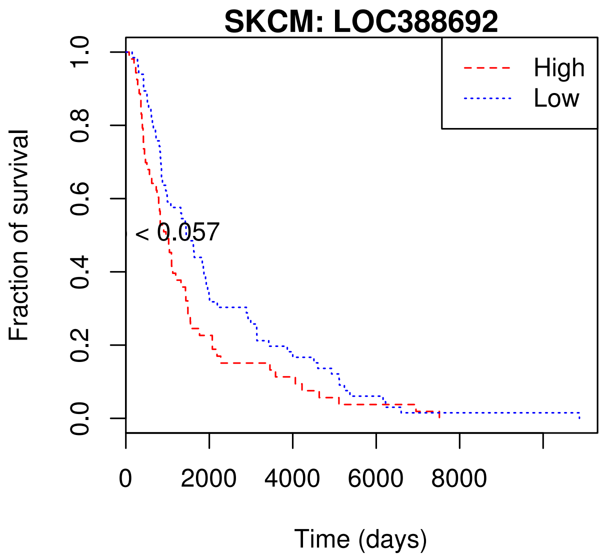
SKCM: LOC388692
High
Low
< 0.057
Fraction of survival
0.0 0.2 0.4 0.6 0.8 1.0
0 2000 4000 6000 8000
Time (days)

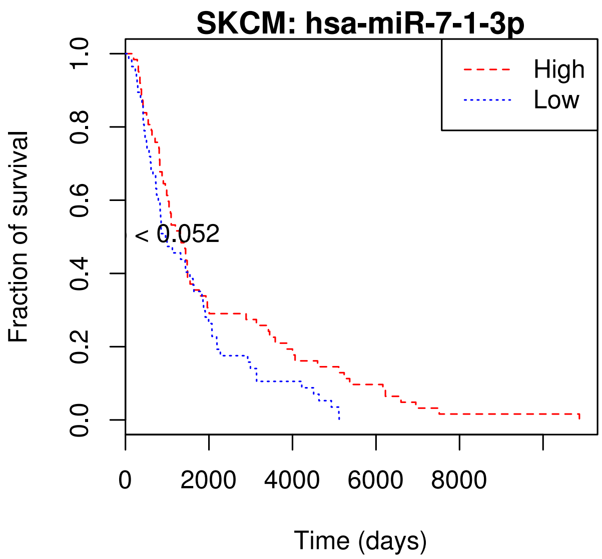
SKCM: hsa-miR-7-1-3p
High
Low
< 0.052
Fraction of survival
0.0 0.2 0.4 0.6 0.8 1.0
0 2000 4000 6000 8000
Time (days)

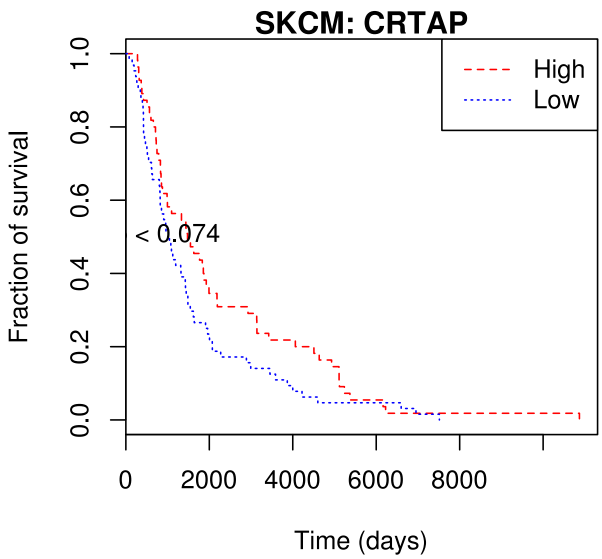
SKCM: CRTAP
High
Low
< 0.074
Fraction of survival
0.0 0.2 0.4 0.6 0.8 1.0
0 2000 4000 6000 8000
Time (days)

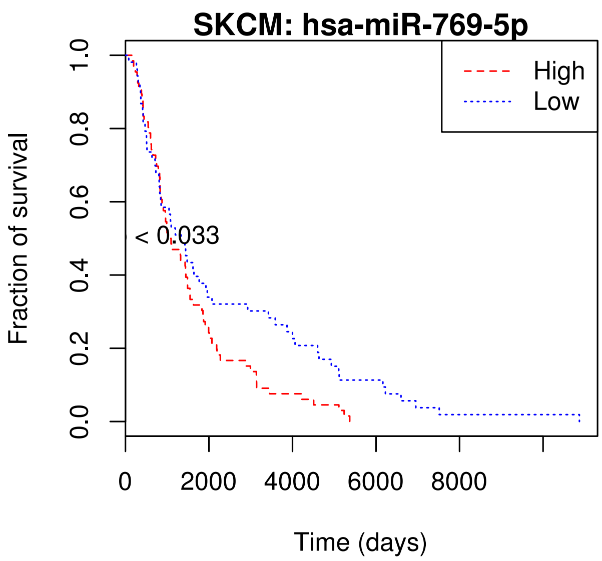
SKCM: hsa-miR-769-5p
High
Low
< 0.033
Fraction of survival
0.0 0.2 0.4 0.6 0.8 1.0
0 2000 4000 6000 8000
Time (days)

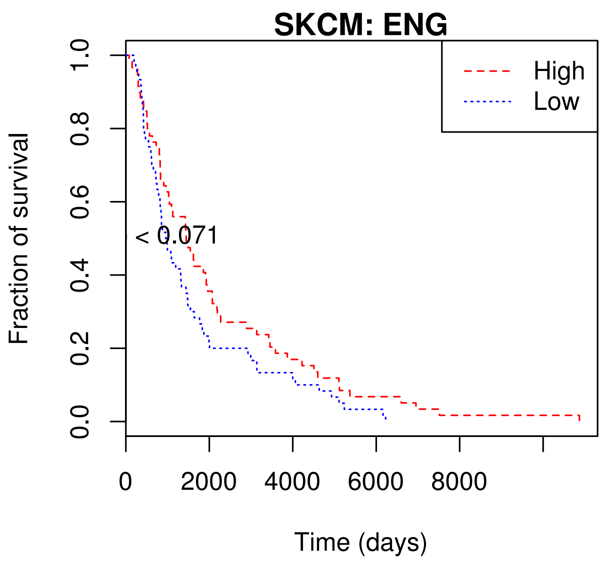
SKCM: ENG
High
Low
< 0.071
Fraction of survival
0.0 0.2 0.4 0.6 0.8 1.0
0 2000 4000 6000 8000
Time (days)

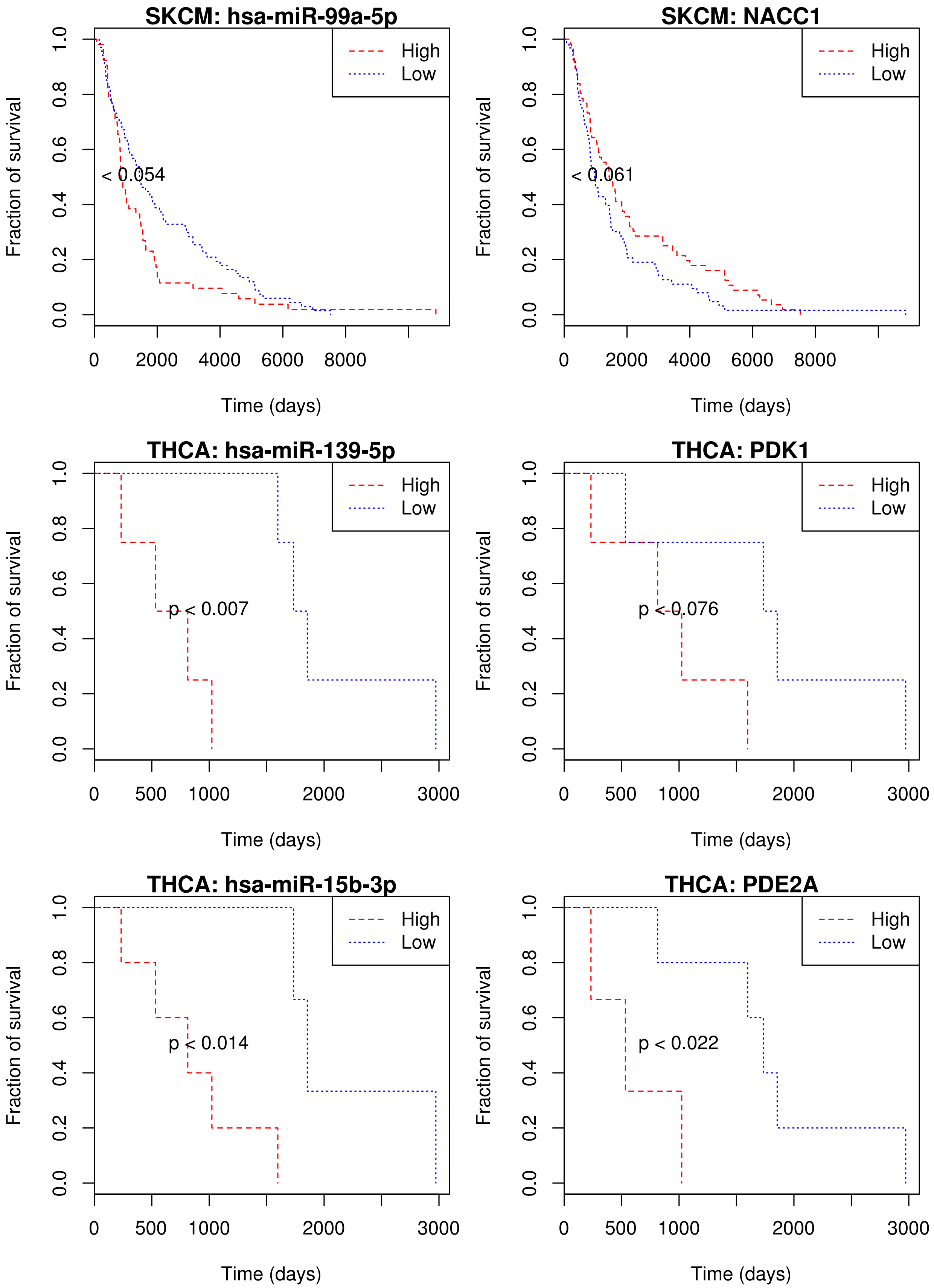
SKCM: hsa-miR-99a-5p
High
Low
< 0.054
Fraction of survival
Time (days)
SKCM: NACC1
High
Low
< 0.061
Fraction of survival
Time (days)
THCA: hsa-miR-139-5p
High
Low
p < 0.007
Fraction of survival
Time (days)
THCA: PDK1
High
Low
p < 0.076
Fraction of survival
Time (days)
THCA: hsa-miR-15b-3p
High
Low
p < 0.014
Fraction of survival
Time (days)
THCA: PDE2A
High
Low
p < 0.022
Fraction of survival
Time (days)

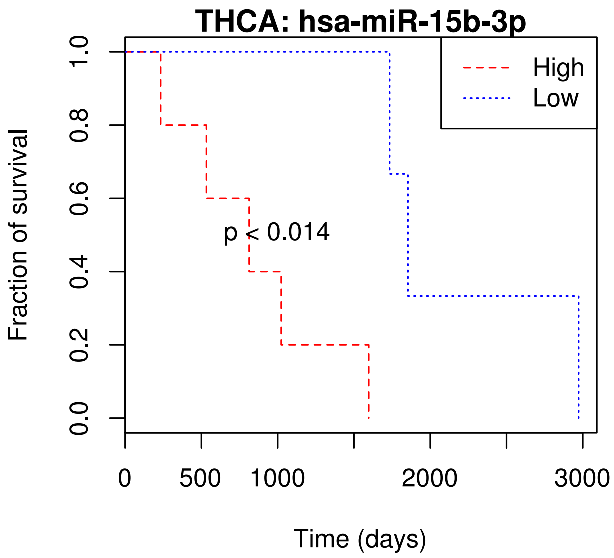
THCA: hsa-miR-15b-3p
High
Low
p < 0.014
Fraction of survival
0.0 0.2 0.4 0.6 0.8 1.0
0 500 1000 2000 3000
Time (days)

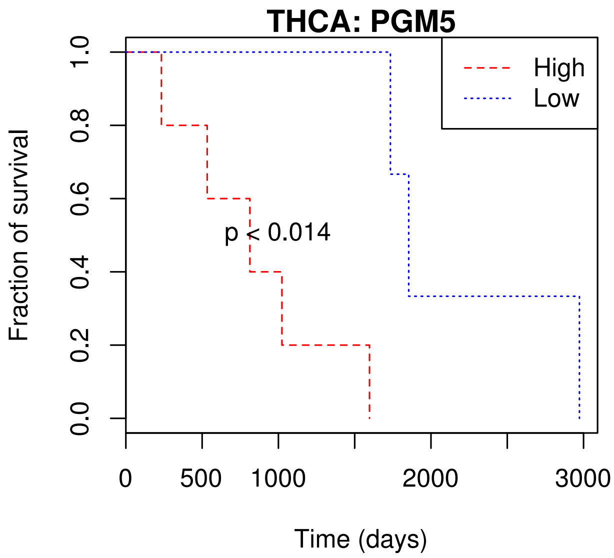
THCA: PGM5
High
Low
p < 0.014
Fraction of survival
0.0 0.2 0.4 0.6 0.8 1.0
0 500 1000 2000 3000
Time (days)

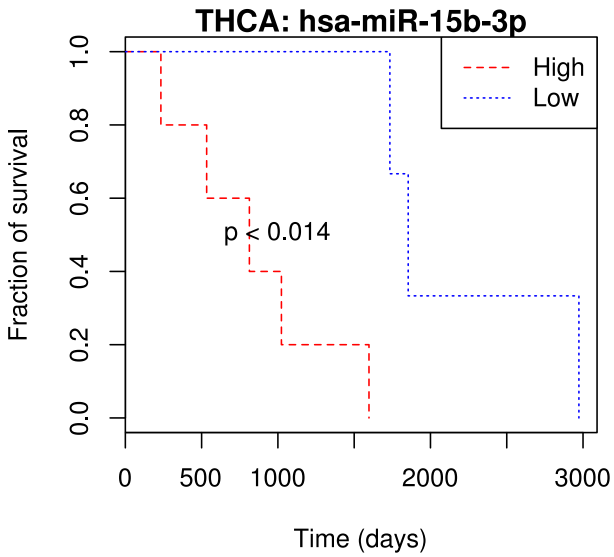
THCA: hsa-miR-15b-3p
High
Low
p < 0.014
Fraction of survival
0.0 0.2 0.4 0.6 0.8 1.0
0 500 1000 2000 3000
Time (days)

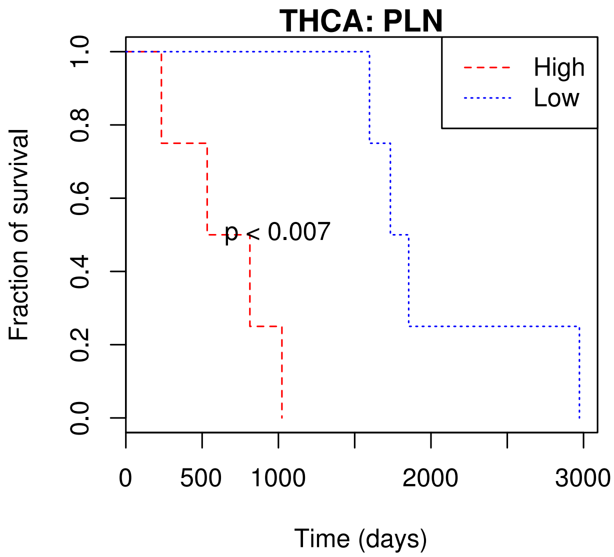
THCA: PLN
High
Low
p < 0.007
Fraction of survival
0.0 0.2 0.4 0.6 0.8 1.0
0 500 1000 2000 3000
Time (days)

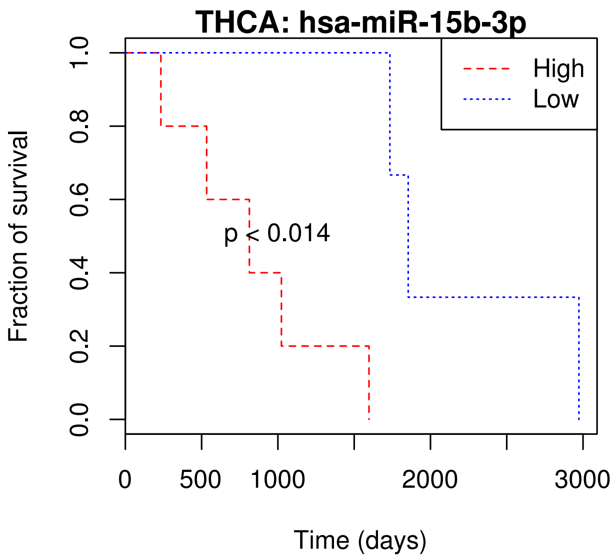
THCA: hsa-miR-15b-3p
High
Low
p < 0.014
Fraction of survival
0.0 0.2 0.4 0.6 0.8 1.0
0 500 1000 2000 3000
Time (days)

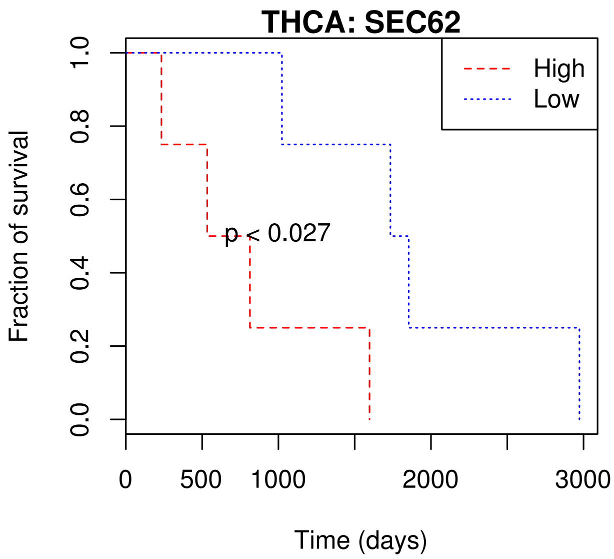
THCA: SEC62
High
Low
p < 0.027
Fraction of survival
0.0 0.2 0.4 0.6 0.8 1.0
0 500 1000 2000 3000
Time (days)

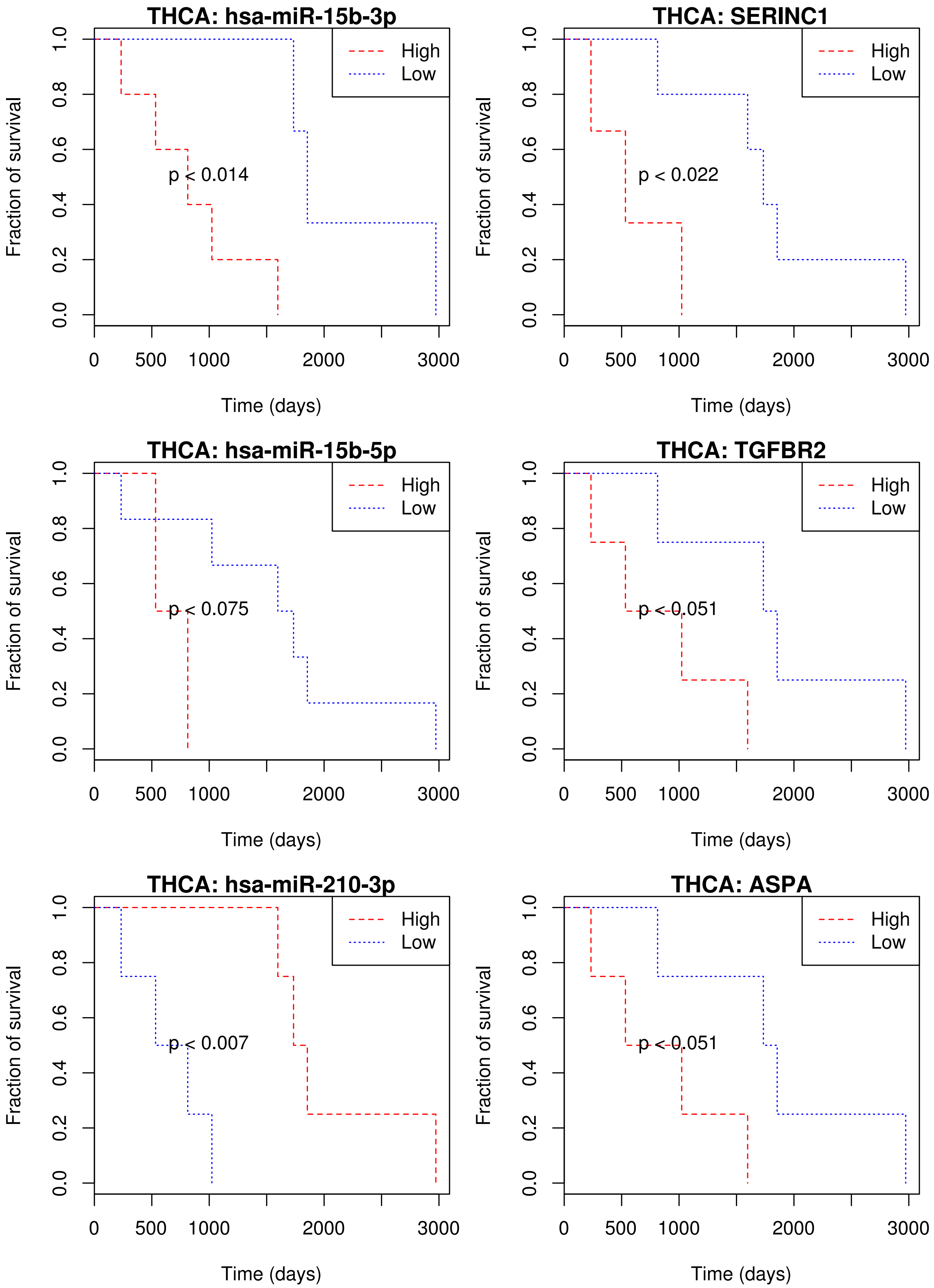
THCA: hsa-miR-15b-3p
p < 0.014
THCA: SERINC1
p < 0.022
THCA: hsa-miR-15b-5p
p < 0.075
THCA: TGFBR2
p < 0.051
THCA: hsa-miR-210-3p
p < 0.007
THCA: ASPA
p < 0.051
High
Low
Fraction of survival
Time (days)
0.0 0.2 0.4 0.6 0.8 1.0
0 500 1000 2000 3000

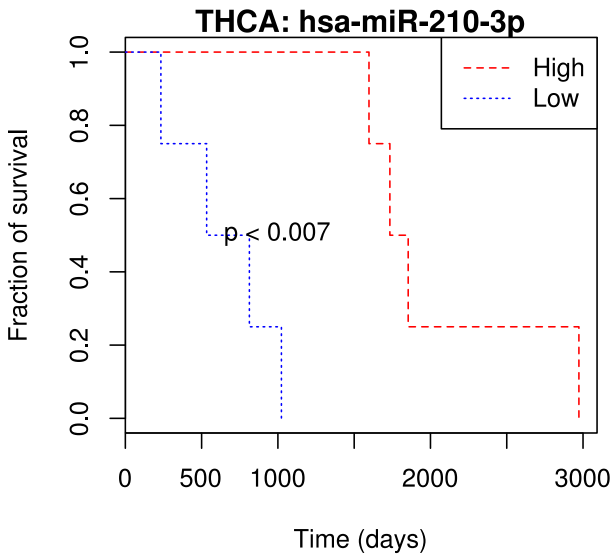
THCA: hsa-miR-210-3p
High
Low
p < 0.007
Fraction of survival
Time (days)
0.0 0.2 0.4 0.6 0.8 1.0
0 500 1000 2000 3000

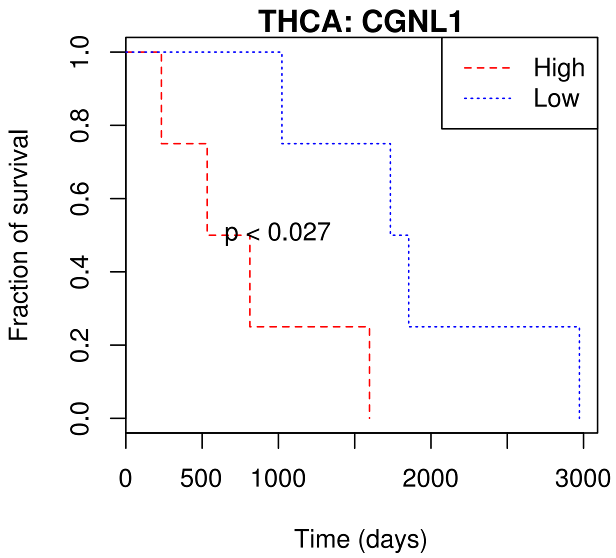
THCA: CGNL1
High
Low
p < 0.027
Fraction of survival
Time (days)
0.0 0.2 0.4 0.6 0.8 1.0
0 500 1000 2000 3000

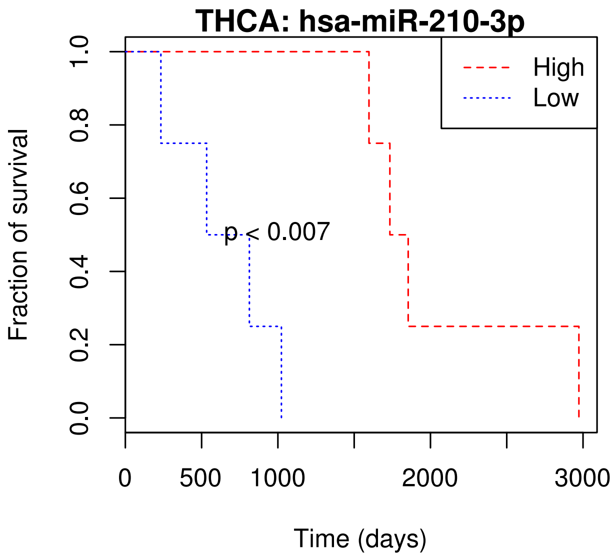
THCA: hsa-miR-210-3p
High
Low
p < 0.007
Fraction of survival
Time (days)
0.0 0.2 0.4 0.6 0.8 1.0
0 500 1000 2000 3000

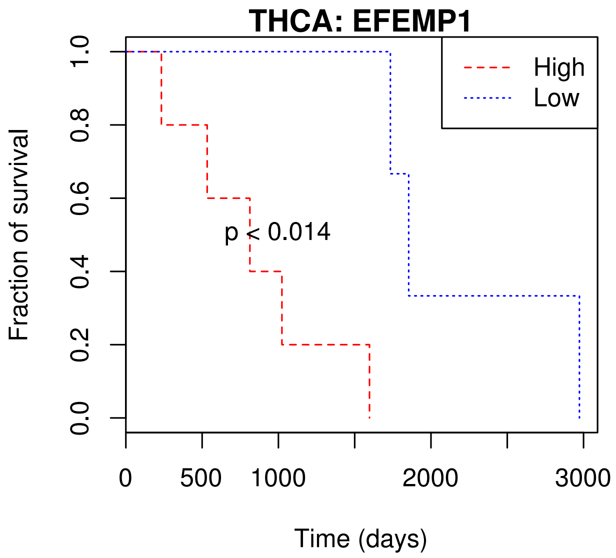
THCA: EFEMP1
High
Low
p < 0.014
Fraction of survival
Time (days)
0.0 0.2 0.4 0.6 0.8 1.0
0 500 1000 2000 3000

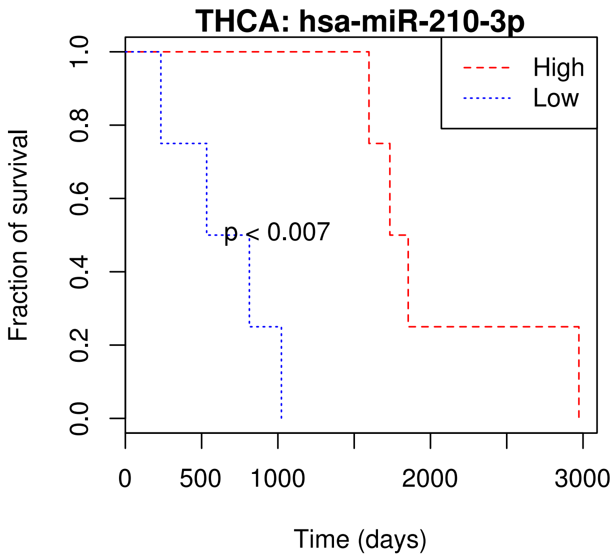
THCA: hsa-miR-210-3p
High
Low
p < 0.007
Fraction of survival
Time (days)
0.0 0.2 0.4 0.6 0.8 1.0
0 500 1000 2000 3000

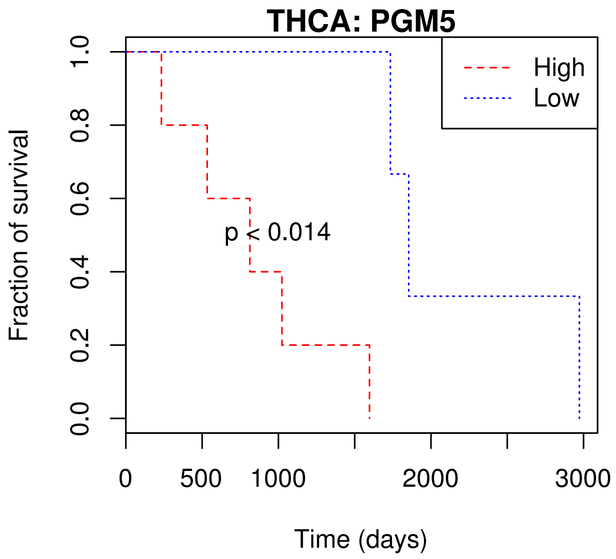
THCA: PGM5
High
Low
p < 0.014
Fraction of survival
Time (days)
0.0 0.2 0.4 0.6 0.8 1.0
0 500 1000 2000 3000

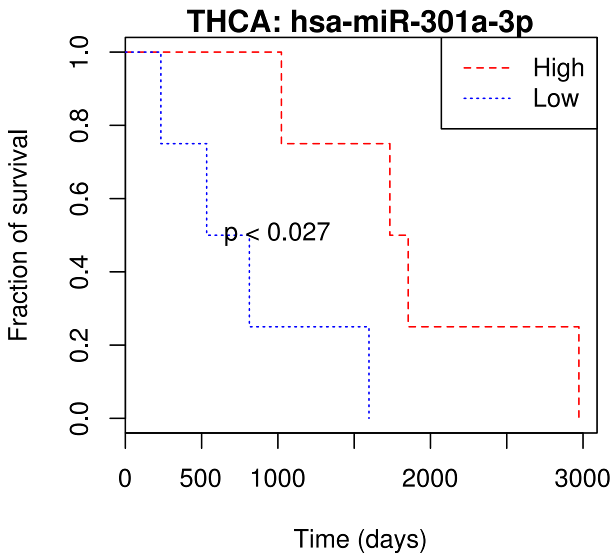
THCA: hsa-miR-301a-3p
High
Low
p < 0.027
Fraction of survival
Time (days)
0.0 0.2 0.4 0.6 0.8 1.0
0 500 1000 2000 3000

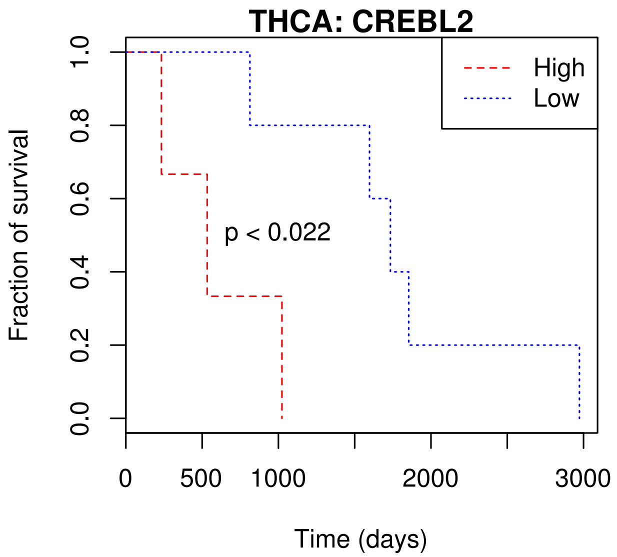
THCA: CREBL2
High
Low
p < 0.022
Fraction of survival
Time (days)
0.0 0.2 0.4 0.6 0.8 1.0
0 500 1000 2000 3000

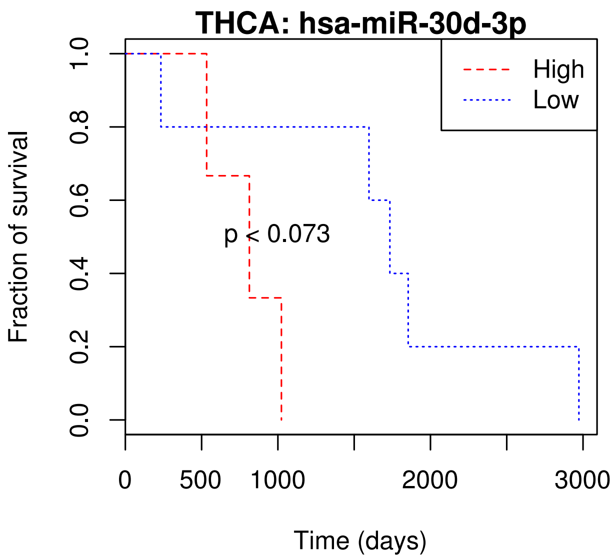
THCA: hsa-miR-30d-3p
High
Low
p < 0.073
Fraction of survival
Time (days)
0.0 0.2 0.4 0.6 0.8 1.0
0 500 1000 2000 3000

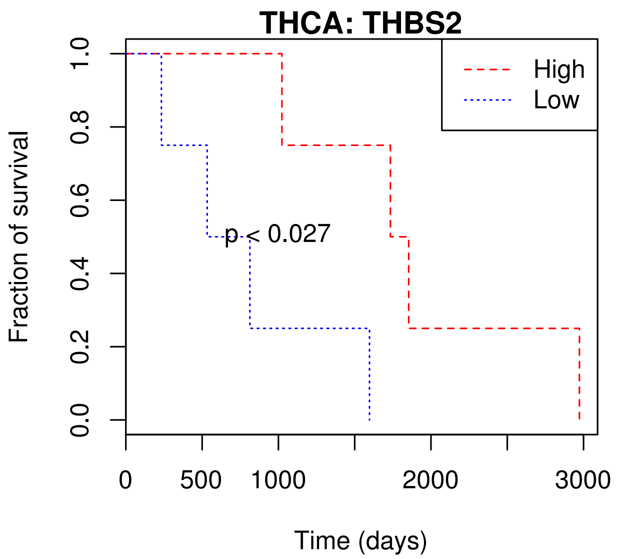
THCA: THBS2
High
Low
p < 0.027
Fraction of survival
Time (days)
0.0 0.2 0.4 0.6 0.8 1.0
0 500 1000 2000 3000

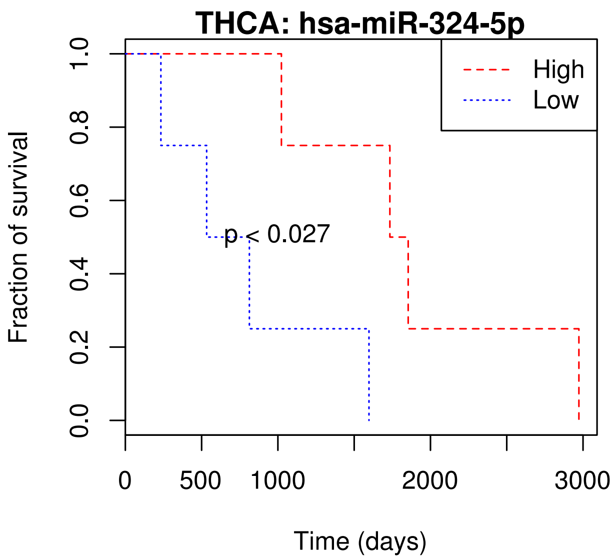
THCA: hsa-miR-324-5p
High
Low
p < 0.027
Fraction of survival
Time (days)
0.0 0.2 0.4 0.6 0.8 1.0
0 500 1000 2000 3000

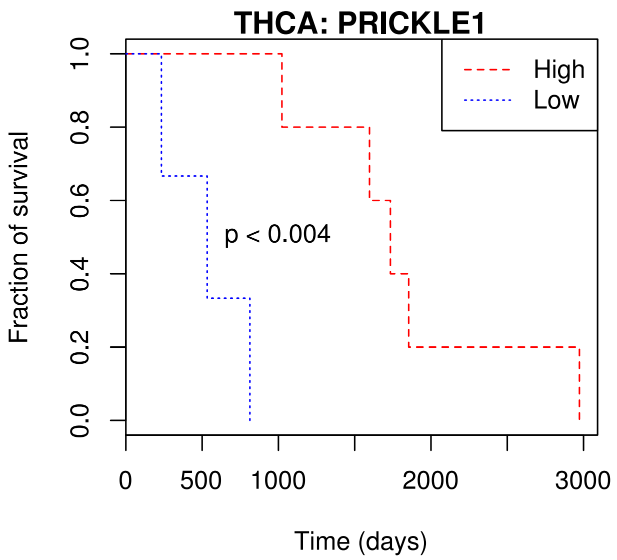
THCA: PRICKLE1
High
Low
p < 0.004
Fraction of survival
Time (days)
0.0 0.2 0.4 0.6 0.8 1.0
0 500 1000 2000 3000

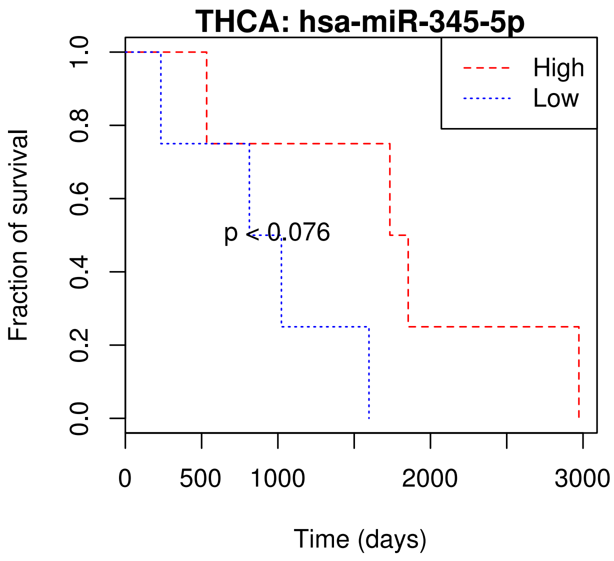
THCA: hsa-miR-345-5p
High
Low
p < 0.076
Fraction of survival
0.0
0.2
0.4
0.6
0.8
1.0
0
500
1000
2000
3000
Time (days)

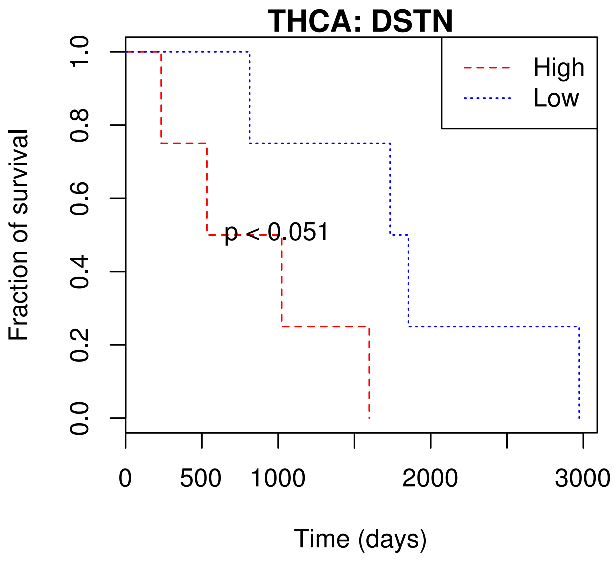
THCA: DSTN
High
Low
p < 0.051
Fraction of survival
0.0
0.2
0.4
0.6
0.8
1.0
0
500
1000
2000
3000
Time (days)

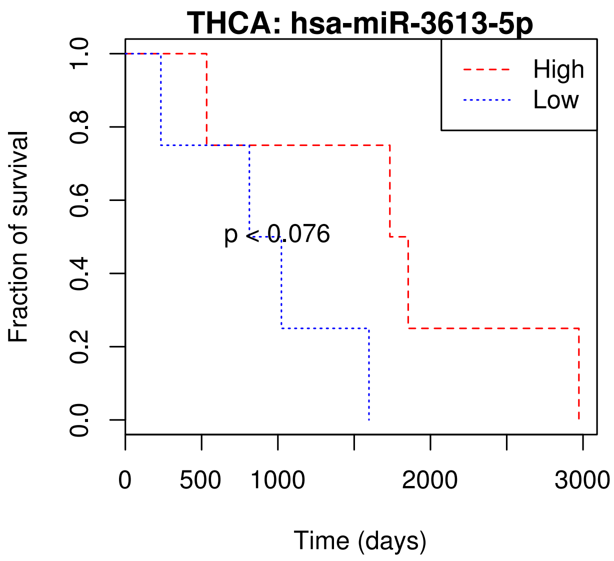
THCA: hsa-miR-3613-5p
High
Low
p < 0.076
Fraction of survival
0.0
0.2
0.4
0.6
0.8
1.0
0
500
1000
2000
3000
Time (days)

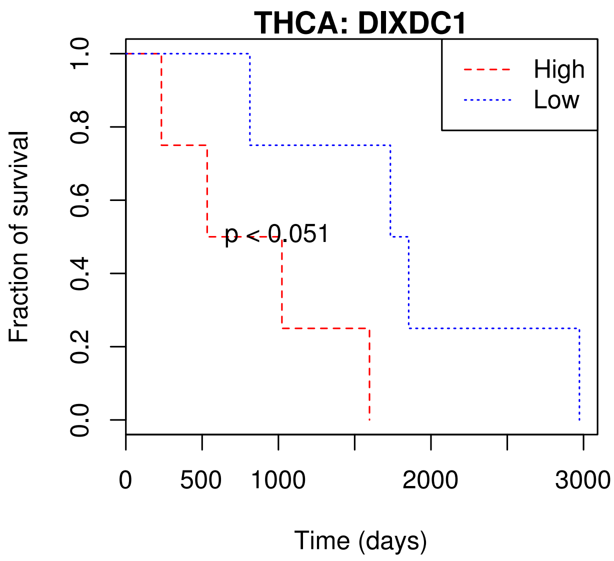
THCA: DIXDC1
High
Low
p < 0.051
Fraction of survival
0.0
0.2
0.4
0.6
0.8
1.0
0
500
1000
2000
3000
Time (days)

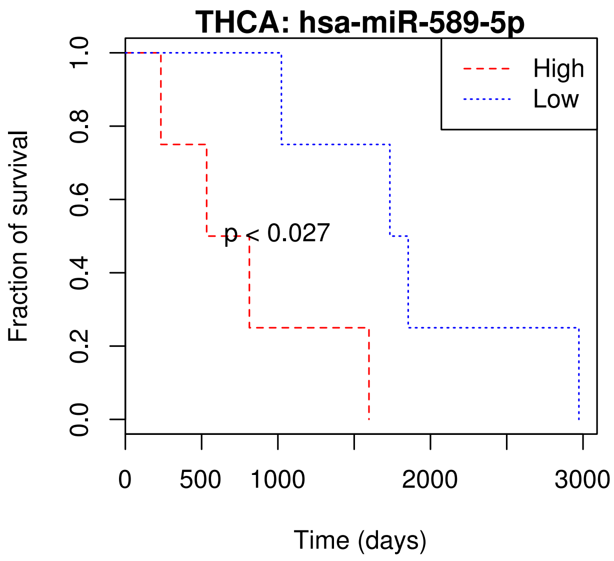
THCA: hsa-miR-589-5p
High
Low
p < 0.027
Fraction of survival
0.0
0.2
0.4
0.6
0.8
1.0
0
500
1000
2000
3000
Time (days)

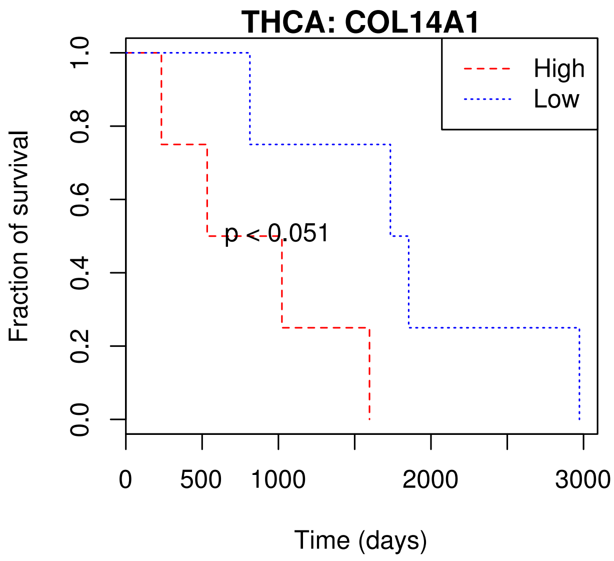
THCA: COL14A1
High
Low
p < 0.051
Fraction of survival
0.0
0.2
0.4
0.6
0.8
1.0
0
500
1000
2000
3000
Time (days)

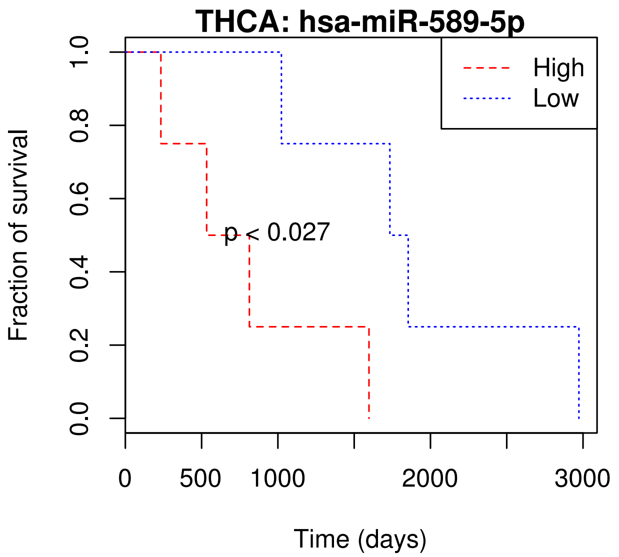
THCA: hsa-miR-589-5p
High
Low
p < 0.027
Fraction of survival
Time (days)
0.0 0.2 0.4 0.6 0.8 1.0
0 500 1000 2000 3000

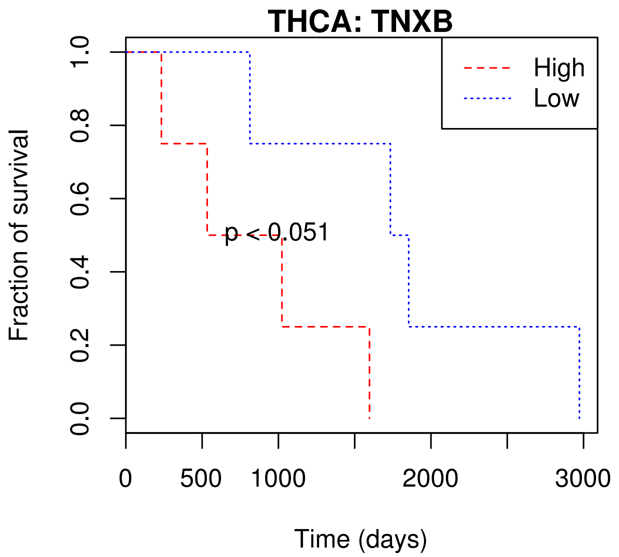
THCA: TNXB
High
Low
p < 0.051
Fraction of survival
Time (days)
0.0 0.2 0.4 0.6 0.8 1.0
0 500 1000 2000 3000

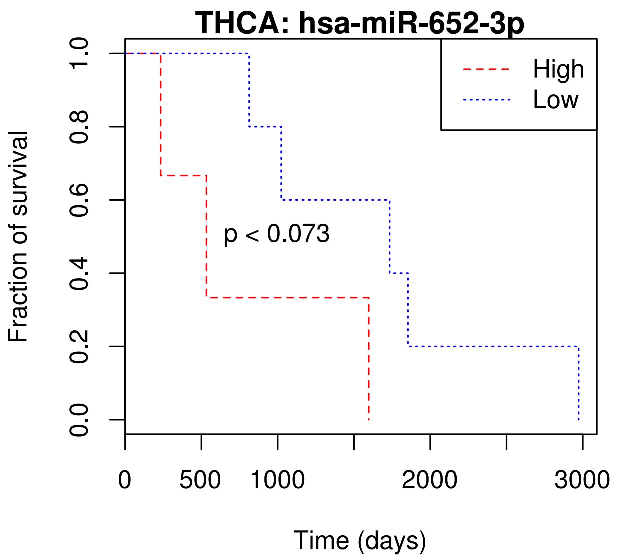
THCA: hsa-miR-652-3p
High
Low
p < 0.073
Fraction of survival
Time (days)
0.0 0.2 0.4 0.6 0.8 1.0
0 500 1000 2000 3000

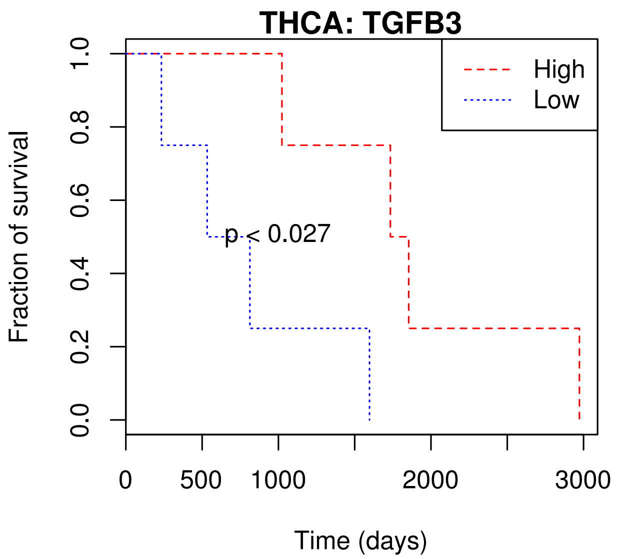
THCA: TGFB3
High
Low
p < 0.027
Fraction of survival
Time (days)
0.0 0.2 0.4 0.6 0.8 1.0
0 500 1000 2000 3000

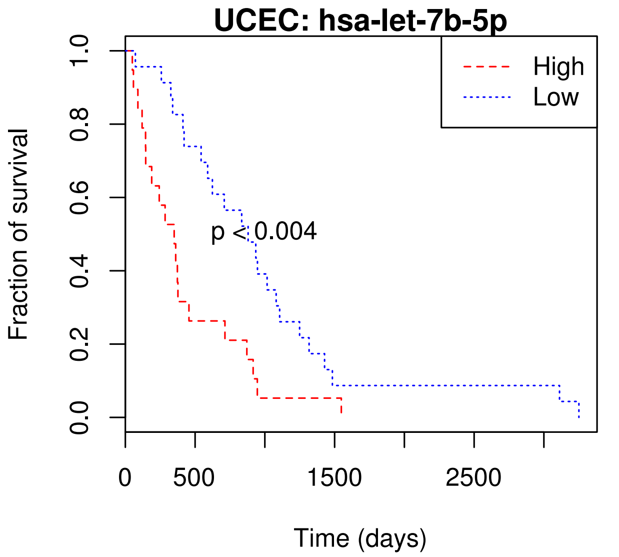
UCEC: hsa-let-7b-5p
High
Low
p < 0.004
Fraction of survival
Time (days)
0.0 0.2 0.4 0.6 0.8 1.0
0 500 1500 2500

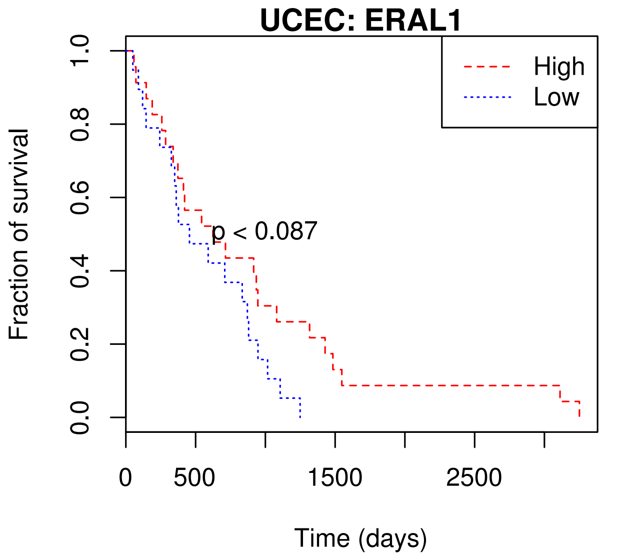
UCEC: ERAL1
High
Low
p < 0.087
Fraction of survival
Time (days)
0.0 0.2 0.4 0.6 0.8 1.0
0 500 1500 2500

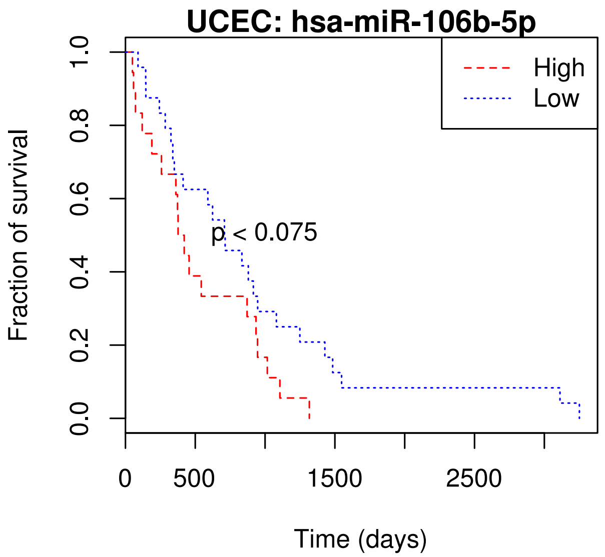
UCEC: hsa-miR-106b-5p
High
Low
p < 0.075
Fraction of survival
0.0
0.2
0.4
0.6
0.8
1.0
0
500
1500
2500
Time (days)

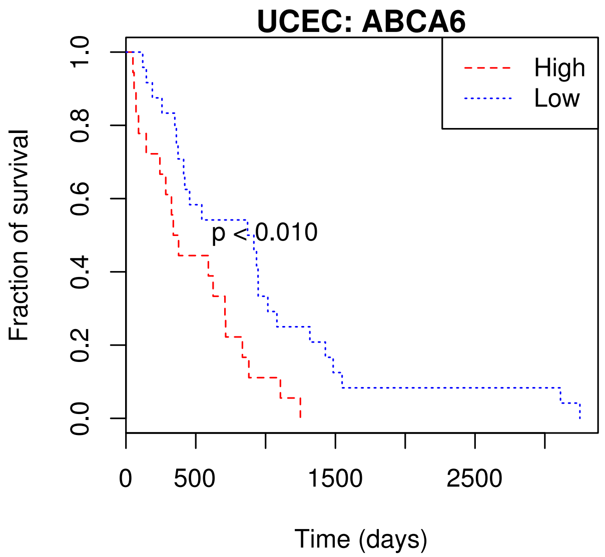
UCEC: ABCA6
High
Low
p < 0.010
Fraction of survival
0.0
0.2
0.4
0.6
0.8
1.0
0
500
1500
2500
Time (days)

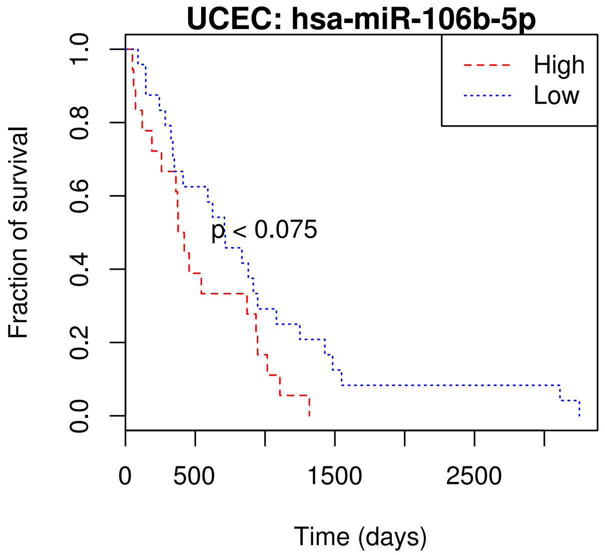
UCEC: hsa-miR-106b-5p
High
Low
p < 0.075
Fraction of survival
0.0
0.2
0.4
0.6
0.8
1.0
0
500
1500
2500
Time (days)

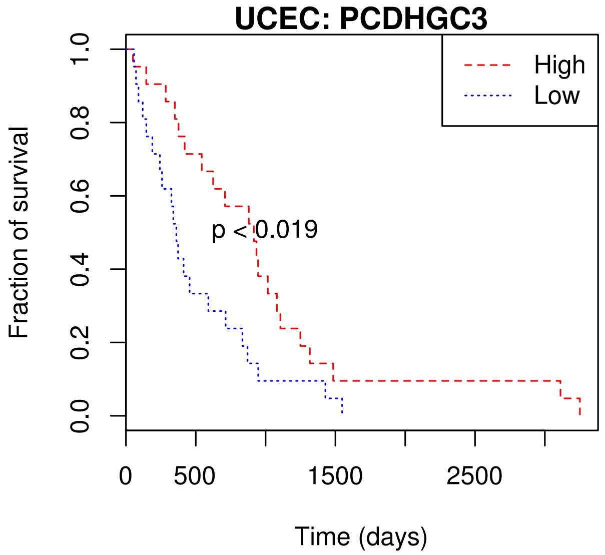
UCEC: PCDHGC3
High
Low
p < 0.019
Fraction of survival
0.0
0.2
0.4
0.6
0.8
1.0
0
500
1500
2500
Time (days)

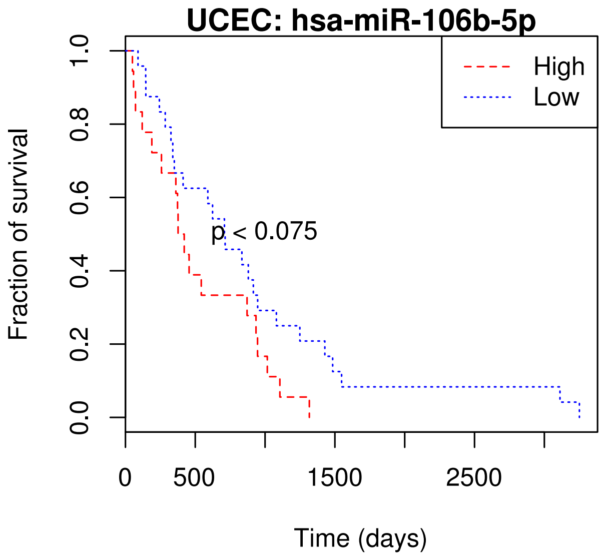
UCEC: hsa-miR-106b-5p
High
Low
p < 0.075
Fraction of survival
0.0
0.2
0.4
0.6
0.8
1.0
0
500
1500
2500
Time (days)

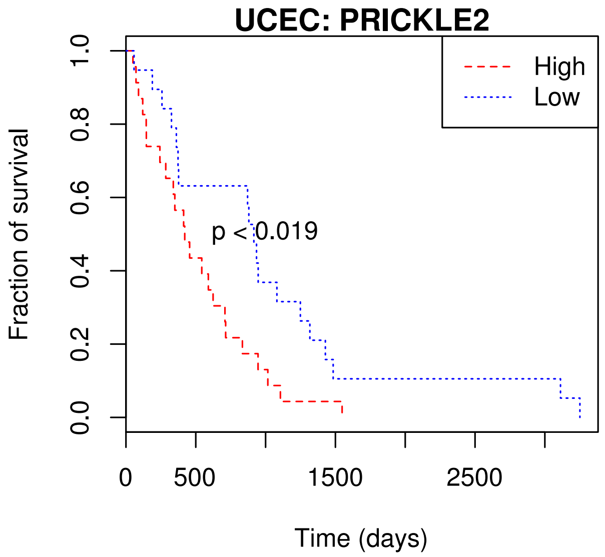
UCEC: PRICKLE2
High
Low
p < 0.019
Fraction of survival
0.0
0.2
0.4
0.6
0.8
1.0
0
500
1500
2500
Time (days)

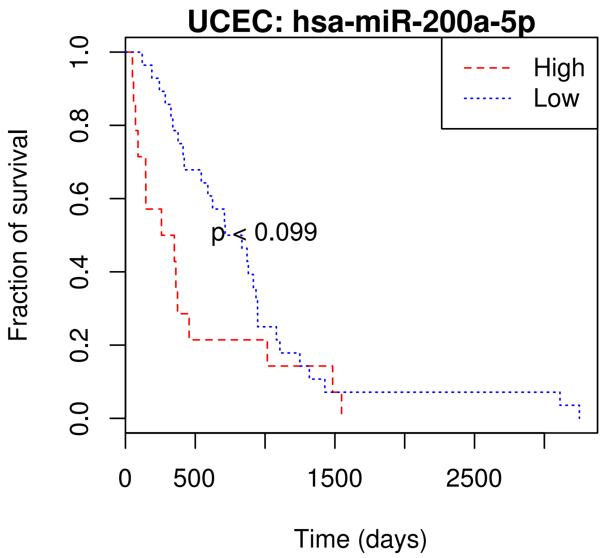
UCEC: hsa-miR-200a-5p
High
Low
p < 0.099
Fraction of survival
Time (days)
0.0 0.2 0.4 0.6 0.8 1.0
0 500 1500 2500

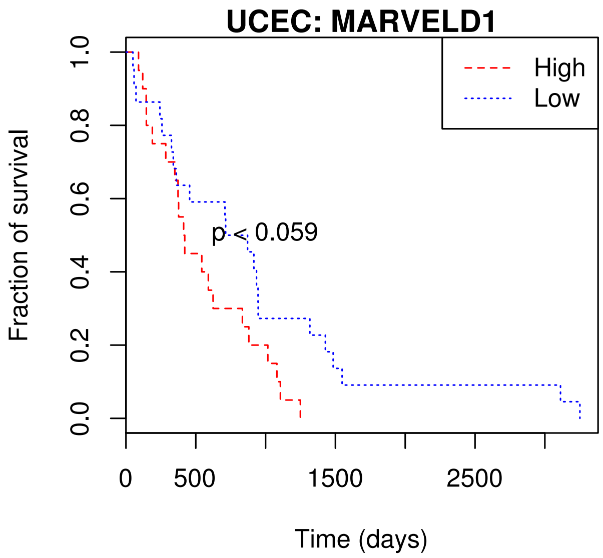
UCEC: MARVELD1
High
Low
p < 0.059
Fraction of survival
Time (days)
0.0 0.2 0.4 0.6 0.8 1.0
0 500 1500 2500

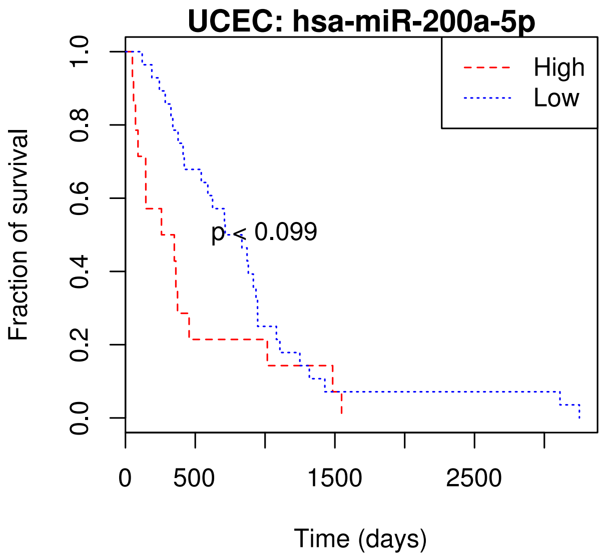
UCEC: hsa-miR-200a-5p
High
Low
p < 0.099
Fraction of survival
Time (days)
0.0 0.2 0.4 0.6 0.8 1.0
0 500 1500 2500

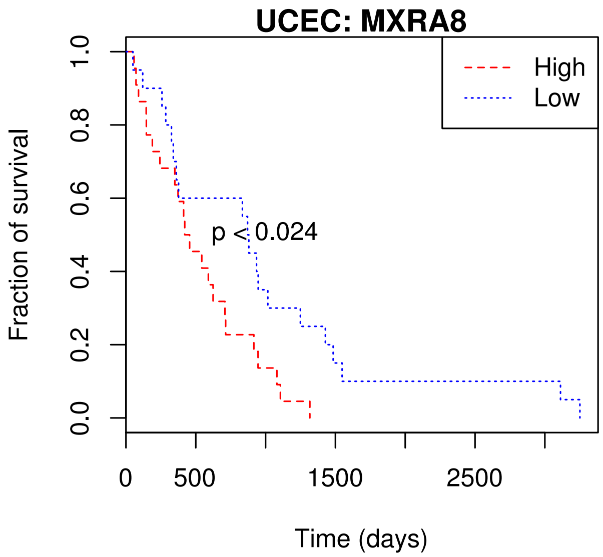
UCEC: MXRA8
High
Low
p < 0.024
Fraction of survival
Time (days)
0.0 0.2 0.4 0.6 0.8 1.0
0 500 1500 2500

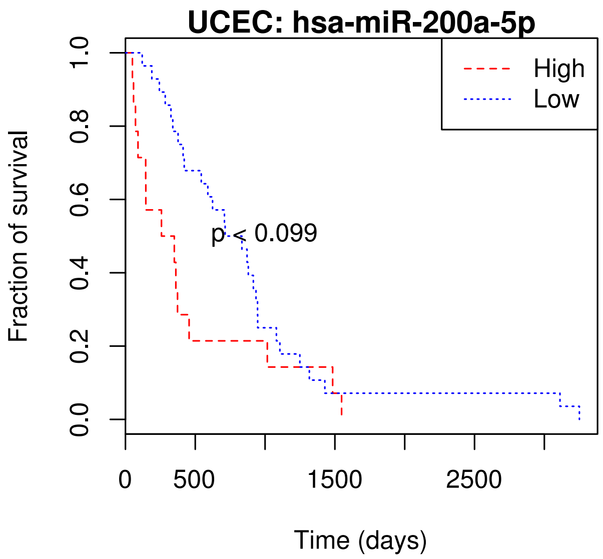
UCEC: hsa-miR-200a-5p
High
Low
p < 0.099
Fraction of survival
Time (days)
0.0 0.2 0.4 0.6 0.8 1.0
0 500 1500 2500

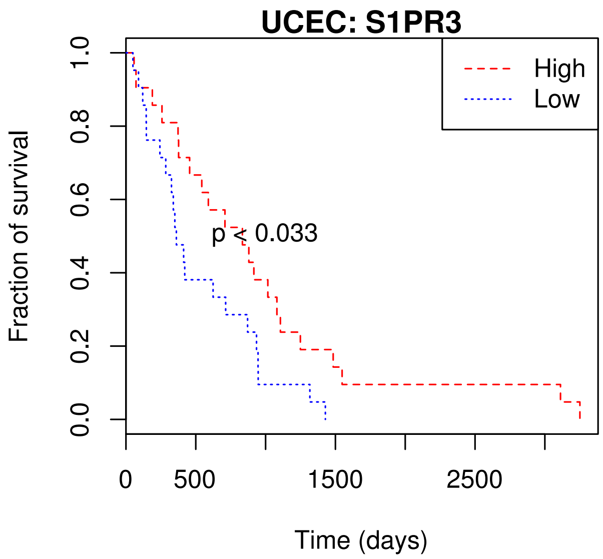
UCEC: S1PR3
High
Low
p < 0.033
Fraction of survival
Time (days)
0.0 0.2 0.4 0.6 0.8 1.0
0 500 1500 2500

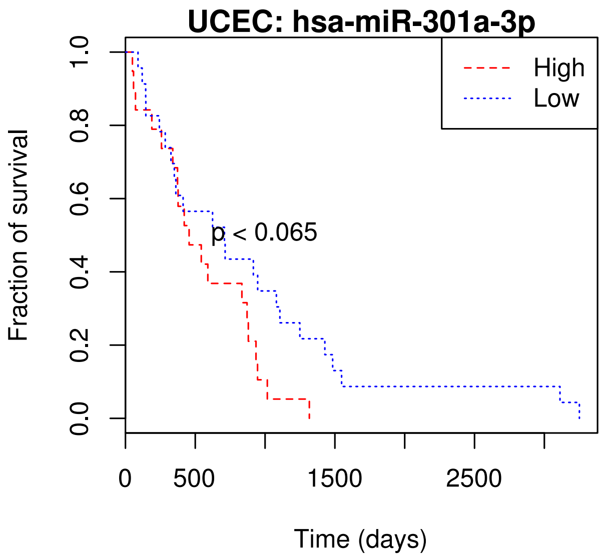
UCEC: hsa-miR-301a-3p
High
Low
p < 0.065
Fraction of survival
0.0
0.2
0.4
0.6
0.8
1.0
0
500
1500
2500
Time (days)

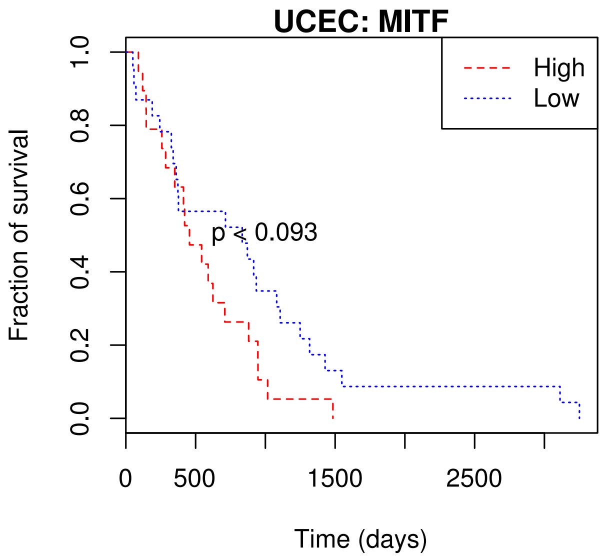
UCEC: MITF
High
Low
p < 0.093
Fraction of survival
0.0
0.2
0.4
0.6
0.8
1.0
0
500
1500
2500
Time (days)

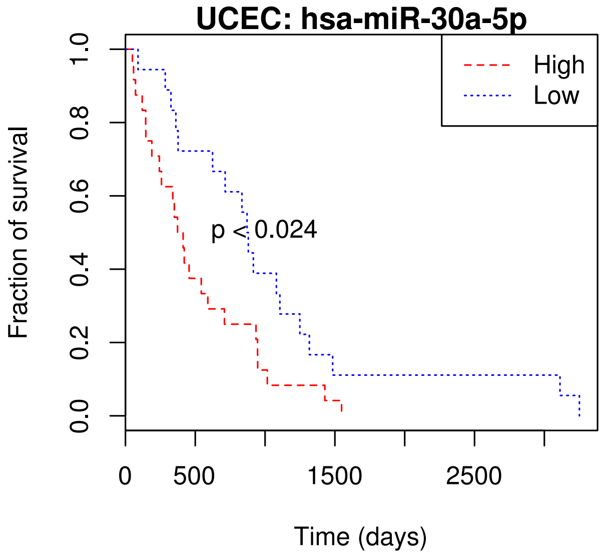
UCEC: hsa-miR-30a-5p
High
Low
p < 0.024
Fraction of survival
0.0
0.2
0.4
0.6
0.8
1.0
0
500
1500
2500
Time (days)

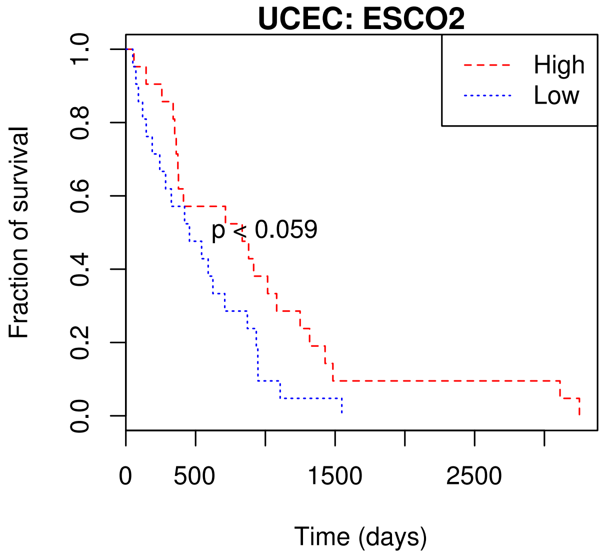
UCEC: ESCO2
High
Low
p < 0.059
Fraction of survival
0.0
0.2
0.4
0.6
0.8
1.0
0
500
1500
2500
Time (days)

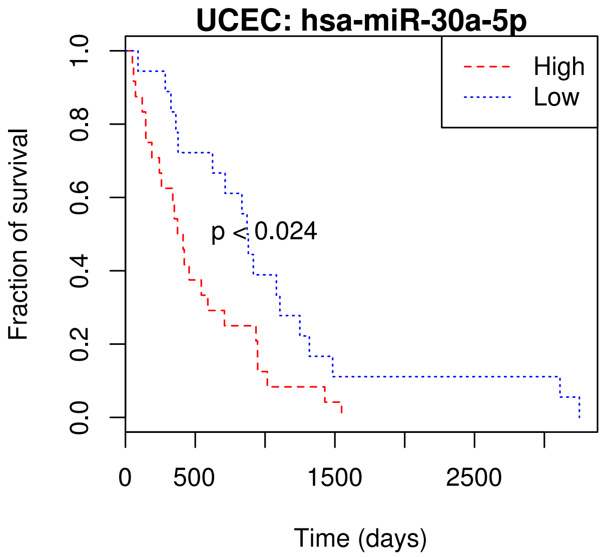
UCEC: hsa-miR-30a-5p
High
Low
p < 0.024
Fraction of survival
0.0
0.2
0.4
0.6
0.8
1.0
0
500
1500
2500
Time (days)

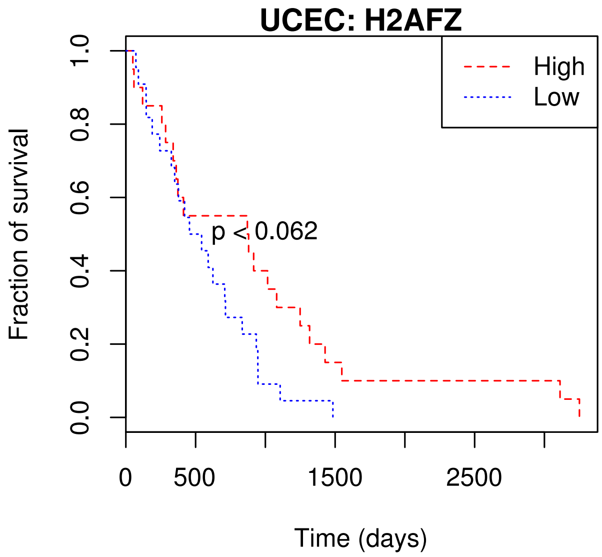
UCEC: H2AFZ
High
Low
p < 0.062
Fraction of survival
0.0
0.2
0.4
0.6
0.8
1.0
0
500
1500
2500
Time (days)

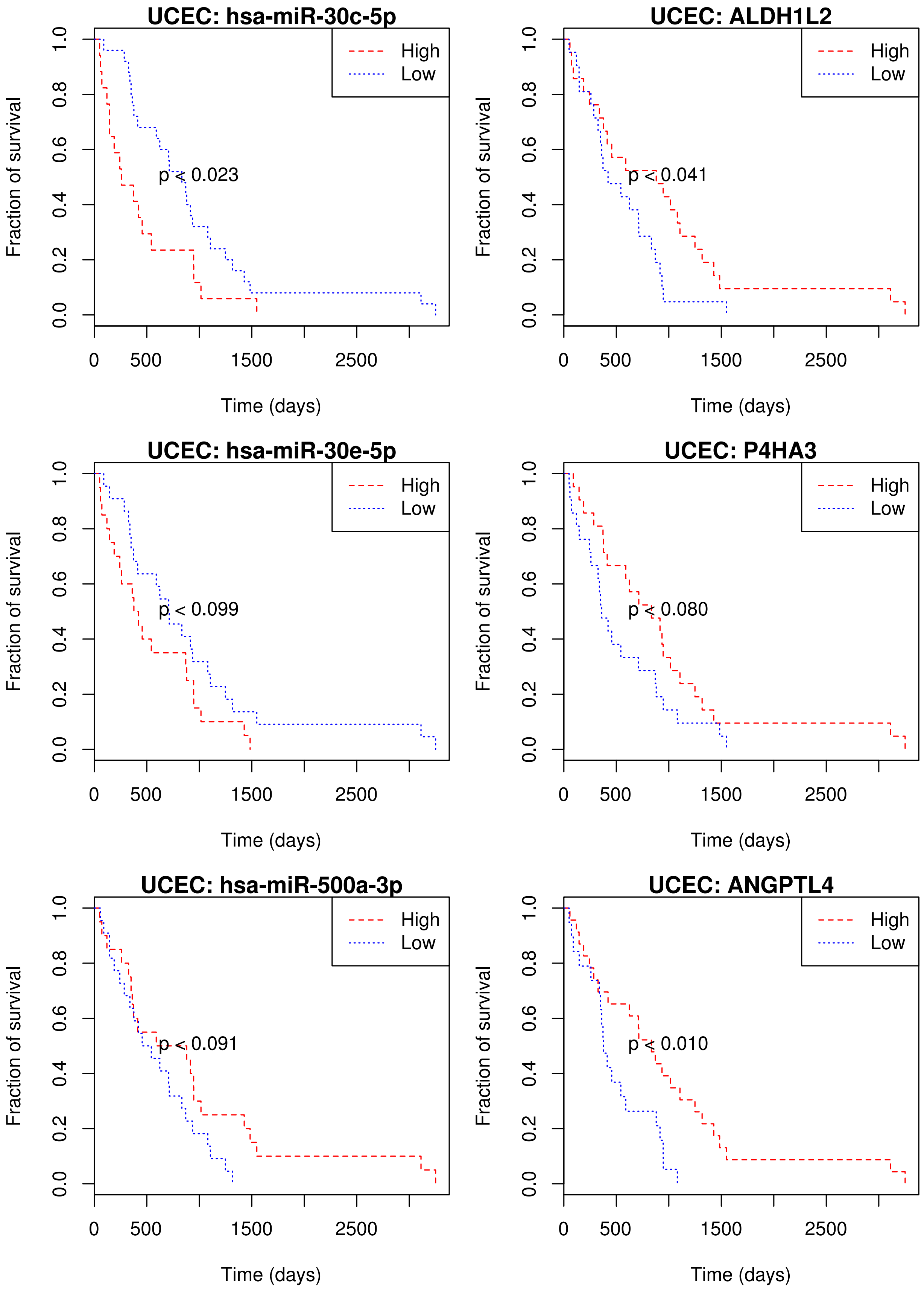
UCEC: hsa-miR-30c-5p
p < 0.023
UCEC: ALDH1L2
p < 0.041
UCEC: hsa-miR-30e-5p
p < 0.099
UCEC: P4HA3
p < 0.080
UCEC: hsa-miR-500a-3p
p < 0.091
UCEC: ANGPTL4
p < 0.010
High
Low
Fraction of survival
Time (days)
0.0 0.2 0.4 0.6 0.8 1.0
0 500 1500 2500

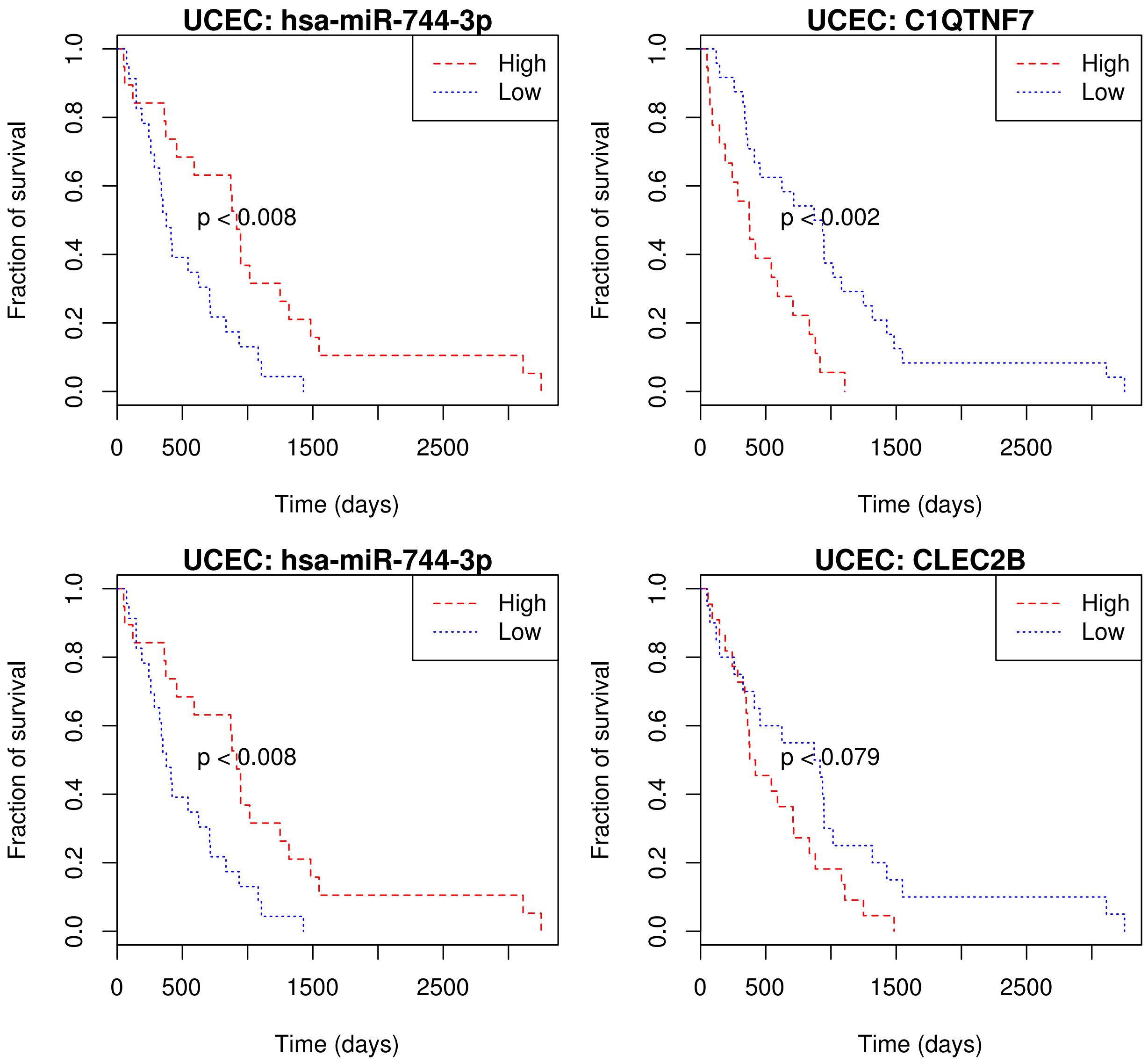

Figure S7: **Kaplan-Meier survival plots of the genes and miRNAs involved in the recurrent interactions**. Each plot display the KM survival curves for the two groups of samples with gene or miRNA expression higher and lower than the sample means. Only the recurrent interactions with both gene and miRNA exhibiting significant prognostic power for specific cancer type(s) ($p < 0.1$; log-rank test) were shown. Notably, some interactions were prominent in more than one cancer types.

Supplementary Tables

Table S1: **A full list of the positive recurrent interactions (XLS)**

Table S2: **Functional enrichments by DAVID of target genes involved in the confidence recurrent network (XLS)**

Table S3: **Survival analysis results (XLS)**. Binary value under each cancer column indicates whether the corresponding interaction (both gene and miRNA; sheet 1), gene alone (sheet 2), or miRNA alone (sheet 3) confer significant ($p < 0.1$) prognostic power (KM-survival and log-rank test).

References

1. Spizzo, R., Nicoloso, M. S., Croce, C. M. & Calin, G. A. SnapShot: MicroRNAs in Cancer. *Cell* **137**, 586–586.e1 (2009).

2. Koturbash, I., Zemp, F. J., Pogribny, I. & Kovalchuk, O. Small molecules with big effects: the role of the microRNAome in cancer and carcinogenesis. *Mutation research* **722**, 94–105 (2011).

3. Forbes, S. A. *et al.* COSMIC: mining complete cancer genomes in the Catalogue of Somatic Mutations in Cancer. *Nucleic acids research* **39**, D945–50 (2011).

4. Shannon, P. *et al.* Cytoscape: a software environment for integrated models of biomolecular interaction networks. *Genome research* **13**, 2498–2504 (2003).
